# Genome sequence, population history, and pelage genetics of the endangered African wild dog (*Lycaon pictus*)

**DOI:** 10.1186/s12864-016-3368-9

**Published:** 2016-12-09

**Authors:** Michael G. Campana, Lillian D. Parker, Melissa T. R. Hawkins, Hillary S. Young, Kristofer M. Helgen, Micaela Szykman Gunther, Rosie Woodroffe, Jesús E. Maldonado, Robert C. Fleischer

**Affiliations:** 1 Center for Conservation Genomics, Smithsonian Conservation Biology Institute, 3001 Connecticut Avenue NW, Washington, DC 20008 USA; 2 Department of Environmental Science and Policy, George Mason University, 4400 University Drive, Fairfax, VA 22030 USA; 3 Division of Mammals, National Museum of Natural History, MRC 108, Smithsonian Institution, Washington, DC 20013 USA; 4 Department of Ecology, Evolution and Marine Biology, University of California Santa Barbara, Santa Barbara, CA 93106 USA; 5 Department of Wildlife, Humboldt State University, 1 Harpst St, Arcata, CA 95521 USA; 6 Institute of Zoology, Zoological Society of London, Regent’s Park, London, NW1 4RY UK

**Keywords:** Lycaon pictus, Genome, Population history, Selection, Pelage

## Abstract

**Background:**

The African wild dog (*Lycaon pictus*) is an endangered African canid threatened by severe habitat fragmentation, human-wildlife conflict, and infectious disease. A highly specialized carnivore, it is distinguished by its social structure, dental morphology, absence of dewclaws, and colorful pelage.

**Results:**

We sequenced the genomes of two individuals from populations representing two distinct ecological histories (Laikipia County, Kenya and KwaZulu-Natal Province, South Africa). We reconstructed population demographic histories for the two individuals and scanned the genomes for evidence of selection.

**Conclusions:**

We show that the African wild dog has undergone at least two effective population size reductions in the last 1,000,000 years. We found evidence of *Lycaon* individual-specific regions of low diversity, suggestive of inbreeding or population-specific selection. Further research is needed to clarify whether these population reductions and low diversity regions are characteristic of the species as a whole. We documented positive selection on the *Lycaon* mitochondrial genome. Finally, we identified several candidate genes (*ASIP*, *MITF*, *MLPH*, *PMEL*) that may play a role in the characteristic *Lycaon* pelage.

**Electronic supplementary material:**

The online version of this article (doi:10.1186/s12864-016-3368-9) contains supplementary material, which is available to authorized users.

## Background

**Fig. 1:**
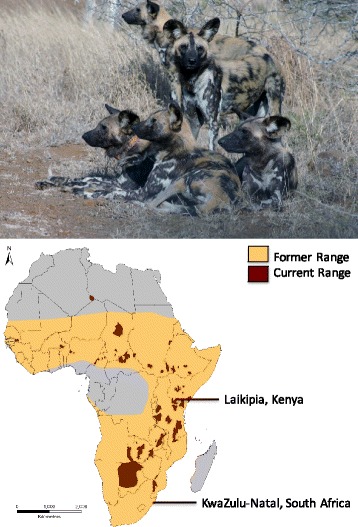
South African wild dog pack (*top*) and map of extant and former wild dog range (*bottom*). The sampling locations of the two individuals are noted on the map. Ranges are modified from Woodroffe and Sillero-Zubiri [1] and Marsden et al. [2]. Extant range data used with permission from the International Union for the Conservation of Nature [Woodroffe R, Sillero-Zubiri C 2012. *Lycaon pictus*. In: IUCN 2016. IUCN Red List of Threatened Species. Version 2016–2. http://www.iucnredlist.org. Downloaded 12 July 2016]. Photograph by Micaela Szykman Gunther

The African wild dog (*Lycaon pictus*) is an endangered canid species (International Union for Conservation of Nature Red List Classification: C2a (i)) [1]. While the species formerly ranged over most of sub-Saharan Africa, wild dogs suffer from a suite of threats including severe habitat fragmentation, human persecution, and disease epidemics. They are now restricted to less than seven percent of their former range [2], with only small, and frequently declining, remnant populations in fragmented pockets of eastern and southern Africa (Fig. 1). They maintain enormous home ranges (varying between 200 and 2000 km^2^) and naturally live at very low densities, even compared to other carnivores [3]. Primarily a hunter of antelopes, the African wild dog is a highly distinct canine. Wild dogs are differentiated from other canine species by their anatomical adaptations related to hypercarnivory and cursorial hunting, including high-crowned, sectorial teeth and the lack of dewclaws [4]. They have a highly specialized social structure in which both males and females disperse to form new packs and only a single dominant pair in each pack reproduces [5]. Wild dogs are also noted for their colorful pelage, from which they derive their species name *pictus* (‘painted’), and the absence of an undercoat.

Eastern and southern populations of wild dogs are genetically and morphologically distinct [6], although there is a large admixture zone covering Botswana, south-eastern Tanzania, and Zimbabwe [7]. Gene flow occurs across the species’ entire range [8], which is unsurprising given the excellent dispersal capabilities documented in *Lycaon pictus* [1]. Nevertheless, Marsden et al. [2] documented both genetic structuring between extant *Lycaon* populations (likely the result of habitat fragmentation) and a recent reduction in effective population size (*N*_e_) since the 1980s. Furthermore, African wild dogs exhibit very little major histocompatibility complex variation, which may reflect population decline [8].

To better understand African wild dog genomic evolution, genetic variation and population history, we shotgun sequenced whole genomes from two individuals from widely separated populations with very different modern ecological histories: Laikipia County, Kenya and Hluhluwe-Imfolozi Park, KwaZulu-Natal Province, South Africa. Wild dogs disappeared from Laikipia, Kenya in the 1980s and reappeared in 2000 – likely recolonizing from a small population in neighboring Samburu district (~50 km distant). The population in Laikipia alone now numbers more than 150 dogs and 15 packs [9]. We sequenced a female (sampled July 2003) from this recolonized population. In contrast, wild dogs were reintroduced to Hluhluwe-Imfolozi Park, KwaZulu-Natal Province in 1980 and remained as a single pack for many years [10]. Eventually, *Lycaon* breeding effectively ceased until new animals were introduced in 1997 and afterwards (2001, 2003, etc.) [11]. The KwaZulu-Natal wild dogs are now managed as part of the South African *Lycaon* “metapopulation” [12, 13]. We sequenced a male (sampled October 2007), born in KwaZulu-Natal to parents that were translocated from Limpopo province in 2003. Therefore, the South African individual’s ancestry represents genes from the northeastern part of the country.

The genomes from these two populations represent some of the first published wild canid genomes and are particularly valuable given the susceptibility of wild dogs to diseases and habitat fragmentation [9, 14]. We used our novel genome sequences to reconstruct the last 1,000,000 years of *Lycaon* genome demography and population history. We identified over a million polymorphic sequence variants for further population-level study. These variants produced ~35 million predicted genic effects. We identified over 15,000 candidate genes that may have undergone adaptation since the *Lycaon*/*Canis* divergence. We found evidence of positive selection on the *Lycaon* mitochondrial genome. Finally, we examined genes involved in canid coat phenotype to identify candidate genes underlying the characteristic *Lycaon* pelage.

## Results and discussion

### Genome sequencing of the African wild dog

Based on alignment with the domestic dog genome, we have sequenced ~90% (5.8× mean read depth) of the Kenyan individual’s genome and ~93% (5.7×) of the South African *Lycaon* individual’s genome. We identified 16,967,383 autosomal sequence variants (including 14,360,480 single nucleotide polymorphisms [SNPs] and 2,606,903 indels) separating our *Lycaon* genomes from the domestic dog (*Canis familiaris*) genome [GenBank: CanFam3.1] (Additional files 1 and 2) [15]. Of these, 1,092,450 (781,329 SNPs and 311,121 indels) were polymorphic in the African wild dog. The remaining 15,874,933 autosomal sequence variants (13,579,151 SNPs and 2,295,782 indels) were monomorphic in the two *Lycaon* individuals. We identified 717,870 X-chromosomal variants (619,606 SNPs and 98,264 indels), of which 32,801 (23,001 SNPs and 9,800 indels) were polymorphic and 685,069 (596,605 SNPs and 88,464 indels) were monomorphic in the African wild dog. Additionally, we sequenced the Kenyan and South African wild dog mitochondrial genomes to depths of 943× and 1021×, respectively. We annotated the *Lycaon* autosomal and X-chromosomal sequences using the domestic dog genome annotations [GenBank:CanFam3.1.81] and the mitochondrial genome [GenBank:NC_002008.4] and MSY sequences [GenBank:KP081776.1] using their reference sequence annotations [15–17].

### *Lycaon* demographic history

**Fig. 2:**
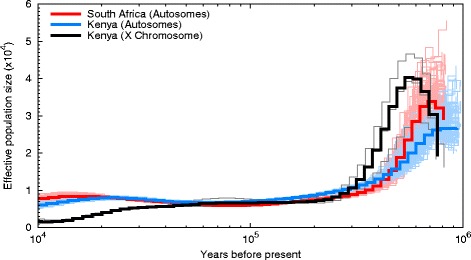
Reconstruction of the *Lycaon* individuals’ autosomal and X-chromosomal demographic history using the pairwise sequentially Markovian coalescent. Initial results are plotted using dark-colored curves, with the bootstrap replicates plotted in lighter hues of the corresponding colors

We analyzed the two wild dogs’ autosomal population histories using PSMC (Fig. 2) [18]. Both genomes exhibited a strong reduction in *N*_e_ starting 700,000 years ago from maximum *N*_e_s of ~28,000 (Kenyan) and ~35,000 (South African) individuals and leveling off 200,000 years ago at *N*_e_s of ~7,000 individuals each. Analysis of the Kenyan individual’s X - chromosomal history using PSMC showed a similar pattern (Fig. 2). *N*_e_ fell from a maximum of ~40,000 individuals 600,000 years ago to ~7000 individuals 200,000 years ago. This *N*_e_ reduction may represent a past population bottleneck e.g. [19] or lineage splitting e.g. [20]. Further genomic analysis of individuals from across the *Lycaon* range, especially those from larger founder populations, would help clarify the cause of this pattern.

PSMC analysis of the Kenyan X chromosome revealed a secondary reduction in *N*_e_ started 70,000 years ago to a final *N*_e_ of ~2000 individuals 10,000 years ago. We are unable to infer more recent population history accurately using this method due to the limited numbers of available mutations in the short time frame [18]. Further research using historical museum *Lycaon* specimens would fill in this temporal gap e.g. [21]. While our reconstructions were robust to coverage variation (see ‘Demographic history reconstruction’ below), higher coverage genomes would also increase the resolution of population history reconstructions [20].

### Candidate selected processes and genomic regions

Based on the domestic dog genome annotations, SnpEff 4.1 L predicted that the identified *Lycaon* autosomal and X-chromosomal sequence variants would cause 34,001,288 and 36,362,161 genic effects in the Kenyan and South African individuals, respectively (Additional files 1 and 2) [22]. The majority of sequence variant effects fell within introns (Kenyan: 62.604%, South African: 63.383%) and intergenic regions (Kenyan: 23.081%, South African: 23.162%). To discover candidate genes that have diverged since *Lycaon*’s divergence with *Canis*, we identified *Lycaon* genes that contained missense mutations and stop codon gains using SnpSift 4.1 L [23]. We identified 15,611 (15,565 genes with missense mutations and 799 with stop codon gains) Kenyan and 9793 (9440 genes containing missense mutations and 741 with stop codon gains) South African wild dog candidate divergent genes. 9506 divergent genes (9159 with missense mutations and 653 with stop codon gains) were found in both wild dogs. These divergent genes were determined by 47,059 Kenyan sequence variants (sequenced at 8.6× mean coverage) of and 27,893 South African variants (6.0× mean coverage). 25,149 variants were shared between the two *Lycaon* individuals. These variants were very homozygous (Kenyan: 95%, South African: 95%), which suggests that they are the result of divergence between the *Lycaon* and *Canis* clades, rather than more recent variants arising within *Lycaon*.

We annotated the candidate divergent genes’ functions using DAVID 6.7 with domestic dog (option “*Canis lupus*”) as the genomic background [24]. We found 76 and 29 enriched processes in the Kenyan and South African individuals, respectively (Additional files 3 and 4). We filtered these terms with a Benjamini-Hochberg false discovery rate of 0.05 [25]. After filtration, seven terms (‘Complement and coagulation cascades’, ‘ECM-receptor interaction’, ‘Neuroactive ligand-receptor interaction’, ‘Hematopoietic cell lineage’, ‘Lysosome’, ‘ABC transporters’, ‘Aminoacyl-tRNA biosynthesis’) were significantly enriched in the Kenyan individual. ‘Olfactory transduction’ was significant in the South African individual. The differences in significant terms may reflect population-specific selection pressures on wild dogs. Further population-level investigation is needed to determine the roles these pathways play in *Lycaon* evolution.

**Fig. 3:**
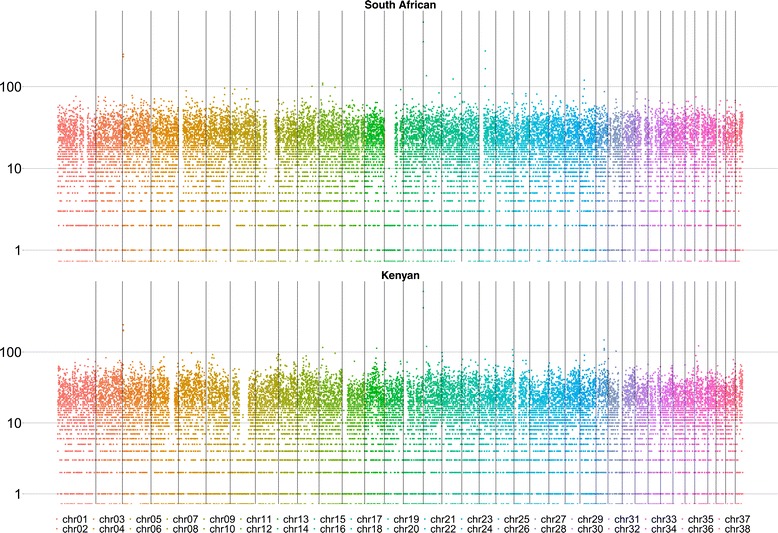
Segregating autosomal SNP sites across the *Lycaon* genomes. Chromosomes are distinguished by color and separated by *black lines*. The number of segregating SNPs per 100,000 bp window is plotted on the y-axis in logarithmic scale. We identified population-specific regions of low diversity in both the Kenyan (chromosomes 4, 6, 7, 12, 15, 21, 27, and 30) and South African (chromosomes 1, 5, 8, 12, 14, 19, 27, 29, 30, 34, 36 and 38) individuals. There are also highly variable regions on chromosomes 3 and 16 in both individuals, chromosome 26 in the Kenyan individual, and chromosome 19 in the South African individual

In order to identify regions of low and high diversity, we calculated the numbers of segregating SNP sites across the *Lycaon* autosomes in 100,000 bp non-overlapping windows using VCFtools 0.1.15 (Fig. 3) [26, 27]. By averaging over large genomic windows, we limited the effects of sequencing errors and allelic drop-out due to low sequencing coverage. We identified 768,416 segregating *Lycaon* SNPs (Kenyan: 398,891 SNPs, South African: 434,911 SNPs). We observed individual-specific regions of low diversity (<10 segregating SNPs/100,000 bp). The Kenyan individual had runs of low diversity (contiguous regions of low diversity at least 5 million bp long) on chromosomes 4, 6, 7, 12, 15, 21, 27, and 30, while the South African individual had runs of low diversity on chromosomes 1, 5, 8, 12, 14, 19, 27, 29, 30, 34, 36 and 38. These low-diversity regions may be the result of inbreeding and/or population-specific natural selection. Due to the long lengths of these low-diversity runs, encompassing numerous genes, we are not currently able to link low diversity levels to selection on individual genes. These results are not surprising since both populations are recently re-established, either by natural recolonization (Laikipia, Kenya) or deliberate reintroduction (KwaZulu-Natal, South Africa). Previous genetic investigations using microsatellites and mitochondrial DNA found some evidence of rare inbreeding in wild dogs from the Greater Limpopo Transfrontier Conservation Area and KwaZulu-Natal [10, 28]. However, free-ranging wild dogs strongly avoid incestuous matings [10]. For instance, at KwaZulu-Natal, Becker et al. [10] observed only one of six breeding pairs being more closely related than third-order kin. While our chromosomal diversity data do not permit us to discern between inbreeding and/or population-specific natural selection, the possibility of inbreeding is, therefore, concerning from a conservation standpoint. Additional population-level data are required to determine the causes and effects of these low-diversity regions.

We also identified regions of high diversity (>200 segregating SNPs per 100,000 bp) on chromosomes 3 and 16 in both individuals and chromosome 19 in the South African individual (Fig. 3). The high-diversity regions comprised 0.44% of the total segregating SNPs in both individuals and included 1138 Kenyan and 970 South African segregating SNPs on chromosome 3,646 Kenyan and 704 South African segregating SNPs on chromosome 16, and 249 South African segregating SNPs on chromosome 19. Using the UCSC Genome Browser [29], we scanned the genome within 100,000 bp upstream and downstream of these high-diversity regions for known genes mapped to CanFam3.1. These included *FER*, *FKBP3*, *SNRPF*, and *PJA2* on chromosome 3, *HUS1* and *PKDIL1* on chromosome 16, and *RNF150* and *TBC1D9* on chromosome 19. These genic regions may have undergone positive or relaxed selection since the *Lycaon*/*Canis* divergence or may represent chromosomal duplications. Future functional analyses will determine which roles (if any) these genes play in *Lycaon* evolution.

### Positive selection on the mitochondrial genome

Each of the 13 protein-encoding mitochondrial genes from the two novel *Lycaon* mitochondrial genomes were aligned against the corresponding genic sequences from the domestic dog mitochondrial reference sequence and the one publically available near-complete *Lycaon pictus* mitochondrial genome sequence [GenBank:KT448283.1] [16, 30]. We calculated ratios of non-synonymous substitutions per non-synonymous site to synonymous substitutions per synonymous site (*dN*/*dS*) and tested for positive selection using the codon-based *Z*-test in MEGA6 [31]. We found evidence for positive selection on 12 of 13 genes (*COX1*, *COX2*, *COX3*, *ATP6*, *ATP8*, *ND1*, *ND2*, *ND3*, *ND4*, *ND4L*, *ND5*, *CYTB*; Additional file 5). To determine whether selection occurred primarily on the *Lycaon* or *Canis* branches, we reran these analyses excluding the domestic dog sequence. We found evidence for positive selection on all 13 *Lycaon* mitochondrial genes (Additional file 5).

To confirm branch specific signatures of selection, we aligned the *Lycaon CYTB* sequences against published canid taxa [GenBank: AY656746, EU442884, GU063864, JF342908, KF646248, KT448273–4, KU696390, KU696404, KU696408, NC_008093, NC_013445] using MAFFT 7.222 [32] as implemented in Geneious 10.0.2 (Biomatters, Ltd., Auckland, New Zealand). We then generated a phylogenetic tree with FastTree 1.0 [33]. Using the ‘codonml’ algorithm in PAML 4.8 [34], we performed pairwise comparisons on *CYTB* codon data and compared the likelihood of the alternate (non-fixed omega values) or null hypotheses (fixed omega values). A likelihood ratio test was calculated from ln values obtained from these comparisons to determine where evidence of selection was occurring in canids. We found significant evidence (*p* ≤ 0.001) of positive selection between African wild dogs and coyotes (*Canis latrans*) (statistic: 128.88) and between *Lycaon* and *Cuon* (statistic: 81.48). We also found branch-specific selection between the Kenyan and South African *Lycaon* individuals (statistic: 92.54).

The 13 candidate selected *Lycaon* mitochondrial genes are involved in the electron transport chain and adenosine triphosphate synthesis. This suggests natural selection on *Lycaon* metabolic processes e.g. [35, 36], which is likely given their unique antelope hunting strategies and diet. Moreover, these results are consistent with African wild dogs’ very high metabolic rate and hunting energy expenditure in comparison to domestic dogs [37].

### Pelage genes

We extracted CDS corresponding to 11 genes involved in canid coat color (agouti signaling peptide [*ASIP*], β-defensin 103 [*DEFB103A*], melanocortin 1 receptor [*MC1R*], melanophilin [*MLPH*], microphthalmia-associated transcription factor [*MITF*], premelanosome protein [*PMEL*], tyrosinase-related protein 1 [*TYRP1*]) and type (fibroblast growth factor 5 [*FGF5*], keratin 71 [*KRT71*], R-spondin 2 [*RSPO2*]) [38–46] using Geneious 9.0.4. In cases where there were multiple isoforms or CDS annotations (*DEFB103A*, *FGF5*, *MITF*, *PMEL*), we chose the longest variant alignable to the domestic dog CDS reference sequence to maximize detection power. To detect positively selected genes, we identified non-synonymous and synonymous SNPs compared to the domestic dog sequence and calculated the non-synonymous/synonymous (*N*/*S*) ratio (Additional file 6).

*ASIP* and *PMEL* had elevated *N*/*S* ratios suggestive of positive selection (5.00 and 9.00 respectively). *Lycaon PMEL* also had a stop codon gain at amino acid 341, suggesting selection at this locus. Additionally, we found a threonine insertion at amino acid 371 in the *Lycaon MLPH* gene and a six amino acid deletion corresponding to domestic dog amino acids 186–191 in the *Lycaon MITF* gene. To further characterize these four candidate genes, we compared the *Lycaon* coding sequences against all publically available canid sequences using BLAST+ 2.5.0 [47]. None of the *Lycaon ASIP*, *PMEL*, *MITF*, and *MLPH* CDS haplotypes have been identified in other canids previously. *Lycaon ASIP* shares 99% nucleotide identity, but only 96% amino acid identity, with domestic dogs. Wild dog *PMEL* shares both 99% nucleotide identity and amino acid identity with domestic dogs. Excluding the six amino acid deletion, *Lycaon MITF* haplotypes had >99% nucleotide and amino acid identity with domestic dogs. *Lycaon MLPH* haplotypes had 98% nucleotide identity and 97–98% amino acid identity (excluding the amino acid insertion) to domestic dogs.

These four genes are strong candidates to explain the characteristic *Lycaon* pelage. Mutations in *ASIP* alter relative production of eumelanin and pheomelanin, resulting in lighter and darker hair colors, in numerous species including domestic dogs [41]. Variants in *PMEL* cause merle patterning in domestic dogs [40]. *MITF* is associated with white-spotting phenotypes in domestic dogs and causes coat color variants in laboratory mice (*Mus musculus*). *MITF* variants are also associated with deafness, small eye size, and poor bone resorption in mice [46]. Nevertheless, further laboratory assays (such as transgenic experiments) are needed to confirm that these identified variants are functional and to determine their phenotypic effects. Furthermore, our data do not permit us to distinguish between positive selection on the *Lycaon* and *Canis* branches (e.g. coat variation associated with dog domestication and breed development).

## Conclusions

We provide two genome sequences of *Lycaon pictus*, representing two individuals from highly divergent ecological regions (Laikipia County, Kenya and KwaZulu-Natal Province, South Africa). We identified over a million polymorphic *Lycaon* SNPs, useful for further population-level analyses. Analyses of these genomes showed that extant *Lycaon* populations have endured at least two population contractions within the last 1,000,000 years. We identified chromosomal regions of high and low diversity and over 15,000 candidate divergent genes. Furthermore, *Lycaon* mitochondrial genomes have undergone positive selection, suggestive of selection for metabolic processes. Finally, we identified four candidate genes (*ASIP*, *MITF*, *MLPH*, *PMEL*) that may be involved in *Lycaon* pelage patterns.

## Methods

### Samples

We sequenced two *Lycaon pictus* individuals sampled during previous studies: a female from Laikipia County, Kenya [2, 9] and a male from Hluhluwe-Imfolozi Park, KwaZulu-Natal Province, South Africa [10, 48, 49]. The Kenyan individual was sampled under an Institutional Animal Care and Use Committee (IACUC) protocol approved by the University of California, Davis (10813) [9], while the South African individual was sampled under IACUC protocols approved by the Smithsonian National Zoological Park (08-21) and Humboldt State University (06/07.W.209.A) [10].

### Laboratory methods

DNA was extracted from blood samples using Qiagen blood and tissue kits (Qiagen, Valencia, CA, USA) and sheared to ~350 bp using a Q800R sonicator (Qsonica, LLC, Newtown, CT, USA). Double-indexed Illumina libraries were built from the sheared DNA using the KAPA Library Preparation Kit – Illumina (Kapa Biosystems, Wilmington, MA, USA) with purification steps performed using carboxyl paramagnetic beads [50]. Library quality was ensured via fluorometric analysis using Qubit® dsDNA HS assays (Life Technologies, Carlsbad, CA, USA), quantitative PCR using the KAPA Library Quantification Kit – Illumina/Universal (Kapa Biosystems, Wilmington, MA, USA), and analysis on a 2100 Bioanalyzer (Agilent Technologies, Santa Clara, CA, USA) high-sensitivity DNA chip. Libraries were pooled equimolarly and 2 × 250 bp paired-end sequenced on a HiSeq 2500 lane (Illumina, Inc., San Diego, CA, USA).

### Sequence quality control

Read pairs were demultiplexed using the BaseSpace® pipeline (Illumina, Inc., San Diego, CA, USA). 75,651,396 and 63,766,266 read pairs were generated for the Kenyan and South African *Lycaon* samples respectively. Raw reads were trimmed and adapter artifacts were removed using Trimmomatic 0.33 (options ILLUMINACLIP: NexteraPE-PE.fa:2:30:10 LEADING:3 TRAILING:3 SLIDINGWINDOW:4:20 MINLEN:36) [51]. Library sequence quality was confirmed using FastQC 0.11.2 [52].

### Mitochondrial genome assembly

The quality-controlled reads were aligned against the circularized domestic dog reference mitochondrial genome [GenBank:NC_002008.4] [16] using Geneious 8.1.6 (medium-low sensitivity, 5 alignment iterations, minimum mapping quality 30). The aligned reads were merged using FLASH 1.2.11 (option –M 250) [53]. PCR duplicates were removed from the merged reads using CD-HIT-DUP 0.5 [54]. The deduplicated reads were then realigned against the dog reference mitochondrial genome in Geneious 8.1.6 (medium sensitivity alignment, 10 alignment iterations, minimum mapping quality 30) to generate the final sequences.

### Autosomal assembly

The non-mitochondrial reads were merged using FLASH 1.2.11 (option –M 250) [51]. The merged, unmerged, and unpaired reads were then concatenated and treated as single-end sequences for downstream processing. The concatenated reads were aligned against the autosomes of the domestic dog genome build CanFam3.1 [15] using the ‘mem’ algorithm in BWA 0.7.12 [55, 56]. Aligned reads below mapping quality 30 and PCR duplicates were removed using the ‘view’ (option –q 30) and ‘rmdup’ (option –s) commands in SAMtools 1.3 [57]. Sequence variants (minimum quality 20) were identified using the SAMtools 1.3 ‘mpileup’ command (option –C50) and BCFtools 1.3 ‘call’ command (option –m) pipeline [57, 58]. Genome completeness was evaluated using BUSCO 1.1b1 and the ‘Vertebrates’ gene set (Additional files 7, 8 and 9) [59]. Genome assemblies were very complete: compared to the 1663 ‘complete’ BUSCO autosomal orthologs found in the domestic dog, 1614 (97%) of the Kenyan and 1665 (100.1%) of the South African individual’s orthologs were complete. Similarly, 747 ‘fragmented’ BUSCO autosomal orthologs were found in the domestic dog, compared to 688 (92.1%) for the Kenyan individual and 707 (94.6%) for the South African individual.

### Allosomal assembly

The Kenyan *Lycaon* individual was a female, while the South African individual was a male. To reconstruct the allosomal sequences, we aligned the unmapped, concatenated nuclear reads against either the CanFam3.1 X chromosome assembly (Kenyan individual) [15] or both the CanFam3.1 X chromosome and domestic dog MSY chromosome assemblies (South African individual) [17] using the ‘mem’ algorithm in BWA 0.7.12 [55, 56]. Aligned reads below mapping quality 30 and PCR duplicates were removed using the ‘view’ (option –q 30) and ‘rmdup’ (option –s) commands in SAMtools 1.3 [57]. Sequence variants (minimum quality 20) were identified using the SAMtools 1.3 ‘mpileup’ command (option –C50) and BCFtools 1.3 ‘call’ command (option –m) pipeline [57, 58]. The mapped MSY reads were then realigned against the reference sequence [GenBank:KP081776.1] using Geneious 8.1.7 (medium sensitivity alignment, five alignment iterations). Y coding region sequences were extracted based on the domestic dog MSY annotations, and consensus sequences were generated using Geneious 8.1.7 (options Highest Quality and Total). We excluded non-coding regions from analysis due to the Y chromosome’s large number of repetitive elements, which complicates accurate alignment [17]. We identified 87 Y SNPs between the African wild dog and the domestic dog, of which 32 were silent mutations, 53 produced amino acid substitutions, and two caused gene truncations (Additional file 10).

### Demographic history reconstruction

Autosomal population history parameters were reconstructed using PSMC r62 (options –N25 –t15–r5 –p “64*1”, minimum quality 20) and tested with 100 bootstrap replicates [18]. We calculated the autosomal mutation rate for each *Lycaon* individual using the total number of identified autosomal sequence variants (13,985,381 and 15,132,667 for the Kenyan and South African individuals respectively), an estimated autosomal genome size of 2.3 Gbp, an estimated generation time of 5 years/generation [7] and an estimated divergence time from the *Canis*/*Cuon* clade of 2.74 million years ago (95% highest posterior density: 2.15–3.38 million years ago) [30]. We estimated the Kenyan and South African *Lycaon* mutation rates as 5.5 × 10^−9^ mutations/site/generation (range: 5.0–7.1 × 10^−9^ mutations/site/generation) and 6.0 × 10^−9^ mutations/site/generation (range: 4.9–7.7 × 10^−9^ mutations/site/generation) respectively. Final PSMC demographic reconstructions were scaled based on an estimated generation time of 5 years/generation [7] and a mutation rate of 5.8 × 10^−9^ mutations/site/generation. While variation of the reconstruction scaling within the extremes of the estimated mutation rates (4.9–7.7 × 10^−9^ mutations/site/generation) varied estimates of *N*_e_ and timing of population history events, overall demographic history patterns remained similar.

The Kenyan X-chromosomal history was reconstructed separately from the autosomal history. We calculated the X-chromosomal mutation rate for the Kenyan individual using the total number of observed Kenyan X-chromosomal variants (634,216), the same divergence and generation times as for the autosomal analyses and a chromosome size of 124 Mbp. We estimated the Kenyan X-chromosomal mutation rate as 4.7 × 10^−9^ mutations/site/generation (range: 3.8–5.9 × 10^−9^ mutations/site/generation). We did not estimate the X-chromosomal mutation rate for the South African male due to his hemizygosity. X-chromosomal PSMC demographic reconstruction and scaling parameters were the same as for the autosomal analyses except that the results were scaled with a mutation rate of 4.7 × 10^−9^ mutations/site/generation. Variation of the mutation rate scaling again did not affect inference of demographic history.

To test the effects of coverage on our demographic reconstructions, we repeated the PSMC analyses under medium depth stringency settings (minimum sequencing depth 2, maximum sequencing depth 12) recommended by PSMC’s authors and high depth stringency settings (minimum sequencing depth 10) recommended in [20]. We recovered nearly identical demographic reconstructions under the medium depth stringency settings (Additional file 11). Under the high depth stringency settings, we were unable to resolve the Kenyan X-chromosomal reconstruction due to missing data. Nevertheless, our autosomal reconstructions under the high depth stringency recovered very similar demographic histories for the last 1,000,000 years, except that *N*_e_ estimates were larger (particularly in the South African individual) (Additional file 12). Increased *N*_e_ estimates are expected since heterozygotes are more likely to be observed in the higher coverage regions [20]. Therefore, we conclude that our reconstructed demographic patterns were robust to coverage variation. Interestingly, under the high depth stringency settings, we also reconstructed two additional contraction and expansion cycles between 10,000,000 and 600,000 years ago in the South African individual’s population history (Additional file 12). These results require further verification with additional genome sequences.

## Additional files

Additional file 1: Genic effects of SnpEff-annotated autosomal and X-chromosomal variants for the Kenyan *Lycaon* individual. (HTML 1596 kb)
Additional file 2: Genic effects of SnpEff-annotated autosomal and X-chromosomal variants for the South African *Lycaon* individual. (HTML 1535 kb)
Additional file 3: DAVID-annotated enriched functional processes for the Kenyan *Lycaon* individual. For each functional term, the total gene count, the associated genes, and the Benjamini-Hochberg false discovery rate value are given. (XLSX 23 kb)
Additional file 4: DAVID-annotated enriched functional processes for the South African *Lycaon* individual. For each functional term, the total gene count, the associated genes, and the Benjamini-Hochberg false discovery rate value are given. (XLSX 16 kb)
Additional file 5: Positive selection test results on the *Lycaon* mitochondrial genome. *Z* statistics are included in parentheses after the probability for each of the three competing models (Neutrality, Positive Selection, Purifying Selection). Results for the tests excluding the domestic dog sequence are denoted by ‘*Lycaon*’ in the column heading. (XLSX 48 kb)
Additional file 6: Positive selection scan results on candidate *Lycaon* pelage genes. Genes were aligned to the reference CDS provided in the table. Gene coordinates are given with reference to the domestic dog genome [GenBank:CanFam3.1]. The total numbers of fixed and polymorphic synonymous and non-synonymous SNPs and the *N*/*S* ratios are provided. Also listed are non-SNP variants identified during the scan. (XLSX 41 kb)
Additional file 7: Autosomal BUSCO orthologs identified in the CanFam3.1 assembly. Scaffolds are identified by GenBank accession. (XLSX 182 kb)
Additional file 8: Autosomal BUSCO orthologs identified in the Kenyan *Lycaon pictus* genome. Scaffolds are identified by GenBank accession. (XLSX 166 kb)
Additional file 9: Autosomal BUSCO orthologs identified in the South African *Lycaon pictus* genome. Scaffolds are identified by GenBank accession. (XLSX 170 kb)
Additional file 10: Genic effects of *Lycaon* MSY coding sequence variants. (XLSX 55 kb)
Additional file 11: Reconstruction of the *Lycaon* individuals’ autosomal and X-chromosomal demographic history using the pairwise sequentially Markovian coalescent with medium coverage stringency. Initial results are plotted using dark-colored curves, with the bootstrap replicates plotted in lighter hues of the corresponding colors. (EPS 252 kb)
Additional file 12: Reconstruction of the *Lycaon* individuals’ autosomal and X-chromosomal demographic history using the pairwise sequentially Markovian coalescent with high coverage stringency. Initial results are plotted using dark-colored curves, with the bootstrap replicates plotted in lighter hues of the corresponding colors. (EPS 189 kb)

## Abbreviations

bp: Base pairs
CDS: Coding sequence
*dN*/*dS*: Non-synonymous substitutions per non-synonymous site to synonymous substitutions per synonymous site
IACUC: Institutional Animal Care and Use Committee
MSY: Male-specific Y chromosome
N/S: Non-synonymous substitutions to synonymous substitutions
*N*_e_: Effective population size
SNP: Single nucleotide polymorphism
