# Supplementary material for: Genome sequence, population history, and pelage genetics of the endangered African wild dog (Lycaon pictus)
Source: BMC Genomics. 2016 Dec 9;17:1013. doi: 10.1186/s12864-016-3368-9 (PMC5148847; doi:10.1186/s12864-016-3368-9)
Supplement: Additional file 1: — Genic effects of SnpEff-annotated autosomal and X-chromosomal variants for the Kenyan Lycaon individual. (HTML 1596 kb) [file 12864_2016_3368_MOESM1_ESM.html]

### SnpEff: Variant analysis

|  |
| --- |
| **Contents** Summary   Variant rate by chromosome  Variants by type   Number of variants by impact    Number of variants by functional class    Number of variants by effect   Quality histogram  InDel length histogram  Base variant table  Transition vs transversions (ts/tv)   Allele frequency    Allele Count    Codon change table    Amino acid change table    Chromosome variants plots    Details by gene |


---


**Summary**

|  |  |
| --- | --- |
| **Genome** | canfam3\_nuclear |
| **Date** | 2016-04-13 00:46 |
| **SnpEff version** | ``` SnpEff 4.1l (build 2015-10-03), by Pablo Cingolani ``` |
| **Command line arguments** | ``` SnpEff  canfam3_nuclear WDF20_nuclear_concat.q20.vcf ``` |
| **Warnings** | 415,127 |
| **Errors** | 0 |
| **Number of lines (input file)** | 14,619,597 |
| **Number of variants (before filter)** | 14,725,007 |
| **Number of not variants  (i.e. reference equals alternative)** | 0 |
| **Number of variants processed   (i.e. after filter and non-variants)** | 14,725,007 |
| **Number of known variants  (i.e. non-empty ID)** | 0 ( 0% ) |
| **Number of multi-allelic VCF entries  (i.e. more than two alleles)** | 39,860 |
| **Number of effects** | 34,001,288 |
| **Genome total length** | 2,410,960,148 |
| **Genome effective length** | 2,367,221,317 |
| **Variant rate** | 1 variant every 160 bases |


---


 **Variants rate details** 

| Chromosome | Length | Variants | Variants rate |
| --- | --- | --- | --- |
| NC\_006583\_3 | 122,678,785 | 789,347 | 155 |
| NC\_006584\_3 | 85,426,708 | 544,958 | 156 |
| NC\_006585\_3 | 91,889,043 | 582,256 | 157 |
| NC\_006586\_3 | 88,276,631 | 555,516 | 158 |
| NC\_006587\_3 | 88,915,250 | 604,189 | 147 |
| NC\_006588\_3 | 77,573,801 | 501,773 | 154 |
| NC\_006589\_3 | 80,974,532 | 494,505 | 163 |
| NC\_006590\_3 | 74,330,416 | 458,084 | 162 |
| NC\_006591\_3 | 61,074,082 | 459,801 | 132 |
| NC\_006592\_3 | 69,331,447 | 457,295 | 151 |
| NC\_006593\_3 | 74,389,097 | 440,522 | 168 |
| NC\_006594\_3 | 72,498,081 | 426,991 | 169 |
| NC\_006595\_3 | 63,241,923 | 408,966 | 154 |
| NC\_006596\_3 | 60,966,679 | 364,043 | 167 |
| NC\_006597\_3 | 64,190,966 | 397,907 | 161 |
| NC\_006598\_3 | 59,632,846 | 383,354 | 155 |
| NC\_006599\_3 | 64,289,059 | 415,394 | 154 |
| NC\_006600\_3 | 55,844,845 | 372,478 | 149 |
| NC\_006601\_3 | 53,741,614 | 331,982 | 161 |
| NC\_006602\_3 | 58,134,056 | 378,362 | 153 |
| NC\_006603\_3 | 50,858,623 | 322,220 | 157 |
| NC\_006604\_3 | 61,439,934 | 366,894 | 167 |
| NC\_006605\_3 | 52,294,480 | 319,850 | 163 |
| NC\_006606\_3 | 47,698,779 | 323,558 | 147 |
| NC\_006607\_3 | 51,628,933 | 333,320 | 154 |
| NC\_006608\_3 | 38,964,690 | 262,586 | 148 |
| NC\_006609\_3 | 45,876,710 | 277,442 | 165 |
| NC\_006610\_3 | 41,182,112 | 279,734 | 147 |
| NC\_006611\_3 | 41,845,238 | 256,645 | 163 |
| NC\_006612\_3 | 40,214,260 | 246,363 | 163 |
| NC\_006613\_3 | 39,895,921 | 249,123 | 160 |
| NC\_006614\_3 | 38,810,281 | 223,077 | 173 |
| NC\_006615\_3 | 31,377,067 | 195,734 | 160 |
| NC\_006616\_3 | 42,124,431 | 278,263 | 151 |
| NC\_006617\_3 | 26,524,999 | 180,415 | 147 |
| NC\_006618\_3 | 30,810,995 | 187,161 | 164 |
| NC\_006619\_3 | 30,902,991 | 202,945 | 152 |
| NC\_006620\_3 | 23,914,537 | 160,262 | 149 |
| NC\_006621\_3 | 123,869,142 | 635,780 | 194 |
| NW\_003726127\_1 | 2,660,953 | 257 | 10,353 |
| NW\_003726128\_1 | 1,881,673 | 1 | 1,881,673 |
| NW\_003726129\_1 | 1,415,205 | 10 | 141,520 |
| NW\_003726130\_1 | 1,067,467 | 49 | 21,785 |
| NW\_003726132\_1 | 881,102 | 37 | 23,813 |
| NW\_003726133\_1 | 822,601 | 5,593 | 147 |
| NW\_003726135\_1 | 745,551 | 225 | 3,313 |
| NW\_003726136\_1 | 602,611 | 692 | 870 |
| NW\_003726137\_1 | 573,630 | 5,225 | 109 |
| NW\_003726139\_1 | 557,017 | 132 | 4,219 |
| NW\_003726140\_1 | 510,604 | 2 | 255,302 |
| NW\_003726141\_1 | 497,093 | 132 | 3,765 |
| NW\_003726142\_1 | 492,630 | 68 | 7,244 |
| NW\_003726143\_1 | 577,062 | 1,053 | 548 |
| NW\_003726144\_1 | 561,270 | 204 | 2,751 |
| NW\_003726145\_1 | 407,330 | 10 | 40,733 |
| NW\_003726147\_1 | 508,668 | 28 | 18,166 |
| NW\_003726148\_1 | 540,093 | 61 | 8,853 |
| NW\_003726149\_1 | 448,434 | 1,617 | 277 |
| NW\_003726150\_1 | 377,800 | 12 | 31,483 |
| NW\_003726151\_1 | 384,880 | 106 | 3,630 |
| NW\_003726153\_1 | 399,987 | 98 | 4,081 |
| NW\_003726156\_1 | 365,470 | 270 | 1,353 |
| NW\_003726159\_1 | 310,270 | 25 | 12,410 |
| NW\_003726160\_1 | 433,650 | 5 | 86,730 |
| NW\_003726161\_1 | 273,228 | 3 | 91,076 |
| NW\_003726162\_1 | 281,659 | 8 | 35,207 |
| NW\_003726167\_1 | 252,720 | 148 | 1,707 |
| NW\_003726168\_1 | 254,975 | 345 | 739 |
| NW\_003726170\_1 | 223,744 | 3 | 74,581 |
| NW\_003726174\_1 | 386,314 | 1,491 | 259 |
| NW\_003726177\_1 | 216,332 | 40 | 5,408 |
| NW\_003726180\_1 | 200,932 | 136 | 1,477 |
| NW\_003726183\_1 | 192,561 | 302 | 637 |
| NW\_003726184\_1 | 210,134 | 11 | 19,103 |
| NW\_003726185\_1 | 197,844 | 47 | 4,209 |
| NW\_003726186\_1 | 196,813 | 106 | 1,856 |
| NW\_003726188\_1 | 210,247 | 20 | 10,512 |
| NW\_003726189\_1 | 177,735 | 15 | 11,849 |
| NW\_003726191\_1 | 166,866 | 65 | 2,567 |
| NW\_003726193\_1 | 157,598 | 94 | 1,676 |
| NW\_003726194\_1 | 143,980 | 1,604 | 89 |
| NW\_003726195\_1 | 141,074 | 753 | 187 |
| NW\_003726197\_1 | 142,260 | 130 | 1,094 |
| NW\_003726199\_1 | 142,296 | 14 | 10,164 |
| NW\_003726200\_1 | 133,119 | 147 | 905 |
| NW\_003726201\_1 | 129,748 | 181 | 716 |
| NW\_003726203\_1 | 127,429 | 563 | 226 |
| NW\_003726205\_1 | 131,872 | 452 | 291 |
| NW\_003726206\_1 | 133,432 | 232 | 575 |
| NW\_003726210\_1 | 115,952 | 598 | 193 |
| NW\_003726211\_1 | 118,237 | 3 | 39,412 |
| NW\_003726213\_1 | 117,332 | 1 | 117,332 |
| NW\_003726214\_1 | 111,759 | 928 | 120 |
| NW\_003726216\_1 | 112,014 | 9 | 12,446 |
| NW\_003726217\_1 | 106,428 | 2 | 53,214 |
| NW\_003726220\_1 | 103,248 | 4 | 25,812 |
| NW\_003726222\_1 | 118,473 | 538 | 220 |
| NW\_003726224\_1 | 109,585 | 27 | 4,058 |
| NW\_003726225\_1 | 124,049 | 48 | 2,584 |
| NW\_003726227\_1 | 101,741 | 549 | 185 |
| NW\_003726228\_1 | 97,365 | 1 | 97,365 |
| NW\_003726229\_1 | 94,395 | 2 | 47,197 |
| NW\_003726230\_1 | 102,992 | 432 | 238 |
| NW\_003726231\_1 | 95,509 | 182 | 524 |
| NW\_003726236\_1 | 85,476 | 661 | 129 |
| NW\_003726237\_1 | 110,512 | 1 | 110,512 |
| NW\_003726239\_1 | 82,459 | 376 | 219 |
| NW\_003726241\_1 | 82,380 | 61 | 1,350 |
| NW\_003726242\_1 | 102,198 | 7 | 14,599 |
| NW\_003726244\_1 | 87,041 | 3 | 29,013 |
| NW\_003726246\_1 | 230,124 | 249 | 924 |
| NW\_003726247\_1 | 78,357 | 3 | 26,119 |
| NW\_003726248\_1 | 256,406 | 441 | 581 |
| NW\_003726251\_1 | 77,100 | 5 | 15,420 |
| NW\_003726252\_1 | 77,130 | 112 | 688 |
| NW\_003726253\_1 | 79,078 | 103 | 767 |
| NW\_003726254\_1 | 90,550 | 3 | 30,183 |
| NW\_003726257\_1 | 97,802 | 301 | 324 |
| NW\_003726258\_1 | 79,712 | 909 | 87 |
| NW\_003726260\_1 | 73,720 | 25 | 2,948 |
| NW\_003726261\_1 | 97,799 | 1 | 97,799 |
| NW\_003726262\_1 | 73,171 | 58 | 1,261 |
| NW\_003726263\_1 | 104,732 | 6 | 17,455 |
| NW\_003726264\_1 | 78,935 | 880 | 89 |
| NW\_003726266\_1 | 68,721 | 1 | 68,721 |
| NW\_003726268\_1 | 68,272 | 690 | 98 |
| NW\_003726273\_1 | 67,496 | 380 | 177 |
| NW\_003726275\_1 | 67,057 | 49 | 1,368 |
| NW\_003726280\_1 | 65,696 | 71 | 925 |
| NW\_003726283\_1 | 88,153 | 1 | 88,153 |
| NW\_003726285\_1 | 70,351 | 294 | 239 |
| NW\_003726287\_1 | 69,126 | 26 | 2,658 |
| NW\_003726289\_1 | 64,123 | 512 | 125 |
| NW\_003726290\_1 | 61,944 | 10 | 6,194 |
| NW\_003726292\_1 | 61,779 | 399 | 154 |
| NW\_003726293\_1 | 90,840 | 5 | 18,168 |
| NW\_003726294\_1 | 61,194 | 14 | 4,371 |
| NW\_003726295\_1 | 63,245 | 5 | 12,649 |
| NW\_003726297\_1 | 88,336 | 29 | 3,046 |
| NW\_003726300\_1 | 70,666 | 8 | 8,833 |
| NW\_003726301\_1 | 71,007 | 3 | 23,669 |
| NW\_003726303\_1 | 59,817 | 4 | 14,954 |
| NW\_003726304\_1 | 66,981 | 79 | 847 |
| NW\_003726305\_1 | 62,805 | 79 | 795 |
| NW\_003726306\_1 | 68,857 | 5 | 13,771 |
| NW\_003726308\_1 | 84,024 | 93 | 903 |
| NW\_003726311\_1 | 56,534 | 3 | 18,844 |
| NW\_003726312\_1 | 59,023 | 23 | 2,566 |
| NW\_003726315\_1 | 54,307 | 5 | 10,861 |
| NW\_003726318\_1 | 57,907 | 39 | 1,484 |
| NW\_003726321\_1 | 54,660 | 2 | 27,330 |
| NW\_003726327\_1 | 52,288 | 3 | 17,429 |
| NW\_003726329\_1 | 50,869 | 9 | 5,652 |
| NW\_003726330\_1 | 53,506 | 8 | 6,688 |
| NW\_003726334\_1 | 59,752 | 160 | 373 |
| NW\_003726336\_1 | 49,027 | 304 | 161 |
| NW\_003726338\_1 | 57,792 | 2 | 28,896 |
| NW\_003726339\_1 | 74,646 | 1 | 74,646 |
| NW\_003726342\_1 | 75,626 | 7 | 10,803 |
| NW\_003726343\_1 | 48,436 | 107 | 452 |
| NW\_003726344\_1 | 47,485 | 337 | 140 |
| NW\_003726348\_1 | 53,681 | 105 | 511 |
| NW\_003726349\_1 | 73,441 | 33 | 2,225 |
| NW\_003726351\_1 | 70,154 | 9 | 7,794 |
| NW\_003726352\_1 | 67,100 | 3 | 22,366 |
| NW\_003726353\_1 | 48,085 | 80 | 601 |
| NW\_003726356\_1 | 58,859 | 329 | 178 |
| NW\_003726357\_1 | 45,065 | 34 | 1,325 |
| NW\_003726358\_1 | 46,098 | 7 | 6,585 |
| NW\_003726362\_1 | 47,960 | 3 | 15,986 |
| NW\_003726363\_1 | 44,571 | 6 | 7,428 |
| NW\_003726365\_1 | 51,679 | 56 | 922 |
| NW\_003726367\_1 | 50,939 | 3 | 16,979 |
| NW\_003726369\_1 | 44,216 | 6 | 7,369 |
| NW\_003726370\_1 | 43,704 | 243 | 179 |
| NW\_003726378\_1 | 42,461 | 18 | 2,358 |
| NW\_003726381\_1 | 69,503 | 9 | 7,722 |
| NW\_003726386\_1 | 43,681 | 182 | 240 |
| NW\_003726389\_1 | 57,240 | 4 | 14,310 |
| NW\_003726390\_1 | 42,861 | 2 | 21,430 |
| NW\_003726392\_1 | 40,931 | 164 | 249 |
| NW\_003726394\_1 | 41,006 | 6 | 6,834 |
| NW\_003726396\_1 | 46,509 | 10 | 4,650 |
| NW\_003726399\_1 | 40,818 | 25 | 1,632 |
| NW\_003726400\_1 | 45,697 | 1 | 45,697 |
| NW\_003726405\_1 | 39,345 | 387 | 101 |
| NW\_003726407\_1 | 47,441 | 49 | 968 |
| NW\_003726410\_1 | 64,983 | 49 | 1,326 |
| NW\_003726416\_1 | 41,432 | 28 | 1,479 |
| NW\_003726419\_1 | 59,547 | 3 | 19,849 |
| NW\_003726420\_1 | 58,434 | 2 | 29,217 |
| NW\_003726422\_1 | 52,308 | 6 | 8,718 |
| NW\_003726424\_1 | 69,036 | 1 | 69,036 |
| NW\_003726425\_1 | 44,261 | 4 | 11,065 |
| NW\_003726427\_1 | 36,777 | 31 | 1,186 |
| NW\_003726435\_1 | 37,117 | 149 | 249 |
| NW\_003726437\_1 | 41,256 | 161 | 256 |
| NW\_003726438\_1 | 45,145 | 4 | 11,286 |
| NW\_003726439\_1 | 40,256 | 1 | 40,256 |
| NW\_003726442\_1 | 35,523 | 296 | 120 |
| NW\_003726445\_1 | 67,206 | 3 | 22,402 |
| NW\_003726447\_1 | 52,898 | 1 | 52,898 |
| NW\_003726455\_1 | 34,700 | 95 | 365 |
| NW\_003726457\_1 | 37,165 | 13 | 2,858 |
| NW\_003726458\_1 | 59,196 | 1 | 59,196 |
| NW\_003726459\_1 | 34,229 | 7 | 4,889 |
| NW\_003726460\_1 | 35,730 | 22 | 1,624 |
| NW\_003726461\_1 | 41,582 | 71 | 585 |
| NW\_003726462\_1 | 33,876 | 67 | 505 |
| NW\_003726465\_1 | 33,186 | 18 | 1,843 |
| NW\_003726466\_1 | 34,753 | 18 | 1,930 |
| NW\_003726477\_1 | 42,604 | 2 | 21,302 |
| NW\_003726478\_1 | 31,902 | 58 | 550 |
| NW\_003726481\_1 | 31,497 | 313 | 100 |
| NW\_003726482\_1 | 32,248 | 4 | 8,062 |
| NW\_003726485\_1 | 52,768 | 3 | 17,589 |
| NW\_003726487\_1 | 31,015 | 150 | 206 |
| NW\_003726488\_1 | 30,934 | 129 | 239 |
| NW\_003726489\_1 | 45,980 | 6 | 7,663 |
| NW\_003726491\_1 | 39,405 | 2 | 19,702 |
| NW\_003726492\_1 | 39,951 | 1 | 39,951 |
| NW\_003726493\_1 | 31,643 | 3 | 10,547 |
| NW\_003726497\_1 | 30,556 | 2 | 15,278 |
| NW\_003726498\_1 | 30,501 | 51 | 598 |
| NW\_003726501\_1 | 31,768 | 100 | 317 |
| NW\_003726505\_1 | 30,166 | 221 | 136 |
| NW\_003726512\_1 | 38,238 | 4 | 9,559 |
| NW\_003726519\_1 | 29,710 | 30 | 990 |
| NW\_003726520\_1 | 29,345 | 162 | 181 |
| NW\_003726523\_1 | 29,738 | 57 | 521 |
| NW\_003726526\_1 | 30,345 | 250 | 121 |
| NW\_003726528\_1 | 29,319 | 4 | 7,329 |
| NW\_003726529\_1 | 28,914 | 40 | 722 |
| NW\_003726530\_1 | 32,931 | 281 | 117 |
| NW\_003726532\_1 | 28,934 | 91 | 317 |
| NW\_003726534\_1 | 28,793 | 115 | 250 |
| NW\_003726540\_1 | 29,108 | 1 | 29,108 |
| NW\_003726545\_1 | 51,377 | 49 | 1,048 |
| NW\_003726547\_1 | 28,369 | 3 | 9,456 |
| NW\_003726549\_1 | 28,200 | 169 | 166 |
| NW\_003726551\_1 | 38,154 | 2 | 19,077 |
| NW\_003726552\_1 | 38,510 | 9 | 4,278 |
| NW\_003726553\_1 | 37,277 | 1 | 37,277 |
| NW\_003726555\_1 | 27,996 | 66 | 424 |
| NW\_003726556\_1 | 38,339 | 1 | 38,339 |
| NW\_003726562\_1 | 27,833 | 53 | 525 |
| NW\_003726563\_1 | 41,669 | 23 | 1,811 |
| NW\_003726567\_1 | 27,463 | 174 | 157 |
| NW\_003726568\_1 | 27,432 | 10 | 2,743 |
| NW\_003726569\_1 | 28,005 | 23 | 1,217 |
| NW\_003726570\_1 | 27,325 | 10 | 2,732 |
| NW\_003726575\_1 | 32,600 | 2 | 16,300 |
| NW\_003726586\_1 | 26,747 | 3 | 8,915 |
| NW\_003726587\_1 | 27,868 | 1 | 27,868 |
| NW\_003726588\_1 | 29,594 | 10 | 2,959 |
| NW\_003726589\_1 | 28,772 | 221 | 130 |
| NW\_003726592\_1 | 26,523 | 94 | 282 |
| NW\_003726593\_1 | 44,314 | 12 | 3,692 |
| NW\_003726594\_1 | 33,462 | 1 | 33,462 |
| NW\_003726597\_1 | 26,369 | 3 | 8,789 |
| NW\_003726599\_1 | 27,345 | 118 | 231 |
| NW\_003726600\_1 | 36,341 | 45 | 807 |
| NW\_003726602\_1 | 26,155 | 131 | 199 |
| NW\_003726605\_1 | 41,410 | 9 | 4,601 |
| NW\_003726606\_1 | 46,217 | 1 | 46,217 |
| NW\_003726611\_1 | 26,077 | 8 | 3,259 |
| NW\_003726613\_1 | 25,686 | 106 | 242 |
| NW\_003726614\_1 | 45,208 | 4 | 11,302 |
| NW\_003726619\_1 | 25,554 | 3 | 8,518 |
| NW\_003726620\_1 | 37,928 | 3 | 12,642 |
| NW\_003726630\_1 | 25,556 | 6 | 4,259 |
| NW\_003726632\_1 | 24,821 | 57 | 435 |
| NW\_003726636\_1 | 24,666 | 185 | 133 |
| NW\_003726637\_1 | 24,856 | 71 | 350 |
| NW\_003726638\_1 | 33,600 | 8 | 4,200 |
| NW\_003726646\_1 | 24,163 | 30 | 805 |
| NW\_003726647\_1 | 40,390 | 59 | 684 |
| NW\_003726649\_1 | 36,033 | 2 | 18,016 |
| NW\_003726650\_1 | 23,898 | 23 | 1,039 |
| NW\_003726653\_1 | 23,825 | 1 | 23,825 |
| NW\_003726654\_1 | 36,336 | 4 | 9,084 |
| NW\_003726663\_1 | 23,542 | 120 | 196 |
| NW\_003726664\_1 | 26,716 | 87 | 307 |
| NW\_003726665\_1 | 23,440 | 67 | 349 |
| NW\_003726666\_1 | 24,967 | 169 | 147 |
| NW\_003726668\_1 | 23,398 | 10 | 2,339 |
| NW\_003726674\_1 | 35,965 | 2 | 17,982 |
| NW\_003726676\_1 | 37,016 | 8 | 4,627 |
| NW\_003726677\_1 | 36,383 | 54 | 673 |
| NW\_003726681\_1 | 23,924 | 209 | 114 |
| NW\_003726682\_1 | 23,069 | 65 | 354 |
| NW\_003726687\_1 | 37,035 | 5 | 7,407 |
| NW\_003726690\_1 | 32,820 | 4 | 8,205 |
| NW\_003726691\_1 | 22,972 | 86 | 267 |
| NW\_003726697\_1 | 23,313 | 5 | 4,662 |
| NW\_003726702\_1 | 30,594 | 75 | 407 |
| NW\_003726706\_1 | 22,331 | 198 | 112 |
| NW\_003726710\_1 | 22,218 | 23 | 966 |
| NW\_003726713\_1 | 26,196 | 58 | 451 |
| NW\_003726716\_1 | 22,078 | 210 | 105 |
| NW\_003726719\_1 | 22,057 | 9 | 2,450 |
| NW\_003726722\_1 | 21,926 | 70 | 313 |
| NW\_003726729\_1 | 21,667 | 81 | 267 |
| NW\_003726730\_1 | 21,638 | 18 | 1,202 |
| NW\_003726732\_1 | 21,584 | 1 | 21,584 |
| NW\_003726735\_1 | 28,254 | 8 | 3,531 |
| NW\_003726739\_1 | 42,665 | 8 | 5,333 |
| NW\_003726740\_1 | 27,551 | 105 | 262 |
| NW\_003726741\_1 | 21,300 | 107 | 199 |
| NW\_003726742\_1 | 21,212 | 39 | 543 |
| NW\_003726743\_1 | 23,068 | 1 | 23,068 |
| NW\_003726745\_1 | 24,946 | 30 | 831 |
| NW\_003726747\_1 | 21,029 | 49 | 429 |
| NW\_003726748\_1 | 21,019 | 57 | 368 |
| NW\_003726749\_1 | 22,065 | 157 | 140 |
| NW\_003726751\_1 | 20,926 | 201 | 104 |
| NW\_003726755\_1 | 20,819 | 1 | 20,819 |
| NW\_003726758\_1 | 20,706 | 17 | 1,218 |
| NW\_003726759\_1 | 34,690 | 123 | 282 |
| NW\_003726764\_1 | 20,596 | 97 | 212 |
| NW\_003726768\_1 | 20,472 | 20 | 1,023 |
| NW\_003726769\_1 | 20,448 | 89 | 229 |
| NW\_003726783\_1 | 20,017 | 121 | 165 |
| NW\_003726785\_1 | 19,960 | 1 | 19,960 |
| NW\_003726786\_1 | 19,955 | 1 | 19,955 |
| NW\_003726787\_1 | 19,951 | 47 | 424 |
| NW\_003726791\_1 | 26,207 | 24 | 1,091 |
| NW\_003726793\_1 | 21,446 | 210 | 102 |
| NW\_003726799\_1 | 19,813 | 7 | 2,830 |
| NW\_003726806\_1 | 20,693 | 1 | 20,693 |
| NW\_003726807\_1 | 20,650 | 3 | 6,883 |
| NW\_003726810\_1 | 19,874 | 29 | 685 |
| NW\_003726813\_1 | 19,477 | 33 | 590 |
| NW\_003726817\_1 | 33,123 | 5 | 6,624 |
| NW\_003726819\_1 | 53,625 | 6 | 8,937 |
| NW\_003726820\_1 | 19,341 | 165 | 117 |
| NW\_003726823\_1 | 28,455 | 17 | 1,673 |
| NW\_003726826\_1 | 19,270 | 61 | 315 |
| NW\_003726830\_1 | 19,177 | 192 | 99 |
| NW\_003726831\_1 | 21,352 | 2 | 10,676 |
| NW\_003726833\_1 | 19,163 | 199 | 96 |
| NW\_003726835\_1 | 19,766 | 14 | 1,411 |
| NW\_003726839\_1 | 37,094 | 11 | 3,372 |
| NW\_003726842\_1 | 21,618 | 174 | 124 |
| NW\_003726847\_1 | 18,954 | 15 | 1,263 |
| NW\_003726855\_1 | 20,000 | 14 | 1,428 |
| NW\_003726858\_1 | 18,807 | 34 | 553 |
| NW\_003726866\_1 | 18,815 | 1 | 18,815 |
| NW\_003726869\_1 | 18,667 | 67 | 278 |
| NW\_003726872\_1 | 23,772 | 164 | 144 |
| NW\_003726875\_1 | 21,006 | 2 | 10,503 |
| NW\_003726879\_1 | 27,860 | 3 | 9,286 |
| NW\_003726880\_1 | 18,404 | 148 | 124 |
| NW\_003726881\_1 | 18,737 | 46 | 407 |
| NW\_003726887\_1 | 18,280 | 188 | 97 |
| NW\_003726889\_1 | 18,258 | 63 | 289 |
| NW\_003726890\_1 | 20,358 | 1 | 20,358 |
| NW\_003726895\_1 | 18,120 | 9 | 2,013 |
| NW\_003726896\_1 | 28,668 | 6 | 4,778 |
| NW\_003726900\_1 | 24,093 | 4 | 6,023 |
| NW\_003726901\_1 | 17,983 | 1 | 17,983 |
| NW\_003726913\_1 | 17,768 | 3 | 5,922 |
| NW\_003726919\_1 | 17,621 | 173 | 101 |
| NW\_003726920\_1 | 19,944 | 7 | 2,849 |
| NW\_003726922\_1 | 23,498 | 1 | 23,498 |
| NW\_003726924\_1 | 17,500 | 101 | 173 |
| NW\_003726927\_1 | 17,485 | 45 | 388 |
| NW\_003726928\_1 | 17,439 | 3 | 5,813 |
| NW\_003726935\_1 | 17,333 | 45 | 385 |
| NW\_003726937\_1 | 17,237 | 87 | 198 |
| NW\_003726938\_1 | 17,223 | 1 | 17,223 |
| NW\_003726948\_1 | 16,999 | 12 | 1,416 |
| NW\_003726956\_1 | 20,014 | 3 | 6,671 |
| NW\_003726959\_1 | 16,645 | 8 | 2,080 |
| NW\_003726963\_1 | 16,573 | 37 | 447 |
| NW\_003726973\_1 | 16,411 | 21 | 781 |
| NW\_003726974\_1 | 16,406 | 53 | 309 |
| NW\_003726980\_1 | 16,274 | 5 | 3,254 |
| NW\_003726981\_1 | 16,274 | 5 | 3,254 |
| NW\_003726983\_1 | 16,219 | 70 | 231 |
| NW\_003726984\_1 | 19,561 | 9 | 2,173 |
| NW\_003726986\_1 | 16,168 | 116 | 139 |
| NW\_003726989\_1 | 18,404 | 144 | 127 |
| NW\_003726992\_1 | 16,101 | 19 | 847 |
| NW\_003726996\_1 | 16,033 | 1 | 16,033 |
| NW\_003726998\_1 | 16,945 | 38 | 445 |
| NW\_003727000\_1 | 19,044 | 3 | 6,348 |
| NW\_003727002\_1 | 15,997 | 2 | 7,998 |
| NW\_003727006\_1 | 15,896 | 4 | 3,974 |
| NW\_003727012\_1 | 15,960 | 12 | 1,330 |
| NW\_003727013\_1 | 15,826 | 14 | 1,130 |
| NW\_003727015\_1 | 22,770 | 82 | 277 |
| NW\_003727026\_1 | 15,837 | 1 | 15,837 |
| NW\_003727032\_1 | 15,586 | 47 | 331 |
| NW\_003727033\_1 | 15,927 | 5 | 3,185 |
| NW\_003727035\_1 | 15,954 | 1 | 15,954 |
| NW\_003727038\_1 | 15,446 | 19 | 812 |
| NW\_003727044\_1 | 26,016 | 7 | 3,716 |
| NW\_003727047\_1 | 15,314 | 30 | 510 |
| NW\_003727053\_1 | 15,218 | 180 | 84 |
| NW\_003727056\_1 | 16,518 | 2 | 8,259 |
| NW\_003727060\_1 | 15,083 | 106 | 142 |
| NW\_003727061\_1 | 15,081 | 49 | 307 |
| NW\_003727062\_1 | 15,050 | 7 | 2,150 |
| NW\_003727065\_1 | 18,475 | 3 | 6,158 |
| NW\_003727067\_1 | 17,792 | 32 | 556 |
| NW\_003727092\_1 | 15,008 | 4 | 3,752 |
| NW\_003727099\_1 | 14,509 | 19 | 763 |
| NW\_003727104\_1 | 14,413 | 23 | 626 |
| NW\_003727105\_1 | 24,251 | 3 | 8,083 |
| NW\_003727109\_1 | 14,354 | 42 | 341 |
| NW\_003727115\_1 | 16,678 | 5 | 3,335 |
| NW\_003727116\_1 | 14,305 | 71 | 201 |
| NW\_003727117\_1 | 14,300 | 59 | 242 |
| NW\_003727119\_1 | 14,274 | 1 | 14,274 |
| NW\_003727127\_1 | 14,197 | 52 | 273 |
| NW\_003727129\_1 | 14,321 | 172 | 83 |
| NW\_003727132\_1 | 20,078 | 15 | 1,338 |
| NW\_003727133\_1 | 14,123 | 113 | 124 |
| NW\_003727135\_1 | 14,570 | 26 | 560 |
| NW\_003727136\_1 | 14,399 | 15 | 959 |
| NW\_003727138\_1 | 14,077 | 66 | 213 |
| NW\_003727141\_1 | 18,091 | 85 | 212 |
| NW\_003727143\_1 | 19,932 | 2 | 9,966 |
| NW\_003727146\_1 | 17,760 | 117 | 151 |
| NW\_003727149\_1 | 17,765 | 11 | 1,615 |
| NW\_003727157\_1 | 18,815 | 7 | 2,687 |
| NW\_003727159\_1 | 26,714 | 3 | 8,904 |
| NW\_003727161\_1 | 13,787 | 1 | 13,787 |
| NW\_003727164\_1 | 13,729 | 8 | 1,716 |
| NW\_003727165\_1 | 13,717 | 94 | 145 |
| NW\_003727167\_1 | 21,823 | 5 | 4,364 |
| NW\_003727174\_1 | 13,593 | 3 | 4,531 |
| NW\_003727176\_1 | 16,606 | 19 | 874 |
| NW\_003727177\_1 | 13,589 | 41 | 331 |
| NW\_003727179\_1 | 16,840 | 1 | 16,840 |
| NW\_003727180\_1 | 13,585 | 3 | 4,528 |
| NW\_003727188\_1 | 16,064 | 45 | 356 |
| NW\_003727196\_1 | 13,330 | 40 | 333 |
| NW\_003727198\_1 | 13,325 | 2 | 6,662 |
| NW\_003727206\_1 | 23,501 | 6 | 3,916 |
| NW\_003727209\_1 | 13,242 | 5 | 2,648 |
| NW\_003727210\_1 | 13,231 | 1 | 13,231 |
| NW\_003727215\_1 | 13,152 | 120 | 109 |
| NW\_003727216\_1 | 15,666 | 4 | 3,916 |
| NW\_003727217\_1 | 15,908 | 9 | 1,767 |
| NW\_003727218\_1 | 13,146 | 80 | 164 |
| NW\_003727223\_1 | 14,890 | 34 | 437 |
| NW\_003727238\_1 | 12,940 | 137 | 94 |
| NW\_003727240\_1 | 16,589 | 7 | 2,369 |
| NW\_003727242\_1 | 13,514 | 6 | 2,252 |
| NW\_003727244\_1 | 12,896 | 5 | 2,579 |
| NW\_003727246\_1 | 12,820 | 8 | 1,602 |
| NW\_003727249\_1 | 12,809 | 1 | 12,809 |
| NW\_003727253\_1 | 12,732 | 1 | 12,732 |
| NW\_003727255\_1 | 12,718 | 55 | 231 |
| NW\_003727256\_1 | 12,715 | 50 | 254 |
| NW\_003727257\_1 | 12,710 | 12 | 1,059 |
| NW\_003727258\_1 | 14,782 | 1 | 14,782 |
| NW\_003727263\_1 | 15,591 | 26 | 599 |
| NW\_003727264\_1 | 12,644 | 47 | 269 |
| NW\_003727270\_1 | 12,569 | 145 | 86 |
| NW\_003727275\_1 | 12,525 | 2 | 6,262 |
| NW\_003727282\_1 | 12,486 | 2 | 6,243 |
| NW\_003727284\_1 | 24,688 | 10 | 2,468 |
| NW\_003727286\_1 | 12,450 | 10 | 1,245 |
| NW\_003727291\_1 | 13,870 | 4 | 3,467 |
| NW\_003727298\_1 | 13,665 | 4 | 3,416 |
| NW\_003727302\_1 | 12,224 | 7 | 1,746 |
| NW\_003727305\_1 | 13,126 | 16 | 820 |
| NW\_003727306\_1 | 15,591 | 7 | 2,227 |
| NW\_003727323\_1 | 12,099 | 35 | 345 |
| NW\_003727324\_1 | 12,095 | 30 | 403 |
| NW\_003727329\_1 | 12,070 | 27 | 447 |
| NW\_003727331\_1 | 14,862 | 15 | 990 |
| NW\_003727349\_1 | 15,148 | 58 | 261 |
| NW\_003727351\_1 | 11,841 | 55 | 215 |
| NW\_003727353\_1 | 17,963 | 9 | 1,995 |
| NW\_003727360\_1 | 12,857 | 2 | 6,428 |
| NW\_003727364\_1 | 11,732 | 81 | 144 |
| NW\_003727369\_1 | 11,686 | 59 | 198 |
| NW\_003727376\_1 | 11,635 | 4 | 2,908 |
| NW\_003727384\_1 | 11,578 | 71 | 163 |
| NW\_003727388\_1 | 14,150 | 23 | 615 |
| NW\_003727392\_1 | 11,546 | 2 | 5,773 |
| NW\_003727404\_1 | 14,051 | 11 | 1,277 |
| NW\_003727405\_1 | 15,624 | 4 | 3,906 |
| NW\_003727411\_1 | 11,390 | 30 | 379 |
| NW\_003727413\_1 | 13,788 | 18 | 766 |
| NW\_003727417\_1 | 11,342 | 1 | 11,342 |
| NW\_003727423\_1 | 11,270 | 39 | 288 |
| NW\_003727429\_1 | 11,448 | 34 | 336 |
| NW\_003727433\_1 | 12,555 | 53 | 236 |
| NW\_003727450\_1 | 16,619 | 11 | 1,510 |
| NW\_003727464\_1 | 11,030 | 23 | 479 |
| NW\_003727474\_1 | 10,880 | 91 | 119 |
| NW\_003727478\_1 | 10,853 | 2 | 5,426 |
| NW\_003727483\_1 | 10,837 | 29 | 373 |
| NW\_003727489\_1 | 10,792 | 130 | 83 |
| NW\_003727490\_1 | 13,507 | 46 | 293 |
| NW\_003727498\_1 | 10,728 | 1 | 10,728 |
| NW\_003727501\_1 | 10,696 | 9 | 1,188 |
| NW\_003727507\_1 | 11,838 | 16 | 739 |
| NW\_003727508\_1 | 10,664 | 89 | 119 |
| NW\_003727509\_1 | 11,181 | 3 | 3,727 |
| NW\_003727510\_1 | 13,538 | 20 | 676 |
| NW\_003727512\_1 | 10,625 | 16 | 664 |
| NW\_003727522\_1 | 10,537 | 3 | 3,512 |
| NW\_003727525\_1 | 10,534 | 1 | 10,534 |
| NW\_003727533\_1 | 10,500 | 47 | 223 |
| NW\_003727545\_1 | 16,700 | 6 | 2,783 |
| NW\_003727546\_1 | 10,403 | 57 | 182 |
| NW\_003727565\_1 | 10,311 | 2 | 5,155 |
| NW\_003727572\_1 | 10,592 | 29 | 365 |
| NW\_003727577\_1 | 10,263 | 2 | 5,131 |
| NW\_003727579\_1 | 10,589 | 17 | 622 |
| NW\_003727582\_1 | 11,833 | 21 | 563 |
| NW\_003727596\_1 | 10,162 | 1 | 10,162 |
| NW\_003727601\_1 | 13,354 | 20 | 667 |
| NW\_003727611\_1 | 13,874 | 39 | 355 |
| NW\_003727617\_1 | 10,065 | 88 | 114 |
| NW\_003727618\_1 | 10,093 | 19 | 531 |
| NW\_003727621\_1 | 10,484 | 49 | 213 |
| NW\_003727629\_1 | 11,521 | 57 | 202 |
| NW\_003727630\_1 | 11,079 | 1 | 11,079 |
| NW\_003727634\_1 | 15,551 | 39 | 398 |
| NW\_003727639\_1 | 9,913 | 35 | 283 |
| NW\_003727640\_1 | 9,908 | 17 | 582 |
| NW\_003727646\_1 | 9,843 | 17 | 579 |
| NW\_003727647\_1 | 12,747 | 5 | 2,549 |
| NW\_003727649\_1 | 9,812 | 66 | 148 |
| NW\_003727655\_1 | 9,786 | 31 | 315 |
| NW\_003727659\_1 | 9,731 | 4 | 2,432 |
| NW\_003727665\_1 | 9,679 | 91 | 106 |
| NW\_003727667\_1 | 9,657 | 84 | 114 |
| NW\_003727674\_1 | 10,330 | 11 | 939 |
| NW\_003727678\_1 | 9,580 | 2 | 4,790 |
| NW\_003727682\_1 | 10,339 | 2 | 5,169 |
| NW\_003727684\_1 | 9,548 | 84 | 113 |
| NW\_003727690\_1 | 13,392 | 4 | 3,348 |
| NW\_003727698\_1 | 9,481 | 83 | 114 |
| NW\_003727700\_1 | 9,477 | 1 | 9,477 |
| NW\_003727702\_1 | 9,453 | 87 | 108 |
| NW\_003727707\_1 | 15,866 | 1 | 15,866 |
| NW\_003727718\_1 | 11,135 | 3 | 3,711 |
| NW\_003727727\_1 | 9,334 | 5 | 1,866 |
| NW\_003727731\_1 | 12,406 | 2 | 6,203 |
| NW\_003727735\_1 | 9,301 | 25 | 372 |
| NW\_003727763\_1 | 9,159 | 29 | 315 |
| NW\_003727765\_1 | 9,150 | 5 | 1,830 |
| NW\_003727773\_1 | 9,120 | 43 | 212 |
| NW\_003727775\_1 | 9,112 | 26 | 350 |
| NW\_003727793\_1 | 9,031 | 50 | 180 |
| NW\_003727798\_1 | 9,000 | 2 | 4,500 |
| NW\_003727802\_1 | 8,985 | 1 | 8,985 |
| NW\_003727811\_1 | 10,513 | 9 | 1,168 |
| NW\_003727812\_1 | 8,942 | 31 | 288 |
| NW\_003727816\_1 | 11,959 | 12 | 996 |
| NW\_003727818\_1 | 8,930 | 6 | 1,488 |
| NW\_003727820\_1 | 8,988 | 18 | 499 |
| NW\_003727825\_1 | 8,890 | 39 | 227 |
| NW\_003727831\_1 | 11,392 | 72 | 158 |
| NW\_003727868\_1 | 8,677 | 57 | 152 |
| NW\_003727881\_1 | 8,616 | 11 | 783 |
| NW\_003727883\_1 | 8,611 | 51 | 168 |
| NW\_003727885\_1 | 8,606 | 29 | 296 |
| NW\_003727888\_1 | 8,592 | 2 | 4,296 |
| NW\_003727890\_1 | 8,572 | 12 | 714 |
| NW\_003727910\_1 | 8,489 | 15 | 565 |
| NW\_003727913\_1 | 8,468 | 39 | 217 |
| NW\_003727925\_1 | 8,415 | 34 | 247 |
| NW\_003727928\_1 | 8,409 | 61 | 137 |
| NW\_003727941\_1 | 9,862 | 23 | 428 |
| NW\_003727946\_1 | 8,339 | 1 | 8,339 |
| NW\_003727951\_1 | 8,308 | 53 | 156 |
| NW\_003727972\_1 | 8,235 | 58 | 141 |
| NW\_003727975\_1 | 8,218 | 1 | 8,218 |
| NW\_003727979\_1 | 8,655 | 55 | 157 |
| NW\_003727983\_1 | 8,194 | 48 | 170 |
| NW\_003727984\_1 | 8,193 | 35 | 234 |
| NW\_003727996\_1 | 8,154 | 2 | 4,077 |
| NW\_003728001\_1 | 8,141 | 20 | 407 |
| NW\_003728005\_1 | 8,118 | 2 | 4,059 |
| NW\_003728012\_1 | 8,085 | 23 | 351 |
| NW\_003728026\_1 | 8,038 | 30 | 267 |
| NW\_003728039\_1 | 8,006 | 1 | 8,006 |
| NW\_003728044\_1 | 7,975 | 3 | 2,658 |
| NW\_003728045\_1 | 9,411 | 3 | 3,137 |
| NW\_003728047\_1 | 7,972 | 1 | 7,972 |
| NW\_003728053\_1 | 7,951 | 25 | 318 |
| NW\_003728055\_1 | 11,651 | 11 | 1,059 |
| NW\_003728056\_1 | 7,942 | 5 | 1,588 |
| NW\_003728059\_1 | 8,109 | 17 | 477 |
| NW\_003728060\_1 | 8,353 | 1 | 8,353 |
| NW\_003728064\_1 | 7,905 | 62 | 127 |
| NW\_003728076\_1 | 7,858 | 66 | 119 |
| NW\_003728084\_1 | 13,468 | 56 | 240 |
| NW\_003728088\_1 | 7,822 | 39 | 200 |
| NW\_003728089\_1 | 7,821 | 16 | 488 |
| NW\_003728091\_1 | 7,819 | 12 | 651 |
| NW\_003728092\_1 | 7,816 | 18 | 434 |
| NW\_003728097\_1 | 7,802 | 18 | 433 |
| NW\_003728107\_1 | 7,767 | 35 | 221 |
| NW\_003728109\_1 | 7,758 | 13 | 596 |
| NW\_003728139\_1 | 7,680 | 17 | 451 |
| NW\_003728154\_1 | 7,630 | 30 | 254 |
| NW\_003728163\_1 | 7,603 | 87 | 87 |
| NW\_003728169\_1 | 7,584 | 4 | 1,896 |
| NW\_003728176\_1 | 7,556 | 4 | 1,889 |
| NW\_003728179\_1 | 7,533 | 1 | 7,533 |
| NW\_003728180\_1 | 10,986 | 38 | 289 |
| NW\_003728182\_1 | 7,528 | 19 | 396 |
| NW\_003728186\_1 | 10,377 | 26 | 399 |
| NW\_003728199\_1 | 7,466 | 9 | 829 |
| NW\_003728207\_1 | 7,450 | 30 | 248 |
| NW\_003728208\_1 | 7,455 | 2 | 3,727 |
| NW\_003728210\_1 | 7,441 | 2 | 3,720 |
| NW\_003728238\_1 | 7,339 | 49 | 149 |
| NW\_003728239\_1 | 7,339 | 61 | 120 |
| NW\_003728240\_1 | 9,688 | 33 | 293 |
| NW\_003728257\_1 | 7,281 | 40 | 182 |
| NW\_003728259\_1 | 7,279 | 1 | 7,279 |
| NW\_003728261\_1 | 7,272 | 3 | 2,424 |
| NW\_003728277\_1 | 7,227 | 3 | 2,409 |
| NW\_003728283\_1 | 7,214 | 5 | 1,442 |
| NW\_003728285\_1 | 7,210 | 47 | 153 |
| NW\_003728288\_1 | 7,386 | 3 | 2,462 |
| NW\_003728310\_1 | 7,142 | 62 | 115 |
| NW\_003728319\_1 | 7,105 | 1 | 7,105 |
| NW\_003728324\_1 | 7,099 | 3 | 2,366 |
| NW\_003728328\_1 | 13,114 | 12 | 1,092 |
| NW\_003728330\_1 | 7,066 | 42 | 168 |
| NW\_003728334\_1 | 7,058 | 7 | 1,008 |
| NW\_003728347\_1 | 7,017 | 1 | 7,017 |
| NW\_003728357\_1 | 6,989 | 14 | 499 |
| NW\_003728358\_1 | 6,998 | 5 | 1,399 |
| NW\_003728362\_1 | 6,977 | 43 | 162 |
| NW\_003728365\_1 | 9,343 | 3 | 3,114 |
| NW\_003728371\_1 | 6,945 | 3 | 2,315 |
| NW\_003728374\_1 | 6,942 | 36 | 192 |
| NW\_003728382\_1 | 6,928 | 3 | 2,309 |
| NW\_003728396\_1 | 6,893 | 1 | 6,893 |
| NW\_003728406\_1 | 6,862 | 1 | 6,862 |
| NW\_003728408\_1 | 7,248 | 15 | 483 |
| NW\_003728431\_1 | 6,812 | 28 | 243 |
| NW\_003728442\_1 | 6,784 | 6 | 1,130 |
| NW\_003728448\_1 | 6,755 | 27 | 250 |
| NW\_003728453\_1 | 6,732 | 1 | 6,732 |
| NW\_003728457\_1 | 6,731 | 53 | 127 |
| NW\_003728458\_1 | 11,326 | 6 | 1,887 |
| NW\_003728460\_1 | 6,714 | 31 | 216 |
| NW\_003728462\_1 | 6,712 | 15 | 447 |
| NW\_003728463\_1 | 6,698 | 3 | 2,232 |
| NW\_003728470\_1 | 6,686 | 16 | 417 |
| NW\_003728477\_1 | 6,667 | 2 | 3,333 |
| NW\_003728488\_1 | 6,742 | 8 | 842 |
| NW\_003728492\_1 | 6,611 | 44 | 150 |
| NW\_003728514\_1 | 6,684 | 21 | 318 |
| NW\_003728521\_1 | 6,533 | 49 | 133 |
| NW\_003728528\_1 | 6,518 | 37 | 176 |
| NW\_003728539\_1 | 6,481 | 18 | 360 |
| NW\_003728547\_1 | 6,452 | 1 | 6,452 |
| NW\_003728554\_1 | 6,427 | 6 | 1,071 |
| NW\_003728558\_1 | 6,422 | 2 | 3,211 |
| NW\_003728572\_1 | 6,389 | 47 | 135 |
| NW\_003728578\_1 | 6,354 | 1 | 6,354 |
| NW\_003728585\_1 | 6,342 | 14 | 453 |
| NW\_003728595\_1 | 7,225 | 25 | 289 |
| NW\_003728620\_1 | 6,266 | 13 | 482 |
| NW\_003728621\_1 | 6,266 | 44 | 142 |
| NW\_003728632\_1 | 6,246 | 42 | 148 |
| NW\_003728644\_1 | 6,223 | 53 | 117 |
| NW\_003728652\_1 | 6,208 | 12 | 517 |
| NW\_003728653\_1 | 6,207 | 1 | 6,207 |
| NW\_003728657\_1 | 6,192 | 11 | 562 |
| NW\_003728664\_1 | 8,707 | 1 | 8,707 |
| NW\_003728668\_1 | 6,173 | 13 | 474 |
| NW\_003728670\_1 | 9,546 | 22 | 433 |
| NW\_003728680\_1 | 6,145 | 26 | 236 |
| NW\_003728682\_1 | 6,144 | 24 | 256 |
| NW\_003728687\_1 | 6,133 | 29 | 211 |
| NW\_003728689\_1 | 6,131 | 5 | 1,226 |
| NW\_003728703\_1 | 6,084 | 8 | 760 |
| NW\_003728707\_1 | 6,061 | 24 | 252 |
| NW\_003728732\_1 | 6,000 | 8 | 750 |
| NW\_003728743\_1 | 5,964 | 5 | 1,192 |
| NW\_003728755\_1 | 5,926 | 1 | 5,926 |
| NW\_003728762\_1 | 5,907 | 7 | 843 |
| NW\_003728766\_1 | 5,888 | 2 | 2,944 |
| NW\_003728770\_1 | 5,880 | 6 | 980 |
| NW\_003728781\_1 | 5,852 | 25 | 234 |
| NW\_003728783\_1 | 5,842 | 10 | 584 |
| NW\_003728787\_1 | 5,818 | 64 | 90 |
| NW\_003728799\_1 | 5,804 | 35 | 165 |
| NW\_003728802\_1 | 5,793 | 36 | 160 |
| NW\_003728808\_1 | 5,773 | 3 | 1,924 |
| NW\_003728809\_1 | 5,768 | 53 | 108 |
| NW\_003728814\_1 | 5,747 | 3 | 1,915 |
| NW\_003728818\_1 | 5,723 | 64 | 89 |
| NW\_003728821\_1 | 5,712 | 21 | 272 |
| NW\_003728828\_1 | 5,695 | 1 | 5,695 |
| NW\_003728829\_1 | 5,694 | 52 | 109 |
| NW\_003728837\_1 | 5,678 | 18 | 315 |
| NW\_003728838\_1 | 5,677 | 1 | 5,677 |
| NW\_003728858\_1 | 5,629 | 13 | 433 |
| NW\_003728868\_1 | 5,605 | 56 | 100 |
| NW\_003728870\_1 | 5,588 | 14 | 399 |
| NW\_003728885\_1 | 5,549 | 2 | 2,774 |
| NW\_003728891\_1 | 5,531 | 32 | 172 |
| NW\_003728899\_1 | 5,496 | 38 | 144 |
| NW\_003728912\_1 | 8,038 | 19 | 423 |
| NW\_003728923\_1 | 5,416 | 48 | 112 |
| NW\_003728943\_1 | 5,366 | 35 | 153 |
| NW\_003728951\_1 | 5,332 | 4 | 1,333 |
| NW\_003728952\_1 | 5,330 | 24 | 222 |
| NW\_003728964\_1 | 5,295 | 10 | 529 |
| NW\_003728970\_1 | 5,290 | 24 | 220 |
| NW\_003728973\_1 | 5,288 | 8 | 661 |
| NW\_003728986\_1 | 5,246 | 60 | 87 |
| NW\_003728988\_1 | 5,243 | 16 | 327 |
| NW\_003728990\_1 | 5,240 | 1 | 5,240 |
| NW\_003728991\_1 | 5,235 | 1 | 5,235 |
| NW\_003729001\_1 | 5,185 | 2 | 2,592 |
| NW\_003729004\_1 | 5,174 | 31 | 166 |
| NW\_003729009\_1 | 5,154 | 3 | 1,718 |
| NW\_003729013\_1 | 5,147 | 28 | 183 |
| NW\_003729014\_1 | 5,137 | 3 | 1,712 |
| NW\_003729019\_1 | 5,124 | 3 | 1,708 |
| NW\_003729020\_1 | 5,123 | 32 | 160 |
| NW\_003729025\_1 | 5,115 | 16 | 319 |
| NW\_003729038\_1 | 5,054 | 62 | 81 |
| NW\_003729040\_1 | 5,051 | 17 | 297 |
| NW\_003729050\_1 | 5,015 | 1 | 5,015 |
| NW\_003729051\_1 | 5,010 | 23 | 217 |
| NW\_003729060\_1 | 4,989 | 1 | 4,989 |
| NW\_003729061\_1 | 4,988 | 1 | 4,988 |
| NW\_003729070\_1 | 4,967 | 27 | 183 |
| NW\_003729071\_1 | 4,965 | 8 | 620 |
| NW\_003729087\_1 | 4,922 | 2 | 2,461 |
| NW\_003729088\_1 | 4,921 | 3 | 1,640 |
| NW\_003729093\_1 | 4,893 | 33 | 148 |
| NW\_003729105\_1 | 4,863 | 31 | 156 |
| NW\_003729115\_1 | 4,818 | 35 | 137 |
| NW\_003729117\_1 | 4,798 | 31 | 154 |
| NW\_003729148\_1 | 4,694 | 2 | 2,347 |
| NW\_003729155\_1 | 4,676 | 26 | 179 |
| NW\_003729158\_1 | 4,669 | 7 | 667 |
| NW\_003729172\_1 | 4,601 | 5 | 920 |
| NW\_003729173\_1 | 4,600 | 26 | 176 |
| NW\_003729178\_1 | 4,577 | 5 | 915 |
| NW\_003729189\_1 | 4,550 | 12 | 379 |
| NW\_003729191\_1 | 4,541 | 3 | 1,513 |
| NW\_003729192\_1 | 4,539 | 12 | 378 |
| NW\_003729203\_1 | 4,454 | 7 | 636 |
| NW\_003729207\_1 | 4,439 | 50 | 88 |
| Total | 2,367,221,317 | 14,725,007 | 160 |


---


 **Number variantss by type**

| **Type** | **Total** |
| --- | --- |
| **SNP** | 12,659,463 |
| **MNP** | 0 |
| **INS** | 973,872 |
| **DEL** | 1,091,672 |
| **MIXED** | 0 |
| **INTERVAL** | 0 |
| **Total** | 14,725,007 |
| --- | --- |


---


 **Number of effects by impact** 

| **Type (alphabetical order)** |  | Count | Percent |
| --- | --- | --- | --- |
| **HIGH** |  | 6,332 | 0.019% |
| **LOW** |  | 237,185 | 0.698% |
| **MODERATE** |  | 108,736 | 0.32% |
| **MODIFIER** |  | 33,649,035 | 98.964% |


---


 **Number of effects by functional class** 

| **Type (alphabetical order)** |  | Count | Percent |
| --- | --- | --- | --- |
| **MISSENSE** |  | 104,471 | 35.541% |
| **NONSENSE** |  | 846 | 0.288% |
| **SILENT** |  | 188,627 | 64.171% |

  

Missense / Silent ratio: 0.5538


---


 **Number of effects by type and region** 

| Type | Region |
| --- | --- |
| | **Type (alphabetical order)** |  | Count | Percent | | --- | --- | --- | --- | | **3\_prime\_UTR\_variant** |  | 293,670 | 0.864% | | **5\_prime\_UTR\_premature\_start\_codon\_gain\_variant** |  | 11,576 | 0.034% | | **5\_prime\_UTR\_truncation+exon\_loss\_variant** |  | 8 | 0% | | **5\_prime\_UTR\_variant** |  | 85,616 | 0.252% | | **chromosome\_number\_variation** |  | 1 | 0% | | **disruptive\_inframe\_deletion** |  | 909 | 0.003% | | **disruptive\_inframe\_deletion+splice\_region\_variant** |  | 5 | 0% | | **disruptive\_inframe\_insertion** |  | 791 | 0.002% | | **disruptive\_inframe\_insertion+splice\_region\_variant** |  | 4 | 0% | | **downstream\_gene\_variant** |  | 2,019,856 | 5.941% | | **exon\_loss\_variant+splice\_donor\_variant+splice\_region\_variant+intron\_variant** |  | 1 | 0% | | **frameshift\_variant** |  | 2,658 | 0.008% | | **frameshift\_variant+splice\_acceptor\_variant+splice\_region\_variant+intron\_variant** |  | 29 | 0% | | **frameshift\_variant+splice\_donor\_variant+splice\_region\_variant+intron\_variant** |  | 42 | 0% | | **frameshift\_variant+splice\_region\_variant** |  | 107 | 0% | | **frameshift\_variant+start\_lost** |  | 24 | 0% | | **frameshift\_variant+start\_lost+splice\_region\_variant** |  | 5 | 0% | | **frameshift\_variant+stop\_gained** |  | 26 | 0% | | **frameshift\_variant+stop\_lost** |  | 47 | 0% | | **frameshift\_variant+stop\_lost+splice\_acceptor\_variant+splice\_region\_variant+intron\_variant** |  | 2 | 0% | | **frameshift\_variant+stop\_lost+splice\_region\_variant** |  | 2 | 0% | | **inframe\_deletion** |  | 1,685 | 0.005% | | **inframe\_deletion+splice\_region\_variant** |  | 3 | 0% | | **inframe\_insertion** |  | 1,434 | 0.004% | | **inframe\_insertion+splice\_region\_variant** |  | 8 | 0% | | **initiator\_codon\_variant** |  | 39 | 0% | | **intergenic\_region** |  | 7,847,956 | 23.081% | | **intragenic\_variant** |  | 2,618 | 0.008% | | **intron\_variant** |  | 21,286,013 | 62.604% | | **missense\_variant** |  | 101,871 | 0.3% | | **missense\_variant+splice\_region\_variant** |  | 2,018 | 0.006% | | **non\_coding\_exon\_variant** |  | 115,618 | 0.34% | | **non\_coding\_transcript\_variant** |  | 352 | 0.001% | | **splice\_acceptor\_variant+3\_prime\_UTR\_variant+intron\_variant** |  | 2 | 0% | | **splice\_acceptor\_variant+5\_prime\_UTR\_variant+intron\_variant** |  | 2 | 0% | | **splice\_acceptor\_variant+disruptive\_inframe\_deletion+splice\_region\_variant+intron\_variant** |  | 5 | 0% | | **splice\_acceptor\_variant+inframe\_deletion+splice\_region\_variant+intron\_variant** |  | 1 | 0% | | **splice\_acceptor\_variant+intron\_variant** |  | 810 | 0.002% | | **splice\_acceptor\_variant+splice\_donor\_variant+splice\_region\_variant+intron\_variant** |  | 3 | 0% | | **splice\_acceptor\_variant+splice\_donor\_variant+splice\_region\_variant+intron\_variant+non\_coding\_exon\_variant** |  | 1 | 0% | | **splice\_acceptor\_variant+splice\_region\_variant+5\_prime\_UTR\_variant+intron\_variant** |  | 4 | 0% | | **splice\_acceptor\_variant+splice\_region\_variant+intron\_variant** |  | 39 | 0% | | **splice\_acceptor\_variant+splice\_region\_variant+intron\_variant+non\_coding\_exon\_variant** |  | 21 | 0% | | **splice\_donor\_variant+3\_prime\_UTR\_variant+intron\_variant** |  | 2 | 0% | | **splice\_donor\_variant+5\_prime\_UTR\_variant+intron\_variant** |  | 4 | 0% | | **splice\_donor\_variant+disruptive\_inframe\_deletion+splice\_region\_variant+intron\_variant** |  | 4 | 0% | | **splice\_donor\_variant+inframe\_deletion+splice\_region\_variant+intron\_variant** |  | 5 | 0% | | **splice\_donor\_variant+intron\_variant** |  | 932 | 0.003% | | **splice\_donor\_variant+splice\_region\_variant+3\_prime\_UTR\_variant+intron\_variant** |  | 2 | 0% | | **splice\_donor\_variant+splice\_region\_variant+5\_prime\_UTR\_variant+intron\_variant** |  | 8 | 0% | | **splice\_donor\_variant+splice\_region\_variant+intron\_variant** |  | 73 | 0% | | **splice\_donor\_variant+splice\_region\_variant+intron\_variant+non\_coding\_exon\_variant** |  | 24 | 0% | | **splice\_region\_variant** |  | 1,670 | 0.005% | | **splice\_region\_variant+downstream\_gene\_variant** |  | 6 | 0% | | **splice\_region\_variant+intron\_variant** |  | 33,855 | 0.1% | | **splice\_region\_variant+non\_coding\_exon\_variant** |  | 1,413 | 0.004% | | **splice\_region\_variant+stop\_retained\_variant** |  | 18 | 0% | | **splice\_region\_variant+synonymous\_variant** |  | 3,977 | 0.012% | | **start\_lost** |  | 216 | 0.001% | | **start\_lost+disruptive\_inframe\_deletion** |  | 1 | 0% | | **start\_lost+inframe\_deletion** |  | 9 | 0% | | **start\_lost+splice\_region\_variant** |  | 9 | 0% | | **stop\_gained** |  | 821 | 0.002% | | **stop\_gained+disruptive\_inframe\_insertion** |  | 2 | 0% | | **stop\_gained+inframe\_insertion** |  | 16 | 0% | | **stop\_gained+inframe\_insertion+splice\_region\_variant** |  | 7 | 0% | | **stop\_gained+splice\_region\_variant** |  | 25 | 0% | | **stop\_lost** |  | 300 | 0.001% | | **stop\_lost+disruptive\_inframe\_deletion** |  | 2 | 0% | | **stop\_lost+inframe\_deletion** |  | 17 | 0% | | **stop\_lost+inframe\_deletion+splice\_region\_variant** |  | 1 | 0% | | **stop\_lost+inframe\_insertion** |  | 1 | 0% | | **stop\_lost+splice\_region\_variant** |  | 19 | 0% | | **stop\_retained\_variant** |  | 122 | 0% | | **synonymous\_variant** |  | 184,509 | 0.543% | | **upstream\_gene\_variant** |  | 1,997,336 | 5.874% | | | **Type (alphabetical order)** |  | Count | Percent | | --- | --- | --- | --- | | **DOWNSTREAM** |  | 2,019,856 | 5.941% | | **EXON** |  | 413,131 | 1.215% | | **INTERGENIC** |  | 7,847,956 | 23.081% | | **INTRON** |  | 21,286,013 | 62.604% | | **NONE** |  | 2,971 | 0.009% | | **SPLICE\_SITE\_ACCEPTOR** |  | 888 | 0.003% | | **SPLICE\_SITE\_DONOR** |  | 1,054 | 0.003% | | **SPLICE\_SITE\_REGION** |  | 40,939 | 0.12% | | **TRANSCRIPT** |  | 274 | 0.001% | | **UPSTREAM** |  | 1,997,336 | 5.874% | | **UTR\_3\_PRIME** |  | 293,670 | 0.864% | | **UTR\_5\_PRIME** |  | 97,200 | 0.286% | |


---


 **Quality:**

```
|  |  |
| --- | --- |
| Min | 20 |
| Max | 228 |
| Mean | 123.738 |
| Median | 117 |
| Standard deviation | 64.439 |
| Values | 20,21,22,23,24,25,26,27,28,29,30,31,32,33,34,35,36,37,38,39,40,41,42,43,44,45,46,47,48,49,50,51,52,53,54,55,56,57,58,59,60,61,62,63,64,65,66,67,68,69,70,71,72,73,74,75,76,77,78,79,80,81,82,83,84,85,86,87,88,89,90,91,92,93,94,95,96,97,98,99,100,101,102,103,104,105,106,107,108,109,110,111,112,113,114,115,116,117,118,119,120,121,122,123,124,125,126,127,128,129,130,131,132,133,134,135,136,137,138,139,140,141,142,143,144,145,146,147,148,149,150,151,152,153,154,155,156,157,158,159,160,161,162,163,164,165,166,167,168,169,170,171,172,173,174,175,176,177,178,179,180,181,182,183,184,185,186,187,188,189,190,191,192,193,194,195,196,197,198,199,200,201,202,203,204,205,206,207,208,209,210,211,212,213,214,215,216,217,218,219,220,221,222,223,224,225,226,227,228 |
| Count | 58320,54463,136412,59971,51114,57452,55950,52967,57420,62312,67267,67456,73711,63264,69812,67833,83033,76264,89846,72112,70291,110456,94177,159565,85941,135353,61324,62773,94348,61742,122140,66700,118559,61889,55704,54031,57948,63036,64852,60417,67335,65267,60623,75089,72912,77960,71010,69974,69833,85342,100204,68751,91584,64837,67040,62104,86555,69211,77933,71757,75196,103252,88280,147510,66372,101238,57446,58934,54441,59569,57918,60076,70708,59817,71722,60316,59312,61012,62641,62469,61722,65307,65568,73600,71638,82218,69903,88329,71461,104779,74540,102413,89038,66223,79249,86278,52817,83601,51979,65006,52454,54343,55357,58539,58744,58746,65110,57653,65363,61169,66685,69145,71355,66598,73341,64522,69789,61757,75788,60866,70519,71111,75458,76854,75911,58010,62164,54319,57307,58194,50478,54785,53862,55043,53021,52658,59268,56996,55633,60782,59627,63644,67877,58771,68072,57353,71120,55080,58113,51296,55584,51800,50763,46948,51399,49479,48097,50603,49373,52802,48955,53097,53719,49696,52817,50944,51955,49617,50500,51620,53134,48834,50909,47684,46921,42591,42992,42108,41705,41096,41938,41020,42500,41848,42606,43949,45366,41608,46986,38729,44930,40892,36624,40869,37259,37731,35322,35911,34743,35113,36541,35873,35186,33998,37288,33352,35486,32516,1507577 |
```


---


 **Insertions and deletions length:**

```
|  |  |
| --- | --- |
| Min | 0 |
| Max | 99 |
| Mean | 1.961 |
| Median | 1 |
| Standard deviation | 3.751 |
| Values | 0,1,2,3,4,5,6,7,8,9,10,11,12,13,14,15,16,17,18,19,20,21,22,23,24,25,26,27,28,29,30,31,32,33,34,35,36,37,38,39,40,41,42,43,44,45,46,47,48,49,50,51,52,53,54,55,56,57,58,59,60,61,62,63,64,65,66,67,68,69,70,71,72,73,74,75,76,77,78,79,80,82,83,84,85,86,87,88,90,91,95,99 |
| Count | 357355,1260631,95419,123880,38917,38312,18084,26436,12338,15773,8038,13525,5998,7469,4627,5956,3073,4027,2356,3191,2048,2064,1548,1972,1289,1249,1057,1107,769,792,609,607,480,426,408,439,287,268,251,270,227,176,141,150,153,131,109,109,87,93,74,91,54,53,53,55,42,48,34,39,35,27,27,20,24,9,17,8,9,19,9,8,5,8,2,4,10,3,7,4,2,4,2,4,1,1,3,2,1,2,2,1 |
```


---


 **Base changes (SNPs)** 

|  |  |  |  |  |
| --- | --- | --- | --- | --- |
|  | **A** | **C** | **G** | **T** |
| **A** | 0 | 477,126 | 2,120,256 | 464,739 |
| **C** | 495,843 | 0 | 453,826 | 2,320,189 |
| **G** | 2,318,614 | 453,407 | 0 | 495,784 |
| **T** | 463,773 | 2,119,024 | 476,882 | 0 |

---


  **Ts/Tv (transitions / transversions)** 

**Note:** Only SNPs are used for this statistic.  
**Note:** This Ts/Tv ratio is a 'raw' ratio (ratio of observed events).

|  |  |
| --- | --- |
| Transitions | 17,458,968 |
| Transversions | 7,436,273 |
| Ts/Tv ratio | 2.3478 |

**All variants:**

```
Sample ,WDF20_nuclear_concat_auto.rmdup.bam,Total
Transitions ,17458968,17458968
Transversions ,7436273,7436273
Ts/Tv ,2.348,2.348
```

**Only known variants** (i.e. the ones having a non-empty ID field):

```
No results available (empty input?)
```

---


  **Allele frequency** 
  

|  |  |
| --- | --- |
| Min | 50 |
| Max | 100 |
| Mean | 98.158 |
| Median | 100 |
| Standard deviation | 9.419 |
| Values | 50,100 |
| Count | 538668,14080929 |

---


  **Allele Count** 
  

|  |  |
| --- | --- |
| Min | 1 |
| Max | 2 |
| Mean | 1.963 |
| Median | 2 |
| Standard deviation | 0.188 |
| Values | 1,2 |
| Count | 538668,14080929 |

---


  **Hom/Het per sample** 
  
  
  

```
Sample_names , WDF20_nuclear_concat_auto.rmdup.bam
Reference , 0
Het , 538668
Hom , 14080929
Missing , 0
```

---


 **Codon changes**

How to read this table:   
- Rows are reference codons and columns are changed codons. E.g. Row 'AAA' column 'TAA' indicates how many 'AAA' codons have been replaced by 'TAA' codons.  
- Red background colors indicate that more changes happened (heat-map).  
- Diagonals are indicated using grey background color   
- WARNING: This table may include different translation codon tables (e.g. mamalian DNA and mitochondrial DNA).

|  | - | AAA | AAC | AAG | AAT | ACA | ACC | ACG | ACT | AGA | AGC | AGG | AGT | ATA | ATC | ATG | ATT | CAA | CAC | CAG | CAT | CCA | CCC | CCG | CCT | CGA | CGC | CGG | CGT | CTA | CTC | CTG | CTT | GAA | GAC | GAG | GAT | GCA | GCC | GCG | GCT | GGA | GGC | GGG | GGT | GTA | GTC | GTG | GTT | NGC | NNN | TAA | TAC | TAG | TAT | TCA | TCC | TCG | TCT | TGA | TGC | TGG | TGT | TTA | TTC | TTG | TTT |
| --- | --- | --- | --- | --- | --- | --- | --- | --- | --- | --- | --- | --- | --- | --- | --- | --- | --- | --- | --- | --- | --- | --- | --- | --- | --- | --- | --- | --- | --- | --- | --- | --- | --- | --- | --- | --- | --- | --- | --- | --- | --- | --- | --- | --- | --- | --- | --- | --- | --- | --- | --- | --- | --- | --- | --- | --- | --- | --- | --- | --- | --- | --- | --- | --- | --- | --- | --- |
| - |  | 68 | 100 | 69 | 40 | 31 | 99 | 31 | 62 | 31 | 163 | 73 | 55 | 21 | 30 | 21 | 25 | 45 | 130 | 307 | 52 | 74 | 418 | 324 | 127 | 28 | 124 | 142 | 33 | 11 | 96 | 201 | 37 | 181 | 178 | 366 | 61 | 96 | 399 | 366 | 108 | 208 | 447 | 289 | 96 | 21 | 82 | 102 | 44 |  |  | 10 | 17 | 14 | 13 | 42 | 125 | 32 | 94 | 11 | 50 | 36 | 33 | 27 | 45 | 23 | 35 |
| AAA | 106 | 35 | 188 | 2,249 | 122 | 78 |  |  |  | 506 |  |  |  | 38 |  |  |  | 149 |  |  |  |  |  |  |  |  |  |  |  |  |  |  |  | 504 |  |  |  |  |  |  |  |  |  |  |  |  |  |  |  |  |  | 12 |  |  |  |  |  |  |  |  |  |  |  |  |  |  |  |
| AAC | 67 | 130 | 22 | 131 | 3,671 |  | 112 |  |  |  | 854 | 2 |  |  | 36 |  |  |  | 103 |  |  |  |  |  |  |  |  |  |  |  |  |  |  |  | 440 |  |  |  |  |  |  |  | 5 |  |  |  |  | 1 |  |  |  |  | 50 |  |  |  |  |  |  |  |  |  |  |  |  |  |  |
| AAG | 176 | 2,839 | 119 | 67 | 89 | 6 | 1 | 134 |  | 1 |  | 777 |  |  | 1 | 70 |  |  |  | 237 |  |  | 1 |  |  |  |  |  |  |  |  |  |  |  |  | 633 |  |  |  |  |  |  |  | 1 |  |  |  |  |  |  |  | 2 |  | 15 |  |  |  |  |  |  |  |  |  |  |  |  |  |
| AAT | 72 | 78 | 3,011 | 74 | 21 |  |  |  | 106 | 1 |  |  | 755 |  | 4 |  | 52 |  |  |  | 89 |  |  |  |  |  |  |  |  |  |  |  |  | 2 |  | 5 | 397 |  |  |  |  |  |  |  |  |  |  |  |  |  |  | 1 |  |  | 62 |  | 1 |  |  |  |  |  |  |  |  |  |  |
| ACA | 83 | 123 | 2 |  |  | 32 | 526 | 3,462 | 312 | 116 | 2 |  | 1 | 548 |  |  |  |  |  |  |  | 117 |  |  |  |  |  |  |  |  |  |  |  | 1 | 3 |  | 1 | 804 |  |  |  |  |  |  |  |  |  |  |  |  |  |  | 1 |  |  | 71 |  |  | 1 |  |  |  |  |  |  |  |  |
| ACC | 129 | 3 | 187 |  |  | 716 | 56 | 735 | 3,133 | 1 | 245 | 1 |  |  | 403 | 1 |  |  | 1 |  |  |  | 147 |  | 1 |  |  |  |  |  |  |  |  |  | 3 |  |  |  | 1,306 |  |  |  |  |  |  |  |  |  |  |  |  |  |  |  |  |  | 111 |  |  |  |  |  |  |  | 1 |  |  |
| ACG | 69 |  | 1 | 65 |  | 4,600 | 642 | 22 | 478 |  |  | 100 |  |  |  | 1,380 |  |  |  |  |  |  |  | 47 |  |  |  |  |  |  |  |  |  |  | 1 |  |  |  |  | 356 |  |  |  |  |  |  |  |  |  |  |  |  |  |  |  |  |  | 54 |  |  |  |  |  |  |  |  |  |
| ACT | 86 |  | 2 |  | 128 | 319 | 2,562 | 463 | 31 | 1 | 1 |  | 223 |  |  |  | 404 |  |  |  |  |  |  |  | 106 |  |  |  |  |  |  |  |  |  |  | 1 |  |  |  |  | 759 |  |  |  |  |  |  |  |  |  |  |  |  |  |  |  |  |  | 124 |  |  | 1 |  |  |  |  |  |
| AGA | 122 | 444 |  |  |  | 78 | 2 |  |  | 20 | 120 | 1,035 | 29 | 68 |  | 1 |  |  |  | 7 |  |  |  |  |  | 307 |  |  |  |  |  |  |  | 1 |  | 3 |  |  |  |  |  | 412 |  |  |  |  |  |  |  |  |  |  |  |  |  |  |  |  |  | 18 |  |  |  |  |  |  |  |
| AGC | 313 | 5 | 894 | 3 | 6 | 3 | 197 | 8 |  | 72 | 68 | 137 | 3,186 |  | 79 |  |  |  |  | 4 |  | 9 |  |  |  |  | 79 |  |  |  | 2 |  |  | 1 |  | 10 |  |  | 1 |  |  |  | 822 |  |  |  |  |  |  |  |  |  |  |  |  |  |  | 1 |  |  | 87 |  |  |  |  |  |  |
| AGG | 91 | 1 | 1 | 851 |  | 1 |  | 36 |  | 1,201 | 143 | 27 | 76 |  |  | 61 |  |  |  | 2 |  | 1 |  |  |  |  |  | 534 |  |  |  |  |  |  |  | 3 |  |  |  | 2 |  |  | 2 | 543 |  |  |  | 2 |  |  |  |  |  |  |  |  |  |  |  |  |  | 50 |  |  |  |  |  |
| AGT | 82 | 1 |  | 2 | 799 | 1 |  |  | 221 | 69 | 2,128 | 140 | 38 |  | 2 | 1 | 65 |  |  | 1 |  |  |  |  |  |  |  |  | 41 |  |  |  |  |  |  |  |  |  |  |  |  |  |  |  | 478 |  |  |  |  |  |  |  |  |  |  |  |  |  |  |  |  |  | 59 |  |  |  |  |
| ATA | 26 | 44 |  |  | 2 | 341 |  | 1 |  | 57 |  |  |  | 23 | 357 | 405 | 226 |  |  |  |  |  |  |  |  |  |  |  |  | 71 |  |  |  |  |  |  |  | 5 |  |  |  |  |  |  |  | 661 |  |  |  |  |  |  |  |  |  |  | 2 |  |  |  |  |  |  | 42 |  |  |  |
| ATC | 38 |  | 66 |  | 2 |  | 354 |  |  | 1 | 112 | 1 |  | 420 | 10 | 107 | 2,836 |  |  |  | 1 |  |  | 1 |  |  |  |  |  |  | 130 |  |  |  |  | 1 | 3 |  |  |  |  |  |  |  |  |  | 982 |  |  |  |  |  |  |  |  |  |  |  |  |  |  |  |  |  | 53 |  |  |
| ATG | 104 | 4 |  | 114 | 9 |  |  | 1,205 | 1 | 1 | 9 | 82 | 1 | 586 | 81 | 32 | 168 |  |  |  | 1 |  |  | 1 |  |  |  |  |  |  |  | 321 |  |  |  |  | 6 |  |  |  |  |  |  |  |  | 1 |  | 1,079 |  |  |  |  |  |  | 2 |  |  |  |  |  |  |  |  |  |  | 206 | 2 |
| ATT | 53 |  |  |  | 42 |  |  | 1 | 310 |  | 1 |  | 26 | 287 | 2,235 | 83 | 10 |  |  |  | 1 |  |  |  |  |  |  |  |  |  |  |  | 75 | 1 |  |  |  |  |  |  |  |  |  |  |  |  |  |  | 765 |  |  |  |  |  |  | 1 |  |  |  |  |  |  |  |  |  |  | 119 |
| CAA | 90 | 147 |  |  |  |  |  |  |  | 1 | 3 |  |  |  |  |  |  | 21 | 113 | 2,475 | 63 | 122 |  |  |  | 543 |  |  |  | 44 |  | 1 |  | 174 |  |  |  |  |  |  |  |  |  |  |  |  |  |  |  |  |  | 60 |  |  |  |  |  |  |  |  |  |  |  |  |  |  |  |
| CAC | 130 |  | 133 |  |  |  |  |  |  |  |  |  |  |  |  |  |  | 151 | 35 | 238 | 2,909 | 5 | 105 |  | 1 |  | 845 | 2 |  |  | 75 | 4 | 1 |  | 89 |  |  | 1 | 1 |  |  |  |  |  |  |  | 8 |  |  |  |  |  | 341 |  |  |  |  |  |  |  |  |  |  |  |  |  |  |
| CAG | 691 |  |  | 282 |  |  |  |  |  |  |  |  |  |  |  |  |  | 2,677 | 209 | 105 | 225 | 7 | 7 | 219 |  |  | 4 | 1,367 |  | 3 |  | 120 |  |  |  | 351 |  | 1 |  |  |  |  |  | 1 |  |  |  |  |  |  |  |  |  | 157 |  | 2 |  |  |  |  |  |  |  |  |  |  |  |
| CAT | 67 |  |  |  | 105 |  |  |  |  |  |  |  |  |  |  |  |  | 66 | 2,567 | 227 | 8 | 2 | 5 |  | 125 |  |  |  | 573 |  | 1 | 1 | 52 |  |  |  | 94 | 1 |  |  |  |  |  |  |  |  |  |  |  |  |  |  |  |  | 215 |  |  |  |  |  |  |  |  |  |  |  |  |
| CCA | 277 |  |  |  |  | 168 | 1 | 1 |  |  |  |  |  |  |  |  |  | 121 | 2 | 2 |  | 23 | 574 | 4,456 | 351 | 103 |  |  | 1 | 363 | 1 | 6 |  |  |  |  |  | 106 | 2 |  |  |  |  | 1 |  |  |  |  |  |  |  |  |  |  |  | 489 |  |  |  |  |  |  |  |  |  |  |  |
| CCC | 496 |  |  |  |  |  | 255 |  |  |  |  |  |  |  |  |  |  |  | 174 | 9 |  | 502 | 106 | 1,035 | 4,147 |  | 167 | 1 |  |  | 438 | 2 |  | 1 | 1 |  |  |  | 265 | 2 |  |  | 6 |  |  |  | 1 |  |  |  |  |  |  |  |  | 1 | 735 |  |  | 1 |  |  |  |  |  |  |  |
| CCG | 374 |  |  |  |  | 1 | 2 | 67 | 1 |  |  |  |  |  |  |  |  |  |  | 327 |  | 5,339 | 1,008 | 69 | 601 |  | 1 | 263 |  | 3 |  | 1,417 | 1 |  |  | 1 |  | 2 | 1 | 99 | 1 |  |  |  |  |  |  |  |  |  |  |  |  |  |  |  | 1 | 219 |  |  |  |  |  |  |  |  |  |
| CCT | 208 |  |  |  |  |  | 1 |  | 189 | 13 |  |  |  |  |  |  |  |  |  |  | 122 | 342 | 3,033 | 426 | 38 |  |  | 1 | 90 | 1 |  | 1 | 440 |  |  |  |  |  | 1 |  | 170 |  |  | 1 |  |  |  |  |  |  |  |  |  |  |  |  |  |  | 680 |  |  |  |  |  |  |  |  |
| CGA | 23 |  |  |  |  |  |  |  |  | 331 | 1 |  |  |  |  |  |  | 675 |  | 1 | 1 | 70 |  |  |  | 8 | 178 | 793 | 92 | 22 |  | 1 | 1 |  |  |  |  |  |  |  |  | 71 |  |  |  |  |  |  |  |  |  |  |  |  |  |  |  |  |  | 200 |  |  |  |  |  |  |  |
| CGC | 129 |  |  |  |  |  |  |  |  |  | 126 | 1 |  |  |  |  |  | 1 | 1,132 | 9 |  |  | 150 | 9 | 1 | 204 | 79 | 366 | 1,467 |  | 66 | 2 |  | 4 |  |  |  |  | 1 | 1 |  |  | 167 |  |  |  |  |  |  |  |  |  |  |  |  |  |  |  |  |  | 787 | 1 |  |  |  |  |  |
| CGG | 220 |  |  |  |  |  |  | 1 |  |  |  | 750 |  |  |  |  |  |  | 3 | 1,854 | 1 | 1 | 3 | 259 | 1 | 965 | 295 | 63 | 249 | 1 | 4 | 171 | 1 |  |  |  |  |  | 1 | 6 |  |  |  | 180 |  |  |  |  |  |  |  |  |  |  |  |  |  |  |  |  |  | 833 |  |  |  |  |  |
| CGT | 63 |  |  |  |  |  |  |  |  |  |  |  | 65 |  |  |  |  | 7 |  |  | 845 |  | 1 | 2 | 72 | 61 | 1,212 | 210 | 8 |  |  |  | 52 |  |  |  |  |  |  | 1 |  |  |  |  | 70 |  |  |  |  |  |  |  |  |  |  |  |  |  |  |  |  |  | 430 |  |  |  |  |
| CTA | 29 |  |  |  |  |  |  |  |  |  |  |  |  | 65 |  |  |  | 27 |  | 1 |  | 223 |  |  |  | 41 |  |  |  | 3 | 303 | 1,974 | 114 |  |  |  |  |  |  |  |  |  |  |  |  | 78 |  |  |  |  |  |  | 1 |  |  |  |  |  |  |  |  |  |  | 554 |  |  |  |
| CTC | 109 |  |  |  |  |  |  |  | 2 |  |  |  |  |  | 167 |  |  |  | 97 |  | 1 |  | 317 |  | 2 |  | 83 |  |  | 381 | 39 | 804 | 2,504 |  |  |  |  |  |  |  |  |  |  |  |  |  | 154 |  |  |  |  |  |  |  |  |  |  |  | 2 |  |  |  |  |  | 548 |  |  |
| CTG | 207 |  |  |  |  |  |  |  |  | 1 |  |  |  |  |  | 233 |  |  | 1 | 167 | 1 |  | 6 | 964 | 2 | 1 |  | 117 | 10 | 2,691 | 893 | 89 | 534 |  |  |  |  |  | 2 | 1 |  |  | 5 |  |  |  |  | 225 |  |  |  |  |  |  |  |  |  |  | 1 |  |  |  |  | 1 |  | 2,608 |  |
| CTT | 38 |  |  |  |  |  |  |  |  |  |  |  |  |  |  |  | 108 |  |  | 1 | 101 | 1 | 1 |  | 344 |  |  |  | 86 | 144 | 1,745 | 461 | 24 |  |  |  |  |  |  |  | 1 |  |  |  |  |  |  |  | 81 |  |  |  |  |  |  |  |  |  |  |  |  |  |  |  |  |  | 272 |
| GAA | 275 | 628 |  |  |  |  | 2 |  |  |  |  |  |  |  |  |  |  | 180 |  | 2 |  |  |  |  |  |  |  |  |  |  |  |  |  | 40 | 272 | 3,581 | 229 | 119 |  |  | 2 | 337 |  | 3 | 2 | 48 | 1 |  |  |  |  | 24 |  |  |  |  |  |  |  |  |  |  |  |  |  |  |  |
| GAC | 215 | 1 | 692 |  |  |  |  |  |  |  |  |  |  |  |  |  |  |  | 63 |  |  |  |  |  |  |  |  |  |  |  |  |  |  | 349 | 73 | 364 | 4,970 | 1 | 127 | 3 |  | 8 | 411 | 1 | 2 |  | 51 | 1 |  |  |  |  | 53 |  |  |  |  |  |  |  |  |  |  |  |  |  |  |
| GAG | 587 |  |  | 949 |  |  |  |  |  |  | 2 |  |  |  |  |  |  |  |  | 374 |  |  |  | 2 |  | 2 |  |  |  |  |  | 1 |  | 4,356 | 417 | 125 | 310 | 5 | 7 | 186 | 12 | 6 | 8 | 481 | 1 |  |  | 120 |  |  |  |  |  | 43 |  |  |  |  |  | 1 |  |  | 1 |  |  |  |  |
| GAT | 132 |  |  |  | 585 |  |  |  |  |  |  | 1 |  |  |  |  |  |  |  |  | 84 |  |  | 2 |  |  |  |  |  |  |  |  |  | 218 | 3,838 | 293 | 73 |  |  |  | 89 |  |  | 1 | 269 | 1 | 10 |  | 77 |  |  |  |  |  | 67 |  |  |  |  |  |  |  |  |  |  |  | 2 |
| GCA | 141 |  |  |  |  | 1,140 |  |  |  |  | 2 | 8 |  |  | 1 |  |  |  |  | 5 |  | 226 |  |  |  |  | 2 |  |  |  |  | 1 |  | 146 | 1 | 1 | 1 | 33 | 688 | 3,752 | 373 | 139 | 2 |  | 21 | 424 |  |  |  |  |  |  |  |  |  | 181 |  |  |  |  | 5 |  |  |  |  |  |  |
| GCC | 490 |  |  |  |  |  | 1,685 |  | 1 |  | 1 |  |  |  |  |  |  |  | 1 | 1 |  |  | 250 | 3 |  |  | 3 |  |  |  | 4 |  |  |  | 132 | 13 | 1 | 626 | 96 | 893 | 4,595 |  | 254 | 3 | 1 |  | 739 |  |  |  |  |  |  |  |  |  | 420 |  |  |  | 2 |  |  |  |  |  |  |
| GCG | 302 |  |  |  |  |  |  | 590 |  |  | 2 |  |  |  |  |  |  |  |  | 2 |  |  |  | 119 |  |  | 4 | 1 |  |  |  |  |  |  |  | 241 | 4 | 4,743 | 810 | 114 | 491 |  | 17 | 173 | 2 |  |  | 1,348 |  |  |  |  |  |  |  |  |  | 113 |  |  |  |  |  |  |  |  |  |
| GCT | 187 |  |  |  |  |  | 1 |  | 915 |  | 1 |  |  |  |  |  |  |  |  |  |  |  |  |  | 204 |  |  |  |  |  |  |  |  | 11 | 2 |  | 94 | 342 | 3,533 | 425 | 17 |  | 2 | 4 | 187 | 1 |  |  | 446 |  |  |  |  |  |  | 1 |  |  | 216 |  |  |  |  |  |  | 1 |  |
| GGA | 177 | 6 |  |  |  |  |  |  |  | 525 |  | 3 |  |  |  |  | 8 |  |  |  |  |  |  | 9 |  | 72 |  | 2 |  |  |  | 1 | 10 | 431 |  |  |  | 114 | 1 | 4 |  | 39 | 435 | 1,682 | 255 | 68 | 1 | 1 |  |  |  |  |  |  |  |  | 3 |  |  | 34 |  | 1 |  |  |  |  |  |
| GGC | 648 | 1 |  |  | 1 |  | 1 |  |  |  | 1,327 |  |  | 1 |  |  |  |  |  |  |  |  |  |  |  |  | 170 | 2 |  |  | 1 |  |  |  | 502 | 5 |  | 1 | 339 | 9 |  | 552 | 62 | 645 | 2,807 |  | 125 | 2 |  |  |  |  |  |  |  |  |  | 3 |  |  | 135 | 1 | 1 |  |  |  |  |
| GGG | 363 |  |  |  |  | 4 |  |  |  |  |  | 683 |  |  |  |  |  |  |  |  |  |  | 1 | 3 |  |  | 1 | 272 |  |  | 1 | 1 |  | 1 | 5 | 582 |  | 4 | 4 | 131 |  | 1,666 | 602 | 57 | 361 |  | 6 | 153 |  |  |  |  |  |  |  |  |  | 1 |  |  | 1 | 95 | 1 |  |  |  |  |
| GGT | 135 |  |  |  |  |  |  |  |  |  |  | 1 | 620 |  |  |  |  |  |  |  |  |  |  |  |  |  |  | 1 | 89 |  |  |  |  | 1 |  | 30 | 362 |  |  |  | 118 | 249 | 2,008 | 236 | 41 |  |  | 2 | 61 |  |  |  |  |  |  |  |  |  |  |  |  |  | 77 |  |  |  |  |
| GTA | 15 |  |  |  |  |  |  |  |  |  |  |  |  | 816 |  |  |  |  |  |  |  |  |  |  |  |  |  |  |  | 94 |  |  |  | 26 |  | 4 |  | 282 | 1 |  |  | 66 | 3 |  | 2 | 19 | 210 | 1,522 | 184 |  |  |  |  |  |  |  |  |  |  |  |  |  |  | 55 |  |  |  |
| GTC | 86 |  | 3 |  |  |  |  |  |  |  |  |  |  |  | 1,457 | 1 | 1 |  |  |  |  |  |  |  |  |  |  |  |  |  | 199 |  |  |  | 45 | 2 |  |  | 505 | 5 | 2 |  | 70 | 2 | 6 | 203 | 22 | 372 | 1,445 |  |  |  |  |  |  |  |  | 1 |  |  |  |  |  |  | 79 |  |  |
| GTG | 170 |  |  |  |  |  |  |  |  |  |  |  |  |  |  | 1,569 |  |  |  | 4 |  |  | 1 | 1 | 1 |  |  |  | 1 |  |  | 309 |  | 9 |  | 118 |  |  | 1 | 1,074 | 5 | 1 | 2 | 127 | 7 | 2,037 | 450 | 57 | 397 |  |  |  |  |  |  |  |  |  |  |  |  |  |  |  |  | 161 |  |
| GTT | 54 |  |  |  |  | 9 |  |  |  |  |  |  | 1 |  |  |  | 869 |  |  |  |  |  |  |  |  |  |  |  |  |  |  |  | 98 | 6 |  |  | 51 | 2 |  |  | 389 |  |  |  | 63 | 129 | 1,111 | 378 | 17 |  |  |  |  | 1 |  |  |  |  | 4 |  |  |  |  |  |  |  | 100 |
| NGC | 1 |  |  |  |  |  |  |  |  |  |  |  |  |  |  |  |  |  |  |  |  |  |  |  |  |  |  |  |  |  |  |  |  |  |  |  |  |  |  |  |  |  |  |  |  |  |  |  |  |  |  |  |  |  |  |  |  |  |  |  |  |  |  |  |  |  |  |
| NNN | 3 |  |  |  |  |  |  |  |  |  |  |  |  |  |  |  |  |  |  |  |  |  |  |  |  |  |  |  |  |  |  |  |  |  |  |  |  |  |  |  |  |  |  |  |  |  |  |  |  |  |  |  |  |  |  |  |  |  |  |  |  |  |  |  |  |  |  |
| TAA | 25 | 11 |  |  |  |  |  |  |  |  |  |  |  |  |  |  |  | 23 |  |  |  |  |  |  |  |  |  |  |  |  |  |  |  | 8 |  |  |  |  |  |  |  |  |  |  |  |  |  |  |  |  |  | 15 | 8 | 13 | 5 | 9 |  |  |  | 20 |  |  |  | 5 |  |  |  |
| TAC | 44 |  | 36 |  |  |  |  |  |  |  |  |  |  |  |  |  | 3 |  | 237 |  |  |  |  |  |  |  |  |  |  |  |  |  |  |  | 64 |  |  |  |  |  |  |  |  |  |  |  |  |  |  |  |  | 16 | 12 | 17 | 3,528 | 1 | 53 |  |  |  | 156 | 1 |  |  | 102 |  |  |
| TAG | 15 |  |  | 8 |  |  |  |  |  |  |  |  |  |  |  |  |  |  |  | 22 |  |  |  |  |  |  |  |  |  |  |  |  |  |  |  | 11 |  |  |  |  |  |  |  |  |  |  |  |  |  |  |  | 50 | 4 | 1 | 3 |  |  | 7 |  | 2 |  | 38 |  | 1 |  | 4 |  |
| TAT | 26 |  |  |  | 20 |  |  |  |  |  |  |  |  |  |  |  |  |  |  |  | 231 |  |  |  |  |  |  |  |  |  |  |  |  |  |  |  | 68 |  |  |  |  |  |  |  |  |  |  |  |  |  |  | 8 | 2,611 | 4 | 3 |  |  |  | 70 |  |  |  | 182 | 3 |  |  | 83 |
| TCA | 62 |  |  |  |  | 114 |  |  |  |  |  |  | 1 |  | 1 |  |  |  |  |  |  | 354 |  |  | 1 |  |  |  |  | 1 | 2 |  |  |  |  |  |  | 125 |  |  |  |  |  |  |  |  | 1 |  |  |  |  | 16 | 1 | 1 | 4 | 10 | 410 | 2,438 | 234 | 11 |  |  |  | 200 | 8 | 4 |  |
| TCC | 180 |  |  |  |  |  | 180 |  |  |  | 1 |  |  |  | 1 |  |  |  |  |  |  |  | 599 |  |  |  | 3 |  |  |  | 2 |  |  |  |  |  |  |  | 318 |  |  |  | 1 |  |  |  | 1 | 1 |  |  |  |  | 100 |  |  | 501 | 41 | 431 | 2,993 |  | 80 |  | 3 |  | 222 |  |  |
| TCG | 66 |  |  |  |  |  |  | 88 |  |  |  |  |  |  |  |  |  |  |  |  |  | 1 |  | 157 |  |  |  |  |  |  | 2 |  |  |  |  |  |  |  |  | 58 |  |  |  | 1 | 1 |  |  |  |  |  |  |  |  | 13 |  | 3,083 | 624 | 30 | 373 |  |  | 33 |  |  |  | 606 |  |
| TCT | 91 |  |  |  |  | 1 |  |  | 171 |  |  |  |  |  |  |  |  |  |  |  |  |  | 1 |  | 532 |  |  |  |  |  | 5 |  |  |  |  | 1 | 1 |  | 1 |  | 183 |  |  |  |  |  |  |  |  |  |  |  | 1 |  | 80 | 187 | 2,548 | 275 | 33 |  |  |  | 73 | 4 | 5 |  | 314 |
| TGA | 26 |  |  |  |  |  |  |  |  | 6 |  |  |  |  |  |  |  |  |  |  |  |  |  | 3 |  | 72 |  |  |  |  |  |  | 1 |  |  |  |  |  |  |  |  | 16 |  |  |  |  |  | 1 |  |  |  | 57 |  |  |  | 11 |  |  |  | 8 | 7 | 25 | 10 | 11 |  |  |  |
| TGC | 83 |  |  |  |  |  |  |  |  |  | 65 |  |  |  |  | 1 |  |  |  |  |  |  |  |  |  |  | 494 |  |  |  |  |  |  |  |  |  |  |  |  |  |  |  | 81 | 1 |  |  |  | 3 |  |  |  |  | 203 |  |  |  | 84 |  | 3 | 25 | 38 | 46 | 1,811 |  | 75 |  |  |
| TGG | 71 |  |  |  |  |  |  |  |  |  |  | 40 |  |  |  |  |  |  |  |  |  |  |  |  |  |  |  | 525 |  |  |  | 1 |  |  |  |  |  |  |  |  |  |  |  | 64 |  |  |  | 1 |  |  |  |  |  | 93 | 1 | 1 | 1 | 55 |  | 59 | 58 | 51 | 47 |  |  | 77 | 1 |
| TGT | 48 |  |  |  |  |  |  |  |  |  | 1 |  | 72 |  |  |  |  |  |  |  |  |  |  |  |  |  |  |  | 302 |  |  | 2 |  |  |  |  |  |  |  |  |  |  |  |  | 69 |  |  | 2 |  |  |  |  |  |  | 280 |  | 2 |  | 110 | 10 | 1,448 | 81 | 7 |  | 2 | 1 | 70 |
| TTA | 30 |  |  |  |  |  |  |  |  |  |  |  |  | 75 |  |  |  |  |  |  |  |  |  |  |  |  |  |  |  | 440 |  |  |  |  |  |  |  |  |  |  |  |  |  |  |  | 57 |  |  | 1 |  |  | 5 |  |  |  | 151 |  |  |  | 2 |  |  |  | 12 | 72 | 599 | 67 |
| TTC | 57 |  |  |  |  |  |  |  |  |  |  |  |  |  | 71 |  |  |  |  |  |  |  |  |  | 1 |  |  |  |  |  | 408 |  | 2 |  |  |  |  |  | 1 |  |  |  |  |  |  |  | 97 |  |  |  |  |  | 90 |  |  |  | 201 |  |  |  | 83 |  |  | 63 | 26 | 84 | 2,382 |
| TTG | 45 | 1 |  |  |  |  |  |  |  |  |  |  |  |  |  | 115 | 1 |  |  |  |  |  |  |  |  |  |  |  |  |  |  | 1,996 |  |  | 1 |  |  |  |  |  |  |  |  |  |  |  |  | 146 | 2 |  |  |  |  | 20 |  |  |  | 426 | 9 |  |  | 49 |  | 867 | 157 | 17 | 94 |
| TTT | 76 |  |  |  |  |  |  |  |  |  |  |  |  |  |  |  | 68 |  |  |  |  |  |  |  |  |  |  |  | 1 | 1 |  |  | 235 |  |  |  |  |  |  |  |  |  |  |  |  |  |  | 2 | 87 |  |  |  |  |  | 77 |  |  |  | 212 |  |  |  | 72 | 73 | 2,055 | 65 | 12 |


---


 **Amino acid changes**

How to read this table:   
- Rows are reference amino acids and columns are changed amino acids. E.g. Row 'A' column 'E' indicates how many 'A' amino acids have been replaced by 'E' amino acids.  
- Red background colors indicate that more changes happened (heat-map).  
- Diagonals are indicated using grey background color   
- WARNING: This table may include different translation codon tables (e.g. mamalian DNA and mitochondrial DNA).

|  | \* | - | ? | A | C | D | E | F | G | H | I | K | L | M | N | P | Q | R | S | T | V | W | Y |
| --- | --- | --- | --- | --- | --- | --- | --- | --- | --- | --- | --- | --- | --- | --- | --- | --- | --- | --- | --- | --- | --- | --- | --- |
| \* | 166 | 55 | 11 |  | 17 |  | 19 |  | 16 |  |  | 19 | 22 |  |  | 3 | 45 | 78 | 27 |  | 1 | 63 | 20 |
| - | 35 |  | 1,600 | 969 | 83 | 239 | 547 | 80 | 1,040 | 182 | 76 | 137 | 395 | 21 | 140 | 943 | 352 | 431 | 511 | 223 | 249 | 36 | 30 |
| ? |  | 4 | 1 |  |  |  |  |  |  |  |  |  |  |  |  |  |  |  |  |  |  |  |  |
| A |  | 1,120 |  | 21,531 | 7 | 235 | 412 |  | 805 | 1 | 1 |  | 6 |  |  | 802 | 8 | 18 | 937 | 4,332 | 2,958 |  |  |
| C | 35 | 131 |  |  | 3,304 |  |  | 147 | 151 |  |  |  | 3 | 1 |  |  |  | 796 | 337 |  | 5 | 127 | 483 |
| D |  | 343 | 4 | 220 |  | 8,954 | 1,224 | 2 | 692 | 147 |  | 1 |  |  | 1,277 | 2 |  | 1 |  |  | 140 |  | 120 |
| E | 68 | 862 |  | 331 | 1 | 1,228 | 8,102 |  | 838 |  |  | 1,577 | 1 |  |  | 2 | 556 | 2 | 2 | 2 | 169 |  |  |
| F |  | 133 |  | 1 | 155 |  |  | 4,475 |  |  | 139 |  | 931 |  |  | 1 |  | 1 | 413 |  | 186 |  | 167 |
| G | 34 | 1,322 | 1 | 725 | 215 | 869 | 1,050 |  | 11,697 |  | 9 | 7 | 14 |  | 1 | 13 |  | 1,821 | 1,954 | 5 | 419 | 97 |  |
| H |  | 197 |  | 3 |  | 183 |  |  |  | 5,519 |  |  | 134 |  | 238 | 243 | 682 | 1,420 |  |  | 8 |  | 556 |
| I |  | 117 |  | 5 |  | 3 | 2 | 172 |  | 2 | 6,404 | 44 | 318 | 595 | 112 | 1 |  | 59 | 142 | 1,007 | 2,408 |  |  |
| K | 29 | 282 |  |  |  |  | 1,137 |  | 1 |  | 39 | 5,190 |  | 70 | 518 | 1 | 386 | 1,284 |  | 219 |  |  |  |
| L | 27 | 458 |  | 4 |  | 1 |  | 1,210 | 5 | 201 | 416 | 1 | 19,797 | 348 |  | 1,860 | 196 | 339 | 589 | 2 | 744 | 49 | 1 |
| M |  | 104 |  |  |  | 6 |  | 2 |  | 1 | 835 | 118 | 527 | 32 | 9 | 1 |  | 83 | 10 | 1,206 | 1,080 |  | 2 |
| N | 1 | 139 |  |  |  | 837 | 7 |  | 5 | 192 | 92 | 413 |  |  | 6,725 |  |  | 3 | 1,610 | 218 | 1 |  | 112 |
| P | 1 | 1,355 |  | 649 |  | 1 | 2 |  | 8 | 298 |  |  | 2,673 |  |  | 22,050 | 459 | 640 | 2,125 | 686 | 1 |  |  |
| Q | 217 | 781 |  | 1 |  |  | 525 |  | 1 | 610 |  | 429 | 168 |  |  | 355 | 5,278 | 1,915 | 5 |  |  |  |  |
| R | 218 | 648 |  | 12 | 1,217 |  | 11 |  | 1,445 | 1,982 | 68 | 1,296 | 321 | 62 | 1 | 570 | 2,556 | 10,456 | 560 | 118 | 2 | 884 |  |
| S | 41 | 794 |  | 686 | 302 | 1 | 12 | 549 | 1,303 |  | 148 | 11 | 828 | 1 | 1,699 | 1,654 | 5 | 541 | 19,634 | 984 | 3 | 33 | 186 |
| T |  | 367 |  | 3,225 |  | 8 | 2 | 1 |  | 1 | 1,355 | 191 |  | 1,381 | 320 | 418 |  | 219 | 833 | 18,089 |  | 1 | 1 |
| V | 1 | 325 |  | 2,266 |  | 96 | 165 | 179 | 349 |  | 3,143 |  | 916 | 1,570 | 3 | 3 | 4 | 1 | 6 | 9 | 8,553 |  |  |
| W | 152 | 71 |  |  | 105 |  |  | 1 | 64 |  |  |  | 78 |  |  |  |  | 565 | 57 |  | 1 | 51 | 1 |
| Y | 45 | 70 |  |  | 338 | 132 |  | 185 |  | 468 | 3 |  | 3 |  | 56 |  |  |  | 124 |  |  | 1 | 6,154 |


---


 **Variants by chromosome**

```
		  

		NC_006583_3, Position,0,1000000,2000000,3000000,4000000,5000000,6000000,7000000,8000000,9000000,10000000,11000000,12000000,13000000,14000000,15000000,16000000,17000000,18000000,19000000,20000000,21000000,22000000,23000000,24000000,25000000,26000000,27000000,28000000,29000000,30000000,31000000,32000000,33000000,34000000,35000000,36000000,37000000,38000000,39000000,40000000,41000000,42000000,43000000,44000000,45000000,46000000,47000000,48000000,49000000,50000000,51000000,52000000,53000000,54000000,55000000,56000000,57000000,58000000,59000000,60000000,61000000,62000000,63000000,64000000,65000000,66000000,67000000,68000000,69000000,70000000,71000000,72000000,73000000,74000000,75000000,76000000,77000000,78000000,79000000,80000000,81000000,82000000,83000000,84000000,85000000,86000000,87000000,88000000,89000000,90000000,91000000,92000000,93000000,94000000,95000000,96000000,97000000,98000000,99000000,100000000,101000000,102000000,103000000,104000000,105000000,106000000,107000000,108000000,109000000,110000000,111000000,112000000,113000000,114000000,115000000,116000000,117000000,118000000,119000000,120000000,121000000,122000000
NC_006583_3,Count,5583,8319,7711,6819,6572,6285,6275,6366,6316,6102,6256,5950,6116,7026,6514,6472,6901,7670,7060,6219,6235,6539,7102,7341,8051,5870,5942,6391,5795,6290,6204,5932,5473,5738,5583,5941,5777,5628,5881,6248,6295,6190,5266,5628,6097,5786,6110,6355,6461,7091,6105,5307,6715,7113,8302,9135,7759,5938,5785,5675,4925,4924,6054,6623,7006,5447,6550,6627,7831,6344,5902,5560,6527,5958,6461,5755,5855,5307,5670,5553,6200,6035,6514,6075,5791,6252,6562,6171,6098,7090,6486,5978,5876,6031,6961,8415,9029,7965,7461,5732,5299,5223,6743,4006,4295,5694,6440,6519,6991,6505,6779,6050,6725,6771,7025,5351,6067,7286,8201,8233,9166,9112,5659

	
```

```
		  

		NC_006584_3, Position,0,1000000,2000000,3000000,4000000,5000000,6000000,7000000,8000000,9000000,10000000,11000000,12000000,13000000,14000000,15000000,16000000,17000000,18000000,19000000,20000000,21000000,22000000,23000000,24000000,25000000,26000000,27000000,28000000,29000000,30000000,31000000,32000000,33000000,34000000,35000000,36000000,37000000,38000000,39000000,40000000,41000000,42000000,43000000,44000000,45000000,46000000,47000000,48000000,49000000,50000000,51000000,52000000,53000000,54000000,55000000,56000000,57000000,58000000,59000000,60000000,61000000,62000000,63000000,64000000,65000000,66000000,67000000,68000000,69000000,70000000,71000000,72000000,73000000,74000000,75000000,76000000,77000000,78000000,79000000,80000000,81000000,82000000,83000000,84000000,85000000
NC_006584_3,Count,4559,5466,5722,5639,1169,294,2799,5526,5264,5940,5387,5250,5539,4893,5330,5791,6377,5849,3671,5469,5753,6150,6539,6306,6898,6932,6247,6375,7015,6900,7352,8378,9394,9288,7651,5271,5749,5660,5913,5971,5845,6429,5652,5032,5642,6055,5313,5583,5926,6150,5929,5320,6396,6715,6560,7077,8419,6216,6920,6888,7225,6900,5827,6863,7357,7053,6628,5666,6061,6114,8627,6277,5898,6303,7343,7326,7622,7029,7892,8600,10597,7745,10079,8982,9793,3408

	
```

```
		  

		NC_006585_3, Position,0,1000000,2000000,3000000,4000000,5000000,6000000,7000000,8000000,9000000,10000000,11000000,12000000,13000000,14000000,15000000,16000000,17000000,18000000,19000000,20000000,21000000,22000000,23000000,24000000,25000000,26000000,27000000,28000000,29000000,30000000,31000000,32000000,33000000,34000000,35000000,36000000,37000000,38000000,39000000,40000000,41000000,42000000,43000000,44000000,45000000,46000000,47000000,48000000,49000000,50000000,51000000,52000000,53000000,54000000,55000000,56000000,57000000,58000000,59000000,60000000,61000000,62000000,63000000,64000000,65000000,66000000,67000000,68000000,69000000,70000000,71000000,72000000,73000000,74000000,75000000,76000000,77000000,78000000,79000000,80000000,81000000,82000000,83000000,84000000,85000000,86000000,87000000,88000000,89000000,90000000,91000000
NC_006585_3,Count,5800,5829,7191,5311,6373,5112,5995,5993,6299,6027,6345,6012,6196,5910,5777,4815,5492,5058,4982,4739,5102,5590,5092,5754,5982,5608,6181,5924,5462,5674,6023,3485,5934,6112,5974,5492,5562,6051,6502,6306,6876,5569,6085,6157,5756,5921,6857,6311,6852,6655,6384,7409,6333,5948,5947,6378,7341,8512,7404,9107,9422,6099,7123,5887,6618,6054,6173,6499,6601,8138,7375,7196,5137,5906,7310,6071,6212,6376,6085,6419,6178,6365,6542,7061,7771,7484,6798,7916,7728,7689,8074,7081

	
```

```
		  

		NC_006586_3, Position,0,1000000,2000000,3000000,4000000,5000000,6000000,7000000,8000000,9000000,10000000,11000000,12000000,13000000,14000000,15000000,16000000,17000000,18000000,19000000,20000000,21000000,22000000,23000000,24000000,25000000,26000000,27000000,28000000,29000000,30000000,31000000,32000000,33000000,34000000,35000000,36000000,37000000,38000000,39000000,40000000,41000000,42000000,43000000,44000000,45000000,46000000,47000000,48000000,49000000,50000000,51000000,52000000,53000000,54000000,55000000,56000000,57000000,58000000,59000000,60000000,61000000,62000000,63000000,64000000,65000000,66000000,67000000,68000000,69000000,70000000,71000000,72000000,73000000,74000000,75000000,76000000,77000000,78000000,79000000,80000000,81000000,82000000,83000000,84000000,85000000,86000000,87000000,88000000
NC_006586_3,Count,5725,5602,4979,5844,5388,6346,6092,6466,6387,6553,5957,5864,6236,5871,6015,5827,5463,5735,4958,5018,6939,8133,8102,4968,4734,5811,6142,7387,9208,8207,6083,6229,7604,7608,8413,6687,5744,6973,6525,6089,5691,6526,5952,6083,5506,5634,5675,6001,5581,5763,6486,6079,6304,6640,6094,6477,6262,6504,6763,6759,6496,5202,5164,5661,5735,5707,5748,5782,6023,5643,6163,4756,6275,6447,5798,6116,5879,6294,6475,6004,6100,6639,7105,7023,6842,7172,9070,8738,2767

	
```

```
		  

		NC_006587_3, Position,0,1000000,2000000,3000000,4000000,5000000,6000000,7000000,8000000,9000000,10000000,11000000,12000000,13000000,14000000,15000000,16000000,17000000,18000000,19000000,20000000,21000000,22000000,23000000,24000000,25000000,26000000,27000000,28000000,29000000,30000000,31000000,32000000,33000000,34000000,35000000,36000000,37000000,38000000,39000000,40000000,41000000,42000000,43000000,44000000,45000000,46000000,47000000,48000000,49000000,50000000,51000000,52000000,53000000,54000000,55000000,56000000,57000000,58000000,59000000,60000000,61000000,62000000,63000000,64000000,65000000,66000000,67000000,68000000,69000000,70000000,71000000,72000000,73000000,74000000,75000000,76000000,77000000,78000000,79000000,80000000,81000000,82000000,83000000,84000000,85000000,86000000,87000000,88000000
NC_006587_3,Count,7107,7185,7635,7720,6764,7412,6172,7016,7269,6782,6429,7038,6535,7473,7345,6530,6638,7813,6601,7374,6332,5971,6288,6135,6198,5646,6268,6494,6539,6778,8083,8354,6498,6868,7032,7075,6406,6951,6876,5966,5902,6810,6783,5516,5722,5524,5678,5510,5540,5977,6621,6271,6854,7312,7387,6726,6539,8356,8991,7804,6252,6828,6694,6075,7908,8096,8163,8216,8087,6483,6868,6428,7116,6798,5656,6339,5901,5785,5161,5923,5768,5754,6610,8494,7241,7249,7998,7722,7157

	
```

```
		  

		NC_006588_3, Position,0,1000000,2000000,3000000,4000000,5000000,6000000,7000000,8000000,9000000,10000000,11000000,12000000,13000000,14000000,15000000,16000000,17000000,18000000,19000000,20000000,21000000,22000000,23000000,24000000,25000000,26000000,27000000,28000000,29000000,30000000,31000000,32000000,33000000,34000000,35000000,36000000,37000000,38000000,39000000,40000000,41000000,42000000,43000000,44000000,45000000,46000000,47000000,48000000,49000000,50000000,51000000,52000000,53000000,54000000,55000000,56000000,57000000,58000000,59000000,60000000,61000000,62000000,63000000,64000000,65000000,66000000,67000000,68000000,69000000,70000000,71000000,72000000,73000000,74000000,75000000,76000000,77000000
NC_006588_3,Count,3906,5852,7023,5366,6990,7385,7126,6846,6643,5617,6719,6018,6463,5980,7563,7639,7255,5868,6835,7470,7118,6558,6886,5421,5544,5693,6580,7043,6759,6304,7263,7986,7147,7827,6800,5660,6572,6206,8242,9577,6333,6234,6185,5253,6050,5588,5842,5520,6479,5942,6131,5431,5619,6064,6521,5741,5499,5680,6309,5669,6112,5748,5829,6028,6482,5765,6378,6469,6435,6415,7098,7023,6221,6785,7221,7093,7692,5139

	
```

```
		  

		NC_006589_3, Position,0,1000000,2000000,3000000,4000000,5000000,6000000,7000000,8000000,9000000,10000000,11000000,12000000,13000000,14000000,15000000,16000000,17000000,18000000,19000000,20000000,21000000,22000000,23000000,24000000,25000000,26000000,27000000,28000000,29000000,30000000,31000000,32000000,33000000,34000000,35000000,36000000,37000000,38000000,39000000,40000000,41000000,42000000,43000000,44000000,45000000,46000000,47000000,48000000,49000000,50000000,51000000,52000000,53000000,54000000,55000000,56000000,57000000,58000000,59000000,60000000,61000000,62000000,63000000,64000000,65000000,66000000,67000000,68000000,69000000,70000000,71000000,72000000,73000000,74000000,75000000,76000000,77000000,78000000,79000000,80000000
NC_006589_3,Count,8160,4801,6160,5155,6124,5487,7185,7031,6538,6898,6406,5821,5916,5753,5883,5882,6172,6274,5627,5449,5788,6078,5925,5941,5494,5425,5502,5254,5430,6112,6521,5733,5593,6492,5666,5950,6545,5657,6813,6668,6729,6531,6191,6454,6877,5882,5406,5168,5681,5480,5946,5936,6140,5522,5896,5636,4935,6044,6241,5696,6372,6453,5820,6332,6235,6159,5401,6129,5656,5647,6673,6015,6223,6808,6960,6109,7010,6803,6608,7700,7692

	
```

```
		  

		NC_006590_3, Position,0,1000000,2000000,3000000,4000000,5000000,6000000,7000000,8000000,9000000,10000000,11000000,12000000,13000000,14000000,15000000,16000000,17000000,18000000,19000000,20000000,21000000,22000000,23000000,24000000,25000000,26000000,27000000,28000000,29000000,30000000,31000000,32000000,33000000,34000000,35000000,36000000,37000000,38000000,39000000,40000000,41000000,42000000,43000000,44000000,45000000,46000000,47000000,48000000,49000000,50000000,51000000,52000000,53000000,54000000,55000000,56000000,57000000,58000000,59000000,60000000,61000000,62000000,63000000,64000000,65000000,66000000,67000000,68000000,69000000,70000000,71000000,72000000,73000000,74000000
NC_006590_3,Count,5736,7289,5654,5787,6107,5768,4906,5399,5346,5401,5052,5506,5609,5437,5358,5189,5918,5705,5595,5881,5693,5323,5129,5612,6059,5534,5507,5860,5543,5409,5603,5516,5949,5544,6144,5199,5536,5931,5664,6084,5439,5253,5739,6130,5746,6251,5982,5522,5951,7213,6544,6394,6025,6024,6629,5979,6388,6798,6849,6010,6788,7127,6507,8361,8773,7873,7686,7720,8116,8129,7001,8644,10227,5016,1768

	
```

```
		  

		NC_006591_3, Position,0,1000000,2000000,3000000,4000000,5000000,6000000,7000000,8000000,9000000,10000000,11000000,12000000,13000000,14000000,15000000,16000000,17000000,18000000,19000000,20000000,21000000,22000000,23000000,24000000,25000000,26000000,27000000,28000000,29000000,30000000,31000000,32000000,33000000,34000000,35000000,36000000,37000000,38000000,39000000,40000000,41000000,42000000,43000000,44000000,45000000,46000000,47000000,48000000,49000000,50000000,51000000,52000000,53000000,54000000,55000000,56000000,57000000,58000000,59000000,60000000,61000000
NC_006591_3,Count,9869,8945,9219,8875,7044,6880,9358,3726,4293,5834,5374,5538,5591,6440,6658,6675,5193,5209,4400,6111,5771,6118,5244,71329,5661,6395,6565,6780,5943,6008,6012,6366,6877,4974,4607,5305,5895,5931,7076,7619,6025,5631,6185,5037,5773,6421,5571,6590,7346,8869,9253,7917,6893,7486,7573,6856,6615,5788,6230,5968,7631,435

	
```

```
		  

		NC_006592_3, Position,0,1000000,2000000,3000000,4000000,5000000,6000000,7000000,8000000,9000000,10000000,11000000,12000000,13000000,14000000,15000000,16000000,17000000,18000000,19000000,20000000,21000000,22000000,23000000,24000000,25000000,26000000,27000000,28000000,29000000,30000000,31000000,32000000,33000000,34000000,35000000,36000000,37000000,38000000,39000000,40000000,41000000,42000000,43000000,44000000,45000000,46000000,47000000,48000000,49000000,50000000,51000000,52000000,53000000,54000000,55000000,56000000,57000000,58000000,59000000,60000000,61000000,62000000,63000000,64000000,65000000,66000000,67000000,68000000,69000000
NC_006592_3,Count,5691,5765,6131,5580,5837,6210,6256,5763,6567,6776,6001,6820,5743,6448,6637,6337,5768,10388,10978,9349,8190,9112,7980,6661,5339,6875,6773,8155,7507,6870,6313,6303,6343,6759,7234,5675,6680,7460,6757,5974,6417,6430,5636,5322,5849,6550,5439,6129,6919,6292,6039,6319,5923,5757,5868,6432,6068,5912,4900,5938,6030,5376,5929,5371,7155,6952,7274,8493,7654,2917

	
```

```
		  

		NC_006593_3, Position,0,1000000,2000000,3000000,4000000,5000000,6000000,7000000,8000000,9000000,10000000,11000000,12000000,13000000,14000000,15000000,16000000,17000000,18000000,19000000,20000000,21000000,22000000,23000000,24000000,25000000,26000000,27000000,28000000,29000000,30000000,31000000,32000000,33000000,34000000,35000000,36000000,37000000,38000000,39000000,40000000,41000000,42000000,43000000,44000000,45000000,46000000,47000000,48000000,49000000,50000000,51000000,52000000,53000000,54000000,55000000,56000000,57000000,58000000,59000000,60000000,61000000,62000000,63000000,64000000,65000000,66000000,67000000,68000000,69000000,70000000,71000000,72000000,73000000,74000000
NC_006593_3,Count,4510,5490,5992,6109,5731,5869,6452,5863,5001,1696,3666,2255,5619,5609,6069,5654,5757,5605,5171,5102,5530,6400,5842,6531,6955,5685,5589,5251,6068,5968,5631,5675,5467,5935,5545,5880,5223,5177,5579,6015,5231,5319,5496,5775,5753,5689,5710,5521,5448,5616,5486,5036,5361,5690,6159,7190,6285,5694,6261,6032,6436,5931,6345,7140,6728,6651,5754,7032,9219,7027,9096,8100,8232,9946,2967

	
```

```
		  

		NC_006594_3, Position,0,1000000,2000000,3000000,4000000,5000000,6000000,7000000,8000000,9000000,10000000,11000000,12000000,13000000,14000000,15000000,16000000,17000000,18000000,19000000,20000000,21000000,22000000,23000000,24000000,25000000,26000000,27000000,28000000,29000000,30000000,31000000,32000000,33000000,34000000,35000000,36000000,37000000,38000000,39000000,40000000,41000000,42000000,43000000,44000000,45000000,46000000,47000000,48000000,49000000,50000000,51000000,52000000,53000000,54000000,55000000,56000000,57000000,58000000,59000000,60000000,61000000,62000000,63000000,64000000,65000000,66000000,67000000,68000000,69000000,70000000,71000000,72000000
NC_006594_3,Count,5938,5504,5769,6019,5566,6005,5805,5352,6465,7114,6511,5279,6340,5319,6335,6401,5814,6011,6146,6357,6277,6121,5458,5293,4938,5399,5502,5652,5601,5390,6004,5084,5393,5394,4728,5139,5413,5077,5566,5062,5080,5399,5239,5368,5682,6228,5792,5426,5440,5649,5758,5899,5877,5614,5844,5520,5373,5798,5714,5556,5740,6013,6392,6617,6367,6345,6075,6090,8015,7497,8226,8209,4608

	
```

```
		  

		NC_006595_3, Position,0,1000000,2000000,3000000,4000000,5000000,6000000,7000000,8000000,9000000,10000000,11000000,12000000,13000000,14000000,15000000,16000000,17000000,18000000,19000000,20000000,21000000,22000000,23000000,24000000,25000000,26000000,27000000,28000000,29000000,30000000,31000000,32000000,33000000,34000000,35000000,36000000,37000000,38000000,39000000,40000000,41000000,42000000,43000000,44000000,45000000,46000000,47000000,48000000,49000000,50000000,51000000,52000000,53000000,54000000,55000000,56000000,57000000,58000000,59000000,60000000,61000000,62000000,63000000
NC_006595_3,Count,5407,4876,5583,5460,5866,5459,5383,5705,5222,5426,5419,5618,5401,5511,5642,5865,6304,6669,5888,6381,6426,6814,6801,6764,6548,7221,5539,7540,6535,8227,7962,7378,6603,8324,9038,8996,8841,9054,8330,8169,6170,6793,6244,6644,6653,7502,7095,6167,5597,6133,6039,5833,6281,6099,5878,6220,6063,6149,5256,5465,6057,6536,6904,993

	
```

```
		  

		NC_006596_3, Position,0,1000000,2000000,3000000,4000000,5000000,6000000,7000000,8000000,9000000,10000000,11000000,12000000,13000000,14000000,15000000,16000000,17000000,18000000,19000000,20000000,21000000,22000000,23000000,24000000,25000000,26000000,27000000,28000000,29000000,30000000,31000000,32000000,33000000,34000000,35000000,36000000,37000000,38000000,39000000,40000000,41000000,42000000,43000000,44000000,45000000,46000000,47000000,48000000,49000000,50000000,51000000,52000000,53000000,54000000,55000000,56000000,57000000,58000000,59000000,60000000
NC_006596_3,Count,4295,5568,6113,5937,6222,6251,5505,5349,5862,5325,5430,5314,5740,5740,5943,6060,5618,5290,5481,5630,5354,5509,6111,5343,5552,5598,5541,5951,5880,5605,5516,5909,5174,5313,5055,6278,5958,5691,6372,6652,5460,6222,6827,7301,6235,5421,6569,7131,6869,5748,5972,6099,5730,5527,6286,6407,7626,6436,7055,8208,7879

	
```

```
		  

		NC_006597_3, Position,0,1000000,2000000,3000000,4000000,5000000,6000000,7000000,8000000,9000000,10000000,11000000,12000000,13000000,14000000,15000000,16000000,17000000,18000000,19000000,20000000,21000000,22000000,23000000,24000000,25000000,26000000,27000000,28000000,29000000,30000000,31000000,32000000,33000000,34000000,35000000,36000000,37000000,38000000,39000000,40000000,41000000,42000000,43000000,44000000,45000000,46000000,47000000,48000000,49000000,50000000,51000000,52000000,53000000,54000000,55000000,56000000,57000000,58000000,59000000,60000000,61000000,62000000,63000000,64000000
NC_006597_3,Count,5408,6482,5772,5657,6888,9200,5999,9667,6695,5556,4981,5308,7827,6900,5236,5611,6601,5966,6382,6479,4956,5683,5910,5994,6048,5285,5312,5426,5817,5356,6007,5828,6246,5740,6258,5718,5475,5904,5529,5748,5814,5799,7080,5808,5878,5992,5985,6214,5026,6632,6060,6221,6461,6643,6328,6389,6179,6567,6274,6989,6337,7360,7372,8176,1468

	
```

```
		  

		NC_006598_3, Position,0,1000000,2000000,3000000,4000000,5000000,6000000,7000000,8000000,9000000,10000000,11000000,12000000,13000000,14000000,15000000,16000000,17000000,18000000,19000000,20000000,21000000,22000000,23000000,24000000,25000000,26000000,27000000,28000000,29000000,30000000,31000000,32000000,33000000,34000000,35000000,36000000,37000000,38000000,39000000,40000000,41000000,42000000,43000000,44000000,45000000,46000000,47000000,48000000,49000000,50000000,51000000,52000000,53000000,54000000,55000000,56000000,57000000,58000000,59000000
NC_006598_3,Count,9391,5009,6843,6375,5956,5802,6370,6487,5727,5554,3977,5869,6074,4370,6121,6705,6589,7743,8339,7227,8816,6158,6301,6508,6655,5786,5237,6608,6634,6430,6376,6278,5928,5839,6107,6842,6225,5754,5538,5409,5309,6120,6023,6493,6548,6725,6725,6741,6658,6602,5393,6152,7002,7174,7264,9158,8130,8170,8487,523

	
```

```
		  

		NC_006599_3, Position,0,1000000,2000000,3000000,4000000,5000000,6000000,7000000,8000000,9000000,10000000,11000000,12000000,13000000,14000000,15000000,16000000,17000000,18000000,19000000,20000000,21000000,22000000,23000000,24000000,25000000,26000000,27000000,28000000,29000000,30000000,31000000,32000000,33000000,34000000,35000000,36000000,37000000,38000000,39000000,40000000,41000000,42000000,43000000,44000000,45000000,46000000,47000000,48000000,49000000,50000000,51000000,52000000,53000000,54000000,55000000,56000000,57000000,58000000,59000000,60000000,61000000,62000000,63000000,64000000
NC_006599_3,Count,9370,10141,7963,7852,7446,7985,6626,8122,7495,6844,6878,7884,6260,6152,6375,6952,5870,6556,6117,5763,6554,4541,6352,6946,7126,5252,6193,6099,5779,5540,6034,5639,5949,5847,6544,6843,6258,5867,5759,6663,6263,6057,6140,6124,6409,5651,5948,6308,6464,6210,5643,6463,5770,7359,6624,5553,5420,5222,5443,5235,6151,6845,6560,7508,1588

	
```

```
		  

		NC_006600_3, Position,0,1000000,2000000,3000000,4000000,5000000,6000000,7000000,8000000,9000000,10000000,11000000,12000000,13000000,14000000,15000000,16000000,17000000,18000000,19000000,20000000,21000000,22000000,23000000,24000000,25000000,26000000,27000000,28000000,29000000,30000000,31000000,32000000,33000000,34000000,35000000,36000000,37000000,38000000,39000000,40000000,41000000,42000000,43000000,44000000,45000000,46000000,47000000,48000000,49000000,50000000,51000000,52000000,53000000,54000000,55000000
NC_006600_3,Count,7800,8478,8075,6644,5718,6093,6143,7199,6457,5803,6325,5929,6800,5750,6570,5979,5470,5689,3469,5862,6002,5777,5825,5478,5685,7580,7180,7903,7509,6440,6521,7582,7781,7708,6082,6455,6138,6245,7246,6064,3943,5915,5854,6846,8864,7534,9064,9420,9087,7147,6098,6359,7177,6607,7035,6074

	
```

```
		  

		NC_006601_3, Position,0,1000000,2000000,3000000,4000000,5000000,6000000,7000000,8000000,9000000,10000000,11000000,12000000,13000000,14000000,15000000,16000000,17000000,18000000,19000000,20000000,21000000,22000000,23000000,24000000,25000000,26000000,27000000,28000000,29000000,30000000,31000000,32000000,33000000,34000000,35000000,36000000,37000000,38000000,39000000,40000000,41000000,42000000,43000000,44000000,45000000,46000000,47000000,48000000,49000000,50000000,51000000,52000000,53000000
NC_006601_3,Count,6510,6849,6608,6788,5838,6097,5517,5831,5563,5325,5405,5452,5130,5220,5670,5871,5334,4903,5288,4537,5024,5931,6048,6323,6315,6494,6248,6348,7298,8027,6264,6938,6402,6402,6380,6498,6684,6822,5551,6786,6194,6264,6092,6315,5775,5917,5907,6397,6365,6219,7284,7564,7271,5899

	
```

```
		  

		NC_006602_3, Position,0,1000000,2000000,3000000,4000000,5000000,6000000,7000000,8000000,9000000,10000000,11000000,12000000,13000000,14000000,15000000,16000000,17000000,18000000,19000000,20000000,21000000,22000000,23000000,24000000,25000000,26000000,27000000,28000000,29000000,30000000,31000000,32000000,33000000,34000000,35000000,36000000,37000000,38000000,39000000,40000000,41000000,42000000,43000000,44000000,45000000,46000000,47000000,48000000,49000000,50000000,51000000,52000000,53000000,54000000,55000000,56000000,57000000,58000000
NC_006602_3,Count,5688,6717,5751,7009,6718,5958,5721,6238,6076,6904,6741,6135,6242,5681,5449,5987,6304,6125,6797,6635,6017,5720,6292,6441,6517,6753,6339,6436,6311,6377,6498,6339,6384,5803,4993,6528,6653,5914,5236,5674,5691,5722,7178,6451,6368,6951,6671,5519,6749,6055,6757,6457,7854,6870,8664,9172,9148,10617,1367

	
```

```
		  

		NC_006603_3, Position,0,1000000,2000000,3000000,4000000,5000000,6000000,7000000,8000000,9000000,10000000,11000000,12000000,13000000,14000000,15000000,16000000,17000000,18000000,19000000,20000000,21000000,22000000,23000000,24000000,25000000,26000000,27000000,28000000,29000000,30000000,31000000,32000000,33000000,34000000,35000000,36000000,37000000,38000000,39000000,40000000,41000000,42000000,43000000,44000000,45000000,46000000,47000000,48000000,49000000,50000000
NC_006603_3,Count,5181,5238,5638,5393,5878,5633,6886,6585,5771,6050,5933,6453,6272,5491,5261,6357,6075,5851,6517,7128,5963,6205,6373,6479,5605,5695,5704,6038,6022,5603,5399,6909,5705,5001,7179,7651,6684,5913,6509,5265,6696,8162,7314,7140,7222,7094,7677,8031,7127,7888,6376

	
```

```
		  

		NC_006604_3, Position,0,1000000,2000000,3000000,4000000,5000000,6000000,7000000,8000000,9000000,10000000,11000000,12000000,13000000,14000000,15000000,16000000,17000000,18000000,19000000,20000000,21000000,22000000,23000000,24000000,25000000,26000000,27000000,28000000,29000000,30000000,31000000,32000000,33000000,34000000,35000000,36000000,37000000,38000000,39000000,40000000,41000000,42000000,43000000,44000000,45000000,46000000,47000000,48000000,49000000,50000000,51000000,52000000,53000000,54000000,55000000,56000000,57000000,58000000,59000000,60000000,61000000
NC_006604_3,Count,7236,6550,6161,5515,6124,6060,6011,5712,5509,6317,6174,5880,5332,5539,5573,5596,5164,5400,5522,5237,5397,5553,5143,4895,5355,5281,5571,5675,5084,5527,5539,6000,5596,5629,5624,5283,5833,5714,5462,5764,5844,5545,5362,5591,6164,6526,5840,6470,6563,5570,5887,6017,6135,6251,6606,7189,7057,7400,8129,7975,9112,3124

	
```

```
		  

		NC_006605_3, Position,0,1000000,2000000,3000000,4000000,5000000,6000000,7000000,8000000,9000000,10000000,11000000,12000000,13000000,14000000,15000000,16000000,17000000,18000000,19000000,20000000,21000000,22000000,23000000,24000000,25000000,26000000,27000000,28000000,29000000,30000000,31000000,32000000,33000000,34000000,35000000,36000000,37000000,38000000,39000000,40000000,41000000,42000000,43000000,44000000,45000000,46000000,47000000,48000000,49000000,50000000,51000000,52000000
NC_006605_3,Count,5907,5644,5201,5428,5680,5567,5863,5974,6390,6465,5975,6829,6929,6680,5912,5398,5733,6648,5929,5690,5545,5940,5687,5423,5785,5258,5643,5848,6108,5764,5810,7073,5475,5402,5816,6716,6861,5864,5958,6080,6101,6036,5637,6044,6671,6914,6521,7104,6814,7574,7292,6663,2581

	
```

```
		  

		NC_006606_3, Position,0,1000000,2000000,3000000,4000000,5000000,6000000,7000000,8000000,9000000,10000000,11000000,12000000,13000000,14000000,15000000,16000000,17000000,18000000,19000000,20000000,21000000,22000000,23000000,24000000,25000000,26000000,27000000,28000000,29000000,30000000,31000000,32000000,33000000,34000000,35000000,36000000,37000000,38000000,39000000,40000000,41000000,42000000,43000000,44000000,45000000,46000000,47000000
NC_006606_3,Count,6099,5673,5655,5900,5993,6630,6678,6017,4881,6051,6040,5621,6098,6238,6059,5942,6211,6157,6682,5580,5794,5955,6243,5265,5521,6021,7031,7625,7241,6433,6869,6614,6099,6916,7279,7136,7189,7979,6895,7274,7006,8181,9743,7319,9331,10686,10913,6795

	
```

```
		  

		NC_006607_3, Position,0,1000000,2000000,3000000,4000000,5000000,6000000,7000000,8000000,9000000,10000000,11000000,12000000,13000000,14000000,15000000,16000000,17000000,18000000,19000000,20000000,21000000,22000000,23000000,24000000,25000000,26000000,27000000,28000000,29000000,30000000,31000000,32000000,33000000,34000000,35000000,36000000,37000000,38000000,39000000,40000000,41000000,42000000,43000000,44000000,45000000,46000000,47000000,48000000,49000000,50000000,51000000
NC_006607_3,Count,5348,5754,5991,4902,5779,5213,5794,5549,5685,6466,6028,5687,6668,5734,6748,6211,5607,5774,5097,5298,5321,5702,5858,6020,5574,5847,7040,6321,5332,5939,7001,6401,6343,6342,6484,6819,6878,6604,6465,6752,6828,7310,6953,6327,7142,8400,9602,9199,9383,10777,5236,5787

	
```

```
		  

		NC_006608_3, Position,0,1000000,2000000,3000000,4000000,5000000,6000000,7000000,8000000,9000000,10000000,11000000,12000000,13000000,14000000,15000000,16000000,17000000,18000000,19000000,20000000,21000000,22000000,23000000,24000000,25000000,26000000,27000000,28000000,29000000,30000000,31000000,32000000,33000000,34000000,35000000,36000000,37000000,38000000
NC_006608_3,Count,6749,6793,7616,7298,7021,6970,5822,6184,5864,5162,7319,8139,7096,6997,6296,6326,5633,6971,6803,7489,8265,5931,5984,6040,5873,1275,4723,5885,5731,7660,6737,5684,8096,7740,7913,8002,8706,8430,9363

	
```

```
		  

		NC_006609_3, Position,0,1000000,2000000,3000000,4000000,5000000,6000000,7000000,8000000,9000000,10000000,11000000,12000000,13000000,14000000,15000000,16000000,17000000,18000000,19000000,20000000,21000000,22000000,23000000,24000000,25000000,26000000,27000000,28000000,29000000,30000000,31000000,32000000,33000000,34000000,35000000,36000000,37000000,38000000,39000000,40000000,41000000,42000000,43000000,44000000,45000000
NC_006609_3,Count,7714,7337,9429,6712,6069,5991,6536,6881,5707,6562,6222,6167,6170,5436,5842,6089,5736,5765,6110,5466,6115,5440,5614,5050,5366,6145,5792,5469,5596,5685,5912,6154,6150,6218,6088,5605,5737,5664,5951,6782,5605,5969,5641,5278,5714,4761

	
```

```
		  

		NC_006610_3, Position,0,1000000,2000000,3000000,4000000,5000000,6000000,7000000,8000000,9000000,10000000,11000000,12000000,13000000,14000000,15000000,16000000,17000000,18000000,19000000,20000000,21000000,22000000,23000000,24000000,25000000,26000000,27000000,28000000,29000000,30000000,31000000,32000000,33000000,34000000,35000000,36000000,37000000,38000000,39000000,40000000,41000000
NC_006610_3,Count,6060,5974,6501,5977,5681,5720,5016,5663,5648,5619,5744,6082,5663,6038,5158,5514,6702,6604,6556,6404,6380,6491,6016,6261,6890,6310,5833,7171,7013,6764,7387,7852,7193,9053,7409,8031,8424,9006,9105,10649,10818,1354

	
```

```
		  

		NC_006611_3, Position,0,1000000,2000000,3000000,4000000,5000000,6000000,7000000,8000000,9000000,10000000,11000000,12000000,13000000,14000000,15000000,16000000,17000000,18000000,19000000,20000000,21000000,22000000,23000000,24000000,25000000,26000000,27000000,28000000,29000000,30000000,31000000,32000000,33000000,34000000,35000000,36000000,37000000,38000000,39000000,40000000,41000000
NC_006611_3,Count,5228,5580,5499,5748,5611,5660,5643,6238,6323,6081,6171,6294,6518,6008,5538,5701,5113,5863,6042,5364,5892,6429,5620,5769,5702,5692,5795,6145,6263,5305,6068,6280,6081,6028,6475,6491,6897,6949,7798,7396,8462,6885

	
```

```
		  

		NC_006612_3, Position,0,1000000,2000000,3000000,4000000,5000000,6000000,7000000,8000000,9000000,10000000,11000000,12000000,13000000,14000000,15000000,16000000,17000000,18000000,19000000,20000000,21000000,22000000,23000000,24000000,25000000,26000000,27000000,28000000,29000000,30000000,31000000,32000000,33000000,34000000,35000000,36000000,37000000,38000000,39000000,40000000
NC_006612_3,Count,5048,5343,5377,4991,5000,5328,5640,5921,5536,5526,5276,6286,6373,5673,5794,5365,5542,6118,5068,5181,5294,5061,6092,6350,6025,5821,6102,6940,5573,6407,7321,6838,7547,8856,7652,6444,6807,8829,8321,6477,1220

	
```

```
		  

		NC_006613_3, Position,0,1000000,2000000,3000000,4000000,5000000,6000000,7000000,8000000,9000000,10000000,11000000,12000000,13000000,14000000,15000000,16000000,17000000,18000000,19000000,20000000,21000000,22000000,23000000,24000000,25000000,26000000,27000000,28000000,29000000,30000000,31000000,32000000,33000000,34000000,35000000,36000000,37000000,38000000,39000000
NC_006613_3,Count,5274,5372,4863,5073,5292,5545,5666,5772,5808,5569,5620,5435,5664,5431,6272,5886,6084,5402,6026,6192,6510,6597,6419,5978,6892,6397,7175,5471,65,3007,7410,7169,6410,7079,7582,8170,10384,10046,10025,8091

	
```

```
		  

		NC_006614_3, Position,0,1000000,2000000,3000000,4000000,5000000,6000000,7000000,8000000,9000000,10000000,11000000,12000000,13000000,14000000,15000000,16000000,17000000,18000000,19000000,20000000,21000000,22000000,23000000,24000000,25000000,26000000,27000000,28000000,29000000,30000000,31000000,32000000,33000000,34000000,35000000,36000000,37000000,38000000
NC_006614_3,Count,6035,6562,6386,6118,5758,6130,6139,6274,6098,6261,6311,5938,5792,5727,5677,5625,5963,5731,5997,5974,5571,5846,5865,5920,5774,5950,5461,5735,5755,5609,5800,5288,5107,5095,5058,5041,5045,4970,3691

	
```

```
		  

		NC_006615_3, Position,0,1000000,2000000,3000000,4000000,5000000,6000000,7000000,8000000,9000000,10000000,11000000,12000000,13000000,14000000,15000000,16000000,17000000,18000000,19000000,20000000,21000000,22000000,23000000,24000000,25000000,26000000,27000000,28000000,29000000,30000000,31000000
NC_006615_3,Count,5686,5914,6046,5666,5481,6584,5341,6092,6187,5876,6046,5581,5360,6190,6282,5922,6542,6286,5707,5930,6208,6988,6339,5926,4928,5921,8019,7941,6344,7314,7853,3234

	
```

```
		  

		NC_006616_3, Position,0,1000000,2000000,3000000,4000000,5000000,6000000,7000000,8000000,9000000,10000000,11000000,12000000,13000000,14000000,15000000,16000000,17000000,18000000,19000000,20000000,21000000,22000000,23000000,24000000,25000000,26000000,27000000,28000000,29000000,30000000,31000000,32000000,33000000,34000000,35000000,36000000,37000000,38000000,39000000,40000000,41000000,42000000
NC_006616_3,Count,5359,5564,5630,6265,6706,6489,7062,8212,7568,8555,8836,8738,5402,5604,4980,5973,5393,5856,5975,7104,6540,6338,6730,5999,6866,6400,5944,6214,5993,5956,6005,6036,6601,6072,5944,7062,7744,6350,6895,7337,8192,8993,781

	
```

```
		  

		NC_006617_3, Position,0,100000,200000,300000,400000,500000,600000,700000,800000,900000,1000000,1100000,1200000,1300000,1400000,1500000,1600000,1700000,1800000,1900000,2000000,2100000,2200000,2300000,2400000,2500000,2600000,2700000,2800000,2900000,3000000,3100000,3200000,3300000,3400000,3500000,3600000,3700000,3800000,3900000,4000000,4100000,4200000,4300000,4400000,4500000,4600000,4700000,4800000,4900000,5000000,5100000,5200000,5300000,5400000,5500000,5600000,5700000,5800000,5900000,6000000,6100000,6200000,6300000,6400000,6500000,6600000,6700000,6800000,6900000,7000000,7100000,7200000,7300000,7400000,7500000,7600000,7700000,7800000,7900000,8000000,8100000,8200000,8300000,8400000,8500000,8600000,8700000,8800000,8900000,9000000,9100000,9200000,9300000,9400000,9500000,9600000,9700000,9800000,9900000,10000000,10100000,10200000,10300000,10400000,10500000,10600000,10700000,10800000,10900000,11000000,11100000,11200000,11300000,11400000,11500000,11600000,11700000,11800000,11900000,12000000,12100000,12200000,12300000,12400000,12500000,12600000,12700000,12800000,12900000,13000000,13100000,13200000,13300000,13400000,13500000,13600000,13700000,13800000,13900000,14000000,14100000,14200000,14300000,14400000,14500000,14600000,14700000,14800000,14900000,15000000,15100000,15200000,15300000,15400000,15500000,15600000,15700000,15800000,15900000,16000000,16100000,16200000,16300000,16400000,16500000,16600000,16700000,16800000,16900000,17000000,17100000,17200000,17300000,17400000,17500000,17600000,17700000,17800000,17900000,18000000,18100000,18200000,18300000,18400000,18500000,18600000,18700000,18800000,18900000,19000000,19100000,19200000,19300000,19400000,19500000,19600000,19700000,19800000,19900000,20000000,20100000,20200000,20300000,20400000,20500000,20600000,20700000,20800000,20900000,21000000,21100000,21200000,21300000,21400000,21500000,21600000,21700000,21800000,21900000,22000000,22100000,22200000,22300000,22400000,22500000,22600000,22700000,22800000,22900000,23000000,23100000,23200000,23300000,23400000,23500000,23600000,23700000,23800000,23900000,24000000,24100000,24200000,24300000,24400000,24500000,24600000,24700000,24800000,24900000,25000000,25100000,25200000,25300000,25400000,25500000,25600000,25700000,25800000,25900000,26000000,26100000,26200000,26300000,26400000,26500000
NC_006617_3,Count,434,566,494,523,451,585,688,921,771,629,656,652,632,778,662,863,744,802,762,681,736,881,685,598,532,558,675,666,814,764,675,766,819,725,735,1071,976,865,781,858,899,811,617,668,675,674,692,688,674,690,813,693,784,722,573,685,651,715,809,837,771,532,713,643,627,614,655,760,820,777,706,646,769,727,804,707,701,697,893,629,591,675,711,564,673,677,596,703,605,612,630,583,601,587,588,571,654,699,702,687,717,701,757,760,705,587,714,755,710,810,735,606,781,748,696,595,620,707,794,695,661,673,639,754,673,725,758,758,820,752,698,625,668,640,717,851,721,719,719,786,732,765,729,696,596,674,763,886,615,694,721,713,678,637,759,696,619,637,640,673,737,679,595,667,625,545,653,629,669,780,741,675,612,631,578,579,664,595,659,674,649,681,675,643,693,713,546,668,704,706,642,597,622,629,593,575,627,630,741,709,715,659,710,690,672,663,681,680,759,672,637,667,652,662,619,742,665,658,602,576,604,608,702,706,685,730,648,616,658,713,690,519,639,685,678,696,684,633,547,595,664,578,707,668,653,684,687,628,724,687,660,531,725,646,561,638,671,714,681,704,389,394,798,708,112,139

	
```

```
		  

		NC_006618_3, Position,0,1000000,2000000,3000000,4000000,5000000,6000000,7000000,8000000,9000000,10000000,11000000,12000000,13000000,14000000,15000000,16000000,17000000,18000000,19000000,20000000,21000000,22000000,23000000,24000000,25000000,26000000,27000000,28000000,29000000,30000000
NC_006618_3,Count,5053,5594,5779,6118,6486,5925,5784,5548,5672,6072,5806,6126,6297,6614,5231,5396,6039,6694,5985,6160,5705,5502,5945,6344,6727,6001,6744,6687,6901,6629,5597

	
```

```
		  

		NC_006619_3, Position,0,1000000,2000000,3000000,4000000,5000000,6000000,7000000,8000000,9000000,10000000,11000000,12000000,13000000,14000000,15000000,16000000,17000000,18000000,19000000,20000000,21000000,22000000,23000000,24000000,25000000,26000000,27000000,28000000,29000000,30000000
NC_006619_3,Count,5217,7143,6991,5880,5911,6739,6267,6187,6164,6451,5797,5160,5629,6328,6098,6168,6114,6056,6237,6247,5518,6223,6937,8441,7776,6767,7454,7271,7598,7054,9122

	
```

```
		  

		NC_006620_3, Position,0,100000,200000,300000,400000,500000,600000,700000,800000,900000,1000000,1100000,1200000,1300000,1400000,1500000,1600000,1700000,1800000,1900000,2000000,2100000,2200000,2300000,2400000,2500000,2600000,2700000,2800000,2900000,3000000,3100000,3200000,3300000,3400000,3500000,3600000,3700000,3800000,3900000,4000000,4100000,4200000,4300000,4400000,4500000,4600000,4700000,4800000,4900000,5000000,5100000,5200000,5300000,5400000,5500000,5600000,5700000,5800000,5900000,6000000,6100000,6200000,6300000,6400000,6500000,6600000,6700000,6800000,6900000,7000000,7100000,7200000,7300000,7400000,7500000,7600000,7700000,7800000,7900000,8000000,8100000,8200000,8300000,8400000,8500000,8600000,8700000,8800000,8900000,9000000,9100000,9200000,9300000,9400000,9500000,9600000,9700000,9800000,9900000,10000000,10100000,10200000,10300000,10400000,10500000,10600000,10700000,10800000,10900000,11000000,11100000,11200000,11300000,11400000,11500000,11600000,11700000,11800000,11900000,12000000,12100000,12200000,12300000,12400000,12500000,12600000,12700000,12800000,12900000,13000000,13100000,13200000,13300000,13400000,13500000,13600000,13700000,13800000,13900000,14000000,14100000,14200000,14300000,14400000,14500000,14600000,14700000,14800000,14900000,15000000,15100000,15200000,15300000,15400000,15500000,15600000,15700000,15800000,15900000,16000000,16100000,16200000,16300000,16400000,16500000,16600000,16700000,16800000,16900000,17000000,17100000,17200000,17300000,17400000,17500000,17600000,17700000,17800000,17900000,18000000,18100000,18200000,18300000,18400000,18500000,18600000,18700000,18800000,18900000,19000000,19100000,19200000,19300000,19400000,19500000,19600000,19700000,19800000,19900000,20000000,20100000,20200000,20300000,20400000,20500000,20600000,20700000,20800000,20900000,21000000,21100000,21200000,21300000,21400000,21500000,21600000,21700000,21800000,21900000,22000000,22100000,22200000,22300000,22400000,22500000,22600000,22700000,22800000,22900000,23000000,23100000,23200000,23300000,23400000,23500000,23600000,23700000,23800000,23900000
NC_006620_3,Count,594,660,721,676,553,504,653,816,709,712,657,817,793,757,704,840,567,690,720,847,787,682,688,844,673,630,702,787,730,517,568,548,538,587,651,583,589,687,734,603,555,608,566,626,622,609,553,626,668,664,565,649,654,608,640,626,665,637,445,631,690,720,726,713,656,676,629,580,648,628,574,544,602,608,563,542,616,667,553,571,637,578,580,700,622,651,630,600,539,518,620,581,603,677,628,558,551,537,581,652,572,549,621,618,644,600,697,594,643,666,691,600,577,620,754,614,593,665,590,587,535,655,679,611,704,716,673,651,545,534,713,625,617,683,676,706,695,658,642,504,587,615,728,636,635,657,712,570,592,697,745,674,607,760,709,736,664,551,644,694,708,662,645,707,807,679,615,741,663,591,594,741,616,745,697,612,697,740,763,840,778,812,765,726,854,742,726,592,727,579,585,685,597,616,633,623,653,685,669,701,775,788,676,954,944,912,885,654,514,603,541,517,769,718,737,614,668,862,799,577,1091,972,1020,889,895,751,804,865,901,694,658,729,541,722,707,1170,569,871,772,109

	
```

```
		  

		NC_006621_3, Position,0,1000000,2000000,3000000,4000000,5000000,6000000,7000000,8000000,9000000,10000000,11000000,12000000,13000000,14000000,15000000,16000000,17000000,18000000,19000000,20000000,21000000,22000000,23000000,24000000,25000000,26000000,27000000,28000000,29000000,30000000,31000000,32000000,33000000,34000000,35000000,36000000,37000000,38000000,39000000,40000000,41000000,42000000,43000000,44000000,45000000,46000000,47000000,48000000,49000000,50000000,51000000,52000000,53000000,54000000,55000000,56000000,57000000,58000000,59000000,60000000,61000000,62000000,63000000,64000000,65000000,66000000,67000000,68000000,69000000,70000000,71000000,72000000,73000000,74000000,75000000,76000000,77000000,78000000,79000000,80000000,81000000,82000000,83000000,84000000,85000000,86000000,87000000,88000000,89000000,90000000,91000000,92000000,93000000,94000000,95000000,96000000,97000000,98000000,99000000,100000000,101000000,102000000,103000000,104000000,105000000,106000000,107000000,108000000,109000000,110000000,111000000,112000000,113000000,114000000,115000000,116000000,117000000,118000000,119000000,120000000,121000000,122000000,123000000
NC_006621_3,Count,7798,8366,8269,8073,8365,6863,7373,5681,5515,6955,6008,5233,5458,5245,4895,5068,4754,4919,5054,4629,4937,5134,4426,4639,5315,5200,5178,4567,5205,5117,5240,4875,5073,5835,6558,4617,5331,5790,4986,5131,4811,5015,4777,4453,3608,4163,3341,3928,2265,4897,5089,5266,4793,5147,4493,4702,4311,4721,4513,4341,4754,4496,4797,4893,5013,4850,5049,4725,5100,5483,5387,5364,5418,5433,5098,4540,4198,4598,4827,4388,4308,4899,4776,4949,4800,4626,4811,5023,5010,4788,4911,5455,4488,4638,4641,4844,5072,4555,5054,5093,4912,4537,4655,5102,5161,4849,4919,5222,4908,5011,5513,6054,5997,5707,5493,4897,4930,4338,4975,5804,4681,7065,5373,4216

	
```

```
		  

		NW_003726127_1, Position,0,10000,20000,30000,40000,50000,60000,70000,80000,90000,100000,110000,120000,130000,140000,150000,160000,170000,180000,190000,200000,210000,220000,230000,240000,250000,260000,270000,280000,290000,300000,310000,320000,330000,340000,350000,360000,370000,380000,390000,400000,410000,420000,430000,440000,450000,460000,470000,480000,490000,500000,510000,520000,530000,540000,550000,560000,570000,580000,590000,600000,610000,620000,630000,640000,650000,660000,670000,680000,690000,700000,710000,720000,730000,740000,750000,760000,770000,780000,790000,800000,810000,820000,830000,840000,850000,860000,870000,880000,890000,900000,910000,920000,930000,940000,950000,960000,970000,980000,990000,1000000,1010000,1020000,1030000,1040000,1050000,1060000,1070000,1080000,1090000,1100000,1110000,1120000,1130000,1140000,1150000,1160000,1170000,1180000,1190000,1200000,1210000,1220000,1230000,1240000,1250000,1260000,1270000,1280000,1290000,1300000,1310000,1320000,1330000,1340000,1350000,1360000,1370000,1380000,1390000,1400000,1410000,1420000,1430000,1440000,1450000,1460000,1470000,1480000,1490000,1500000,1510000,1520000,1530000,1540000,1550000,1560000,1570000,1580000,1590000,1600000,1610000,1620000,1630000,1640000,1650000,1660000,1670000,1680000,1690000,1700000,1710000,1720000,1730000,1740000,1750000,1760000,1770000,1780000,1790000,1800000,1810000,1820000,1830000,1840000,1850000,1860000,1870000,1880000,1890000,1900000,1910000,1920000,1930000,1940000,1950000,1960000,1970000,1980000,1990000,2000000,2010000,2020000,2030000,2040000,2050000,2060000,2070000,2080000,2090000,2100000,2110000,2120000,2130000,2140000,2150000,2160000,2170000,2180000,2190000,2200000,2210000,2220000,2230000,2240000,2250000,2260000,2270000,2280000,2290000,2300000,2310000,2320000,2330000,2340000,2350000,2360000,2370000,2380000,2390000,2400000,2410000,2420000,2430000,2440000,2450000,2460000,2470000,2480000,2490000,2500000,2510000,2520000,2530000,2540000,2550000,2560000,2570000,2580000,2590000,2600000,2610000,2620000,2630000,2640000,2650000,2660000
NW_003726127_1,Count,0,0,3,0,0,0,0,0,0,0,0,0,0,0,1,0,0,0,0,0,0,0,0,0,0,0,0,0,0,2,0,0,0,0,0,0,0,0,1,0,0,0,3,0,0,0,0,0,0,3,0,0,2,0,0,0,0,0,0,0,0,0,0,0,0,0,0,0,0,0,0,0,0,0,2,0,0,0,0,0,0,0,0,0,0,0,0,0,0,0,0,0,0,0,0,0,0,0,0,0,0,0,0,0,0,0,0,0,0,0,0,0,0,0,0,0,0,0,2,2,0,5,4,0,0,0,0,0,0,0,0,0,4,3,0,0,0,0,0,0,0,0,0,0,0,0,0,0,0,0,0,0,0,2,0,0,0,0,0,0,0,0,0,0,0,3,0,0,0,0,0,0,0,0,0,4,0,0,0,0,2,0,0,0,0,0,4,6,0,0,0,0,0,0,0,0,0,0,0,0,0,0,0,0,0,0,0,0,0,0,0,10,0,0,0,0,0,0,1,0,0,0,0,0,0,0,0,0,0,0,0,0,0,0,0,0,5,1,12,3,10,5,20,3,1,8,2,11,0,3,0,0,0,0,1,8,5,14,14,7,11,8,36,0,0,0,0

	
```

```
		  

		NW_003726128_1, Position,0,10000,20000,30000,40000,50000,60000,70000,80000,90000,100000,110000,120000,130000,140000,150000,160000,170000,180000,190000,200000,210000,220000,230000,240000,250000,260000,270000,280000,290000,300000,310000,320000,330000,340000,350000,360000,370000,380000,390000,400000,410000,420000,430000,440000,450000,460000,470000,480000,490000,500000,510000,520000,530000,540000,550000,560000,570000,580000,590000,600000,610000,620000,630000,640000,650000,660000,670000,680000,690000,700000,710000,720000,730000,740000,750000,760000,770000,780000,790000,800000,810000,820000,830000,840000,850000,860000,870000,880000,890000,900000,910000,920000,930000,940000,950000,960000,970000,980000,990000,1000000,1010000,1020000,1030000,1040000,1050000,1060000,1070000,1080000,1090000,1100000,1110000,1120000,1130000,1140000,1150000,1160000,1170000,1180000,1190000,1200000,1210000,1220000,1230000,1240000,1250000,1260000,1270000,1280000,1290000,1300000,1310000,1320000,1330000,1340000,1350000,1360000,1370000,1380000,1390000,1400000,1410000,1420000,1430000,1440000,1450000,1460000,1470000,1480000,1490000,1500000,1510000,1520000,1530000,1540000,1550000,1560000,1570000,1580000,1590000,1600000,1610000,1620000,1630000,1640000,1650000,1660000,1670000,1680000,1690000,1700000,1710000,1720000,1730000,1740000,1750000,1760000,1770000,1780000,1790000,1800000,1810000,1820000,1830000,1840000,1850000,1860000,1870000,1880000
NW_003726128_1,Count,0,0,0,0,0,0,0,0,0,0,0,0,0,0,0,0,0,0,0,0,0,0,0,0,0,0,0,0,0,0,0,0,0,0,0,0,0,0,0,0,0,0,0,0,0,0,0,0,0,0,0,0,0,0,0,0,0,0,0,0,0,0,0,0,0,0,0,0,0,0,0,0,0,0,0,0,0,0,0,0,0,0,0,0,0,0,0,0,0,0,0,0,0,0,0,0,0,0,0,1,0,0,0,0,0,0,0,0,0,0,0,0,0,0,0,0,0,0,0,0,0,0,0,0,0,0,0,0,0,0,0,0,0,0,0,0,0,0,0,0,0,0,0,0,0,0,0,0,0,0,0,0,0,0,0,0,0,0,0,0,0,0,0,0,0,0,0,0,0,0,0,0,0,0,0,0,0,0,0,0,0,0,0,0,0,0,0,0,0

	
```

```
		  

		NW_003726129_1, Position,0,10000,20000,30000,40000,50000,60000,70000,80000,90000,100000,110000,120000,130000,140000,150000,160000,170000,180000,190000,200000,210000,220000,230000,240000,250000,260000,270000,280000,290000,300000,310000,320000,330000,340000,350000,360000,370000,380000,390000,400000,410000,420000,430000,440000,450000,460000,470000,480000,490000,500000,510000,520000,530000,540000,550000,560000,570000,580000,590000,600000,610000,620000,630000,640000,650000,660000,670000,680000,690000,700000,710000,720000,730000,740000,750000,760000,770000,780000,790000,800000,810000,820000,830000,840000,850000,860000,870000,880000,890000,900000,910000,920000,930000,940000,950000,960000,970000,980000,990000,1000000,1010000,1020000,1030000,1040000,1050000,1060000,1070000,1080000,1090000,1100000,1110000,1120000,1130000,1140000,1150000,1160000,1170000,1180000,1190000,1200000,1210000,1220000,1230000,1240000,1250000,1260000,1270000,1280000,1290000,1300000,1310000,1320000,1330000,1340000,1350000,1360000,1370000,1380000,1390000,1400000,1410000
NW_003726129_1,Count,2,0,0,0,6,2,0,0,0,0,0,0,0,0,0,0,0,0,0,0,0,0,0,0,0,0,0,0,0,0,0,0,0,0,0,0,0,0,0,0,0,0,0,0,0,0,0,0,0,0,0,0,0,0,0,0,0,0,0,0,0,0,0,0,0,0,0,0,0,0,0,0,0,0,0,0,0,0,0,0,0,0,0,0,0,0,0,0,0,0,0,0,0,0,0,0,0,0,0,0,0,0,0,0,0,0,0,0,0,0,0,0,0,0,0,0,0,0,0,0,0,0,0,0,0,0,0,0,0,0,0,0,0,0,0,0,0,0,0,0,0,0

	
```

```
		  

		NW_003726130_1, Position,0,10000,20000,30000,40000,50000,60000,70000,80000,90000,100000,110000,120000,130000,140000,150000,160000,170000,180000,190000,200000,210000,220000,230000,240000,250000,260000,270000,280000,290000,300000,310000,320000,330000,340000,350000,360000,370000,380000,390000,400000,410000,420000,430000,440000,450000,460000,470000,480000,490000,500000,510000,520000,530000,540000,550000,560000,570000,580000,590000,600000,610000,620000,630000,640000,650000,660000,670000,680000,690000,700000,710000,720000,730000,740000,750000,760000,770000,780000,790000,800000,810000,820000,830000,840000,850000,860000,870000,880000,890000,900000,910000,920000,930000,940000,950000,960000,970000,980000,990000,1000000,1010000,1020000,1030000,1040000,1050000,1060000
NW_003726130_1,Count,0,3,0,0,0,0,0,0,0,0,0,0,0,0,0,0,0,0,0,0,0,0,0,0,0,0,0,0,0,0,0,0,0,0,0,0,0,0,0,0,0,0,0,0,0,0,0,0,0,0,0,0,0,0,0,0,0,0,0,0,4,0,0,0,0,0,0,0,0,0,0,0,0,0,0,0,0,0,0,0,0,0,0,0,0,0,0,0,0,1,0,0,0,0,0,0,0,0,0,0,0,1,2,0,1,22,15

	
```

```
		  

		NW_003726132_1, Position,0,10000,20000,30000,40000,50000,60000,70000,80000,90000,100000,110000,120000,130000,140000,150000,160000,170000,180000,190000,200000,210000,220000,230000,240000,250000,260000,270000,280000,290000,300000,310000,320000,330000,340000,350000,360000,370000,380000,390000,400000,410000,420000,430000,440000,450000,460000,470000,480000,490000,500000,510000,520000,530000,540000,550000,560000,570000,580000,590000,600000,610000,620000,630000,640000,650000,660000,670000,680000,690000,700000,710000,720000,730000,740000,750000,760000,770000,780000,790000,800000,810000,820000,830000,840000,850000,860000,870000,880000
NW_003726132_1,Count,0,0,0,0,0,0,0,0,0,0,0,0,0,0,0,0,0,4,0,4,0,7,6,4,0,0,0,0,0,0,0,0,0,0,0,0,0,0,0,0,0,0,0,0,0,0,0,0,0,0,0,0,0,0,0,0,0,0,0,0,0,0,0,0,1,0,0,0,0,0,0,0,0,0,0,0,0,0,0,0,0,0,0,0,0,0,4,7,0

	
```

```
		  

		NW_003726133_1, Position,0,10000,20000,30000,40000,50000,60000,70000,80000,90000,100000,110000,120000,130000,140000,150000,160000,170000,180000,190000,200000,210000,220000,230000,240000,250000,260000,270000,280000,290000,300000,310000,320000,330000,340000,350000,360000,370000,380000,390000,400000,410000,420000,430000,440000,450000,460000,470000,480000,490000,500000,510000,520000,530000,540000,550000,560000,570000,580000,590000,600000,610000,620000,630000,640000,650000,660000,670000,680000,690000,700000,710000,720000,730000,740000,750000,760000,770000,780000,790000,800000,810000,820000
NW_003726133_1,Count,0,0,0,0,51,92,104,147,76,104,110,120,87,118,111,96,98,84,104,133,107,71,8,55,99,20,54,8,36,6,5,0,0,0,0,0,0,74,148,145,31,111,116,132,152,146,132,106,92,106,35,0,0,0,0,43,80,109,97,120,113,132,99,83,86,140,58,123,128,129,148,85,104,82,3,0,1,0,0,0,0,0,0

	
```

```
		  

		NW_003726135_1, Position,0,10000,20000,30000,40000,50000,60000,70000,80000,90000,100000,110000,120000,130000,140000,150000,160000,170000,180000,190000,200000,210000,220000,230000,240000,250000,260000,270000,280000,290000,300000,310000,320000,330000,340000,350000,360000,370000,380000,390000,400000,410000,420000,430000,440000,450000,460000,470000,480000,490000,500000,510000,520000,530000,540000,550000,560000,570000,580000,590000,600000,610000,620000,630000,640000,650000,660000,670000,680000,690000,700000,710000,720000,730000,740000
NW_003726135_1,Count,0,0,0,0,0,0,0,0,0,0,0,0,0,0,0,0,0,0,0,0,0,0,0,0,0,0,0,0,0,0,0,0,0,0,5,16,13,8,5,8,0,0,0,1,0,0,0,0,3,0,0,21,6,6,7,0,4,4,17,2,1,0,0,5,0,6,21,0,9,4,0,0,0,23,30

	
```

```
		  

		NW_003726136_1, Position,0,10000,20000,30000,40000,50000,60000,70000,80000,90000,100000,110000,120000,130000,140000,150000,160000,170000,180000,190000,200000,210000,220000,230000,240000,250000,260000,270000,280000,290000,300000,310000,320000,330000,340000,350000,360000,370000,380000,390000,400000,410000,420000,430000,440000,450000,460000,470000,480000,490000,500000,510000,520000,530000,540000,550000,560000,570000,580000,590000,600000
NW_003726136_1,Count,4,4,2,0,0,2,4,4,1,0,0,1,10,3,10,4,8,5,0,5,0,0,4,5,3,0,9,1,0,6,17,5,3,0,20,2,12,11,5,12,0,5,18,14,9,0,1,5,0,6,7,11,2,2,23,83,81,64,73,87,19

	
```

```
		  

		NW_003726137_1, Position,0,10000,20000,30000,40000,50000,60000,70000,80000,90000,100000,110000,120000,130000,140000,150000,160000,170000,180000,190000,200000,210000,220000,230000,240000,250000,260000,270000,280000,290000,300000,310000,320000,330000,340000,350000,360000,370000,380000,390000,400000,410000,420000,430000,440000,450000,460000,470000,480000,490000,500000,510000,520000,530000,540000,550000,560000,570000
NW_003726137_1,Count,93,120,68,77,104,85,100,137,103,70,47,42,61,61,58,67,65,73,105,67,79,102,94,84,113,106,64,86,108,95,98,89,87,119,90,108,104,126,119,129,102,135,74,91,69,127,108,95,133,103,117,112,113,89,5,76,43,30

	
```

```
		  

		NW_003726139_1, Position,0,10000,20000,30000,40000,50000,60000,70000,80000,90000,100000,110000,120000,130000,140000,150000,160000,170000,180000,190000,200000,210000,220000,230000,240000,250000,260000,270000,280000,290000,300000,310000,320000,330000,340000,350000,360000,370000,380000,390000,400000,410000,420000,430000,440000,450000,460000,470000,480000,490000,500000,510000,520000,530000,540000,550000
NW_003726139_1,Count,0,0,0,1,0,0,0,3,0,0,3,0,0,0,0,0,0,0,0,3,0,0,0,0,0,0,0,12,28,0,0,0,0,0,0,0,0,0,3,0,0,5,0,0,0,0,0,0,0,0,7,3,11,18,15,20

	
```

```
		  

		NW_003726140_1, Position,0,10000,20000,30000,40000,50000,60000,70000,80000,90000,100000,110000,120000,130000,140000,150000,160000,170000,180000,190000,200000,210000,220000,230000,240000,250000,260000,270000,280000,290000,300000,310000,320000,330000,340000,350000,360000,370000,380000,390000,400000,410000,420000,430000,440000,450000,460000,470000,480000,490000,500000,510000
NW_003726140_1,Count,0,0,0,0,0,0,0,0,0,0,0,0,0,0,0,0,0,0,0,0,0,0,0,0,0,0,0,0,0,0,0,0,0,0,0,0,0,2,0,0,0,0,0,0,0,0,0,0,0,0,0,0

	
```

```
		  

		NW_003726141_1, Position,0,10000,20000,30000,40000,50000,60000,70000,80000,90000,100000,110000,120000,130000,140000,150000,160000,170000,180000,190000,200000,210000,220000,230000,240000,250000,260000,270000,280000,290000,300000,310000,320000,330000,340000,350000,360000,370000,380000,390000,400000,410000,420000,430000,440000,450000,460000,470000,480000,490000
NW_003726141_1,Count,35,0,0,0,0,0,1,7,0,0,0,0,0,5,14,15,4,0,0,0,0,0,0,7,0,0,2,0,0,0,0,0,1,4,2,0,0,11,14,6,1,2,0,1,0,0,0,0,0,0

	
```

```
		  

		NW_003726142_1, Position,0,10000,20000,30000,40000,50000,60000,70000,80000,90000,100000,110000,120000,130000,140000,150000,160000,170000,180000,190000,200000,210000,220000,230000,240000,250000,260000,270000,280000,290000,300000,310000,320000,330000,340000,350000,360000,370000,380000,390000,400000,410000,420000,430000,440000,450000,460000,470000,480000,490000
NW_003726142_1,Count,6,1,0,0,0,0,0,0,0,0,2,6,2,3,15,1,3,4,1,0,0,7,2,6,3,1,0,0,0,0,0,0,0,0,0,0,2,2,0,0,0,0,0,0,0,0,1,0,0,0

	
```

```
		  

		NW_003726143_1, Position,0,10000,20000,30000,40000,50000,60000,70000,80000,90000,100000,110000,120000,130000,140000,150000,160000,170000,180000,190000,200000,210000,220000,230000,240000,250000,260000,270000,280000,290000,300000,310000,320000,330000,340000,350000,360000,370000,380000,390000,400000,410000,420000,430000,440000,450000,460000,470000,480000,490000,500000,510000,520000,530000,540000,550000,560000,570000
NW_003726143_1,Count,93,62,65,76,23,0,0,0,0,0,0,64,88,76,62,66,79,4,0,3,67,46,54,68,39,6,0,0,0,0,0,0,0,0,0,0,0,0,0,0,0,0,0,0,1,8,0,0,0,0,0,0,0,0,0,0,0,3

	
```

```
		  

		NW_003726144_1, Position,0,10000,20000,30000,40000,50000,60000,70000,80000,90000,100000,110000,120000,130000,140000,150000,160000,170000,180000,190000,200000,210000,220000,230000,240000,250000,260000,270000,280000,290000,300000,310000,320000,330000,340000,350000,360000,370000,380000,390000,400000,410000,420000,430000,440000,450000,460000,470000,480000,490000,500000,510000,520000,530000,540000,550000,560000
NW_003726144_1,Count,18,1,3,3,6,1,4,0,0,0,4,4,11,2,3,11,3,0,0,0,3,0,6,3,22,4,0,6,1,0,0,0,0,11,11,6,7,0,0,1,9,1,0,0,0,0,0,0,0,0,0,2,4,16,1,15,1

	
```

```
		  

		NW_003726145_1, Position,0,10000,20000,30000,40000,50000,60000,70000,80000,90000,100000,110000,120000,130000,140000,150000,160000,170000,180000,190000,200000,210000,220000,230000,240000,250000,260000,270000,280000,290000,300000,310000,320000,330000,340000,350000,360000,370000,380000,390000,400000
NW_003726145_1,Count,10,0,0,0,0,0,0,0,0,0,0,0,0,0,0,0,0,0,0,0,0,0,0,0,0,0,0,0,0,0,0,0,0,0,0,0,0,0,0,0,0

	
```

```
		  

		NW_003726147_1, Position,0,10000,20000,30000,40000,50000,60000,70000,80000,90000,100000,110000,120000,130000,140000,150000,160000,170000,180000,190000,200000,210000,220000,230000,240000,250000,260000,270000,280000,290000,300000,310000,320000,330000,340000,350000,360000,370000,380000,390000,400000,410000,420000,430000,440000,450000,460000,470000,480000,490000,500000
NW_003726147_1,Count,4,0,0,0,0,0,0,0,0,0,3,9,0,2,0,4,0,0,0,0,0,0,0,1,0,0,0,0,0,0,2,0,0,0,0,0,0,0,0,0,0,0,0,0,0,0,0,3,0,0,0

	
```

```
		  

		NW_003726148_1, Position,0,10000,20000,30000,40000,50000,60000,70000,80000,90000,100000,110000,120000,130000,140000,150000,160000,170000,180000,190000,200000,210000,220000,230000,240000,250000,260000,270000,280000,290000,300000,310000,320000,330000,340000,350000,360000,370000,380000,390000,400000,410000,420000,430000,440000,450000,460000,470000,480000,490000,500000,510000,520000,530000,540000
NW_003726148_1,Count,5,0,0,0,0,0,0,0,0,0,0,0,0,0,0,0,0,0,0,0,28,0,0,0,0,0,0,0,0,0,0,0,0,0,0,0,0,0,0,0,6,3,0,0,0,0,0,0,0,5,0,3,0,11,0

	
```

```
		  

		NW_003726149_1, Position,0,10000,20000,30000,40000,50000,60000,70000,80000,90000,100000,110000,120000,130000,140000,150000,160000,170000,180000,190000,200000,210000,220000,230000,240000,250000,260000,270000,280000,290000,300000,310000,320000,330000,340000,350000,360000,370000,380000,390000,400000,410000,420000,430000,440000
NW_003726149_1,Count,56,74,23,57,59,43,35,39,68,24,20,24,28,77,69,53,0,0,0,0,0,0,41,20,35,22,16,5,1,65,78,45,0,0,23,31,44,59,63,66,42,45,79,63,25

	
```

```
		  

		NW_003726150_1, Position,0,10000,20000,30000,40000,50000,60000,70000,80000,90000,100000,110000,120000,130000,140000,150000,160000,170000,180000,190000,200000,210000,220000,230000,240000,250000,260000,270000,280000,290000,300000,310000,320000,330000,340000,350000,360000,370000
NW_003726150_1,Count,0,0,0,0,3,2,0,0,3,0,0,0,0,0,0,0,0,0,0,0,0,0,0,0,0,0,0,0,0,0,0,4,0,0,0,0,0,0

	
```

```
		  

		NW_003726151_1, Position,0,10000,20000,30000,40000,50000,60000,70000,80000,90000,100000,110000,120000,130000,140000,150000,160000,170000,180000,190000,200000,210000,220000,230000,240000,250000,260000,270000,280000,290000,300000,310000,320000,330000,340000,350000,360000,370000,380000
NW_003726151_1,Count,15,4,3,9,2,0,0,0,0,0,0,0,0,0,1,0,0,1,0,0,0,0,0,2,5,7,17,5,0,0,3,0,3,4,13,11,0,0,1

	
```

```
		  

		NW_003726153_1, Position,0,10000,20000,30000,40000,50000,60000,70000,80000,90000,100000,110000,120000,130000,140000,150000,160000,170000,180000,190000,200000,210000,220000,230000,240000,250000,260000,270000,280000,290000,300000,310000,320000,330000,340000,350000,360000,370000,380000,390000
NW_003726153_1,Count,20,28,8,7,0,0,0,0,0,0,0,0,0,0,0,0,0,0,4,0,0,0,0,0,0,0,0,0,0,1,0,2,1,2,13,7,5,0,0,0

	
```

```
		  

		NW_003726156_1, Position,0,10000,20000,30000,40000,50000,60000,70000,80000,90000,100000,110000,120000,130000,140000,150000,160000,170000,180000,190000,200000,210000,220000,230000,240000,250000,260000,270000,280000,290000,300000,310000,320000,330000,340000,350000,360000
NW_003726156_1,Count,82,6,8,11,2,0,13,0,1,8,10,0,3,1,16,3,19,1,0,12,10,12,8,0,3,0,0,1,0,0,0,8,6,3,8,3,12

	
```

```
		  

		NW_003726159_1, Position,0,10000,20000,30000,40000,50000,60000,70000,80000,90000,100000,110000,120000,130000,140000,150000,160000,170000,180000,190000,200000,210000,220000,230000,240000,250000,260000,270000,280000,290000,300000,310000
NW_003726159_1,Count,0,1,0,1,0,0,0,0,0,0,0,2,0,0,0,0,0,0,0,0,0,2,0,0,0,0,2,3,7,7,0,0

	
```

```
		  

		NW_003726160_1, Position,0,10000,20000,30000,40000,50000,60000,70000,80000,90000,100000,110000,120000,130000,140000,150000,160000,170000,180000,190000,200000,210000,220000,230000,240000,250000,260000,270000,280000,290000,300000,310000,320000,330000,340000,350000,360000,370000,380000,390000,400000,410000,420000,430000
NW_003726160_1,Count,2,0,0,0,0,0,0,0,0,0,0,0,1,0,2,0,0,0,0,0,0,0,0,0,0,0,0,0,0,0,0,0,0,0,0,0,0,0,0,0,0,0,0,0

	
```

```
		  

		NW_003726161_1, Position,0,1000,2000,3000,4000,5000,6000,7000,8000,9000,10000,11000,12000,13000,14000,15000,16000,17000,18000,19000,20000,21000,22000,23000,24000,25000,26000,27000,28000,29000,30000,31000,32000,33000,34000,35000,36000,37000,38000,39000,40000,41000,42000,43000,44000,45000,46000,47000,48000,49000,50000,51000,52000,53000,54000,55000,56000,57000,58000,59000,60000,61000,62000,63000,64000,65000,66000,67000,68000,69000,70000,71000,72000,73000,74000,75000,76000,77000,78000,79000,80000,81000,82000,83000,84000,85000,86000,87000,88000,89000,90000,91000,92000,93000,94000,95000,96000,97000,98000,99000,100000,101000,102000,103000,104000,105000,106000,107000,108000,109000,110000,111000,112000,113000,114000,115000,116000,117000,118000,119000,120000,121000,122000,123000,124000,125000,126000,127000,128000,129000,130000,131000,132000,133000,134000,135000,136000,137000,138000,139000,140000,141000,142000,143000,144000,145000,146000,147000,148000,149000,150000,151000,152000,153000,154000,155000,156000,157000,158000,159000,160000,161000,162000,163000,164000,165000,166000,167000,168000,169000,170000,171000,172000,173000,174000,175000,176000,177000,178000,179000,180000,181000,182000,183000,184000,185000,186000,187000,188000,189000,190000,191000,192000,193000,194000,195000,196000,197000,198000,199000,200000,201000,202000,203000,204000,205000,206000,207000,208000,209000,210000,211000,212000,213000,214000,215000,216000,217000,218000,219000,220000,221000,222000,223000,224000,225000,226000,227000,228000,229000,230000,231000,232000,233000,234000,235000,236000,237000,238000,239000,240000,241000,242000,243000,244000,245000,246000,247000,248000,249000,250000,251000,252000,253000,254000,255000,256000,257000,258000,259000,260000,261000,262000,263000,264000,265000,266000,267000,268000,269000,270000,271000,272000,273000
NW_003726161_1,Count,0,0,0,0,0,0,0,0,0,0,0,0,0,0,0,0,0,0,0,0,0,0,0,0,0,0,0,0,0,0,0,0,0,0,0,0,0,0,0,0,0,0,0,0,0,0,0,0,0,0,0,0,0,0,0,0,0,0,0,0,0,0,0,0,0,0,0,0,0,0,0,0,0,0,0,0,0,0,0,0,0,0,0,0,0,0,0,0,0,0,0,0,0,0,0,0,0,0,0,0,0,0,0,0,0,0,0,0,0,0,0,0,0,0,0,0,0,0,0,0,0,0,0,0,0,0,0,0,0,0,0,0,0,0,0,0,0,0,0,0,0,0,0,0,0,0,0,0,0,0,0,0,0,0,0,0,0,0,0,0,0,0,0,0,0,0,0,0,0,0,0,0,0,0,0,0,0,0,0,0,0,0,0,0,0,0,0,0,0,0,0,0,0,0,0,0,0,0,0,0,0,0,0,0,0,0,0,0,0,0,0,0,0,0,0,0,0,0,0,0,0,0,0,0,0,0,0,0,0,0,0,0,0,0,0,0,0,0,0,0,0,0,0,0,0,0,0,0,0,0,0,0,0,0,0,0,0,0,0,0,3,0,0,0,0,0,0,0,0,0,0,0,0,0

	
```

```
		  

		NW_003726162_1, Position,0,1000,2000,3000,4000,5000,6000,7000,8000,9000,10000,11000,12000,13000,14000,15000,16000,17000,18000,19000,20000,21000,22000,23000,24000,25000,26000,27000,28000,29000,30000,31000,32000,33000,34000,35000,36000,37000,38000,39000,40000,41000,42000,43000,44000,45000,46000,47000,48000,49000,50000,51000,52000,53000,54000,55000,56000,57000,58000,59000,60000,61000,62000,63000,64000,65000,66000,67000,68000,69000,70000,71000,72000,73000,74000,75000,76000,77000,78000,79000,80000,81000,82000,83000,84000,85000,86000,87000,88000,89000,90000,91000,92000,93000,94000,95000,96000,97000,98000,99000,100000,101000,102000,103000,104000,105000,106000,107000,108000,109000,110000,111000,112000,113000,114000,115000,116000,117000,118000,119000,120000,121000,122000,123000,124000,125000,126000,127000,128000,129000,130000,131000,132000,133000,134000,135000,136000,137000,138000,139000,140000,141000,142000,143000,144000,145000,146000,147000,148000,149000,150000,151000,152000,153000,154000,155000,156000,157000,158000,159000,160000,161000,162000,163000,164000,165000,166000,167000,168000,169000,170000,171000,172000,173000,174000,175000,176000,177000,178000,179000,180000,181000,182000,183000,184000,185000,186000,187000,188000,189000,190000,191000,192000,193000,194000,195000,196000,197000,198000,199000,200000,201000,202000,203000,204000,205000,206000,207000,208000,209000,210000,211000,212000,213000,214000,215000,216000,217000,218000,219000,220000,221000,222000,223000,224000,225000,226000,227000,228000,229000,230000,231000,232000,233000,234000,235000,236000,237000,238000,239000,240000,241000,242000,243000,244000,245000,246000,247000,248000,249000,250000,251000,252000,253000,254000,255000,256000,257000,258000,259000,260000,261000,262000,263000,264000,265000,266000,267000,268000,269000,270000,271000,272000,273000,274000,275000,276000,277000,278000,279000,280000,281000
NW_003726162_1,Count,0,0,0,0,0,0,0,0,0,0,0,0,0,0,0,0,0,0,0,0,0,0,0,0,0,0,0,0,0,0,0,0,0,0,0,0,0,0,0,0,0,0,0,0,0,0,0,0,0,0,0,0,0,0,0,0,0,0,0,0,2,0,0,0,0,0,0,0,0,0,0,0,0,0,0,0,0,0,0,0,0,0,0,0,0,0,0,0,0,0,0,0,0,0,0,0,0,0,0,0,0,0,0,0,0,0,0,0,0,0,0,0,0,0,0,0,0,0,2,0,0,0,0,0,0,0,0,0,0,0,0,0,0,0,0,0,0,0,0,0,0,0,0,0,0,0,0,0,0,0,0,0,0,0,0,0,0,0,0,0,0,0,0,0,0,0,0,0,0,0,0,0,0,0,0,0,0,0,0,0,1,3,0,0,0,0,0,0,0,0,0,0,0,0,0,0,0,0,0,0,0,0,0,0,0,0,0,0,0,0,0,0,0,0,0,0,0,0,0,0,0,0,0,0,0,0,0,0,0,0,0,0,0,0,0,0,0,0,0,0,0,0,0,0,0,0,0,0,0,0,0,0,0,0,0,0,0,0,0,0,0,0,0,0,0,0,0,0,0,0,0,0,0,0,0,0,0,0,0,0,0,0

	
```

```
		  

		NW_003726167_1, Position,0,1000,2000,3000,4000,5000,6000,7000,8000,9000,10000,11000,12000,13000,14000,15000,16000,17000,18000,19000,20000,21000,22000,23000,24000,25000,26000,27000,28000,29000,30000,31000,32000,33000,34000,35000,36000,37000,38000,39000,40000,41000,42000,43000,44000,45000,46000,47000,48000,49000,50000,51000,52000,53000,54000,55000,56000,57000,58000,59000,60000,61000,62000,63000,64000,65000,66000,67000,68000,69000,70000,71000,72000,73000,74000,75000,76000,77000,78000,79000,80000,81000,82000,83000,84000,85000,86000,87000,88000,89000,90000,91000,92000,93000,94000,95000,96000,97000,98000,99000,100000,101000,102000,103000,104000,105000,106000,107000,108000,109000,110000,111000,112000,113000,114000,115000,116000,117000,118000,119000,120000,121000,122000,123000,124000,125000,126000,127000,128000,129000,130000,131000,132000,133000,134000,135000,136000,137000,138000,139000,140000,141000,142000,143000,144000,145000,146000,147000,148000,149000,150000,151000,152000,153000,154000,155000,156000,157000,158000,159000,160000,161000,162000,163000,164000,165000,166000,167000,168000,169000,170000,171000,172000,173000,174000,175000,176000,177000,178000,179000,180000,181000,182000,183000,184000,185000,186000,187000,188000,189000,190000,191000,192000,193000,194000,195000,196000,197000,198000,199000,200000,201000,202000,203000,204000,205000,206000,207000,208000,209000,210000,211000,212000,213000,214000,215000,216000,217000,218000,219000,220000,221000,222000,223000,224000,225000,226000,227000,228000,229000,230000,231000,232000,233000,234000,235000,236000,237000,238000,239000,240000,241000,242000,243000,244000,245000,246000,247000,248000,249000,250000,251000,252000
NW_003726167_1,Count,0,0,6,3,2,7,0,10,0,2,0,2,3,0,0,0,0,1,0,0,0,0,0,0,2,3,4,0,1,0,0,0,1,5,0,1,0,2,1,0,1,4,2,1,7,0,3,0,0,3,0,9,4,3,0,0,0,0,2,0,1,0,0,0,0,0,0,0,0,0,0,0,0,0,0,0,0,0,7,1,0,0,0,0,0,0,0,0,0,0,0,0,0,0,0,0,0,0,0,0,0,0,0,0,0,0,0,0,0,0,0,0,0,1,0,0,0,0,0,0,0,0,0,0,0,0,3,0,0,0,0,0,0,0,0,0,0,4,0,0,0,1,0,0,0,0,0,0,0,0,0,0,0,0,0,0,0,0,0,0,0,0,0,0,0,0,0,0,0,0,0,0,0,0,0,0,0,0,0,0,0,0,0,0,0,0,0,0,0,0,0,0,0,0,0,0,0,0,0,0,0,0,0,0,0,0,0,3,0,0,2,0,0,0,0,1,0,0,0,0,0,0,0,0,0,0,0,6,3,0,7,0,1,0,3,0,0,0,2,2,2,0,0,0,2,0,0,0,0,0,0,0,1

	
```

```
		  

		NW_003726168_1, Position,0,1000,2000,3000,4000,5000,6000,7000,8000,9000,10000,11000,12000,13000,14000,15000,16000,17000,18000,19000,20000,21000,22000,23000,24000,25000,26000,27000,28000,29000,30000,31000,32000,33000,34000,35000,36000,37000,38000,39000,40000,41000,42000,43000,44000,45000,46000,47000,48000,49000,50000,51000,52000,53000,54000,55000,56000,57000,58000,59000,60000,61000,62000,63000,64000,65000,66000,67000,68000,69000,70000,71000,72000,73000,74000,75000,76000,77000,78000,79000,80000,81000,82000,83000,84000,85000,86000,87000,88000,89000,90000,91000,92000,93000,94000,95000,96000,97000,98000,99000,100000,101000,102000,103000,104000,105000,106000,107000,108000,109000,110000,111000,112000,113000,114000,115000,116000,117000,118000,119000,120000,121000,122000,123000,124000,125000,126000,127000,128000,129000,130000,131000,132000,133000,134000,135000,136000,137000,138000,139000,140000,141000,142000,143000,144000,145000,146000,147000,148000,149000,150000,151000,152000,153000,154000,155000,156000,157000,158000,159000,160000,161000,162000,163000,164000,165000,166000,167000,168000,169000,170000,171000,172000,173000,174000,175000,176000,177000,178000,179000,180000,181000,182000,183000,184000,185000,186000,187000,188000,189000,190000,191000,192000,193000,194000,195000,196000,197000,198000,199000,200000,201000,202000,203000,204000,205000,206000,207000,208000,209000,210000,211000,212000,213000,214000,215000,216000,217000,218000,219000,220000,221000,222000,223000,224000,225000,226000,227000,228000,229000,230000,231000,232000,233000,234000,235000,236000,237000,238000,239000,240000,241000,242000,243000,244000,245000,246000,247000,248000,249000,250000,251000,252000,253000,254000
NW_003726168_1,Count,0,0,0,0,0,0,0,0,2,0,3,0,3,0,0,0,0,0,0,0,0,0,0,3,0,0,0,2,1,0,0,0,0,0,0,0,0,0,0,0,0,0,0,0,0,0,0,0,0,0,0,0,0,0,0,0,0,0,0,0,0,0,0,0,0,0,0,0,0,0,0,0,0,0,0,0,0,0,0,0,3,0,0,0,0,0,0,0,0,0,0,0,0,0,0,0,0,0,14,9,0,13,9,14,2,4,0,3,4,5,1,1,8,2,5,0,2,0,0,0,1,5,11,0,1,0,2,0,0,0,0,0,0,0,0,9,3,0,0,0,0,7,8,2,3,0,0,3,4,0,0,0,6,3,4,5,2,0,0,0,1,0,0,0,0,0,4,0,0,0,4,0,0,0,0,0,0,0,0,0,0,0,0,0,0,0,0,0,0,0,0,0,0,3,3,0,0,0,0,0,0,0,0,5,2,0,0,0,0,1,4,1,2,9,0,0,0,0,0,0,0,0,0,0,0,10,0,0,3,0,0,0,0,0,0,5,6,10,3,0,0,2,4,5,0,5,9,10,4,5,10,1,8,12,2

	
```

```
		  

		NW_003726170_1, Position,0,1000,2000,3000,4000,5000,6000,7000,8000,9000,10000,11000,12000,13000,14000,15000,16000,17000,18000,19000,20000,21000,22000,23000,24000,25000,26000,27000,28000,29000,30000,31000,32000,33000,34000,35000,36000,37000,38000,39000,40000,41000,42000,43000,44000,45000,46000,47000,48000,49000,50000,51000,52000,53000,54000,55000,56000,57000,58000,59000,60000,61000,62000,63000,64000,65000,66000,67000,68000,69000,70000,71000,72000,73000,74000,75000,76000,77000,78000,79000,80000,81000,82000,83000,84000,85000,86000,87000,88000,89000,90000,91000,92000,93000,94000,95000,96000,97000,98000,99000,100000,101000,102000,103000,104000,105000,106000,107000,108000,109000,110000,111000,112000,113000,114000,115000,116000,117000,118000,119000,120000,121000,122000,123000,124000,125000,126000,127000,128000,129000,130000,131000,132000,133000,134000,135000,136000,137000,138000,139000,140000,141000,142000,143000,144000,145000,146000,147000,148000,149000,150000,151000,152000,153000,154000,155000,156000,157000,158000,159000,160000,161000,162000,163000,164000,165000,166000,167000,168000,169000,170000,171000,172000,173000,174000,175000,176000,177000,178000,179000,180000,181000,182000,183000,184000,185000,186000,187000,188000,189000,190000,191000,192000,193000,194000,195000,196000,197000,198000,199000,200000,201000,202000,203000,204000,205000,206000,207000,208000,209000,210000,211000,212000,213000,214000,215000,216000,217000,218000,219000,220000,221000,222000,223000
NW_003726170_1,Count,0,0,0,0,0,0,0,0,0,0,0,0,0,0,0,0,0,0,0,0,0,0,0,0,0,0,0,0,0,0,0,0,0,0,0,0,0,0,0,0,0,0,0,0,0,0,0,0,0,0,0,0,0,0,0,0,0,0,0,0,0,0,0,0,0,0,0,0,0,0,0,0,0,0,0,0,0,0,0,0,0,0,0,0,0,0,0,0,0,0,0,0,0,0,0,0,0,0,0,0,0,0,0,0,0,0,0,0,0,0,0,0,0,0,0,0,0,0,0,0,0,0,0,0,0,0,0,0,0,0,0,0,0,0,0,0,0,0,0,0,0,0,0,0,0,0,0,0,0,0,0,3,0,0,0,0,0,0,0,0,0,0,0,0,0,0,0,0,0,0,0,0,0,0,0,0,0,0,0,0,0,0,0,0,0,0,0,0,0,0,0,0,0,0,0,0,0,0,0,0,0,0,0,0,0,0,0,0,0,0,0,0,0,0,0,0,0,0,0,0,0,0,0,0

	
```

```
		  

		NW_003726174_1, Position,0,10000,20000,30000,40000,50000,60000,70000,80000,90000,100000,110000,120000,130000,140000,150000,160000,170000,180000,190000,200000,210000,220000,230000,240000,250000,260000,270000,280000,290000,300000,310000,320000,330000,340000,350000,360000,370000,380000
NW_003726174_1,Count,87,59,33,0,0,0,0,0,0,0,0,0,0,0,0,0,0,25,59,57,57,75,56,96,79,94,77,49,24,54,78,54,72,39,3,63,73,62,66

	
```

```
		  

		NW_003726177_1, Position,0,1000,2000,3000,4000,5000,6000,7000,8000,9000,10000,11000,12000,13000,14000,15000,16000,17000,18000,19000,20000,21000,22000,23000,24000,25000,26000,27000,28000,29000,30000,31000,32000,33000,34000,35000,36000,37000,38000,39000,40000,41000,42000,43000,44000,45000,46000,47000,48000,49000,50000,51000,52000,53000,54000,55000,56000,57000,58000,59000,60000,61000,62000,63000,64000,65000,66000,67000,68000,69000,70000,71000,72000,73000,74000,75000,76000,77000,78000,79000,80000,81000,82000,83000,84000,85000,86000,87000,88000,89000,90000,91000,92000,93000,94000,95000,96000,97000,98000,99000,100000,101000,102000,103000,104000,105000,106000,107000,108000,109000,110000,111000,112000,113000,114000,115000,116000,117000,118000,119000,120000,121000,122000,123000,124000,125000,126000,127000,128000,129000,130000,131000,132000,133000,134000,135000,136000,137000,138000,139000,140000,141000,142000,143000,144000,145000,146000,147000,148000,149000,150000,151000,152000,153000,154000,155000,156000,157000,158000,159000,160000,161000,162000,163000,164000,165000,166000,167000,168000,169000,170000,171000,172000,173000,174000,175000,176000,177000,178000,179000,180000,181000,182000,183000,184000,185000,186000,187000,188000,189000,190000,191000,192000,193000,194000,195000,196000,197000,198000,199000,200000,201000,202000,203000,204000,205000,206000,207000,208000,209000,210000,211000,212000,213000,214000,215000,216000
NW_003726177_1,Count,0,0,1,1,0,5,3,0,0,0,0,0,0,4,0,0,0,0,0,0,0,0,0,0,0,0,0,0,0,0,0,0,0,1,0,0,0,0,0,3,0,0,0,0,0,0,0,0,0,0,0,0,0,0,0,0,0,0,0,0,0,0,0,0,0,0,0,0,0,0,0,0,0,0,0,0,0,0,0,0,0,0,0,0,0,0,0,0,0,0,0,3,0,0,0,0,0,0,0,0,0,0,0,0,0,0,0,0,0,0,0,0,0,0,0,0,0,0,3,8,0,0,1,0,0,0,0,0,0,0,0,0,0,0,0,0,0,1,0,0,2,0,0,0,0,0,0,2,0,0,0,0,0,0,0,0,0,0,0,0,0,0,0,0,0,0,0,0,0,0,0,2,0,0,0,0,0,0,0,0,0,0,0,0,0,0,0,0,0,0,0,0,0,0,0,0,0,0,0,0,0,0,0,0,0,0,0,0,0,0,0,0,0,0,0,0,0

	
```

```
		  

		NW_003726180_1, Position,0,1000,2000,3000,4000,5000,6000,7000,8000,9000,10000,11000,12000,13000,14000,15000,16000,17000,18000,19000,20000,21000,22000,23000,24000,25000,26000,27000,28000,29000,30000,31000,32000,33000,34000,35000,36000,37000,38000,39000,40000,41000,42000,43000,44000,45000,46000,47000,48000,49000,50000,51000,52000,53000,54000,55000,56000,57000,58000,59000,60000,61000,62000,63000,64000,65000,66000,67000,68000,69000,70000,71000,72000,73000,74000,75000,76000,77000,78000,79000,80000,81000,82000,83000,84000,85000,86000,87000,88000,89000,90000,91000,92000,93000,94000,95000,96000,97000,98000,99000,100000,101000,102000,103000,104000,105000,106000,107000,108000,109000,110000,111000,112000,113000,114000,115000,116000,117000,118000,119000,120000,121000,122000,123000,124000,125000,126000,127000,128000,129000,130000,131000,132000,133000,134000,135000,136000,137000,138000,139000,140000,141000,142000,143000,144000,145000,146000,147000,148000,149000,150000,151000,152000,153000,154000,155000,156000,157000,158000,159000,160000,161000,162000,163000,164000,165000,166000,167000,168000,169000,170000,171000,172000,173000,174000,175000,176000,177000,178000,179000,180000,181000,182000,183000,184000,185000,186000,187000,188000,189000,190000,191000,192000,193000,194000,195000,196000,197000,198000,199000,200000
NW_003726180_1,Count,0,0,0,0,0,3,1,0,0,0,0,0,0,0,0,0,0,0,1,0,0,0,0,0,0,0,5,1,6,4,0,0,0,2,1,4,0,2,0,3,0,0,3,0,0,0,4,0,2,0,0,5,1,2,0,0,4,0,0,0,0,0,0,0,0,0,0,0,0,0,0,0,0,0,0,0,0,0,0,0,0,0,0,0,0,3,0,3,0,0,0,0,0,1,0,0,0,0,0,0,0,0,0,0,0,0,0,0,0,1,0,0,0,0,0,0,0,0,0,0,0,0,0,0,0,0,0,0,0,0,0,0,0,0,0,0,0,0,0,0,0,0,0,0,0,0,4,2,4,6,0,0,1,5,0,0,3,0,2,0,0,1,0,0,0,2,2,3,0,0,0,0,0,0,0,0,1,2,0,0,0,0,0,0,0,6,6,4,4,0,0,0,0,0,0,8,6,0,2,0,0

	
```

```
		  

		NW_003726183_1, Position,0,1000,2000,3000,4000,5000,6000,7000,8000,9000,10000,11000,12000,13000,14000,15000,16000,17000,18000,19000,20000,21000,22000,23000,24000,25000,26000,27000,28000,29000,30000,31000,32000,33000,34000,35000,36000,37000,38000,39000,40000,41000,42000,43000,44000,45000,46000,47000,48000,49000,50000,51000,52000,53000,54000,55000,56000,57000,58000,59000,60000,61000,62000,63000,64000,65000,66000,67000,68000,69000,70000,71000,72000,73000,74000,75000,76000,77000,78000,79000,80000,81000,82000,83000,84000,85000,86000,87000,88000,89000,90000,91000,92000,93000,94000,95000,96000,97000,98000,99000,100000,101000,102000,103000,104000,105000,106000,107000,108000,109000,110000,111000,112000,113000,114000,115000,116000,117000,118000,119000,120000,121000,122000,123000,124000,125000,126000,127000,128000,129000,130000,131000,132000,133000,134000,135000,136000,137000,138000,139000,140000,141000,142000,143000,144000,145000,146000,147000,148000,149000,150000,151000,152000,153000,154000,155000,156000,157000,158000,159000,160000,161000,162000,163000,164000,165000,166000,167000,168000,169000,170000,171000,172000,173000,174000,175000,176000,177000,178000,179000,180000,181000,182000,183000,184000,185000,186000,187000,188000,189000,190000,191000,192000
NW_003726183_1,Count,0,0,0,0,0,0,0,0,0,0,0,0,0,0,2,0,0,0,4,0,0,0,0,4,4,0,0,7,0,1,1,0,0,0,0,2,4,1,0,0,1,0,0,0,0,0,0,1,0,0,0,0,0,2,0,0,0,0,0,0,0,0,0,0,0,0,0,2,0,0,0,1,0,0,0,0,0,0,0,0,0,0,0,0,0,0,0,0,0,0,0,1,0,0,5,0,0,0,0,0,0,1,0,0,1,3,4,1,0,4,3,0,0,0,1,3,1,0,0,0,0,0,4,0,0,0,1,1,1,0,9,3,0,7,2,8,10,6,1,1,0,9,0,4,9,6,6,10,0,0,0,0,0,0,0,6,0,1,0,4,5,0,0,5,0,0,2,9,1,0,0,6,5,10,0,7,14,7,2,4,7,8,1,12,2,4,0,5,2,6,3,0,1

	
```

```
		  

		NW_003726184_1, Position,0,1000,2000,3000,4000,5000,6000,7000,8000,9000,10000,11000,12000,13000,14000,15000,16000,17000,18000,19000,20000,21000,22000,23000,24000,25000,26000,27000,28000,29000,30000,31000,32000,33000,34000,35000,36000,37000,38000,39000,40000,41000,42000,43000,44000,45000,46000,47000,48000,49000,50000,51000,52000,53000,54000,55000,56000,57000,58000,59000,60000,61000,62000,63000,64000,65000,66000,67000,68000,69000,70000,71000,72000,73000,74000,75000,76000,77000,78000,79000,80000,81000,82000,83000,84000,85000,86000,87000,88000,89000,90000,91000,92000,93000,94000,95000,96000,97000,98000,99000,100000,101000,102000,103000,104000,105000,106000,107000,108000,109000,110000,111000,112000,113000,114000,115000,116000,117000,118000,119000,120000,121000,122000,123000,124000,125000,126000,127000,128000,129000,130000,131000,132000,133000,134000,135000,136000,137000,138000,139000,140000,141000,142000,143000,144000,145000,146000,147000,148000,149000,150000,151000,152000,153000,154000,155000,156000,157000,158000,159000,160000,161000,162000,163000,164000,165000,166000,167000,168000,169000,170000,171000,172000,173000,174000,175000,176000,177000,178000,179000,180000,181000,182000,183000,184000,185000,186000,187000,188000,189000,190000,191000,192000,193000,194000,195000,196000,197000,198000,199000,200000,201000,202000,203000,204000,205000,206000,207000,208000,209000,210000
NW_003726184_1,Count,0,0,0,0,0,0,0,0,0,0,0,0,0,0,0,0,0,0,0,0,0,0,0,0,0,0,0,0,0,0,0,0,0,0,0,0,0,0,0,0,0,0,0,0,0,0,0,0,0,0,0,0,0,0,0,0,0,0,0,0,0,0,0,0,0,0,0,0,0,0,0,0,0,0,0,0,0,0,0,0,0,0,0,0,0,0,0,0,0,0,0,0,0,0,0,0,0,0,0,0,0,0,0,0,0,0,0,0,0,0,0,0,0,0,0,0,0,0,0,0,0,0,0,0,0,0,0,0,0,0,0,0,0,0,0,0,0,0,0,0,0,0,0,0,0,0,0,0,0,0,0,0,0,0,0,0,0,0,0,0,0,0,0,0,0,0,0,0,0,0,0,0,0,4,0,0,0,0,0,0,0,0,0,0,0,0,0,0,0,0,0,0,0,0,0,0,0,0,0,0,0,0,0,0,0,7,0,0,0,0,0

	
```

```
		  

		NW_003726185_1, Position,0,1000,2000,3000,4000,5000,6000,7000,8000,9000,10000,11000,12000,13000,14000,15000,16000,17000,18000,19000,20000,21000,22000,23000,24000,25000,26000,27000,28000,29000,30000,31000,32000,33000,34000,35000,36000,37000,38000,39000,40000,41000,42000,43000,44000,45000,46000,47000,48000,49000,50000,51000,52000,53000,54000,55000,56000,57000,58000,59000,60000,61000,62000,63000,64000,65000,66000,67000,68000,69000,70000,71000,72000,73000,74000,75000,76000,77000,78000,79000,80000,81000,82000,83000,84000,85000,86000,87000,88000,89000,90000,91000,92000,93000,94000,95000,96000,97000,98000,99000,100000,101000,102000,103000,104000,105000,106000,107000,108000,109000,110000,111000,112000,113000,114000,115000,116000,117000,118000,119000,120000,121000,122000,123000,124000,125000,126000,127000,128000,129000,130000,131000,132000,133000,134000,135000,136000,137000,138000,139000,140000,141000,142000,143000,144000,145000,146000,147000,148000,149000,150000,151000,152000,153000,154000,155000,156000,157000,158000,159000,160000,161000,162000,163000,164000,165000,166000,167000,168000,169000,170000,171000,172000,173000,174000,175000,176000,177000,178000,179000,180000,181000,182000,183000,184000,185000,186000,187000,188000,189000,190000,191000,192000,193000,194000,195000,196000,197000
NW_003726185_1,Count,0,0,0,0,0,0,0,0,0,0,0,0,0,0,0,0,0,0,0,0,0,0,0,0,0,0,0,0,0,0,0,0,0,0,3,1,0,1,2,0,0,0,0,0,0,16,2,0,0,1,0,0,0,0,0,0,0,0,0,0,0,4,0,0,0,0,0,0,0,0,0,0,0,0,2,0,0,0,0,0,0,0,0,0,2,0,0,3,3,0,0,0,0,0,0,0,0,0,0,0,0,0,1,1,0,0,0,2,0,0,0,0,0,0,0,0,0,0,0,0,0,0,0,0,0,0,0,0,0,0,0,0,0,0,0,0,0,0,0,0,0,0,0,0,0,0,0,0,0,0,0,0,0,0,0,0,0,0,0,0,0,0,0,0,0,0,0,0,0,0,0,0,0,0,0,0,0,0,0,0,0,0,0,0,0,0,0,0,0,0,0,0,3,0,0,0,0,0

	
```

```
		  

		NW_003726186_1, Position,0,1000,2000,3000,4000,5000,6000,7000,8000,9000,10000,11000,12000,13000,14000,15000,16000,17000,18000,19000,20000,21000,22000,23000,24000,25000,26000,27000,28000,29000,30000,31000,32000,33000,34000,35000,36000,37000,38000,39000,40000,41000,42000,43000,44000,45000,46000,47000,48000,49000,50000,51000,52000,53000,54000,55000,56000,57000,58000,59000,60000,61000,62000,63000,64000,65000,66000,67000,68000,69000,70000,71000,72000,73000,74000,75000,76000,77000,78000,79000,80000,81000,82000,83000,84000,85000,86000,87000,88000,89000,90000,91000,92000,93000,94000,95000,96000,97000,98000,99000,100000,101000,102000,103000,104000,105000,106000,107000,108000,109000,110000,111000,112000,113000,114000,115000,116000,117000,118000,119000,120000,121000,122000,123000,124000,125000,126000,127000,128000,129000,130000,131000,132000,133000,134000,135000,136000,137000,138000,139000,140000,141000,142000,143000,144000,145000,146000,147000,148000,149000,150000,151000,152000,153000,154000,155000,156000,157000,158000,159000,160000,161000,162000,163000,164000,165000,166000,167000,168000,169000,170000,171000,172000,173000,174000,175000,176000,177000,178000,179000,180000,181000,182000,183000,184000,185000,186000,187000,188000,189000,190000,191000,192000,193000,194000,195000,196000
NW_003726186_1,Count,0,0,0,0,0,0,0,0,0,0,0,0,0,0,0,0,0,0,0,0,0,0,0,0,0,0,0,0,0,0,0,0,0,0,0,0,0,0,0,0,0,0,2,1,0,3,0,0,0,0,0,0,0,0,0,0,0,0,4,0,0,0,0,0,0,0,0,0,0,0,0,0,0,0,0,0,0,0,0,0,0,3,4,5,0,5,3,0,4,0,0,1,2,0,6,0,2,0,1,7,3,0,0,0,0,3,2,5,1,6,5,0,1,0,5,3,4,0,0,0,2,0,0,0,0,0,0,0,0,0,1,0,0,2,0,0,0,0,0,0,0,0,0,0,0,0,0,0,0,0,0,0,0,0,0,0,0,0,0,0,0,0,0,0,0,0,0,0,0,0,0,0,0,0,0,0,2,0,0,0,0,0,0,0,0,0,1,0,1,0,0,0,0,5,0,0,1

	
```

```
		  

		NW_003726188_1, Position,0,1000,2000,3000,4000,5000,6000,7000,8000,9000,10000,11000,12000,13000,14000,15000,16000,17000,18000,19000,20000,21000,22000,23000,24000,25000,26000,27000,28000,29000,30000,31000,32000,33000,34000,35000,36000,37000,38000,39000,40000,41000,42000,43000,44000,45000,46000,47000,48000,49000,50000,51000,52000,53000,54000,55000,56000,57000,58000,59000,60000,61000,62000,63000,64000,65000,66000,67000,68000,69000,70000,71000,72000,73000,74000,75000,76000,77000,78000,79000,80000,81000,82000,83000,84000,85000,86000,87000,88000,89000,90000,91000,92000,93000,94000,95000,96000,97000,98000,99000,100000,101000,102000,103000,104000,105000,106000,107000,108000,109000,110000,111000,112000,113000,114000,115000,116000,117000,118000,119000,120000,121000,122000,123000,124000,125000,126000,127000,128000,129000,130000,131000,132000,133000,134000,135000,136000,137000,138000,139000,140000,141000,142000,143000,144000,145000,146000,147000,148000,149000,150000,151000,152000,153000,154000,155000,156000,157000,158000,159000,160000,161000,162000,163000,164000,165000,166000,167000,168000,169000,170000,171000,172000,173000,174000,175000,176000,177000,178000,179000,180000,181000,182000,183000,184000,185000,186000,187000,188000,189000,190000,191000,192000,193000,194000,195000,196000,197000,198000,199000,200000,201000,202000,203000,204000,205000,206000,207000,208000,209000,210000
NW_003726188_1,Count,0,0,0,0,0,0,0,0,0,0,0,0,0,0,0,0,0,0,0,0,0,0,0,0,0,0,0,0,0,0,0,0,0,0,0,0,0,0,0,0,0,0,0,0,0,0,0,0,0,0,0,0,0,0,0,2,4,0,0,0,4,0,0,0,0,0,0,0,0,0,0,0,0,0,0,0,0,0,0,0,0,0,0,0,0,0,0,0,0,0,0,0,0,0,0,0,0,0,0,0,0,0,0,0,0,0,4,0,2,3,0,0,0,0,0,0,0,0,0,0,0,0,0,0,0,0,0,0,0,0,0,1,0,0,0,0,0,0,0,0,0,0,0,0,0,0,0,0,0,0,0,0,0,0,0,0,0,0,0,0,0,0,0,0,0,0,0,0,0,0,0,0,0,0,0,0,0,0,0,0,0,0,0,0,0,0,0,0,0,0,0,0,0,0,0,0,0,0,0,0,0,0,0,0,0,0,0,0,0,0,0

	
```

```
		  

		NW_003726189_1, Position,0,1000,2000,3000,4000,5000,6000,7000,8000,9000,10000,11000,12000,13000,14000,15000,16000,17000,18000,19000,20000,21000,22000,23000,24000,25000,26000,27000,28000,29000,30000,31000,32000,33000,34000,35000,36000,37000,38000,39000,40000,41000,42000,43000,44000,45000,46000,47000,48000,49000,50000,51000,52000,53000,54000,55000,56000,57000,58000,59000,60000,61000,62000,63000,64000,65000,66000,67000,68000,69000,70000,71000,72000,73000,74000,75000,76000,77000,78000,79000,80000,81000,82000,83000,84000,85000,86000,87000,88000,89000,90000,91000,92000,93000,94000,95000,96000,97000,98000,99000,100000,101000,102000,103000,104000,105000,106000,107000,108000,109000,110000,111000,112000,113000,114000,115000,116000,117000,118000,119000,120000,121000,122000,123000,124000,125000,126000,127000,128000,129000,130000,131000,132000,133000,134000,135000,136000,137000,138000,139000,140000,141000,142000,143000,144000,145000,146000,147000,148000,149000,150000,151000,152000,153000,154000,155000,156000,157000,158000,159000,160000,161000,162000,163000,164000,165000,166000,167000,168000,169000,170000,171000,172000,173000,174000,175000,176000,177000
NW_003726189_1,Count,0,0,0,0,0,0,0,0,0,0,0,0,0,0,0,0,0,0,0,0,0,0,0,0,0,0,0,0,0,0,0,0,0,0,0,0,0,0,0,0,0,0,0,0,0,0,0,0,0,0,0,0,0,0,0,0,0,0,0,0,3,0,7,0,0,0,0,0,0,0,0,0,0,0,0,0,0,0,0,0,0,0,0,0,0,0,0,0,0,0,0,0,0,0,0,0,0,0,0,0,0,0,0,0,0,0,0,0,0,0,0,0,0,0,0,0,0,0,0,0,0,0,0,0,0,0,0,0,0,0,0,0,0,0,0,0,0,0,0,0,0,0,5,0,0,0,0,0,0,0,0,0,0,0,0,0,0,0,0,0,0,0,0,0,0,0,0,0,0,0,0,0,0,0,0,0,0,0

	
```

```
		  

		NW_003726191_1, Position,0,1000,2000,3000,4000,5000,6000,7000,8000,9000,10000,11000,12000,13000,14000,15000,16000,17000,18000,19000,20000,21000,22000,23000,24000,25000,26000,27000,28000,29000,30000,31000,32000,33000,34000,35000,36000,37000,38000,39000,40000,41000,42000,43000,44000,45000,46000,47000,48000,49000,50000,51000,52000,53000,54000,55000,56000,57000,58000,59000,60000,61000,62000,63000,64000,65000,66000,67000,68000,69000,70000,71000,72000,73000,74000,75000,76000,77000,78000,79000,80000,81000,82000,83000,84000,85000,86000,87000,88000,89000,90000,91000,92000,93000,94000,95000,96000,97000,98000,99000,100000,101000,102000,103000,104000,105000,106000,107000,108000,109000,110000,111000,112000,113000,114000,115000,116000,117000,118000,119000,120000,121000,122000,123000,124000,125000,126000,127000,128000,129000,130000,131000,132000,133000,134000,135000,136000,137000,138000,139000,140000,141000,142000,143000,144000,145000,146000,147000,148000,149000,150000,151000,152000,153000,154000,155000,156000,157000,158000,159000,160000,161000,162000,163000,164000,165000,166000
NW_003726191_1,Count,0,0,0,0,0,0,0,0,0,0,0,0,0,0,0,0,0,0,0,0,0,0,0,0,0,0,0,0,0,0,0,0,0,0,0,0,0,0,0,0,0,0,0,0,0,0,0,0,0,0,0,0,0,0,0,0,0,0,0,0,0,0,0,0,0,0,0,1,0,0,0,0,0,0,0,0,0,0,0,0,0,0,0,0,0,0,0,0,0,0,0,0,0,0,0,0,0,0,0,0,0,0,0,0,0,0,0,2,0,0,0,0,4,0,0,0,0,0,0,0,0,2,0,0,3,5,0,0,0,0,0,0,0,0,0,5,16,4,2,0,0,0,0,0,0,0,0,0,0,0,0,0,0,0,0,0,0,0,0,0,0,10,4,2,5,0,0

	
```

```
		  

		NW_003726193_1, Position,0,1000,2000,3000,4000,5000,6000,7000,8000,9000,10000,11000,12000,13000,14000,15000,16000,17000,18000,19000,20000,21000,22000,23000,24000,25000,26000,27000,28000,29000,30000,31000,32000,33000,34000,35000,36000,37000,38000,39000,40000,41000,42000,43000,44000,45000,46000,47000,48000,49000,50000,51000,52000,53000,54000,55000,56000,57000,58000,59000,60000,61000,62000,63000,64000,65000,66000,67000,68000,69000,70000,71000,72000,73000,74000,75000,76000,77000,78000,79000,80000,81000,82000,83000,84000,85000,86000,87000,88000,89000,90000,91000,92000,93000,94000,95000,96000,97000,98000,99000,100000,101000,102000,103000,104000,105000,106000,107000,108000,109000,110000,111000,112000,113000,114000,115000,116000,117000,118000,119000,120000,121000,122000,123000,124000,125000,126000,127000,128000,129000,130000,131000,132000,133000,134000,135000,136000,137000,138000,139000,140000,141000,142000,143000,144000,145000,146000,147000,148000,149000,150000,151000,152000,153000,154000,155000,156000,157000
NW_003726193_1,Count,0,0,0,0,0,0,0,0,0,0,0,0,0,0,0,0,0,0,0,1,0,0,0,0,0,0,0,0,0,0,0,0,0,0,0,0,0,0,0,0,0,0,0,0,0,0,0,0,0,0,0,0,0,0,0,0,0,0,0,0,0,0,0,0,0,0,0,0,1,0,0,0,0,0,0,0,2,0,0,1,0,0,0,0,2,2,2,0,2,0,0,0,0,0,0,0,2,0,0,0,1,8,0,0,2,6,1,0,2,0,0,0,0,6,2,7,3,0,0,0,0,0,0,0,1,0,0,0,0,0,0,0,0,0,0,1,13,3,1,2,0,0,3,0,6,0,4,0,0,0,0,0,0,0,0,7,0,0

	
```

```
		  

		NW_003726194_1, Position,0,1000,2000,3000,4000,5000,6000,7000,8000,9000,10000,11000,12000,13000,14000,15000,16000,17000,18000,19000,20000,21000,22000,23000,24000,25000,26000,27000,28000,29000,30000,31000,32000,33000,34000,35000,36000,37000,38000,39000,40000,41000,42000,43000,44000,45000,46000,47000,48000,49000,50000,51000,52000,53000,54000,55000,56000,57000,58000,59000,60000,61000,62000,63000,64000,65000,66000,67000,68000,69000,70000,71000,72000,73000,74000,75000,76000,77000,78000,79000,80000,81000,82000,83000,84000,85000,86000,87000,88000,89000,90000,91000,92000,93000,94000,95000,96000,97000,98000,99000,100000,101000,102000,103000,104000,105000,106000,107000,108000,109000,110000,111000,112000,113000,114000,115000,116000,117000,118000,119000,120000,121000,122000,123000,124000,125000,126000,127000,128000,129000,130000,131000,132000,133000,134000,135000,136000,137000,138000,139000,140000,141000,142000,143000
NW_003726194_1,Count,9,11,7,11,6,17,14,13,11,13,11,14,15,8,8,14,17,14,13,10,7,10,16,13,13,13,6,9,12,2,1,12,18,12,10,7,15,19,11,8,5,11,11,13,8,16,9,8,5,7,14,3,18,12,14,13,13,11,11,15,16,12,16,14,10,13,10,18,18,14,15,13,7,14,3,12,14,10,11,12,4,4,7,15,16,12,17,8,6,14,17,16,9,7,9,4,13,8,17,7,9,15,18,10,18,9,11,8,11,16,15,10,12,8,8,9,7,8,11,15,10,13,7,12,11,19,6,16,15,9,18,3,0,3,12,9,9,4,11,14,9,11,13,13

	
```

```
		  

		NW_003726195_1, Position,0,1000,2000,3000,4000,5000,6000,7000,8000,9000,10000,11000,12000,13000,14000,15000,16000,17000,18000,19000,20000,21000,22000,23000,24000,25000,26000,27000,28000,29000,30000,31000,32000,33000,34000,35000,36000,37000,38000,39000,40000,41000,42000,43000,44000,45000,46000,47000,48000,49000,50000,51000,52000,53000,54000,55000,56000,57000,58000,59000,60000,61000,62000,63000,64000,65000,66000,67000,68000,69000,70000,71000,72000,73000,74000,75000,76000,77000,78000,79000,80000,81000,82000,83000,84000,85000,86000,87000,88000,89000,90000,91000,92000,93000,94000,95000,96000,97000,98000,99000,100000,101000,102000,103000,104000,105000,106000,107000,108000,109000,110000,111000,112000,113000,114000,115000,116000,117000,118000,119000,120000,121000,122000,123000,124000,125000,126000,127000,128000,129000,130000,131000,132000,133000,134000,135000,136000,137000,138000,139000,140000,141000
NW_003726195_1,Count,4,2,5,0,3,9,0,4,3,3,5,8,3,14,10,2,2,0,0,0,0,0,0,0,0,2,0,0,4,10,11,6,3,9,5,5,4,2,10,13,2,8,8,9,8,5,1,0,0,9,19,1,9,8,4,6,13,10,6,7,0,0,0,5,12,11,10,12,8,7,8,6,6,7,10,14,12,7,8,8,13,13,7,3,4,5,6,5,4,10,5,8,10,8,0,0,0,0,6,2,0,0,7,9,7,11,7,6,9,10,5,8,0,0,0,0,0,8,13,7,8,0,0,0,0,0,0,0,4,4,10,9,6,11,9,15,13,3,0,0,0,0

	
```

```
		  

		NW_003726197_1, Position,0,1000,2000,3000,4000,5000,6000,7000,8000,9000,10000,11000,12000,13000,14000,15000,16000,17000,18000,19000,20000,21000,22000,23000,24000,25000,26000,27000,28000,29000,30000,31000,32000,33000,34000,35000,36000,37000,38000,39000,40000,41000,42000,43000,44000,45000,46000,47000,48000,49000,50000,51000,52000,53000,54000,55000,56000,57000,58000,59000,60000,61000,62000,63000,64000,65000,66000,67000,68000,69000,70000,71000,72000,73000,74000,75000,76000,77000,78000,79000,80000,81000,82000,83000,84000,85000,86000,87000,88000,89000,90000,91000,92000,93000,94000,95000,96000,97000,98000,99000,100000,101000,102000,103000,104000,105000,106000,107000,108000,109000,110000,111000,112000,113000,114000,115000,116000,117000,118000,119000,120000,121000,122000,123000,124000,125000,126000,127000,128000,129000,130000,131000,132000,133000,134000,135000,136000,137000,138000,139000,140000,141000,142000
NW_003726197_1,Count,0,0,0,0,0,0,0,0,4,0,1,0,1,3,5,4,6,9,2,1,1,6,4,1,3,1,2,2,1,0,5,1,0,0,5,5,0,3,2,1,5,0,2,0,3,0,2,1,6,1,4,2,3,1,3,0,0,0,0,0,0,0,0,0,0,0,0,0,0,0,0,0,0,0,0,0,0,0,0,0,0,0,0,0,0,0,0,0,0,0,0,0,0,0,0,0,0,0,0,0,0,0,0,0,0,0,0,0,0,0,0,0,0,0,0,0,0,0,0,0,0,0,0,0,0,0,0,0,0,0,0,2,1,0,3,6,4,2,0,0,0,0,0

	
```

```
		  

		NW_003726199_1, Position,0,1000,2000,3000,4000,5000,6000,7000,8000,9000,10000,11000,12000,13000,14000,15000,16000,17000,18000,19000,20000,21000,22000,23000,24000,25000,26000,27000,28000,29000,30000,31000,32000,33000,34000,35000,36000,37000,38000,39000,40000,41000,42000,43000,44000,45000,46000,47000,48000,49000,50000,51000,52000,53000,54000,55000,56000,57000,58000,59000,60000,61000,62000,63000,64000,65000,66000,67000,68000,69000,70000,71000,72000,73000,74000,75000,76000,77000,78000,79000,80000,81000,82000,83000,84000,85000,86000,87000,88000,89000,90000,91000,92000,93000,94000,95000,96000,97000,98000,99000,100000,101000,102000,103000,104000,105000,106000,107000,108000,109000,110000,111000,112000,113000,114000,115000,116000,117000,118000,119000,120000,121000,122000,123000,124000,125000,126000,127000,128000,129000,130000,131000,132000,133000,134000,135000,136000,137000,138000,139000,140000,141000,142000
NW_003726199_1,Count,0,0,0,0,0,0,0,0,0,0,0,0,0,0,0,0,0,0,0,0,0,0,0,0,2,0,0,0,0,0,0,0,0,0,0,0,0,0,0,0,0,0,0,0,0,0,0,0,0,0,0,0,0,3,0,2,4,0,0,0,0,3,0,0,0,0,0,0,0,0,0,0,0,0,0,0,0,0,0,0,0,0,0,0,0,0,0,0,0,0,0,0,0,0,0,0,0,0,0,0,0,0,0,0,0,0,0,0,0,0,0,0,0,0,0,0,0,0,0,0,0,0,0,0,0,0,0,0,0,0,0,0,0,0,0,0,0,0,0,0,0,0,0

	
```

```
		  

		NW_003726200_1, Position,0,1000,2000,3000,4000,5000,6000,7000,8000,9000,10000,11000,12000,13000,14000,15000,16000,17000,18000,19000,20000,21000,22000,23000,24000,25000,26000,27000,28000,29000,30000,31000,32000,33000,34000,35000,36000,37000,38000,39000,40000,41000,42000,43000,44000,45000,46000,47000,48000,49000,50000,51000,52000,53000,54000,55000,56000,57000,58000,59000,60000,61000,62000,63000,64000,65000,66000,67000,68000,69000,70000,71000,72000,73000,74000,75000,76000,77000,78000,79000,80000,81000,82000,83000,84000,85000,86000,87000,88000,89000,90000,91000,92000,93000,94000,95000,96000,97000,98000,99000,100000,101000,102000,103000,104000,105000,106000,107000,108000,109000,110000,111000,112000,113000,114000,115000,116000,117000,118000,119000,120000,121000,122000,123000,124000,125000,126000,127000,128000,129000,130000,131000,132000,133000
NW_003726200_1,Count,0,0,0,0,0,0,0,0,0,0,0,0,0,0,0,0,0,0,0,0,0,0,0,0,0,0,0,0,0,0,0,0,0,0,0,0,0,0,0,0,0,0,0,0,0,0,0,0,0,0,0,6,2,11,4,2,2,1,6,6,9,6,3,5,4,0,4,2,6,10,6,12,1,5,5,11,1,3,11,0,0,0,0,0,0,0,0,3,0,0,0,0,0,0,0,0,0,0,0,0,0,0,0,0,0,0,0,0,0,0,0,0,0,0,0,0,0,0,0,0,0,0,0,0,0,0,0,0,0,0,0,0,0,0

	
```

```
		  

		NW_003726201_1, Position,0,1000,2000,3000,4000,5000,6000,7000,8000,9000,10000,11000,12000,13000,14000,15000,16000,17000,18000,19000,20000,21000,22000,23000,24000,25000,26000,27000,28000,29000,30000,31000,32000,33000,34000,35000,36000,37000,38000,39000,40000,41000,42000,43000,44000,45000,46000,47000,48000,49000,50000,51000,52000,53000,54000,55000,56000,57000,58000,59000,60000,61000,62000,63000,64000,65000,66000,67000,68000,69000,70000,71000,72000,73000,74000,75000,76000,77000,78000,79000,80000,81000,82000,83000,84000,85000,86000,87000,88000,89000,90000,91000,92000,93000,94000,95000,96000,97000,98000,99000,100000,101000,102000,103000,104000,105000,106000,107000,108000,109000,110000,111000,112000,113000,114000,115000,116000,117000,118000,119000,120000,121000,122000,123000,124000,125000,126000,127000,128000,129000
NW_003726201_1,Count,3,2,0,5,0,0,0,0,0,2,0,0,0,0,0,0,0,8,1,1,0,0,1,1,4,0,0,0,8,0,3,2,0,0,0,0,0,0,0,0,0,0,2,1,0,0,0,0,1,0,1,2,0,4,5,0,0,0,0,0,0,0,0,0,5,2,0,4,5,0,0,0,0,3,4,5,0,0,0,8,0,0,0,0,0,3,0,0,0,0,0,0,0,0,2,4,0,0,0,5,7,2,6,10,7,3,7,10,2,0,6,4,0,1,0,3,0,0,0,0,0,0,0,0,0,4,0,1,1,0

	
```

```
		  

		NW_003726203_1, Position,0,1000,2000,3000,4000,5000,6000,7000,8000,9000,10000,11000,12000,13000,14000,15000,16000,17000,18000,19000,20000,21000,22000,23000,24000,25000,26000,27000,28000,29000,30000,31000,32000,33000,34000,35000,36000,37000,38000,39000,40000,41000,42000,43000,44000,45000,46000,47000,48000,49000,50000,51000,52000,53000,54000,55000,56000,57000,58000,59000,60000,61000,62000,63000,64000,65000,66000,67000,68000,69000,70000,71000,72000,73000,74000,75000,76000,77000,78000,79000,80000,81000,82000,83000,84000,85000,86000,87000,88000,89000,90000,91000,92000,93000,94000,95000,96000,97000,98000,99000,100000,101000,102000,103000,104000,105000,106000,107000,108000,109000,110000,111000,112000,113000,114000,115000,116000,117000,118000,119000,120000,121000,122000,123000,124000,125000,126000,127000
NW_003726203_1,Count,1,21,13,4,13,8,7,10,12,9,5,0,5,1,3,0,6,4,4,6,0,4,2,2,1,5,4,4,2,6,1,6,3,7,0,3,1,4,3,2,6,2,2,0,3,4,0,0,3,2,1,1,2,0,3,9,5,5,4,8,0,0,3,1,0,2,5,1,0,0,6,2,2,2,4,2,0,7,0,0,1,4,5,1,0,0,2,4,1,5,1,5,4,8,8,1,5,7,1,3,9,10,6,4,6,2,12,12,10,5,9,11,9,7,10,7,4,9,3,2,9,8,12,10,7,4,8,3

	
```

```
		  

		NW_003726205_1, Position,0,1000,2000,3000,4000,5000,6000,7000,8000,9000,10000,11000,12000,13000,14000,15000,16000,17000,18000,19000,20000,21000,22000,23000,24000,25000,26000,27000,28000,29000,30000,31000,32000,33000,34000,35000,36000,37000,38000,39000,40000,41000,42000,43000,44000,45000,46000,47000,48000,49000,50000,51000,52000,53000,54000,55000,56000,57000,58000,59000,60000,61000,62000,63000,64000,65000,66000,67000,68000,69000,70000,71000,72000,73000,74000,75000,76000,77000,78000,79000,80000,81000,82000,83000,84000,85000,86000,87000,88000,89000,90000,91000,92000,93000,94000,95000,96000,97000,98000,99000,100000,101000,102000,103000,104000,105000,106000,107000,108000,109000,110000,111000,112000,113000,114000,115000,116000,117000,118000,119000,120000,121000,122000,123000,124000,125000,126000,127000,128000,129000,130000,131000
NW_003726205_1,Count,6,3,4,4,4,7,7,10,1,4,9,5,10,6,7,1,0,0,0,0,0,2,9,0,2,2,3,11,8,2,4,4,9,3,7,2,7,12,9,7,15,14,9,8,8,9,5,7,7,4,4,6,4,7,8,1,5,3,1,1,0,0,0,0,0,0,2,5,0,0,1,1,11,4,7,0,0,0,0,0,0,0,0,3,4,3,4,2,12,4,9,3,1,8,9,8,8,2,8,8,4,0,0,0,0,0,0,0,0,0,0,0,0,0,0,0,0,0,0,0,0,0,0,0,0,0,0,0,0,2,2,0

	
```

```
		  

		NW_003726206_1, Position,0,1000,2000,3000,4000,5000,6000,7000,8000,9000,10000,11000,12000,13000,14000,15000,16000,17000,18000,19000,20000,21000,22000,23000,24000,25000,26000,27000,28000,29000,30000,31000,32000,33000,34000,35000,36000,37000,38000,39000,40000,41000,42000,43000,44000,45000,46000,47000,48000,49000,50000,51000,52000,53000,54000,55000,56000,57000,58000,59000,60000,61000,62000,63000,64000,65000,66000,67000,68000,69000,70000,71000,72000,73000,74000,75000,76000,77000,78000,79000,80000,81000,82000,83000,84000,85000,86000,87000,88000,89000,90000,91000,92000,93000,94000,95000,96000,97000,98000,99000,100000,101000,102000,103000,104000,105000,106000,107000,108000,109000,110000,111000,112000,113000,114000,115000,116000,117000,118000,119000,120000,121000,122000,123000,124000,125000,126000,127000,128000,129000,130000,131000,132000,133000
NW_003726206_1,Count,3,0,0,0,0,0,0,0,0,0,0,0,0,0,0,0,0,0,0,0,0,0,0,0,0,0,0,0,0,0,0,0,0,0,0,0,0,0,0,0,0,0,0,0,0,0,0,0,0,0,0,0,0,0,0,0,0,0,0,0,0,0,0,0,0,0,0,0,0,0,0,0,0,0,0,0,0,0,0,0,0,0,0,0,0,2,6,2,4,1,9,8,10,3,4,6,9,8,11,4,5,12,6,5,6,5,0,4,0,0,4,3,0,4,4,1,10,2,5,2,4,5,8,6,3,4,8,6,0,6,8,2,4,0

	
```

```
		  

		NW_003726210_1, Position,0,1000,2000,3000,4000,5000,6000,7000,8000,9000,10000,11000,12000,13000,14000,15000,16000,17000,18000,19000,20000,21000,22000,23000,24000,25000,26000,27000,28000,29000,30000,31000,32000,33000,34000,35000,36000,37000,38000,39000,40000,41000,42000,43000,44000,45000,46000,47000,48000,49000,50000,51000,52000,53000,54000,55000,56000,57000,58000,59000,60000,61000,62000,63000,64000,65000,66000,67000,68000,69000,70000,71000,72000,73000,74000,75000,76000,77000,78000,79000,80000,81000,82000,83000,84000,85000,86000,87000,88000,89000,90000,91000,92000,93000,94000,95000,96000,97000,98000,99000,100000,101000,102000,103000,104000,105000,106000,107000,108000,109000,110000,111000,112000,113000,114000,115000
NW_003726210_1,Count,0,0,0,0,0,0,0,0,0,0,0,0,0,0,0,0,0,0,0,0,0,0,0,0,0,5,0,0,0,7,0,10,8,11,2,5,14,12,10,16,8,6,4,9,0,4,0,0,0,6,2,5,0,5,5,17,10,8,20,17,7,7,11,3,0,0,3,0,0,0,4,0,0,0,0,4,6,2,0,10,13,11,11,18,16,13,17,10,12,17,12,17,17,9,0,0,0,4,3,0,0,0,0,9,7,13,9,3,12,0,12,8,7,6,19,10

	
```

```
		  

		NW_003726211_1, Position,0,1000,2000,3000,4000,5000,6000,7000,8000,9000,10000,11000,12000,13000,14000,15000,16000,17000,18000,19000,20000,21000,22000,23000,24000,25000,26000,27000,28000,29000,30000,31000,32000,33000,34000,35000,36000,37000,38000,39000,40000,41000,42000,43000,44000,45000,46000,47000,48000,49000,50000,51000,52000,53000,54000,55000,56000,57000,58000,59000,60000,61000,62000,63000,64000,65000,66000,67000,68000,69000,70000,71000,72000,73000,74000,75000,76000,77000,78000,79000,80000,81000,82000,83000,84000,85000,86000,87000,88000,89000,90000,91000,92000,93000,94000,95000,96000,97000,98000,99000,100000,101000,102000,103000,104000,105000,106000,107000,108000,109000,110000,111000,112000,113000,114000,115000,116000,117000,118000
NW_003726211_1,Count,0,0,0,0,0,0,0,0,3,0,0,0,0,0,0,0,0,0,0,0,0,0,0,0,0,0,0,0,0,0,0,0,0,0,0,0,0,0,0,0,0,0,0,0,0,0,0,0,0,0,0,0,0,0,0,0,0,0,0,0,0,0,0,0,0,0,0,0,0,0,0,0,0,0,0,0,0,0,0,0,0,0,0,0,0,0,0,0,0,0,0,0,0,0,0,0,0,0,0,0,0,0,0,0,0,0,0,0,0,0,0,0,0,0,0,0,0,0,0

	
```

```
		  

		NW_003726213_1, Position,0,1000,2000,3000,4000,5000,6000,7000,8000,9000,10000,11000,12000,13000,14000,15000,16000,17000,18000,19000,20000,21000,22000,23000,24000,25000,26000,27000,28000,29000,30000,31000,32000,33000,34000,35000,36000,37000,38000,39000,40000,41000,42000,43000,44000,45000,46000,47000,48000,49000,50000,51000,52000,53000,54000,55000,56000,57000,58000,59000,60000,61000,62000,63000,64000,65000,66000,67000,68000,69000,70000,71000,72000,73000,74000,75000,76000,77000,78000,79000,80000,81000,82000,83000,84000,85000,86000,87000,88000,89000,90000,91000,92000,93000,94000,95000,96000,97000,98000,99000,100000,101000,102000,103000,104000,105000,106000,107000,108000,109000,110000,111000,112000,113000,114000,115000,116000,117000
NW_003726213_1,Count,0,0,0,0,0,0,0,0,0,0,0,0,0,0,0,0,0,0,0,0,0,0,0,0,0,0,0,0,0,0,0,0,0,0,0,0,0,0,0,0,0,0,0,0,0,0,0,0,0,0,0,0,0,0,0,0,0,0,0,0,0,0,0,0,0,0,0,0,0,0,0,0,0,0,0,0,0,0,0,0,0,0,0,0,0,0,0,0,0,0,0,0,0,0,0,0,0,0,0,0,0,0,0,0,0,0,0,0,0,0,0,0,0,0,0,1,0,0

	
```

```
		  

		NW_003726214_1, Position,0,1000,2000,3000,4000,5000,6000,7000,8000,9000,10000,11000,12000,13000,14000,15000,16000,17000,18000,19000,20000,21000,22000,23000,24000,25000,26000,27000,28000,29000,30000,31000,32000,33000,34000,35000,36000,37000,38000,39000,40000,41000,42000,43000,44000,45000,46000,47000,48000,49000,50000,51000,52000,53000,54000,55000,56000,57000,58000,59000,60000,61000,62000,63000,64000,65000,66000,67000,68000,69000,70000,71000,72000,73000,74000,75000,76000,77000,78000,79000,80000,81000,82000,83000,84000,85000,86000,87000,88000,89000,90000,91000,92000,93000,94000,95000,96000,97000,98000,99000,100000,101000,102000,103000,104000,105000,106000,107000,108000,109000,110000,111000
NW_003726214_1,Count,13,11,8,5,7,7,10,11,3,8,8,7,6,5,7,8,11,13,6,1,7,5,9,4,9,5,9,5,10,7,4,3,3,1,5,5,6,6,6,6,2,3,17,5,9,4,3,13,5,10,10,6,12,10,6,12,6,6,9,3,6,12,4,12,15,9,13,7,11,6,6,11,10,11,8,12,10,10,11,8,8,10,11,8,15,14,16,11,7,8,8,8,5,8,10,5,1,9,4,14,12,16,16,10,8,17,20,9,9,8,4,6

	
```

```
		  

		NW_003726216_1, Position,0,1000,2000,3000,4000,5000,6000,7000,8000,9000,10000,11000,12000,13000,14000,15000,16000,17000,18000,19000,20000,21000,22000,23000,24000,25000,26000,27000,28000,29000,30000,31000,32000,33000,34000,35000,36000,37000,38000,39000,40000,41000,42000,43000,44000,45000,46000,47000,48000,49000,50000,51000,52000,53000,54000,55000,56000,57000,58000,59000,60000,61000,62000,63000,64000,65000,66000,67000,68000,69000,70000,71000,72000,73000,74000,75000,76000,77000,78000,79000,80000,81000,82000,83000,84000,85000,86000,87000,88000,89000,90000,91000,92000,93000,94000,95000,96000,97000,98000,99000,100000,101000,102000,103000,104000,105000,106000,107000,108000,109000,110000,111000,112000
NW_003726216_1,Count,0,0,0,0,0,0,0,0,0,0,0,0,0,0,0,0,0,0,0,0,0,0,0,0,0,0,0,0,0,0,0,0,0,0,0,0,0,0,0,0,0,0,0,0,0,0,0,0,0,0,0,0,0,2,0,0,0,0,0,0,0,0,0,0,0,0,0,0,0,0,0,0,0,0,0,0,0,0,0,0,0,0,0,1,0,0,0,0,0,2,0,0,0,0,0,0,0,0,0,0,0,4,0,0,0,0,0,0,0,0,0,0,0

	
```

```
		  

		NW_003726217_1, Position,0,1000,2000,3000,4000,5000,6000,7000,8000,9000,10000,11000,12000,13000,14000,15000,16000,17000,18000,19000,20000,21000,22000,23000,24000,25000,26000,27000,28000,29000,30000,31000,32000,33000,34000,35000,36000,37000,38000,39000,40000,41000,42000,43000,44000,45000,46000,47000,48000,49000,50000,51000,52000,53000,54000,55000,56000,57000,58000,59000,60000,61000,62000,63000,64000,65000,66000,67000,68000,69000,70000,71000,72000,73000,74000,75000,76000,77000,78000,79000,80000,81000,82000,83000,84000,85000,86000,87000,88000,89000,90000,91000,92000,93000,94000,95000,96000,97000,98000,99000,100000,101000,102000,103000,104000,105000,106000
NW_003726217_1,Count,0,0,0,0,0,0,0,0,0,0,0,0,0,0,0,0,0,0,0,0,0,0,0,0,0,0,0,0,0,0,0,0,0,0,0,0,0,0,0,0,0,0,0,0,0,0,0,0,0,0,0,0,0,0,0,0,0,0,0,0,0,0,0,0,0,0,2,0,0,0,0,0,0,0,0,0,0,0,0,0,0,0,0,0,0,0,0,0,0,0,0,0,0,0,0,0,0,0,0,0,0,0,0,0,0,0,0

	
```

```
		  

		NW_003726220_1, Position,0,1000,2000,3000,4000,5000,6000,7000,8000,9000,10000,11000,12000,13000,14000,15000,16000,17000,18000,19000,20000,21000,22000,23000,24000,25000,26000,27000,28000,29000,30000,31000,32000,33000,34000,35000,36000,37000,38000,39000,40000,41000,42000,43000,44000,45000,46000,47000,48000,49000,50000,51000,52000,53000,54000,55000,56000,57000,58000,59000,60000,61000,62000,63000,64000,65000,66000,67000,68000,69000,70000,71000,72000,73000,74000,75000,76000,77000,78000,79000,80000,81000,82000,83000,84000,85000,86000,87000,88000,89000,90000,91000,92000,93000,94000,95000,96000,97000,98000,99000,100000,101000,102000,103000
NW_003726220_1,Count,0,0,0,0,0,0,0,0,0,0,0,0,0,0,0,0,0,0,0,0,0,0,0,0,0,0,0,0,0,0,0,0,0,0,0,0,0,0,0,0,0,0,0,0,0,0,0,0,0,0,0,0,0,0,0,0,0,0,0,0,0,0,0,0,0,0,0,0,0,0,0,0,0,0,0,0,0,0,0,0,0,0,0,0,0,0,0,0,0,0,0,0,0,0,0,0,0,0,0,0,2,2,0,0

	
```

```
		  

		NW_003726222_1, Position,0,1000,2000,3000,4000,5000,6000,7000,8000,9000,10000,11000,12000,13000,14000,15000,16000,17000,18000,19000,20000,21000,22000,23000,24000,25000,26000,27000,28000,29000,30000,31000,32000,33000,34000,35000,36000,37000,38000,39000,40000,41000,42000,43000,44000,45000,46000,47000,48000,49000,50000,51000,52000,53000,54000,55000,56000,57000,58000,59000,60000,61000,62000,63000,64000,65000,66000,67000,68000,69000,70000,71000,72000,73000,74000,75000,76000,77000,78000,79000,80000,81000,82000,83000,84000,85000,86000,87000,88000,89000,90000,91000,92000,93000,94000,95000,96000,97000,98000,99000,100000,101000,102000,103000,104000,105000,106000,107000,108000,109000,110000,111000,112000,113000,114000,115000,116000,117000,118000
NW_003726222_1,Count,1,2,0,0,0,0,0,0,0,0,0,0,0,0,0,0,0,0,0,0,0,0,0,0,0,0,0,0,1,0,0,0,6,4,0,3,9,14,13,11,2,3,12,1,0,0,5,0,2,3,3,0,0,8,8,6,4,4,5,6,8,7,6,9,9,7,7,11,9,10,0,3,14,8,2,8,10,13,6,11,17,6,12,7,2,0,1,8,1,1,9,8,0,2,15,10,10,6,5,6,5,8,1,8,6,1,0,4,9,0,12,8,9,12,7,11,9,6,2

	
```

```
		  

		NW_003726224_1, Position,0,1000,2000,3000,4000,5000,6000,7000,8000,9000,10000,11000,12000,13000,14000,15000,16000,17000,18000,19000,20000,21000,22000,23000,24000,25000,26000,27000,28000,29000,30000,31000,32000,33000,34000,35000,36000,37000,38000,39000,40000,41000,42000,43000,44000,45000,46000,47000,48000,49000,50000,51000,52000,53000,54000,55000,56000,57000,58000,59000,60000,61000,62000,63000,64000,65000,66000,67000,68000,69000,70000,71000,72000,73000,74000,75000,76000,77000,78000,79000,80000,81000,82000,83000,84000,85000,86000,87000,88000,89000,90000,91000,92000,93000,94000,95000,96000,97000,98000,99000,100000,101000,102000,103000,104000,105000,106000,107000,108000,109000
NW_003726224_1,Count,0,0,0,5,0,9,0,5,0,0,0,0,0,0,0,0,0,0,0,0,0,0,0,0,0,0,0,0,0,0,0,0,0,0,0,0,0,0,0,0,0,0,0,0,0,0,0,0,0,0,0,0,0,0,0,0,0,0,0,0,0,0,0,0,0,0,0,0,0,0,2,0,0,0,0,0,0,0,0,0,0,0,0,0,0,0,0,0,0,0,0,0,0,0,0,3,0,0,0,0,0,0,0,0,0,0,0,0,3,0

	
```

```
		  

		NW_003726225_1, Position,0,1000,2000,3000,4000,5000,6000,7000,8000,9000,10000,11000,12000,13000,14000,15000,16000,17000,18000,19000,20000,21000,22000,23000,24000,25000,26000,27000,28000,29000,30000,31000,32000,33000,34000,35000,36000,37000,38000,39000,40000,41000,42000,43000,44000,45000,46000,47000,48000,49000,50000,51000,52000,53000,54000,55000,56000,57000,58000,59000,60000,61000,62000,63000,64000,65000,66000,67000,68000,69000,70000,71000,72000,73000,74000,75000,76000,77000,78000,79000,80000,81000,82000,83000,84000,85000,86000,87000,88000,89000,90000,91000,92000,93000,94000,95000,96000,97000,98000,99000,100000,101000,102000,103000,104000,105000,106000,107000,108000,109000,110000,111000,112000,113000,114000,115000,116000,117000,118000,119000,120000,121000,122000,123000,124000
NW_003726225_1,Count,3,0,1,2,0,0,0,1,0,0,0,0,0,0,0,7,5,3,2,0,0,0,0,0,4,3,0,2,0,0,0,0,0,0,0,0,0,0,0,0,0,0,0,0,0,0,0,0,0,0,0,0,0,0,0,0,0,0,0,0,0,0,0,0,0,0,0,0,0,0,0,0,0,0,0,0,0,0,0,0,0,0,0,0,0,2,0,0,0,0,0,0,0,0,0,0,0,0,0,0,0,0,0,0,0,0,0,0,0,0,7,0,0,0,0,1,4,0,0,0,1,0,0,0,0

	
```

```
		  

		NW_003726227_1, Position,0,1000,2000,3000,4000,5000,6000,7000,8000,9000,10000,11000,12000,13000,14000,15000,16000,17000,18000,19000,20000,21000,22000,23000,24000,25000,26000,27000,28000,29000,30000,31000,32000,33000,34000,35000,36000,37000,38000,39000,40000,41000,42000,43000,44000,45000,46000,47000,48000,49000,50000,51000,52000,53000,54000,55000,56000,57000,58000,59000,60000,61000,62000,63000,64000,65000,66000,67000,68000,69000,70000,71000,72000,73000,74000,75000,76000,77000,78000,79000,80000,81000,82000,83000,84000,85000,86000,87000,88000,89000,90000,91000,92000,93000,94000,95000,96000,97000,98000,99000,100000,101000
NW_003726227_1,Count,2,8,6,10,2,1,2,10,8,4,7,3,3,2,7,8,3,3,4,10,4,9,13,5,6,8,4,5,3,2,9,3,5,6,5,8,6,3,1,5,4,5,4,3,3,5,4,0,5,8,6,2,3,4,2,7,2,2,5,11,3,7,9,6,2,6,0,9,1,7,7,4,4,4,3,6,8,10,12,9,7,6,5,1,5,9,8,6,7,6,15,13,7,5,8,4,10,4,8,0,0,0

	
```

```
		  

		NW_003726228_1, Position,0,1000,2000,3000,4000,5000,6000,7000,8000,9000,10000,11000,12000,13000,14000,15000,16000,17000,18000,19000,20000,21000,22000,23000,24000,25000,26000,27000,28000,29000,30000,31000,32000,33000,34000,35000,36000,37000,38000,39000,40000,41000,42000,43000,44000,45000,46000,47000,48000,49000,50000,51000,52000,53000,54000,55000,56000,57000,58000,59000,60000,61000,62000,63000,64000,65000,66000,67000,68000,69000,70000,71000,72000,73000,74000,75000,76000,77000,78000,79000,80000,81000,82000,83000,84000,85000,86000,87000,88000,89000,90000,91000,92000,93000,94000,95000,96000,97000
NW_003726228_1,Count,0,0,0,0,0,0,0,0,0,0,0,0,0,0,0,0,0,0,0,0,0,0,0,0,0,0,0,0,0,0,0,0,0,0,0,0,0,0,0,0,0,0,0,0,0,0,0,0,0,0,0,0,0,0,0,0,0,0,0,0,0,0,0,0,0,0,1,0,0,0,0,0,0,0,0,0,0,0,0,0,0,0,0,0,0,0,0,0,0,0,0,0,0,0,0,0,0,0

	
```

```
		  

		NW_003726229_1, Position,0,1000,2000,3000,4000,5000,6000,7000,8000,9000,10000,11000,12000,13000,14000,15000,16000,17000,18000,19000,20000,21000,22000,23000,24000,25000,26000,27000,28000,29000,30000,31000,32000,33000,34000,35000,36000,37000,38000,39000,40000,41000,42000,43000,44000,45000,46000,47000,48000,49000,50000,51000,52000,53000,54000,55000,56000,57000,58000,59000,60000,61000,62000,63000,64000,65000,66000,67000,68000,69000,70000,71000,72000,73000,74000,75000,76000,77000,78000,79000,80000,81000,82000,83000,84000,85000,86000,87000,88000,89000,90000,91000,92000,93000,94000
NW_003726229_1,Count,2,0,0,0,0,0,0,0,0,0,0,0,0,0,0,0,0,0,0,0,0,0,0,0,0,0,0,0,0,0,0,0,0,0,0,0,0,0,0,0,0,0,0,0,0,0,0,0,0,0,0,0,0,0,0,0,0,0,0,0,0,0,0,0,0,0,0,0,0,0,0,0,0,0,0,0,0,0,0,0,0,0,0,0,0,0,0,0,0,0,0,0,0,0,0

	
```

```
		  

		NW_003726230_1, Position,0,1000,2000,3000,4000,5000,6000,7000,8000,9000,10000,11000,12000,13000,14000,15000,16000,17000,18000,19000,20000,21000,22000,23000,24000,25000,26000,27000,28000,29000,30000,31000,32000,33000,34000,35000,36000,37000,38000,39000,40000,41000,42000,43000,44000,45000,46000,47000,48000,49000,50000,51000,52000,53000,54000,55000,56000,57000,58000,59000,60000,61000,62000,63000,64000,65000,66000,67000,68000,69000,70000,71000,72000,73000,74000,75000,76000,77000,78000,79000,80000,81000,82000,83000,84000,85000,86000,87000,88000,89000,90000,91000,92000,93000,94000,95000,96000,97000,98000,99000,100000,101000,102000
NW_003726230_1,Count,9,4,6,8,3,8,7,9,8,5,3,5,6,2,0,5,3,5,7,9,7,4,8,12,4,2,4,2,4,8,6,5,9,7,2,8,8,5,4,6,3,8,5,3,5,7,0,0,0,0,0,4,4,0,6,3,0,0,0,0,0,2,1,5,5,5,4,5,5,0,0,0,0,0,0,0,0,4,2,4,13,4,3,4,7,3,4,3,9,1,6,8,2,1,0,4,8,9,2,6,7,5,6

	
```

```
		  

		NW_003726231_1, Position,0,1000,2000,3000,4000,5000,6000,7000,8000,9000,10000,11000,12000,13000,14000,15000,16000,17000,18000,19000,20000,21000,22000,23000,24000,25000,26000,27000,28000,29000,30000,31000,32000,33000,34000,35000,36000,37000,38000,39000,40000,41000,42000,43000,44000,45000,46000,47000,48000,49000,50000,51000,52000,53000,54000,55000,56000,57000,58000,59000,60000,61000,62000,63000,64000,65000,66000,67000,68000,69000,70000,71000,72000,73000,74000,75000,76000,77000,78000,79000,80000,81000,82000,83000,84000,85000,86000,87000,88000,89000,90000,91000,92000,93000,94000,95000
NW_003726231_1,Count,0,1,3,4,8,0,3,0,0,2,10,7,1,4,9,0,2,1,0,5,2,6,4,0,3,1,9,4,0,0,0,1,1,0,0,0,3,0,3,0,0,0,0,0,0,0,0,6,4,5,4,0,0,2,0,4,0,0,0,0,0,0,0,0,2,3,1,0,0,0,0,3,0,3,0,3,0,8,5,0,0,0,0,2,6,6,0,0,0,3,0,3,8,3,0,1

	
```

```
		  

		NW_003726236_1, Position,0,1000,2000,3000,4000,5000,6000,7000,8000,9000,10000,11000,12000,13000,14000,15000,16000,17000,18000,19000,20000,21000,22000,23000,24000,25000,26000,27000,28000,29000,30000,31000,32000,33000,34000,35000,36000,37000,38000,39000,40000,41000,42000,43000,44000,45000,46000,47000,48000,49000,50000,51000,52000,53000,54000,55000,56000,57000,58000,59000,60000,61000,62000,63000,64000,65000,66000,67000,68000,69000,70000,71000,72000,73000,74000,75000,76000,77000,78000,79000,80000,81000,82000,83000,84000,85000
NW_003726236_1,Count,7,6,6,5,5,6,10,17,5,12,8,7,4,7,6,9,9,9,8,12,12,12,4,10,9,7,14,14,13,13,13,14,7,8,5,10,8,11,6,14,9,6,11,8,0,0,1,0,0,0,2,9,5,8,3,9,11,4,6,4,13,6,6,8,4,4,4,7,9,7,3,9,8,5,9,14,11,9,7,3,15,11,7,9,15,0

	
```

```
		  

		NW_003726237_1, Position,0,1000,2000,3000,4000,5000,6000,7000,8000,9000,10000,11000,12000,13000,14000,15000,16000,17000,18000,19000,20000,21000,22000,23000,24000,25000,26000,27000,28000,29000,30000,31000,32000,33000,34000,35000,36000,37000,38000,39000,40000,41000,42000,43000,44000,45000,46000,47000,48000,49000,50000,51000,52000,53000,54000,55000,56000,57000,58000,59000,60000,61000,62000,63000,64000,65000,66000,67000,68000,69000,70000,71000,72000,73000,74000,75000,76000,77000,78000,79000,80000,81000,82000,83000,84000,85000,86000,87000,88000,89000,90000,91000,92000,93000,94000,95000,96000,97000,98000,99000,100000,101000,102000,103000,104000,105000,106000,107000,108000,109000,110000
NW_003726237_1,Count,0,0,0,0,0,0,0,0,0,0,0,0,0,0,0,0,0,0,0,0,0,0,0,0,0,0,0,0,0,0,0,0,0,0,0,0,0,0,0,0,0,0,0,0,0,0,0,0,0,0,0,0,0,0,0,0,0,0,0,0,0,0,0,0,1,0,0,0,0,0,0,0,0,0,0,0,0,0,0,0,0,0,0,0,0,0,0,0,0,0,0,0,0,0,0,0,0,0,0,0,0,0,0,0,0,0,0,0,0,0,0

	
```

```
		  

		NW_003726239_1, Position,0,1000,2000,3000,4000,5000,6000,7000,8000,9000,10000,11000,12000,13000,14000,15000,16000,17000,18000,19000,20000,21000,22000,23000,24000,25000,26000,27000,28000,29000,30000,31000,32000,33000,34000,35000,36000,37000,38000,39000,40000,41000,42000,43000,44000,45000,46000,47000,48000,49000,50000,51000,52000,53000,54000,55000,56000,57000,58000,59000,60000,61000,62000,63000,64000,65000,66000,67000,68000,69000,70000,71000,72000,73000,74000,75000,76000,77000,78000,79000,80000,81000,82000
NW_003726239_1,Count,10,8,8,10,4,3,3,8,6,4,5,4,8,0,2,0,0,0,0,3,3,3,3,2,0,7,6,4,9,4,8,4,6,7,3,7,4,5,3,7,3,4,2,6,8,7,10,3,8,5,7,4,4,5,4,8,6,1,4,0,0,3,1,0,4,0,0,0,0,6,10,2,3,6,7,6,5,8,7,15,6,7,0

	
```

```
		  

		NW_003726241_1, Position,0,1000,2000,3000,4000,5000,6000,7000,8000,9000,10000,11000,12000,13000,14000,15000,16000,17000,18000,19000,20000,21000,22000,23000,24000,25000,26000,27000,28000,29000,30000,31000,32000,33000,34000,35000,36000,37000,38000,39000,40000,41000,42000,43000,44000,45000,46000,47000,48000,49000,50000,51000,52000,53000,54000,55000,56000,57000,58000,59000,60000,61000,62000,63000,64000,65000,66000,67000,68000,69000,70000,71000,72000,73000,74000,75000,76000,77000,78000,79000,80000,81000,82000
NW_003726241_1,Count,0,9,1,0,0,0,1,0,4,0,0,0,0,0,5,3,0,0,4,0,8,1,1,2,0,0,5,0,3,0,4,0,0,4,0,0,0,0,0,0,0,0,0,0,3,0,0,0,0,0,0,0,0,0,0,0,0,0,0,3,0,0,0,0,0,0,0,0,0,0,0,0,0,0,0,0,0,0,0,0,0,0,0

	
```

```
		  

		NW_003726242_1, Position,0,1000,2000,3000,4000,5000,6000,7000,8000,9000,10000,11000,12000,13000,14000,15000,16000,17000,18000,19000,20000,21000,22000,23000,24000,25000,26000,27000,28000,29000,30000,31000,32000,33000,34000,35000,36000,37000,38000,39000,40000,41000,42000,43000,44000,45000,46000,47000,48000,49000,50000,51000,52000,53000,54000,55000,56000,57000,58000,59000,60000,61000,62000,63000,64000,65000,66000,67000,68000,69000,70000,71000,72000,73000,74000,75000,76000,77000,78000,79000,80000,81000,82000,83000,84000,85000,86000,87000,88000,89000,90000,91000,92000,93000,94000,95000,96000,97000,98000,99000,100000,101000,102000
NW_003726242_1,Count,0,0,0,0,0,0,0,0,0,0,0,0,0,0,0,0,0,0,0,0,0,0,0,0,0,0,0,0,0,0,0,0,0,0,0,0,0,0,0,0,0,0,0,0,0,0,0,0,0,0,0,0,0,0,0,0,0,0,0,0,0,0,0,0,4,0,0,0,0,0,0,0,0,0,0,1,0,0,0,0,0,0,0,0,0,0,0,2,0,0,0,0,0,0,0,0,0,0,0,0,0,0,0

	
```

```
		  

		NW_003726244_1, Position,0,1000,2000,3000,4000,5000,6000,7000,8000,9000,10000,11000,12000,13000,14000,15000,16000,17000,18000,19000,20000,21000,22000,23000,24000,25000,26000,27000,28000,29000,30000,31000,32000,33000,34000,35000,36000,37000,38000,39000,40000,41000,42000,43000,44000,45000,46000,47000,48000,49000,50000,51000,52000,53000,54000,55000,56000,57000,58000,59000,60000,61000,62000,63000,64000,65000,66000,67000,68000,69000,70000,71000,72000,73000,74000,75000,76000,77000,78000,79000,80000,81000,82000,83000,84000,85000,86000,87000
NW_003726244_1,Count,0,0,0,0,0,0,0,0,0,1,0,0,0,0,0,0,0,0,0,0,0,0,0,0,0,0,0,0,0,0,0,0,0,0,0,0,0,0,0,0,0,0,0,0,0,0,0,0,0,0,0,0,0,0,0,0,0,0,0,0,0,0,0,0,0,0,0,0,0,0,0,0,0,0,0,0,0,0,0,0,0,0,0,0,2,0,0,0

	
```

```
		  

		NW_003726246_1, Position,0,1000,2000,3000,4000,5000,6000,7000,8000,9000,10000,11000,12000,13000,14000,15000,16000,17000,18000,19000,20000,21000,22000,23000,24000,25000,26000,27000,28000,29000,30000,31000,32000,33000,34000,35000,36000,37000,38000,39000,40000,41000,42000,43000,44000,45000,46000,47000,48000,49000,50000,51000,52000,53000,54000,55000,56000,57000,58000,59000,60000,61000,62000,63000,64000,65000,66000,67000,68000,69000,70000,71000,72000,73000,74000,75000,76000,77000,78000,79000,80000,81000,82000,83000,84000,85000,86000,87000,88000,89000,90000,91000,92000,93000,94000,95000,96000,97000,98000,99000,100000,101000,102000,103000,104000,105000,106000,107000,108000,109000,110000,111000,112000,113000,114000,115000,116000,117000,118000,119000,120000,121000,122000,123000,124000,125000,126000,127000,128000,129000,130000,131000,132000,133000,134000,135000,136000,137000,138000,139000,140000,141000,142000,143000,144000,145000,146000,147000,148000,149000,150000,151000,152000,153000,154000,155000,156000,157000,158000,159000,160000,161000,162000,163000,164000,165000,166000,167000,168000,169000,170000,171000,172000,173000,174000,175000,176000,177000,178000,179000,180000,181000,182000,183000,184000,185000,186000,187000,188000,189000,190000,191000,192000,193000,194000,195000,196000,197000,198000,199000,200000,201000,202000,203000,204000,205000,206000,207000,208000,209000,210000,211000,212000,213000,214000,215000,216000,217000,218000,219000,220000,221000,222000,223000,224000,225000,226000,227000,228000,229000,230000
NW_003726246_1,Count,0,0,0,0,0,0,0,0,0,0,0,0,0,0,0,0,0,0,0,0,0,0,0,0,0,0,0,0,0,0,0,0,0,0,0,0,0,0,0,0,0,0,0,0,0,0,0,0,0,0,0,0,0,0,0,0,0,0,0,0,0,0,0,0,0,0,0,0,0,0,0,0,0,0,0,0,0,0,0,0,0,0,0,0,0,0,0,0,0,0,0,0,0,0,0,0,0,0,0,0,0,0,0,0,0,0,0,0,0,0,0,0,0,0,0,0,0,0,0,0,0,0,0,0,0,0,0,0,0,0,0,0,0,0,0,0,0,0,0,0,0,0,0,0,0,0,0,0,0,0,0,0,2,5,0,1,3,0,7,0,2,0,0,2,7,3,2,0,1,2,6,5,2,1,4,2,2,4,6,2,3,8,2,2,6,5,5,2,3,8,2,5,4,2,5,7,3,3,7,3,5,6,0,3,6,9,6,4,2,9,0,1,1,6,1,3,2,1,8,2,2,3,4,7,2,0,0,0,0,0,0

	
```

```
		  

		NW_003726247_1, Position,0,1000,2000,3000,4000,5000,6000,7000,8000,9000,10000,11000,12000,13000,14000,15000,16000,17000,18000,19000,20000,21000,22000,23000,24000,25000,26000,27000,28000,29000,30000,31000,32000,33000,34000,35000,36000,37000,38000,39000,40000,41000,42000,43000,44000,45000,46000,47000,48000,49000,50000,51000,52000,53000,54000,55000,56000,57000,58000,59000,60000,61000,62000,63000,64000,65000,66000,67000,68000,69000,70000,71000,72000,73000,74000,75000,76000,77000,78000
NW_003726247_1,Count,0,0,0,0,0,0,0,0,0,0,0,0,0,0,0,0,0,0,0,0,0,0,0,0,0,0,0,0,0,0,0,0,0,0,0,0,0,0,0,0,0,0,0,0,0,0,0,0,0,0,0,0,0,0,0,0,0,0,0,0,0,0,0,0,0,0,0,0,0,0,3,0,0,0,0,0,0,0,0

	
```

```
		  

		NW_003726248_1, Position,0,1000,2000,3000,4000,5000,6000,7000,8000,9000,10000,11000,12000,13000,14000,15000,16000,17000,18000,19000,20000,21000,22000,23000,24000,25000,26000,27000,28000,29000,30000,31000,32000,33000,34000,35000,36000,37000,38000,39000,40000,41000,42000,43000,44000,45000,46000,47000,48000,49000,50000,51000,52000,53000,54000,55000,56000,57000,58000,59000,60000,61000,62000,63000,64000,65000,66000,67000,68000,69000,70000,71000,72000,73000,74000,75000,76000,77000,78000,79000,80000,81000,82000,83000,84000,85000,86000,87000,88000,89000,90000,91000,92000,93000,94000,95000,96000,97000,98000,99000,100000,101000,102000,103000,104000,105000,106000,107000,108000,109000,110000,111000,112000,113000,114000,115000,116000,117000,118000,119000,120000,121000,122000,123000,124000,125000,126000,127000,128000,129000,130000,131000,132000,133000,134000,135000,136000,137000,138000,139000,140000,141000,142000,143000,144000,145000,146000,147000,148000,149000,150000,151000,152000,153000,154000,155000,156000,157000,158000,159000,160000,161000,162000,163000,164000,165000,166000,167000,168000,169000,170000,171000,172000,173000,174000,175000,176000,177000,178000,179000,180000,181000,182000,183000,184000,185000,186000,187000,188000,189000,190000,191000,192000,193000,194000,195000,196000,197000,198000,199000,200000,201000,202000,203000,204000,205000,206000,207000,208000,209000,210000,211000,212000,213000,214000,215000,216000,217000,218000,219000,220000,221000,222000,223000,224000,225000,226000,227000,228000,229000,230000,231000,232000,233000,234000,235000,236000,237000,238000,239000,240000,241000,242000,243000,244000,245000,246000,247000,248000,249000,250000,251000,252000,253000,254000,255000,256000
NW_003726248_1,Count,11,10,11,3,13,6,10,6,5,15,6,3,17,11,8,8,9,7,9,10,4,10,7,8,5,7,6,12,12,8,7,10,8,7,9,5,5,5,8,2,11,10,6,2,6,0,5,9,7,5,1,3,3,4,2,6,3,4,5,4,2,5,2,3,2,3,0,0,0,0,0,0,0,0,0,0,0,0,0,0,0,0,0,0,0,0,0,0,0,0,0,0,0,0,0,0,0,0,0,0,0,0,0,0,0,0,0,0,0,0,0,0,0,0,0,0,0,0,0,0,0,0,0,0,0,0,0,0,0,0,0,0,0,0,0,0,0,0,0,0,0,0,0,0,0,0,0,0,0,0,0,0,0,0,0,0,0,0,0,0,0,0,0,0,0,0,0,0,0,0,0,0,0,0,0,0,0,0,0,0,0,0,0,0,0,0,0,0,0,0,0,0,0,0,0,0,0,0,0,0,0,0,0,0,0,0,0,0,0,0,0,0,0,0,0,0,0,0,0,0,0,0,0,0,0,0,0,0,0,0,0,0,0,0,0,0,0,0,0,0,0,0,0,0,0,0,0,0,0,2,2,0,0,0,0,1,0

	
```

```
		  

		NW_003726251_1, Position,0,1000,2000,3000,4000,5000,6000,7000,8000,9000,10000,11000,12000,13000,14000,15000,16000,17000,18000,19000,20000,21000,22000,23000,24000,25000,26000,27000,28000,29000,30000,31000,32000,33000,34000,35000,36000,37000,38000,39000,40000,41000,42000,43000,44000,45000,46000,47000,48000,49000,50000,51000,52000,53000,54000,55000,56000,57000,58000,59000,60000,61000,62000,63000,64000,65000,66000,67000,68000,69000,70000,71000,72000,73000,74000,75000,76000,77000
NW_003726251_1,Count,0,0,0,0,0,0,0,0,0,0,0,0,0,0,0,0,0,0,0,0,0,0,0,0,0,0,0,0,0,0,0,0,0,0,0,0,0,0,0,0,0,0,0,0,0,0,0,0,0,0,3,0,0,0,0,0,0,2,0,0,0,0,0,0,0,0,0,0,0,0,0,0,0,0,0,0,0,0

	
```

```
		  

		NW_003726252_1, Position,0,1000,2000,3000,4000,5000,6000,7000,8000,9000,10000,11000,12000,13000,14000,15000,16000,17000,18000,19000,20000,21000,22000,23000,24000,25000,26000,27000,28000,29000,30000,31000,32000,33000,34000,35000,36000,37000,38000,39000,40000,41000,42000,43000,44000,45000,46000,47000,48000,49000,50000,51000,52000,53000,54000,55000,56000,57000,58000,59000,60000,61000,62000,63000,64000,65000,66000,67000,68000,69000,70000,71000,72000,73000,74000,75000,76000,77000
NW_003726252_1,Count,0,0,6,5,0,1,4,6,0,3,3,8,11,7,15,9,0,0,11,0,3,0,0,0,0,1,0,0,0,0,0,0,0,0,0,0,0,0,0,0,0,0,0,0,0,0,0,0,0,0,2,2,0,0,0,0,0,3,0,0,0,2,0,0,0,0,0,0,0,0,0,5,5,0,0,0,0,0

	
```

```
		  

		NW_003726253_1, Position,0,1000,2000,3000,4000,5000,6000,7000,8000,9000,10000,11000,12000,13000,14000,15000,16000,17000,18000,19000,20000,21000,22000,23000,24000,25000,26000,27000,28000,29000,30000,31000,32000,33000,34000,35000,36000,37000,38000,39000,40000,41000,42000,43000,44000,45000,46000,47000,48000,49000,50000,51000,52000,53000,54000,55000,56000,57000,58000,59000,60000,61000,62000,63000,64000,65000,66000,67000,68000,69000,70000,71000,72000,73000,74000,75000,76000,77000,78000,79000
NW_003726253_1,Count,3,8,4,5,11,2,0,0,0,0,0,0,3,3,0,0,0,0,0,0,0,0,0,0,0,0,0,0,1,0,0,0,1,1,0,4,2,4,3,3,5,7,7,5,5,0,7,0,0,0,0,0,0,0,0,5,0,0,4,0,0,0,0,0,0,0,0,0,0,0,0,0,0,0,0,0,0,0,0,0

	
```

```
		  

		NW_003726254_1, Position,0,1000,2000,3000,4000,5000,6000,7000,8000,9000,10000,11000,12000,13000,14000,15000,16000,17000,18000,19000,20000,21000,22000,23000,24000,25000,26000,27000,28000,29000,30000,31000,32000,33000,34000,35000,36000,37000,38000,39000,40000,41000,42000,43000,44000,45000,46000,47000,48000,49000,50000,51000,52000,53000,54000,55000,56000,57000,58000,59000,60000,61000,62000,63000,64000,65000,66000,67000,68000,69000,70000,71000,72000,73000,74000,75000,76000,77000,78000,79000,80000,81000,82000,83000,84000,85000,86000,87000,88000,89000,90000
NW_003726254_1,Count,0,0,0,0,0,0,0,0,0,0,0,0,1,0,0,0,0,0,0,0,0,0,0,0,0,0,0,0,0,0,0,0,0,0,0,0,0,0,0,0,0,2,0,0,0,0,0,0,0,0,0,0,0,0,0,0,0,0,0,0,0,0,0,0,0,0,0,0,0,0,0,0,0,0,0,0,0,0,0,0,0,0,0,0,0,0,0,0,0,0,0

	
```

```
		  

		NW_003726257_1, Position,0,1000,2000,3000,4000,5000,6000,7000,8000,9000,10000,11000,12000,13000,14000,15000,16000,17000,18000,19000,20000,21000,22000,23000,24000,25000,26000,27000,28000,29000,30000,31000,32000,33000,34000,35000,36000,37000,38000,39000,40000,41000,42000,43000,44000,45000,46000,47000,48000,49000,50000,51000,52000,53000,54000,55000,56000,57000,58000,59000,60000,61000,62000,63000,64000,65000,66000,67000,68000,69000,70000,71000,72000,73000,74000,75000,76000,77000,78000,79000,80000,81000,82000,83000,84000,85000,86000,87000,88000,89000,90000,91000,92000,93000,94000,95000,96000,97000
NW_003726257_1,Count,4,1,7,7,6,2,8,2,3,6,3,4,7,4,5,6,2,2,8,5,4,8,1,0,0,0,0,0,0,0,0,0,0,0,0,0,0,0,0,0,0,0,0,0,0,0,10,8,10,1,4,6,4,2,3,2,4,9,3,4,3,4,2,3,5,3,6,3,3,8,4,5,5,4,1,1,5,7,4,4,3,3,7,0,2,5,9,4,4,5,2,1,0,0,0,1,0,0

	
```

```
		  

		NW_003726258_1, Position,0,1000,2000,3000,4000,5000,6000,7000,8000,9000,10000,11000,12000,13000,14000,15000,16000,17000,18000,19000,20000,21000,22000,23000,24000,25000,26000,27000,28000,29000,30000,31000,32000,33000,34000,35000,36000,37000,38000,39000,40000,41000,42000,43000,44000,45000,46000,47000,48000,49000,50000,51000,52000,53000,54000,55000,56000,57000,58000,59000,60000,61000,62000,63000,64000,65000,66000,67000,68000,69000,70000,71000,72000,73000,74000,75000,76000,77000,78000,79000
NW_003726258_1,Count,11,19,16,19,13,15,13,15,9,17,13,17,6,14,12,11,18,12,2,2,12,12,2,0,3,15,10,17,13,10,16,12,8,9,11,8,9,17,5,9,18,16,8,17,14,17,7,6,12,20,15,9,7,3,0,7,17,15,11,7,8,18,11,14,13,9,14,10,3,0,13,17,15,14,3,14,19,13,13,10

	
```

```
		  

		NW_003726260_1, Position,0,1000,2000,3000,4000,5000,6000,7000,8000,9000,10000,11000,12000,13000,14000,15000,16000,17000,18000,19000,20000,21000,22000,23000,24000,25000,26000,27000,28000,29000,30000,31000,32000,33000,34000,35000,36000,37000,38000,39000,40000,41000,42000,43000,44000,45000,46000,47000,48000,49000,50000,51000,52000,53000,54000,55000,56000,57000,58000,59000,60000,61000,62000,63000,64000,65000,66000,67000,68000,69000,70000,71000,72000,73000
NW_003726260_1,Count,0,0,0,0,0,0,0,0,0,0,0,0,0,0,0,0,0,0,0,3,0,0,0,0,0,1,0,0,0,0,0,0,0,0,0,0,0,0,0,0,0,0,0,0,0,0,0,1,0,2,0,0,5,0,0,0,0,0,0,0,0,0,0,0,0,5,1,0,0,0,4,2,1,0

	
```

```
		  

		NW_003726261_1, Position,0,1000,2000,3000,4000,5000,6000,7000,8000,9000,10000,11000,12000,13000,14000,15000,16000,17000,18000,19000,20000,21000,22000,23000,24000,25000,26000,27000,28000,29000,30000,31000,32000,33000,34000,35000,36000,37000,38000,39000,40000,41000,42000,43000,44000,45000,46000,47000,48000,49000,50000,51000,52000,53000,54000,55000,56000,57000,58000,59000,60000,61000,62000,63000,64000,65000,66000,67000,68000,69000,70000,71000,72000,73000,74000,75000,76000,77000,78000,79000,80000,81000,82000,83000,84000,85000,86000,87000,88000,89000,90000,91000,92000,93000,94000,95000,96000,97000
NW_003726261_1,Count,0,1,0,0,0,0,0,0,0,0,0,0,0,0,0,0,0,0,0,0,0,0,0,0,0,0,0,0,0,0,0,0,0,0,0,0,0,0,0,0,0,0,0,0,0,0,0,0,0,0,0,0,0,0,0,0,0,0,0,0,0,0,0,0,0,0,0,0,0,0,0,0,0,0,0,0,0,0,0,0,0,0,0,0,0,0,0,0,0,0,0,0,0,0,0,0,0,0

	
```

```
		  

		NW_003726262_1, Position,0,1000,2000,3000,4000,5000,6000,7000,8000,9000,10000,11000,12000,13000,14000,15000,16000,17000,18000,19000,20000,21000,22000,23000,24000,25000,26000,27000,28000,29000,30000,31000,32000,33000,34000,35000,36000,37000,38000,39000,40000,41000,42000,43000,44000,45000,46000,47000,48000,49000,50000,51000,52000,53000,54000,55000,56000,57000,58000,59000,60000,61000,62000,63000,64000,65000,66000,67000,68000,69000,70000,71000,72000,73000
NW_003726262_1,Count,0,0,0,4,0,0,0,0,0,0,4,0,0,0,0,0,0,3,3,0,0,2,2,2,0,1,0,0,1,0,0,0,0,0,0,0,0,0,0,0,0,0,0,7,0,0,0,0,3,0,0,0,0,0,0,0,0,0,0,0,0,0,0,1,0,7,10,3,0,0,0,0,5,0

	
```

```
		  

		NW_003726263_1, Position,0,1000,2000,3000,4000,5000,6000,7000,8000,9000,10000,11000,12000,13000,14000,15000,16000,17000,18000,19000,20000,21000,22000,23000,24000,25000,26000,27000,28000,29000,30000,31000,32000,33000,34000,35000,36000,37000,38000,39000,40000,41000,42000,43000,44000,45000,46000,47000,48000,49000,50000,51000,52000,53000,54000,55000,56000,57000,58000,59000,60000,61000,62000,63000,64000,65000,66000,67000,68000,69000,70000,71000,72000,73000,74000,75000,76000,77000,78000,79000,80000,81000,82000,83000,84000,85000,86000,87000,88000,89000,90000,91000,92000,93000,94000,95000,96000,97000,98000,99000,100000,101000,102000,103000,104000
NW_003726263_1,Count,6,0,0,0,0,0,0,0,0,0,0,0,0,0,0,0,0,0,0,0,0,0,0,0,0,0,0,0,0,0,0,0,0,0,0,0,0,0,0,0,0,0,0,0,0,0,0,0,0,0,0,0,0,0,0,0,0,0,0,0,0,0,0,0,0,0,0,0,0,0,0,0,0,0,0,0,0,0,0,0,0,0,0,0,0,0,0,0,0,0,0,0,0,0,0,0,0,0,0,0,0,0,0,0,0

	
```

```
		  

		NW_003726264_1, Position,0,1000,2000,3000,4000,5000,6000,7000,8000,9000,10000,11000,12000,13000,14000,15000,16000,17000,18000,19000,20000,21000,22000,23000,24000,25000,26000,27000,28000,29000,30000,31000,32000,33000,34000,35000,36000,37000,38000,39000,40000,41000,42000,43000,44000,45000,46000,47000,48000,49000,50000,51000,52000,53000,54000,55000,56000,57000,58000,59000,60000,61000,62000,63000,64000,65000,66000,67000,68000,69000,70000,71000,72000,73000,74000,75000,76000,77000,78000
NW_003726264_1,Count,12,12,14,10,11,15,9,12,16,12,14,8,16,11,7,4,18,15,6,18,1,0,0,0,5,10,12,11,13,14,17,11,11,6,2,14,17,15,16,0,12,13,5,1,16,8,15,6,17,9,18,15,15,14,12,12,11,12,9,7,10,12,12,19,14,14,10,6,15,14,14,3,4,14,12,15,14,22,14

	
```

```
		  

		NW_003726266_1, Position,0,1000,2000,3000,4000,5000,6000,7000,8000,9000,10000,11000,12000,13000,14000,15000,16000,17000,18000,19000,20000,21000,22000,23000,24000,25000,26000,27000,28000,29000,30000,31000,32000,33000,34000,35000,36000,37000,38000,39000,40000,41000,42000,43000,44000,45000,46000,47000,48000,49000,50000,51000,52000,53000,54000,55000,56000,57000,58000,59000,60000,61000,62000,63000,64000,65000,66000,67000,68000
NW_003726266_1,Count,0,0,0,0,0,0,0,0,0,0,0,0,0,0,0,0,0,0,0,0,0,0,0,0,0,0,0,0,0,0,0,0,0,0,0,1,0,0,0,0,0,0,0,0,0,0,0,0,0,0,0,0,0,0,0,0,0,0,0,0,0,0,0,0,0,0,0,0,0

	
```

```
		  

		NW_003726268_1, Position,0,1000,2000,3000,4000,5000,6000,7000,8000,9000,10000,11000,12000,13000,14000,15000,16000,17000,18000,19000,20000,21000,22000,23000,24000,25000,26000,27000,28000,29000,30000,31000,32000,33000,34000,35000,36000,37000,38000,39000,40000,41000,42000,43000,44000,45000,46000,47000,48000,49000,50000,51000,52000,53000,54000,55000,56000,57000,58000,59000,60000,61000,62000,63000,64000,65000,66000,67000,68000
NW_003726268_1,Count,5,8,11,12,12,11,13,12,12,11,4,9,6,9,8,16,6,8,7,9,7,9,10,13,11,14,13,10,13,13,12,15,12,12,15,13,8,9,8,4,9,10,7,6,19,11,15,14,17,8,14,10,5,7,7,8,9,6,3,10,15,13,11,7,10,12,10,7,0

	
```

```
		  

		NW_003726273_1, Position,0,1000,2000,3000,4000,5000,6000,7000,8000,9000,10000,11000,12000,13000,14000,15000,16000,17000,18000,19000,20000,21000,22000,23000,24000,25000,26000,27000,28000,29000,30000,31000,32000,33000,34000,35000,36000,37000,38000,39000,40000,41000,42000,43000,44000,45000,46000,47000,48000,49000,50000,51000,52000,53000,54000,55000,56000,57000,58000,59000,60000,61000,62000,63000,64000,65000,66000,67000
NW_003726273_1,Count,3,8,7,1,10,6,8,4,6,5,6,8,4,5,6,6,7,1,4,6,3,6,12,13,5,5,8,2,6,5,8,3,6,1,1,4,3,2,8,2,7,7,3,7,3,7,8,6,5,3,6,7,7,9,5,8,9,8,9,5,5,2,3,7,8,4,6,2

	
```

```
		  

		NW_003726275_1, Position,0,1000,2000,3000,4000,5000,6000,7000,8000,9000,10000,11000,12000,13000,14000,15000,16000,17000,18000,19000,20000,21000,22000,23000,24000,25000,26000,27000,28000,29000,30000,31000,32000,33000,34000,35000,36000,37000,38000,39000,40000,41000,42000,43000,44000,45000,46000,47000,48000,49000,50000,51000,52000,53000,54000,55000,56000,57000,58000,59000,60000,61000,62000,63000,64000,65000,66000,67000
NW_003726275_1,Count,0,0,0,0,0,0,0,0,0,0,0,1,2,0,0,0,0,0,0,0,0,0,0,0,0,0,0,0,0,0,0,0,0,2,0,0,0,0,0,2,4,0,0,6,0,0,0,0,0,0,8,0,0,0,0,4,1,7,0,5,5,1,0,1,0,0,0,0

	
```

```
		  

		NW_003726280_1, Position,0,1000,2000,3000,4000,5000,6000,7000,8000,9000,10000,11000,12000,13000,14000,15000,16000,17000,18000,19000,20000,21000,22000,23000,24000,25000,26000,27000,28000,29000,30000,31000,32000,33000,34000,35000,36000,37000,38000,39000,40000,41000,42000,43000,44000,45000,46000,47000,48000,49000,50000,51000,52000,53000,54000,55000,56000,57000,58000,59000,60000,61000,62000,63000,64000,65000
NW_003726280_1,Count,0,0,0,0,0,0,0,0,0,0,0,0,0,0,0,0,0,0,0,0,0,5,0,0,1,6,0,0,0,0,0,0,0,0,0,0,1,14,3,5,0,0,3,0,0,0,0,1,0,0,0,0,0,0,7,0,0,0,4,0,9,3,8,0,1,0

	
```

```
		  

		NW_003726283_1, Position,0,1000,2000,3000,4000,5000,6000,7000,8000,9000,10000,11000,12000,13000,14000,15000,16000,17000,18000,19000,20000,21000,22000,23000,24000,25000,26000,27000,28000,29000,30000,31000,32000,33000,34000,35000,36000,37000,38000,39000,40000,41000,42000,43000,44000,45000,46000,47000,48000,49000,50000,51000,52000,53000,54000,55000,56000,57000,58000,59000,60000,61000,62000,63000,64000,65000,66000,67000,68000,69000,70000,71000,72000,73000,74000,75000,76000,77000,78000,79000,80000,81000,82000,83000,84000,85000,86000,87000,88000
NW_003726283_1,Count,0,0,0,0,0,0,0,0,0,0,0,0,0,0,0,0,0,0,0,0,0,0,0,0,0,0,0,0,0,0,0,0,0,0,0,0,0,0,0,0,0,0,0,0,0,0,0,0,0,0,0,0,0,0,0,0,0,0,0,0,0,0,0,0,1,0,0,0,0,0,0,0,0,0,0,0,0,0,0,0,0,0,0,0,0,0,0,0,0

	
```

```
		  

		NW_003726285_1, Position,0,1000,2000,3000,4000,5000,6000,7000,8000,9000,10000,11000,12000,13000,14000,15000,16000,17000,18000,19000,20000,21000,22000,23000,24000,25000,26000,27000,28000,29000,30000,31000,32000,33000,34000,35000,36000,37000,38000,39000,40000,41000,42000,43000,44000,45000,46000,47000,48000,49000,50000,51000,52000,53000,54000,55000,56000,57000,58000,59000,60000,61000,62000,63000,64000,65000,66000,67000,68000,69000,70000
NW_003726285_1,Count,0,0,0,0,0,0,0,0,2,0,0,0,0,0,0,0,0,0,0,0,0,0,0,0,0,3,6,5,5,8,9,6,4,6,11,10,14,14,0,6,5,6,2,4,10,5,10,5,6,4,3,15,6,9,9,11,5,8,9,8,3,6,4,9,11,9,3,0,0,0,0

	
```

```
		  

		NW_003726287_1, Position,0,1000,2000,3000,4000,5000,6000,7000,8000,9000,10000,11000,12000,13000,14000,15000,16000,17000,18000,19000,20000,21000,22000,23000,24000,25000,26000,27000,28000,29000,30000,31000,32000,33000,34000,35000,36000,37000,38000,39000,40000,41000,42000,43000,44000,45000,46000,47000,48000,49000,50000,51000,52000,53000,54000,55000,56000,57000,58000,59000,60000,61000,62000,63000,64000,65000,66000,67000,68000,69000
NW_003726287_1,Count,0,0,0,0,0,0,0,0,0,0,0,0,0,0,0,0,0,0,0,0,0,0,0,0,0,0,0,0,0,0,0,0,0,0,1,0,4,2,1,0,0,5,0,0,0,0,0,0,0,0,0,0,0,0,0,0,4,3,0,6,0,0,0,0,0,0,0,0,0,0

	
```

```
		  

		NW_003726289_1, Position,0,1000,2000,3000,4000,5000,6000,7000,8000,9000,10000,11000,12000,13000,14000,15000,16000,17000,18000,19000,20000,21000,22000,23000,24000,25000,26000,27000,28000,29000,30000,31000,32000,33000,34000,35000,36000,37000,38000,39000,40000,41000,42000,43000,44000,45000,46000,47000,48000,49000,50000,51000,52000,53000,54000,55000,56000,57000,58000,59000,60000,61000,62000,63000,64000
NW_003726289_1,Count,0,0,0,8,15,8,12,9,14,8,0,1,0,2,4,0,0,0,0,0,0,0,0,0,10,11,10,6,13,7,4,10,4,9,9,12,15,15,9,7,7,4,11,11,12,6,3,13,13,9,15,20,10,17,10,15,13,19,11,10,14,17,13,6,1

	
```

```
		  

		NW_003726290_1, Position,0,1000,2000,3000,4000,5000,6000,7000,8000,9000,10000,11000,12000,13000,14000,15000,16000,17000,18000,19000,20000,21000,22000,23000,24000,25000,26000,27000,28000,29000,30000,31000,32000,33000,34000,35000,36000,37000,38000,39000,40000,41000,42000,43000,44000,45000,46000,47000,48000,49000,50000,51000,52000,53000,54000,55000,56000,57000,58000,59000,60000,61000
NW_003726290_1,Count,0,0,0,0,0,0,0,0,0,0,0,0,0,0,0,0,0,0,0,0,3,2,3,1,0,0,0,0,0,0,0,0,0,0,0,0,0,0,0,1,0,0,0,0,0,0,0,0,0,0,0,0,0,0,0,0,0,0,0,0,0,0

	
```

```
		  

		NW_003726292_1, Position,0,1000,2000,3000,4000,5000,6000,7000,8000,9000,10000,11000,12000,13000,14000,15000,16000,17000,18000,19000,20000,21000,22000,23000,24000,25000,26000,27000,28000,29000,30000,31000,32000,33000,34000,35000,36000,37000,38000,39000,40000,41000,42000,43000,44000,45000,46000,47000,48000,49000,50000,51000,52000,53000,54000,55000,56000,57000,58000,59000,60000,61000
NW_003726292_1,Count,9,17,12,6,13,12,16,12,9,11,7,10,9,12,9,11,12,8,4,9,9,11,1,11,13,17,15,16,10,8,0,10,5,8,10,13,3,0,12,9,0,0,3,7,0,0,0,0,0,0,0,0,0,0,0,0,0,0,0,0,0,0

	
```

```
		  

		NW_003726293_1, Position,0,1000,2000,3000,4000,5000,6000,7000,8000,9000,10000,11000,12000,13000,14000,15000,16000,17000,18000,19000,20000,21000,22000,23000,24000,25000,26000,27000,28000,29000,30000,31000,32000,33000,34000,35000,36000,37000,38000,39000,40000,41000,42000,43000,44000,45000,46000,47000,48000,49000,50000,51000,52000,53000,54000,55000,56000,57000,58000,59000,60000,61000,62000,63000,64000,65000,66000,67000,68000,69000,70000,71000,72000,73000,74000,75000,76000,77000,78000,79000,80000,81000,82000,83000,84000,85000,86000,87000,88000,89000,90000
NW_003726293_1,Count,0,5,0,0,0,0,0,0,0,0,0,0,0,0,0,0,0,0,0,0,0,0,0,0,0,0,0,0,0,0,0,0,0,0,0,0,0,0,0,0,0,0,0,0,0,0,0,0,0,0,0,0,0,0,0,0,0,0,0,0,0,0,0,0,0,0,0,0,0,0,0,0,0,0,0,0,0,0,0,0,0,0,0,0,0,0,0,0,0,0,0

	
```

```
		  

		NW_003726294_1, Position,0,1000,2000,3000,4000,5000,6000,7000,8000,9000,10000,11000,12000,13000,14000,15000,16000,17000,18000,19000,20000,21000,22000,23000,24000,25000,26000,27000,28000,29000,30000,31000,32000,33000,34000,35000,36000,37000,38000,39000,40000,41000,42000,43000,44000,45000,46000,47000,48000,49000,50000,51000,52000,53000,54000,55000,56000,57000,58000,59000,60000,61000
NW_003726294_1,Count,0,0,0,0,0,0,0,0,0,0,0,0,0,0,0,0,0,0,0,0,0,0,2,0,0,0,0,0,0,0,0,0,0,0,0,0,3,0,4,0,0,0,3,2,0,0,0,0,0,0,0,0,0,0,0,0,0,0,0,0,0,0

	
```

```
		  

		NW_003726295_1, Position,0,1000,2000,3000,4000,5000,6000,7000,8000,9000,10000,11000,12000,13000,14000,15000,16000,17000,18000,19000,20000,21000,22000,23000,24000,25000,26000,27000,28000,29000,30000,31000,32000,33000,34000,35000,36000,37000,38000,39000,40000,41000,42000,43000,44000,45000,46000,47000,48000,49000,50000,51000,52000,53000,54000,55000,56000,57000,58000,59000,60000,61000,62000,63000
NW_003726295_1,Count,0,0,0,0,0,0,0,0,0,0,0,0,0,0,0,0,0,0,0,0,0,0,0,0,0,0,0,3,2,0,0,0,0,0,0,0,0,0,0,0,0,0,0,0,0,0,0,0,0,0,0,0,0,0,0,0,0,0,0,0,0,0,0,0

	
```

```
		  

		NW_003726297_1, Position,0,1000,2000,3000,4000,5000,6000,7000,8000,9000,10000,11000,12000,13000,14000,15000,16000,17000,18000,19000,20000,21000,22000,23000,24000,25000,26000,27000,28000,29000,30000,31000,32000,33000,34000,35000,36000,37000,38000,39000,40000,41000,42000,43000,44000,45000,46000,47000,48000,49000,50000,51000,52000,53000,54000,55000,56000,57000,58000,59000,60000,61000,62000,63000,64000,65000,66000,67000,68000,69000,70000,71000,72000,73000,74000,75000,76000,77000,78000,79000,80000,81000,82000,83000,84000,85000,86000,87000,88000
NW_003726297_1,Count,0,0,0,0,0,0,0,0,0,0,0,2,0,5,0,0,0,0,0,0,0,0,0,0,0,0,0,0,0,0,0,0,0,0,0,0,0,0,0,0,0,0,0,0,0,0,0,0,0,0,0,0,0,0,0,0,0,0,0,0,0,0,0,0,0,0,0,0,0,0,0,0,0,0,3,0,0,0,0,4,1,3,1,3,0,0,2,5,0

	
```

```
		  

		NW_003726300_1, Position,0,1000,2000,3000,4000,5000,6000,7000,8000,9000,10000,11000,12000,13000,14000,15000,16000,17000,18000,19000,20000,21000,22000,23000,24000,25000,26000,27000,28000,29000,30000,31000,32000,33000,34000,35000,36000,37000,38000,39000,40000,41000,42000,43000,44000,45000,46000,47000,48000,49000,50000,51000,52000,53000,54000,55000,56000,57000,58000,59000,60000,61000,62000,63000,64000,65000,66000,67000,68000,69000,70000
NW_003726300_1,Count,0,0,0,0,0,0,1,1,0,0,0,0,0,0,0,0,0,0,0,0,0,0,0,0,0,0,0,0,0,0,0,0,0,0,0,0,0,0,0,0,0,0,0,0,0,0,0,0,0,0,0,0,0,0,0,0,0,0,2,0,1,0,0,0,0,0,2,1,0,0,0

	
```

```
		  

		NW_003726301_1, Position,0,1000,2000,3000,4000,5000,6000,7000,8000,9000,10000,11000,12000,13000,14000,15000,16000,17000,18000,19000,20000,21000,22000,23000,24000,25000,26000,27000,28000,29000,30000,31000,32000,33000,34000,35000,36000,37000,38000,39000,40000,41000,42000,43000,44000,45000,46000,47000,48000,49000,50000,51000,52000,53000,54000,55000,56000,57000,58000,59000,60000,61000,62000,63000,64000,65000,66000,67000,68000,69000,70000,71000
NW_003726301_1,Count,0,0,0,0,0,0,0,3,0,0,0,0,0,0,0,0,0,0,0,0,0,0,0,0,0,0,0,0,0,0,0,0,0,0,0,0,0,0,0,0,0,0,0,0,0,0,0,0,0,0,0,0,0,0,0,0,0,0,0,0,0,0,0,0,0,0,0,0,0,0,0,0

	
```

```
		  

		NW_003726303_1, Position,0,1000,2000,3000,4000,5000,6000,7000,8000,9000,10000,11000,12000,13000,14000,15000,16000,17000,18000,19000,20000,21000,22000,23000,24000,25000,26000,27000,28000,29000,30000,31000,32000,33000,34000,35000,36000,37000,38000,39000,40000,41000,42000,43000,44000,45000,46000,47000,48000,49000,50000,51000,52000,53000,54000,55000,56000,57000,58000,59000
NW_003726303_1,Count,0,0,3,0,0,0,0,0,0,0,0,0,0,0,0,0,0,0,0,0,0,0,0,0,0,0,0,0,0,0,0,0,0,0,0,0,0,0,0,0,0,0,1,0,0,0,0,0,0,0,0,0,0,0,0,0,0,0,0,0

	
```

```
		  

		NW_003726304_1, Position,0,1000,2000,3000,4000,5000,6000,7000,8000,9000,10000,11000,12000,13000,14000,15000,16000,17000,18000,19000,20000,21000,22000,23000,24000,25000,26000,27000,28000,29000,30000,31000,32000,33000,34000,35000,36000,37000,38000,39000,40000,41000,42000,43000,44000,45000,46000,47000,48000,49000,50000,51000,52000,53000,54000,55000,56000,57000,58000,59000,60000,61000,62000,63000,64000,65000,66000
NW_003726304_1,Count,0,0,0,0,0,0,0,0,0,0,0,0,0,0,0,0,0,0,0,0,0,0,0,0,0,3,6,0,0,0,0,0,0,0,0,0,0,0,0,0,0,0,0,0,5,0,0,0,0,3,2,0,0,0,2,0,2,9,8,4,7,6,3,2,4,7,6

	
```

```
		  

		NW_003726305_1, Position,0,1000,2000,3000,4000,5000,6000,7000,8000,9000,10000,11000,12000,13000,14000,15000,16000,17000,18000,19000,20000,21000,22000,23000,24000,25000,26000,27000,28000,29000,30000,31000,32000,33000,34000,35000,36000,37000,38000,39000,40000,41000,42000,43000,44000,45000,46000,47000,48000,49000,50000,51000,52000,53000,54000,55000,56000,57000,58000,59000,60000,61000,62000
NW_003726305_1,Count,0,0,2,0,0,0,1,0,0,0,0,3,0,4,2,1,0,3,11,0,0,1,3,6,0,1,2,0,0,0,3,0,0,0,0,0,0,5,3,0,1,0,0,0,0,0,0,0,0,5,0,3,2,0,0,0,5,0,0,2,5,5,0

	
```

```
		  

		NW_003726306_1, Position,0,1000,2000,3000,4000,5000,6000,7000,8000,9000,10000,11000,12000,13000,14000,15000,16000,17000,18000,19000,20000,21000,22000,23000,24000,25000,26000,27000,28000,29000,30000,31000,32000,33000,34000,35000,36000,37000,38000,39000,40000,41000,42000,43000,44000,45000,46000,47000,48000,49000,50000,51000,52000,53000,54000,55000,56000,57000,58000,59000,60000,61000,62000,63000,64000,65000,66000,67000,68000
NW_003726306_1,Count,0,0,0,0,0,0,0,0,0,0,0,0,0,0,0,0,0,0,0,0,0,0,0,0,0,0,0,0,0,0,0,0,0,0,0,0,4,0,0,0,0,0,0,0,0,0,0,0,0,0,0,0,0,0,0,0,0,0,0,0,0,0,1,0,0,0,0,0,0

	
```

```
		  

		NW_003726308_1, Position,0,1000,2000,3000,4000,5000,6000,7000,8000,9000,10000,11000,12000,13000,14000,15000,16000,17000,18000,19000,20000,21000,22000,23000,24000,25000,26000,27000,28000,29000,30000,31000,32000,33000,34000,35000,36000,37000,38000,39000,40000,41000,42000,43000,44000,45000,46000,47000,48000,49000,50000,51000,52000,53000,54000,55000,56000,57000,58000,59000,60000,61000,62000,63000,64000,65000,66000,67000,68000,69000,70000,71000,72000,73000,74000,75000,76000,77000,78000,79000,80000,81000,82000,83000,84000
NW_003726308_1,Count,0,0,0,0,0,0,0,0,0,0,0,0,0,0,0,0,0,0,0,0,0,0,0,0,0,0,0,0,0,0,0,0,0,5,4,0,3,0,2,2,3,4,0,0,0,0,0,7,7,2,6,14,10,10,6,1,0,0,0,3,0,0,0,0,0,0,0,0,0,0,0,0,0,0,0,0,4,0,0,0,0,0,0,0,0

	
```

```
		  

		NW_003726311_1, Position,0,1000,2000,3000,4000,5000,6000,7000,8000,9000,10000,11000,12000,13000,14000,15000,16000,17000,18000,19000,20000,21000,22000,23000,24000,25000,26000,27000,28000,29000,30000,31000,32000,33000,34000,35000,36000,37000,38000,39000,40000,41000,42000,43000,44000,45000,46000,47000,48000,49000,50000,51000,52000,53000,54000,55000,56000
NW_003726311_1,Count,0,0,0,0,0,0,0,0,0,0,0,0,0,0,0,0,0,0,0,0,0,0,0,0,0,0,0,0,0,0,0,0,0,0,0,0,0,0,0,0,0,0,0,0,0,0,0,0,0,0,0,0,0,0,0,0,3

	
```

```
		  

		NW_003726312_1, Position,0,1000,2000,3000,4000,5000,6000,7000,8000,9000,10000,11000,12000,13000,14000,15000,16000,17000,18000,19000,20000,21000,22000,23000,24000,25000,26000,27000,28000,29000,30000,31000,32000,33000,34000,35000,36000,37000,38000,39000,40000,41000,42000,43000,44000,45000,46000,47000,48000,49000,50000,51000,52000,53000,54000,55000,56000,57000,58000,59000
NW_003726312_1,Count,0,0,0,0,0,0,0,0,0,0,0,0,0,0,0,0,0,0,0,0,0,0,0,0,0,0,0,3,0,2,2,10,0,5,0,0,0,0,0,0,0,0,0,0,0,0,0,1,0,0,0,0,0,0,0,0,0,0,0,0

	
```

```
		  

		NW_003726315_1, Position,0,1000,2000,3000,4000,5000,6000,7000,8000,9000,10000,11000,12000,13000,14000,15000,16000,17000,18000,19000,20000,21000,22000,23000,24000,25000,26000,27000,28000,29000,30000,31000,32000,33000,34000,35000,36000,37000,38000,39000,40000,41000,42000,43000,44000,45000,46000,47000,48000,49000,50000,51000,52000,53000,54000
NW_003726315_1,Count,0,0,0,0,0,0,0,0,0,0,0,0,0,0,0,0,0,0,0,0,0,0,0,0,0,3,2,0,0,0,0,0,0,0,0,0,0,0,0,0,0,0,0,0,0,0,0,0,0,0,0,0,0,0,0

	
```

```
		  

		NW_003726318_1, Position,0,1000,2000,3000,4000,5000,6000,7000,8000,9000,10000,11000,12000,13000,14000,15000,16000,17000,18000,19000,20000,21000,22000,23000,24000,25000,26000,27000,28000,29000,30000,31000,32000,33000,34000,35000,36000,37000,38000,39000,40000,41000,42000,43000,44000,45000,46000,47000,48000,49000,50000,51000,52000,53000,54000,55000,56000,57000
NW_003726318_1,Count,0,0,0,0,0,0,0,0,0,0,0,0,0,0,2,0,0,0,0,0,0,0,0,0,0,0,0,0,0,4,0,0,0,0,0,0,0,0,0,0,0,0,0,0,0,0,2,0,2,2,3,2,5,10,2,0,2,3

	
```

```
		  

		NW_003726321_1, Position,0,1000,2000,3000,4000,5000,6000,7000,8000,9000,10000,11000,12000,13000,14000,15000,16000,17000,18000,19000,20000,21000,22000,23000,24000,25000,26000,27000,28000,29000,30000,31000,32000,33000,34000,35000,36000,37000,38000,39000,40000,41000,42000,43000,44000,45000,46000,47000,48000,49000,50000,51000,52000,53000,54000
NW_003726321_1,Count,0,0,0,0,0,0,0,0,0,0,0,0,0,0,0,0,0,0,0,0,0,0,0,2,0,0,0,0,0,0,0,0,0,0,0,0,0,0,0,0,0,0,0,0,0,0,0,0,0,0,0,0,0,0,0

	
```

```
		  

		NW_003726327_1, Position,0,1000,2000,3000,4000,5000,6000,7000,8000,9000,10000,11000,12000,13000,14000,15000,16000,17000,18000,19000,20000,21000,22000,23000,24000,25000,26000,27000,28000,29000,30000,31000,32000,33000,34000,35000,36000,37000,38000,39000,40000,41000,42000,43000,44000,45000,46000,47000,48000,49000,50000,51000,52000
NW_003726327_1,Count,0,0,0,0,0,0,0,0,0,0,0,0,0,0,0,0,0,0,0,0,0,0,0,0,0,0,0,0,3,0,0,0,0,0,0,0,0,0,0,0,0,0,0,0,0,0,0,0,0,0,0,0,0

	
```

```
		  

		NW_003726329_1, Position,0,1000,2000,3000,4000,5000,6000,7000,8000,9000,10000,11000,12000,13000,14000,15000,16000,17000,18000,19000,20000,21000,22000,23000,24000,25000,26000,27000,28000,29000,30000,31000,32000,33000,34000,35000,36000,37000,38000,39000,40000,41000,42000,43000,44000,45000,46000,47000,48000,49000,50000
NW_003726329_1,Count,0,0,0,0,0,0,0,0,0,0,0,0,0,0,0,0,0,0,0,0,0,0,0,0,0,0,0,0,0,1,6,2,0,0,0,0,0,0,0,0,0,0,0,0,0,0,0,0,0,0,0

	
```

```
		  

		NW_003726330_1, Position,0,1000,2000,3000,4000,5000,6000,7000,8000,9000,10000,11000,12000,13000,14000,15000,16000,17000,18000,19000,20000,21000,22000,23000,24000,25000,26000,27000,28000,29000,30000,31000,32000,33000,34000,35000,36000,37000,38000,39000,40000,41000,42000,43000,44000,45000,46000,47000,48000,49000,50000,51000,52000,53000
NW_003726330_1,Count,0,1,0,0,0,3,0,0,0,0,2,0,0,0,0,0,0,0,2,0,0,0,0,0,0,0,0,0,0,0,0,0,0,0,0,0,0,0,0,0,0,0,0,0,0,0,0,0,0,0,0,0,0,0

	
```

```
		  

		NW_003726334_1, Position,0,1000,2000,3000,4000,5000,6000,7000,8000,9000,10000,11000,12000,13000,14000,15000,16000,17000,18000,19000,20000,21000,22000,23000,24000,25000,26000,27000,28000,29000,30000,31000,32000,33000,34000,35000,36000,37000,38000,39000,40000,41000,42000,43000,44000,45000,46000,47000,48000,49000,50000,51000,52000,53000,54000,55000,56000,57000,58000,59000
NW_003726334_1,Count,0,0,0,0,0,0,4,0,0,0,0,0,4,0,0,0,0,0,0,0,0,0,0,0,0,0,0,0,0,0,0,1,0,0,0,0,6,16,2,0,0,0,0,0,5,12,12,7,12,5,10,10,6,0,12,11,12,4,3,6

	
```

```
		  

		NW_003726336_1, Position,0,1000,2000,3000,4000,5000,6000,7000,8000,9000,10000,11000,12000,13000,14000,15000,16000,17000,18000,19000,20000,21000,22000,23000,24000,25000,26000,27000,28000,29000,30000,31000,32000,33000,34000,35000,36000,37000,38000,39000,40000,41000,42000,43000,44000,45000,46000,47000,48000,49000
NW_003726336_1,Count,0,0,0,0,5,5,6,6,9,14,5,9,6,10,10,9,10,12,13,16,10,4,11,17,18,7,9,6,5,5,7,1,3,0,3,4,6,10,1,4,7,9,0,2,2,0,8,0,0,0

	
```

```
		  

		NW_003726338_1, Position,0,1000,2000,3000,4000,5000,6000,7000,8000,9000,10000,11000,12000,13000,14000,15000,16000,17000,18000,19000,20000,21000,22000,23000,24000,25000,26000,27000,28000,29000,30000,31000,32000,33000,34000,35000,36000,37000,38000,39000,40000,41000,42000,43000,44000,45000,46000,47000,48000,49000,50000,51000,52000,53000,54000,55000,56000,57000
NW_003726338_1,Count,0,0,0,0,0,2,0,0,0,0,0,0,0,0,0,0,0,0,0,0,0,0,0,0,0,0,0,0,0,0,0,0,0,0,0,0,0,0,0,0,0,0,0,0,0,0,0,0,0,0,0,0,0,0,0,0,0,0

	
```

```
		  

		NW_003726339_1, Position,0,1000,2000,3000,4000,5000,6000,7000,8000,9000,10000,11000,12000,13000,14000,15000,16000,17000,18000,19000,20000,21000,22000,23000,24000,25000,26000,27000,28000,29000,30000,31000,32000,33000,34000,35000,36000,37000,38000,39000,40000,41000,42000,43000,44000,45000,46000,47000,48000,49000,50000,51000,52000,53000,54000,55000,56000,57000,58000,59000,60000,61000,62000,63000,64000,65000,66000,67000,68000,69000,70000,71000,72000,73000,74000
NW_003726339_1,Count,0,0,0,0,0,0,0,0,0,0,0,0,0,0,0,0,0,0,0,0,0,0,0,0,0,0,0,0,0,0,0,0,0,0,0,0,0,0,0,0,0,0,0,1,0,0,0,0,0,0,0,0,0,0,0,0,0,0,0,0,0,0,0,0,0,0,0,0,0,0,0,0,0,0,0

	
```

```
		  

		NW_003726342_1, Position,0,1000,2000,3000,4000,5000,6000,7000,8000,9000,10000,11000,12000,13000,14000,15000,16000,17000,18000,19000,20000,21000,22000,23000,24000,25000,26000,27000,28000,29000,30000,31000,32000,33000,34000,35000,36000,37000,38000,39000,40000,41000,42000,43000,44000,45000,46000,47000,48000,49000,50000,51000,52000,53000,54000,55000,56000,57000,58000,59000,60000,61000,62000,63000,64000,65000,66000,67000,68000,69000,70000,71000,72000,73000,74000,75000
NW_003726342_1,Count,7,0,0,0,0,0,0,0,0,0,0,0,0,0,0,0,0,0,0,0,0,0,0,0,0,0,0,0,0,0,0,0,0,0,0,0,0,0,0,0,0,0,0,0,0,0,0,0,0,0,0,0,0,0,0,0,0,0,0,0,0,0,0,0,0,0,0,0,0,0,0,0,0,0,0,0

	
```

```
		  

		NW_003726343_1, Position,0,1000,2000,3000,4000,5000,6000,7000,8000,9000,10000,11000,12000,13000,14000,15000,16000,17000,18000,19000,20000,21000,22000,23000,24000,25000,26000,27000,28000,29000,30000,31000,32000,33000,34000,35000,36000,37000,38000,39000,40000,41000,42000,43000,44000,45000,46000,47000,48000
NW_003726343_1,Count,0,11,9,0,4,2,3,6,7,8,6,10,9,1,5,16,2,1,6,1,0,0,0,0,0,0,0,0,0,0,0,0,0,0,0,0,0,0,0,0,0,0,0,0,0,0,0,0,0

	
```

```
		  

		NW_003726344_1, Position,0,1000,2000,3000,4000,5000,6000,7000,8000,9000,10000,11000,12000,13000,14000,15000,16000,17000,18000,19000,20000,21000,22000,23000,24000,25000,26000,27000,28000,29000,30000,31000,32000,33000,34000,35000,36000,37000,38000,39000,40000,41000,42000,43000,44000,45000,46000,47000
NW_003726344_1,Count,0,0,0,0,7,6,13,15,16,13,8,2,15,8,6,7,12,6,10,4,9,7,9,7,9,6,14,14,9,10,10,11,10,7,6,8,13,10,7,7,1,3,2,0,0,0,0,0

	
```

```
		  

		NW_003726348_1, Position,0,1000,2000,3000,4000,5000,6000,7000,8000,9000,10000,11000,12000,13000,14000,15000,16000,17000,18000,19000,20000,21000,22000,23000,24000,25000,26000,27000,28000,29000,30000,31000,32000,33000,34000,35000,36000,37000,38000,39000,40000,41000,42000,43000,44000,45000,46000,47000,48000,49000,50000,51000,52000,53000
NW_003726348_1,Count,5,10,10,1,1,0,4,5,1,3,6,0,2,3,0,3,2,9,1,0,1,2,6,2,2,3,3,1,1,7,2,2,7,0,0,0,0,0,0,0,0,0,0,0,0,0,0,0,0,0,0,0,0,0

	
```

```
		  

		NW_003726349_1, Position,0,1000,2000,3000,4000,5000,6000,7000,8000,9000,10000,11000,12000,13000,14000,15000,16000,17000,18000,19000,20000,21000,22000,23000,24000,25000,26000,27000,28000,29000,30000,31000,32000,33000,34000,35000,36000,37000,38000,39000,40000,41000,42000,43000,44000,45000,46000,47000,48000,49000,50000,51000,52000,53000,54000,55000,56000,57000,58000,59000,60000,61000,62000,63000,64000,65000,66000,67000,68000,69000,70000,71000,72000,73000
NW_003726349_1,Count,0,0,0,0,0,0,0,0,0,0,0,0,0,0,0,0,0,0,0,0,0,0,0,7,10,13,3,0,0,0,0,0,0,0,0,0,0,0,0,0,0,0,0,0,0,0,0,0,0,0,0,0,0,0,0,0,0,0,0,0,0,0,0,0,0,0,0,0,0,0,0,0,0,0

	
```

```
		  

		NW_003726351_1, Position,0,1000,2000,3000,4000,5000,6000,7000,8000,9000,10000,11000,12000,13000,14000,15000,16000,17000,18000,19000,20000,21000,22000,23000,24000,25000,26000,27000,28000,29000,30000,31000,32000,33000,34000,35000,36000,37000,38000,39000,40000,41000,42000,43000,44000,45000,46000,47000,48000,49000,50000,51000,52000,53000,54000,55000,56000,57000,58000,59000,60000,61000,62000,63000,64000,65000,66000,67000,68000,69000,70000
NW_003726351_1,Count,0,0,0,0,0,0,0,0,0,0,0,0,0,0,0,0,0,0,0,0,0,0,0,0,0,0,0,0,0,0,0,0,0,0,0,0,0,0,0,0,0,0,0,0,0,0,0,0,0,8,1,0,0,0,0,0,0,0,0,0,0,0,0,0,0,0,0,0,0,0,0

	
```

```
		  

		NW_003726352_1, Position,0,1000,2000,3000,4000,5000,6000,7000,8000,9000,10000,11000,12000,13000,14000,15000,16000,17000,18000,19000,20000,21000,22000,23000,24000,25000,26000,27000,28000,29000,30000,31000,32000,33000,34000,35000,36000,37000,38000,39000,40000,41000,42000,43000,44000,45000,46000,47000,48000,49000,50000,51000,52000,53000,54000,55000,56000,57000,58000,59000,60000,61000,62000,63000,64000,65000,66000,67000
NW_003726352_1,Count,3,0,0,0,0,0,0,0,0,0,0,0,0,0,0,0,0,0,0,0,0,0,0,0,0,0,0,0,0,0,0,0,0,0,0,0,0,0,0,0,0,0,0,0,0,0,0,0,0,0,0,0,0,0,0,0,0,0,0,0,0,0,0,0,0,0,0,0

	
```

```
		  

		NW_003726353_1, Position,0,1000,2000,3000,4000,5000,6000,7000,8000,9000,10000,11000,12000,13000,14000,15000,16000,17000,18000,19000,20000,21000,22000,23000,24000,25000,26000,27000,28000,29000,30000,31000,32000,33000,34000,35000,36000,37000,38000,39000,40000,41000,42000,43000,44000,45000,46000,47000,48000
NW_003726353_1,Count,5,0,4,4,0,0,0,0,0,0,0,0,0,0,1,0,11,4,5,1,0,3,0,0,0,3,0,2,0,0,2,0,1,0,2,5,0,2,0,0,0,1,0,1,2,17,4,0,0

	
```

```
		  

		NW_003726356_1, Position,0,1000,2000,3000,4000,5000,6000,7000,8000,9000,10000,11000,12000,13000,14000,15000,16000,17000,18000,19000,20000,21000,22000,23000,24000,25000,26000,27000,28000,29000,30000,31000,32000,33000,34000,35000,36000,37000,38000,39000,40000,41000,42000,43000,44000,45000,46000,47000,48000,49000,50000,51000,52000,53000,54000,55000,56000,57000,58000
NW_003726356_1,Count,10,2,2,3,7,7,7,10,0,0,0,3,10,13,14,13,15,10,10,10,9,13,9,5,11,3,6,3,10,6,0,0,0,0,0,0,0,0,0,0,0,8,0,0,3,8,3,0,0,9,13,2,8,10,6,13,14,4,7

	
```

```
		  

		NW_003726357_1, Position,0,1000,2000,3000,4000,5000,6000,7000,8000,9000,10000,11000,12000,13000,14000,15000,16000,17000,18000,19000,20000,21000,22000,23000,24000,25000,26000,27000,28000,29000,30000,31000,32000,33000,34000,35000,36000,37000,38000,39000,40000,41000,42000,43000,44000,45000
NW_003726357_1,Count,0,0,0,0,0,0,0,0,4,0,1,6,0,0,0,0,0,0,0,0,0,1,0,0,5,0,0,0,0,0,0,0,0,0,0,9,1,3,0,1,0,0,0,0,3,0

	
```

```
		  

		NW_003726358_1, Position,0,1000,2000,3000,4000,5000,6000,7000,8000,9000,10000,11000,12000,13000,14000,15000,16000,17000,18000,19000,20000,21000,22000,23000,24000,25000,26000,27000,28000,29000,30000,31000,32000,33000,34000,35000,36000,37000,38000,39000,40000,41000,42000,43000,44000,45000,46000
NW_003726358_1,Count,0,0,0,0,0,0,0,0,0,0,0,2,1,0,0,1,0,0,0,0,0,0,0,0,0,0,0,0,0,0,0,0,0,0,0,0,3,0,0,0,0,0,0,0,0,0,0

	
```

```
		  

		NW_003726362_1, Position,0,1000,2000,3000,4000,5000,6000,7000,8000,9000,10000,11000,12000,13000,14000,15000,16000,17000,18000,19000,20000,21000,22000,23000,24000,25000,26000,27000,28000,29000,30000,31000,32000,33000,34000,35000,36000,37000,38000,39000,40000,41000,42000,43000,44000,45000,46000,47000
NW_003726362_1,Count,0,0,0,0,0,0,0,0,0,0,0,0,0,0,0,0,0,0,0,0,0,0,0,0,0,0,0,0,0,3,0,0,0,0,0,0,0,0,0,0,0,0,0,0,0,0,0,0

	
```

```
		  

		NW_003726363_1, Position,0,1000,2000,3000,4000,5000,6000,7000,8000,9000,10000,11000,12000,13000,14000,15000,16000,17000,18000,19000,20000,21000,22000,23000,24000,25000,26000,27000,28000,29000,30000,31000,32000,33000,34000,35000,36000,37000,38000,39000,40000,41000,42000,43000,44000
NW_003726363_1,Count,0,0,0,0,0,0,0,0,0,1,0,0,0,0,0,0,0,0,0,0,0,0,0,0,4,1,0,0,0,0,0,0,0,0,0,0,0,0,0,0,0,0,0,0,0

	
```

```
		  

		NW_003726365_1, Position,0,1000,2000,3000,4000,5000,6000,7000,8000,9000,10000,11000,12000,13000,14000,15000,16000,17000,18000,19000,20000,21000,22000,23000,24000,25000,26000,27000,28000,29000,30000,31000,32000,33000,34000,35000,36000,37000,38000,39000,40000,41000,42000,43000,44000,45000,46000,47000,48000,49000,50000,51000
NW_003726365_1,Count,0,0,0,0,0,2,2,0,0,0,3,0,0,0,0,0,0,0,0,0,0,0,0,0,0,0,0,5,0,0,3,1,3,10,2,4,0,0,6,13,2,0,0,0,0,0,0,0,0,0,0,0

	
```

```
		  

		NW_003726367_1, Position,0,1000,2000,3000,4000,5000,6000,7000,8000,9000,10000,11000,12000,13000,14000,15000,16000,17000,18000,19000,20000,21000,22000,23000,24000,25000,26000,27000,28000,29000,30000,31000,32000,33000,34000,35000,36000,37000,38000,39000,40000,41000,42000,43000,44000,45000,46000,47000,48000,49000,50000
NW_003726367_1,Count,0,0,0,0,0,0,0,0,0,0,0,0,0,0,0,0,1,2,0,0,0,0,0,0,0,0,0,0,0,0,0,0,0,0,0,0,0,0,0,0,0,0,0,0,0,0,0,0,0,0,0

	
```

```
		  

		NW_003726369_1, Position,0,1000,2000,3000,4000,5000,6000,7000,8000,9000,10000,11000,12000,13000,14000,15000,16000,17000,18000,19000,20000,21000,22000,23000,24000,25000,26000,27000,28000,29000,30000,31000,32000,33000,34000,35000,36000,37000,38000,39000,40000,41000,42000,43000,44000
NW_003726369_1,Count,0,0,0,0,0,0,0,0,0,0,0,0,0,0,0,0,0,0,0,0,0,0,0,6,0,0,0,0,0,0,0,0,0,0,0,0,0,0,0,0,0,0,0,0,0

	
```

```
		  

		NW_003726370_1, Position,0,1000,2000,3000,4000,5000,6000,7000,8000,9000,10000,11000,12000,13000,14000,15000,16000,17000,18000,19000,20000,21000,22000,23000,24000,25000,26000,27000,28000,29000,30000,31000,32000,33000,34000,35000,36000,37000,38000,39000,40000,41000,42000,43000
NW_003726370_1,Count,1,0,6,7,5,10,4,2,2,10,13,4,10,8,7,9,5,3,11,2,6,6,3,3,4,4,7,12,4,8,3,2,4,5,4,7,10,6,8,4,4,4,4,2

	
```

```
		  

		NW_003726378_1, Position,0,1000,2000,3000,4000,5000,6000,7000,8000,9000,10000,11000,12000,13000,14000,15000,16000,17000,18000,19000,20000,21000,22000,23000,24000,25000,26000,27000,28000,29000,30000,31000,32000,33000,34000,35000,36000,37000,38000,39000,40000,41000,42000
NW_003726378_1,Count,0,0,0,0,0,0,0,0,0,0,0,0,0,0,0,0,0,0,0,0,0,0,0,0,0,0,0,0,0,0,0,0,8,3,0,0,0,1,0,0,0,3,3

	
```

```
		  

		NW_003726381_1, Position,0,1000,2000,3000,4000,5000,6000,7000,8000,9000,10000,11000,12000,13000,14000,15000,16000,17000,18000,19000,20000,21000,22000,23000,24000,25000,26000,27000,28000,29000,30000,31000,32000,33000,34000,35000,36000,37000,38000,39000,40000,41000,42000,43000,44000,45000,46000,47000,48000,49000,50000,51000,52000,53000,54000,55000,56000,57000,58000,59000,60000,61000,62000,63000,64000,65000,66000,67000,68000,69000
NW_003726381_1,Count,0,0,0,0,0,0,0,0,0,0,0,0,0,0,0,0,0,0,0,0,0,0,0,0,0,0,0,0,0,0,0,0,0,0,0,0,0,0,0,0,0,0,0,0,0,0,0,0,0,0,0,0,0,0,0,5,1,2,0,0,0,1,0,0,0,0,0,0,0,0

	
```

```
		  

		NW_003726386_1, Position,0,1000,2000,3000,4000,5000,6000,7000,8000,9000,10000,11000,12000,13000,14000,15000,16000,17000,18000,19000,20000,21000,22000,23000,24000,25000,26000,27000,28000,29000,30000,31000,32000,33000,34000,35000,36000,37000,38000,39000,40000,41000,42000,43000
NW_003726386_1,Count,0,0,1,5,10,10,0,0,0,0,0,0,0,0,0,0,9,1,7,6,8,3,1,3,2,9,3,9,11,11,4,9,4,12,2,0,7,5,14,7,9,0,0,0

	
```

```
		  

		NW_003726389_1, Position,0,1000,2000,3000,4000,5000,6000,7000,8000,9000,10000,11000,12000,13000,14000,15000,16000,17000,18000,19000,20000,21000,22000,23000,24000,25000,26000,27000,28000,29000,30000,31000,32000,33000,34000,35000,36000,37000,38000,39000,40000,41000,42000,43000,44000,45000,46000,47000,48000,49000,50000,51000,52000,53000,54000,55000,56000,57000
NW_003726389_1,Count,0,0,0,0,0,0,0,0,0,0,0,0,0,0,0,0,0,0,0,0,0,0,0,0,0,0,0,0,0,0,0,0,0,0,0,0,0,0,4,0,0,0,0,0,0,0,0,0,0,0,0,0,0,0,0,0,0,0

	
```

```
		  

		NW_003726390_1, Position,0,1000,2000,3000,4000,5000,6000,7000,8000,9000,10000,11000,12000,13000,14000,15000,16000,17000,18000,19000,20000,21000,22000,23000,24000,25000,26000,27000,28000,29000,30000,31000,32000,33000,34000,35000,36000,37000,38000,39000,40000,41000,42000
NW_003726390_1,Count,0,0,0,0,0,0,0,0,0,0,0,0,0,0,0,0,0,0,0,0,0,0,0,0,0,0,0,0,0,0,2,0,0,0,0,0,0,0,0,0,0,0,0

	
```

```
		  

		NW_003726392_1, Position,0,1000,2000,3000,4000,5000,6000,7000,8000,9000,10000,11000,12000,13000,14000,15000,16000,17000,18000,19000,20000,21000,22000,23000,24000,25000,26000,27000,28000,29000,30000,31000,32000,33000,34000,35000,36000,37000,38000,39000,40000
NW_003726392_1,Count,0,6,3,5,3,5,2,4,0,4,4,3,7,5,8,3,7,3,0,6,3,7,5,6,5,5,1,2,4,9,3,7,4,3,9,6,7,0,0,0,0

	
```

```
		  

		NW_003726394_1, Position,0,1000,2000,3000,4000,5000,6000,7000,8000,9000,10000,11000,12000,13000,14000,15000,16000,17000,18000,19000,20000,21000,22000,23000,24000,25000,26000,27000,28000,29000,30000,31000,32000,33000,34000,35000,36000,37000,38000,39000,40000,41000
NW_003726394_1,Count,0,0,0,0,0,0,0,0,0,0,0,0,0,0,0,0,0,0,0,0,1,5,0,0,0,0,0,0,0,0,0,0,0,0,0,0,0,0,0,0,0,0

	
```

```
		  

		NW_003726396_1, Position,0,1000,2000,3000,4000,5000,6000,7000,8000,9000,10000,11000,12000,13000,14000,15000,16000,17000,18000,19000,20000,21000,22000,23000,24000,25000,26000,27000,28000,29000,30000,31000,32000,33000,34000,35000,36000,37000,38000,39000,40000,41000,42000,43000,44000,45000,46000
NW_003726396_1,Count,0,0,0,0,0,0,0,5,0,0,0,0,0,0,0,0,2,0,0,0,2,0,0,0,1,0,0,0,0,0,0,0,0,0,0,0,0,0,0,0,0,0,0,0,0,0,0

	
```

```
		  

		NW_003726399_1, Position,0,1000,2000,3000,4000,5000,6000,7000,8000,9000,10000,11000,12000,13000,14000,15000,16000,17000,18000,19000,20000,21000,22000,23000,24000,25000,26000,27000,28000,29000,30000,31000,32000,33000,34000,35000,36000,37000,38000,39000,40000
NW_003726399_1,Count,0,0,0,0,0,0,0,0,0,0,0,0,0,0,0,0,0,1,0,0,0,1,0,5,0,2,6,4,1,0,1,0,0,0,0,0,0,4,0,0,0

	
```

```
		  

		NW_003726400_1, Position,0,1000,2000,3000,4000,5000,6000,7000,8000,9000,10000,11000,12000,13000,14000,15000,16000,17000,18000,19000,20000,21000,22000,23000,24000,25000,26000,27000,28000,29000,30000,31000,32000,33000,34000,35000,36000,37000,38000,39000,40000,41000,42000,43000,44000,45000
NW_003726400_1,Count,0,0,0,0,0,0,0,0,0,0,0,0,0,0,0,0,0,0,1,0,0,0,0,0,0,0,0,0,0,0,0,0,0,0,0,0,0,0,0,0,0,0,0,0,0,0

	
```

```
		  

		NW_003726405_1, Position,0,1000,2000,3000,4000,5000,6000,7000,8000,9000,10000,11000,12000,13000,14000,15000,16000,17000,18000,19000,20000,21000,22000,23000,24000,25000,26000,27000,28000,29000,30000,31000,32000,33000,34000,35000,36000,37000,38000,39000
NW_003726405_1,Count,7,17,10,16,13,7,6,6,9,10,13,10,14,12,13,15,17,10,11,13,11,18,12,10,11,10,6,8,7,6,2,12,10,10,8,9,0,2,6,0

	
```

```
		  

		NW_003726407_1, Position,0,1000,2000,3000,4000,5000,6000,7000,8000,9000,10000,11000,12000,13000,14000,15000,16000,17000,18000,19000,20000,21000,22000,23000,24000,25000,26000,27000,28000,29000,30000,31000,32000,33000,34000,35000,36000,37000,38000,39000,40000,41000,42000,43000,44000,45000,46000,47000
NW_003726407_1,Count,0,0,3,0,0,0,0,0,0,0,0,0,0,10,19,8,0,3,2,3,1,0,0,0,0,0,0,0,0,0,0,0,0,0,0,0,0,0,0,0,0,0,0,0,0,0,0,0

	
```

```
		  

		NW_003726410_1, Position,0,1000,2000,3000,4000,5000,6000,7000,8000,9000,10000,11000,12000,13000,14000,15000,16000,17000,18000,19000,20000,21000,22000,23000,24000,25000,26000,27000,28000,29000,30000,31000,32000,33000,34000,35000,36000,37000,38000,39000,40000,41000,42000,43000,44000,45000,46000,47000,48000,49000,50000,51000,52000,53000,54000,55000,56000,57000,58000,59000,60000,61000,62000,63000,64000
NW_003726410_1,Count,0,0,2,0,0,0,0,0,0,0,0,0,0,0,0,0,0,0,0,0,0,0,0,0,0,0,0,0,0,0,0,0,0,0,0,1,0,12,6,10,9,9,0,0,0,0,0,0,0,0,0,0,0,0,0,0,0,0,0,0,0,0,0,0,0

	
```

```
		  

		NW_003726416_1, Position,0,1000,2000,3000,4000,5000,6000,7000,8000,9000,10000,11000,12000,13000,14000,15000,16000,17000,18000,19000,20000,21000,22000,23000,24000,25000,26000,27000,28000,29000,30000,31000,32000,33000,34000,35000,36000,37000,38000,39000,40000,41000
NW_003726416_1,Count,1,0,0,7,0,1,6,0,3,0,0,0,1,0,0,0,0,0,0,0,0,0,0,0,0,0,0,0,7,2,0,0,0,0,0,0,0,0,0,0,0,0

	
```

```
		  

		NW_003726419_1, Position,0,1000,2000,3000,4000,5000,6000,7000,8000,9000,10000,11000,12000,13000,14000,15000,16000,17000,18000,19000,20000,21000,22000,23000,24000,25000,26000,27000,28000,29000,30000,31000,32000,33000,34000,35000,36000,37000,38000,39000,40000,41000,42000,43000,44000,45000,46000,47000,48000,49000,50000,51000,52000,53000,54000,55000,56000,57000,58000,59000
NW_003726419_1,Count,0,0,0,0,0,0,0,0,0,0,0,0,0,0,0,0,0,0,0,0,0,0,0,0,0,0,0,0,0,0,0,0,0,0,0,0,0,0,0,0,0,0,0,0,0,0,0,0,0,0,0,0,0,0,3,0,0,0,0,0

	
```

```
		  

		NW_003726420_1, Position,0,1000,2000,3000,4000,5000,6000,7000,8000,9000,10000,11000,12000,13000,14000,15000,16000,17000,18000,19000,20000,21000,22000,23000,24000,25000,26000,27000,28000,29000,30000,31000,32000,33000,34000,35000,36000,37000,38000,39000,40000,41000,42000,43000,44000,45000,46000,47000,48000,49000,50000,51000,52000,53000,54000,55000,56000,57000,58000
NW_003726420_1,Count,0,0,0,0,0,0,0,0,0,0,0,0,0,0,0,0,0,0,2,0,0,0,0,0,0,0,0,0,0,0,0,0,0,0,0,0,0,0,0,0,0,0,0,0,0,0,0,0,0,0,0,0,0,0,0,0,0,0,0

	
```

```
		  

		NW_003726422_1, Position,0,1000,2000,3000,4000,5000,6000,7000,8000,9000,10000,11000,12000,13000,14000,15000,16000,17000,18000,19000,20000,21000,22000,23000,24000,25000,26000,27000,28000,29000,30000,31000,32000,33000,34000,35000,36000,37000,38000,39000,40000,41000,42000,43000,44000,45000,46000,47000,48000,49000,50000,51000,52000
NW_003726422_1,Count,0,0,0,0,0,0,0,0,0,0,2,0,0,0,0,0,1,0,0,0,0,0,0,0,0,0,0,0,0,0,0,1,2,0,0,0,0,0,0,0,0,0,0,0,0,0,0,0,0,0,0,0,0

	
```

```
		  

		NW_003726424_1, Position,0,1000,2000,3000,4000,5000,6000,7000,8000,9000,10000,11000,12000,13000,14000,15000,16000,17000,18000,19000,20000,21000,22000,23000,24000,25000,26000,27000,28000,29000,30000,31000,32000,33000,34000,35000,36000,37000,38000,39000,40000,41000,42000,43000,44000,45000,46000,47000,48000,49000,50000,51000,52000,53000,54000,55000,56000,57000,58000,59000,60000,61000,62000,63000,64000,65000,66000,67000,68000,69000
NW_003726424_1,Count,0,0,1,0,0,0,0,0,0,0,0,0,0,0,0,0,0,0,0,0,0,0,0,0,0,0,0,0,0,0,0,0,0,0,0,0,0,0,0,0,0,0,0,0,0,0,0,0,0,0,0,0,0,0,0,0,0,0,0,0,0,0,0,0,0,0,0,0,0,0

	
```

```
		  

		NW_003726425_1, Position,0,1000,2000,3000,4000,5000,6000,7000,8000,9000,10000,11000,12000,13000,14000,15000,16000,17000,18000,19000,20000,21000,22000,23000,24000,25000,26000,27000,28000,29000,30000,31000,32000,33000,34000,35000,36000,37000,38000,39000,40000,41000,42000,43000,44000
NW_003726425_1,Count,0,0,0,0,0,0,0,0,0,0,0,0,0,0,0,0,0,0,0,0,0,0,0,0,0,0,0,0,0,0,0,0,0,0,0,0,0,4,0,0,0,0,0,0,0

	
```

```
		  

		NW_003726427_1, Position,0,1000,2000,3000,4000,5000,6000,7000,8000,9000,10000,11000,12000,13000,14000,15000,16000,17000,18000,19000,20000,21000,22000,23000,24000,25000,26000,27000,28000,29000,30000,31000,32000,33000,34000,35000,36000
NW_003726427_1,Count,3,0,0,0,0,0,0,0,0,0,0,0,0,0,0,0,0,0,0,0,2,0,0,0,0,2,0,0,0,0,3,0,1,3,6,4,7

	
```

```
		  

		NW_003726435_1, Position,0,1000,2000,3000,4000,5000,6000,7000,8000,9000,10000,11000,12000,13000,14000,15000,16000,17000,18000,19000,20000,21000,22000,23000,24000,25000,26000,27000,28000,29000,30000,31000,32000,33000,34000,35000,36000,37000
NW_003726435_1,Count,0,0,0,0,0,0,0,0,0,0,3,0,0,0,0,0,0,0,0,8,21,5,0,0,0,22,11,20,23,22,14,0,0,0,0,0,0,0

	
```

```
		  

		NW_003726437_1, Position,0,1000,2000,3000,4000,5000,6000,7000,8000,9000,10000,11000,12000,13000,14000,15000,16000,17000,18000,19000,20000,21000,22000,23000,24000,25000,26000,27000,28000,29000,30000,31000,32000,33000,34000,35000,36000,37000,38000,39000,40000,41000
NW_003726437_1,Count,0,4,7,2,5,0,0,0,0,0,6,5,4,4,4,1,10,4,0,10,10,13,7,9,4,1,12,1,3,0,2,0,0,0,0,0,1,3,2,10,12,5

	
```

```
		  

		NW_003726438_1, Position,0,1000,2000,3000,4000,5000,6000,7000,8000,9000,10000,11000,12000,13000,14000,15000,16000,17000,18000,19000,20000,21000,22000,23000,24000,25000,26000,27000,28000,29000,30000,31000,32000,33000,34000,35000,36000,37000,38000,39000,40000,41000,42000,43000,44000,45000
NW_003726438_1,Count,0,0,0,0,0,0,0,0,0,0,0,0,0,0,0,0,0,3,0,0,0,0,0,0,0,0,0,0,0,0,0,0,0,0,0,0,0,0,0,0,0,0,0,0,1,0

	
```

```
		  

		NW_003726439_1, Position,0,1000,2000,3000,4000,5000,6000,7000,8000,9000,10000,11000,12000,13000,14000,15000,16000,17000,18000,19000,20000,21000,22000,23000,24000,25000,26000,27000,28000,29000,30000,31000,32000,33000,34000,35000,36000,37000,38000,39000,40000
NW_003726439_1,Count,0,0,0,0,0,0,0,0,0,0,0,0,0,0,0,0,0,0,0,0,1,0,0,0,0,0,0,0,0,0,0,0,0,0,0,0,0,0,0,0,0

	
```

```
		  

		NW_003726442_1, Position,0,1000,2000,3000,4000,5000,6000,7000,8000,9000,10000,11000,12000,13000,14000,15000,16000,17000,18000,19000,20000,21000,22000,23000,24000,25000,26000,27000,28000,29000,30000,31000,32000,33000,34000,35000
NW_003726442_1,Count,5,9,13,12,8,5,7,6,15,11,10,3,5,2,9,11,11,9,16,6,4,11,12,13,13,12,4,6,0,3,3,5,4,17,12,4

	
```

```
		  

		NW_003726445_1, Position,0,1000,2000,3000,4000,5000,6000,7000,8000,9000,10000,11000,12000,13000,14000,15000,16000,17000,18000,19000,20000,21000,22000,23000,24000,25000,26000,27000,28000,29000,30000,31000,32000,33000,34000,35000,36000,37000,38000,39000,40000,41000,42000,43000,44000,45000,46000,47000,48000,49000,50000,51000,52000,53000,54000,55000,56000,57000,58000,59000,60000,61000,62000,63000,64000,65000,66000,67000
NW_003726445_1,Count,0,0,0,0,0,0,0,0,0,0,0,0,0,0,0,0,0,0,0,0,0,0,0,0,0,0,0,0,0,0,0,0,0,0,0,0,0,0,0,0,0,0,0,0,0,0,0,0,0,0,0,0,0,0,0,0,0,0,0,0,0,3,0,0,0,0,0,0

	
```

```
		  

		NW_003726447_1, Position,0,1000,2000,3000,4000,5000,6000,7000,8000,9000,10000,11000,12000,13000,14000,15000,16000,17000,18000,19000,20000,21000,22000,23000,24000,25000,26000,27000,28000,29000,30000,31000,32000,33000,34000,35000,36000,37000,38000,39000,40000,41000,42000,43000,44000,45000,46000,47000,48000,49000,50000,51000,52000
NW_003726447_1,Count,0,0,0,0,0,0,0,0,0,0,0,0,0,0,0,0,0,0,0,0,0,0,0,0,0,0,0,0,0,1,0,0,0,0,0,0,0,0,0,0,0,0,0,0,0,0,0,0,0,0,0,0,0

	
```

```
		  

		NW_003726455_1, Position,0,1000,2000,3000,4000,5000,6000,7000,8000,9000,10000,11000,12000,13000,14000,15000,16000,17000,18000,19000,20000,21000,22000,23000,24000,25000,26000,27000,28000,29000,30000,31000,32000,33000,34000
NW_003726455_1,Count,0,3,1,0,0,6,5,5,7,5,4,0,0,0,0,0,2,8,5,2,11,1,7,3,9,4,0,0,0,0,0,0,6,1,0

	
```

```
		  

		NW_003726457_1, Position,0,1000,2000,3000,4000,5000,6000,7000,8000,9000,10000,11000,12000,13000,14000,15000,16000,17000,18000,19000,20000,21000,22000,23000,24000,25000,26000,27000,28000,29000,30000,31000,32000,33000,34000,35000,36000,37000
NW_003726457_1,Count,0,0,0,2,2,1,0,0,0,0,0,0,0,0,0,0,0,0,0,0,0,0,2,0,0,0,0,2,2,0,0,0,0,0,0,2,0,0

	
```

```
		  

		NW_003726458_1, Position,0,1000,2000,3000,4000,5000,6000,7000,8000,9000,10000,11000,12000,13000,14000,15000,16000,17000,18000,19000,20000,21000,22000,23000,24000,25000,26000,27000,28000,29000,30000,31000,32000,33000,34000,35000,36000,37000,38000,39000,40000,41000,42000,43000,44000,45000,46000,47000,48000,49000,50000,51000,52000,53000,54000,55000,56000,57000,58000,59000
NW_003726458_1,Count,0,0,0,0,0,0,0,0,0,0,0,0,0,0,0,1,0,0,0,0,0,0,0,0,0,0,0,0,0,0,0,0,0,0,0,0,0,0,0,0,0,0,0,0,0,0,0,0,0,0,0,0,0,0,0,0,0,0,0,0

	
```

```
		  

		NW_003726459_1, Position,0,1000,2000,3000,4000,5000,6000,7000,8000,9000,10000,11000,12000,13000,14000,15000,16000,17000,18000,19000,20000,21000,22000,23000,24000,25000,26000,27000,28000,29000,30000,31000,32000,33000,34000
NW_003726459_1,Count,0,0,0,0,0,0,0,0,0,0,0,0,0,0,0,0,0,0,0,0,0,0,0,0,0,0,0,0,0,0,0,4,0,2,1

	
```

```
		  

		NW_003726460_1, Position,0,1000,2000,3000,4000,5000,6000,7000,8000,9000,10000,11000,12000,13000,14000,15000,16000,17000,18000,19000,20000,21000,22000,23000,24000,25000,26000,27000,28000,29000,30000,31000,32000,33000,34000,35000
NW_003726460_1,Count,0,0,0,0,0,1,0,1,5,0,0,0,0,0,0,0,0,0,10,5,0,0,0,0,0,0,0,0,0,0,0,0,0,0,0,0

	
```

```
		  

		NW_003726461_1, Position,0,1000,2000,3000,4000,5000,6000,7000,8000,9000,10000,11000,12000,13000,14000,15000,16000,17000,18000,19000,20000,21000,22000,23000,24000,25000,26000,27000,28000,29000,30000,31000,32000,33000,34000,35000,36000,37000,38000,39000,40000,41000
NW_003726461_1,Count,0,0,0,0,0,0,0,0,0,0,0,0,0,0,0,0,0,0,0,0,7,0,0,0,0,0,0,1,3,0,0,0,0,0,0,12,9,11,4,7,13,4

	
```

```
		  

		NW_003726462_1, Position,0,1000,2000,3000,4000,5000,6000,7000,8000,9000,10000,11000,12000,13000,14000,15000,16000,17000,18000,19000,20000,21000,22000,23000,24000,25000,26000,27000,28000,29000,30000,31000,32000,33000
NW_003726462_1,Count,0,6,3,0,14,0,0,2,0,0,2,9,9,0,0,0,3,6,0,0,5,6,0,0,0,0,0,0,0,0,1,0,0,1

	
```

```
		  

		NW_003726465_1, Position,0,1000,2000,3000,4000,5000,6000,7000,8000,9000,10000,11000,12000,13000,14000,15000,16000,17000,18000,19000,20000,21000,22000,23000,24000,25000,26000,27000,28000,29000,30000,31000,32000,33000
NW_003726465_1,Count,0,0,0,0,0,0,0,0,0,0,2,0,0,0,0,0,0,0,0,0,0,0,0,0,0,0,5,0,0,0,7,0,4,0

	
```

```
		  

		NW_003726466_1, Position,0,1000,2000,3000,4000,5000,6000,7000,8000,9000,10000,11000,12000,13000,14000,15000,16000,17000,18000,19000,20000,21000,22000,23000,24000,25000,26000,27000,28000,29000,30000,31000,32000,33000,34000
NW_003726466_1,Count,0,0,0,2,1,0,0,0,1,3,0,0,0,0,0,2,1,3,0,0,0,0,3,0,0,0,0,2,0,0,0,0,0,0,0

	
```

```
		  

		NW_003726477_1, Position,0,1000,2000,3000,4000,5000,6000,7000,8000,9000,10000,11000,12000,13000,14000,15000,16000,17000,18000,19000,20000,21000,22000,23000,24000,25000,26000,27000,28000,29000,30000,31000,32000,33000,34000,35000,36000,37000,38000,39000,40000,41000,42000
NW_003726477_1,Count,0,0,0,0,0,0,0,0,0,0,0,0,0,0,0,0,0,0,0,2,0,0,0,0,0,0,0,0,0,0,0,0,0,0,0,0,0,0,0,0,0,0,0

	
```

```
		  

		NW_003726478_1, Position,0,1000,2000,3000,4000,5000,6000,7000,8000,9000,10000,11000,12000,13000,14000,15000,16000,17000,18000,19000,20000,21000,22000,23000,24000,25000,26000,27000,28000,29000,30000,31000
NW_003726478_1,Count,3,1,3,2,0,1,0,4,0,0,0,1,5,3,3,1,7,5,5,0,4,0,0,7,1,0,0,0,0,1,1,0

	
```

```
		  

		NW_003726481_1, Position,0,1000,2000,3000,4000,5000,6000,7000,8000,9000,10000,11000,12000,13000,14000,15000,16000,17000,18000,19000,20000,21000,22000,23000,24000,25000,26000,27000,28000,29000,30000,31000
NW_003726481_1,Count,8,11,12,5,11,15,8,16,8,21,17,16,10,7,6,6,21,13,10,12,11,6,2,4,7,5,7,6,6,14,9,3

	
```

```
		  

		NW_003726482_1, Position,0,1000,2000,3000,4000,5000,6000,7000,8000,9000,10000,11000,12000,13000,14000,15000,16000,17000,18000,19000,20000,21000,22000,23000,24000,25000,26000,27000,28000,29000,30000,31000,32000
NW_003726482_1,Count,0,3,1,0,0,0,0,0,0,0,0,0,0,0,0,0,0,0,0,0,0,0,0,0,0,0,0,0,0,0,0,0,0

	
```

```
		  

		NW_003726485_1, Position,0,1000,2000,3000,4000,5000,6000,7000,8000,9000,10000,11000,12000,13000,14000,15000,16000,17000,18000,19000,20000,21000,22000,23000,24000,25000,26000,27000,28000,29000,30000,31000,32000,33000,34000,35000,36000,37000,38000,39000,40000,41000,42000,43000,44000,45000,46000,47000,48000,49000,50000,51000,52000
NW_003726485_1,Count,0,0,0,0,0,0,0,0,0,0,0,0,0,0,0,0,0,0,0,0,0,0,0,0,0,0,0,0,0,0,0,0,0,0,0,0,0,0,0,0,0,0,3,0,0,0,0,0,0,0,0,0,0

	
```

```
		  

		NW_003726487_1, Position,0,1000,2000,3000,4000,5000,6000,7000,8000,9000,10000,11000,12000,13000,14000,15000,16000,17000,18000,19000,20000,21000,22000,23000,24000,25000,26000,27000,28000,29000,30000,31000
NW_003726487_1,Count,4,10,6,9,3,6,8,6,8,4,13,15,8,6,6,11,10,7,7,0,0,0,3,0,0,0,0,0,0,0,0,0

	
```

```
		  

		NW_003726488_1, Position,0,1000,2000,3000,4000,5000,6000,7000,8000,9000,10000,11000,12000,13000,14000,15000,16000,17000,18000,19000,20000,21000,22000,23000,24000,25000,26000,27000,28000,29000,30000
NW_003726488_1,Count,0,0,0,0,2,2,3,3,7,13,13,8,8,2,6,4,8,4,5,4,7,9,7,1,3,8,2,0,0,0,0

	
```

```
		  

		NW_003726489_1, Position,0,1000,2000,3000,4000,5000,6000,7000,8000,9000,10000,11000,12000,13000,14000,15000,16000,17000,18000,19000,20000,21000,22000,23000,24000,25000,26000,27000,28000,29000,30000,31000,32000,33000,34000,35000,36000,37000,38000,39000,40000,41000,42000,43000,44000,45000
NW_003726489_1,Count,0,0,0,0,0,0,0,0,0,0,0,0,0,0,0,0,0,0,0,0,0,2,0,0,0,0,0,0,0,0,0,0,0,0,0,0,4,0,0,0,0,0,0,0,0,0

	
```

```
		  

		NW_003726491_1, Position,0,1000,2000,3000,4000,5000,6000,7000,8000,9000,10000,11000,12000,13000,14000,15000,16000,17000,18000,19000,20000,21000,22000,23000,24000,25000,26000,27000,28000,29000,30000,31000,32000,33000,34000,35000,36000,37000,38000,39000
NW_003726491_1,Count,0,0,0,0,0,0,0,0,0,0,0,0,0,0,0,0,0,0,0,0,0,0,0,0,0,0,0,0,0,0,1,1,0,0,0,0,0,0,0,0

	
```

```
		  

		NW_003726492_1, Position,0,1000,2000,3000,4000,5000,6000,7000,8000,9000,10000,11000,12000,13000,14000,15000,16000,17000,18000,19000,20000,21000,22000,23000,24000,25000,26000,27000,28000,29000,30000,31000,32000,33000,34000,35000,36000,37000,38000,39000
NW_003726492_1,Count,0,0,0,0,0,0,0,0,0,0,0,0,0,0,0,0,0,0,0,0,0,0,0,1,0,0,0,0,0,0,0,0,0,0,0,0,0,0,0,0

	
```

```
		  

		NW_003726493_1, Position,0,1000,2000,3000,4000,5000,6000,7000,8000,9000,10000,11000,12000,13000,14000,15000,16000,17000,18000,19000,20000,21000,22000,23000,24000,25000,26000,27000,28000,29000,30000,31000
NW_003726493_1,Count,0,0,0,0,0,0,0,0,0,0,0,0,0,0,0,0,0,0,0,0,0,0,0,0,0,0,0,0,0,0,0,3

	
```

```
		  

		NW_003726497_1, Position,0,1000,2000,3000,4000,5000,6000,7000,8000,9000,10000,11000,12000,13000,14000,15000,16000,17000,18000,19000,20000,21000,22000,23000,24000,25000,26000,27000,28000,29000,30000
NW_003726497_1,Count,0,0,0,0,0,0,0,0,0,0,0,0,2,0,0,0,0,0,0,0,0,0,0,0,0,0,0,0,0,0,0

	
```

```
		  

		NW_003726498_1, Position,0,1000,2000,3000,4000,5000,6000,7000,8000,9000,10000,11000,12000,13000,14000,15000,16000,17000,18000,19000,20000,21000,22000,23000,24000,25000,26000,27000,28000,29000,30000
NW_003726498_1,Count,3,4,12,10,6,7,0,1,0,0,0,0,0,0,0,0,0,3,0,0,0,0,0,0,0,0,0,0,0,4,1

	
```

```
		  

		NW_003726501_1, Position,0,1000,2000,3000,4000,5000,6000,7000,8000,9000,10000,11000,12000,13000,14000,15000,16000,17000,18000,19000,20000,21000,22000,23000,24000,25000,26000,27000,28000,29000,30000,31000
NW_003726501_1,Count,0,0,0,2,7,9,11,5,0,0,0,3,0,0,0,0,0,0,0,0,2,9,2,6,0,5,5,11,7,3,10,3

	
```

```
		  

		NW_003726505_1, Position,0,1000,2000,3000,4000,5000,6000,7000,8000,9000,10000,11000,12000,13000,14000,15000,16000,17000,18000,19000,20000,21000,22000,23000,24000,25000,26000,27000,28000,29000,30000
NW_003726505_1,Count,0,0,0,0,15,12,4,15,8,8,11,11,8,13,9,4,7,5,8,9,12,4,11,10,7,6,1,16,7,0,0

	
```

```
		  

		NW_003726512_1, Position,0,1000,2000,3000,4000,5000,6000,7000,8000,9000,10000,11000,12000,13000,14000,15000,16000,17000,18000,19000,20000,21000,22000,23000,24000,25000,26000,27000,28000,29000,30000,31000,32000,33000,34000,35000,36000,37000,38000
NW_003726512_1,Count,0,0,0,0,0,0,0,0,0,0,0,0,0,0,0,0,0,0,0,0,0,0,0,0,0,0,0,0,0,0,0,0,0,0,0,0,0,2,2

	
```

```
		  

		NW_003726519_1, Position,0,100,200,300,400,500,600,700,800,900,1000,1100,1200,1300,1400,1500,1600,1700,1800,1900,2000,2100,2200,2300,2400,2500,2600,2700,2800,2900,3000,3100,3200,3300,3400,3500,3600,3700,3800,3900,4000,4100,4200,4300,4400,4500,4600,4700,4800,4900,5000,5100,5200,5300,5400,5500,5600,5700,5800,5900,6000,6100,6200,6300,6400,6500,6600,6700,6800,6900,7000,7100,7200,7300,7400,7500,7600,7700,7800,7900,8000,8100,8200,8300,8400,8500,8600,8700,8800,8900,9000,9100,9200,9300,9400,9500,9600,9700,9800,9900,10000,10100,10200,10300,10400,10500,10600,10700,10800,10900,11000,11100,11200,11300,11400,11500,11600,11700,11800,11900,12000,12100,12200,12300,12400,12500,12600,12700,12800,12900,13000,13100,13200,13300,13400,13500,13600,13700,13800,13900,14000,14100,14200,14300,14400,14500,14600,14700,14800,14900,15000,15100,15200,15300,15400,15500,15600,15700,15800,15900,16000,16100,16200,16300,16400,16500,16600,16700,16800,16900,17000,17100,17200,17300,17400,17500,17600,17700,17800,17900,18000,18100,18200,18300,18400,18500,18600,18700,18800,18900,19000,19100,19200,19300,19400,19500,19600,19700,19800,19900,20000,20100,20200,20300,20400,20500,20600,20700,20800,20900,21000,21100,21200,21300,21400,21500,21600,21700,21800,21900,22000,22100,22200,22300,22400,22500,22600,22700,22800,22900,23000,23100,23200,23300,23400,23500,23600,23700,23800,23900,24000,24100,24200,24300,24400,24500,24600,24700,24800,24900,25000,25100,25200,25300,25400,25500,25600,25700,25800,25900,26000,26100,26200,26300,26400,26500,26600,26700,26800,26900,27000,27100,27200,27300,27400,27500,27600,27700,27800,27900,28000,28100,28200,28300,28400,28500,28600,28700,28800,28900,29000,29100,29200,29300,29400,29500,29600,29700
NW_003726519_1,Count,0,0,0,0,0,0,0,0,0,0,0,0,0,0,0,0,0,0,0,0,0,0,0,0,0,0,0,0,1,3,1,0,2,0,1,3,0,0,0,0,0,0,0,0,0,0,0,0,0,0,0,0,0,0,1,1,0,0,0,0,0,0,0,0,0,0,0,0,0,0,0,0,0,0,0,0,0,0,0,0,0,0,0,0,1,1,0,0,0,0,0,0,0,0,0,0,0,0,0,0,0,0,0,0,0,0,0,0,0,0,0,0,0,0,0,0,0,0,0,0,0,0,0,0,3,0,0,0,1,1,0,0,1,0,0,0,0,0,0,0,0,0,0,0,0,0,0,0,0,0,0,0,0,0,0,0,0,0,0,0,0,0,0,0,0,0,0,0,0,0,0,0,0,0,0,0,0,0,0,0,0,0,0,0,0,0,0,1,2,0,0,0,0,0,0,0,0,0,0,0,0,0,0,0,0,0,0,0,0,0,0,0,0,0,0,0,0,0,0,0,0,3,0,0,3,0,0,0,0,0,0,0,0,0,0,0,0,0,0,0,0,0,0,0,0,0,0,0,0,0,0,0,0,0,0,0,0,0,0,0,0,0,0,0,0,0,0,0,0,0,0,0,0,0,0,0,0,0,0,0,0,0,0,0,0,0,0,0,0,0,0,0,0,0,0,0,0,0

	
```

```
		  

		NW_003726520_1, Position,0,100,200,300,400,500,600,700,800,900,1000,1100,1200,1300,1400,1500,1600,1700,1800,1900,2000,2100,2200,2300,2400,2500,2600,2700,2800,2900,3000,3100,3200,3300,3400,3500,3600,3700,3800,3900,4000,4100,4200,4300,4400,4500,4600,4700,4800,4900,5000,5100,5200,5300,5400,5500,5600,5700,5800,5900,6000,6100,6200,6300,6400,6500,6600,6700,6800,6900,7000,7100,7200,7300,7400,7500,7600,7700,7800,7900,8000,8100,8200,8300,8400,8500,8600,8700,8800,8900,9000,9100,9200,9300,9400,9500,9600,9700,9800,9900,10000,10100,10200,10300,10400,10500,10600,10700,10800,10900,11000,11100,11200,11300,11400,11500,11600,11700,11800,11900,12000,12100,12200,12300,12400,12500,12600,12700,12800,12900,13000,13100,13200,13300,13400,13500,13600,13700,13800,13900,14000,14100,14200,14300,14400,14500,14600,14700,14800,14900,15000,15100,15200,15300,15400,15500,15600,15700,15800,15900,16000,16100,16200,16300,16400,16500,16600,16700,16800,16900,17000,17100,17200,17300,17400,17500,17600,17700,17800,17900,18000,18100,18200,18300,18400,18500,18600,18700,18800,18900,19000,19100,19200,19300,19400,19500,19600,19700,19800,19900,20000,20100,20200,20300,20400,20500,20600,20700,20800,20900,21000,21100,21200,21300,21400,21500,21600,21700,21800,21900,22000,22100,22200,22300,22400,22500,22600,22700,22800,22900,23000,23100,23200,23300,23400,23500,23600,23700,23800,23900,24000,24100,24200,24300,24400,24500,24600,24700,24800,24900,25000,25100,25200,25300,25400,25500,25600,25700,25800,25900,26000,26100,26200,26300,26400,26500,26600,26700,26800,26900,27000,27100,27200,27300,27400,27500,27600,27700,27800,27900,28000,28100,28200,28300,28400,28500,28600,28700,28800,28900,29000,29100,29200,29300
NW_003726520_1,Count,2,1,0,0,1,0,2,0,5,1,0,0,0,0,0,0,0,0,0,0,0,0,0,0,0,0,0,0,0,0,0,0,0,0,0,0,2,0,0,1,1,2,0,1,1,0,0,0,0,0,0,0,0,0,2,0,2,0,0,1,1,2,2,1,1,1,3,1,2,1,1,2,1,2,0,0,0,0,0,0,2,0,0,0,0,0,0,0,0,0,0,0,0,0,0,0,0,0,0,0,1,0,2,2,0,0,0,0,0,2,1,1,1,1,0,0,0,0,0,0,0,0,0,0,0,0,0,0,0,0,0,0,1,3,1,0,0,1,1,1,0,0,0,0,0,2,0,0,1,0,1,1,1,2,0,0,3,0,0,0,0,0,0,1,2,0,1,1,1,1,1,0,0,1,0,0,0,0,0,0,0,0,0,0,0,0,0,0,0,0,1,0,1,0,2,0,3,0,0,0,1,4,0,0,0,1,0,0,0,0,0,0,1,2,1,1,1,0,0,0,0,0,0,0,0,0,1,1,2,0,0,0,1,2,3,1,0,0,1,1,3,1,2,0,0,2,2,0,2,1,0,3,1,2,3,1,1,3,0,0,2,1,0,0,0,1,1,0,0,0,1,0,0,1,0,0,2,0,0,0,0,0,2,0,3,0,0,0,0,1,1,0,0,0

	
```

```
		  

		NW_003726523_1, Position,0,100,200,300,400,500,600,700,800,900,1000,1100,1200,1300,1400,1500,1600,1700,1800,1900,2000,2100,2200,2300,2400,2500,2600,2700,2800,2900,3000,3100,3200,3300,3400,3500,3600,3700,3800,3900,4000,4100,4200,4300,4400,4500,4600,4700,4800,4900,5000,5100,5200,5300,5400,5500,5600,5700,5800,5900,6000,6100,6200,6300,6400,6500,6600,6700,6800,6900,7000,7100,7200,7300,7400,7500,7600,7700,7800,7900,8000,8100,8200,8300,8400,8500,8600,8700,8800,8900,9000,9100,9200,9300,9400,9500,9600,9700,9800,9900,10000,10100,10200,10300,10400,10500,10600,10700,10800,10900,11000,11100,11200,11300,11400,11500,11600,11700,11800,11900,12000,12100,12200,12300,12400,12500,12600,12700,12800,12900,13000,13100,13200,13300,13400,13500,13600,13700,13800,13900,14000,14100,14200,14300,14400,14500,14600,14700,14800,14900,15000,15100,15200,15300,15400,15500,15600,15700,15800,15900,16000,16100,16200,16300,16400,16500,16600,16700,16800,16900,17000,17100,17200,17300,17400,17500,17600,17700,17800,17900,18000,18100,18200,18300,18400,18500,18600,18700,18800,18900,19000,19100,19200,19300,19400,19500,19600,19700,19800,19900,20000,20100,20200,20300,20400,20500,20600,20700,20800,20900,21000,21100,21200,21300,21400,21500,21600,21700,21800,21900,22000,22100,22200,22300,22400,22500,22600,22700,22800,22900,23000,23100,23200,23300,23400,23500,23600,23700,23800,23900,24000,24100,24200,24300,24400,24500,24600,24700,24800,24900,25000,25100,25200,25300,25400,25500,25600,25700,25800,25900,26000,26100,26200,26300,26400,26500,26600,26700,26800,26900,27000,27100,27200,27300,27400,27500,27600,27700,27800,27900,28000,28100,28200,28300,28400,28500,28600,28700,28800,28900,29000,29100,29200,29300,29400,29500,29600,29700
NW_003726523_1,Count,0,0,0,0,0,0,0,0,0,0,0,0,0,0,0,0,0,1,0,2,0,0,0,0,1,0,1,2,0,0,0,0,0,0,0,0,0,1,0,0,0,0,0,0,0,0,0,0,0,0,0,0,0,0,0,0,0,0,0,0,0,0,0,0,0,0,0,0,0,0,0,0,0,0,0,0,0,0,0,0,0,0,0,0,0,0,0,0,0,0,0,0,0,0,0,2,1,0,0,2,1,0,2,0,1,2,1,1,2,1,3,2,0,0,0,0,0,0,0,0,0,0,0,0,0,0,2,1,1,2,0,0,0,0,0,0,0,0,0,0,0,0,0,0,0,0,0,0,0,0,0,0,0,0,0,0,1,0,1,0,0,0,0,0,0,0,0,0,0,0,0,0,0,0,0,0,0,0,0,0,0,0,0,0,0,0,0,0,0,0,0,0,0,0,0,0,0,0,0,0,0,2,4,0,0,1,0,0,0,0,0,0,0,0,0,0,0,0,0,0,0,0,0,0,1,0,0,0,0,0,0,0,0,0,0,0,0,0,2,1,3,2,0,0,0,0,0,0,0,0,0,2,0,0,0,0,0,0,0,1,0,0,0,0,0,0,0,0,0,0,0,0,0,0,0,0,1,0,0,0,0,0,0,0,0,0,0,0,0,0,0,0,0,0,0,0,0,0

	
```

```
		  

		NW_003726526_1, Position,0,1000,2000,3000,4000,5000,6000,7000,8000,9000,10000,11000,12000,13000,14000,15000,16000,17000,18000,19000,20000,21000,22000,23000,24000,25000,26000,27000,28000,29000,30000
NW_003726526_1,Count,8,5,6,6,16,11,11,7,16,6,8,11,4,10,3,14,11,8,10,8,5,7,12,5,11,8,7,6,6,4,0

	
```

```
		  

		NW_003726528_1, Position,0,100,200,300,400,500,600,700,800,900,1000,1100,1200,1300,1400,1500,1600,1700,1800,1900,2000,2100,2200,2300,2400,2500,2600,2700,2800,2900,3000,3100,3200,3300,3400,3500,3600,3700,3800,3900,4000,4100,4200,4300,4400,4500,4600,4700,4800,4900,5000,5100,5200,5300,5400,5500,5600,5700,5800,5900,6000,6100,6200,6300,6400,6500,6600,6700,6800,6900,7000,7100,7200,7300,7400,7500,7600,7700,7800,7900,8000,8100,8200,8300,8400,8500,8600,8700,8800,8900,9000,9100,9200,9300,9400,9500,9600,9700,9800,9900,10000,10100,10200,10300,10400,10500,10600,10700,10800,10900,11000,11100,11200,11300,11400,11500,11600,11700,11800,11900,12000,12100,12200,12300,12400,12500,12600,12700,12800,12900,13000,13100,13200,13300,13400,13500,13600,13700,13800,13900,14000,14100,14200,14300,14400,14500,14600,14700,14800,14900,15000,15100,15200,15300,15400,15500,15600,15700,15800,15900,16000,16100,16200,16300,16400,16500,16600,16700,16800,16900,17000,17100,17200,17300,17400,17500,17600,17700,17800,17900,18000,18100,18200,18300,18400,18500,18600,18700,18800,18900,19000,19100,19200,19300,19400,19500,19600,19700,19800,19900,20000,20100,20200,20300,20400,20500,20600,20700,20800,20900,21000,21100,21200,21300,21400,21500,21600,21700,21800,21900,22000,22100,22200,22300,22400,22500,22600,22700,22800,22900,23000,23100,23200,23300,23400,23500,23600,23700,23800,23900,24000,24100,24200,24300,24400,24500,24600,24700,24800,24900,25000,25100,25200,25300,25400,25500,25600,25700,25800,25900,26000,26100,26200,26300,26400,26500,26600,26700,26800,26900,27000,27100,27200,27300,27400,27500,27600,27700,27800,27900,28000,28100,28200,28300,28400,28500,28600,28700,28800,28900,29000,29100,29200,29300
NW_003726528_1,Count,0,0,0,0,0,0,0,0,0,0,0,0,0,0,0,0,0,0,0,0,0,0,0,0,0,0,0,0,0,0,0,0,0,0,0,0,0,0,0,0,0,0,0,0,0,0,0,0,0,0,0,0,0,0,0,0,0,0,0,0,0,0,0,0,0,0,0,0,0,0,0,0,0,0,0,0,0,0,0,0,0,0,0,0,0,0,0,0,0,0,0,0,0,0,0,0,0,0,0,0,0,0,0,0,0,0,0,0,0,0,0,0,0,0,0,0,0,0,0,0,0,0,0,0,0,0,0,0,0,0,0,0,0,0,0,0,0,0,0,0,0,0,0,0,0,0,0,0,0,0,0,0,0,0,0,0,0,0,0,0,0,0,0,0,0,0,0,0,0,0,0,0,0,0,0,0,0,0,0,0,0,0,0,0,0,0,0,0,0,0,0,0,0,0,0,0,0,0,0,0,0,0,0,0,0,0,0,0,0,0,0,0,0,0,0,0,0,0,0,0,0,0,0,0,0,0,0,0,0,0,0,0,0,0,0,0,0,0,0,0,0,0,0,0,0,0,1,1,0,0,0,0,0,0,0,0,0,0,0,0,0,0,0,0,0,0,0,0,0,0,0,0,0,0,0,0,0,0,0,0,0,0,0,0,0,0,0,0,0,2,0,0,0,0

	
```

```
		  

		NW_003726529_1, Position,0,100,200,300,400,500,600,700,800,900,1000,1100,1200,1300,1400,1500,1600,1700,1800,1900,2000,2100,2200,2300,2400,2500,2600,2700,2800,2900,3000,3100,3200,3300,3400,3500,3600,3700,3800,3900,4000,4100,4200,4300,4400,4500,4600,4700,4800,4900,5000,5100,5200,5300,5400,5500,5600,5700,5800,5900,6000,6100,6200,6300,6400,6500,6600,6700,6800,6900,7000,7100,7200,7300,7400,7500,7600,7700,7800,7900,8000,8100,8200,8300,8400,8500,8600,8700,8800,8900,9000,9100,9200,9300,9400,9500,9600,9700,9800,9900,10000,10100,10200,10300,10400,10500,10600,10700,10800,10900,11000,11100,11200,11300,11400,11500,11600,11700,11800,11900,12000,12100,12200,12300,12400,12500,12600,12700,12800,12900,13000,13100,13200,13300,13400,13500,13600,13700,13800,13900,14000,14100,14200,14300,14400,14500,14600,14700,14800,14900,15000,15100,15200,15300,15400,15500,15600,15700,15800,15900,16000,16100,16200,16300,16400,16500,16600,16700,16800,16900,17000,17100,17200,17300,17400,17500,17600,17700,17800,17900,18000,18100,18200,18300,18400,18500,18600,18700,18800,18900,19000,19100,19200,19300,19400,19500,19600,19700,19800,19900,20000,20100,20200,20300,20400,20500,20600,20700,20800,20900,21000,21100,21200,21300,21400,21500,21600,21700,21800,21900,22000,22100,22200,22300,22400,22500,22600,22700,22800,22900,23000,23100,23200,23300,23400,23500,23600,23700,23800,23900,24000,24100,24200,24300,24400,24500,24600,24700,24800,24900,25000,25100,25200,25300,25400,25500,25600,25700,25800,25900,26000,26100,26200,26300,26400,26500,26600,26700,26800,26900,27000,27100,27200,27300,27400,27500,27600,27700,27800,27900,28000,28100,28200,28300,28400,28500,28600,28700,28800,28900
NW_003726529_1,Count,0,0,0,0,0,0,0,0,0,0,0,0,0,3,3,0,3,0,0,0,0,0,0,0,1,0,0,0,0,0,0,0,2,1,0,0,0,0,0,0,0,0,0,0,0,0,0,0,0,0,0,0,0,0,0,1,1,0,0,0,0,0,0,0,0,0,0,0,0,0,0,0,0,0,0,0,1,3,0,0,0,0,0,0,0,0,0,1,0,2,0,0,0,0,0,0,0,0,0,0,0,0,0,0,0,0,0,0,0,0,0,0,0,0,0,0,0,0,0,0,0,0,0,0,0,0,0,0,0,0,0,0,0,0,0,0,0,0,0,0,0,0,0,0,0,0,0,0,0,0,0,0,1,0,0,0,0,0,0,0,0,0,0,0,0,0,0,0,0,0,0,0,3,1,1,3,0,0,0,0,0,0,0,0,0,1,1,0,0,0,0,0,0,0,0,0,0,0,0,0,0,0,0,0,0,0,0,0,2,1,0,0,0,0,0,0,0,0,0,0,0,0,0,0,0,2,1,1,0,0,0,0,0,0,0,0,0,0,0,0,0,0,0,0,0,0,0,0,0,0,0,0,0,0,0,0,0,0,0,0,0,0,0,0,0,0,0,0,0,0,0,0,0,0,0,0,0,0,0,0,0,0,0,0,0,0,0,0,0,0

	
```

```
		  

		NW_003726530_1, Position,0,1000,2000,3000,4000,5000,6000,7000,8000,9000,10000,11000,12000,13000,14000,15000,16000,17000,18000,19000,20000,21000,22000,23000,24000,25000,26000,27000,28000,29000,30000,31000,32000
NW_003726530_1,Count,10,12,6,5,2,4,2,16,13,14,9,2,18,22,13,11,4,7,4,7,3,8,12,23,9,9,7,1,0,1,13,5,9

	
```

```
		  

		NW_003726532_1, Position,0,100,200,300,400,500,600,700,800,900,1000,1100,1200,1300,1400,1500,1600,1700,1800,1900,2000,2100,2200,2300,2400,2500,2600,2700,2800,2900,3000,3100,3200,3300,3400,3500,3600,3700,3800,3900,4000,4100,4200,4300,4400,4500,4600,4700,4800,4900,5000,5100,5200,5300,5400,5500,5600,5700,5800,5900,6000,6100,6200,6300,6400,6500,6600,6700,6800,6900,7000,7100,7200,7300,7400,7500,7600,7700,7800,7900,8000,8100,8200,8300,8400,8500,8600,8700,8800,8900,9000,9100,9200,9300,9400,9500,9600,9700,9800,9900,10000,10100,10200,10300,10400,10500,10600,10700,10800,10900,11000,11100,11200,11300,11400,11500,11600,11700,11800,11900,12000,12100,12200,12300,12400,12500,12600,12700,12800,12900,13000,13100,13200,13300,13400,13500,13600,13700,13800,13900,14000,14100,14200,14300,14400,14500,14600,14700,14800,14900,15000,15100,15200,15300,15400,15500,15600,15700,15800,15900,16000,16100,16200,16300,16400,16500,16600,16700,16800,16900,17000,17100,17200,17300,17400,17500,17600,17700,17800,17900,18000,18100,18200,18300,18400,18500,18600,18700,18800,18900,19000,19100,19200,19300,19400,19500,19600,19700,19800,19900,20000,20100,20200,20300,20400,20500,20600,20700,20800,20900,21000,21100,21200,21300,21400,21500,21600,21700,21800,21900,22000,22100,22200,22300,22400,22500,22600,22700,22800,22900,23000,23100,23200,23300,23400,23500,23600,23700,23800,23900,24000,24100,24200,24300,24400,24500,24600,24700,24800,24900,25000,25100,25200,25300,25400,25500,25600,25700,25800,25900,26000,26100,26200,26300,26400,26500,26600,26700,26800,26900,27000,27100,27200,27300,27400,27500,27600,27700,27800,27900,28000,28100,28200,28300,28400,28500,28600,28700,28800,28900
NW_003726532_1,Count,0,0,0,0,0,0,0,0,0,0,0,0,0,0,0,0,0,0,0,0,0,0,0,0,0,0,0,0,0,0,0,0,0,0,0,0,0,0,0,0,0,0,0,0,0,0,0,0,0,0,0,0,0,0,0,0,0,0,0,0,2,0,2,1,1,0,0,0,0,1,2,0,0,0,0,0,0,0,0,1,0,0,0,0,1,0,0,0,0,0,0,0,0,0,0,0,0,0,0,0,0,0,0,0,0,0,0,0,0,0,1,0,1,0,1,0,1,1,1,0,2,0,2,1,0,0,1,0,1,1,0,0,0,2,0,0,0,1,0,0,2,3,0,0,1,2,0,2,1,0,0,0,1,0,0,1,0,0,0,0,1,1,1,0,1,2,0,1,0,1,2,0,2,0,0,0,1,2,0,1,2,1,1,1,0,0,0,0,1,0,1,1,0,1,0,0,0,1,2,2,2,0,1,1,3,0,2,0,0,0,0,0,0,0,0,0,0,0,0,0,0,0,0,0,0,0,0,0,0,0,0,0,0,0,0,0,0,0,0,0,0,0,0,0,0,0,0,0,0,0,0,0,0,0,0,0,0,0,0,0,0,0,0,0,0,0,0,3,0,1,0,0,0,0,0,0,0,0,0,0,0,0,0,0,0,0,2,3,1,1

	
```

```
		  

		NW_003726534_1, Position,0,100,200,300,400,500,600,700,800,900,1000,1100,1200,1300,1400,1500,1600,1700,1800,1900,2000,2100,2200,2300,2400,2500,2600,2700,2800,2900,3000,3100,3200,3300,3400,3500,3600,3700,3800,3900,4000,4100,4200,4300,4400,4500,4600,4700,4800,4900,5000,5100,5200,5300,5400,5500,5600,5700,5800,5900,6000,6100,6200,6300,6400,6500,6600,6700,6800,6900,7000,7100,7200,7300,7400,7500,7600,7700,7800,7900,8000,8100,8200,8300,8400,8500,8600,8700,8800,8900,9000,9100,9200,9300,9400,9500,9600,9700,9800,9900,10000,10100,10200,10300,10400,10500,10600,10700,10800,10900,11000,11100,11200,11300,11400,11500,11600,11700,11800,11900,12000,12100,12200,12300,12400,12500,12600,12700,12800,12900,13000,13100,13200,13300,13400,13500,13600,13700,13800,13900,14000,14100,14200,14300,14400,14500,14600,14700,14800,14900,15000,15100,15200,15300,15400,15500,15600,15700,15800,15900,16000,16100,16200,16300,16400,16500,16600,16700,16800,16900,17000,17100,17200,17300,17400,17500,17600,17700,17800,17900,18000,18100,18200,18300,18400,18500,18600,18700,18800,18900,19000,19100,19200,19300,19400,19500,19600,19700,19800,19900,20000,20100,20200,20300,20400,20500,20600,20700,20800,20900,21000,21100,21200,21300,21400,21500,21600,21700,21800,21900,22000,22100,22200,22300,22400,22500,22600,22700,22800,22900,23000,23100,23200,23300,23400,23500,23600,23700,23800,23900,24000,24100,24200,24300,24400,24500,24600,24700,24800,24900,25000,25100,25200,25300,25400,25500,25600,25700,25800,25900,26000,26100,26200,26300,26400,26500,26600,26700,26800,26900,27000,27100,27200,27300,27400,27500,27600,27700,27800,27900,28000,28100,28200,28300,28400,28500,28600,28700
NW_003726534_1,Count,0,1,1,2,2,0,2,1,1,2,2,0,0,2,1,0,1,1,3,0,1,1,0,0,1,0,0,1,2,1,2,3,0,0,0,0,0,1,0,0,1,2,1,2,0,0,0,0,0,0,0,0,0,0,0,0,0,1,1,0,2,2,2,1,0,0,0,0,1,0,0,0,0,0,0,0,0,2,1,0,0,0,0,0,0,0,0,0,0,0,4,0,0,0,0,0,0,0,0,0,0,0,0,0,2,2,0,0,0,0,0,0,0,0,0,0,0,0,2,1,0,0,0,0,0,0,0,2,2,0,0,0,0,0,0,0,0,0,0,0,0,0,0,3,0,0,0,0,0,0,0,0,0,0,0,3,1,1,0,0,0,0,0,2,0,0,0,0,0,0,1,1,1,1,1,0,0,0,0,0,0,0,0,0,0,0,0,0,0,0,0,0,0,0,0,0,0,0,0,0,0,0,0,0,0,0,0,0,0,0,0,0,0,0,0,0,0,0,0,0,0,0,0,0,0,0,0,1,0,4,3,1,3,0,1,1,5,0,0,0,0,0,0,0,3,2,0,0,1,0,0,0,0,0,1,0,2,2,0,0,0,0,0,0,0,0,0,0,0,0,0,0,0,0,0,0,0,0,1,0,0,0,0,0,0,0,0,0

	
```

```
		  

		NW_003726540_1, Position,0,100,200,300,400,500,600,700,800,900,1000,1100,1200,1300,1400,1500,1600,1700,1800,1900,2000,2100,2200,2300,2400,2500,2600,2700,2800,2900,3000,3100,3200,3300,3400,3500,3600,3700,3800,3900,4000,4100,4200,4300,4400,4500,4600,4700,4800,4900,5000,5100,5200,5300,5400,5500,5600,5700,5800,5900,6000,6100,6200,6300,6400,6500,6600,6700,6800,6900,7000,7100,7200,7300,7400,7500,7600,7700,7800,7900,8000,8100,8200,8300,8400,8500,8600,8700,8800,8900,9000,9100,9200,9300,9400,9500,9600,9700,9800,9900,10000,10100,10200,10300,10400,10500,10600,10700,10800,10900,11000,11100,11200,11300,11400,11500,11600,11700,11800,11900,12000,12100,12200,12300,12400,12500,12600,12700,12800,12900,13000,13100,13200,13300,13400,13500,13600,13700,13800,13900,14000,14100,14200,14300,14400,14500,14600,14700,14800,14900,15000,15100,15200,15300,15400,15500,15600,15700,15800,15900,16000,16100,16200,16300,16400,16500,16600,16700,16800,16900,17000,17100,17200,17300,17400,17500,17600,17700,17800,17900,18000,18100,18200,18300,18400,18500,18600,18700,18800,18900,19000,19100,19200,19300,19400,19500,19600,19700,19800,19900,20000,20100,20200,20300,20400,20500,20600,20700,20800,20900,21000,21100,21200,21300,21400,21500,21600,21700,21800,21900,22000,22100,22200,22300,22400,22500,22600,22700,22800,22900,23000,23100,23200,23300,23400,23500,23600,23700,23800,23900,24000,24100,24200,24300,24400,24500,24600,24700,24800,24900,25000,25100,25200,25300,25400,25500,25600,25700,25800,25900,26000,26100,26200,26300,26400,26500,26600,26700,26800,26900,27000,27100,27200,27300,27400,27500,27600,27700,27800,27900,28000,28100,28200,28300,28400,28500,28600,28700,28800,28900,29000,29100
NW_003726540_1,Count,0,0,0,0,0,0,0,0,0,0,0,0,0,0,0,0,0,0,0,0,0,0,0,0,0,0,0,0,0,0,0,0,0,0,0,0,0,0,0,0,0,0,0,0,0,0,0,0,0,0,0,0,0,0,0,0,0,0,0,0,0,0,0,0,0,0,0,0,0,0,0,0,0,0,0,0,0,0,0,0,0,0,0,0,0,0,0,0,0,0,0,0,0,0,0,0,0,0,0,0,0,0,0,0,0,0,0,0,0,0,0,0,0,0,0,0,0,0,0,0,0,0,0,0,0,0,0,0,0,0,0,0,0,0,0,0,0,0,0,0,0,0,0,0,0,0,0,0,0,0,0,0,0,0,0,0,0,0,0,0,0,0,0,0,0,0,0,0,0,0,0,0,0,0,0,0,0,0,0,0,0,0,0,0,0,0,0,0,0,0,0,0,0,0,0,0,0,0,0,0,0,0,0,0,0,0,0,0,0,0,0,0,0,0,0,0,0,0,0,0,0,0,0,0,0,0,0,0,0,0,0,0,0,0,0,0,1,0,0,0,0,0,0,0,0,0,0,0,0,0,0,0,0,0,0,0,0,0,0,0,0,0,0,0,0,0,0,0,0,0,0,0,0,0,0,0,0,0,0,0,0,0,0,0,0,0,0,0,0,0,0,0

	
```

```
		  

		NW_003726545_1, Position,0,1000,2000,3000,4000,5000,6000,7000,8000,9000,10000,11000,12000,13000,14000,15000,16000,17000,18000,19000,20000,21000,22000,23000,24000,25000,26000,27000,28000,29000,30000,31000,32000,33000,34000,35000,36000,37000,38000,39000,40000,41000,42000,43000,44000,45000,46000,47000,48000,49000,50000,51000
NW_003726545_1,Count,9,8,0,0,0,0,0,0,0,17,8,0,0,0,0,0,0,0,0,0,0,0,0,0,0,0,0,0,0,0,0,0,0,0,0,7,0,0,0,0,0,0,0,0,0,0,0,0,0,0,0,0

	
```

```
		  

		NW_003726547_1, Position,0,100,200,300,400,500,600,700,800,900,1000,1100,1200,1300,1400,1500,1600,1700,1800,1900,2000,2100,2200,2300,2400,2500,2600,2700,2800,2900,3000,3100,3200,3300,3400,3500,3600,3700,3800,3900,4000,4100,4200,4300,4400,4500,4600,4700,4800,4900,5000,5100,5200,5300,5400,5500,5600,5700,5800,5900,6000,6100,6200,6300,6400,6500,6600,6700,6800,6900,7000,7100,7200,7300,7400,7500,7600,7700,7800,7900,8000,8100,8200,8300,8400,8500,8600,8700,8800,8900,9000,9100,9200,9300,9400,9500,9600,9700,9800,9900,10000,10100,10200,10300,10400,10500,10600,10700,10800,10900,11000,11100,11200,11300,11400,11500,11600,11700,11800,11900,12000,12100,12200,12300,12400,12500,12600,12700,12800,12900,13000,13100,13200,13300,13400,13500,13600,13700,13800,13900,14000,14100,14200,14300,14400,14500,14600,14700,14800,14900,15000,15100,15200,15300,15400,15500,15600,15700,15800,15900,16000,16100,16200,16300,16400,16500,16600,16700,16800,16900,17000,17100,17200,17300,17400,17500,17600,17700,17800,17900,18000,18100,18200,18300,18400,18500,18600,18700,18800,18900,19000,19100,19200,19300,19400,19500,19600,19700,19800,19900,20000,20100,20200,20300,20400,20500,20600,20700,20800,20900,21000,21100,21200,21300,21400,21500,21600,21700,21800,21900,22000,22100,22200,22300,22400,22500,22600,22700,22800,22900,23000,23100,23200,23300,23400,23500,23600,23700,23800,23900,24000,24100,24200,24300,24400,24500,24600,24700,24800,24900,25000,25100,25200,25300,25400,25500,25600,25700,25800,25900,26000,26100,26200,26300,26400,26500,26600,26700,26800,26900,27000,27100,27200,27300,27400,27500,27600,27700,27800,27900,28000,28100,28200,28300
NW_003726547_1,Count,0,0,0,0,0,0,0,0,0,0,0,0,0,0,0,0,0,0,0,0,0,0,0,0,0,0,0,0,0,0,0,0,0,0,0,0,0,0,0,0,0,0,0,0,0,0,0,0,0,0,0,0,0,0,0,0,0,0,0,0,0,0,0,0,0,0,0,0,0,0,0,0,0,0,0,0,0,0,0,0,0,0,0,0,0,0,0,0,0,0,0,0,0,0,0,0,0,0,0,0,0,0,0,0,0,0,0,0,0,0,0,0,0,0,0,0,0,0,0,0,0,0,0,0,0,0,0,0,0,0,0,0,0,0,0,0,0,0,0,0,0,0,0,0,0,0,0,0,0,0,0,0,0,0,0,0,0,0,0,0,0,0,0,0,0,0,0,0,0,0,0,0,0,0,0,0,0,0,0,0,0,0,3,0,0,0,0,0,0,0,0,0,0,0,0,0,0,0,0,0,0,0,0,0,0,0,0,0,0,0,0,0,0,0,0,0,0,0,0,0,0,0,0,0,0,0,0,0,0,0,0,0,0,0,0,0,0,0,0,0,0,0,0,0,0,0,0,0,0,0,0,0,0,0,0,0,0,0,0,0,0,0,0,0,0,0,0,0,0,0,0,0,0,0,0,0,0,0,0,0,0,0,0,0

	
```

```
		  

		NW_003726549_1, Position,0,100,200,300,400,500,600,700,800,900,1000,1100,1200,1300,1400,1500,1600,1700,1800,1900,2000,2100,2200,2300,2400,2500,2600,2700,2800,2900,3000,3100,3200,3300,3400,3500,3600,3700,3800,3900,4000,4100,4200,4300,4400,4500,4600,4700,4800,4900,5000,5100,5200,5300,5400,5500,5600,5700,5800,5900,6000,6100,6200,6300,6400,6500,6600,6700,6800,6900,7000,7100,7200,7300,7400,7500,7600,7700,7800,7900,8000,8100,8200,8300,8400,8500,8600,8700,8800,8900,9000,9100,9200,9300,9400,9500,9600,9700,9800,9900,10000,10100,10200,10300,10400,10500,10600,10700,10800,10900,11000,11100,11200,11300,11400,11500,11600,11700,11800,11900,12000,12100,12200,12300,12400,12500,12600,12700,12800,12900,13000,13100,13200,13300,13400,13500,13600,13700,13800,13900,14000,14100,14200,14300,14400,14500,14600,14700,14800,14900,15000,15100,15200,15300,15400,15500,15600,15700,15800,15900,16000,16100,16200,16300,16400,16500,16600,16700,16800,16900,17000,17100,17200,17300,17400,17500,17600,17700,17800,17900,18000,18100,18200,18300,18400,18500,18600,18700,18800,18900,19000,19100,19200,19300,19400,19500,19600,19700,19800,19900,20000,20100,20200,20300,20400,20500,20600,20700,20800,20900,21000,21100,21200,21300,21400,21500,21600,21700,21800,21900,22000,22100,22200,22300,22400,22500,22600,22700,22800,22900,23000,23100,23200,23300,23400,23500,23600,23700,23800,23900,24000,24100,24200,24300,24400,24500,24600,24700,24800,24900,25000,25100,25200,25300,25400,25500,25600,25700,25800,25900,26000,26100,26200,26300,26400,26500,26600,26700,26800,26900,27000,27100,27200,27300,27400,27500,27600,27700,27800,27900,28000,28100,28200
NW_003726549_1,Count,0,0,0,2,0,1,0,0,2,1,3,1,1,1,0,0,0,1,0,0,0,0,2,1,1,1,0,0,0,1,0,0,0,0,1,0,0,0,0,0,2,0,1,0,1,0,0,0,0,1,0,1,3,0,0,0,0,1,1,0,1,0,1,0,0,0,0,0,2,2,1,0,1,1,0,1,0,0,1,2,0,0,0,3,1,1,0,1,0,0,1,0,1,0,1,0,0,1,0,1,0,0,1,0,0,0,1,0,0,0,0,0,2,1,0,0,1,1,2,1,0,1,1,0,1,1,1,1,1,0,1,3,0,1,0,1,0,0,1,2,1,0,0,1,1,1,2,0,1,1,1,0,1,1,1,1,1,0,1,1,0,1,1,2,0,0,1,1,1,0,0,0,1,1,1,1,0,0,0,0,0,4,0,1,0,0,0,0,3,1,0,0,0,0,0,2,0,0,0,0,0,0,0,0,0,2,1,2,1,1,4,0,2,1,1,1,1,0,0,0,0,0,0,0,1,1,1,1,1,0,0,2,0,0,0,3,0,3,0,1,0,0,0,0,0,2,0,2,1,1,1,0,0,0,1,1,1,0,1,0,2,0,0,1,1,0,1,0,1,0,1,0,0,1,0,0,0,0,0,0,0,0,0

	
```

```
		  

		NW_003726551_1, Position,0,1000,2000,3000,4000,5000,6000,7000,8000,9000,10000,11000,12000,13000,14000,15000,16000,17000,18000,19000,20000,21000,22000,23000,24000,25000,26000,27000,28000,29000,30000,31000,32000,33000,34000,35000,36000,37000,38000
NW_003726551_1,Count,0,0,0,0,0,0,0,0,0,1,0,0,0,0,0,0,0,0,0,0,0,0,0,0,0,0,0,0,0,1,0,0,0,0,0,0,0,0,0

	
```

```
		  

		NW_003726552_1, Position,0,1000,2000,3000,4000,5000,6000,7000,8000,9000,10000,11000,12000,13000,14000,15000,16000,17000,18000,19000,20000,21000,22000,23000,24000,25000,26000,27000,28000,29000,30000,31000,32000,33000,34000,35000,36000,37000,38000
NW_003726552_1,Count,0,0,0,0,0,0,0,0,0,0,0,0,0,0,0,0,0,0,0,0,0,0,0,0,0,0,0,0,0,0,0,0,0,0,0,0,8,1,0

	
```

```
		  

		NW_003726553_1, Position,0,1000,2000,3000,4000,5000,6000,7000,8000,9000,10000,11000,12000,13000,14000,15000,16000,17000,18000,19000,20000,21000,22000,23000,24000,25000,26000,27000,28000,29000,30000,31000,32000,33000,34000,35000,36000,37000
NW_003726553_1,Count,0,0,0,0,0,0,0,0,0,0,0,1,0,0,0,0,0,0,0,0,0,0,0,0,0,0,0,0,0,0,0,0,0,0,0,0,0,0

	
```

```
		  

		NW_003726555_1, Position,0,100,200,300,400,500,600,700,800,900,1000,1100,1200,1300,1400,1500,1600,1700,1800,1900,2000,2100,2200,2300,2400,2500,2600,2700,2800,2900,3000,3100,3200,3300,3400,3500,3600,3700,3800,3900,4000,4100,4200,4300,4400,4500,4600,4700,4800,4900,5000,5100,5200,5300,5400,5500,5600,5700,5800,5900,6000,6100,6200,6300,6400,6500,6600,6700,6800,6900,7000,7100,7200,7300,7400,7500,7600,7700,7800,7900,8000,8100,8200,8300,8400,8500,8600,8700,8800,8900,9000,9100,9200,9300,9400,9500,9600,9700,9800,9900,10000,10100,10200,10300,10400,10500,10600,10700,10800,10900,11000,11100,11200,11300,11400,11500,11600,11700,11800,11900,12000,12100,12200,12300,12400,12500,12600,12700,12800,12900,13000,13100,13200,13300,13400,13500,13600,13700,13800,13900,14000,14100,14200,14300,14400,14500,14600,14700,14800,14900,15000,15100,15200,15300,15400,15500,15600,15700,15800,15900,16000,16100,16200,16300,16400,16500,16600,16700,16800,16900,17000,17100,17200,17300,17400,17500,17600,17700,17800,17900,18000,18100,18200,18300,18400,18500,18600,18700,18800,18900,19000,19100,19200,19300,19400,19500,19600,19700,19800,19900,20000,20100,20200,20300,20400,20500,20600,20700,20800,20900,21000,21100,21200,21300,21400,21500,21600,21700,21800,21900,22000,22100,22200,22300,22400,22500,22600,22700,22800,22900,23000,23100,23200,23300,23400,23500,23600,23700,23800,23900,24000,24100,24200,24300,24400,24500,24600,24700,24800,24900,25000,25100,25200,25300,25400,25500,25600,25700,25800,25900,26000,26100,26200,26300,26400,26500,26600,26700,26800,26900,27000,27100,27200,27300,27400,27500,27600,27700,27800,27900
NW_003726555_1,Count,0,0,0,0,0,0,0,0,0,0,0,2,1,0,1,0,0,1,0,0,1,1,2,1,0,1,0,0,0,0,0,1,2,0,1,2,0,0,0,0,1,0,1,0,0,1,0,1,0,2,0,1,0,0,0,0,0,0,0,0,0,0,0,0,0,0,0,0,0,0,0,0,0,0,0,0,0,0,0,0,0,0,0,0,0,0,0,0,0,0,0,0,0,0,0,0,0,0,0,0,0,0,0,0,0,0,0,0,2,1,0,1,0,2,0,3,1,0,0,0,0,1,1,2,3,1,0,0,0,0,1,1,1,1,0,0,0,0,0,1,0,0,0,0,1,1,0,2,1,0,0,0,0,0,2,0,0,0,0,2,0,0,0,0,0,0,0,0,0,0,0,0,0,0,0,0,0,0,0,0,0,0,0,0,0,0,0,0,0,0,0,0,0,0,0,0,0,0,0,0,0,0,0,0,0,0,0,0,0,0,0,1,0,0,0,0,0,1,1,2,3,0,1,1,0,0,0,0,0,0,0,0,0,0,0,0,0,0,0,0,0,0,0,0,0,0,0,0,0,0,0,0,0,0,0,0,0,0,0,0,0,0,0,0,0,0,0,0,0,0,0,0,0,0,0,0,0,0,0,0

	
```

```
		  

		NW_003726556_1, Position,0,1000,2000,3000,4000,5000,6000,7000,8000,9000,10000,11000,12000,13000,14000,15000,16000,17000,18000,19000,20000,21000,22000,23000,24000,25000,26000,27000,28000,29000,30000,31000,32000,33000,34000,35000,36000,37000,38000
NW_003726556_1,Count,0,0,0,0,0,0,0,0,0,0,0,0,0,0,0,0,0,0,0,0,0,0,1,0,0,0,0,0,0,0,0,0,0,0,0,0,0,0,0

	
```

```
		  

		NW_003726562_1, Position,0,100,200,300,400,500,600,700,800,900,1000,1100,1200,1300,1400,1500,1600,1700,1800,1900,2000,2100,2200,2300,2400,2500,2600,2700,2800,2900,3000,3100,3200,3300,3400,3500,3600,3700,3800,3900,4000,4100,4200,4300,4400,4500,4600,4700,4800,4900,5000,5100,5200,5300,5400,5500,5600,5700,5800,5900,6000,6100,6200,6300,6400,6500,6600,6700,6800,6900,7000,7100,7200,7300,7400,7500,7600,7700,7800,7900,8000,8100,8200,8300,8400,8500,8600,8700,8800,8900,9000,9100,9200,9300,9400,9500,9600,9700,9800,9900,10000,10100,10200,10300,10400,10500,10600,10700,10800,10900,11000,11100,11200,11300,11400,11500,11600,11700,11800,11900,12000,12100,12200,12300,12400,12500,12600,12700,12800,12900,13000,13100,13200,13300,13400,13500,13600,13700,13800,13900,14000,14100,14200,14300,14400,14500,14600,14700,14800,14900,15000,15100,15200,15300,15400,15500,15600,15700,15800,15900,16000,16100,16200,16300,16400,16500,16600,16700,16800,16900,17000,17100,17200,17300,17400,17500,17600,17700,17800,17900,18000,18100,18200,18300,18400,18500,18600,18700,18800,18900,19000,19100,19200,19300,19400,19500,19600,19700,19800,19900,20000,20100,20200,20300,20400,20500,20600,20700,20800,20900,21000,21100,21200,21300,21400,21500,21600,21700,21800,21900,22000,22100,22200,22300,22400,22500,22600,22700,22800,22900,23000,23100,23200,23300,23400,23500,23600,23700,23800,23900,24000,24100,24200,24300,24400,24500,24600,24700,24800,24900,25000,25100,25200,25300,25400,25500,25600,25700,25800,25900,26000,26100,26200,26300,26400,26500,26600,26700,26800,26900,27000,27100,27200,27300,27400,27500,27600,27700,27800
NW_003726562_1,Count,0,0,0,0,0,0,0,0,0,0,0,0,0,0,0,0,0,0,0,0,0,0,0,0,0,0,0,0,0,0,0,0,0,0,0,0,0,0,0,0,0,0,0,0,0,0,0,0,0,0,0,0,0,0,0,0,0,0,0,0,0,0,0,0,0,0,0,0,0,0,0,0,0,0,0,0,0,0,0,0,0,0,0,0,0,0,0,0,0,0,0,0,0,0,0,0,0,0,0,0,0,0,0,0,0,0,3,1,0,0,0,0,0,0,0,0,0,0,0,0,0,0,0,0,0,0,0,0,0,0,0,0,0,0,0,0,0,0,0,0,0,0,0,0,0,0,0,0,0,0,0,0,0,0,0,0,0,4,0,1,1,0,0,0,0,0,0,0,0,0,0,3,2,1,4,0,0,0,0,0,1,2,2,1,1,0,0,0,0,0,0,0,0,0,0,0,0,0,0,0,0,0,0,0,0,0,1,1,2,3,0,0,0,0,0,0,0,0,0,0,0,0,0,0,0,0,0,0,0,0,1,1,0,0,0,0,0,0,0,0,0,0,0,0,0,0,0,0,0,0,0,1,0,0,1,0,0,0,0,0,0,0,1,0,3,1,0,2,1,2,2,0,0,0,1,1,1,0,0

	
```

```
		  

		NW_003726563_1, Position,0,1000,2000,3000,4000,5000,6000,7000,8000,9000,10000,11000,12000,13000,14000,15000,16000,17000,18000,19000,20000,21000,22000,23000,24000,25000,26000,27000,28000,29000,30000,31000,32000,33000,34000,35000,36000,37000,38000,39000,40000,41000
NW_003726563_1,Count,0,0,0,0,0,0,0,0,0,0,1,1,0,1,1,0,4,0,0,0,1,4,1,1,0,0,0,0,0,0,0,0,0,0,2,2,1,3,0,0,0,0

	
```

```
		  

		NW_003726567_1, Position,0,100,200,300,400,500,600,700,800,900,1000,1100,1200,1300,1400,1500,1600,1700,1800,1900,2000,2100,2200,2300,2400,2500,2600,2700,2800,2900,3000,3100,3200,3300,3400,3500,3600,3700,3800,3900,4000,4100,4200,4300,4400,4500,4600,4700,4800,4900,5000,5100,5200,5300,5400,5500,5600,5700,5800,5900,6000,6100,6200,6300,6400,6500,6600,6700,6800,6900,7000,7100,7200,7300,7400,7500,7600,7700,7800,7900,8000,8100,8200,8300,8400,8500,8600,8700,8800,8900,9000,9100,9200,9300,9400,9500,9600,9700,9800,9900,10000,10100,10200,10300,10400,10500,10600,10700,10800,10900,11000,11100,11200,11300,11400,11500,11600,11700,11800,11900,12000,12100,12200,12300,12400,12500,12600,12700,12800,12900,13000,13100,13200,13300,13400,13500,13600,13700,13800,13900,14000,14100,14200,14300,14400,14500,14600,14700,14800,14900,15000,15100,15200,15300,15400,15500,15600,15700,15800,15900,16000,16100,16200,16300,16400,16500,16600,16700,16800,16900,17000,17100,17200,17300,17400,17500,17600,17700,17800,17900,18000,18100,18200,18300,18400,18500,18600,18700,18800,18900,19000,19100,19200,19300,19400,19500,19600,19700,19800,19900,20000,20100,20200,20300,20400,20500,20600,20700,20800,20900,21000,21100,21200,21300,21400,21500,21600,21700,21800,21900,22000,22100,22200,22300,22400,22500,22600,22700,22800,22900,23000,23100,23200,23300,23400,23500,23600,23700,23800,23900,24000,24100,24200,24300,24400,24500,24600,24700,24800,24900,25000,25100,25200,25300,25400,25500,25600,25700,25800,25900,26000,26100,26200,26300,26400,26500,26600,26700,26800,26900,27000,27100,27200,27300,27400
NW_003726567_1,Count,1,1,2,0,1,2,1,1,0,1,0,0,1,1,0,0,0,3,1,2,1,1,0,1,0,3,1,1,1,0,2,2,1,1,0,2,2,0,0,0,0,0,2,0,0,0,1,0,3,1,1,0,0,2,1,1,0,1,1,0,0,1,0,1,1,0,1,0,0,1,1,2,1,1,1,1,2,1,1,1,0,0,1,1,1,1,1,0,0,0,1,1,1,1,1,2,0,0,1,1,0,3,0,1,2,2,0,0,0,0,0,0,0,0,0,0,1,1,0,1,0,0,0,1,0,2,1,0,0,0,1,0,0,0,1,0,0,0,1,0,1,0,2,2,1,0,0,0,0,0,0,0,0,0,0,2,1,2,0,0,2,0,0,0,1,0,0,1,1,0,0,3,0,0,0,0,1,0,1,1,0,0,0,0,1,0,2,0,1,2,1,0,2,1,0,4,1,1,0,2,0,2,1,2,1,0,0,1,0,3,0,2,0,1,0,0,2,1,2,3,0,0,0,0,0,0,0,1,0,0,0,0,0,0,0,0,0,0,0,0,0,0,0,0,0,0,0,0,0,0,1,1,0,0,0,0,0,0,1,0,0,0,0,0,0,2,0,0,2,0,0,1,1,3,0

	
```

```
		  

		NW_003726568_1, Position,0,100,200,300,400,500,600,700,800,900,1000,1100,1200,1300,1400,1500,1600,1700,1800,1900,2000,2100,2200,2300,2400,2500,2600,2700,2800,2900,3000,3100,3200,3300,3400,3500,3600,3700,3800,3900,4000,4100,4200,4300,4400,4500,4600,4700,4800,4900,5000,5100,5200,5300,5400,5500,5600,5700,5800,5900,6000,6100,6200,6300,6400,6500,6600,6700,6800,6900,7000,7100,7200,7300,7400,7500,7600,7700,7800,7900,8000,8100,8200,8300,8400,8500,8600,8700,8800,8900,9000,9100,9200,9300,9400,9500,9600,9700,9800,9900,10000,10100,10200,10300,10400,10500,10600,10700,10800,10900,11000,11100,11200,11300,11400,11500,11600,11700,11800,11900,12000,12100,12200,12300,12400,12500,12600,12700,12800,12900,13000,13100,13200,13300,13400,13500,13600,13700,13800,13900,14000,14100,14200,14300,14400,14500,14600,14700,14800,14900,15000,15100,15200,15300,15400,15500,15600,15700,15800,15900,16000,16100,16200,16300,16400,16500,16600,16700,16800,16900,17000,17100,17200,17300,17400,17500,17600,17700,17800,17900,18000,18100,18200,18300,18400,18500,18600,18700,18800,18900,19000,19100,19200,19300,19400,19500,19600,19700,19800,19900,20000,20100,20200,20300,20400,20500,20600,20700,20800,20900,21000,21100,21200,21300,21400,21500,21600,21700,21800,21900,22000,22100,22200,22300,22400,22500,22600,22700,22800,22900,23000,23100,23200,23300,23400,23500,23600,23700,23800,23900,24000,24100,24200,24300,24400,24500,24600,24700,24800,24900,25000,25100,25200,25300,25400,25500,25600,25700,25800,25900,26000,26100,26200,26300,26400,26500,26600,26700,26800,26900,27000,27100,27200,27300,27400
NW_003726568_1,Count,0,0,0,0,0,0,0,0,0,0,0,0,0,0,0,0,0,0,0,0,0,0,0,0,0,0,0,0,0,0,0,0,0,0,0,0,0,0,0,0,0,0,0,0,0,0,0,0,0,0,0,0,0,0,0,0,0,0,0,0,0,0,0,0,0,0,0,0,0,0,0,0,0,0,0,0,0,0,0,0,0,0,0,0,0,0,0,0,0,0,0,0,0,0,0,0,0,0,0,0,0,0,0,0,0,0,0,0,0,0,0,0,0,0,0,0,0,0,0,0,0,0,0,0,0,0,0,0,0,0,0,0,0,0,0,0,0,0,0,0,0,0,0,0,0,0,0,0,0,0,0,0,0,0,0,0,0,0,0,0,0,0,0,0,0,0,0,0,0,0,0,0,0,0,0,0,0,0,0,0,0,0,0,0,0,0,0,0,0,0,0,0,0,0,0,0,0,0,0,0,0,0,0,0,0,0,0,0,0,0,0,0,0,0,0,0,0,0,0,0,0,0,0,0,0,0,0,0,0,0,0,0,0,0,0,0,0,0,0,0,0,0,0,0,0,0,0,0,0,0,0,0,0,0,0,0,0,0,0,0,0,0,0,0,0,0,2,3,0,0,3,0,2,0,0

	
```

```
		  

		NW_003726569_1, Position,0,100,200,300,400,500,600,700,800,900,1000,1100,1200,1300,1400,1500,1600,1700,1800,1900,2000,2100,2200,2300,2400,2500,2600,2700,2800,2900,3000,3100,3200,3300,3400,3500,3600,3700,3800,3900,4000,4100,4200,4300,4400,4500,4600,4700,4800,4900,5000,5100,5200,5300,5400,5500,5600,5700,5800,5900,6000,6100,6200,6300,6400,6500,6600,6700,6800,6900,7000,7100,7200,7300,7400,7500,7600,7700,7800,7900,8000,8100,8200,8300,8400,8500,8600,8700,8800,8900,9000,9100,9200,9300,9400,9500,9600,9700,9800,9900,10000,10100,10200,10300,10400,10500,10600,10700,10800,10900,11000,11100,11200,11300,11400,11500,11600,11700,11800,11900,12000,12100,12200,12300,12400,12500,12600,12700,12800,12900,13000,13100,13200,13300,13400,13500,13600,13700,13800,13900,14000,14100,14200,14300,14400,14500,14600,14700,14800,14900,15000,15100,15200,15300,15400,15500,15600,15700,15800,15900,16000,16100,16200,16300,16400,16500,16600,16700,16800,16900,17000,17100,17200,17300,17400,17500,17600,17700,17800,17900,18000,18100,18200,18300,18400,18500,18600,18700,18800,18900,19000,19100,19200,19300,19400,19500,19600,19700,19800,19900,20000,20100,20200,20300,20400,20500,20600,20700,20800,20900,21000,21100,21200,21300,21400,21500,21600,21700,21800,21900,22000,22100,22200,22300,22400,22500,22600,22700,22800,22900,23000,23100,23200,23300,23400,23500,23600,23700,23800,23900,24000,24100,24200,24300,24400,24500,24600,24700,24800,24900,25000,25100,25200,25300,25400,25500,25600,25700,25800,25900,26000,26100,26200,26300,26400,26500,26600,26700,26800,26900,27000,27100,27200,27300,27400,27500,27600,27700,27800,27900,28000
NW_003726569_1,Count,0,0,0,0,0,0,0,0,0,0,0,0,0,0,0,0,0,0,0,0,1,0,0,0,0,0,0,0,0,0,0,0,0,0,0,0,0,0,0,0,1,0,3,1,1,0,0,0,0,0,0,0,0,0,0,0,0,0,0,0,0,0,0,0,0,0,0,0,0,0,0,0,0,0,0,0,0,0,0,0,0,0,0,0,0,0,0,0,0,0,0,0,0,0,0,0,0,0,0,0,0,0,0,0,0,0,0,0,0,0,0,1,0,0,0,0,0,0,0,0,0,0,0,0,0,0,0,0,0,0,0,0,0,0,0,0,0,0,0,0,0,0,0,0,0,0,0,0,0,0,0,0,0,0,1,1,1,0,0,0,0,0,0,0,0,0,0,0,0,0,0,0,0,0,0,0,0,0,0,0,0,0,0,0,1,0,0,0,0,0,0,0,0,0,0,0,0,0,0,0,0,0,0,0,0,0,0,0,1,0,0,0,0,0,0,0,0,0,0,0,0,0,0,0,0,0,0,0,0,0,0,0,0,0,0,0,0,0,0,0,0,1,3,0,0,0,0,0,0,0,0,0,0,0,0,0,2,1,1,1,1,0,0,0,0,0,0,0,0,0,0,0,0,0,0,0,0,0,0,0,0

	
```

```
		  

		NW_003726570_1, Position,0,100,200,300,400,500,600,700,800,900,1000,1100,1200,1300,1400,1500,1600,1700,1800,1900,2000,2100,2200,2300,2400,2500,2600,2700,2800,2900,3000,3100,3200,3300,3400,3500,3600,3700,3800,3900,4000,4100,4200,4300,4400,4500,4600,4700,4800,4900,5000,5100,5200,5300,5400,5500,5600,5700,5800,5900,6000,6100,6200,6300,6400,6500,6600,6700,6800,6900,7000,7100,7200,7300,7400,7500,7600,7700,7800,7900,8000,8100,8200,8300,8400,8500,8600,8700,8800,8900,9000,9100,9200,9300,9400,9500,9600,9700,9800,9900,10000,10100,10200,10300,10400,10500,10600,10700,10800,10900,11000,11100,11200,11300,11400,11500,11600,11700,11800,11900,12000,12100,12200,12300,12400,12500,12600,12700,12800,12900,13000,13100,13200,13300,13400,13500,13600,13700,13800,13900,14000,14100,14200,14300,14400,14500,14600,14700,14800,14900,15000,15100,15200,15300,15400,15500,15600,15700,15800,15900,16000,16100,16200,16300,16400,16500,16600,16700,16800,16900,17000,17100,17200,17300,17400,17500,17600,17700,17800,17900,18000,18100,18200,18300,18400,18500,18600,18700,18800,18900,19000,19100,19200,19300,19400,19500,19600,19700,19800,19900,20000,20100,20200,20300,20400,20500,20600,20700,20800,20900,21000,21100,21200,21300,21400,21500,21600,21700,21800,21900,22000,22100,22200,22300,22400,22500,22600,22700,22800,22900,23000,23100,23200,23300,23400,23500,23600,23700,23800,23900,24000,24100,24200,24300,24400,24500,24600,24700,24800,24900,25000,25100,25200,25300,25400,25500,25600,25700,25800,25900,26000,26100,26200,26300,26400,26500,26600,26700,26800,26900,27000,27100,27200,27300
NW_003726570_1,Count,0,0,0,0,0,0,0,0,0,0,0,0,0,0,0,0,0,0,0,0,0,0,0,0,0,0,0,0,0,0,0,0,0,0,0,0,0,0,0,0,0,0,0,0,0,0,0,0,0,0,0,0,0,0,0,0,0,0,0,0,0,0,0,0,0,0,0,0,0,0,0,0,0,0,0,0,0,0,0,0,0,0,0,0,0,0,0,0,0,0,0,0,0,0,0,0,0,0,0,0,0,0,0,0,0,0,0,0,0,0,0,0,1,0,0,0,0,0,0,0,0,0,0,0,0,0,0,0,0,0,0,0,0,0,0,0,0,0,0,0,0,0,0,0,0,0,0,0,0,0,0,0,0,0,0,0,0,0,0,0,0,0,0,0,0,0,0,0,0,0,0,0,0,0,0,0,0,0,0,0,0,0,0,0,0,0,0,0,0,0,0,0,0,0,0,0,0,0,0,0,0,0,0,0,0,0,0,0,0,0,0,0,0,0,0,0,0,0,0,0,0,0,0,0,0,0,0,0,0,0,0,0,0,0,0,0,0,0,0,0,0,0,0,0,2,0,0,0,0,0,0,0,0,1,0,0,0,1,2,1,0,0,2,0,0,0,0,0,0,0,0,0,0,0

	
```

```
		  

		NW_003726575_1, Position,0,1000,2000,3000,4000,5000,6000,7000,8000,9000,10000,11000,12000,13000,14000,15000,16000,17000,18000,19000,20000,21000,22000,23000,24000,25000,26000,27000,28000,29000,30000,31000,32000
NW_003726575_1,Count,0,0,0,0,0,0,0,0,0,0,0,0,0,0,0,0,2,0,0,0,0,0,0,0,0,0,0,0,0,0,0,0,0

	
```

```
		  

		NW_003726586_1, Position,0,100,200,300,400,500,600,700,800,900,1000,1100,1200,1300,1400,1500,1600,1700,1800,1900,2000,2100,2200,2300,2400,2500,2600,2700,2800,2900,3000,3100,3200,3300,3400,3500,3600,3700,3800,3900,4000,4100,4200,4300,4400,4500,4600,4700,4800,4900,5000,5100,5200,5300,5400,5500,5600,5700,5800,5900,6000,6100,6200,6300,6400,6500,6600,6700,6800,6900,7000,7100,7200,7300,7400,7500,7600,7700,7800,7900,8000,8100,8200,8300,8400,8500,8600,8700,8800,8900,9000,9100,9200,9300,9400,9500,9600,9700,9800,9900,10000,10100,10200,10300,10400,10500,10600,10700,10800,10900,11000,11100,11200,11300,11400,11500,11600,11700,11800,11900,12000,12100,12200,12300,12400,12500,12600,12700,12800,12900,13000,13100,13200,13300,13400,13500,13600,13700,13800,13900,14000,14100,14200,14300,14400,14500,14600,14700,14800,14900,15000,15100,15200,15300,15400,15500,15600,15700,15800,15900,16000,16100,16200,16300,16400,16500,16600,16700,16800,16900,17000,17100,17200,17300,17400,17500,17600,17700,17800,17900,18000,18100,18200,18300,18400,18500,18600,18700,18800,18900,19000,19100,19200,19300,19400,19500,19600,19700,19800,19900,20000,20100,20200,20300,20400,20500,20600,20700,20800,20900,21000,21100,21200,21300,21400,21500,21600,21700,21800,21900,22000,22100,22200,22300,22400,22500,22600,22700,22800,22900,23000,23100,23200,23300,23400,23500,23600,23700,23800,23900,24000,24100,24200,24300,24400,24500,24600,24700,24800,24900,25000,25100,25200,25300,25400,25500,25600,25700,25800,25900,26000,26100,26200,26300,26400,26500,26600,26700
NW_003726586_1,Count,0,0,0,0,0,0,0,0,0,0,0,0,0,0,0,0,0,0,0,0,0,0,0,0,0,0,0,0,0,0,0,0,0,0,0,0,0,0,0,0,0,0,0,0,0,0,0,0,0,0,0,0,0,0,0,0,0,0,0,0,0,0,0,0,0,0,0,0,0,0,0,0,0,0,0,0,0,0,0,0,0,0,0,0,0,0,0,0,0,0,0,0,0,0,0,0,0,0,0,0,0,0,0,0,0,0,0,0,0,0,0,0,0,0,0,0,0,0,0,0,0,0,0,0,0,0,0,0,0,0,0,0,0,0,0,0,0,0,0,0,0,0,0,0,0,0,0,0,0,0,0,0,0,0,0,0,0,0,0,0,0,0,0,0,0,0,0,0,0,0,0,0,0,0,0,0,0,0,0,0,0,0,0,0,0,0,0,0,0,0,0,0,0,0,0,0,0,0,0,0,0,0,0,0,0,0,0,0,0,0,0,0,0,0,0,0,0,0,0,0,0,0,0,0,0,0,0,0,0,0,0,0,0,0,0,0,0,0,0,0,0,0,0,0,1,0,2,0,0,0,0,0,0,0,0,0,0,0,0,0,0,0,0,0,0,0,0,0

	
```

```
		  

		NW_003726587_1, Position,0,100,200,300,400,500,600,700,800,900,1000,1100,1200,1300,1400,1500,1600,1700,1800,1900,2000,2100,2200,2300,2400,2500,2600,2700,2800,2900,3000,3100,3200,3300,3400,3500,3600,3700,3800,3900,4000,4100,4200,4300,4400,4500,4600,4700,4800,4900,5000,5100,5200,5300,5400,5500,5600,5700,5800,5900,6000,6100,6200,6300,6400,6500,6600,6700,6800,6900,7000,7100,7200,7300,7400,7500,7600,7700,7800,7900,8000,8100,8200,8300,8400,8500,8600,8700,8800,8900,9000,9100,9200,9300,9400,9500,9600,9700,9800,9900,10000,10100,10200,10300,10400,10500,10600,10700,10800,10900,11000,11100,11200,11300,11400,11500,11600,11700,11800,11900,12000,12100,12200,12300,12400,12500,12600,12700,12800,12900,13000,13100,13200,13300,13400,13500,13600,13700,13800,13900,14000,14100,14200,14300,14400,14500,14600,14700,14800,14900,15000,15100,15200,15300,15400,15500,15600,15700,15800,15900,16000,16100,16200,16300,16400,16500,16600,16700,16800,16900,17000,17100,17200,17300,17400,17500,17600,17700,17800,17900,18000,18100,18200,18300,18400,18500,18600,18700,18800,18900,19000,19100,19200,19300,19400,19500,19600,19700,19800,19900,20000,20100,20200,20300,20400,20500,20600,20700,20800,20900,21000,21100,21200,21300,21400,21500,21600,21700,21800,21900,22000,22100,22200,22300,22400,22500,22600,22700,22800,22900,23000,23100,23200,23300,23400,23500,23600,23700,23800,23900,24000,24100,24200,24300,24400,24500,24600,24700,24800,24900,25000,25100,25200,25300,25400,25500,25600,25700,25800,25900,26000,26100,26200,26300,26400,26500,26600,26700,26800,26900,27000,27100,27200,27300,27400,27500,27600,27700,27800
NW_003726587_1,Count,0,0,0,0,0,0,0,0,0,0,0,0,0,0,0,0,0,0,0,0,0,0,0,0,0,0,0,0,0,0,0,0,0,0,0,0,0,0,0,0,0,0,0,0,0,0,0,0,0,0,0,0,0,0,0,0,0,0,0,0,0,0,0,0,0,0,0,0,0,0,0,0,0,0,0,0,0,0,0,0,0,0,0,0,0,0,0,0,0,0,0,0,0,0,0,0,0,0,0,0,0,0,0,0,0,0,0,0,0,0,0,0,0,0,0,0,0,0,0,0,0,0,0,0,0,0,0,0,0,0,0,0,0,0,0,0,0,0,0,0,0,0,0,0,0,0,0,0,0,0,0,0,0,0,0,0,0,0,0,0,0,0,0,0,0,0,0,0,0,0,0,0,0,0,0,0,0,0,0,0,0,0,0,0,0,0,0,0,0,0,0,0,0,0,0,0,0,0,0,0,0,0,0,0,0,0,0,0,0,0,0,0,0,0,1,0,0,0,0,0,0,0,0,0,0,0,0,0,0,0,0,0,0,0,0,0,0,0,0,0,0,0,0,0,0,0,0,0,0,0,0,0,0,0,0,0,0,0,0,0,0,0,0,0,0,0,0,0,0,0,0,0,0,0,0,0,0,0,0

	
```

```
		  

		NW_003726588_1, Position,0,100,200,300,400,500,600,700,800,900,1000,1100,1200,1300,1400,1500,1600,1700,1800,1900,2000,2100,2200,2300,2400,2500,2600,2700,2800,2900,3000,3100,3200,3300,3400,3500,3600,3700,3800,3900,4000,4100,4200,4300,4400,4500,4600,4700,4800,4900,5000,5100,5200,5300,5400,5500,5600,5700,5800,5900,6000,6100,6200,6300,6400,6500,6600,6700,6800,6900,7000,7100,7200,7300,7400,7500,7600,7700,7800,7900,8000,8100,8200,8300,8400,8500,8600,8700,8800,8900,9000,9100,9200,9300,9400,9500,9600,9700,9800,9900,10000,10100,10200,10300,10400,10500,10600,10700,10800,10900,11000,11100,11200,11300,11400,11500,11600,11700,11800,11900,12000,12100,12200,12300,12400,12500,12600,12700,12800,12900,13000,13100,13200,13300,13400,13500,13600,13700,13800,13900,14000,14100,14200,14300,14400,14500,14600,14700,14800,14900,15000,15100,15200,15300,15400,15500,15600,15700,15800,15900,16000,16100,16200,16300,16400,16500,16600,16700,16800,16900,17000,17100,17200,17300,17400,17500,17600,17700,17800,17900,18000,18100,18200,18300,18400,18500,18600,18700,18800,18900,19000,19100,19200,19300,19400,19500,19600,19700,19800,19900,20000,20100,20200,20300,20400,20500,20600,20700,20800,20900,21000,21100,21200,21300,21400,21500,21600,21700,21800,21900,22000,22100,22200,22300,22400,22500,22600,22700,22800,22900,23000,23100,23200,23300,23400,23500,23600,23700,23800,23900,24000,24100,24200,24300,24400,24500,24600,24700,24800,24900,25000,25100,25200,25300,25400,25500,25600,25700,25800,25900,26000,26100,26200,26300,26400,26500,26600,26700,26800,26900,27000,27100,27200,27300,27400,27500,27600,27700,27800,27900,28000,28100,28200,28300,28400,28500,28600,28700,28800,28900,29000,29100,29200,29300,29400,29500
NW_003726588_1,Count,0,0,0,0,1,0,0,0,0,1,1,2,0,0,0,0,0,0,0,0,0,0,0,0,0,0,0,0,0,0,0,0,0,0,0,0,0,0,0,0,0,0,0,0,0,0,0,0,0,0,0,0,0,0,0,0,0,0,0,0,0,0,0,0,0,1,1,0,0,0,0,0,0,0,0,0,0,0,0,0,0,0,0,0,0,0,0,0,0,0,0,0,0,0,0,0,0,0,0,0,0,0,0,0,0,0,0,0,0,0,0,0,0,0,0,0,0,0,0,0,0,0,0,0,0,0,0,0,0,0,0,0,0,0,0,0,0,0,0,0,0,0,0,0,0,0,0,0,0,0,0,0,0,0,0,0,0,0,0,0,0,0,0,0,0,0,0,0,0,0,0,0,0,0,0,0,0,1,1,1,0,0,0,0,0,0,0,0,0,0,0,0,0,0,0,0,0,0,0,0,0,0,0,0,0,0,0,0,0,0,0,0,0,0,0,0,0,0,0,0,0,0,0,0,0,0,0,0,0,0,0,0,0,0,0,0,0,0,0,0,0,0,0,0,0,0,0,0,0,0,0,0,0,0,0,0,0,0,0,0,0,0,0,0,0,0,0,0,0,0,0,0,0,0,0,0,0,0,0,0,0,0,0,0,0,0,0,0,0,0,0,0,0,0,0,0

	
```

```
		  

		NW_003726589_1, Position,0,100,200,300,400,500,600,700,800,900,1000,1100,1200,1300,1400,1500,1600,1700,1800,1900,2000,2100,2200,2300,2400,2500,2600,2700,2800,2900,3000,3100,3200,3300,3400,3500,3600,3700,3800,3900,4000,4100,4200,4300,4400,4500,4600,4700,4800,4900,5000,5100,5200,5300,5400,5500,5600,5700,5800,5900,6000,6100,6200,6300,6400,6500,6600,6700,6800,6900,7000,7100,7200,7300,7400,7500,7600,7700,7800,7900,8000,8100,8200,8300,8400,8500,8600,8700,8800,8900,9000,9100,9200,9300,9400,9500,9600,9700,9800,9900,10000,10100,10200,10300,10400,10500,10600,10700,10800,10900,11000,11100,11200,11300,11400,11500,11600,11700,11800,11900,12000,12100,12200,12300,12400,12500,12600,12700,12800,12900,13000,13100,13200,13300,13400,13500,13600,13700,13800,13900,14000,14100,14200,14300,14400,14500,14600,14700,14800,14900,15000,15100,15200,15300,15400,15500,15600,15700,15800,15900,16000,16100,16200,16300,16400,16500,16600,16700,16800,16900,17000,17100,17200,17300,17400,17500,17600,17700,17800,17900,18000,18100,18200,18300,18400,18500,18600,18700,18800,18900,19000,19100,19200,19300,19400,19500,19600,19700,19800,19900,20000,20100,20200,20300,20400,20500,20600,20700,20800,20900,21000,21100,21200,21300,21400,21500,21600,21700,21800,21900,22000,22100,22200,22300,22400,22500,22600,22700,22800,22900,23000,23100,23200,23300,23400,23500,23600,23700,23800,23900,24000,24100,24200,24300,24400,24500,24600,24700,24800,24900,25000,25100,25200,25300,25400,25500,25600,25700,25800,25900,26000,26100,26200,26300,26400,26500,26600,26700,26800,26900,27000,27100,27200,27300,27400,27500,27600,27700,27800,27900,28000,28100,28200,28300,28400,28500,28600,28700
NW_003726589_1,Count,0,0,1,0,1,1,2,1,2,1,0,2,1,1,1,2,0,3,2,1,0,3,2,1,0,3,1,0,0,1,0,2,1,0,1,0,1,2,1,1,2,1,0,0,2,0,2,0,0,0,0,0,0,1,0,0,2,0,0,1,2,0,1,0,0,0,1,1,0,1,0,1,1,0,0,0,0,0,0,3,1,0,0,1,0,0,1,1,0,1,0,1,1,2,1,0,0,0,0,0,0,1,0,3,0,1,2,0,0,2,0,0,1,2,2,1,1,0,0,2,0,0,0,0,0,0,0,1,3,1,0,0,1,1,2,2,0,0,3,0,0,1,0,0,1,2,3,2,1,0,1,0,0,2,0,0,1,0,0,0,1,0,0,1,0,1,2,0,0,0,0,0,1,1,1,0,0,0,0,1,0,0,0,0,1,2,0,2,1,2,1,1,0,1,0,0,1,1,1,0,0,1,2,1,2,0,0,1,2,1,0,1,2,0,0,0,4,0,1,0,1,1,1,3,1,2,1,3,0,0,1,1,1,2,1,2,0,0,4,0,1,1,0,2,0,0,1,2,0,2,0,0,0,0,0,0,0,0,0,0,0,0,0,0,3,1,5,1,0,0,2,0,0,3,1,0,2,0,1,2,2,1,1,0,0,0,0,1

	
```

```
		  

		NW_003726592_1, Position,0,100,200,300,400,500,600,700,800,900,1000,1100,1200,1300,1400,1500,1600,1700,1800,1900,2000,2100,2200,2300,2400,2500,2600,2700,2800,2900,3000,3100,3200,3300,3400,3500,3600,3700,3800,3900,4000,4100,4200,4300,4400,4500,4600,4700,4800,4900,5000,5100,5200,5300,5400,5500,5600,5700,5800,5900,6000,6100,6200,6300,6400,6500,6600,6700,6800,6900,7000,7100,7200,7300,7400,7500,7600,7700,7800,7900,8000,8100,8200,8300,8400,8500,8600,8700,8800,8900,9000,9100,9200,9300,9400,9500,9600,9700,9800,9900,10000,10100,10200,10300,10400,10500,10600,10700,10800,10900,11000,11100,11200,11300,11400,11500,11600,11700,11800,11900,12000,12100,12200,12300,12400,12500,12600,12700,12800,12900,13000,13100,13200,13300,13400,13500,13600,13700,13800,13900,14000,14100,14200,14300,14400,14500,14600,14700,14800,14900,15000,15100,15200,15300,15400,15500,15600,15700,15800,15900,16000,16100,16200,16300,16400,16500,16600,16700,16800,16900,17000,17100,17200,17300,17400,17500,17600,17700,17800,17900,18000,18100,18200,18300,18400,18500,18600,18700,18800,18900,19000,19100,19200,19300,19400,19500,19600,19700,19800,19900,20000,20100,20200,20300,20400,20500,20600,20700,20800,20900,21000,21100,21200,21300,21400,21500,21600,21700,21800,21900,22000,22100,22200,22300,22400,22500,22600,22700,22800,22900,23000,23100,23200,23300,23400,23500,23600,23700,23800,23900,24000,24100,24200,24300,24400,24500,24600,24700,24800,24900,25000,25100,25200,25300,25400,25500,25600,25700,25800,25900,26000,26100,26200,26300,26400,26500
NW_003726592_1,Count,0,0,0,0,0,0,0,0,0,0,0,0,0,0,0,0,0,0,0,0,0,0,0,0,0,0,0,0,0,0,0,0,0,0,0,0,0,0,1,2,0,0,0,0,0,0,0,0,1,1,0,4,0,0,0,0,2,1,3,1,2,0,1,3,0,0,0,0,0,0,0,0,0,0,0,0,0,0,0,0,0,0,0,0,0,0,0,0,0,0,0,0,0,0,0,0,0,0,0,0,0,0,0,0,0,0,1,0,0,0,1,1,2,1,1,1,0,1,0,0,0,0,0,1,1,0,0,2,0,1,0,0,0,0,0,1,1,2,0,0,1,1,0,1,0,0,0,2,1,2,1,0,2,1,0,0,1,1,1,3,2,0,1,0,0,1,0,1,0,0,0,0,1,1,0,4,2,0,0,0,0,2,0,0,2,0,1,1,1,1,1,2,0,0,1,0,1,0,0,0,0,2,0,1,1,1,0,1,0,0,0,1,1,0,0,0,0,2,0,0,0,0,0,0,0,0,0,0,0,0,0,0,0,0,0,0,0,0,0,0,0,0,0,0,0,0,0,0,0,0,0,0,0,0,0,0,0,0,0,0,0,0,0,0,0,0

	
```

```
		  

		NW_003726593_1, Position,0,1000,2000,3000,4000,5000,6000,7000,8000,9000,10000,11000,12000,13000,14000,15000,16000,17000,18000,19000,20000,21000,22000,23000,24000,25000,26000,27000,28000,29000,30000,31000,32000,33000,34000,35000,36000,37000,38000,39000,40000,41000,42000,43000,44000
NW_003726593_1,Count,0,0,0,0,0,0,0,0,1,11,0,0,0,0,0,0,0,0,0,0,0,0,0,0,0,0,0,0,0,0,0,0,0,0,0,0,0,0,0,0,0,0,0,0,0

	
```

```
		  

		NW_003726594_1, Position,0,1000,2000,3000,4000,5000,6000,7000,8000,9000,10000,11000,12000,13000,14000,15000,16000,17000,18000,19000,20000,21000,22000,23000,24000,25000,26000,27000,28000,29000,30000,31000,32000,33000
NW_003726594_1,Count,1,0,0,0,0,0,0,0,0,0,0,0,0,0,0,0,0,0,0,0,0,0,0,0,0,0,0,0,0,0,0,0,0,0

	
```

```
		  

		NW_003726597_1, Position,0,100,200,300,400,500,600,700,800,900,1000,1100,1200,1300,1400,1500,1600,1700,1800,1900,2000,2100,2200,2300,2400,2500,2600,2700,2800,2900,3000,3100,3200,3300,3400,3500,3600,3700,3800,3900,4000,4100,4200,4300,4400,4500,4600,4700,4800,4900,5000,5100,5200,5300,5400,5500,5600,5700,5800,5900,6000,6100,6200,6300,6400,6500,6600,6700,6800,6900,7000,7100,7200,7300,7400,7500,7600,7700,7800,7900,8000,8100,8200,8300,8400,8500,8600,8700,8800,8900,9000,9100,9200,9300,9400,9500,9600,9700,9800,9900,10000,10100,10200,10300,10400,10500,10600,10700,10800,10900,11000,11100,11200,11300,11400,11500,11600,11700,11800,11900,12000,12100,12200,12300,12400,12500,12600,12700,12800,12900,13000,13100,13200,13300,13400,13500,13600,13700,13800,13900,14000,14100,14200,14300,14400,14500,14600,14700,14800,14900,15000,15100,15200,15300,15400,15500,15600,15700,15800,15900,16000,16100,16200,16300,16400,16500,16600,16700,16800,16900,17000,17100,17200,17300,17400,17500,17600,17700,17800,17900,18000,18100,18200,18300,18400,18500,18600,18700,18800,18900,19000,19100,19200,19300,19400,19500,19600,19700,19800,19900,20000,20100,20200,20300,20400,20500,20600,20700,20800,20900,21000,21100,21200,21300,21400,21500,21600,21700,21800,21900,22000,22100,22200,22300,22400,22500,22600,22700,22800,22900,23000,23100,23200,23300,23400,23500,23600,23700,23800,23900,24000,24100,24200,24300,24400,24500,24600,24700,24800,24900,25000,25100,25200,25300,25400,25500,25600,25700,25800,25900,26000,26100,26200,26300
NW_003726597_1,Count,0,0,0,0,0,0,0,0,0,0,0,0,0,0,0,0,0,0,0,0,0,0,0,0,0,0,0,0,0,0,0,0,0,0,0,0,0,0,0,0,0,0,0,0,0,0,0,0,0,0,0,0,0,0,0,0,0,0,0,0,0,0,0,0,0,0,0,0,0,0,0,0,0,0,0,0,0,0,0,0,0,0,0,0,0,0,0,0,0,0,0,0,0,0,0,0,0,0,0,0,0,0,0,0,0,0,0,0,0,0,0,0,0,0,0,0,0,0,0,0,0,0,0,0,0,0,0,0,0,0,0,0,0,0,0,0,0,0,0,0,0,0,0,0,0,0,0,0,0,0,0,0,0,0,0,0,0,0,0,0,0,0,0,0,0,0,0,0,0,0,0,0,0,0,0,0,0,0,0,0,0,0,0,0,0,0,0,0,0,0,0,0,0,0,0,0,0,0,0,0,0,0,0,0,0,0,0,0,0,0,0,0,0,0,0,0,0,0,0,0,0,0,1,2,0,0,0,0,0,0,0,0,0,0,0,0,0,0,0,0,0,0,0,0,0,0,0,0,0,0,0,0,0,0,0,0,0,0,0,0,0,0,0,0

	
```

```
		  

		NW_003726599_1, Position,0,100,200,300,400,500,600,700,800,900,1000,1100,1200,1300,1400,1500,1600,1700,1800,1900,2000,2100,2200,2300,2400,2500,2600,2700,2800,2900,3000,3100,3200,3300,3400,3500,3600,3700,3800,3900,4000,4100,4200,4300,4400,4500,4600,4700,4800,4900,5000,5100,5200,5300,5400,5500,5600,5700,5800,5900,6000,6100,6200,6300,6400,6500,6600,6700,6800,6900,7000,7100,7200,7300,7400,7500,7600,7700,7800,7900,8000,8100,8200,8300,8400,8500,8600,8700,8800,8900,9000,9100,9200,9300,9400,9500,9600,9700,9800,9900,10000,10100,10200,10300,10400,10500,10600,10700,10800,10900,11000,11100,11200,11300,11400,11500,11600,11700,11800,11900,12000,12100,12200,12300,12400,12500,12600,12700,12800,12900,13000,13100,13200,13300,13400,13500,13600,13700,13800,13900,14000,14100,14200,14300,14400,14500,14600,14700,14800,14900,15000,15100,15200,15300,15400,15500,15600,15700,15800,15900,16000,16100,16200,16300,16400,16500,16600,16700,16800,16900,17000,17100,17200,17300,17400,17500,17600,17700,17800,17900,18000,18100,18200,18300,18400,18500,18600,18700,18800,18900,19000,19100,19200,19300,19400,19500,19600,19700,19800,19900,20000,20100,20200,20300,20400,20500,20600,20700,20800,20900,21000,21100,21200,21300,21400,21500,21600,21700,21800,21900,22000,22100,22200,22300,22400,22500,22600,22700,22800,22900,23000,23100,23200,23300,23400,23500,23600,23700,23800,23900,24000,24100,24200,24300,24400,24500,24600,24700,24800,24900,25000,25100,25200,25300,25400,25500,25600,25700,25800,25900,26000,26100,26200,26300,26400,26500,26600,26700,26800,26900,27000,27100,27200,27300
NW_003726599_1,Count,0,0,0,0,0,1,1,0,0,2,1,0,3,0,1,2,3,1,0,0,1,1,0,0,0,0,0,0,0,0,0,0,0,0,0,0,1,3,0,2,2,0,0,0,1,0,0,0,1,1,2,0,0,0,0,0,0,0,0,0,0,0,0,0,2,1,1,1,3,0,0,0,0,0,0,0,0,0,4,0,0,1,0,2,0,0,0,0,0,0,0,0,0,0,0,0,0,0,0,0,0,0,0,0,1,0,2,0,0,0,0,0,0,0,0,0,0,0,3,1,3,1,1,3,0,0,0,0,0,0,0,0,0,0,0,0,0,0,1,0,3,1,0,0,0,0,0,1,0,0,0,0,0,0,0,0,0,0,0,0,0,0,1,2,0,0,0,0,0,0,0,0,0,0,0,0,0,0,0,0,0,0,0,0,0,3,2,0,0,0,0,0,0,0,0,0,0,0,0,0,0,0,0,0,0,0,2,1,0,0,0,0,0,0,2,2,2,0,0,0,2,1,0,3,1,0,0,0,0,2,1,0,0,0,0,1,1,2,1,0,0,1,0,0,2,1,3,3,0,1,2,1,0,3,0,1,1,1,0,0,0,0,0,0,0,0,0,0,0,0,0,0,0,0

	
```

```
		  

		NW_003726600_1, Position,0,1000,2000,3000,4000,5000,6000,7000,8000,9000,10000,11000,12000,13000,14000,15000,16000,17000,18000,19000,20000,21000,22000,23000,24000,25000,26000,27000,28000,29000,30000,31000,32000,33000,34000,35000,36000
NW_003726600_1,Count,3,3,4,2,3,0,0,0,1,0,0,0,0,0,0,0,3,0,10,11,0,0,0,0,0,0,0,0,0,0,0,0,0,0,0,3,2

	
```

```
		  

		NW_003726602_1, Position,0,100,200,300,400,500,600,700,800,900,1000,1100,1200,1300,1400,1500,1600,1700,1800,1900,2000,2100,2200,2300,2400,2500,2600,2700,2800,2900,3000,3100,3200,3300,3400,3500,3600,3700,3800,3900,4000,4100,4200,4300,4400,4500,4600,4700,4800,4900,5000,5100,5200,5300,5400,5500,5600,5700,5800,5900,6000,6100,6200,6300,6400,6500,6600,6700,6800,6900,7000,7100,7200,7300,7400,7500,7600,7700,7800,7900,8000,8100,8200,8300,8400,8500,8600,8700,8800,8900,9000,9100,9200,9300,9400,9500,9600,9700,9800,9900,10000,10100,10200,10300,10400,10500,10600,10700,10800,10900,11000,11100,11200,11300,11400,11500,11600,11700,11800,11900,12000,12100,12200,12300,12400,12500,12600,12700,12800,12900,13000,13100,13200,13300,13400,13500,13600,13700,13800,13900,14000,14100,14200,14300,14400,14500,14600,14700,14800,14900,15000,15100,15200,15300,15400,15500,15600,15700,15800,15900,16000,16100,16200,16300,16400,16500,16600,16700,16800,16900,17000,17100,17200,17300,17400,17500,17600,17700,17800,17900,18000,18100,18200,18300,18400,18500,18600,18700,18800,18900,19000,19100,19200,19300,19400,19500,19600,19700,19800,19900,20000,20100,20200,20300,20400,20500,20600,20700,20800,20900,21000,21100,21200,21300,21400,21500,21600,21700,21800,21900,22000,22100,22200,22300,22400,22500,22600,22700,22800,22900,23000,23100,23200,23300,23400,23500,23600,23700,23800,23900,24000,24100,24200,24300,24400,24500,24600,24700,24800,24900,25000,25100,25200,25300,25400,25500,25600,25700,25800,25900,26000,26100
NW_003726602_1,Count,0,0,0,1,1,4,0,1,2,0,0,0,2,1,0,1,1,0,1,1,0,0,0,0,1,1,0,1,1,1,3,2,2,0,0,1,0,1,1,1,0,1,0,0,0,0,1,2,1,0,1,0,1,2,0,3,0,0,0,1,2,0,1,0,0,0,0,1,2,0,3,3,0,1,0,0,0,0,0,0,0,0,0,1,2,0,0,1,1,2,0,0,1,2,3,1,2,2,0,2,1,1,1,0,0,0,0,0,0,3,1,0,0,0,0,0,0,0,0,0,0,0,0,0,0,0,0,0,0,0,0,0,0,0,2,1,0,0,1,1,0,0,2,1,1,1,1,0,0,0,0,0,0,0,2,1,1,0,1,1,0,0,0,0,0,0,0,0,0,0,0,0,1,1,0,2,0,0,0,0,0,0,0,1,2,0,1,0,0,0,0,1,0,3,0,0,0,2,1,0,1,0,0,0,0,1,2,1,0,0,0,1,1,1,1,0,0,0,0,0,0,0,0,0,0,0,2,1,0,0,0,0,0,0,0,0,0,0,0,0,0,2,0,0,0,0,0,0,0,0,0,0,0,0,0,0,0,0,0,0,0,0

	
```

```
		  

		NW_003726605_1, Position,0,1000,2000,3000,4000,5000,6000,7000,8000,9000,10000,11000,12000,13000,14000,15000,16000,17000,18000,19000,20000,21000,22000,23000,24000,25000,26000,27000,28000,29000,30000,31000,32000,33000,34000,35000,36000,37000,38000,39000,40000,41000
NW_003726605_1,Count,0,0,0,0,0,0,0,0,0,0,0,0,0,0,0,2,0,1,0,6,0,0,0,0,0,0,0,0,0,0,0,0,0,0,0,0,0,0,0,0,0,0

	
```

```
		  

		NW_003726606_1, Position,0,1000,2000,3000,4000,5000,6000,7000,8000,9000,10000,11000,12000,13000,14000,15000,16000,17000,18000,19000,20000,21000,22000,23000,24000,25000,26000,27000,28000,29000,30000,31000,32000,33000,34000,35000,36000,37000,38000,39000,40000,41000,42000,43000,44000,45000,46000
NW_003726606_1,Count,0,0,0,0,0,0,0,0,0,0,0,0,0,0,0,0,0,0,0,0,0,0,0,0,0,0,0,0,1,0,0,0,0,0,0,0,0,0,0,0,0,0,0,0,0,0,0

	
```

```
		  

		NW_003726611_1, Position,0,100,200,300,400,500,600,700,800,900,1000,1100,1200,1300,1400,1500,1600,1700,1800,1900,2000,2100,2200,2300,2400,2500,2600,2700,2800,2900,3000,3100,3200,3300,3400,3500,3600,3700,3800,3900,4000,4100,4200,4300,4400,4500,4600,4700,4800,4900,5000,5100,5200,5300,5400,5500,5600,5700,5800,5900,6000,6100,6200,6300,6400,6500,6600,6700,6800,6900,7000,7100,7200,7300,7400,7500,7600,7700,7800,7900,8000,8100,8200,8300,8400,8500,8600,8700,8800,8900,9000,9100,9200,9300,9400,9500,9600,9700,9800,9900,10000,10100,10200,10300,10400,10500,10600,10700,10800,10900,11000,11100,11200,11300,11400,11500,11600,11700,11800,11900,12000,12100,12200,12300,12400,12500,12600,12700,12800,12900,13000,13100,13200,13300,13400,13500,13600,13700,13800,13900,14000,14100,14200,14300,14400,14500,14600,14700,14800,14900,15000,15100,15200,15300,15400,15500,15600,15700,15800,15900,16000,16100,16200,16300,16400,16500,16600,16700,16800,16900,17000,17100,17200,17300,17400,17500,17600,17700,17800,17900,18000,18100,18200,18300,18400,18500,18600,18700,18800,18900,19000,19100,19200,19300,19400,19500,19600,19700,19800,19900,20000,20100,20200,20300,20400,20500,20600,20700,20800,20900,21000,21100,21200,21300,21400,21500,21600,21700,21800,21900,22000,22100,22200,22300,22400,22500,22600,22700,22800,22900,23000,23100,23200,23300,23400,23500,23600,23700,23800,23900,24000,24100,24200,24300,24400,24500,24600,24700,24800,24900,25000,25100,25200,25300,25400,25500,25600,25700,25800,25900,26000
NW_003726611_1,Count,0,0,0,0,0,0,0,0,0,0,0,0,0,0,0,0,0,0,0,0,0,0,0,0,0,0,0,0,0,0,0,0,0,0,0,0,0,0,0,0,0,0,0,0,0,0,0,0,0,0,0,0,0,0,0,0,0,0,0,0,0,0,0,0,0,0,0,0,0,0,0,0,0,0,0,0,0,0,0,0,0,0,0,0,0,0,0,0,0,0,0,0,0,0,0,0,1,4,0,2,1,0,0,0,0,0,0,0,0,0,0,0,0,0,0,0,0,0,0,0,0,0,0,0,0,0,0,0,0,0,0,0,0,0,0,0,0,0,0,0,0,0,0,0,0,0,0,0,0,0,0,0,0,0,0,0,0,0,0,0,0,0,0,0,0,0,0,0,0,0,0,0,0,0,0,0,0,0,0,0,0,0,0,0,0,0,0,0,0,0,0,0,0,0,0,0,0,0,0,0,0,0,0,0,0,0,0,0,0,0,0,0,0,0,0,0,0,0,0,0,0,0,0,0,0,0,0,0,0,0,0,0,0,0,0,0,0,0,0,0,0,0,0,0,0,0,0,0,0,0,0,0,0,0,0,0,0,0,0,0,0

	
```

```
		  

		NW_003726613_1, Position,0,100,200,300,400,500,600,700,800,900,1000,1100,1200,1300,1400,1500,1600,1700,1800,1900,2000,2100,2200,2300,2400,2500,2600,2700,2800,2900,3000,3100,3200,3300,3400,3500,3600,3700,3800,3900,4000,4100,4200,4300,4400,4500,4600,4700,4800,4900,5000,5100,5200,5300,5400,5500,5600,5700,5800,5900,6000,6100,6200,6300,6400,6500,6600,6700,6800,6900,7000,7100,7200,7300,7400,7500,7600,7700,7800,7900,8000,8100,8200,8300,8400,8500,8600,8700,8800,8900,9000,9100,9200,9300,9400,9500,9600,9700,9800,9900,10000,10100,10200,10300,10400,10500,10600,10700,10800,10900,11000,11100,11200,11300,11400,11500,11600,11700,11800,11900,12000,12100,12200,12300,12400,12500,12600,12700,12800,12900,13000,13100,13200,13300,13400,13500,13600,13700,13800,13900,14000,14100,14200,14300,14400,14500,14600,14700,14800,14900,15000,15100,15200,15300,15400,15500,15600,15700,15800,15900,16000,16100,16200,16300,16400,16500,16600,16700,16800,16900,17000,17100,17200,17300,17400,17500,17600,17700,17800,17900,18000,18100,18200,18300,18400,18500,18600,18700,18800,18900,19000,19100,19200,19300,19400,19500,19600,19700,19800,19900,20000,20100,20200,20300,20400,20500,20600,20700,20800,20900,21000,21100,21200,21300,21400,21500,21600,21700,21800,21900,22000,22100,22200,22300,22400,22500,22600,22700,22800,22900,23000,23100,23200,23300,23400,23500,23600,23700,23800,23900,24000,24100,24200,24300,24400,24500,24600,24700,24800,24900,25000,25100,25200,25300,25400,25500,25600
NW_003726613_1,Count,0,0,0,0,0,0,0,0,0,0,0,0,0,1,0,0,0,1,4,2,1,0,0,1,0,0,0,0,1,0,0,0,0,0,0,0,0,0,0,0,0,0,0,0,1,3,1,0,0,0,0,0,0,0,0,1,1,2,0,0,0,0,1,0,0,0,1,1,0,1,0,2,0,2,0,0,0,1,0,0,0,2,0,0,0,0,0,0,1,0,1,1,0,1,0,1,0,1,0,0,0,0,1,0,1,1,2,1,2,4,0,1,1,0,1,0,0,0,0,0,0,0,0,1,0,0,1,0,0,0,0,0,0,0,0,0,0,0,0,0,0,0,0,0,2,0,0,0,1,1,1,0,0,0,0,0,2,0,1,2,0,1,0,0,0,0,0,0,1,0,0,1,1,2,0,0,0,0,1,0,0,1,0,1,1,0,0,2,0,3,0,1,0,1,2,0,1,1,0,0,0,0,1,0,0,0,0,0,0,2,2,1,1,0,2,0,1,1,0,0,1,0,0,0,0,1,0,1,0,0,0,2,0,2,0,0,0,0,0,0,1,2,0,0,0,0,0,0,0,0,0,0,0,0,0,0,0

	
```

```
		  

		NW_003726614_1, Position,0,1000,2000,3000,4000,5000,6000,7000,8000,9000,10000,11000,12000,13000,14000,15000,16000,17000,18000,19000,20000,21000,22000,23000,24000,25000,26000,27000,28000,29000,30000,31000,32000,33000,34000,35000,36000,37000,38000,39000,40000,41000,42000,43000,44000,45000
NW_003726614_1,Count,0,0,0,0,0,0,0,0,0,0,0,0,0,0,0,0,2,0,0,0,0,0,0,1,0,0,0,0,0,0,0,0,0,0,0,0,0,0,0,0,0,1,0,0,0,0

	
```

```
		  

		NW_003726619_1, Position,0,100,200,300,400,500,600,700,800,900,1000,1100,1200,1300,1400,1500,1600,1700,1800,1900,2000,2100,2200,2300,2400,2500,2600,2700,2800,2900,3000,3100,3200,3300,3400,3500,3600,3700,3800,3900,4000,4100,4200,4300,4400,4500,4600,4700,4800,4900,5000,5100,5200,5300,5400,5500,5600,5700,5800,5900,6000,6100,6200,6300,6400,6500,6600,6700,6800,6900,7000,7100,7200,7300,7400,7500,7600,7700,7800,7900,8000,8100,8200,8300,8400,8500,8600,8700,8800,8900,9000,9100,9200,9300,9400,9500,9600,9700,9800,9900,10000,10100,10200,10300,10400,10500,10600,10700,10800,10900,11000,11100,11200,11300,11400,11500,11600,11700,11800,11900,12000,12100,12200,12300,12400,12500,12600,12700,12800,12900,13000,13100,13200,13300,13400,13500,13600,13700,13800,13900,14000,14100,14200,14300,14400,14500,14600,14700,14800,14900,15000,15100,15200,15300,15400,15500,15600,15700,15800,15900,16000,16100,16200,16300,16400,16500,16600,16700,16800,16900,17000,17100,17200,17300,17400,17500,17600,17700,17800,17900,18000,18100,18200,18300,18400,18500,18600,18700,18800,18900,19000,19100,19200,19300,19400,19500,19600,19700,19800,19900,20000,20100,20200,20300,20400,20500,20600,20700,20800,20900,21000,21100,21200,21300,21400,21500,21600,21700,21800,21900,22000,22100,22200,22300,22400,22500,22600,22700,22800,22900,23000,23100,23200,23300,23400,23500,23600,23700,23800,23900,24000,24100,24200,24300,24400,24500,24600,24700,24800,24900,25000,25100,25200,25300,25400,25500
NW_003726619_1,Count,0,0,0,0,0,0,0,0,0,0,0,0,0,0,0,0,0,0,0,0,0,0,0,0,0,0,0,0,0,0,0,0,0,0,0,0,0,0,0,0,0,0,0,0,0,0,0,0,0,0,0,0,0,0,0,0,0,0,0,0,0,0,0,0,0,0,0,0,0,0,0,0,0,0,0,0,0,0,0,0,0,0,0,0,0,0,0,0,0,0,0,0,0,0,0,0,0,0,0,0,0,0,0,0,0,0,0,0,0,0,0,0,0,0,0,0,0,0,0,0,0,0,0,0,0,0,0,0,0,0,0,0,0,0,0,0,0,0,0,0,0,0,0,0,0,0,0,0,0,0,0,0,0,0,0,0,0,0,0,0,0,0,0,0,0,0,0,0,0,0,0,0,0,0,0,0,0,0,0,0,0,0,0,0,0,0,0,0,0,0,0,0,0,0,0,0,0,0,0,0,0,0,0,0,0,0,0,0,0,0,0,0,0,0,0,0,0,3,0,0,0,0,0,0,0,0,0,0,0,0,0,0,0,0,0,0,0,0,0,0,0,0,0,0,0,0,0,0,0,0,0,0,0,0,0,0

	
```

```
		  

		NW_003726620_1, Position,0,1000,2000,3000,4000,5000,6000,7000,8000,9000,10000,11000,12000,13000,14000,15000,16000,17000,18000,19000,20000,21000,22000,23000,24000,25000,26000,27000,28000,29000,30000,31000,32000,33000,34000,35000,36000,37000
NW_003726620_1,Count,0,0,0,0,0,0,0,0,0,0,0,0,0,0,0,0,0,0,0,0,0,0,0,0,0,0,0,0,0,0,0,3,0,0,0,0,0,0

	
```

```
		  

		NW_003726630_1, Position,0,100,200,300,400,500,600,700,800,900,1000,1100,1200,1300,1400,1500,1600,1700,1800,1900,2000,2100,2200,2300,2400,2500,2600,2700,2800,2900,3000,3100,3200,3300,3400,3500,3600,3700,3800,3900,4000,4100,4200,4300,4400,4500,4600,4700,4800,4900,5000,5100,5200,5300,5400,5500,5600,5700,5800,5900,6000,6100,6200,6300,6400,6500,6600,6700,6800,6900,7000,7100,7200,7300,7400,7500,7600,7700,7800,7900,8000,8100,8200,8300,8400,8500,8600,8700,8800,8900,9000,9100,9200,9300,9400,9500,9600,9700,9800,9900,10000,10100,10200,10300,10400,10500,10600,10700,10800,10900,11000,11100,11200,11300,11400,11500,11600,11700,11800,11900,12000,12100,12200,12300,12400,12500,12600,12700,12800,12900,13000,13100,13200,13300,13400,13500,13600,13700,13800,13900,14000,14100,14200,14300,14400,14500,14600,14700,14800,14900,15000,15100,15200,15300,15400,15500,15600,15700,15800,15900,16000,16100,16200,16300,16400,16500,16600,16700,16800,16900,17000,17100,17200,17300,17400,17500,17600,17700,17800,17900,18000,18100,18200,18300,18400,18500,18600,18700,18800,18900,19000,19100,19200,19300,19400,19500,19600,19700,19800,19900,20000,20100,20200,20300,20400,20500,20600,20700,20800,20900,21000,21100,21200,21300,21400,21500,21600,21700,21800,21900,22000,22100,22200,22300,22400,22500,22600,22700,22800,22900,23000,23100,23200,23300,23400,23500,23600,23700,23800,23900,24000,24100,24200,24300,24400,24500,24600,24700,24800,24900,25000,25100,25200,25300,25400,25500
NW_003726630_1,Count,0,0,0,0,0,0,0,0,0,0,0,0,0,0,0,0,0,0,0,0,0,0,0,0,0,0,0,0,0,0,0,0,0,0,0,0,0,0,0,0,0,0,0,0,0,0,3,1,0,0,0,0,0,0,0,0,0,0,0,0,0,0,0,0,0,0,0,0,0,0,0,0,0,0,0,0,0,0,0,0,0,0,0,0,0,0,0,0,0,0,0,0,1,0,0,0,0,0,0,0,0,0,0,0,0,0,0,0,0,0,0,0,0,0,0,0,0,0,0,0,0,0,0,0,0,0,0,0,0,0,0,0,0,0,0,0,0,0,0,0,0,0,0,0,0,0,0,0,0,0,0,0,0,0,0,0,0,0,0,0,0,0,0,0,0,0,0,0,0,0,0,0,0,0,0,0,0,0,0,0,0,0,0,0,0,0,0,0,0,0,0,0,0,0,0,0,0,0,0,0,0,0,0,0,0,0,0,0,0,0,0,0,0,0,0,0,0,0,0,0,0,0,0,0,0,0,0,0,0,0,0,0,1,0,0,0,0,0,0,0,0,0,0,0,0,0,0,0,0,0,0,0,0,0,0,0

	
```

```
		  

		NW_003726632_1, Position,0,100,200,300,400,500,600,700,800,900,1000,1100,1200,1300,1400,1500,1600,1700,1800,1900,2000,2100,2200,2300,2400,2500,2600,2700,2800,2900,3000,3100,3200,3300,3400,3500,3600,3700,3800,3900,4000,4100,4200,4300,4400,4500,4600,4700,4800,4900,5000,5100,5200,5300,5400,5500,5600,5700,5800,5900,6000,6100,6200,6300,6400,6500,6600,6700,6800,6900,7000,7100,7200,7300,7400,7500,7600,7700,7800,7900,8000,8100,8200,8300,8400,8500,8600,8700,8800,8900,9000,9100,9200,9300,9400,9500,9600,9700,9800,9900,10000,10100,10200,10300,10400,10500,10600,10700,10800,10900,11000,11100,11200,11300,11400,11500,11600,11700,11800,11900,12000,12100,12200,12300,12400,12500,12600,12700,12800,12900,13000,13100,13200,13300,13400,13500,13600,13700,13800,13900,14000,14100,14200,14300,14400,14500,14600,14700,14800,14900,15000,15100,15200,15300,15400,15500,15600,15700,15800,15900,16000,16100,16200,16300,16400,16500,16600,16700,16800,16900,17000,17100,17200,17300,17400,17500,17600,17700,17800,17900,18000,18100,18200,18300,18400,18500,18600,18700,18800,18900,19000,19100,19200,19300,19400,19500,19600,19700,19800,19900,20000,20100,20200,20300,20400,20500,20600,20700,20800,20900,21000,21100,21200,21300,21400,21500,21600,21700,21800,21900,22000,22100,22200,22300,22400,22500,22600,22700,22800,22900,23000,23100,23200,23300,23400,23500,23600,23700,23800,23900,24000,24100,24200,24300,24400,24500,24600,24700,24800
NW_003726632_1,Count,0,0,0,0,0,0,0,0,0,0,0,0,0,0,0,0,0,0,0,0,0,0,0,1,1,0,0,0,0,0,0,0,0,0,0,0,0,0,0,0,0,0,0,0,0,0,0,0,0,0,0,0,1,1,0,0,0,0,0,0,1,2,2,1,2,0,0,0,0,0,0,0,0,0,0,0,0,0,0,2,0,1,0,0,0,0,0,0,0,0,0,0,0,0,0,0,0,0,0,0,0,0,0,2,1,1,0,0,0,1,0,2,1,0,0,0,1,1,2,0,0,0,0,1,0,0,0,0,0,0,0,0,0,1,0,0,0,1,0,0,1,0,0,0,1,0,0,0,0,0,0,0,0,0,2,0,1,1,2,0,0,0,0,0,0,0,0,0,0,0,0,0,2,2,1,0,0,0,0,0,0,1,3,0,0,1,0,2,2,1,1,0,1,0,1,0,0,0,0,0,0,0,0,0,0,0,0,0,0,0,0,0,0,0,0,0,0,0,0,0,0,0,0,0,0,0,0,0,0,0,0,0,0,0,1,0,0,0,0,0,0,0,0,0,0,0,0,0,0

	
```

```
		  

		NW_003726636_1, Position,0,100,200,300,400,500,600,700,800,900,1000,1100,1200,1300,1400,1500,1600,1700,1800,1900,2000,2100,2200,2300,2400,2500,2600,2700,2800,2900,3000,3100,3200,3300,3400,3500,3600,3700,3800,3900,4000,4100,4200,4300,4400,4500,4600,4700,4800,4900,5000,5100,5200,5300,5400,5500,5600,5700,5800,5900,6000,6100,6200,6300,6400,6500,6600,6700,6800,6900,7000,7100,7200,7300,7400,7500,7600,7700,7800,7900,8000,8100,8200,8300,8400,8500,8600,8700,8800,8900,9000,9100,9200,9300,9400,9500,9600,9700,9800,9900,10000,10100,10200,10300,10400,10500,10600,10700,10800,10900,11000,11100,11200,11300,11400,11500,11600,11700,11800,11900,12000,12100,12200,12300,12400,12500,12600,12700,12800,12900,13000,13100,13200,13300,13400,13500,13600,13700,13800,13900,14000,14100,14200,14300,14400,14500,14600,14700,14800,14900,15000,15100,15200,15300,15400,15500,15600,15700,15800,15900,16000,16100,16200,16300,16400,16500,16600,16700,16800,16900,17000,17100,17200,17300,17400,17500,17600,17700,17800,17900,18000,18100,18200,18300,18400,18500,18600,18700,18800,18900,19000,19100,19200,19300,19400,19500,19600,19700,19800,19900,20000,20100,20200,20300,20400,20500,20600,20700,20800,20900,21000,21100,21200,21300,21400,21500,21600,21700,21800,21900,22000,22100,22200,22300,22400,22500,22600,22700,22800,22900,23000,23100,23200,23300,23400,23500,23600,23700,23800,23900,24000,24100,24200,24300,24400,24500,24600
NW_003726636_1,Count,0,1,0,2,1,3,1,0,3,0,0,0,0,2,0,0,0,0,1,1,2,2,0,1,2,0,2,1,2,3,0,2,1,3,0,1,0,0,0,1,0,0,0,0,0,1,2,0,0,1,2,0,3,0,2,0,1,1,2,2,0,1,2,0,1,1,3,1,0,1,2,2,0,0,1,1,2,1,1,2,1,1,1,1,1,0,1,3,1,1,4,0,0,0,0,2,1,2,2,1,0,2,1,3,1,2,0,0,2,1,1,0,3,0,0,3,3,1,3,1,1,0,2,1,4,2,0,2,1,4,2,2,2,3,2,1,3,0,3,0,2,0,1,0,0,1,2,0,0,0,0,0,0,0,0,0,0,0,0,0,0,0,0,0,0,0,2,0,0,0,0,0,0,0,0,0,0,0,0,0,0,0,0,0,0,0,2,1,0,3,0,0,0,1,0,0,0,1,0,1,0,0,0,0,0,0,0,0,0,0,0,0,0,0,0,0,0,0,0,0,0,0,0,0,0,0,1,2,0,3,0,0,0,0,0,0,0,0,0,0,0,0,0,0,0,0,0

	
```

```
		  

		NW_003726637_1, Position,0,100,200,300,400,500,600,700,800,900,1000,1100,1200,1300,1400,1500,1600,1700,1800,1900,2000,2100,2200,2300,2400,2500,2600,2700,2800,2900,3000,3100,3200,3300,3400,3500,3600,3700,3800,3900,4000,4100,4200,4300,4400,4500,4600,4700,4800,4900,5000,5100,5200,5300,5400,5500,5600,5700,5800,5900,6000,6100,6200,6300,6400,6500,6600,6700,6800,6900,7000,7100,7200,7300,7400,7500,7600,7700,7800,7900,8000,8100,8200,8300,8400,8500,8600,8700,8800,8900,9000,9100,9200,9300,9400,9500,9600,9700,9800,9900,10000,10100,10200,10300,10400,10500,10600,10700,10800,10900,11000,11100,11200,11300,11400,11500,11600,11700,11800,11900,12000,12100,12200,12300,12400,12500,12600,12700,12800,12900,13000,13100,13200,13300,13400,13500,13600,13700,13800,13900,14000,14100,14200,14300,14400,14500,14600,14700,14800,14900,15000,15100,15200,15300,15400,15500,15600,15700,15800,15900,16000,16100,16200,16300,16400,16500,16600,16700,16800,16900,17000,17100,17200,17300,17400,17500,17600,17700,17800,17900,18000,18100,18200,18300,18400,18500,18600,18700,18800,18900,19000,19100,19200,19300,19400,19500,19600,19700,19800,19900,20000,20100,20200,20300,20400,20500,20600,20700,20800,20900,21000,21100,21200,21300,21400,21500,21600,21700,21800,21900,22000,22100,22200,22300,22400,22500,22600,22700,22800,22900,23000,23100,23200,23300,23400,23500,23600,23700,23800,23900,24000,24100,24200,24300,24400,24500,24600,24700,24800
NW_003726637_1,Count,1,1,2,0,0,0,0,2,0,3,3,0,0,1,0,2,0,0,1,2,2,0,0,0,2,1,1,1,1,2,0,4,0,0,0,3,1,0,0,0,0,0,1,1,0,0,0,0,0,0,0,0,0,0,0,0,0,0,0,0,0,0,0,0,0,0,0,0,0,0,0,0,0,0,0,0,0,0,0,0,0,0,0,0,0,0,0,0,0,0,0,0,0,0,0,0,0,0,0,0,0,0,0,0,0,0,0,0,0,0,0,0,0,0,0,0,0,0,0,0,0,0,0,0,0,0,0,0,0,0,0,0,0,0,0,0,0,0,0,0,0,0,0,0,0,0,0,0,0,0,0,0,0,0,0,0,0,0,0,0,0,0,0,0,0,0,0,0,0,0,0,0,0,0,0,0,0,0,0,0,0,0,0,0,0,0,0,0,0,0,0,0,0,0,0,0,0,0,0,0,0,0,0,0,0,0,0,0,0,0,0,0,0,0,0,0,0,0,0,0,0,0,0,0,0,0,4,0,1,2,2,1,2,3,0,0,1,3,2,1,0,3,1,2,2,3,0,0,0

	
```

```
		  

		NW_003726638_1, Position,0,1000,2000,3000,4000,5000,6000,7000,8000,9000,10000,11000,12000,13000,14000,15000,16000,17000,18000,19000,20000,21000,22000,23000,24000,25000,26000,27000,28000,29000,30000,31000,32000,33000
NW_003726638_1,Count,0,0,0,0,0,0,0,0,0,0,0,0,0,0,0,0,0,0,3,5,0,0,0,0,0,0,0,0,0,0,0,0,0,0

	
```

```
		  

		NW_003726646_1, Position,0,100,200,300,400,500,600,700,800,900,1000,1100,1200,1300,1400,1500,1600,1700,1800,1900,2000,2100,2200,2300,2400,2500,2600,2700,2800,2900,3000,3100,3200,3300,3400,3500,3600,3700,3800,3900,4000,4100,4200,4300,4400,4500,4600,4700,4800,4900,5000,5100,5200,5300,5400,5500,5600,5700,5800,5900,6000,6100,6200,6300,6400,6500,6600,6700,6800,6900,7000,7100,7200,7300,7400,7500,7600,7700,7800,7900,8000,8100,8200,8300,8400,8500,8600,8700,8800,8900,9000,9100,9200,9300,9400,9500,9600,9700,9800,9900,10000,10100,10200,10300,10400,10500,10600,10700,10800,10900,11000,11100,11200,11300,11400,11500,11600,11700,11800,11900,12000,12100,12200,12300,12400,12500,12600,12700,12800,12900,13000,13100,13200,13300,13400,13500,13600,13700,13800,13900,14000,14100,14200,14300,14400,14500,14600,14700,14800,14900,15000,15100,15200,15300,15400,15500,15600,15700,15800,15900,16000,16100,16200,16300,16400,16500,16600,16700,16800,16900,17000,17100,17200,17300,17400,17500,17600,17700,17800,17900,18000,18100,18200,18300,18400,18500,18600,18700,18800,18900,19000,19100,19200,19300,19400,19500,19600,19700,19800,19900,20000,20100,20200,20300,20400,20500,20600,20700,20800,20900,21000,21100,21200,21300,21400,21500,21600,21700,21800,21900,22000,22100,22200,22300,22400,22500,22600,22700,22800,22900,23000,23100,23200,23300,23400,23500,23600,23700,23800,23900,24000,24100
NW_003726646_1,Count,0,0,0,0,0,0,3,1,0,2,1,2,1,0,0,0,0,0,0,0,0,0,0,0,0,0,0,0,0,0,0,0,0,0,0,0,0,0,0,0,0,0,0,0,0,0,0,1,3,0,0,1,0,0,0,0,0,0,0,0,0,0,0,0,0,0,0,0,0,0,0,0,0,0,0,0,0,0,0,0,0,0,0,0,0,0,0,0,0,0,0,0,0,0,0,0,0,0,0,0,0,0,1,1,0,0,0,0,0,0,0,0,0,0,0,0,0,0,0,0,0,0,0,0,0,0,0,0,0,0,0,0,0,0,0,0,0,0,0,0,1,1,1,1,0,0,0,0,0,0,0,0,0,0,0,0,0,0,0,0,0,0,2,0,0,0,0,0,0,0,0,0,1,2,2,0,2,0,0,0,0,0,0,0,0,0,0,0,0,0,0,0,0,0,0,0,0,0,0,0,0,0,0,0,0,0,0,0,0,0,0,0,0,0,0,0,0,0,0,0,0,0,0,0,0,0,0,0,0,0,0,0,0,0,0,0,0,0,0,0,0,0

	
```

```
		  

		NW_003726647_1, Position,0,1000,2000,3000,4000,5000,6000,7000,8000,9000,10000,11000,12000,13000,14000,15000,16000,17000,18000,19000,20000,21000,22000,23000,24000,25000,26000,27000,28000,29000,30000,31000,32000,33000,34000,35000,36000,37000,38000,39000,40000
NW_003726647_1,Count,0,0,0,0,0,0,0,0,0,0,0,0,0,3,0,0,0,0,0,7,0,0,0,8,0,0,0,6,0,1,11,6,0,0,0,0,0,0,12,5,0

	
```

```
		  

		NW_003726649_1, Position,0,1000,2000,3000,4000,5000,6000,7000,8000,9000,10000,11000,12000,13000,14000,15000,16000,17000,18000,19000,20000,21000,22000,23000,24000,25000,26000,27000,28000,29000,30000,31000,32000,33000,34000,35000,36000
NW_003726649_1,Count,0,0,0,0,0,0,0,0,0,0,0,0,0,0,0,0,0,0,0,0,0,0,0,0,0,0,2,0,0,0,0,0,0,0,0,0,0

	
```

```
		  

		NW_003726650_1, Position,0,100,200,300,400,500,600,700,800,900,1000,1100,1200,1300,1400,1500,1600,1700,1800,1900,2000,2100,2200,2300,2400,2500,2600,2700,2800,2900,3000,3100,3200,3300,3400,3500,3600,3700,3800,3900,4000,4100,4200,4300,4400,4500,4600,4700,4800,4900,5000,5100,5200,5300,5400,5500,5600,5700,5800,5900,6000,6100,6200,6300,6400,6500,6600,6700,6800,6900,7000,7100,7200,7300,7400,7500,7600,7700,7800,7900,8000,8100,8200,8300,8400,8500,8600,8700,8800,8900,9000,9100,9200,9300,9400,9500,9600,9700,9800,9900,10000,10100,10200,10300,10400,10500,10600,10700,10800,10900,11000,11100,11200,11300,11400,11500,11600,11700,11800,11900,12000,12100,12200,12300,12400,12500,12600,12700,12800,12900,13000,13100,13200,13300,13400,13500,13600,13700,13800,13900,14000,14100,14200,14300,14400,14500,14600,14700,14800,14900,15000,15100,15200,15300,15400,15500,15600,15700,15800,15900,16000,16100,16200,16300,16400,16500,16600,16700,16800,16900,17000,17100,17200,17300,17400,17500,17600,17700,17800,17900,18000,18100,18200,18300,18400,18500,18600,18700,18800,18900,19000,19100,19200,19300,19400,19500,19600,19700,19800,19900,20000,20100,20200,20300,20400,20500,20600,20700,20800,20900,21000,21100,21200,21300,21400,21500,21600,21700,21800,21900,22000,22100,22200,22300,22400,22500,22600,22700,22800,22900,23000,23100,23200,23300,23400,23500,23600,23700,23800
NW_003726650_1,Count,0,0,0,0,0,0,0,0,0,0,0,0,0,0,0,0,0,0,0,0,0,0,0,0,0,0,0,0,0,0,0,0,0,0,0,0,0,0,0,0,0,0,0,0,0,0,0,0,0,0,0,0,0,0,0,1,2,0,0,0,0,0,0,0,0,0,0,0,0,0,0,0,0,2,3,0,1,0,0,0,0,0,0,0,0,0,0,0,0,0,0,0,0,0,0,0,0,0,0,0,0,0,0,0,0,0,0,0,0,0,0,0,0,0,0,0,0,0,0,0,0,0,0,0,0,0,0,0,0,0,0,0,0,0,0,0,0,0,0,0,0,0,0,0,0,0,0,2,0,0,0,0,0,0,0,0,0,0,0,0,0,0,0,0,0,0,0,0,0,0,0,0,0,0,0,0,0,0,0,3,1,0,0,0,0,0,0,0,0,0,0,0,0,0,0,0,0,0,0,0,0,0,0,0,0,0,0,0,0,0,0,0,0,0,0,3,2,3,0,0,0,0,0,0,0,0,0,0,0,0,0,0,0,0,0,0,0,0,0

	
```

```
		  

		NW_003726653_1, Position,0,100,200,300,400,500,600,700,800,900,1000,1100,1200,1300,1400,1500,1600,1700,1800,1900,2000,2100,2200,2300,2400,2500,2600,2700,2800,2900,3000,3100,3200,3300,3400,3500,3600,3700,3800,3900,4000,4100,4200,4300,4400,4500,4600,4700,4800,4900,5000,5100,5200,5300,5400,5500,5600,5700,5800,5900,6000,6100,6200,6300,6400,6500,6600,6700,6800,6900,7000,7100,7200,7300,7400,7500,7600,7700,7800,7900,8000,8100,8200,8300,8400,8500,8600,8700,8800,8900,9000,9100,9200,9300,9400,9500,9600,9700,9800,9900,10000,10100,10200,10300,10400,10500,10600,10700,10800,10900,11000,11100,11200,11300,11400,11500,11600,11700,11800,11900,12000,12100,12200,12300,12400,12500,12600,12700,12800,12900,13000,13100,13200,13300,13400,13500,13600,13700,13800,13900,14000,14100,14200,14300,14400,14500,14600,14700,14800,14900,15000,15100,15200,15300,15400,15500,15600,15700,15800,15900,16000,16100,16200,16300,16400,16500,16600,16700,16800,16900,17000,17100,17200,17300,17400,17500,17600,17700,17800,17900,18000,18100,18200,18300,18400,18500,18600,18700,18800,18900,19000,19100,19200,19300,19400,19500,19600,19700,19800,19900,20000,20100,20200,20300,20400,20500,20600,20700,20800,20900,21000,21100,21200,21300,21400,21500,21600,21700,21800,21900,22000,22100,22200,22300,22400,22500,22600,22700,22800,22900,23000,23100,23200,23300,23400,23500,23600,23700,23800
NW_003726653_1,Count,0,0,0,0,0,0,0,0,0,0,0,0,0,0,0,0,0,0,0,0,0,0,0,0,0,0,0,0,0,0,0,0,0,0,0,0,0,0,0,0,0,0,0,0,0,0,0,0,0,0,0,0,0,0,0,0,0,0,0,0,0,0,0,0,0,0,0,0,0,0,0,0,0,0,0,0,0,0,0,0,0,0,0,0,0,0,0,0,0,0,0,0,0,0,0,0,0,0,0,0,0,0,0,0,0,0,0,0,0,0,0,0,0,0,0,0,0,0,0,0,0,0,0,0,0,0,0,0,0,0,0,0,0,0,0,0,0,0,0,0,0,0,0,0,0,0,0,0,0,0,0,0,0,0,0,0,0,0,0,0,0,0,0,0,0,0,0,0,0,0,0,0,0,0,0,0,0,0,0,0,0,0,0,0,0,0,0,0,0,0,0,0,0,0,0,1,0,0,0,0,0,0,0,0,0,0,0,0,0,0,0,0,0,0,0,0,0,0,0,0,0,0,0,0,0,0,0,0,0,0,0,0,0,0,0,0,0,0,0

	
```

```
		  

		NW_003726654_1, Position,0,1000,2000,3000,4000,5000,6000,7000,8000,9000,10000,11000,12000,13000,14000,15000,16000,17000,18000,19000,20000,21000,22000,23000,24000,25000,26000,27000,28000,29000,30000,31000,32000,33000,34000,35000,36000
NW_003726654_1,Count,0,0,0,4,0,0,0,0,0,0,0,0,0,0,0,0,0,0,0,0,0,0,0,0,0,0,0,0,0,0,0,0,0,0,0,0,0

	
```

```
		  

		NW_003726663_1, Position,0,100,200,300,400,500,600,700,800,900,1000,1100,1200,1300,1400,1500,1600,1700,1800,1900,2000,2100,2200,2300,2400,2500,2600,2700,2800,2900,3000,3100,3200,3300,3400,3500,3600,3700,3800,3900,4000,4100,4200,4300,4400,4500,4600,4700,4800,4900,5000,5100,5200,5300,5400,5500,5600,5700,5800,5900,6000,6100,6200,6300,6400,6500,6600,6700,6800,6900,7000,7100,7200,7300,7400,7500,7600,7700,7800,7900,8000,8100,8200,8300,8400,8500,8600,8700,8800,8900,9000,9100,9200,9300,9400,9500,9600,9700,9800,9900,10000,10100,10200,10300,10400,10500,10600,10700,10800,10900,11000,11100,11200,11300,11400,11500,11600,11700,11800,11900,12000,12100,12200,12300,12400,12500,12600,12700,12800,12900,13000,13100,13200,13300,13400,13500,13600,13700,13800,13900,14000,14100,14200,14300,14400,14500,14600,14700,14800,14900,15000,15100,15200,15300,15400,15500,15600,15700,15800,15900,16000,16100,16200,16300,16400,16500,16600,16700,16800,16900,17000,17100,17200,17300,17400,17500,17600,17700,17800,17900,18000,18100,18200,18300,18400,18500,18600,18700,18800,18900,19000,19100,19200,19300,19400,19500,19600,19700,19800,19900,20000,20100,20200,20300,20400,20500,20600,20700,20800,20900,21000,21100,21200,21300,21400,21500,21600,21700,21800,21900,22000,22100,22200,22300,22400,22500,22600,22700,22800,22900,23000,23100,23200,23300,23400,23500
NW_003726663_1,Count,0,0,0,0,0,0,0,0,0,0,0,0,0,0,0,0,0,0,0,0,0,0,0,0,0,0,0,0,0,0,0,0,0,0,0,0,0,0,0,0,0,0,0,0,0,0,0,0,0,0,0,0,0,0,0,0,0,0,0,0,0,0,0,0,0,0,0,0,0,0,0,0,0,0,0,0,0,0,0,0,0,0,0,0,0,0,0,0,0,0,0,0,0,1,0,0,0,0,0,0,0,0,0,0,0,0,0,0,0,0,0,0,0,0,0,0,0,0,0,2,4,4,0,0,0,2,2,1,0,0,0,0,0,0,3,1,1,0,0,0,1,1,2,1,2,2,2,1,0,4,0,1,2,2,0,0,0,2,1,0,1,2,0,2,0,0,0,3,1,1,0,0,0,2,1,0,0,0,0,0,0,0,2,3,0,1,1,0,3,1,2,1,0,4,1,4,0,1,1,1,0,1,0,0,0,0,1,0,4,1,2,4,0,0,0,0,0,4,1,2,0,0,0,1,0,2,2,1,1,1,2,1,3,1,1,0

	
```

```
		  

		NW_003726664_1, Position,0,100,200,300,400,500,600,700,800,900,1000,1100,1200,1300,1400,1500,1600,1700,1800,1900,2000,2100,2200,2300,2400,2500,2600,2700,2800,2900,3000,3100,3200,3300,3400,3500,3600,3700,3800,3900,4000,4100,4200,4300,4400,4500,4600,4700,4800,4900,5000,5100,5200,5300,5400,5500,5600,5700,5800,5900,6000,6100,6200,6300,6400,6500,6600,6700,6800,6900,7000,7100,7200,7300,7400,7500,7600,7700,7800,7900,8000,8100,8200,8300,8400,8500,8600,8700,8800,8900,9000,9100,9200,9300,9400,9500,9600,9700,9800,9900,10000,10100,10200,10300,10400,10500,10600,10700,10800,10900,11000,11100,11200,11300,11400,11500,11600,11700,11800,11900,12000,12100,12200,12300,12400,12500,12600,12700,12800,12900,13000,13100,13200,13300,13400,13500,13600,13700,13800,13900,14000,14100,14200,14300,14400,14500,14600,14700,14800,14900,15000,15100,15200,15300,15400,15500,15600,15700,15800,15900,16000,16100,16200,16300,16400,16500,16600,16700,16800,16900,17000,17100,17200,17300,17400,17500,17600,17700,17800,17900,18000,18100,18200,18300,18400,18500,18600,18700,18800,18900,19000,19100,19200,19300,19400,19500,19600,19700,19800,19900,20000,20100,20200,20300,20400,20500,20600,20700,20800,20900,21000,21100,21200,21300,21400,21500,21600,21700,21800,21900,22000,22100,22200,22300,22400,22500,22600,22700,22800,22900,23000,23100,23200,23300,23400,23500,23600,23700,23800,23900,24000,24100,24200,24300,24400,24500,24600,24700,24800,24900,25000,25100,25200,25300,25400,25500,25600,25700,25800,25900,26000,26100,26200,26300,26400,26500,26600,26700
NW_003726664_1,Count,0,0,0,0,0,0,0,0,0,0,0,0,0,0,0,0,0,0,0,0,0,0,0,0,0,0,0,0,0,0,0,0,0,0,0,0,0,0,0,0,0,0,0,0,1,1,0,0,0,0,0,0,0,0,0,0,0,0,0,0,0,0,0,0,0,0,0,0,0,0,0,0,0,1,1,2,0,0,0,0,0,2,0,0,2,0,0,0,0,0,0,0,0,0,0,0,0,0,0,0,0,0,0,0,0,2,0,1,1,0,2,1,1,2,1,0,0,3,1,1,1,1,0,0,0,0,0,0,1,0,2,1,0,2,0,1,0,0,3,0,0,0,1,1,2,1,0,0,2,3,1,2,2,1,1,4,1,1,1,2,0,0,0,0,1,0,0,0,1,0,0,1,1,1,0,0,0,0,1,0,0,0,0,0,0,0,0,0,0,0,0,0,0,0,0,0,0,0,0,0,0,1,3,0,1,0,0,0,0,0,0,0,1,0,0,0,0,0,0,0,0,0,0,0,0,0,0,0,0,0,0,1,0,0,0,0,0,2,2,1,0,0,0,1,1,1,0,0,0,0,0,0,0,0,0,0,0,0,0,0,0,1,1,0,0,0,0,0

	
```

```
		  

		NW_003726665_1, Position,0,100,200,300,400,500,600,700,800,900,1000,1100,1200,1300,1400,1500,1600,1700,1800,1900,2000,2100,2200,2300,2400,2500,2600,2700,2800,2900,3000,3100,3200,3300,3400,3500,3600,3700,3800,3900,4000,4100,4200,4300,4400,4500,4600,4700,4800,4900,5000,5100,5200,5300,5400,5500,5600,5700,5800,5900,6000,6100,6200,6300,6400,6500,6600,6700,6800,6900,7000,7100,7200,7300,7400,7500,7600,7700,7800,7900,8000,8100,8200,8300,8400,8500,8600,8700,8800,8900,9000,9100,9200,9300,9400,9500,9600,9700,9800,9900,10000,10100,10200,10300,10400,10500,10600,10700,10800,10900,11000,11100,11200,11300,11400,11500,11600,11700,11800,11900,12000,12100,12200,12300,12400,12500,12600,12700,12800,12900,13000,13100,13200,13300,13400,13500,13600,13700,13800,13900,14000,14100,14200,14300,14400,14500,14600,14700,14800,14900,15000,15100,15200,15300,15400,15500,15600,15700,15800,15900,16000,16100,16200,16300,16400,16500,16600,16700,16800,16900,17000,17100,17200,17300,17400,17500,17600,17700,17800,17900,18000,18100,18200,18300,18400,18500,18600,18700,18800,18900,19000,19100,19200,19300,19400,19500,19600,19700,19800,19900,20000,20100,20200,20300,20400,20500,20600,20700,20800,20900,21000,21100,21200,21300,21400,21500,21600,21700,21800,21900,22000,22100,22200,22300,22400,22500,22600,22700,22800,22900,23000,23100,23200,23300,23400
NW_003726665_1,Count,0,0,0,0,0,0,0,0,0,0,0,0,0,2,0,0,0,0,0,0,0,0,0,0,0,0,0,0,0,0,0,0,0,0,0,0,0,0,0,0,0,0,0,0,0,0,0,0,0,0,0,0,0,0,0,1,1,1,0,0,0,0,0,0,2,1,0,0,0,0,0,0,0,0,0,0,0,0,0,0,0,1,3,2,0,0,0,0,0,1,0,0,0,0,2,2,1,1,1,0,0,2,1,1,0,0,0,0,0,1,1,0,0,0,0,0,0,0,0,0,0,0,0,0,0,0,0,0,0,3,0,1,1,0,3,1,1,2,0,0,0,0,0,0,0,0,0,0,0,1,2,1,0,0,0,0,0,0,0,0,0,0,0,0,0,0,0,0,0,0,0,0,0,0,0,0,0,0,0,0,0,1,2,2,0,0,0,2,0,2,3,0,1,2,0,1,1,2,0,0,0,0,0,0,0,0,0,1,0,0,0,0,1,0,0,2,0,0,0,0,0,0,0,0,0,0,0,0,0,0,0,0,0,0,0

	
```

```
		  

		NW_003726666_1, Position,0,100,200,300,400,500,600,700,800,900,1000,1100,1200,1300,1400,1500,1600,1700,1800,1900,2000,2100,2200,2300,2400,2500,2600,2700,2800,2900,3000,3100,3200,3300,3400,3500,3600,3700,3800,3900,4000,4100,4200,4300,4400,4500,4600,4700,4800,4900,5000,5100,5200,5300,5400,5500,5600,5700,5800,5900,6000,6100,6200,6300,6400,6500,6600,6700,6800,6900,7000,7100,7200,7300,7400,7500,7600,7700,7800,7900,8000,8100,8200,8300,8400,8500,8600,8700,8800,8900,9000,9100,9200,9300,9400,9500,9600,9700,9800,9900,10000,10100,10200,10300,10400,10500,10600,10700,10800,10900,11000,11100,11200,11300,11400,11500,11600,11700,11800,11900,12000,12100,12200,12300,12400,12500,12600,12700,12800,12900,13000,13100,13200,13300,13400,13500,13600,13700,13800,13900,14000,14100,14200,14300,14400,14500,14600,14700,14800,14900,15000,15100,15200,15300,15400,15500,15600,15700,15800,15900,16000,16100,16200,16300,16400,16500,16600,16700,16800,16900,17000,17100,17200,17300,17400,17500,17600,17700,17800,17900,18000,18100,18200,18300,18400,18500,18600,18700,18800,18900,19000,19100,19200,19300,19400,19500,19600,19700,19800,19900,20000,20100,20200,20300,20400,20500,20600,20700,20800,20900,21000,21100,21200,21300,21400,21500,21600,21700,21800,21900,22000,22100,22200,22300,22400,22500,22600,22700,22800,22900,23000,23100,23200,23300,23400,23500,23600,23700,23800,23900,24000,24100,24200,24300,24400,24500,24600,24700,24800,24900
NW_003726666_1,Count,0,0,0,0,0,1,0,1,2,0,0,4,0,2,2,2,1,1,2,0,2,2,0,0,0,0,0,0,0,0,0,0,0,0,0,0,0,1,2,0,0,0,0,0,0,1,1,0,0,1,2,0,2,2,3,1,0,1,1,0,0,0,0,0,0,0,0,0,0,0,0,0,0,0,0,0,0,1,2,1,1,1,1,0,2,1,0,0,0,0,0,0,0,0,0,0,0,0,0,0,0,0,1,0,0,0,1,1,1,0,0,0,0,0,0,0,0,0,0,0,0,0,0,0,0,0,0,0,0,0,1,0,0,0,0,0,0,2,0,0,0,0,0,0,0,0,3,2,0,3,1,0,0,0,0,0,0,3,1,2,0,3,0,0,0,0,1,2,2,2,3,1,2,1,3,1,1,0,3,1,2,2,2,2,3,3,1,1,2,3,0,2,3,0,0,0,0,0,0,0,1,2,0,3,1,1,1,0,2,2,0,5,2,0,0,1,3,0,4,1,0,3,1,2,0,3,1,2,2,2,0,0,0,0,0,0,0,0,0,0,0,0,0,0,0,0,0,0,0,0

	
```

```
		  

		NW_003726668_1, Position,0,100,200,300,400,500,600,700,800,900,1000,1100,1200,1300,1400,1500,1600,1700,1800,1900,2000,2100,2200,2300,2400,2500,2600,2700,2800,2900,3000,3100,3200,3300,3400,3500,3600,3700,3800,3900,4000,4100,4200,4300,4400,4500,4600,4700,4800,4900,5000,5100,5200,5300,5400,5500,5600,5700,5800,5900,6000,6100,6200,6300,6400,6500,6600,6700,6800,6900,7000,7100,7200,7300,7400,7500,7600,7700,7800,7900,8000,8100,8200,8300,8400,8500,8600,8700,8800,8900,9000,9100,9200,9300,9400,9500,9600,9700,9800,9900,10000,10100,10200,10300,10400,10500,10600,10700,10800,10900,11000,11100,11200,11300,11400,11500,11600,11700,11800,11900,12000,12100,12200,12300,12400,12500,12600,12700,12800,12900,13000,13100,13200,13300,13400,13500,13600,13700,13800,13900,14000,14100,14200,14300,14400,14500,14600,14700,14800,14900,15000,15100,15200,15300,15400,15500,15600,15700,15800,15900,16000,16100,16200,16300,16400,16500,16600,16700,16800,16900,17000,17100,17200,17300,17400,17500,17600,17700,17800,17900,18000,18100,18200,18300,18400,18500,18600,18700,18800,18900,19000,19100,19200,19300,19400,19500,19600,19700,19800,19900,20000,20100,20200,20300,20400,20500,20600,20700,20800,20900,21000,21100,21200,21300,21400,21500,21600,21700,21800,21900,22000,22100,22200,22300,22400,22500,22600,22700,22800,22900,23000,23100,23200,23300
NW_003726668_1,Count,0,0,0,0,0,0,0,0,0,0,0,0,0,0,0,0,0,0,0,0,0,0,0,0,0,0,0,0,0,0,0,0,0,0,0,0,0,0,0,0,0,0,0,0,0,0,0,0,0,0,0,0,0,0,0,0,0,0,0,0,0,0,0,0,0,0,0,0,0,0,0,0,0,0,0,0,2,1,0,0,0,0,0,0,0,0,0,0,0,0,0,0,0,0,0,4,3,0,0,0,0,0,0,0,0,0,0,0,0,0,0,0,0,0,0,0,0,0,0,0,0,0,0,0,0,0,0,0,0,0,0,0,0,0,0,0,0,0,0,0,0,0,0,0,0,0,0,0,0,0,0,0,0,0,0,0,0,0,0,0,0,0,0,0,0,0,0,0,0,0,0,0,0,0,0,0,0,0,0,0,0,0,0,0,0,0,0,0,0,0,0,0,0,0,0,0,0,0,0,0,0,0,0,0,0,0,0,0,0,0,0,0,0,0,0,0,0,0,0,0,0,0,0,0,0,0,0,0,0,0,0,0,0,0

	
```

```
		  

		NW_003726674_1, Position,0,1000,2000,3000,4000,5000,6000,7000,8000,9000,10000,11000,12000,13000,14000,15000,16000,17000,18000,19000,20000,21000,22000,23000,24000,25000,26000,27000,28000,29000,30000,31000,32000,33000,34000,35000
NW_003726674_1,Count,0,0,0,0,0,0,0,0,0,0,0,0,0,2,0,0,0,0,0,0,0,0,0,0,0,0,0,0,0,0,0,0,0,0,0,0

	
```

```
		  

		NW_003726676_1, Position,0,1000,2000,3000,4000,5000,6000,7000,8000,9000,10000,11000,12000,13000,14000,15000,16000,17000,18000,19000,20000,21000,22000,23000,24000,25000,26000,27000,28000,29000,30000,31000,32000,33000,34000,35000,36000,37000
NW_003726676_1,Count,0,0,0,8,0,0,0,0,0,0,0,0,0,0,0,0,0,0,0,0,0,0,0,0,0,0,0,0,0,0,0,0,0,0,0,0,0,0

	
```

```
		  

		NW_003726677_1, Position,0,1000,2000,3000,4000,5000,6000,7000,8000,9000,10000,11000,12000,13000,14000,15000,16000,17000,18000,19000,20000,21000,22000,23000,24000,25000,26000,27000,28000,29000,30000,31000,32000,33000,34000,35000,36000
NW_003726677_1,Count,0,0,0,0,0,0,0,0,0,0,0,0,0,0,0,0,0,0,0,0,0,0,0,0,0,13,19,19,3,0,0,0,0,0,0,0,0

	
```

```
		  

		NW_003726681_1, Position,0,100,200,300,400,500,600,700,800,900,1000,1100,1200,1300,1400,1500,1600,1700,1800,1900,2000,2100,2200,2300,2400,2500,2600,2700,2800,2900,3000,3100,3200,3300,3400,3500,3600,3700,3800,3900,4000,4100,4200,4300,4400,4500,4600,4700,4800,4900,5000,5100,5200,5300,5400,5500,5600,5700,5800,5900,6000,6100,6200,6300,6400,6500,6600,6700,6800,6900,7000,7100,7200,7300,7400,7500,7600,7700,7800,7900,8000,8100,8200,8300,8400,8500,8600,8700,8800,8900,9000,9100,9200,9300,9400,9500,9600,9700,9800,9900,10000,10100,10200,10300,10400,10500,10600,10700,10800,10900,11000,11100,11200,11300,11400,11500,11600,11700,11800,11900,12000,12100,12200,12300,12400,12500,12600,12700,12800,12900,13000,13100,13200,13300,13400,13500,13600,13700,13800,13900,14000,14100,14200,14300,14400,14500,14600,14700,14800,14900,15000,15100,15200,15300,15400,15500,15600,15700,15800,15900,16000,16100,16200,16300,16400,16500,16600,16700,16800,16900,17000,17100,17200,17300,17400,17500,17600,17700,17800,17900,18000,18100,18200,18300,18400,18500,18600,18700,18800,18900,19000,19100,19200,19300,19400,19500,19600,19700,19800,19900,20000,20100,20200,20300,20400,20500,20600,20700,20800,20900,21000,21100,21200,21300,21400,21500,21600,21700,21800,21900,22000,22100,22200,22300,22400,22500,22600,22700,22800,22900,23000,23100,23200,23300,23400,23500,23600,23700,23800,23900
NW_003726681_1,Count,0,1,2,2,0,1,1,3,1,0,1,0,3,1,2,1,1,1,0,2,3,2,2,0,3,0,0,2,0,1,2,3,1,2,0,0,0,3,0,1,0,3,1,1,0,1,2,2,1,2,1,2,0,0,1,0,0,0,2,1,1,2,1,1,0,0,0,1,3,1,4,3,3,2,0,4,1,3,1,2,2,2,1,0,1,2,0,0,0,3,0,0,3,0,1,1,1,1,1,0,1,1,1,1,3,1,0,3,0,2,1,0,0,0,0,1,0,0,0,0,0,0,0,0,0,0,0,2,0,2,0,3,0,0,1,0,0,1,1,2,0,2,0,2,3,2,0,0,0,0,2,0,0,0,1,3,2,0,0,0,0,0,0,1,0,0,1,0,1,0,0,3,1,2,2,0,0,1,0,0,0,0,0,0,0,0,2,0,1,0,1,0,1,0,1,0,0,0,0,0,0,2,2,0,1,0,0,0,1,2,0,0,1,0,0,0,2,1,0,1,0,1,1,0,0,1,0,0,0,0,1,0,1,2,1,2,1,1,2,0

	
```

```
		  

		NW_003726682_1, Position,0,100,200,300,400,500,600,700,800,900,1000,1100,1200,1300,1400,1500,1600,1700,1800,1900,2000,2100,2200,2300,2400,2500,2600,2700,2800,2900,3000,3100,3200,3300,3400,3500,3600,3700,3800,3900,4000,4100,4200,4300,4400,4500,4600,4700,4800,4900,5000,5100,5200,5300,5400,5500,5600,5700,5800,5900,6000,6100,6200,6300,6400,6500,6600,6700,6800,6900,7000,7100,7200,7300,7400,7500,7600,7700,7800,7900,8000,8100,8200,8300,8400,8500,8600,8700,8800,8900,9000,9100,9200,9300,9400,9500,9600,9700,9800,9900,10000,10100,10200,10300,10400,10500,10600,10700,10800,10900,11000,11100,11200,11300,11400,11500,11600,11700,11800,11900,12000,12100,12200,12300,12400,12500,12600,12700,12800,12900,13000,13100,13200,13300,13400,13500,13600,13700,13800,13900,14000,14100,14200,14300,14400,14500,14600,14700,14800,14900,15000,15100,15200,15300,15400,15500,15600,15700,15800,15900,16000,16100,16200,16300,16400,16500,16600,16700,16800,16900,17000,17100,17200,17300,17400,17500,17600,17700,17800,17900,18000,18100,18200,18300,18400,18500,18600,18700,18800,18900,19000,19100,19200,19300,19400,19500,19600,19700,19800,19900,20000,20100,20200,20300,20400,20500,20600,20700,20800,20900,21000,21100,21200,21300,21400,21500,21600,21700,21800,21900,22000,22100,22200,22300,22400,22500,22600,22700,22800,22900,23000
NW_003726682_1,Count,0,1,0,1,0,0,0,0,0,0,0,2,0,0,0,0,0,0,0,2,0,0,0,0,0,0,3,0,0,1,0,0,1,0,1,0,0,0,0,0,2,0,0,0,1,0,2,0,1,0,0,0,0,1,2,1,0,2,4,0,0,1,2,3,0,1,0,0,0,1,2,0,2,1,3,1,0,0,0,0,0,0,0,0,0,0,0,0,0,0,0,0,0,0,0,0,0,0,0,0,0,0,0,0,0,0,0,0,0,0,0,0,0,0,0,0,0,0,0,0,0,0,0,0,0,0,0,0,0,0,0,0,0,0,0,0,0,0,0,0,0,0,0,0,0,0,0,0,0,1,0,0,0,0,0,0,0,0,0,0,0,0,0,0,1,0,0,1,0,1,3,1,0,0,1,1,3,0,0,3,0,0,0,2,0,1,1,0,0,0,0,0,0,0,0,0,0,0,0,0,0,0,0,0,0,0,0,0,0,0,0,0,0,0,0,0,0,0,0,0,0,0,0,0,0,0,0,0,0,0,0

	
```

```
		  

		NW_003726687_1, Position,0,1000,2000,3000,4000,5000,6000,7000,8000,9000,10000,11000,12000,13000,14000,15000,16000,17000,18000,19000,20000,21000,22000,23000,24000,25000,26000,27000,28000,29000,30000,31000,32000,33000,34000,35000,36000,37000
NW_003726687_1,Count,0,0,0,0,0,0,0,0,0,0,0,1,0,0,0,0,0,0,0,0,0,0,1,0,0,0,0,0,3,0,0,0,0,0,0,0,0,0

	
```

```
		  

		NW_003726690_1, Position,0,1000,2000,3000,4000,5000,6000,7000,8000,9000,10000,11000,12000,13000,14000,15000,16000,17000,18000,19000,20000,21000,22000,23000,24000,25000,26000,27000,28000,29000,30000,31000,32000
NW_003726690_1,Count,4,0,0,0,0,0,0,0,0,0,0,0,0,0,0,0,0,0,0,0,0,0,0,0,0,0,0,0,0,0,0,0,0

	
```

```
		  

		NW_003726691_1, Position,0,100,200,300,400,500,600,700,800,900,1000,1100,1200,1300,1400,1500,1600,1700,1800,1900,2000,2100,2200,2300,2400,2500,2600,2700,2800,2900,3000,3100,3200,3300,3400,3500,3600,3700,3800,3900,4000,4100,4200,4300,4400,4500,4600,4700,4800,4900,5000,5100,5200,5300,5400,5500,5600,5700,5800,5900,6000,6100,6200,6300,6400,6500,6600,6700,6800,6900,7000,7100,7200,7300,7400,7500,7600,7700,7800,7900,8000,8100,8200,8300,8400,8500,8600,8700,8800,8900,9000,9100,9200,9300,9400,9500,9600,9700,9800,9900,10000,10100,10200,10300,10400,10500,10600,10700,10800,10900,11000,11100,11200,11300,11400,11500,11600,11700,11800,11900,12000,12100,12200,12300,12400,12500,12600,12700,12800,12900,13000,13100,13200,13300,13400,13500,13600,13700,13800,13900,14000,14100,14200,14300,14400,14500,14600,14700,14800,14900,15000,15100,15200,15300,15400,15500,15600,15700,15800,15900,16000,16100,16200,16300,16400,16500,16600,16700,16800,16900,17000,17100,17200,17300,17400,17500,17600,17700,17800,17900,18000,18100,18200,18300,18400,18500,18600,18700,18800,18900,19000,19100,19200,19300,19400,19500,19600,19700,19800,19900,20000,20100,20200,20300,20400,20500,20600,20700,20800,20900,21000,21100,21200,21300,21400,21500,21600,21700,21800,21900,22000,22100,22200,22300,22400,22500,22600,22700,22800,22900
NW_003726691_1,Count,0,0,0,0,0,0,0,0,0,0,0,0,0,0,0,0,0,0,0,0,0,0,0,0,0,0,0,0,0,0,0,0,0,0,0,0,0,0,0,0,0,0,0,0,0,0,1,0,1,1,2,1,1,1,1,3,0,0,1,1,2,1,2,1,0,0,2,1,0,1,0,0,0,0,0,0,1,2,1,0,0,0,0,0,0,0,0,0,0,0,0,0,0,0,1,0,0,0,0,0,0,0,0,0,0,0,0,0,0,0,0,0,3,1,0,2,0,1,1,0,0,0,0,1,1,2,1,2,1,3,0,2,3,1,0,0,1,0,0,0,0,0,0,0,0,1,1,0,1,1,1,4,0,1,0,3,0,1,0,0,1,1,0,3,1,0,0,1,0,0,1,1,0,0,0,0,0,0,3,1,0,2,0,0,1,0,0,1,0,0,0,0,0,0,0,0,0,0,0,0,0,0,0,0,0,0,0,0,0,0,0,0,0,0,0,0,0,0,0,0,0,0,0,0,0,0,0,0,0,0

	
```

```
		  

		NW_003726697_1, Position,0,100,200,300,400,500,600,700,800,900,1000,1100,1200,1300,1400,1500,1600,1700,1800,1900,2000,2100,2200,2300,2400,2500,2600,2700,2800,2900,3000,3100,3200,3300,3400,3500,3600,3700,3800,3900,4000,4100,4200,4300,4400,4500,4600,4700,4800,4900,5000,5100,5200,5300,5400,5500,5600,5700,5800,5900,6000,6100,6200,6300,6400,6500,6600,6700,6800,6900,7000,7100,7200,7300,7400,7500,7600,7700,7800,7900,8000,8100,8200,8300,8400,8500,8600,8700,8800,8900,9000,9100,9200,9300,9400,9500,9600,9700,9800,9900,10000,10100,10200,10300,10400,10500,10600,10700,10800,10900,11000,11100,11200,11300,11400,11500,11600,11700,11800,11900,12000,12100,12200,12300,12400,12500,12600,12700,12800,12900,13000,13100,13200,13300,13400,13500,13600,13700,13800,13900,14000,14100,14200,14300,14400,14500,14600,14700,14800,14900,15000,15100,15200,15300,15400,15500,15600,15700,15800,15900,16000,16100,16200,16300,16400,16500,16600,16700,16800,16900,17000,17100,17200,17300,17400,17500,17600,17700,17800,17900,18000,18100,18200,18300,18400,18500,18600,18700,18800,18900,19000,19100,19200,19300,19400,19500,19600,19700,19800,19900,20000,20100,20200,20300,20400,20500,20600,20700,20800,20900,21000,21100,21200,21300,21400,21500,21600,21700,21800,21900,22000,22100,22200,22300,22400,22500,22600,22700,22800,22900,23000,23100,23200,23300
NW_003726697_1,Count,0,0,0,0,0,0,0,0,0,0,0,0,0,0,0,0,0,0,0,0,0,0,0,0,0,0,0,0,0,0,0,0,0,0,0,0,0,0,0,0,0,0,0,0,0,0,0,0,0,0,0,0,0,0,0,0,0,0,0,0,0,0,0,0,0,0,0,0,0,0,0,0,0,0,0,0,0,0,0,0,0,0,0,0,0,0,0,0,0,0,0,0,0,0,0,0,0,0,0,0,0,0,0,0,0,0,0,0,0,0,0,0,0,0,0,0,0,0,0,0,0,0,0,0,0,0,0,0,0,0,0,0,0,0,0,0,0,0,0,0,0,0,0,0,0,0,0,0,0,0,0,0,0,0,0,0,0,0,0,0,0,0,0,0,0,0,0,0,0,0,0,0,0,0,0,0,0,0,0,0,0,0,0,0,0,0,0,0,0,0,0,0,0,0,0,0,0,3,0,0,0,0,0,0,0,0,0,0,0,1,1,0,0,0,0,0,0,0,0,0,0,0,0,0,0,0,0,0,0,0,0,0,0,0

	
```

```
		  

		NW_003726702_1, Position,0,1000,2000,3000,4000,5000,6000,7000,8000,9000,10000,11000,12000,13000,14000,15000,16000,17000,18000,19000,20000,21000,22000,23000,24000,25000,26000,27000,28000,29000,30000
NW_003726702_1,Count,0,0,0,0,0,0,0,0,0,0,10,2,2,12,3,0,0,0,0,0,0,0,0,0,1,7,10,9,8,9,2

	
```

```
		  

		NW_003726706_1, Position,0,100,200,300,400,500,600,700,800,900,1000,1100,1200,1300,1400,1500,1600,1700,1800,1900,2000,2100,2200,2300,2400,2500,2600,2700,2800,2900,3000,3100,3200,3300,3400,3500,3600,3700,3800,3900,4000,4100,4200,4300,4400,4500,4600,4700,4800,4900,5000,5100,5200,5300,5400,5500,5600,5700,5800,5900,6000,6100,6200,6300,6400,6500,6600,6700,6800,6900,7000,7100,7200,7300,7400,7500,7600,7700,7800,7900,8000,8100,8200,8300,8400,8500,8600,8700,8800,8900,9000,9100,9200,9300,9400,9500,9600,9700,9800,9900,10000,10100,10200,10300,10400,10500,10600,10700,10800,10900,11000,11100,11200,11300,11400,11500,11600,11700,11800,11900,12000,12100,12200,12300,12400,12500,12600,12700,12800,12900,13000,13100,13200,13300,13400,13500,13600,13700,13800,13900,14000,14100,14200,14300,14400,14500,14600,14700,14800,14900,15000,15100,15200,15300,15400,15500,15600,15700,15800,15900,16000,16100,16200,16300,16400,16500,16600,16700,16800,16900,17000,17100,17200,17300,17400,17500,17600,17700,17800,17900,18000,18100,18200,18300,18400,18500,18600,18700,18800,18900,19000,19100,19200,19300,19400,19500,19600,19700,19800,19900,20000,20100,20200,20300,20400,20500,20600,20700,20800,20900,21000,21100,21200,21300,21400,21500,21600,21700,21800,21900,22000,22100,22200,22300
NW_003726706_1,Count,0,0,0,0,0,0,0,0,0,0,0,0,0,0,1,2,0,0,0,0,3,2,4,2,2,1,0,0,3,0,0,0,0,0,0,0,1,1,2,4,2,5,1,0,2,0,0,0,0,1,0,0,1,0,1,0,0,0,3,4,0,2,3,2,2,2,1,3,2,0,0,2,2,0,0,2,4,1,2,2,2,0,2,0,0,0,0,4,0,0,1,3,1,2,1,1,0,0,0,2,0,1,1,2,2,1,0,3,0,2,1,1,0,0,2,0,2,2,1,2,0,2,0,1,1,0,0,2,1,1,1,1,2,1,0,0,1,1,1,0,2,1,0,1,0,2,1,0,2,0,0,0,1,1,0,1,0,2,2,0,2,0,2,1,1,3,1,0,0,0,0,0,0,1,1,0,2,0,0,0,2,0,0,0,0,1,1,0,1,0,2,1,4,0,0,0,2,0,1,3,0,1,1,2,0,0,2,2,0,0,0,0,0,0,0,0,0,0,2,1,1,0,0,0

	
```

```
		  

		NW_003726710_1, Position,0,100,200,300,400,500,600,700,800,900,1000,1100,1200,1300,1400,1500,1600,1700,1800,1900,2000,2100,2200,2300,2400,2500,2600,2700,2800,2900,3000,3100,3200,3300,3400,3500,3600,3700,3800,3900,4000,4100,4200,4300,4400,4500,4600,4700,4800,4900,5000,5100,5200,5300,5400,5500,5600,5700,5800,5900,6000,6100,6200,6300,6400,6500,6600,6700,6800,6900,7000,7100,7200,7300,7400,7500,7600,7700,7800,7900,8000,8100,8200,8300,8400,8500,8600,8700,8800,8900,9000,9100,9200,9300,9400,9500,9600,9700,9800,9900,10000,10100,10200,10300,10400,10500,10600,10700,10800,10900,11000,11100,11200,11300,11400,11500,11600,11700,11800,11900,12000,12100,12200,12300,12400,12500,12600,12700,12800,12900,13000,13100,13200,13300,13400,13500,13600,13700,13800,13900,14000,14100,14200,14300,14400,14500,14600,14700,14800,14900,15000,15100,15200,15300,15400,15500,15600,15700,15800,15900,16000,16100,16200,16300,16400,16500,16600,16700,16800,16900,17000,17100,17200,17300,17400,17500,17600,17700,17800,17900,18000,18100,18200,18300,18400,18500,18600,18700,18800,18900,19000,19100,19200,19300,19400,19500,19600,19700,19800,19900,20000,20100,20200,20300,20400,20500,20600,20700,20800,20900,21000,21100,21200,21300,21400,21500,21600,21700,21800,21900,22000,22100,22200
NW_003726710_1,Count,0,0,0,0,0,0,2,1,0,0,0,0,0,2,1,0,0,0,0,0,0,0,0,0,0,0,0,0,0,0,0,0,0,0,0,0,0,0,0,0,0,0,0,0,0,0,0,0,2,1,0,1,1,1,0,0,0,0,0,0,0,0,0,0,0,0,1,3,1,1,1,0,0,0,0,0,1,1,2,0,0,0,0,0,0,0,0,0,0,0,0,0,0,0,0,0,0,0,0,0,0,0,0,0,0,0,0,0,0,0,0,0,0,0,0,0,0,0,0,0,0,0,0,0,0,0,0,0,0,0,0,0,0,0,0,0,0,0,0,0,0,0,0,0,0,0,0,0,0,0,0,0,0,0,0,0,0,0,0,0,0,0,0,0,0,0,0,0,0,0,0,0,0,0,0,0,0,0,0,0,0,0,0,0,0,0,0,0,0,0,0,0,0,0,0,0,0,0,0,0,0,0,0,0,0,0,0,0,0,0,0,0,0,0,0,0,0,0,0,0,0,0,0

	
```

```
		  

		NW_003726713_1, Position,0,100,200,300,400,500,600,700,800,900,1000,1100,1200,1300,1400,1500,1600,1700,1800,1900,2000,2100,2200,2300,2400,2500,2600,2700,2800,2900,3000,3100,3200,3300,3400,3500,3600,3700,3800,3900,4000,4100,4200,4300,4400,4500,4600,4700,4800,4900,5000,5100,5200,5300,5400,5500,5600,5700,5800,5900,6000,6100,6200,6300,6400,6500,6600,6700,6800,6900,7000,7100,7200,7300,7400,7500,7600,7700,7800,7900,8000,8100,8200,8300,8400,8500,8600,8700,8800,8900,9000,9100,9200,9300,9400,9500,9600,9700,9800,9900,10000,10100,10200,10300,10400,10500,10600,10700,10800,10900,11000,11100,11200,11300,11400,11500,11600,11700,11800,11900,12000,12100,12200,12300,12400,12500,12600,12700,12800,12900,13000,13100,13200,13300,13400,13500,13600,13700,13800,13900,14000,14100,14200,14300,14400,14500,14600,14700,14800,14900,15000,15100,15200,15300,15400,15500,15600,15700,15800,15900,16000,16100,16200,16300,16400,16500,16600,16700,16800,16900,17000,17100,17200,17300,17400,17500,17600,17700,17800,17900,18000,18100,18200,18300,18400,18500,18600,18700,18800,18900,19000,19100,19200,19300,19400,19500,19600,19700,19800,19900,20000,20100,20200,20300,20400,20500,20600,20700,20800,20900,21000,21100,21200,21300,21400,21500,21600,21700,21800,21900,22000,22100,22200,22300,22400,22500,22600,22700,22800,22900,23000,23100,23200,23300,23400,23500,23600,23700,23800,23900,24000,24100,24200,24300,24400,24500,24600,24700,24800,24900,25000,25100,25200,25300,25400,25500,25600,25700,25800,25900,26000,26100
NW_003726713_1,Count,0,0,0,0,0,0,0,0,0,0,0,0,0,0,0,0,0,0,0,0,0,0,0,0,0,0,0,0,0,0,0,0,0,0,0,0,0,0,0,0,0,0,0,0,0,0,0,0,0,0,0,0,0,0,0,0,0,0,0,0,0,0,0,0,0,0,0,0,0,0,0,0,0,0,0,0,0,0,0,0,0,0,0,0,0,0,0,0,0,0,0,0,0,0,0,0,0,0,0,0,0,0,0,0,0,0,0,0,0,0,0,0,0,0,0,0,0,0,0,0,0,0,0,0,0,0,0,0,0,0,0,0,0,0,0,0,0,0,0,0,0,0,0,0,0,0,0,0,0,0,0,0,0,0,0,0,0,0,0,0,0,0,0,1,1,0,1,2,1,1,1,0,0,0,1,0,0,0,0,0,0,0,0,0,0,0,0,0,0,0,0,0,0,0,1,0,0,0,0,0,0,0,0,0,0,0,0,0,0,0,0,0,0,0,0,0,0,0,0,2,1,4,3,2,2,0,1,4,1,0,0,0,0,2,0,0,1,3,0,0,0,2,0,0,0,0,3,1,0,1,0,1,0,1,0,1,1,3,2,3,2,1

	
```

```
		  

		NW_003726716_1, Position,0,100,200,300,400,500,600,700,800,900,1000,1100,1200,1300,1400,1500,1600,1700,1800,1900,2000,2100,2200,2300,2400,2500,2600,2700,2800,2900,3000,3100,3200,3300,3400,3500,3600,3700,3800,3900,4000,4100,4200,4300,4400,4500,4600,4700,4800,4900,5000,5100,5200,5300,5400,5500,5600,5700,5800,5900,6000,6100,6200,6300,6400,6500,6600,6700,6800,6900,7000,7100,7200,7300,7400,7500,7600,7700,7800,7900,8000,8100,8200,8300,8400,8500,8600,8700,8800,8900,9000,9100,9200,9300,9400,9500,9600,9700,9800,9900,10000,10100,10200,10300,10400,10500,10600,10700,10800,10900,11000,11100,11200,11300,11400,11500,11600,11700,11800,11900,12000,12100,12200,12300,12400,12500,12600,12700,12800,12900,13000,13100,13200,13300,13400,13500,13600,13700,13800,13900,14000,14100,14200,14300,14400,14500,14600,14700,14800,14900,15000,15100,15200,15300,15400,15500,15600,15700,15800,15900,16000,16100,16200,16300,16400,16500,16600,16700,16800,16900,17000,17100,17200,17300,17400,17500,17600,17700,17800,17900,18000,18100,18200,18300,18400,18500,18600,18700,18800,18900,19000,19100,19200,19300,19400,19500,19600,19700,19800,19900,20000,20100,20200,20300,20400,20500,20600,20700,20800,20900,21000,21100,21200,21300,21400,21500,21600,21700,21800,21900,22000
NW_003726716_1,Count,0,0,2,1,0,0,0,0,0,0,2,0,3,0,3,1,0,1,0,0,0,0,0,0,0,2,0,0,3,3,2,1,1,1,0,1,4,0,0,2,3,3,1,2,1,0,1,2,0,0,2,2,2,2,2,1,1,1,2,0,0,1,1,1,1,2,1,1,2,0,3,0,0,1,3,2,0,0,3,1,1,0,1,0,3,1,1,4,2,1,3,0,0,4,0,0,0,0,0,0,0,0,0,0,0,0,0,2,0,1,2,2,2,0,1,3,0,1,1,1,3,0,0,0,0,0,0,0,0,2,1,0,2,0,0,0,1,2,3,1,0,4,0,3,3,3,1,1,2,0,0,3,1,0,0,0,0,0,0,0,1,1,1,0,1,0,3,0,1,2,0,1,2,2,0,0,0,2,3,2,0,0,0,2,0,4,1,1,0,0,0,0,0,1,1,0,0,0,2,3,1,0,1,1,1,0,1,0,0,0,0,1,3,0,0,0,1,3,0,3,0

	
```

```
		  

		NW_003726719_1, Position,0,100,200,300,400,500,600,700,800,900,1000,1100,1200,1300,1400,1500,1600,1700,1800,1900,2000,2100,2200,2300,2400,2500,2600,2700,2800,2900,3000,3100,3200,3300,3400,3500,3600,3700,3800,3900,4000,4100,4200,4300,4400,4500,4600,4700,4800,4900,5000,5100,5200,5300,5400,5500,5600,5700,5800,5900,6000,6100,6200,6300,6400,6500,6600,6700,6800,6900,7000,7100,7200,7300,7400,7500,7600,7700,7800,7900,8000,8100,8200,8300,8400,8500,8600,8700,8800,8900,9000,9100,9200,9300,9400,9500,9600,9700,9800,9900,10000,10100,10200,10300,10400,10500,10600,10700,10800,10900,11000,11100,11200,11300,11400,11500,11600,11700,11800,11900,12000,12100,12200,12300,12400,12500,12600,12700,12800,12900,13000,13100,13200,13300,13400,13500,13600,13700,13800,13900,14000,14100,14200,14300,14400,14500,14600,14700,14800,14900,15000,15100,15200,15300,15400,15500,15600,15700,15800,15900,16000,16100,16200,16300,16400,16500,16600,16700,16800,16900,17000,17100,17200,17300,17400,17500,17600,17700,17800,17900,18000,18100,18200,18300,18400,18500,18600,18700,18800,18900,19000,19100,19200,19300,19400,19500,19600,19700,19800,19900,20000,20100,20200,20300,20400,20500,20600,20700,20800,20900,21000,21100,21200,21300,21400,21500,21600,21700,21800,21900,22000
NW_003726719_1,Count,0,0,0,0,0,0,0,0,0,0,0,0,0,0,0,0,0,0,0,0,0,0,0,0,0,0,0,0,0,0,0,0,0,0,0,0,0,0,0,0,0,0,0,0,0,0,0,0,0,0,0,0,0,0,0,0,0,0,0,0,0,0,0,0,0,0,0,0,0,0,0,0,0,0,0,0,0,0,0,0,0,0,0,0,0,0,0,0,0,0,0,2,3,0,2,0,0,1,1,0,0,0,0,0,0,0,0,0,0,0,0,0,0,0,0,0,0,0,0,0,0,0,0,0,0,0,0,0,0,0,0,0,0,0,0,0,0,0,0,0,0,0,0,0,0,0,0,0,0,0,0,0,0,0,0,0,0,0,0,0,0,0,0,0,0,0,0,0,0,0,0,0,0,0,0,0,0,0,0,0,0,0,0,0,0,0,0,0,0,0,0,0,0,0,0,0,0,0,0,0,0,0,0,0,0,0,0,0,0,0,0,0,0,0,0,0,0,0,0,0,0

	
```

```
		  

		NW_003726722_1, Position,0,100,200,300,400,500,600,700,800,900,1000,1100,1200,1300,1400,1500,1600,1700,1800,1900,2000,2100,2200,2300,2400,2500,2600,2700,2800,2900,3000,3100,3200,3300,3400,3500,3600,3700,3800,3900,4000,4100,4200,4300,4400,4500,4600,4700,4800,4900,5000,5100,5200,5300,5400,5500,5600,5700,5800,5900,6000,6100,6200,6300,6400,6500,6600,6700,6800,6900,7000,7100,7200,7300,7400,7500,7600,7700,7800,7900,8000,8100,8200,8300,8400,8500,8600,8700,8800,8900,9000,9100,9200,9300,9400,9500,9600,9700,9800,9900,10000,10100,10200,10300,10400,10500,10600,10700,10800,10900,11000,11100,11200,11300,11400,11500,11600,11700,11800,11900,12000,12100,12200,12300,12400,12500,12600,12700,12800,12900,13000,13100,13200,13300,13400,13500,13600,13700,13800,13900,14000,14100,14200,14300,14400,14500,14600,14700,14800,14900,15000,15100,15200,15300,15400,15500,15600,15700,15800,15900,16000,16100,16200,16300,16400,16500,16600,16700,16800,16900,17000,17100,17200,17300,17400,17500,17600,17700,17800,17900,18000,18100,18200,18300,18400,18500,18600,18700,18800,18900,19000,19100,19200,19300,19400,19500,19600,19700,19800,19900,20000,20100,20200,20300,20400,20500,20600,20700,20800,20900,21000,21100,21200,21300,21400,21500,21600,21700,21800,21900
NW_003726722_1,Count,0,0,0,0,0,0,0,0,0,0,0,0,0,0,0,0,0,0,0,0,2,2,1,2,0,1,0,0,0,0,0,0,0,0,0,0,0,0,0,0,0,0,0,0,0,0,0,0,0,0,0,0,0,0,0,0,0,1,0,0,0,0,2,0,1,0,0,0,0,0,0,0,0,0,0,0,0,0,0,0,0,0,0,0,0,0,0,0,0,0,0,0,0,0,0,0,0,0,0,4,0,1,0,0,0,1,1,0,1,0,0,1,2,2,1,0,1,1,1,0,1,0,0,0,0,0,0,0,0,3,1,0,0,0,0,0,0,0,0,0,0,0,0,0,0,3,1,1,1,0,2,1,0,0,1,2,0,2,0,1,2,1,1,0,0,0,0,0,0,0,0,0,0,0,0,0,0,0,0,0,0,0,0,0,0,1,0,0,0,1,0,0,3,0,0,0,1,2,1,1,2,2,1,0,0,0,0,0,0,0,0,0,0,0,0,0,0,2,0,0

	
```

```
		  

		NW_003726729_1, Position,0,100,200,300,400,500,600,700,800,900,1000,1100,1200,1300,1400,1500,1600,1700,1800,1900,2000,2100,2200,2300,2400,2500,2600,2700,2800,2900,3000,3100,3200,3300,3400,3500,3600,3700,3800,3900,4000,4100,4200,4300,4400,4500,4600,4700,4800,4900,5000,5100,5200,5300,5400,5500,5600,5700,5800,5900,6000,6100,6200,6300,6400,6500,6600,6700,6800,6900,7000,7100,7200,7300,7400,7500,7600,7700,7800,7900,8000,8100,8200,8300,8400,8500,8600,8700,8800,8900,9000,9100,9200,9300,9400,9500,9600,9700,9800,9900,10000,10100,10200,10300,10400,10500,10600,10700,10800,10900,11000,11100,11200,11300,11400,11500,11600,11700,11800,11900,12000,12100,12200,12300,12400,12500,12600,12700,12800,12900,13000,13100,13200,13300,13400,13500,13600,13700,13800,13900,14000,14100,14200,14300,14400,14500,14600,14700,14800,14900,15000,15100,15200,15300,15400,15500,15600,15700,15800,15900,16000,16100,16200,16300,16400,16500,16600,16700,16800,16900,17000,17100,17200,17300,17400,17500,17600,17700,17800,17900,18000,18100,18200,18300,18400,18500,18600,18700,18800,18900,19000,19100,19200,19300,19400,19500,19600,19700,19800,19900,20000,20100,20200,20300,20400,20500,20600,20700,20800,20900,21000,21100,21200,21300,21400,21500,21600
NW_003726729_1,Count,0,0,0,0,0,0,0,0,1,2,0,0,0,0,3,0,0,0,0,0,0,0,0,0,0,0,0,0,0,0,0,0,0,0,0,0,2,5,0,0,0,0,0,1,1,0,0,0,0,0,0,2,1,2,2,2,0,0,0,0,0,0,0,0,2,1,2,1,1,1,0,0,0,0,0,3,1,0,0,0,0,0,0,0,0,0,0,0,0,0,0,0,0,0,0,1,0,0,0,0,0,0,0,0,0,0,0,0,0,0,0,0,0,0,0,0,0,0,0,0,2,1,0,0,0,0,2,1,2,2,1,0,0,2,0,0,0,0,2,0,0,0,0,0,0,0,0,3,2,1,1,0,0,0,0,0,0,0,0,0,0,3,0,0,0,0,0,0,0,0,0,0,0,0,0,1,2,0,1,0,0,1,1,0,0,0,0,0,0,0,0,0,0,0,0,1,0,4,0,1,1,0,0,0,0,0,0,0,0,2,0,2,1,1,0,0,0

	
```

```
		  

		NW_003726730_1, Position,0,100,200,300,400,500,600,700,800,900,1000,1100,1200,1300,1400,1500,1600,1700,1800,1900,2000,2100,2200,2300,2400,2500,2600,2700,2800,2900,3000,3100,3200,3300,3400,3500,3600,3700,3800,3900,4000,4100,4200,4300,4400,4500,4600,4700,4800,4900,5000,5100,5200,5300,5400,5500,5600,5700,5800,5900,6000,6100,6200,6300,6400,6500,6600,6700,6800,6900,7000,7100,7200,7300,7400,7500,7600,7700,7800,7900,8000,8100,8200,8300,8400,8500,8600,8700,8800,8900,9000,9100,9200,9300,9400,9500,9600,9700,9800,9900,10000,10100,10200,10300,10400,10500,10600,10700,10800,10900,11000,11100,11200,11300,11400,11500,11600,11700,11800,11900,12000,12100,12200,12300,12400,12500,12600,12700,12800,12900,13000,13100,13200,13300,13400,13500,13600,13700,13800,13900,14000,14100,14200,14300,14400,14500,14600,14700,14800,14900,15000,15100,15200,15300,15400,15500,15600,15700,15800,15900,16000,16100,16200,16300,16400,16500,16600,16700,16800,16900,17000,17100,17200,17300,17400,17500,17600,17700,17800,17900,18000,18100,18200,18300,18400,18500,18600,18700,18800,18900,19000,19100,19200,19300,19400,19500,19600,19700,19800,19900,20000,20100,20200,20300,20400,20500,20600,20700,20800,20900,21000,21100,21200,21300,21400,21500,21600
NW_003726730_1,Count,0,0,0,0,0,0,0,0,1,1,0,0,0,0,0,0,0,0,0,0,0,0,0,0,0,0,0,0,0,0,0,0,0,0,0,0,0,0,0,0,0,0,0,0,0,0,0,0,0,0,3,0,0,0,0,0,0,2,0,0,0,0,0,0,0,0,0,0,1,1,1,0,0,0,0,0,0,0,0,0,0,0,0,0,0,0,0,0,0,0,0,0,0,0,0,0,0,0,0,0,2,0,0,0,0,0,0,0,0,0,0,0,0,0,0,0,0,0,0,0,0,0,0,1,2,1,0,2,0,0,0,0,0,0,0,0,0,0,0,0,0,0,0,0,0,0,0,0,0,0,0,0,0,0,0,0,0,0,0,0,0,0,0,0,0,0,0,0,0,0,0,0,0,0,0,0,0,0,0,0,0,0,0,0,0,0,0,0,0,0,0,0,0,0,0,0,0,0,0,0,0,0,0,0,0,0,0,0,0,0,0,0,0,0,0,0,0

	
```

```
		  

		NW_003726732_1, Position,0,100,200,300,400,500,600,700,800,900,1000,1100,1200,1300,1400,1500,1600,1700,1800,1900,2000,2100,2200,2300,2400,2500,2600,2700,2800,2900,3000,3100,3200,3300,3400,3500,3600,3700,3800,3900,4000,4100,4200,4300,4400,4500,4600,4700,4800,4900,5000,5100,5200,5300,5400,5500,5600,5700,5800,5900,6000,6100,6200,6300,6400,6500,6600,6700,6800,6900,7000,7100,7200,7300,7400,7500,7600,7700,7800,7900,8000,8100,8200,8300,8400,8500,8600,8700,8800,8900,9000,9100,9200,9300,9400,9500,9600,9700,9800,9900,10000,10100,10200,10300,10400,10500,10600,10700,10800,10900,11000,11100,11200,11300,11400,11500,11600,11700,11800,11900,12000,12100,12200,12300,12400,12500,12600,12700,12800,12900,13000,13100,13200,13300,13400,13500,13600,13700,13800,13900,14000,14100,14200,14300,14400,14500,14600,14700,14800,14900,15000,15100,15200,15300,15400,15500,15600,15700,15800,15900,16000,16100,16200,16300,16400,16500,16600,16700,16800,16900,17000,17100,17200,17300,17400,17500,17600,17700,17800,17900,18000,18100,18200,18300,18400,18500,18600,18700,18800,18900,19000,19100,19200,19300,19400,19500,19600,19700,19800,19900,20000,20100,20200,20300,20400,20500,20600,20700,20800,20900,21000,21100,21200,21300,21400,21500
NW_003726732_1,Count,0,0,0,0,0,0,0,0,0,0,0,0,0,0,0,0,0,0,0,0,0,0,0,0,0,0,0,0,0,0,0,0,0,0,0,0,0,0,0,0,0,0,0,0,0,0,0,0,0,0,0,0,0,0,0,0,0,0,0,0,0,0,0,0,0,0,0,0,0,0,0,0,0,0,0,0,0,0,0,0,0,0,0,0,0,0,0,0,0,0,0,0,0,0,0,0,0,0,0,0,0,0,0,0,0,0,0,0,0,0,0,0,0,0,0,0,0,0,0,0,0,0,0,0,0,0,0,0,0,0,0,0,0,0,0,0,0,1,0,0,0,0,0,0,0,0,0,0,0,0,0,0,0,0,0,0,0,0,0,0,0,0,0,0,0,0,0,0,0,0,0,0,0,0,0,0,0,0,0,0,0,0,0,0,0,0,0,0,0,0,0,0,0,0,0,0,0,0,0,0,0,0,0,0,0,0,0,0,0,0,0,0,0,0,0,0

	
```

```
		  

		NW_003726735_1, Position,0,100,200,300,400,500,600,700,800,900,1000,1100,1200,1300,1400,1500,1600,1700,1800,1900,2000,2100,2200,2300,2400,2500,2600,2700,2800,2900,3000,3100,3200,3300,3400,3500,3600,3700,3800,3900,4000,4100,4200,4300,4400,4500,4600,4700,4800,4900,5000,5100,5200,5300,5400,5500,5600,5700,5800,5900,6000,6100,6200,6300,6400,6500,6600,6700,6800,6900,7000,7100,7200,7300,7400,7500,7600,7700,7800,7900,8000,8100,8200,8300,8400,8500,8600,8700,8800,8900,9000,9100,9200,9300,9400,9500,9600,9700,9800,9900,10000,10100,10200,10300,10400,10500,10600,10700,10800,10900,11000,11100,11200,11300,11400,11500,11600,11700,11800,11900,12000,12100,12200,12300,12400,12500,12600,12700,12800,12900,13000,13100,13200,13300,13400,13500,13600,13700,13800,13900,14000,14100,14200,14300,14400,14500,14600,14700,14800,14900,15000,15100,15200,15300,15400,15500,15600,15700,15800,15900,16000,16100,16200,16300,16400,16500,16600,16700,16800,16900,17000,17100,17200,17300,17400,17500,17600,17700,17800,17900,18000,18100,18200,18300,18400,18500,18600,18700,18800,18900,19000,19100,19200,19300,19400,19500,19600,19700,19800,19900,20000,20100,20200,20300,20400,20500,20600,20700,20800,20900,21000,21100,21200,21300,21400,21500,21600,21700,21800,21900,22000,22100,22200,22300,22400,22500,22600,22700,22800,22900,23000,23100,23200,23300,23400,23500,23600,23700,23800,23900,24000,24100,24200,24300,24400,24500,24600,24700,24800,24900,25000,25100,25200,25300,25400,25500,25600,25700,25800,25900,26000,26100,26200,26300,26400,26500,26600,26700,26800,26900,27000,27100,27200,27300,27400,27500,27600,27700,27800,27900,28000,28100,28200
NW_003726735_1,Count,0,0,0,0,0,0,0,0,0,0,0,0,0,0,0,0,0,0,0,0,0,0,0,0,0,0,0,0,0,0,0,0,0,0,0,0,0,0,0,0,0,0,0,0,0,0,0,0,0,0,0,0,0,0,0,0,0,0,0,0,0,0,0,0,0,0,0,0,0,0,0,0,0,0,0,0,0,0,0,0,0,0,0,0,0,0,0,0,0,0,0,0,0,0,0,0,0,0,0,0,0,0,0,0,0,0,0,0,0,0,0,1,1,1,0,2,2,0,0,0,0,0,0,0,0,0,0,0,0,0,0,0,0,0,0,0,0,0,0,0,0,0,0,0,0,0,0,0,0,0,0,0,0,0,0,0,0,0,0,0,0,0,0,0,0,0,0,0,0,0,1,0,0,0,0,0,0,0,0,0,0,0,0,0,0,0,0,0,0,0,0,0,0,0,0,0,0,0,0,0,0,0,0,0,0,0,0,0,0,0,0,0,0,0,0,0,0,0,0,0,0,0,0,0,0,0,0,0,0,0,0,0,0,0,0,0,0,0,0,0,0,0,0,0,0,0,0,0,0,0,0,0,0,0,0,0,0,0,0,0,0,0,0,0,0,0,0,0,0,0,0,0,0,0,0,0,0,0,0,0,0,0,0

	
```

```
		  

		NW_003726739_1, Position,0,1000,2000,3000,4000,5000,6000,7000,8000,9000,10000,11000,12000,13000,14000,15000,16000,17000,18000,19000,20000,21000,22000,23000,24000,25000,26000,27000,28000,29000,30000,31000,32000,33000,34000,35000,36000,37000,38000,39000,40000,41000,42000
NW_003726739_1,Count,0,0,0,0,0,0,0,0,0,0,0,0,0,0,0,0,0,6,0,0,2,0,0,0,0,0,0,0,0,0,0,0,0,0,0,0,0,0,0,0,0,0,0

	
```

```
		  

		NW_003726740_1, Position,0,100,200,300,400,500,600,700,800,900,1000,1100,1200,1300,1400,1500,1600,1700,1800,1900,2000,2100,2200,2300,2400,2500,2600,2700,2800,2900,3000,3100,3200,3300,3400,3500,3600,3700,3800,3900,4000,4100,4200,4300,4400,4500,4600,4700,4800,4900,5000,5100,5200,5300,5400,5500,5600,5700,5800,5900,6000,6100,6200,6300,6400,6500,6600,6700,6800,6900,7000,7100,7200,7300,7400,7500,7600,7700,7800,7900,8000,8100,8200,8300,8400,8500,8600,8700,8800,8900,9000,9100,9200,9300,9400,9500,9600,9700,9800,9900,10000,10100,10200,10300,10400,10500,10600,10700,10800,10900,11000,11100,11200,11300,11400,11500,11600,11700,11800,11900,12000,12100,12200,12300,12400,12500,12600,12700,12800,12900,13000,13100,13200,13300,13400,13500,13600,13700,13800,13900,14000,14100,14200,14300,14400,14500,14600,14700,14800,14900,15000,15100,15200,15300,15400,15500,15600,15700,15800,15900,16000,16100,16200,16300,16400,16500,16600,16700,16800,16900,17000,17100,17200,17300,17400,17500,17600,17700,17800,17900,18000,18100,18200,18300,18400,18500,18600,18700,18800,18900,19000,19100,19200,19300,19400,19500,19600,19700,19800,19900,20000,20100,20200,20300,20400,20500,20600,20700,20800,20900,21000,21100,21200,21300,21400,21500,21600,21700,21800,21900,22000,22100,22200,22300,22400,22500,22600,22700,22800,22900,23000,23100,23200,23300,23400,23500,23600,23700,23800,23900,24000,24100,24200,24300,24400,24500,24600,24700,24800,24900,25000,25100,25200,25300,25400,25500,25600,25700,25800,25900,26000,26100,26200,26300,26400,26500,26600,26700,26800,26900,27000,27100,27200,27300,27400,27500
NW_003726740_1,Count,0,0,0,0,0,1,1,0,1,1,1,0,1,0,2,0,0,1,1,1,1,0,0,0,1,1,1,1,0,0,0,1,0,0,2,0,3,0,1,0,0,0,0,0,0,1,0,0,1,0,1,3,1,1,0,1,0,0,1,0,1,0,0,1,1,0,0,0,1,2,0,1,0,0,0,0,1,0,0,0,1,0,0,1,0,0,1,0,1,1,1,0,1,3,0,0,2,2,2,0,0,0,0,0,0,0,0,0,0,0,0,0,0,0,0,0,0,0,0,0,0,0,0,0,0,0,0,0,0,0,0,0,0,0,0,0,0,0,0,0,0,0,0,0,0,0,0,0,0,0,0,0,0,0,0,0,0,0,0,0,0,0,0,0,0,0,0,0,0,0,0,0,0,0,0,0,0,0,0,0,0,0,0,0,0,0,0,0,0,0,0,0,0,0,0,0,1,0,1,0,1,0,2,1,3,1,0,2,2,0,1,0,0,0,0,0,3,1,0,0,2,0,1,2,0,2,1,0,0,0,0,0,0,0,0,0,0,0,0,1,2,1,1,2,2,1,0,0,1,1,0,0,0,0,2,0,0,0,0,0,0,0,0,0,1,0,0,0,2,0,1,2,1,0,0,0

	
```

```
		  

		NW_003726741_1, Position,0,100,200,300,400,500,600,700,800,900,1000,1100,1200,1300,1400,1500,1600,1700,1800,1900,2000,2100,2200,2300,2400,2500,2600,2700,2800,2900,3000,3100,3200,3300,3400,3500,3600,3700,3800,3900,4000,4100,4200,4300,4400,4500,4600,4700,4800,4900,5000,5100,5200,5300,5400,5500,5600,5700,5800,5900,6000,6100,6200,6300,6400,6500,6600,6700,6800,6900,7000,7100,7200,7300,7400,7500,7600,7700,7800,7900,8000,8100,8200,8300,8400,8500,8600,8700,8800,8900,9000,9100,9200,9300,9400,9500,9600,9700,9800,9900,10000,10100,10200,10300,10400,10500,10600,10700,10800,10900,11000,11100,11200,11300,11400,11500,11600,11700,11800,11900,12000,12100,12200,12300,12400,12500,12600,12700,12800,12900,13000,13100,13200,13300,13400,13500,13600,13700,13800,13900,14000,14100,14200,14300,14400,14500,14600,14700,14800,14900,15000,15100,15200,15300,15400,15500,15600,15700,15800,15900,16000,16100,16200,16300,16400,16500,16600,16700,16800,16900,17000,17100,17200,17300,17400,17500,17600,17700,17800,17900,18000,18100,18200,18300,18400,18500,18600,18700,18800,18900,19000,19100,19200,19300,19400,19500,19600,19700,19800,19900,20000,20100,20200,20300,20400,20500,20600,20700,20800,20900,21000,21100,21200,21300
NW_003726741_1,Count,0,0,0,0,0,0,0,0,0,0,0,0,1,1,0,2,0,0,0,1,1,3,1,1,1,0,0,0,1,0,0,3,0,2,0,1,0,0,0,0,0,2,0,1,0,0,0,0,1,0,1,1,1,0,1,1,0,0,0,3,1,1,2,0,0,0,0,0,1,3,1,0,1,1,0,0,0,2,1,2,1,0,0,1,0,2,1,0,1,0,2,0,1,0,1,1,0,0,0,0,0,0,0,2,1,1,0,0,1,0,0,0,1,2,1,1,1,1,1,0,0,1,0,0,0,0,0,0,0,0,0,0,0,0,0,0,0,0,2,0,1,0,0,0,0,0,0,0,1,2,2,0,0,1,1,0,0,0,0,0,0,3,1,0,0,1,0,1,0,0,0,0,0,1,1,0,1,0,1,1,2,0,0,0,0,1,0,0,0,2,1,0,1,0,0,1,0,0,1,2,0,0,0,0,3,0,0,0,0,0,0,0,0,0

	
```

```
		  

		NW_003726742_1, Position,0,100,200,300,400,500,600,700,800,900,1000,1100,1200,1300,1400,1500,1600,1700,1800,1900,2000,2100,2200,2300,2400,2500,2600,2700,2800,2900,3000,3100,3200,3300,3400,3500,3600,3700,3800,3900,4000,4100,4200,4300,4400,4500,4600,4700,4800,4900,5000,5100,5200,5300,5400,5500,5600,5700,5800,5900,6000,6100,6200,6300,6400,6500,6600,6700,6800,6900,7000,7100,7200,7300,7400,7500,7600,7700,7800,7900,8000,8100,8200,8300,8400,8500,8600,8700,8800,8900,9000,9100,9200,9300,9400,9500,9600,9700,9800,9900,10000,10100,10200,10300,10400,10500,10600,10700,10800,10900,11000,11100,11200,11300,11400,11500,11600,11700,11800,11900,12000,12100,12200,12300,12400,12500,12600,12700,12800,12900,13000,13100,13200,13300,13400,13500,13600,13700,13800,13900,14000,14100,14200,14300,14400,14500,14600,14700,14800,14900,15000,15100,15200,15300,15400,15500,15600,15700,15800,15900,16000,16100,16200,16300,16400,16500,16600,16700,16800,16900,17000,17100,17200,17300,17400,17500,17600,17700,17800,17900,18000,18100,18200,18300,18400,18500,18600,18700,18800,18900,19000,19100,19200,19300,19400,19500,19600,19700,19800,19900,20000,20100,20200,20300,20400,20500,20600,20700,20800,20900,21000,21100,21200
NW_003726742_1,Count,0,0,0,0,0,0,0,0,0,0,0,0,0,0,0,0,0,0,0,0,0,0,0,0,0,0,0,0,0,0,0,0,0,0,0,0,0,0,0,0,0,0,0,0,0,0,0,0,0,0,0,0,0,0,0,0,0,0,0,0,0,0,0,0,0,0,0,0,1,2,1,2,2,2,1,4,0,0,1,1,1,2,2,0,0,0,0,0,0,0,0,0,0,0,0,0,0,0,0,1,2,1,0,0,0,0,0,0,0,0,0,0,0,0,0,0,1,2,2,0,0,0,0,0,0,0,0,0,0,0,0,0,0,0,0,2,0,0,0,1,0,0,0,0,0,0,0,0,0,0,0,0,0,0,0,0,0,0,0,0,0,0,0,0,0,0,0,0,0,0,0,0,0,0,0,0,1,0,1,3,0,0,0,0,0,0,0,0,0,0,0,0,0,0,0,0,0,0,0,0,0,0,0,0,0,0,0,0,0,0,0,0,0

	
```

```
		  

		NW_003726743_1, Position,0,100,200,300,400,500,600,700,800,900,1000,1100,1200,1300,1400,1500,1600,1700,1800,1900,2000,2100,2200,2300,2400,2500,2600,2700,2800,2900,3000,3100,3200,3300,3400,3500,3600,3700,3800,3900,4000,4100,4200,4300,4400,4500,4600,4700,4800,4900,5000,5100,5200,5300,5400,5500,5600,5700,5800,5900,6000,6100,6200,6300,6400,6500,6600,6700,6800,6900,7000,7100,7200,7300,7400,7500,7600,7700,7800,7900,8000,8100,8200,8300,8400,8500,8600,8700,8800,8900,9000,9100,9200,9300,9400,9500,9600,9700,9800,9900,10000,10100,10200,10300,10400,10500,10600,10700,10800,10900,11000,11100,11200,11300,11400,11500,11600,11700,11800,11900,12000,12100,12200,12300,12400,12500,12600,12700,12800,12900,13000,13100,13200,13300,13400,13500,13600,13700,13800,13900,14000,14100,14200,14300,14400,14500,14600,14700,14800,14900,15000,15100,15200,15300,15400,15500,15600,15700,15800,15900,16000,16100,16200,16300,16400,16500,16600,16700,16800,16900,17000,17100,17200,17300,17400,17500,17600,17700,17800,17900,18000,18100,18200,18300,18400,18500,18600,18700,18800,18900,19000,19100,19200,19300,19400,19500,19600,19700,19800,19900,20000,20100,20200,20300,20400,20500,20600,20700,20800,20900,21000,21100,21200,21300,21400,21500,21600,21700,21800,21900,22000,22100,22200,22300,22400,22500,22600,22700,22800,22900,23000
NW_003726743_1,Count,0,0,0,0,0,0,0,0,0,0,0,0,0,0,0,0,0,0,0,0,0,0,0,0,0,0,0,0,0,0,0,0,0,0,0,0,0,0,0,0,0,0,0,0,0,0,0,0,0,0,0,0,0,0,0,0,0,0,0,0,0,0,0,0,0,0,0,0,0,0,0,0,0,1,0,0,0,0,0,0,0,0,0,0,0,0,0,0,0,0,0,0,0,0,0,0,0,0,0,0,0,0,0,0,0,0,0,0,0,0,0,0,0,0,0,0,0,0,0,0,0,0,0,0,0,0,0,0,0,0,0,0,0,0,0,0,0,0,0,0,0,0,0,0,0,0,0,0,0,0,0,0,0,0,0,0,0,0,0,0,0,0,0,0,0,0,0,0,0,0,0,0,0,0,0,0,0,0,0,0,0,0,0,0,0,0,0,0,0,0,0,0,0,0,0,0,0,0,0,0,0,0,0,0,0,0,0,0,0,0,0,0,0,0,0,0,0,0,0,0,0,0,0,0,0,0,0,0,0,0,0

	
```

```
		  

		NW_003726745_1, Position,0,100,200,300,400,500,600,700,800,900,1000,1100,1200,1300,1400,1500,1600,1700,1800,1900,2000,2100,2200,2300,2400,2500,2600,2700,2800,2900,3000,3100,3200,3300,3400,3500,3600,3700,3800,3900,4000,4100,4200,4300,4400,4500,4600,4700,4800,4900,5000,5100,5200,5300,5400,5500,5600,5700,5800,5900,6000,6100,6200,6300,6400,6500,6600,6700,6800,6900,7000,7100,7200,7300,7400,7500,7600,7700,7800,7900,8000,8100,8200,8300,8400,8500,8600,8700,8800,8900,9000,9100,9200,9300,9400,9500,9600,9700,9800,9900,10000,10100,10200,10300,10400,10500,10600,10700,10800,10900,11000,11100,11200,11300,11400,11500,11600,11700,11800,11900,12000,12100,12200,12300,12400,12500,12600,12700,12800,12900,13000,13100,13200,13300,13400,13500,13600,13700,13800,13900,14000,14100,14200,14300,14400,14500,14600,14700,14800,14900,15000,15100,15200,15300,15400,15500,15600,15700,15800,15900,16000,16100,16200,16300,16400,16500,16600,16700,16800,16900,17000,17100,17200,17300,17400,17500,17600,17700,17800,17900,18000,18100,18200,18300,18400,18500,18600,18700,18800,18900,19000,19100,19200,19300,19400,19500,19600,19700,19800,19900,20000,20100,20200,20300,20400,20500,20600,20700,20800,20900,21000,21100,21200,21300,21400,21500,21600,21700,21800,21900,22000,22100,22200,22300,22400,22500,22600,22700,22800,22900,23000,23100,23200,23300,23400,23500,23600,23700,23800,23900,24000,24100,24200,24300,24400,24500,24600,24700,24800,24900
NW_003726745_1,Count,0,3,1,3,1,1,2,0,0,0,2,0,3,0,0,0,0,0,0,0,0,0,0,0,0,0,0,0,0,0,0,0,0,0,0,0,0,0,0,0,0,0,0,0,0,0,0,0,0,0,0,0,0,0,0,0,0,0,0,0,0,0,0,0,0,0,0,0,0,0,0,0,0,0,0,0,0,0,0,0,0,0,0,0,0,0,0,0,0,0,0,0,0,0,0,0,0,0,0,0,0,0,0,0,0,0,0,0,0,0,0,0,0,0,0,0,0,0,0,0,0,0,0,0,0,0,0,0,0,0,0,0,0,0,3,1,0,0,0,0,0,0,0,0,0,0,0,1,4,2,1,2,0,0,0,0,0,0,0,0,0,0,0,0,0,0,0,0,0,0,0,0,0,0,0,0,0,0,0,0,0,0,0,0,0,0,0,0,0,0,0,0,0,0,0,0,0,0,0,0,0,0,0,0,0,0,0,0,0,0,0,0,0,0,0,0,0,0,0,0,0,0,0,0,0,0,0,0,0,0,0,0,0,0,0,0,0,0,0,0,0,0,0,0,0,0,0,0,0,0

	
```

```
		  

		NW_003726747_1, Position,0,100,200,300,400,500,600,700,800,900,1000,1100,1200,1300,1400,1500,1600,1700,1800,1900,2000,2100,2200,2300,2400,2500,2600,2700,2800,2900,3000,3100,3200,3300,3400,3500,3600,3700,3800,3900,4000,4100,4200,4300,4400,4500,4600,4700,4800,4900,5000,5100,5200,5300,5400,5500,5600,5700,5800,5900,6000,6100,6200,6300,6400,6500,6600,6700,6800,6900,7000,7100,7200,7300,7400,7500,7600,7700,7800,7900,8000,8100,8200,8300,8400,8500,8600,8700,8800,8900,9000,9100,9200,9300,9400,9500,9600,9700,9800,9900,10000,10100,10200,10300,10400,10500,10600,10700,10800,10900,11000,11100,11200,11300,11400,11500,11600,11700,11800,11900,12000,12100,12200,12300,12400,12500,12600,12700,12800,12900,13000,13100,13200,13300,13400,13500,13600,13700,13800,13900,14000,14100,14200,14300,14400,14500,14600,14700,14800,14900,15000,15100,15200,15300,15400,15500,15600,15700,15800,15900,16000,16100,16200,16300,16400,16500,16600,16700,16800,16900,17000,17100,17200,17300,17400,17500,17600,17700,17800,17900,18000,18100,18200,18300,18400,18500,18600,18700,18800,18900,19000,19100,19200,19300,19400,19500,19600,19700,19800,19900,20000,20100,20200,20300,20400,20500,20600,20700,20800,20900,21000
NW_003726747_1,Count,0,0,0,0,0,0,0,0,0,0,0,0,0,0,1,0,0,0,0,0,0,0,0,0,0,0,0,0,0,1,1,0,0,0,0,0,0,0,0,0,0,0,0,0,0,0,0,0,0,0,0,0,0,0,0,0,0,1,0,0,2,1,0,0,0,0,1,1,1,0,0,0,0,0,0,0,0,0,0,0,0,0,0,0,0,0,0,0,0,0,0,3,1,0,0,0,1,2,1,0,0,0,0,0,0,0,0,0,0,0,0,0,0,0,0,0,0,0,0,0,0,0,3,2,5,0,2,0,2,2,1,2,0,0,0,0,0,2,2,1,2,2,0,0,0,0,0,2,0,0,1,0,0,0,0,0,0,0,0,0,0,0,0,0,0,0,0,0,0,0,0,0,0,0,0,0,0,0,0,0,0,0,0,0,0,0,0,0,0,0,0,0,0,0,0,0,0,0,0,0,0,0,0,0,0,0,0,0,0,0,0

	
```

```
		  

		NW_003726748_1, Position,0,100,200,300,400,500,600,700,800,900,1000,1100,1200,1300,1400,1500,1600,1700,1800,1900,2000,2100,2200,2300,2400,2500,2600,2700,2800,2900,3000,3100,3200,3300,3400,3500,3600,3700,3800,3900,4000,4100,4200,4300,4400,4500,4600,4700,4800,4900,5000,5100,5200,5300,5400,5500,5600,5700,5800,5900,6000,6100,6200,6300,6400,6500,6600,6700,6800,6900,7000,7100,7200,7300,7400,7500,7600,7700,7800,7900,8000,8100,8200,8300,8400,8500,8600,8700,8800,8900,9000,9100,9200,9300,9400,9500,9600,9700,9800,9900,10000,10100,10200,10300,10400,10500,10600,10700,10800,10900,11000,11100,11200,11300,11400,11500,11600,11700,11800,11900,12000,12100,12200,12300,12400,12500,12600,12700,12800,12900,13000,13100,13200,13300,13400,13500,13600,13700,13800,13900,14000,14100,14200,14300,14400,14500,14600,14700,14800,14900,15000,15100,15200,15300,15400,15500,15600,15700,15800,15900,16000,16100,16200,16300,16400,16500,16600,16700,16800,16900,17000,17100,17200,17300,17400,17500,17600,17700,17800,17900,18000,18100,18200,18300,18400,18500,18600,18700,18800,18900,19000,19100,19200,19300,19400,19500,19600,19700,19800,19900,20000,20100,20200,20300,20400,20500,20600,20700,20800,20900,21000
NW_003726748_1,Count,0,0,0,1,1,1,0,1,0,1,0,0,0,0,0,3,1,0,0,1,3,0,0,2,0,1,3,1,1,2,0,0,1,2,0,2,1,1,2,0,0,0,1,0,1,1,0,0,0,0,0,0,0,0,0,0,0,0,0,0,0,0,0,0,0,0,0,0,0,0,0,0,0,0,0,0,0,0,0,0,0,0,0,0,0,0,0,0,0,0,0,0,0,0,0,0,0,0,0,0,0,0,0,0,0,0,0,0,0,0,0,0,0,0,0,0,0,0,0,0,0,0,0,0,0,0,0,0,0,0,0,0,0,0,0,0,0,0,0,0,0,0,0,0,0,0,0,0,0,1,1,2,0,1,1,0,1,0,2,0,4,0,1,1,1,0,1,1,0,0,0,0,0,0,0,0,0,0,0,0,0,0,0,0,0,0,0,0,0,0,0,0,0,0,0,0,0,0,0,1,1,1,0,0,0,0,1,0,0,0,0

	
```

```
		  

		NW_003726749_1, Position,0,100,200,300,400,500,600,700,800,900,1000,1100,1200,1300,1400,1500,1600,1700,1800,1900,2000,2100,2200,2300,2400,2500,2600,2700,2800,2900,3000,3100,3200,3300,3400,3500,3600,3700,3800,3900,4000,4100,4200,4300,4400,4500,4600,4700,4800,4900,5000,5100,5200,5300,5400,5500,5600,5700,5800,5900,6000,6100,6200,6300,6400,6500,6600,6700,6800,6900,7000,7100,7200,7300,7400,7500,7600,7700,7800,7900,8000,8100,8200,8300,8400,8500,8600,8700,8800,8900,9000,9100,9200,9300,9400,9500,9600,9700,9800,9900,10000,10100,10200,10300,10400,10500,10600,10700,10800,10900,11000,11100,11200,11300,11400,11500,11600,11700,11800,11900,12000,12100,12200,12300,12400,12500,12600,12700,12800,12900,13000,13100,13200,13300,13400,13500,13600,13700,13800,13900,14000,14100,14200,14300,14400,14500,14600,14700,14800,14900,15000,15100,15200,15300,15400,15500,15600,15700,15800,15900,16000,16100,16200,16300,16400,16500,16600,16700,16800,16900,17000,17100,17200,17300,17400,17500,17600,17700,17800,17900,18000,18100,18200,18300,18400,18500,18600,18700,18800,18900,19000,19100,19200,19300,19400,19500,19600,19700,19800,19900,20000,20100,20200,20300,20400,20500,20600,20700,20800,20900,21000,21100,21200,21300,21400,21500,21600,21700,21800,21900,22000
NW_003726749_1,Count,0,1,1,1,4,1,0,2,0,0,0,0,2,1,0,1,1,0,1,0,0,2,2,3,0,0,1,0,0,1,0,3,1,0,0,0,0,0,0,0,0,1,1,1,2,1,1,1,0,1,1,2,0,1,0,2,0,2,3,0,0,0,0,0,1,0,0,0,1,1,0,1,0,0,1,0,0,1,1,0,1,2,1,1,0,0,1,1,2,1,1,1,0,0,2,0,1,0,0,0,0,0,0,0,0,0,0,0,0,0,0,0,0,0,0,0,0,0,0,0,0,0,0,1,1,1,1,1,0,1,2,2,2,2,1,2,1,1,1,0,2,1,0,0,0,0,0,0,3,0,0,0,0,0,1,1,1,2,1,0,0,0,0,0,0,0,1,1,0,0,1,0,1,0,0,0,2,1,0,0,0,2,2,1,0,1,1,2,2,0,1,2,1,1,0,4,1,1,2,2,2,1,1,1,1,2,1,0,2,2,1,1,1,0,3,0,0,1,0,1,0

	
```

```
		  

		NW_003726751_1, Position,0,100,200,300,400,500,600,700,800,900,1000,1100,1200,1300,1400,1500,1600,1700,1800,1900,2000,2100,2200,2300,2400,2500,2600,2700,2800,2900,3000,3100,3200,3300,3400,3500,3600,3700,3800,3900,4000,4100,4200,4300,4400,4500,4600,4700,4800,4900,5000,5100,5200,5300,5400,5500,5600,5700,5800,5900,6000,6100,6200,6300,6400,6500,6600,6700,6800,6900,7000,7100,7200,7300,7400,7500,7600,7700,7800,7900,8000,8100,8200,8300,8400,8500,8600,8700,8800,8900,9000,9100,9200,9300,9400,9500,9600,9700,9800,9900,10000,10100,10200,10300,10400,10500,10600,10700,10800,10900,11000,11100,11200,11300,11400,11500,11600,11700,11800,11900,12000,12100,12200,12300,12400,12500,12600,12700,12800,12900,13000,13100,13200,13300,13400,13500,13600,13700,13800,13900,14000,14100,14200,14300,14400,14500,14600,14700,14800,14900,15000,15100,15200,15300,15400,15500,15600,15700,15800,15900,16000,16100,16200,16300,16400,16500,16600,16700,16800,16900,17000,17100,17200,17300,17400,17500,17600,17700,17800,17900,18000,18100,18200,18300,18400,18500,18600,18700,18800,18900,19000,19100,19200,19300,19400,19500,19600,19700,19800,19900,20000,20100,20200,20300,20400,20500,20600,20700,20800,20900
NW_003726751_1,Count,1,0,2,2,1,1,3,2,1,2,2,0,2,1,1,1,3,0,1,0,1,0,1,0,0,1,0,0,0,0,1,1,2,0,0,0,0,0,0,1,1,0,4,3,0,2,0,1,0,3,1,1,2,3,1,2,1,0,0,2,1,0,1,1,2,1,0,2,3,0,1,1,0,0,1,0,1,2,1,0,1,1,0,4,2,1,2,0,0,0,1,2,2,2,3,0,2,0,2,2,2,0,2,2,0,2,0,0,0,1,0,3,1,2,1,0,0,0,2,2,0,0,1,1,2,2,1,0,2,0,3,1,1,1,2,1,2,2,2,0,1,2,0,2,0,0,1,1,2,0,0,0,2,1,3,0,0,3,1,2,3,0,1,0,0,0,1,1,1,0,0,0,0,0,2,1,0,2,2,0,0,1,1,0,0,1,1,1,3,1,1,2,2,0,1,1,0,0,0,0,0,0,0,0,0,0,0,0,0,0

	
```

```
		  

		NW_003726755_1, Position,0,100,200,300,400,500,600,700,800,900,1000,1100,1200,1300,1400,1500,1600,1700,1800,1900,2000,2100,2200,2300,2400,2500,2600,2700,2800,2900,3000,3100,3200,3300,3400,3500,3600,3700,3800,3900,4000,4100,4200,4300,4400,4500,4600,4700,4800,4900,5000,5100,5200,5300,5400,5500,5600,5700,5800,5900,6000,6100,6200,6300,6400,6500,6600,6700,6800,6900,7000,7100,7200,7300,7400,7500,7600,7700,7800,7900,8000,8100,8200,8300,8400,8500,8600,8700,8800,8900,9000,9100,9200,9300,9400,9500,9600,9700,9800,9900,10000,10100,10200,10300,10400,10500,10600,10700,10800,10900,11000,11100,11200,11300,11400,11500,11600,11700,11800,11900,12000,12100,12200,12300,12400,12500,12600,12700,12800,12900,13000,13100,13200,13300,13400,13500,13600,13700,13800,13900,14000,14100,14200,14300,14400,14500,14600,14700,14800,14900,15000,15100,15200,15300,15400,15500,15600,15700,15800,15900,16000,16100,16200,16300,16400,16500,16600,16700,16800,16900,17000,17100,17200,17300,17400,17500,17600,17700,17800,17900,18000,18100,18200,18300,18400,18500,18600,18700,18800,18900,19000,19100,19200,19300,19400,19500,19600,19700,19800,19900,20000,20100,20200,20300,20400,20500,20600,20700,20800
NW_003726755_1,Count,0,0,0,0,0,0,0,0,0,0,0,0,0,0,0,1,0,0,0,0,0,0,0,0,0,0,0,0,0,0,0,0,0,0,0,0,0,0,0,0,0,0,0,0,0,0,0,0,0,0,0,0,0,0,0,0,0,0,0,0,0,0,0,0,0,0,0,0,0,0,0,0,0,0,0,0,0,0,0,0,0,0,0,0,0,0,0,0,0,0,0,0,0,0,0,0,0,0,0,0,0,0,0,0,0,0,0,0,0,0,0,0,0,0,0,0,0,0,0,0,0,0,0,0,0,0,0,0,0,0,0,0,0,0,0,0,0,0,0,0,0,0,0,0,0,0,0,0,0,0,0,0,0,0,0,0,0,0,0,0,0,0,0,0,0,0,0,0,0,0,0,0,0,0,0,0,0,0,0,0,0,0,0,0,0,0,0,0,0,0,0,0,0,0,0,0,0,0,0,0,0,0,0,0,0,0,0,0,0

	
```

```
		  

		NW_003726758_1, Position,0,100,200,300,400,500,600,700,800,900,1000,1100,1200,1300,1400,1500,1600,1700,1800,1900,2000,2100,2200,2300,2400,2500,2600,2700,2800,2900,3000,3100,3200,3300,3400,3500,3600,3700,3800,3900,4000,4100,4200,4300,4400,4500,4600,4700,4800,4900,5000,5100,5200,5300,5400,5500,5600,5700,5800,5900,6000,6100,6200,6300,6400,6500,6600,6700,6800,6900,7000,7100,7200,7300,7400,7500,7600,7700,7800,7900,8000,8100,8200,8300,8400,8500,8600,8700,8800,8900,9000,9100,9200,9300,9400,9500,9600,9700,9800,9900,10000,10100,10200,10300,10400,10500,10600,10700,10800,10900,11000,11100,11200,11300,11400,11500,11600,11700,11800,11900,12000,12100,12200,12300,12400,12500,12600,12700,12800,12900,13000,13100,13200,13300,13400,13500,13600,13700,13800,13900,14000,14100,14200,14300,14400,14500,14600,14700,14800,14900,15000,15100,15200,15300,15400,15500,15600,15700,15800,15900,16000,16100,16200,16300,16400,16500,16600,16700,16800,16900,17000,17100,17200,17300,17400,17500,17600,17700,17800,17900,18000,18100,18200,18300,18400,18500,18600,18700,18800,18900,19000,19100,19200,19300,19400,19500,19600,19700,19800,19900,20000,20100,20200,20300,20400,20500,20600,20700
NW_003726758_1,Count,0,0,0,0,0,0,0,0,0,0,0,0,0,0,0,0,0,0,0,0,0,0,0,1,1,1,1,0,0,0,0,0,0,0,0,0,0,0,0,0,0,0,0,0,0,0,0,0,0,0,0,0,0,0,0,0,0,0,0,0,0,0,0,0,0,0,0,1,0,0,0,1,1,0,0,0,0,1,0,2,1,3,0,0,0,0,0,0,0,0,0,0,0,0,0,0,0,0,0,0,0,0,0,0,0,0,0,0,0,0,0,0,0,0,0,0,0,0,0,0,0,0,0,0,0,0,0,0,0,0,0,0,2,1,0,0,0,0,0,0,0,0,0,0,0,0,0,0,0,0,0,0,0,0,0,0,0,0,0,0,0,0,0,0,0,0,0,0,0,0,0,0,0,0,0,0,0,0,0,0,0,0,0,0,0,0,0,0,0,0,0,0,0,0,0,0,0,0,0,0,0,0,0,0,0,0,0,0

	
```

```
		  

		NW_003726759_1, Position,0,1000,2000,3000,4000,5000,6000,7000,8000,9000,10000,11000,12000,13000,14000,15000,16000,17000,18000,19000,20000,21000,22000,23000,24000,25000,26000,27000,28000,29000,30000,31000,32000,33000,34000
NW_003726759_1,Count,7,4,0,0,0,0,0,0,0,12,0,0,0,10,4,0,7,6,15,5,0,0,0,0,3,0,0,0,8,9,0,8,12,7,6

	
```

```
		  

		NW_003726764_1, Position,0,100,200,300,400,500,600,700,800,900,1000,1100,1200,1300,1400,1500,1600,1700,1800,1900,2000,2100,2200,2300,2400,2500,2600,2700,2800,2900,3000,3100,3200,3300,3400,3500,3600,3700,3800,3900,4000,4100,4200,4300,4400,4500,4600,4700,4800,4900,5000,5100,5200,5300,5400,5500,5600,5700,5800,5900,6000,6100,6200,6300,6400,6500,6600,6700,6800,6900,7000,7100,7200,7300,7400,7500,7600,7700,7800,7900,8000,8100,8200,8300,8400,8500,8600,8700,8800,8900,9000,9100,9200,9300,9400,9500,9600,9700,9800,9900,10000,10100,10200,10300,10400,10500,10600,10700,10800,10900,11000,11100,11200,11300,11400,11500,11600,11700,11800,11900,12000,12100,12200,12300,12400,12500,12600,12700,12800,12900,13000,13100,13200,13300,13400,13500,13600,13700,13800,13900,14000,14100,14200,14300,14400,14500,14600,14700,14800,14900,15000,15100,15200,15300,15400,15500,15600,15700,15800,15900,16000,16100,16200,16300,16400,16500,16600,16700,16800,16900,17000,17100,17200,17300,17400,17500,17600,17700,17800,17900,18000,18100,18200,18300,18400,18500,18600,18700,18800,18900,19000,19100,19200,19300,19400,19500,19600,19700,19800,19900,20000,20100,20200,20300,20400,20500
NW_003726764_1,Count,0,0,0,0,1,1,0,0,1,3,0,0,0,0,1,4,0,0,0,0,0,1,1,0,0,0,0,0,0,0,0,0,1,0,0,1,3,0,1,3,1,1,2,2,0,1,0,0,3,1,3,0,0,1,2,2,1,0,0,3,1,0,2,2,2,1,2,0,1,0,2,1,1,0,1,2,0,0,2,0,2,0,2,1,1,2,2,1,1,0,0,0,0,0,0,0,0,0,0,0,0,0,0,0,0,0,0,0,0,0,0,0,1,1,2,0,1,1,1,0,0,0,0,0,0,0,0,0,0,0,0,0,0,0,0,0,0,0,0,0,0,0,0,0,0,0,0,0,0,0,0,0,0,0,0,0,0,0,0,0,2,0,0,0,0,0,0,0,1,1,0,0,0,0,0,0,0,0,0,0,0,0,0,0,0,0,0,0,0,0,0,0,0,0,0,1,2,0,2,2,0,0,0,1,1,0

	
```

```
		  

		NW_003726768_1, Position,0,100,200,300,400,500,600,700,800,900,1000,1100,1200,1300,1400,1500,1600,1700,1800,1900,2000,2100,2200,2300,2400,2500,2600,2700,2800,2900,3000,3100,3200,3300,3400,3500,3600,3700,3800,3900,4000,4100,4200,4300,4400,4500,4600,4700,4800,4900,5000,5100,5200,5300,5400,5500,5600,5700,5800,5900,6000,6100,6200,6300,6400,6500,6600,6700,6800,6900,7000,7100,7200,7300,7400,7500,7600,7700,7800,7900,8000,8100,8200,8300,8400,8500,8600,8700,8800,8900,9000,9100,9200,9300,9400,9500,9600,9700,9800,9900,10000,10100,10200,10300,10400,10500,10600,10700,10800,10900,11000,11100,11200,11300,11400,11500,11600,11700,11800,11900,12000,12100,12200,12300,12400,12500,12600,12700,12800,12900,13000,13100,13200,13300,13400,13500,13600,13700,13800,13900,14000,14100,14200,14300,14400,14500,14600,14700,14800,14900,15000,15100,15200,15300,15400,15500,15600,15700,15800,15900,16000,16100,16200,16300,16400,16500,16600,16700,16800,16900,17000,17100,17200,17300,17400,17500,17600,17700,17800,17900,18000,18100,18200,18300,18400,18500,18600,18700,18800,18900,19000,19100,19200,19300,19400,19500,19600,19700,19800,19900,20000,20100,20200,20300,20400
NW_003726768_1,Count,0,0,0,0,0,0,0,0,0,0,0,0,0,0,0,0,0,0,0,0,0,0,0,0,0,0,0,0,0,0,0,0,0,0,0,0,0,0,0,0,0,0,0,0,0,0,0,0,0,0,0,0,0,0,0,0,0,1,2,0,0,0,0,0,0,1,0,0,0,0,0,0,0,0,0,1,0,1,0,0,0,0,0,0,0,0,0,0,0,0,0,0,0,0,0,0,0,0,0,0,0,0,0,0,0,0,0,0,0,0,0,0,0,0,0,0,1,1,0,0,0,1,1,3,0,0,0,0,0,0,0,0,0,0,0,0,0,0,0,0,0,0,0,0,0,0,0,0,0,0,0,0,0,0,0,0,0,1,1,0,0,0,0,0,0,0,0,0,0,0,0,0,0,0,1,0,0,0,0,0,0,0,0,0,0,0,0,0,0,0,0,0,0,0,0,0,0,0,0,0,0,0,1,3,0

	
```

```
		  

		NW_003726769_1, Position,0,100,200,300,400,500,600,700,800,900,1000,1100,1200,1300,1400,1500,1600,1700,1800,1900,2000,2100,2200,2300,2400,2500,2600,2700,2800,2900,3000,3100,3200,3300,3400,3500,3600,3700,3800,3900,4000,4100,4200,4300,4400,4500,4600,4700,4800,4900,5000,5100,5200,5300,5400,5500,5600,5700,5800,5900,6000,6100,6200,6300,6400,6500,6600,6700,6800,6900,7000,7100,7200,7300,7400,7500,7600,7700,7800,7900,8000,8100,8200,8300,8400,8500,8600,8700,8800,8900,9000,9100,9200,9300,9400,9500,9600,9700,9800,9900,10000,10100,10200,10300,10400,10500,10600,10700,10800,10900,11000,11100,11200,11300,11400,11500,11600,11700,11800,11900,12000,12100,12200,12300,12400,12500,12600,12700,12800,12900,13000,13100,13200,13300,13400,13500,13600,13700,13800,13900,14000,14100,14200,14300,14400,14500,14600,14700,14800,14900,15000,15100,15200,15300,15400,15500,15600,15700,15800,15900,16000,16100,16200,16300,16400,16500,16600,16700,16800,16900,17000,17100,17200,17300,17400,17500,17600,17700,17800,17900,18000,18100,18200,18300,18400,18500,18600,18700,18800,18900,19000,19100,19200,19300,19400,19500,19600,19700,19800,19900,20000,20100,20200,20300,20400
NW_003726769_1,Count,0,0,0,0,0,0,0,0,0,0,0,0,0,0,0,0,0,0,0,0,0,0,0,0,0,0,0,0,0,0,0,0,0,0,0,0,0,0,0,0,0,0,0,0,0,0,0,0,0,0,0,0,0,0,0,0,0,0,0,0,0,0,0,0,0,0,0,0,0,0,0,0,0,0,0,0,0,0,0,0,0,0,0,0,0,0,0,0,0,0,0,0,0,0,0,0,0,0,0,0,0,0,0,0,0,0,0,0,0,0,0,0,0,0,0,0,0,0,2,1,2,2,2,2,2,3,0,1,3,1,2,1,0,0,0,0,0,0,0,0,0,0,0,0,0,0,2,1,2,0,4,0,2,1,3,0,1,3,2,0,1,1,0,3,1,1,3,1,2,2,1,0,0,0,0,0,0,0,1,0,0,0,0,0,0,0,0,0,0,0,2,2,2,1,4,5,0,3,3,1,1,3,0,0,0

	
```

```
		  

		NW_003726783_1, Position,0,100,200,300,400,500,600,700,800,900,1000,1100,1200,1300,1400,1500,1600,1700,1800,1900,2000,2100,2200,2300,2400,2500,2600,2700,2800,2900,3000,3100,3200,3300,3400,3500,3600,3700,3800,3900,4000,4100,4200,4300,4400,4500,4600,4700,4800,4900,5000,5100,5200,5300,5400,5500,5600,5700,5800,5900,6000,6100,6200,6300,6400,6500,6600,6700,6800,6900,7000,7100,7200,7300,7400,7500,7600,7700,7800,7900,8000,8100,8200,8300,8400,8500,8600,8700,8800,8900,9000,9100,9200,9300,9400,9500,9600,9700,9800,9900,10000,10100,10200,10300,10400,10500,10600,10700,10800,10900,11000,11100,11200,11300,11400,11500,11600,11700,11800,11900,12000,12100,12200,12300,12400,12500,12600,12700,12800,12900,13000,13100,13200,13300,13400,13500,13600,13700,13800,13900,14000,14100,14200,14300,14400,14500,14600,14700,14800,14900,15000,15100,15200,15300,15400,15500,15600,15700,15800,15900,16000,16100,16200,16300,16400,16500,16600,16700,16800,16900,17000,17100,17200,17300,17400,17500,17600,17700,17800,17900,18000,18100,18200,18300,18400,18500,18600,18700,18800,18900,19000,19100,19200,19300,19400,19500,19600,19700,19800,19900,20000
NW_003726783_1,Count,0,0,0,0,0,0,0,0,0,0,0,0,0,0,0,0,0,0,0,0,0,0,0,0,0,0,0,0,0,0,0,0,0,0,0,0,0,0,0,0,2,2,0,0,1,2,1,0,3,2,1,1,0,1,2,1,1,1,0,0,0,1,2,0,2,0,1,1,1,0,1,0,2,1,0,1,2,1,1,1,2,1,4,1,2,0,0,1,1,1,1,1,0,1,2,2,1,2,0,1,3,0,0,3,0,0,1,1,3,0,0,0,0,0,0,0,1,0,2,0,2,1,2,0,0,0,0,2,0,0,0,0,1,1,0,0,3,0,0,1,0,2,2,2,0,1,0,1,1,0,0,0,0,0,0,0,1,1,1,0,0,2,1,0,0,0,1,0,1,3,0,3,2,1,0,3,0,1,0,0,0,0,0,0,1,0,0,0,0,0,0,0,0,0,0,0,0,0,0,0,0

	
```

```
		  

		NW_003726785_1, Position,0,100,200,300,400,500,600,700,800,900,1000,1100,1200,1300,1400,1500,1600,1700,1800,1900,2000,2100,2200,2300,2400,2500,2600,2700,2800,2900,3000,3100,3200,3300,3400,3500,3600,3700,3800,3900,4000,4100,4200,4300,4400,4500,4600,4700,4800,4900,5000,5100,5200,5300,5400,5500,5600,5700,5800,5900,6000,6100,6200,6300,6400,6500,6600,6700,6800,6900,7000,7100,7200,7300,7400,7500,7600,7700,7800,7900,8000,8100,8200,8300,8400,8500,8600,8700,8800,8900,9000,9100,9200,9300,9400,9500,9600,9700,9800,9900,10000,10100,10200,10300,10400,10500,10600,10700,10800,10900,11000,11100,11200,11300,11400,11500,11600,11700,11800,11900,12000,12100,12200,12300,12400,12500,12600,12700,12800,12900,13000,13100,13200,13300,13400,13500,13600,13700,13800,13900,14000,14100,14200,14300,14400,14500,14600,14700,14800,14900,15000,15100,15200,15300,15400,15500,15600,15700,15800,15900,16000,16100,16200,16300,16400,16500,16600,16700,16800,16900,17000,17100,17200,17300,17400,17500,17600,17700,17800,17900,18000,18100,18200,18300,18400,18500,18600,18700,18800,18900,19000,19100,19200,19300,19400,19500,19600,19700,19800,19900
NW_003726785_1,Count,0,0,0,0,0,0,0,0,0,0,0,0,0,0,0,0,0,0,0,0,0,0,0,0,0,0,0,0,0,0,0,0,0,0,0,0,0,0,0,0,0,0,0,0,0,0,0,0,0,0,0,0,0,0,0,0,0,0,0,0,0,0,0,0,0,0,0,0,0,0,0,0,0,0,0,0,0,0,0,0,0,0,0,0,0,0,0,0,0,0,0,0,0,0,0,0,0,0,0,0,0,0,0,0,0,0,0,0,0,0,0,0,0,0,0,0,0,0,0,0,0,0,0,0,0,0,0,0,0,0,0,0,0,0,0,0,0,0,0,0,0,0,0,0,0,0,0,0,0,0,0,0,0,0,0,0,0,0,0,0,0,0,0,0,0,0,0,0,0,0,0,0,0,0,0,0,0,0,0,0,1,0,0,0,0,0,0,0,0,0,0,0,0,0,0,0,0,0,0,0

	
```

```
		  

		NW_003726786_1, Position,0,100,200,300,400,500,600,700,800,900,1000,1100,1200,1300,1400,1500,1600,1700,1800,1900,2000,2100,2200,2300,2400,2500,2600,2700,2800,2900,3000,3100,3200,3300,3400,3500,3600,3700,3800,3900,4000,4100,4200,4300,4400,4500,4600,4700,4800,4900,5000,5100,5200,5300,5400,5500,5600,5700,5800,5900,6000,6100,6200,6300,6400,6500,6600,6700,6800,6900,7000,7100,7200,7300,7400,7500,7600,7700,7800,7900,8000,8100,8200,8300,8400,8500,8600,8700,8800,8900,9000,9100,9200,9300,9400,9500,9600,9700,9800,9900,10000,10100,10200,10300,10400,10500,10600,10700,10800,10900,11000,11100,11200,11300,11400,11500,11600,11700,11800,11900,12000,12100,12200,12300,12400,12500,12600,12700,12800,12900,13000,13100,13200,13300,13400,13500,13600,13700,13800,13900,14000,14100,14200,14300,14400,14500,14600,14700,14800,14900,15000,15100,15200,15300,15400,15500,15600,15700,15800,15900,16000,16100,16200,16300,16400,16500,16600,16700,16800,16900,17000,17100,17200,17300,17400,17500,17600,17700,17800,17900,18000,18100,18200,18300,18400,18500,18600,18700,18800,18900,19000,19100,19200,19300,19400,19500,19600,19700,19800,19900
NW_003726786_1,Count,0,0,0,0,0,0,0,0,0,0,0,0,0,0,0,0,0,0,0,0,0,0,0,0,0,0,0,0,0,0,0,0,0,0,0,0,0,0,0,0,0,0,0,0,0,0,0,0,0,0,0,0,0,0,0,0,0,0,0,0,0,0,0,0,0,0,0,0,0,0,0,0,0,0,0,0,0,0,0,0,0,0,0,0,0,0,0,0,0,0,0,0,0,0,0,0,0,0,0,0,0,0,0,0,0,0,0,0,0,0,0,0,0,0,0,0,0,0,0,0,0,0,0,0,0,0,0,0,0,0,0,0,0,0,0,0,0,0,0,0,0,0,0,0,0,0,0,0,0,0,0,0,0,0,0,0,0,0,0,0,0,0,0,0,0,0,0,0,0,0,0,0,0,0,0,0,0,0,0,0,0,0,0,0,0,0,0,0,0,0,0,0,0,0,0,0,1,0,0,0

	
```

```
		  

		NW_003726787_1, Position,0,100,200,300,400,500,600,700,800,900,1000,1100,1200,1300,1400,1500,1600,1700,1800,1900,2000,2100,2200,2300,2400,2500,2600,2700,2800,2900,3000,3100,3200,3300,3400,3500,3600,3700,3800,3900,4000,4100,4200,4300,4400,4500,4600,4700,4800,4900,5000,5100,5200,5300,5400,5500,5600,5700,5800,5900,6000,6100,6200,6300,6400,6500,6600,6700,6800,6900,7000,7100,7200,7300,7400,7500,7600,7700,7800,7900,8000,8100,8200,8300,8400,8500,8600,8700,8800,8900,9000,9100,9200,9300,9400,9500,9600,9700,9800,9900,10000,10100,10200,10300,10400,10500,10600,10700,10800,10900,11000,11100,11200,11300,11400,11500,11600,11700,11800,11900,12000,12100,12200,12300,12400,12500,12600,12700,12800,12900,13000,13100,13200,13300,13400,13500,13600,13700,13800,13900,14000,14100,14200,14300,14400,14500,14600,14700,14800,14900,15000,15100,15200,15300,15400,15500,15600,15700,15800,15900,16000,16100,16200,16300,16400,16500,16600,16700,16800,16900,17000,17100,17200,17300,17400,17500,17600,17700,17800,17900,18000,18100,18200,18300,18400,18500,18600,18700,18800,18900,19000,19100,19200,19300,19400,19500,19600,19700,19800,19900
NW_003726787_1,Count,0,0,0,0,0,0,0,0,0,0,0,0,0,0,0,2,0,0,0,0,0,3,0,0,0,0,0,0,0,0,0,0,0,0,0,0,1,1,0,2,0,0,0,0,0,0,0,0,0,0,0,0,1,3,0,3,1,0,0,0,0,0,0,0,0,0,0,0,0,0,0,0,0,0,0,0,0,0,0,0,0,0,0,0,0,0,0,0,0,0,0,0,0,0,0,0,0,0,0,0,0,0,0,0,0,0,0,0,0,0,0,0,0,0,0,0,0,0,0,0,0,0,0,0,0,0,0,0,0,0,0,0,0,0,0,0,0,0,0,0,0,0,0,0,0,0,0,0,0,0,0,2,1,1,3,1,2,0,0,0,1,0,2,4,0,0,2,0,0,0,0,0,1,2,2,1,1,0,0,0,2,0,2,0,0,0,0,0,0,0,0,0,0,0,0,0,0,0,0,0

	
```

```
		  

		NW_003726791_1, Position,0,100,200,300,400,500,600,700,800,900,1000,1100,1200,1300,1400,1500,1600,1700,1800,1900,2000,2100,2200,2300,2400,2500,2600,2700,2800,2900,3000,3100,3200,3300,3400,3500,3600,3700,3800,3900,4000,4100,4200,4300,4400,4500,4600,4700,4800,4900,5000,5100,5200,5300,5400,5500,5600,5700,5800,5900,6000,6100,6200,6300,6400,6500,6600,6700,6800,6900,7000,7100,7200,7300,7400,7500,7600,7700,7800,7900,8000,8100,8200,8300,8400,8500,8600,8700,8800,8900,9000,9100,9200,9300,9400,9500,9600,9700,9800,9900,10000,10100,10200,10300,10400,10500,10600,10700,10800,10900,11000,11100,11200,11300,11400,11500,11600,11700,11800,11900,12000,12100,12200,12300,12400,12500,12600,12700,12800,12900,13000,13100,13200,13300,13400,13500,13600,13700,13800,13900,14000,14100,14200,14300,14400,14500,14600,14700,14800,14900,15000,15100,15200,15300,15400,15500,15600,15700,15800,15900,16000,16100,16200,16300,16400,16500,16600,16700,16800,16900,17000,17100,17200,17300,17400,17500,17600,17700,17800,17900,18000,18100,18200,18300,18400,18500,18600,18700,18800,18900,19000,19100,19200,19300,19400,19500,19600,19700,19800,19900,20000,20100,20200,20300,20400,20500,20600,20700,20800,20900,21000,21100,21200,21300,21400,21500,21600,21700,21800,21900,22000,22100,22200,22300,22400,22500,22600,22700,22800,22900,23000,23100,23200,23300,23400,23500,23600,23700,23800,23900,24000,24100,24200,24300,24400,24500,24600,24700,24800,24900,25000,25100,25200,25300,25400,25500,25600,25700,25800,25900,26000,26100,26200
NW_003726791_1,Count,0,0,1,0,0,2,2,0,2,0,0,1,0,1,0,0,0,0,0,0,0,0,0,0,0,0,0,0,0,0,0,0,0,0,0,0,0,0,0,4,2,0,0,1,0,2,0,1,0,0,0,1,0,0,0,0,0,0,0,0,0,0,0,0,0,0,0,0,0,0,1,0,0,0,0,1,2,0,0,0,0,0,0,0,0,0,0,0,0,0,0,0,0,0,0,0,0,0,0,0,0,0,0,0,0,0,0,0,0,0,0,0,0,0,0,0,0,0,0,0,0,0,0,0,0,0,0,0,0,0,0,0,0,0,0,0,0,0,0,0,0,0,0,0,0,0,0,0,0,0,0,0,0,0,0,0,0,0,0,0,0,0,0,0,0,0,0,0,0,0,0,0,0,0,0,0,0,0,0,0,0,0,0,0,0,0,0,0,0,0,0,0,0,0,0,0,0,0,0,0,0,0,0,0,0,0,0,0,0,0,0,0,0,0,0,0,0,0,0,0,0,0,0,0,0,0,0,0,0,0,0,0,0,0,0,0,0,0,0,0,0,0,0,0,0,0,0,0,0,0,0,0,0,0,0,0,0,0,0,0,0,0,0

	
```

```
		  

		NW_003726793_1, Position,0,100,200,300,400,500,600,700,800,900,1000,1100,1200,1300,1400,1500,1600,1700,1800,1900,2000,2100,2200,2300,2400,2500,2600,2700,2800,2900,3000,3100,3200,3300,3400,3500,3600,3700,3800,3900,4000,4100,4200,4300,4400,4500,4600,4700,4800,4900,5000,5100,5200,5300,5400,5500,5600,5700,5800,5900,6000,6100,6200,6300,6400,6500,6600,6700,6800,6900,7000,7100,7200,7300,7400,7500,7600,7700,7800,7900,8000,8100,8200,8300,8400,8500,8600,8700,8800,8900,9000,9100,9200,9300,9400,9500,9600,9700,9800,9900,10000,10100,10200,10300,10400,10500,10600,10700,10800,10900,11000,11100,11200,11300,11400,11500,11600,11700,11800,11900,12000,12100,12200,12300,12400,12500,12600,12700,12800,12900,13000,13100,13200,13300,13400,13500,13600,13700,13800,13900,14000,14100,14200,14300,14400,14500,14600,14700,14800,14900,15000,15100,15200,15300,15400,15500,15600,15700,15800,15900,16000,16100,16200,16300,16400,16500,16600,16700,16800,16900,17000,17100,17200,17300,17400,17500,17600,17700,17800,17900,18000,18100,18200,18300,18400,18500,18600,18700,18800,18900,19000,19100,19200,19300,19400,19500,19600,19700,19800,19900,20000,20100,20200,20300,20400,20500,20600,20700,20800,20900,21000,21100,21200,21300,21400
NW_003726793_1,Count,0,0,0,0,0,0,0,0,0,0,0,0,0,0,0,0,0,0,0,0,0,0,1,2,0,0,0,0,0,0,0,0,0,0,0,0,0,0,0,0,0,0,0,0,0,0,0,0,0,0,0,1,1,4,2,2,0,0,7,0,0,3,2,1,3,3,1,3,0,1,1,2,1,2,3,2,0,0,0,0,0,0,1,1,1,4,0,2,1,1,1,1,3,1,3,1,3,1,0,0,1,1,1,0,4,1,2,0,1,1,3,1,4,1,1,2,1,2,1,0,0,1,2,2,1,3,0,2,1,3,1,0,3,1,0,1,5,0,0,2,1,0,2,2,0,2,0,0,3,1,1,0,1,1,2,2,3,1,3,2,1,1,3,1,3,2,0,2,0,1,2,3,1,1,1,1,1,1,0,0,0,3,2,2,2,1,1,1,0,0,0,0,0,0,0,0,0,0,0,0,2,1,1,2,2,1,2,3,1,0,0,1,1,0,0

	
```

```
		  

		NW_003726799_1, Position,0,100,200,300,400,500,600,700,800,900,1000,1100,1200,1300,1400,1500,1600,1700,1800,1900,2000,2100,2200,2300,2400,2500,2600,2700,2800,2900,3000,3100,3200,3300,3400,3500,3600,3700,3800,3900,4000,4100,4200,4300,4400,4500,4600,4700,4800,4900,5000,5100,5200,5300,5400,5500,5600,5700,5800,5900,6000,6100,6200,6300,6400,6500,6600,6700,6800,6900,7000,7100,7200,7300,7400,7500,7600,7700,7800,7900,8000,8100,8200,8300,8400,8500,8600,8700,8800,8900,9000,9100,9200,9300,9400,9500,9600,9700,9800,9900,10000,10100,10200,10300,10400,10500,10600,10700,10800,10900,11000,11100,11200,11300,11400,11500,11600,11700,11800,11900,12000,12100,12200,12300,12400,12500,12600,12700,12800,12900,13000,13100,13200,13300,13400,13500,13600,13700,13800,13900,14000,14100,14200,14300,14400,14500,14600,14700,14800,14900,15000,15100,15200,15300,15400,15500,15600,15700,15800,15900,16000,16100,16200,16300,16400,16500,16600,16700,16800,16900,17000,17100,17200,17300,17400,17500,17600,17700,17800,17900,18000,18100,18200,18300,18400,18500,18600,18700,18800,18900,19000,19100,19200,19300,19400,19500,19600,19700,19800
NW_003726799_1,Count,0,0,0,0,0,0,0,0,0,0,0,0,0,0,0,0,0,0,0,0,0,0,0,0,0,0,0,0,0,0,0,0,0,0,0,0,0,0,0,0,0,0,0,0,0,0,0,0,0,0,0,0,0,0,0,0,0,0,0,0,0,0,0,0,0,0,0,0,0,0,0,0,0,0,0,0,0,0,0,0,0,0,0,0,0,0,0,0,0,0,0,0,0,0,0,2,0,0,0,0,0,0,0,0,0,0,0,0,0,0,0,0,0,0,0,0,0,0,0,0,0,0,0,0,0,0,0,0,0,0,0,0,0,0,0,0,0,0,0,0,0,0,0,0,0,0,0,0,0,0,0,0,0,0,0,0,0,0,2,2,0,0,1,0,0,0,0,0,0,0,0,0,0,0,0,0,0,0,0,0,0,0,0,0,0,0,0,0,0,0,0,0,0,0,0,0,0,0,0

	
```

```
		  

		NW_003726806_1, Position,0,100,200,300,400,500,600,700,800,900,1000,1100,1200,1300,1400,1500,1600,1700,1800,1900,2000,2100,2200,2300,2400,2500,2600,2700,2800,2900,3000,3100,3200,3300,3400,3500,3600,3700,3800,3900,4000,4100,4200,4300,4400,4500,4600,4700,4800,4900,5000,5100,5200,5300,5400,5500,5600,5700,5800,5900,6000,6100,6200,6300,6400,6500,6600,6700,6800,6900,7000,7100,7200,7300,7400,7500,7600,7700,7800,7900,8000,8100,8200,8300,8400,8500,8600,8700,8800,8900,9000,9100,9200,9300,9400,9500,9600,9700,9800,9900,10000,10100,10200,10300,10400,10500,10600,10700,10800,10900,11000,11100,11200,11300,11400,11500,11600,11700,11800,11900,12000,12100,12200,12300,12400,12500,12600,12700,12800,12900,13000,13100,13200,13300,13400,13500,13600,13700,13800,13900,14000,14100,14200,14300,14400,14500,14600,14700,14800,14900,15000,15100,15200,15300,15400,15500,15600,15700,15800,15900,16000,16100,16200,16300,16400,16500,16600,16700,16800,16900,17000,17100,17200,17300,17400,17500,17600,17700,17800,17900,18000,18100,18200,18300,18400,18500,18600,18700,18800,18900,19000,19100,19200,19300,19400,19500,19600,19700,19800,19900,20000,20100,20200,20300,20400,20500,20600
NW_003726806_1,Count,0,0,0,0,0,0,0,0,0,0,0,0,0,0,0,0,0,0,0,0,0,0,0,0,0,0,0,0,0,0,0,0,0,0,0,0,0,0,0,0,0,0,0,0,0,0,0,0,0,0,0,0,0,0,0,0,0,0,0,0,0,0,0,0,0,0,0,0,0,0,0,0,0,0,0,0,0,0,0,0,0,0,0,0,0,0,0,0,0,0,0,0,0,0,0,0,0,0,0,0,0,0,0,0,0,0,0,0,0,0,0,0,0,0,0,0,0,0,0,0,0,0,0,0,0,0,0,0,0,0,0,0,0,0,0,0,0,0,0,0,0,0,0,0,0,0,0,0,0,0,0,0,0,0,0,0,0,0,0,0,0,0,0,0,0,0,0,0,0,0,0,0,0,0,0,0,0,0,0,0,0,0,0,0,0,0,0,0,0,0,0,0,0,0,0,0,0,0,1,0,0,0,0,0,0,0,0

	
```

```
[truncated: 364,364 more chars]
