# Supplementary material for: Genome sequence, population history, and pelage genetics of the endangered African wild dog (Lycaon pictus)
Source: BMC Genomics. 2016 Dec 9;17:1013. doi: 10.1186/s12864-016-3368-9 (PMC5148847; doi:10.1186/s12864-016-3368-9)
Supplement: Additional file 2: — Genic effects of SnpEff-annotated autosomal and X-chromosomal variants for the South African Lycaon individual. (HTML 1535 kb) [file 12864_2016_3368_MOESM2_ESM.html]

### SnpEff: Variant analysis

|  |
| --- |
| **Contents** Summary   Variant rate by chromosome  Variants by type   Number of variants by impact    Number of variants by functional class    Number of variants by effect   Quality histogram  InDel length histogram  Base variant table  Transition vs transversions (ts/tv)   Allele frequency    Allele Count    Codon change table    Amino acid change table    Chromosome variants plots    Details by gene |


---


**Summary**

|  |  |
| --- | --- |
| **Genome** | canfam3\_nuclear |
| **Date** | 2016-04-13 01:06 |
| **SnpEff version** | ``` SnpEff 4.1l (build 2015-10-03), by Pablo Cingolani ``` |
| **Command line arguments** | ``` SnpEff  canfam3_nuclear Ananku_nuclear_concat.q20.vcf ``` |
| **Warnings** | 423,067 |
| **Errors** | 0 |
| **Number of lines (input file)** | 15,641,019 |
| **Number of variants (before filter)** | 15,765,115 |
| **Number of not variants  (i.e. reference equals alternative)** | 0 |
| **Number of variants processed   (i.e. after filter and non-variants)** | 15,765,115 |
| **Number of known variants  (i.e. non-empty ID)** | 0 ( 0% ) |
| **Number of multi-allelic VCF entries  (i.e. more than two alleles)** | 54,451 |
| **Number of effects** | 36,362,161 |
| **Genome total length** | 2,410,960,148 |
| **Genome effective length** | 2,363,967,083 |
| **Variant rate** | 1 variant every 149 bases |


---


 **Variants rate details** 

| Chromosome | Length | Variants | Variants rate |
| --- | --- | --- | --- |
| NC\_006583\_3 | 122,678,785 | 842,619 | 145 |
| NC\_006584\_3 | 85,426,708 | 568,671 | 150 |
| NC\_006585\_3 | 91,889,043 | 639,764 | 143 |
| NC\_006586\_3 | 88,276,631 | 610,476 | 144 |
| NC\_006587\_3 | 88,915,250 | 623,929 | 142 |
| NC\_006588\_3 | 77,573,801 | 534,884 | 145 |
| NC\_006589\_3 | 80,974,532 | 543,692 | 148 |
| NC\_006590\_3 | 74,330,416 | 497,189 | 149 |
| NC\_006591\_3 | 61,074,082 | 466,551 | 130 |
| NC\_006592\_3 | 69,331,447 | 481,815 | 143 |
| NC\_006593\_3 | 74,389,097 | 482,089 | 154 |
| NC\_006594\_3 | 72,498,081 | 484,665 | 149 |
| NC\_006595\_3 | 63,241,923 | 450,181 | 140 |
| NC\_006596\_3 | 60,966,679 | 406,864 | 149 |
| NC\_006597\_3 | 64,190,966 | 440,384 | 145 |
| NC\_006598\_3 | 59,632,846 | 414,054 | 144 |
| NC\_006599\_3 | 64,289,059 | 443,759 | 144 |
| NC\_006600\_3 | 55,844,845 | 394,747 | 141 |
| NC\_006601\_3 | 53,741,614 | 378,397 | 142 |
| NC\_006602\_3 | 58,134,056 | 392,169 | 148 |
| NC\_006603\_3 | 50,858,623 | 354,864 | 143 |
| NC\_006604\_3 | 61,439,934 | 424,363 | 144 |
| NC\_006605\_3 | 52,294,480 | 353,702 | 147 |
| NC\_006606\_3 | 47,698,779 | 330,980 | 144 |
| NC\_006607\_3 | 51,628,933 | 358,155 | 144 |
| NC\_006608\_3 | 38,964,690 | 265,237 | 146 |
| NC\_006609\_3 | 45,876,710 | 306,305 | 149 |
| NC\_006610\_3 | 41,182,112 | 289,740 | 142 |
| NC\_006611\_3 | 41,845,238 | 290,507 | 144 |
| NC\_006612\_3 | 40,214,260 | 265,422 | 151 |
| NC\_006613\_3 | 39,895,921 | 278,875 | 143 |
| NC\_006614\_3 | 38,810,281 | 262,378 | 147 |
| NC\_006615\_3 | 31,377,067 | 218,963 | 143 |
| NC\_006616\_3 | 42,124,431 | 304,225 | 138 |
| NC\_006617\_3 | 26,524,999 | 192,442 | 137 |
| NC\_006618\_3 | 30,810,995 | 211,146 | 145 |
| NC\_006619\_3 | 30,902,991 | 223,945 | 137 |
| NC\_006620\_3 | 23,914,537 | 175,884 | 135 |
| NC\_006621\_3 | 123,869,142 | 509,227 | 243 |
| NW\_003726127\_1 | 2,660,953 | 160 | 16,630 |
| NW\_003726129\_1 | 1,415,205 | 6 | 235,867 |
| NW\_003726130\_1 | 1,067,467 | 27 | 39,535 |
| NW\_003726132\_1 | 881,102 | 27 | 32,633 |
| NW\_003726133\_1 | 822,601 | 6,043 | 136 |
| NW\_003726135\_1 | 745,551 | 193 | 3,862 |
| NW\_003726136\_1 | 602,611 | 627 | 961 |
| NW\_003726137\_1 | 573,630 | 4,730 | 121 |
| NW\_003726139\_1 | 557,017 | 94 | 5,925 |
| NW\_003726140\_1 | 510,604 | 3 | 170,201 |
| NW\_003726141\_1 | 497,093 | 114 | 4,360 |
| NW\_003726142\_1 | 492,630 | 63 | 7,819 |
| NW\_003726143\_1 | 577,062 | 978 | 590 |
| NW\_003726144\_1 | 561,270 | 167 | 3,360 |
| NW\_003726145\_1 | 407,330 | 2 | 203,665 |
| NW\_003726147\_1 | 508,668 | 30 | 16,955 |
| NW\_003726148\_1 | 540,093 | 53 | 10,190 |
| NW\_003726149\_1 | 448,434 | 1,701 | 263 |
| NW\_003726150\_1 | 377,800 | 12 | 31,483 |
| NW\_003726151\_1 | 384,880 | 73 | 5,272 |
| NW\_003726153\_1 | 399,987 | 75 | 5,333 |
| NW\_003726156\_1 | 365,470 | 164 | 2,228 |
| NW\_003726159\_1 | 310,270 | 21 | 14,774 |
| NW\_003726160\_1 | 433,650 | 4 | 108,412 |
| NW\_003726162\_1 | 281,659 | 4 | 70,414 |
| NW\_003726163\_1 | 320,442 | 4 | 80,110 |
| NW\_003726167\_1 | 252,720 | 113 | 2,236 |
| NW\_003726168\_1 | 254,975 | 261 | 976 |
| NW\_003726174\_1 | 386,314 | 1,423 | 271 |
| NW\_003726177\_1 | 216,332 | 24 | 9,013 |
| NW\_003726180\_1 | 200,932 | 101 | 1,989 |
| NW\_003726183\_1 | 192,561 | 236 | 815 |
| NW\_003726184\_1 | 210,134 | 5 | 42,026 |
| NW\_003726185\_1 | 197,844 | 21 | 9,421 |
| NW\_003726186\_1 | 196,813 | 89 | 2,211 |
| NW\_003726188\_1 | 210,247 | 26 | 8,086 |
| NW\_003726189\_1 | 177,735 | 10 | 17,773 |
| NW\_003726191\_1 | 166,866 | 44 | 3,792 |
| NW\_003726193\_1 | 157,598 | 67 | 2,352 |
| NW\_003726194\_1 | 143,980 | 1,317 | 109 |
| NW\_003726195\_1 | 141,074 | 725 | 194 |
| NW\_003726197\_1 | 142,260 | 170 | 836 |
| NW\_003726199\_1 | 142,296 | 13 | 10,945 |
| NW\_003726200\_1 | 133,119 | 102 | 1,305 |
| NW\_003726201\_1 | 129,748 | 169 | 767 |
| NW\_003726203\_1 | 127,429 | 630 | 202 |
| NW\_003726205\_1 | 131,872 | 547 | 241 |
| NW\_003726206\_1 | 133,432 | 263 | 507 |
| NW\_003726210\_1 | 115,952 | 605 | 191 |
| NW\_003726213\_1 | 117,332 | 1 | 117,332 |
| NW\_003726214\_1 | 111,759 | 892 | 125 |
| NW\_003726216\_1 | 112,014 | 11 | 10,183 |
| NW\_003726222\_1 | 118,473 | 500 | 236 |
| NW\_003726224\_1 | 109,585 | 10 | 10,958 |
| NW\_003726225\_1 | 124,049 | 65 | 1,908 |
| NW\_003726227\_1 | 101,741 | 619 | 164 |
| NW\_003726230\_1 | 102,992 | 331 | 311 |
| NW\_003726231\_1 | 95,509 | 106 | 901 |
| NW\_003726236\_1 | 85,476 | 616 | 138 |
| NW\_003726239\_1 | 82,459 | 318 | 259 |
| NW\_003726241\_1 | 82,380 | 39 | 2,112 |
| NW\_003726242\_1 | 102,198 | 8 | 12,774 |
| NW\_003726244\_1 | 87,041 | 2 | 43,520 |
| NW\_003726246\_1 | 230,124 | 314 | 732 |
| NW\_003726248\_1 | 256,406 | 419 | 611 |
| NW\_003726251\_1 | 77,100 | 3 | 25,700 |
| NW\_003726252\_1 | 77,130 | 68 | 1,134 |
| NW\_003726253\_1 | 79,078 | 70 | 1,129 |
| NW\_003726254\_1 | 90,550 | 7 | 12,935 |
| NW\_003726256\_1 | 89,070 | 2 | 44,535 |
| NW\_003726257\_1 | 97,802 | 269 | 363 |
| NW\_003726258\_1 | 79,712 | 746 | 106 |
| NW\_003726260\_1 | 73,720 | 14 | 5,265 |
| NW\_003726262\_1 | 73,171 | 24 | 3,048 |
| NW\_003726263\_1 | 104,732 | 6 | 17,455 |
| NW\_003726264\_1 | 78,935 | 736 | 107 |
| NW\_003726268\_1 | 68,272 | 626 | 109 |
| NW\_003726273\_1 | 67,496 | 470 | 143 |
| NW\_003726275\_1 | 67,057 | 31 | 2,163 |
| NW\_003726280\_1 | 65,696 | 40 | 1,642 |
| NW\_003726285\_1 | 70,351 | 312 | 225 |
| NW\_003726287\_1 | 69,126 | 20 | 3,456 |
| NW\_003726289\_1 | 64,123 | 419 | 153 |
| NW\_003726290\_1 | 61,944 | 19 | 3,260 |
| NW\_003726292\_1 | 61,779 | 360 | 171 |
| NW\_003726293\_1 | 90,840 | 4 | 22,710 |
| NW\_003726294\_1 | 61,194 | 1 | 61,194 |
| NW\_003726295\_1 | 63,245 | 6 | 10,540 |
| NW\_003726297\_1 | 88,336 | 16 | 5,521 |
| NW\_003726300\_1 | 70,666 | 7 | 10,095 |
| NW\_003726301\_1 | 71,007 | 10 | 7,100 |
| NW\_003726304\_1 | 66,981 | 78 | 858 |
| NW\_003726305\_1 | 62,805 | 54 | 1,163 |
| NW\_003726306\_1 | 68,857 | 11 | 6,259 |
| NW\_003726308\_1 | 84,024 | 88 | 954 |
| NW\_003726311\_1 | 56,534 | 3 | 18,844 |
| NW\_003726312\_1 | 59,023 | 10 | 5,902 |
| NW\_003726315\_1 | 54,307 | 2 | 27,153 |
| NW\_003726318\_1 | 57,907 | 53 | 1,092 |
| NW\_003726321\_1 | 54,660 | 1 | 54,660 |
| NW\_003726329\_1 | 50,869 | 15 | 3,391 |
| NW\_003726330\_1 | 53,506 | 6 | 8,917 |
| NW\_003726333\_1 | 63,256 | 2 | 31,628 |
| NW\_003726334\_1 | 59,752 | 151 | 395 |
| NW\_003726336\_1 | 49,027 | 291 | 168 |
| NW\_003726338\_1 | 57,792 | 2 | 28,896 |
| NW\_003726342\_1 | 75,626 | 1 | 75,626 |
| NW\_003726343\_1 | 48,436 | 107 | 452 |
| NW\_003726344\_1 | 47,485 | 332 | 143 |
| NW\_003726348\_1 | 53,681 | 143 | 375 |
| NW\_003726349\_1 | 73,441 | 29 | 2,532 |
| NW\_003726350\_1 | 57,620 | 3 | 19,206 |
| NW\_003726351\_1 | 70,154 | 2 | 35,077 |
| NW\_003726352\_1 | 67,100 | 2 | 33,550 |
| NW\_003726353\_1 | 48,085 | 51 | 942 |
| NW\_003726356\_1 | 58,859 | 197 | 298 |
| NW\_003726357\_1 | 45,065 | 10 | 4,506 |
| NW\_003726358\_1 | 46,098 | 10 | 4,609 |
| NW\_003726361\_1 | 45,518 | 2 | 22,759 |
| NW\_003726362\_1 | 47,960 | 2 | 23,980 |
| NW\_003726363\_1 | 44,571 | 1 | 44,571 |
| NW\_003726364\_1 | 84,617 | 3 | 28,205 |
| NW\_003726365\_1 | 51,679 | 36 | 1,435 |
| NW\_003726367\_1 | 50,939 | 7 | 7,277 |
| NW\_003726369\_1 | 44,216 | 6 | 7,369 |
| NW\_003726370\_1 | 43,704 | 171 | 255 |
| NW\_003726378\_1 | 42,461 | 4 | 10,615 |
| NW\_003726381\_1 | 69,503 | 12 | 5,791 |
| NW\_003726386\_1 | 43,681 | 211 | 207 |
| NW\_003726387\_1 | 57,311 | 1 | 57,311 |
| NW\_003726392\_1 | 40,931 | 187 | 218 |
| NW\_003726394\_1 | 41,006 | 1 | 41,006 |
| NW\_003726396\_1 | 46,509 | 14 | 3,322 |
| NW\_003726399\_1 | 40,818 | 6 | 6,803 |
| NW\_003726401\_1 | 46,980 | 6 | 7,830 |
| NW\_003726405\_1 | 39,345 | 228 | 172 |
| NW\_003726407\_1 | 47,441 | 43 | 1,103 |
| NW\_003726410\_1 | 64,983 | 36 | 1,805 |
| NW\_003726416\_1 | 41,432 | 20 | 2,071 |
| NW\_003726419\_1 | 59,547 | 1 | 59,547 |
| NW\_003726422\_1 | 52,308 | 5 | 10,461 |
| NW\_003726424\_1 | 69,036 | 1 | 69,036 |
| NW\_003726425\_1 | 44,261 | 9 | 4,917 |
| NW\_003726427\_1 | 36,777 | 39 | 943 |
| NW\_003726435\_1 | 37,117 | 161 | 230 |
| NW\_003726437\_1 | 41,256 | 146 | 282 |
| NW\_003726438\_1 | 45,145 | 8 | 5,643 |
| NW\_003726439\_1 | 40,256 | 2 | 20,128 |
| NW\_003726442\_1 | 35,523 | 240 | 148 |
| NW\_003726445\_1 | 67,206 | 4 | 16,801 |
| NW\_003726455\_1 | 34,700 | 133 | 260 |
| NW\_003726457\_1 | 37,165 | 19 | 1,956 |
| NW\_003726458\_1 | 59,196 | 2 | 29,598 |
| NW\_003726460\_1 | 35,730 | 25 | 1,429 |
| NW\_003726461\_1 | 41,582 | 56 | 742 |
| NW\_003726462\_1 | 33,876 | 58 | 584 |
| NW\_003726465\_1 | 33,186 | 11 | 3,016 |
| NW\_003726466\_1 | 34,753 | 26 | 1,336 |
| NW\_003726477\_1 | 42,604 | 2 | 21,302 |
| NW\_003726478\_1 | 31,902 | 30 | 1,063 |
| NW\_003726480\_1 | 51,290 | 3 | 17,096 |
| NW\_003726481\_1 | 31,497 | 276 | 114 |
| NW\_003726482\_1 | 32,248 | 7 | 4,606 |
| NW\_003726485\_1 | 52,768 | 3 | 17,589 |
| NW\_003726487\_1 | 31,015 | 143 | 216 |
| NW\_003726488\_1 | 30,934 | 164 | 188 |
| NW\_003726489\_1 | 45,980 | 4 | 11,495 |
| NW\_003726491\_1 | 39,405 | 4 | 9,851 |
| NW\_003726492\_1 | 39,951 | 2 | 19,975 |
| NW\_003726497\_1 | 30,556 | 1 | 30,556 |
| NW\_003726498\_1 | 30,501 | 39 | 782 |
| NW\_003726501\_1 | 31,768 | 108 | 294 |
| NW\_003726505\_1 | 30,166 | 224 | 134 |
| NW\_003726511\_1 | 29,914 | 3 | 9,971 |
| NW\_003726512\_1 | 38,238 | 3 | 12,746 |
| NW\_003726513\_1 | 41,393 | 1 | 41,393 |
| NW\_003726519\_1 | 29,710 | 24 | 1,237 |
| NW\_003726520\_1 | 29,345 | 142 | 206 |
| NW\_003726523\_1 | 29,738 | 53 | 561 |
| NW\_003726526\_1 | 30,345 | 248 | 122 |
| NW\_003726528\_1 | 29,319 | 2 | 14,659 |
| NW\_003726529\_1 | 28,914 | 36 | 803 |
| NW\_003726530\_1 | 32,931 | 231 | 142 |
| NW\_003726532\_1 | 28,934 | 73 | 396 |
| NW\_003726534\_1 | 28,793 | 83 | 346 |
| NW\_003726540\_1 | 29,108 | 2 | 14,554 |
| NW\_003726545\_1 | 51,377 | 36 | 1,427 |
| NW\_003726549\_1 | 28,200 | 166 | 169 |
| NW\_003726551\_1 | 38,154 | 2 | 19,077 |
| NW\_003726552\_1 | 38,510 | 4 | 9,627 |
| NW\_003726553\_1 | 37,277 | 3 | 12,425 |
| NW\_003726555\_1 | 27,996 | 75 | 373 |
| NW\_003726556\_1 | 38,339 | 3 | 12,779 |
| NW\_003726562\_1 | 27,833 | 29 | 959 |
| NW\_003726563\_1 | 41,669 | 21 | 1,984 |
| NW\_003726567\_1 | 27,463 | 176 | 156 |
| NW\_003726568\_1 | 27,432 | 7 | 3,918 |
| NW\_003726569\_1 | 28,005 | 11 | 2,545 |
| NW\_003726570\_1 | 27,325 | 9 | 3,036 |
| NW\_003726575\_1 | 32,600 | 5 | 6,520 |
| NW\_003726584\_1 | 26,802 | 2 | 13,401 |
| NW\_003726588\_1 | 29,594 | 12 | 2,466 |
| NW\_003726589\_1 | 28,772 | 220 | 130 |
| NW\_003726592\_1 | 26,523 | 96 | 276 |
| NW\_003726593\_1 | 44,314 | 14 | 3,165 |
| NW\_003726594\_1 | 33,462 | 3 | 11,154 |
| NW\_003726597\_1 | 26,369 | 4 | 6,592 |
| NW\_003726599\_1 | 27,345 | 134 | 204 |
| NW\_003726600\_1 | 36,341 | 42 | 865 |
| NW\_003726602\_1 | 26,155 | 149 | 175 |
| NW\_003726605\_1 | 41,410 | 10 | 4,141 |
| NW\_003726610\_1 | 28,880 | 4 | 7,220 |
| NW\_003726611\_1 | 26,077 | 8 | 3,259 |
| NW\_003726613\_1 | 25,686 | 115 | 223 |
| NW\_003726614\_1 | 45,208 | 7 | 6,458 |
| NW\_003726619\_1 | 25,554 | 1 | 25,554 |
| NW\_003726620\_1 | 37,928 | 2 | 18,964 |
| NW\_003726630\_1 | 25,556 | 11 | 2,323 |
| NW\_003726632\_1 | 24,821 | 83 | 299 |
| NW\_003726636\_1 | 24,666 | 169 | 145 |
| NW\_003726637\_1 | 24,856 | 57 | 436 |
| NW\_003726638\_1 | 33,600 | 3 | 11,200 |
| NW\_003726643\_1 | 33,135 | 2 | 16,567 |
| NW\_003726644\_1 | 29,863 | 1 | 29,863 |
| NW\_003726646\_1 | 24,163 | 31 | 779 |
| NW\_003726647\_1 | 40,390 | 55 | 734 |
| NW\_003726649\_1 | 36,033 | 4 | 9,008 |
| NW\_003726650\_1 | 23,898 | 13 | 1,838 |
| NW\_003726651\_1 | 29,441 | 1 | 29,441 |
| NW\_003726654\_1 | 36,336 | 7 | 5,190 |
| NW\_003726663\_1 | 23,542 | 72 | 326 |
| NW\_003726664\_1 | 26,716 | 99 | 269 |
| NW\_003726665\_1 | 23,440 | 50 | 468 |
| NW\_003726666\_1 | 24,967 | 121 | 206 |
| NW\_003726668\_1 | 23,398 | 9 | 2,599 |
| NW\_003726674\_1 | 35,965 | 3 | 11,988 |
| NW\_003726676\_1 | 37,016 | 2 | 18,508 |
| NW\_003726677\_1 | 36,383 | 46 | 790 |
| NW\_003726681\_1 | 23,924 | 154 | 155 |
| NW\_003726682\_1 | 23,069 | 77 | 299 |
| NW\_003726687\_1 | 37,035 | 5 | 7,407 |
| NW\_003726691\_1 | 22,972 | 81 | 283 |
| NW\_003726700\_1 | 23,951 | 2 | 11,975 |
| NW\_003726702\_1 | 30,594 | 87 | 351 |
| NW\_003726706\_1 | 22,331 | 156 | 143 |
| NW\_003726710\_1 | 22,218 | 19 | 1,169 |
| NW\_003726713\_1 | 26,196 | 39 | 671 |
| NW\_003726716\_1 | 22,078 | 185 | 119 |
| NW\_003726719\_1 | 22,057 | 18 | 1,225 |
| NW\_003726722\_1 | 21,926 | 93 | 235 |
| NW\_003726729\_1 | 21,667 | 110 | 196 |
| NW\_003726730\_1 | 21,638 | 8 | 2,704 |
| NW\_003726735\_1 | 28,254 | 9 | 3,139 |
| NW\_003726739\_1 | 42,665 | 6 | 7,110 |
| NW\_003726740\_1 | 27,551 | 106 | 259 |
| NW\_003726741\_1 | 21,300 | 117 | 182 |
| NW\_003726742\_1 | 21,212 | 33 | 642 |
| NW\_003726743\_1 | 23,068 | 2 | 11,534 |
| NW\_003726745\_1 | 24,946 | 27 | 923 |
| NW\_003726747\_1 | 21,029 | 24 | 876 |
| NW\_003726748\_1 | 21,019 | 38 | 553 |
| NW\_003726749\_1 | 22,065 | 156 | 141 |
| NW\_003726751\_1 | 20,926 | 195 | 107 |
| NW\_003726755\_1 | 20,819 | 6 | 3,469 |
| NW\_003726758\_1 | 20,706 | 18 | 1,150 |
| NW\_003726759\_1 | 34,690 | 97 | 357 |
| NW\_003726760\_1 | 21,015 | 2 | 10,507 |
| NW\_003726764\_1 | 20,596 | 86 | 239 |
| NW\_003726768\_1 | 20,472 | 16 | 1,279 |
| NW\_003726769\_1 | 20,448 | 53 | 385 |
| NW\_003726780\_1 | 29,620 | 2 | 14,810 |
| NW\_003726783\_1 | 20,017 | 137 | 146 |
| NW\_003726786\_1 | 19,955 | 4 | 4,988 |
| NW\_003726787\_1 | 19,951 | 42 | 475 |
| NW\_003726791\_1 | 26,207 | 17 | 1,541 |
| NW\_003726793\_1 | 21,446 | 163 | 131 |
| NW\_003726799\_1 | 19,813 | 6 | 3,302 |
| NW\_003726800\_1 | 22,216 | 1 | 22,216 |
| NW\_003726807\_1 | 20,650 | 2 | 10,325 |
| NW\_003726810\_1 | 19,874 | 35 | 567 |
| NW\_003726813\_1 | 19,477 | 29 | 671 |
| NW\_003726817\_1 | 33,123 | 1 | 33,123 |
| NW\_003726819\_1 | 53,625 | 4 | 13,406 |
| NW\_003726820\_1 | 19,341 | 157 | 123 |
| NW\_003726823\_1 | 28,455 | 24 | 1,185 |
| NW\_003726826\_1 | 19,270 | 67 | 287 |
| NW\_003726830\_1 | 19,177 | 190 | 100 |
| NW\_003726831\_1 | 21,352 | 1 | 21,352 |
| NW\_003726833\_1 | 19,163 | 170 | 112 |
| NW\_003726835\_1 | 19,766 | 6 | 3,294 |
| NW\_003726837\_1 | 19,964 | 1 | 19,964 |
| NW\_003726839\_1 | 37,094 | 2 | 18,547 |
| NW\_003726842\_1 | 21,618 | 109 | 198 |
| NW\_003726847\_1 | 18,954 | 14 | 1,353 |
| NW\_003726855\_1 | 20,000 | 11 | 1,818 |
| NW\_003726858\_1 | 18,807 | 46 | 408 |
| NW\_003726866\_1 | 18,815 | 1 | 18,815 |
| NW\_003726869\_1 | 18,667 | 71 | 262 |
| NW\_003726872\_1 | 23,772 | 126 | 188 |
| NW\_003726875\_1 | 21,006 | 2 | 10,503 |
| NW\_003726879\_1 | 27,860 | 3 | 9,286 |
| NW\_003726880\_1 | 18,404 | 101 | 182 |
| NW\_003726881\_1 | 18,737 | 47 | 398 |
| NW\_003726885\_1 | 30,129 | 1 | 30,129 |
| NW\_003726887\_1 | 18,280 | 128 | 142 |
| NW\_003726889\_1 | 18,258 | 78 | 234 |
| NW\_003726895\_1 | 18,120 | 12 | 1,510 |
| NW\_003726913\_1 | 17,768 | 1 | 17,768 |
| NW\_003726919\_1 | 17,621 | 153 | 115 |
| NW\_003726920\_1 | 19,944 | 9 | 2,216 |
| NW\_003726922\_1 | 23,498 | 1 | 23,498 |
| NW\_003726924\_1 | 17,500 | 89 | 196 |
| NW\_003726927\_1 | 17,485 | 57 | 306 |
| NW\_003726928\_1 | 17,439 | 8 | 2,179 |
| NW\_003726935\_1 | 17,333 | 43 | 403 |
| NW\_003726937\_1 | 17,237 | 84 | 205 |
| NW\_003726938\_1 | 17,223 | 1 | 17,223 |
| NW\_003726948\_1 | 16,999 | 11 | 1,545 |
| NW\_003726956\_1 | 20,014 | 2 | 10,007 |
| NW\_003726959\_1 | 16,645 | 6 | 2,774 |
| NW\_003726963\_1 | 16,573 | 35 | 473 |
| NW\_003726973\_1 | 16,411 | 14 | 1,172 |
| NW\_003726974\_1 | 16,406 | 44 | 372 |
| NW\_003726980\_1 | 16,274 | 4 | 4,068 |
| NW\_003726981\_1 | 16,274 | 10 | 1,627 |
| NW\_003726983\_1 | 16,219 | 65 | 249 |
| NW\_003726984\_1 | 19,561 | 7 | 2,794 |
| NW\_003726986\_1 | 16,168 | 77 | 209 |
| NW\_003726989\_1 | 18,404 | 143 | 128 |
| NW\_003726992\_1 | 16,101 | 10 | 1,610 |
| NW\_003726998\_1 | 16,945 | 34 | 498 |
| NW\_003727000\_1 | 19,044 | 6 | 3,174 |
| NW\_003727002\_1 | 15,997 | 2 | 7,998 |
| NW\_003727005\_1 | 18,714 | 2 | 9,357 |
| NW\_003727012\_1 | 15,960 | 9 | 1,773 |
| NW\_003727013\_1 | 15,826 | 2 | 7,913 |
| NW\_003727015\_1 | 22,770 | 60 | 379 |
| NW\_003727032\_1 | 15,586 | 43 | 362 |
| NW\_003727033\_1 | 15,927 | 1 | 15,927 |
| NW\_003727035\_1 | 15,954 | 2 | 7,977 |
| NW\_003727038\_1 | 15,446 | 8 | 1,930 |
| NW\_003727044\_1 | 26,016 | 7 | 3,716 |
| NW\_003727047\_1 | 15,314 | 18 | 850 |
| NW\_003727053\_1 | 15,218 | 141 | 107 |
| NW\_003727060\_1 | 15,083 | 79 | 190 |
| NW\_003727061\_1 | 15,081 | 41 | 367 |
| NW\_003727062\_1 | 15,050 | 10 | 1,505 |
| NW\_003727065\_1 | 18,475 | 5 | 3,695 |
| NW\_003727067\_1 | 17,792 | 39 | 456 |
| NW\_003727092\_1 | 15,008 | 5 | 3,001 |
| NW\_003727099\_1 | 14,509 | 27 | 537 |
| NW\_003727104\_1 | 14,413 | 25 | 576 |
| NW\_003727105\_1 | 24,251 | 3 | 8,083 |
| NW\_003727109\_1 | 14,354 | 59 | 243 |
| NW\_003727115\_1 | 16,678 | 2 | 8,339 |
| NW\_003727116\_1 | 14,305 | 90 | 158 |
| NW\_003727117\_1 | 14,300 | 64 | 223 |
| NW\_003727119\_1 | 14,274 | 2 | 7,137 |
| NW\_003727127\_1 | 14,197 | 28 | 507 |
| NW\_003727129\_1 | 14,321 | 127 | 112 |
| NW\_003727132\_1 | 20,078 | 11 | 1,825 |
| NW\_003727133\_1 | 14,123 | 90 | 156 |
| NW\_003727135\_1 | 14,570 | 26 | 560 |
| NW\_003727136\_1 | 14,399 | 12 | 1,199 |
| NW\_003727138\_1 | 14,077 | 70 | 201 |
| NW\_003727141\_1 | 18,091 | 69 | 262 |
| NW\_003727143\_1 | 19,932 | 1 | 19,932 |
| NW\_003727146\_1 | 17,760 | 106 | 167 |
| NW\_003727149\_1 | 17,765 | 17 | 1,045 |
| NW\_003727157\_1 | 18,815 | 3 | 6,271 |
| NW\_003727159\_1 | 26,714 | 3 | 8,904 |
| NW\_003727161\_1 | 13,787 | 2 | 6,893 |
| NW\_003727164\_1 | 13,729 | 5 | 2,745 |
| NW\_003727165\_1 | 13,717 | 78 | 175 |
| NW\_003727167\_1 | 21,823 | 6 | 3,637 |
| NW\_003727176\_1 | 16,606 | 17 | 976 |
| NW\_003727177\_1 | 13,589 | 38 | 357 |
| NW\_003727180\_1 | 13,585 | 2 | 6,792 |
| NW\_003727188\_1 | 16,064 | 57 | 281 |
| NW\_003727196\_1 | 13,330 | 46 | 289 |
| NW\_003727206\_1 | 23,501 | 6 | 3,916 |
| NW\_003727209\_1 | 13,242 | 7 | 1,891 |
| NW\_003727210\_1 | 13,231 | 2 | 6,615 |
| NW\_003727215\_1 | 13,152 | 114 | 115 |
| NW\_003727216\_1 | 15,666 | 8 | 1,958 |
| NW\_003727217\_1 | 15,908 | 3 | 5,302 |
| NW\_003727218\_1 | 13,146 | 111 | 118 |
| NW\_003727223\_1 | 14,890 | 32 | 465 |
| NW\_003727238\_1 | 12,940 | 128 | 101 |
| NW\_003727240\_1 | 16,589 | 13 | 1,276 |
| NW\_003727242\_1 | 13,514 | 19 | 711 |
| NW\_003727244\_1 | 12,896 | 3 | 4,298 |
| NW\_003727246\_1 | 12,820 | 9 | 1,424 |
| NW\_003727249\_1 | 12,809 | 3 | 4,269 |
| NW\_003727253\_1 | 12,732 | 1 | 12,732 |
| NW\_003727255\_1 | 12,718 | 49 | 259 |
| NW\_003727256\_1 | 12,715 | 57 | 223 |
| NW\_003727257\_1 | 12,710 | 6 | 2,118 |
| NW\_003727263\_1 | 15,591 | 30 | 519 |
| NW\_003727264\_1 | 12,644 | 33 | 383 |
| NW\_003727270\_1 | 12,569 | 123 | 102 |
| NW\_003727282\_1 | 12,486 | 1 | 12,486 |
| NW\_003727284\_1 | 24,688 | 15 | 1,645 |
| NW\_003727286\_1 | 12,450 | 22 | 565 |
| NW\_003727291\_1 | 13,870 | 5 | 2,774 |
| NW\_003727298\_1 | 13,665 | 2 | 6,832 |
| NW\_003727302\_1 | 12,224 | 11 | 1,111 |
| NW\_003727305\_1 | 13,126 | 22 | 596 |
| NW\_003727306\_1 | 15,591 | 5 | 3,118 |
| NW\_003727321\_1 | 17,590 | 1 | 17,590 |
| NW\_003727323\_1 | 12,099 | 42 | 288 |
| NW\_003727324\_1 | 12,095 | 22 | 549 |
| NW\_003727329\_1 | 12,070 | 27 | 447 |
| NW\_003727331\_1 | 14,862 | 24 | 619 |
| NW\_003727349\_1 | 15,148 | 41 | 369 |
| NW\_003727351\_1 | 11,841 | 57 | 207 |
| NW\_003727353\_1 | 17,963 | 14 | 1,283 |
| NW\_003727360\_1 | 12,857 | 2 | 6,428 |
| NW\_003727364\_1 | 11,732 | 62 | 189 |
| NW\_003727369\_1 | 11,686 | 61 | 191 |
| NW\_003727374\_1 | 11,653 | 4 | 2,913 |
| NW\_003727376\_1 | 11,635 | 3 | 3,878 |
| NW\_003727384\_1 | 11,578 | 56 | 206 |
| NW\_003727388\_1 | 14,150 | 23 | 615 |
| NW\_003727392\_1 | 11,546 | 1 | 11,546 |
| NW\_003727404\_1 | 14,051 | 7 | 2,007 |
| NW\_003727405\_1 | 15,624 | 4 | 3,906 |
| NW\_003727411\_1 | 11,390 | 19 | 599 |
| NW\_003727413\_1 | 13,788 | 30 | 459 |
| NW\_003727417\_1 | 11,342 | 1 | 11,342 |
| NW\_003727423\_1 | 11,270 | 28 | 402 |
| NW\_003727429\_1 | 11,448 | 39 | 293 |
| NW\_003727433\_1 | 12,555 | 52 | 241 |
| NW\_003727450\_1 | 16,619 | 6 | 2,769 |
| NW\_003727464\_1 | 11,030 | 34 | 324 |
| NW\_003727474\_1 | 10,880 | 86 | 126 |
| NW\_003727478\_1 | 10,853 | 2 | 5,426 |
| NW\_003727483\_1 | 10,837 | 32 | 338 |
| NW\_003727489\_1 | 10,792 | 59 | 182 |
| NW\_003727490\_1 | 13,507 | 33 | 409 |
| NW\_003727501\_1 | 10,696 | 16 | 668 |
| NW\_003727507\_1 | 11,838 | 22 | 538 |
| NW\_003727508\_1 | 10,664 | 88 | 121 |
| NW\_003727509\_1 | 11,181 | 6 | 1,863 |
| NW\_003727510\_1 | 13,538 | 31 | 436 |
| NW\_003727512\_1 | 10,625 | 13 | 817 |
| NW\_003727522\_1 | 10,537 | 10 | 1,053 |
| NW\_003727533\_1 | 10,500 | 44 | 238 |
| NW\_003727545\_1 | 16,700 | 4 | 4,175 |
| NW\_003727546\_1 | 10,403 | 58 | 179 |
| NW\_003727565\_1 | 10,311 | 1 | 10,311 |
| NW\_003727572\_1 | 10,592 | 34 | 311 |
| NW\_003727579\_1 | 10,589 | 10 | 1,058 |
| NW\_003727582\_1 | 11,833 | 16 | 739 |
| NW\_003727601\_1 | 13,354 | 10 | 1,335 |
| NW\_003727611\_1 | 13,874 | 44 | 315 |
| NW\_003727617\_1 | 10,065 | 79 | 127 |
| NW\_003727618\_1 | 10,093 | 17 | 593 |
| NW\_003727621\_1 | 10,484 | 41 | 255 |
| NW\_003727629\_1 | 11,521 | 36 | 320 |
| NW\_003727630\_1 | 11,079 | 9 | 1,231 |
| NW\_003727634\_1 | 15,551 | 36 | 431 |
| NW\_003727639\_1 | 9,913 | 54 | 183 |
| NW\_003727640\_1 | 9,908 | 12 | 825 |
| NW\_003727646\_1 | 9,843 | 22 | 447 |
| NW\_003727647\_1 | 12,747 | 11 | 1,158 |
| NW\_003727649\_1 | 9,812 | 89 | 110 |
| NW\_003727655\_1 | 9,786 | 30 | 326 |
| NW\_003727659\_1 | 9,731 | 6 | 1,621 |
| NW\_003727665\_1 | 9,679 | 88 | 109 |
| NW\_003727667\_1 | 9,657 | 81 | 119 |
| NW\_003727674\_1 | 10,330 | 13 | 794 |
| NW\_003727682\_1 | 10,339 | 3 | 3,446 |
| NW\_003727684\_1 | 9,548 | 70 | 136 |
| NW\_003727690\_1 | 13,392 | 4 | 3,348 |
| NW\_003727698\_1 | 9,481 | 75 | 126 |
| NW\_003727702\_1 | 9,453 | 78 | 121 |
| NW\_003727707\_1 | 15,866 | 2 | 7,933 |
| NW\_003727718\_1 | 11,135 | 2 | 5,567 |
| NW\_003727727\_1 | 9,334 | 6 | 1,555 |
| NW\_003727735\_1 | 9,301 | 16 | 581 |
| NW\_003727763\_1 | 9,159 | 31 | 295 |
| NW\_003727765\_1 | 9,150 | 6 | 1,525 |
| NW\_003727773\_1 | 9,120 | 36 | 253 |
| NW\_003727775\_1 | 9,112 | 29 | 314 |
| NW\_003727776\_1 | 12,554 | 1 | 12,554 |
| NW\_003727793\_1 | 9,031 | 21 | 430 |
| NW\_003727798\_1 | 9,000 | 3 | 3,000 |
| NW\_003727811\_1 | 10,513 | 5 | 2,102 |
| NW\_003727812\_1 | 8,942 | 33 | 270 |
| NW\_003727816\_1 | 11,959 | 12 | 996 |
| NW\_003727818\_1 | 8,930 | 9 | 992 |
| NW\_003727820\_1 | 8,988 | 21 | 428 |
| NW\_003727825\_1 | 8,890 | 25 | 355 |
| NW\_003727831\_1 | 11,392 | 78 | 146 |
| NW\_003727868\_1 | 8,677 | 40 | 216 |
| NW\_003727881\_1 | 8,616 | 11 | 783 |
| NW\_003727883\_1 | 8,611 | 62 | 138 |
| NW\_003727885\_1 | 8,606 | 42 | 204 |
| NW\_003727890\_1 | 8,572 | 12 | 714 |
| NW\_003727910\_1 | 8,489 | 8 | 1,061 |
| NW\_003727913\_1 | 8,468 | 32 | 264 |
| NW\_003727925\_1 | 8,415 | 42 | 200 |
| NW\_003727928\_1 | 8,409 | 56 | 150 |
| NW\_003727941\_1 | 9,862 | 27 | 365 |
| NW\_003727946\_1 | 8,339 | 2 | 4,169 |
| NW\_003727951\_1 | 8,308 | 43 | 193 |
| NW\_003727972\_1 | 8,235 | 54 | 152 |
| NW\_003727975\_1 | 8,218 | 1 | 8,218 |
| NW\_003727979\_1 | 8,655 | 58 | 149 |
| NW\_003727983\_1 | 8,194 | 33 | 248 |
| NW\_003727984\_1 | 8,193 | 48 | 170 |
| NW\_003727996\_1 | 8,154 | 4 | 2,038 |
| NW\_003728001\_1 | 8,141 | 20 | 407 |
| NW\_003728005\_1 | 8,118 | 2 | 4,059 |
| NW\_003728012\_1 | 8,085 | 20 | 404 |
| NW\_003728026\_1 | 8,038 | 37 | 217 |
| NW\_003728045\_1 | 9,411 | 3 | 3,137 |
| NW\_003728053\_1 | 7,951 | 29 | 274 |
| NW\_003728055\_1 | 11,651 | 9 | 1,294 |
| NW\_003728056\_1 | 7,942 | 5 | 1,588 |
| NW\_003728059\_1 | 8,109 | 17 | 477 |
| NW\_003728060\_1 | 8,353 | 3 | 2,784 |
| NW\_003728064\_1 | 7,905 | 61 | 129 |
| NW\_003728076\_1 | 7,858 | 71 | 110 |
| NW\_003728077\_1 | 7,857 | 1 | 7,857 |
| NW\_003728084\_1 | 13,468 | 57 | 236 |
| NW\_003728088\_1 | 7,822 | 45 | 173 |
| NW\_003728089\_1 | 7,821 | 22 | 355 |
| NW\_003728091\_1 | 7,819 | 11 | 710 |
| NW\_003728092\_1 | 7,816 | 17 | 459 |
| NW\_003728097\_1 | 7,802 | 24 | 325 |
| NW\_003728107\_1 | 7,767 | 40 | 194 |
| NW\_003728109\_1 | 7,758 | 16 | 484 |
| NW\_003728139\_1 | 7,680 | 10 | 768 |
| NW\_003728154\_1 | 7,630 | 25 | 305 |
| NW\_003728163\_1 | 7,603 | 68 | 111 |
| NW\_003728167\_1 | 11,127 | 2 | 5,563 |
| NW\_003728176\_1 | 7,556 | 2 | 3,778 |
| NW\_003728179\_1 | 7,533 | 1 | 7,533 |
| NW\_003728180\_1 | 10,986 | 26 | 422 |
| NW\_003728182\_1 | 7,528 | 31 | 242 |
| NW\_003728183\_1 | 7,523 | 1 | 7,523 |
| NW\_003728186\_1 | 10,377 | 22 | 471 |
| NW\_003728199\_1 | 7,466 | 12 | 622 |
| NW\_003728207\_1 | 7,450 | 15 | 496 |
| NW\_003728208\_1 | 7,455 | 1 | 7,455 |
| NW\_003728238\_1 | 7,339 | 49 | 149 |
| NW\_003728239\_1 | 7,339 | 55 | 133 |
| NW\_003728240\_1 | 9,688 | 35 | 276 |
| NW\_003728257\_1 | 7,281 | 47 | 154 |
| NW\_003728277\_1 | 7,227 | 3 | 2,409 |
| NW\_003728283\_1 | 7,214 | 1 | 7,214 |
| NW\_003728285\_1 | 7,210 | 29 | 248 |
| NW\_003728301\_1 | 7,164 | 2 | 3,582 |
| NW\_003728310\_1 | 7,142 | 59 | 121 |
| NW\_003728324\_1 | 7,099 | 3 | 2,366 |
| NW\_003728328\_1 | 13,114 | 13 | 1,008 |
| NW\_003728330\_1 | 7,066 | 49 | 144 |
| NW\_003728334\_1 | 7,058 | 6 | 1,176 |
| NW\_003728347\_1 | 7,017 | 3 | 2,339 |
| NW\_003728357\_1 | 6,989 | 18 | 388 |
| NW\_003728358\_1 | 6,998 | 5 | 1,399 |
| NW\_003728362\_1 | 6,977 | 27 | 258 |
| NW\_003728365\_1 | 9,343 | 5 | 1,868 |
| NW\_003728371\_1 | 6,945 | 2 | 3,472 |
| NW\_003728374\_1 | 6,942 | 31 | 223 |
| NW\_003728396\_1 | 6,893 | 3 | 2,297 |
| NW\_003728406\_1 | 6,862 | 1 | 6,862 |
| NW\_003728408\_1 | 7,248 | 15 | 483 |
| NW\_003728409\_1 | 6,855 | 2 | 3,427 |
| NW\_003728431\_1 | 6,812 | 20 | 340 |
| NW\_003728442\_1 | 6,784 | 11 | 616 |
| NW\_003728448\_1 | 6,755 | 21 | 321 |
| NW\_003728453\_1 | 6,732 | 6 | 1,122 |
| NW\_003728457\_1 | 6,731 | 64 | 105 |
| NW\_003728458\_1 | 11,326 | 2 | 5,663 |
| NW\_003728460\_1 | 6,714 | 27 | 248 |
| NW\_003728462\_1 | 6,712 | 11 | 610 |
| NW\_003728463\_1 | 6,698 | 5 | 1,339 |
| NW\_003728470\_1 | 6,686 | 13 | 514 |
| NW\_003728477\_1 | 6,667 | 5 | 1,333 |
| NW\_003728488\_1 | 6,742 | 5 | 1,348 |
| NW\_003728492\_1 | 6,611 | 26 | 254 |
| NW\_003728514\_1 | 6,684 | 13 | 514 |
| NW\_003728521\_1 | 6,533 | 49 | 133 |
| NW\_003728528\_1 | 6,518 | 25 | 260 |
| NW\_003728539\_1 | 6,481 | 19 | 341 |
| NW\_003728554\_1 | 6,427 | 5 | 1,285 |
| NW\_003728558\_1 | 6,422 | 2 | 3,211 |
| NW\_003728572\_1 | 6,389 | 42 | 152 |
| NW\_003728574\_1 | 6,380 | 12 | 531 |
| NW\_003728578\_1 | 6,354 | 5 | 1,270 |
| NW\_003728585\_1 | 6,342 | 15 | 422 |
| NW\_003728595\_1 | 7,225 | 7 | 1,032 |
| NW\_003728620\_1 | 6,266 | 12 | 522 |
| NW\_003728621\_1 | 6,266 | 38 | 164 |
| NW\_003728632\_1 | 6,246 | 42 | 148 |
| NW\_003728644\_1 | 6,223 | 57 | 109 |
| NW\_003728652\_1 | 6,208 | 17 | 365 |
| NW\_003728653\_1 | 6,207 | 2 | 3,103 |
| NW\_003728657\_1 | 6,192 | 10 | 619 |
| NW\_003728664\_1 | 8,707 | 2 | 4,353 |
| NW\_003728668\_1 | 6,173 | 6 | 1,028 |
| NW\_003728670\_1 | 9,546 | 5 | 1,909 |
| NW\_003728680\_1 | 6,145 | 36 | 170 |
| NW\_003728682\_1 | 6,144 | 15 | 409 |
| NW\_003728687\_1 | 6,133 | 17 | 360 |
| NW\_003728689\_1 | 6,131 | 5 | 1,226 |
| NW\_003728703\_1 | 6,084 | 5 | 1,216 |
| NW\_003728707\_1 | 6,061 | 34 | 178 |
| NW\_003728732\_1 | 6,000 | 6 | 1,000 |
| NW\_003728743\_1 | 5,964 | 1 | 5,964 |
| NW\_003728766\_1 | 5,888 | 3 | 1,962 |
| NW\_003728770\_1 | 5,880 | 8 | 735 |
| NW\_003728781\_1 | 5,852 | 27 | 216 |
| NW\_003728783\_1 | 5,842 | 7 | 834 |
| NW\_003728787\_1 | 5,818 | 53 | 109 |
| NW\_003728799\_1 | 5,804 | 31 | 187 |
| NW\_003728802\_1 | 5,793 | 32 | 181 |
| NW\_003728808\_1 | 5,773 | 3 | 1,924 |
| NW\_003728809\_1 | 5,768 | 18 | 320 |
| NW\_003728814\_1 | 5,747 | 4 | 1,436 |
| NW\_003728818\_1 | 5,723 | 57 | 100 |
| NW\_003728821\_1 | 5,712 | 25 | 228 |
| NW\_003728829\_1 | 5,694 | 49 | 116 |
| NW\_003728837\_1 | 5,678 | 22 | 258 |
| NW\_003728858\_1 | 5,629 | 16 | 351 |
| NW\_003728868\_1 | 5,605 | 54 | 103 |
| NW\_003728870\_1 | 5,588 | 9 | 620 |
| NW\_003728873\_1 | 5,579 | 3 | 1,859 |
| NW\_003728885\_1 | 5,549 | 4 | 1,387 |
| NW\_003728891\_1 | 5,531 | 10 | 553 |
| NW\_003728899\_1 | 5,496 | 9 | 610 |
| NW\_003728912\_1 | 8,038 | 8 | 1,004 |
| NW\_003728923\_1 | 5,416 | 33 | 164 |
| NW\_003728928\_1 | 5,404 | 1 | 5,404 |
| NW\_003728943\_1 | 5,366 | 28 | 191 |
| NW\_003728944\_1 | 5,363 | 1 | 5,363 |
| NW\_003728952\_1 | 5,330 | 28 | 190 |
| NW\_003728964\_1 | 5,295 | 5 | 1,059 |
| NW\_003728970\_1 | 5,290 | 24 | 220 |
| NW\_003728973\_1 | 5,288 | 4 | 1,322 |
| NW\_003728986\_1 | 5,246 | 45 | 116 |
| NW\_003728988\_1 | 5,243 | 2 | 2,621 |
| NW\_003729004\_1 | 5,174 | 41 | 126 |
| NW\_003729009\_1 | 5,154 | 3 | 1,718 |
| NW\_003729013\_1 | 5,147 | 22 | 233 |
| NW\_003729014\_1 | 5,137 | 2 | 2,568 |
| NW\_003729020\_1 | 5,123 | 40 | 128 |
| NW\_003729025\_1 | 5,115 | 32 | 159 |
| NW\_003729038\_1 | 5,054 | 49 | 103 |
| NW\_003729040\_1 | 5,051 | 19 | 265 |
| NW\_003729050\_1 | 5,015 | 1 | 5,015 |
| NW\_003729051\_1 | 5,010 | 25 | 200 |
| NW\_003729060\_1 | 4,989 | 8 | 623 |
| NW\_003729061\_1 | 4,988 | 2 | 2,494 |
| NW\_003729070\_1 | 4,967 | 22 | 225 |
| NW\_003729071\_1 | 4,965 | 9 | 551 |
| NW\_003729076\_1 | 5,700 | 3 | 1,900 |
| NW\_003729087\_1 | 4,922 | 1 | 4,922 |
| NW\_003729088\_1 | 4,921 | 2 | 2,460 |
| NW\_003729093\_1 | 4,893 | 38 | 128 |
| NW\_003729105\_1 | 4,863 | 26 | 187 |
| NW\_003729115\_1 | 4,818 | 25 | 192 |
| NW\_003729117\_1 | 4,798 | 32 | 149 |
| NW\_003729124\_1 | 4,784 | 2 | 2,392 |
| NW\_003729148\_1 | 4,694 | 2 | 2,347 |
| NW\_003729155\_1 | 4,676 | 28 | 167 |
| NW\_003729158\_1 | 4,669 | 3 | 1,556 |
| NW\_003729172\_1 | 4,601 | 3 | 1,533 |
| NW\_003729173\_1 | 4,600 | 13 | 353 |
| NW\_003729178\_1 | 4,577 | 4 | 1,144 |
| NW\_003729189\_1 | 4,550 | 19 | 239 |
| NW\_003729191\_1 | 4,541 | 5 | 908 |
| NW\_003729192\_1 | 4,539 | 13 | 349 |
| NW\_003729203\_1 | 4,454 | 8 | 556 |
| NW\_003729207\_1 | 4,439 | 38 | 116 |
| Total | 2,363,967,083 | 15,765,115 | 149 |


---


 **Number variantss by type**

| **Type** | **Total** |
| --- | --- |
| **SNP** | 13,333,728 |
| **MNP** | 0 |
| **INS** | 1,160,954 |
| **DEL** | 1,270,433 |
| **MIXED** | 0 |
| **INTERVAL** | 0 |
| **Total** | 15,765,115 |
| --- | --- |


---


 **Number of effects by impact** 

| **Type (alphabetical order)** |  | Count | Percent |
| --- | --- | --- | --- |
| **HIGH** |  | 5,824 | 0.016% |
| **LOW** |  | 230,267 | 0.633% |
| **MODERATE** |  | 103,802 | 0.285% |
| **MODIFIER** |  | 36,022,268 | 99.065% |


---


 **Number of effects by functional class** 

| **Type (alphabetical order)** |  | Count | Percent |
| --- | --- | --- | --- |
| **MISSENSE** |  | 99,895 | 35.245% |
| **NONSENSE** |  | 775 | 0.273% |
| **SILENT** |  | 182,759 | 64.481% |

  

Missense / Silent ratio: 0.5466


---


 **Number of effects by type and region** 

| Type | Region |
| --- | --- |
| | **Type (alphabetical order)** |  | Count | Percent | | --- | --- | --- | --- | | **3\_prime\_UTR\_variant** |  | 301,343 | 0.829% | | **5\_prime\_UTR\_premature\_start\_codon\_gain\_variant** |  | 10,644 | 0.029% | | **5\_prime\_UTR\_truncation+exon\_loss\_variant** |  | 8 | 0% | | **5\_prime\_UTR\_variant** |  | 79,370 | 0.218% | | **chromosome\_number\_variation** |  | 1 | 0% | | **disruptive\_inframe\_deletion** |  | 838 | 0.002% | | **disruptive\_inframe\_deletion+splice\_region\_variant** |  | 4 | 0% | | **disruptive\_inframe\_insertion** |  | 713 | 0.002% | | **disruptive\_inframe\_insertion+splice\_region\_variant** |  | 4 | 0% | | **downstream\_gene\_variant** |  | 2,041,130 | 5.613% | | **exon\_loss\_variant+splice\_donor\_variant+splice\_region\_variant+intron\_variant** |  | 2 | 0% | | **frameshift\_variant** |  | 2,360 | 0.006% | | **frameshift\_variant+splice\_acceptor\_variant+splice\_region\_variant+intron\_variant** |  | 27 | 0% | | **frameshift\_variant+splice\_donor\_variant+splice\_region\_variant+intron\_variant** |  | 49 | 0% | | **frameshift\_variant+splice\_region\_variant** |  | 75 | 0% | | **frameshift\_variant+start\_lost** |  | 25 | 0% | | **frameshift\_variant+start\_lost+splice\_region\_variant** |  | 6 | 0% | | **frameshift\_variant+stop\_gained** |  | 44 | 0% | | **frameshift\_variant+stop\_gained+splice\_region\_variant** |  | 11 | 0% | | **frameshift\_variant+stop\_lost** |  | 41 | 0% | | **frameshift\_variant+stop\_lost+splice\_acceptor\_variant+splice\_region\_variant+intron\_variant** |  | 2 | 0% | | **frameshift\_variant+stop\_lost+splice\_region\_variant** |  | 3 | 0% | | **inframe\_deletion** |  | 1,565 | 0.004% | | **inframe\_deletion+splice\_region\_variant** |  | 1 | 0% | | **inframe\_insertion** |  | 1,300 | 0.004% | | **inframe\_insertion+splice\_region\_variant** |  | 7 | 0% | | **initiator\_codon\_variant** |  | 32 | 0% | | **intergenic\_region** |  | 8,422,158 | 23.162% | | **intragenic\_variant** |  | 2,433 | 0.007% | | **intron\_variant** |  | 23,047,490 | 63.383% | | **missense\_variant** |  | 97,416 | 0.268% | | **missense\_variant+splice\_region\_variant** |  | 1,946 | 0.005% | | **non\_coding\_exon\_variant** |  | 111,453 | 0.307% | | **non\_coding\_transcript\_variant** |  | 290 | 0.001% | | **splice\_acceptor\_variant+3\_prime\_UTR\_variant+intron\_variant** |  | 2 | 0% | | **splice\_acceptor\_variant+disruptive\_inframe\_deletion+splice\_region\_variant+intron\_variant** |  | 1 | 0% | | **splice\_acceptor\_variant+inframe\_deletion+splice\_region\_variant+intron\_variant** |  | 7 | 0% | | **splice\_acceptor\_variant+intron\_variant** |  | 824 | 0.002% | | **splice\_acceptor\_variant+splice\_donor\_variant+splice\_region\_variant+intron\_variant** |  | 3 | 0% | | **splice\_acceptor\_variant+splice\_region\_variant+5\_prime\_UTR\_variant+intron\_variant** |  | 8 | 0% | | **splice\_acceptor\_variant+splice\_region\_variant+intron\_variant** |  | 23 | 0% | | **splice\_acceptor\_variant+splice\_region\_variant+intron\_variant+non\_coding\_exon\_variant** |  | 16 | 0% | | **splice\_donor\_variant+3\_prime\_UTR\_variant+intron\_variant** |  | 2 | 0% | | **splice\_donor\_variant+5\_prime\_UTR\_variant+intron\_variant** |  | 4 | 0% | | **splice\_donor\_variant+disruptive\_inframe\_deletion+splice\_region\_variant+intron\_variant** |  | 3 | 0% | | **splice\_donor\_variant+inframe\_deletion+splice\_region\_variant+intron\_variant** |  | 4 | 0% | | **splice\_donor\_variant+intron\_variant** |  | 850 | 0.002% | | **splice\_donor\_variant+splice\_region\_variant+3\_prime\_UTR\_variant+intron\_variant** |  | 2 | 0% | | **splice\_donor\_variant+splice\_region\_variant+5\_prime\_UTR\_variant+intron\_variant** |  | 8 | 0% | | **splice\_donor\_variant+splice\_region\_variant+intron\_variant** |  | 70 | 0% | | **splice\_donor\_variant+splice\_region\_variant+intron\_variant+non\_coding\_exon\_variant** |  | 32 | 0% | | **splice\_region\_variant** |  | 1,697 | 0.005% | | **splice\_region\_variant+downstream\_gene\_variant** |  | 11 | 0% | | **splice\_region\_variant+intron\_variant** |  | 33,726 | 0.093% | | **splice\_region\_variant+non\_coding\_exon\_variant** |  | 1,399 | 0.004% | | **splice\_region\_variant+stop\_retained\_variant** |  | 17 | 0% | | **splice\_region\_variant+synonymous\_variant** |  | 3,993 | 0.011% | | **start\_lost** |  | 194 | 0.001% | | **start\_lost+disruptive\_inframe\_deletion** |  | 1 | 0% | | **start\_lost+inframe\_deletion** |  | 9 | 0% | | **start\_lost+splice\_region\_variant** |  | 9 | 0% | | **stop\_gained** |  | 748 | 0.002% | | **stop\_gained+disruptive\_inframe\_deletion** |  | 1 | 0% | | **stop\_gained+disruptive\_inframe\_insertion** |  | 2 | 0% | | **stop\_gained+inframe\_insertion** |  | 8 | 0% | | **stop\_gained+inframe\_insertion+splice\_region\_variant** |  | 7 | 0% | | **stop\_gained+splice\_region\_variant** |  | 27 | 0% | | **stop\_lost** |  | 281 | 0.001% | | **stop\_lost+disruptive\_inframe\_deletion** |  | 3 | 0% | | **stop\_lost+inframe\_deletion** |  | 10 | 0% | | **stop\_lost+inframe\_insertion** |  | 1 | 0% | | **stop\_lost+splice\_region\_variant** |  | 18 | 0% | | **stop\_retained\_variant** |  | 126 | 0% | | **synonymous\_variant** |  | 178,622 | 0.491% | | **upstream\_gene\_variant** |  | 2,016,601 | 5.546% | | | **Type (alphabetical order)** |  | Count | Percent | | --- | --- | --- | --- | | **DOWNSTREAM** |  | 2,041,130 | 5.613% | | **EXON** |  | 397,746 | 1.094% | | **INTERGENIC** |  | 8,422,158 | 23.162% | | **INTRON** |  | 23,047,490 | 63.383% | | **NONE** |  | 2,724 | 0.007% | | **SPLICE\_SITE\_ACCEPTOR** |  | 884 | 0.002% | | **SPLICE\_SITE\_DONOR** |  | 975 | 0.003% | | **SPLICE\_SITE\_REGION** |  | 40,843 | 0.112% | | **TRANSCRIPT** |  | 245 | 0.001% | | **UPSTREAM** |  | 2,016,601 | 5.546% | | **UTR\_3\_PRIME** |  | 301,343 | 0.829% | | **UTR\_5\_PRIME** |  | 90,022 | 0.248% | |


---


 **Quality:**

```
|  |  |
| --- | --- |
| Min | 20 |
| Max | 228 |
| Mean | 116.777 |
| Median | 112 |
| Standard deviation | 58.697 |
| Values | 20,21,22,23,24,25,26,27,28,29,30,31,32,33,34,35,36,37,38,39,40,41,42,43,44,45,46,47,48,49,50,51,52,53,54,55,56,57,58,59,60,61,62,63,64,65,66,67,68,69,70,71,72,73,74,75,76,77,78,79,80,81,82,83,84,85,86,87,88,89,90,91,92,93,94,95,96,97,98,99,100,101,102,103,104,105,106,107,108,109,110,111,112,113,114,115,116,117,118,119,120,121,122,123,124,125,126,127,128,129,130,131,132,133,134,135,136,137,138,139,140,141,142,143,144,145,146,147,148,149,150,151,152,153,154,155,156,157,158,159,160,161,162,163,164,165,166,167,168,169,170,171,172,173,174,175,176,177,178,179,180,181,182,183,184,185,186,187,188,189,190,191,192,193,194,195,196,197,198,199,200,201,202,203,204,205,206,207,208,209,210,211,212,213,214,215,216,217,218,219,220,221,222,223,224,225,226,227,228 |
| Count | 70829,67119,126831,71722,60705,65244,67818,61186,67152,68499,72299,78728,77595,71569,77683,74142,84672,78327,88085,75819,80485,110165,98827,131089,84311,112134,72360,75743,95461,75224,95977,72683,101709,72469,69886,66872,69966,77433,79192,75116,77888,77902,73792,82681,83778,88809,82096,81871,79114,92667,105157,79680,96115,78160,80499,76185,96412,79572,87856,84399,89922,112724,101936,140542,78109,101679,70842,74571,69054,74768,74748,75935,87281,75361,87046,75257,75580,75275,79160,79331,78389,82889,82873,91575,90490,102570,88270,107716,92091,126397,89586,117556,104501,79679,94475,101906,66467,97936,66940,78746,68073,70375,71168,75968,76083,76749,83976,73563,84461,78550,85098,87684,90236,85282,92147,82264,89207,77705,95765,76379,89659,89336,92975,93235,92054,70210,77692,66906,71222,70800,62759,68305,65545,68134,66160,66193,72751,72123,68521,75576,73811,78134,84407,71618,82117,68471,85679,64685,68355,59491,64312,59341,58243,53391,58588,56880,54757,58654,55700,61274,54932,59373,61444,56195,58655,56979,58179,53752,55758,55560,58461,51986,53488,48862,48457,41574,43324,41600,41087,39807,41325,40049,42067,41061,42091,42498,44729,39379,45685,35923,42232,37543,31870,36796,32394,32503,30278,30706,28845,28488,30273,28452,27943,27155,30315,25811,27822,25045,745539 |
```


---


 **Insertions and deletions length:**

```
|  |  |
| --- | --- |
| Min | 0 |
| Max | 99 |
| Mean | 2 |
| Median | 1 |
| Standard deviation | 3.934 |
| Values | 0,1,2,3,4,5,6,7,8,9,10,11,12,13,14,15,16,17,18,19,20,21,22,23,24,25,26,27,28,29,30,31,32,33,34,35,36,37,38,39,40,41,42,43,44,45,46,47,48,49,50,51,52,53,54,55,56,57,58,59,60,61,62,63,64,65,66,67,68,69,70,71,72,73,74,75,76,77,78,79,80,81,82,83,84,85,86,87,88,89,90,91,92,93,94,95,99 |
| Count | 420446,1474124,114861,149765,48306,45771,21347,31278,14084,18227,9143,15594,6627,8398,5249,6736,3459,4501,2777,3880,2424,2491,1975,2397,1701,1621,1398,1387,1077,1101,888,828,701,626,574,595,444,445,405,371,306,275,236,270,202,202,171,185,149,132,111,115,88,89,68,82,58,62,52,48,42,40,35,39,34,25,30,25,12,28,13,16,13,13,13,14,8,7,6,3,2,5,6,3,3,4,9,2,2,1,1,1,1,4,1,2,1 |
```


---


 **Base changes (SNPs)** 

|  |  |  |  |  |
| --- | --- | --- | --- | --- |
|  | **A** | **C** | **G** | **T** |
| **A** | 0 | 507,440 | 2,215,549 | 531,274 |
| **C** | 528,804 | 0 | 465,118 | 2,421,526 |
| **G** | 2,419,483 | 464,399 | 0 | 528,429 |
| **T** | 530,830 | 2,213,774 | 507,102 | 0 |

---


  **Ts/Tv (transitions / transversions)** 

**Note:** Only SNPs are used for this statistic.  
**Note:** This Ts/Tv ratio is a 'raw' ratio (ratio of observed events).

|  |  |
| --- | --- |
| Transitions | 18,227,939 |
| Transversions | 7,987,881 |
| Ts/Tv ratio | 2.2819 |

**All variants:**

```
Sample ,Ananku_nuclear_concat_auto.rmdup.bam,Total
Transitions ,18227939,18227939
Transversions ,7987881,7987881
Ts/Tv ,2.282,2.282
```

**Only known variants** (i.e. the ones having a non-empty ID field):

```
No results available (empty input?)
```

---


  **Allele frequency** 
  

|  |  |
| --- | --- |
| Min | 50 |
| Max | 100 |
| Mean | 98.203 |
| Median | 100 |
| Standard deviation | 9.307 |
| Values | 50,100 |
| Count | 562087,15078932 |

---


  **Allele Count** 
  

|  |  |
| --- | --- |
| Min | 1 |
| Max | 2 |
| Mean | 1.964 |
| Median | 2 |
| Standard deviation | 0.186 |
| Values | 1,2 |
| Count | 562087,15078932 |

---


  **Hom/Het per sample** 
  
  
  

```
Sample_names , Ananku_nuclear_concat_auto.rmdup.bam
Reference , 0
Het , 562087
Hom , 15078932
Missing , 0
```

---


 **Codon changes**

How to read this table:   
- Rows are reference codons and columns are changed codons. E.g. Row 'AAA' column 'TAA' indicates how many 'AAA' codons have been replaced by 'TAA' codons.  
- Red background colors indicate that more changes happened (heat-map).  
- Diagonals are indicated using grey background color   
- WARNING: This table may include different translation codon tables (e.g. mamalian DNA and mitochondrial DNA).

|  | - | AAA | AAC | AAG | AAT | ACA | ACC | ACG | ACT | AGA | AGC | AGG | AGT | ATA | ATC | ATG | ATT | CAA | CAC | CAG | CAT | CCA | CCC | CCG | CCT | CGA | CGC | CGG | CGT | CTA | CTC | CTG | CTT | GAA | GAC | GAG | GAT | GCA | GCC | GCG | GCT | GGA | GGC | GGG | GGT | GTA | GTC | GTG | GTT | NGC | NNN | TAA | TAC | TAG | TAT | TCA | TCC | TCG | TCT | TGA | TGC | TGG | TGT | TTA | TTC | TTG | TTT |
| --- | --- | --- | --- | --- | --- | --- | --- | --- | --- | --- | --- | --- | --- | --- | --- | --- | --- | --- | --- | --- | --- | --- | --- | --- | --- | --- | --- | --- | --- | --- | --- | --- | --- | --- | --- | --- | --- | --- | --- | --- | --- | --- | --- | --- | --- | --- | --- | --- | --- | --- | --- | --- | --- | --- | --- | --- | --- | --- | --- | --- | --- | --- | --- | --- | --- | --- | --- |
| - |  | 66 | 52 | 70 | 43 | 27 | 55 | 29 | 18 | 36 | 156 | 67 | 55 | 19 | 31 | 19 | 36 | 34 | 67 | 252 | 16 | 53 | 291 | 263 | 127 | 30 | 97 | 136 | 24 | 12 | 74 | 197 | 45 | 121 | 78 | 367 | 53 | 105 | 341 | 294 | 109 | 197 | 414 | 254 | 111 | 22 | 69 | 74 | 45 |  |  | 12 | 16 | 26 | 11 | 38 | 111 | 24 | 80 | 24 | 49 | 32 | 17 | 23 | 45 | 21 | 128 |
| AAA | 92 | 35 | 182 | 2,271 | 116 | 79 |  |  |  | 474 |  |  |  | 43 |  |  |  | 159 |  |  |  |  |  |  |  |  |  |  |  |  |  |  |  | 511 |  | 1 |  |  |  |  |  |  |  |  |  |  |  |  |  |  |  | 13 |  |  |  |  |  |  |  |  |  |  |  |  |  |  |  |
| AAC | 64 | 116 | 19 | 123 | 3,588 |  | 119 |  |  |  | 774 | 2 |  |  | 31 |  | 1 |  | 115 |  |  |  |  |  |  |  |  |  |  |  |  |  |  |  | 400 |  |  |  |  |  |  |  |  |  |  |  |  | 1 |  |  |  |  | 56 |  |  |  |  |  |  |  |  |  |  |  |  |  |  |
| AAG | 157 | 2,804 | 108 | 60 | 97 |  |  | 150 |  | 1 |  | 778 |  |  | 1 | 65 |  |  |  | 214 |  |  |  |  |  |  |  |  |  |  |  |  |  |  |  | 598 |  |  |  |  |  |  |  |  |  |  |  |  |  |  |  | 2 |  | 14 |  |  |  |  |  |  |  |  |  |  |  |  |  |
| AAT | 64 | 101 | 2,980 | 67 | 20 |  |  |  | 115 | 1 |  |  | 752 |  | 4 |  | 55 |  |  |  | 103 |  |  |  |  |  |  |  |  |  |  |  |  | 2 |  | 5 | 411 |  |  |  |  |  |  |  |  |  |  |  |  |  |  | 1 |  |  | 66 |  |  |  |  |  |  |  |  |  |  |  |  |
| ACA | 58 | 137 | 2 |  |  | 28 | 519 | 3,404 | 300 | 102 | 2 |  |  | 575 |  |  |  |  |  |  |  | 123 |  |  |  |  |  |  |  |  |  |  |  | 1 | 3 |  | 1 | 834 |  |  |  |  |  |  |  |  |  |  |  |  |  |  |  |  |  | 71 |  |  | 1 |  |  |  |  |  |  |  |  |
| ACC | 128 | 3 | 186 |  |  | 661 | 42 | 711 | 3,052 | 1 | 206 | 1 |  |  | 415 | 1 |  |  | 1 |  |  |  | 135 |  | 1 |  |  |  |  |  |  |  |  |  | 4 |  |  |  | 1,162 |  |  |  |  |  |  |  |  |  |  |  |  |  |  |  |  |  | 103 |  |  |  |  |  |  |  | 1 |  |  |
| ACG | 79 |  | 1 | 56 |  | 4,426 | 640 | 9 | 454 |  |  | 95 |  |  |  | 1,244 |  |  |  |  |  |  |  | 48 |  |  |  |  |  |  |  |  |  |  | 1 |  |  |  |  | 343 |  |  |  |  |  |  |  |  |  |  |  |  |  |  |  |  |  | 47 |  |  |  |  |  |  |  |  |  |
| ACT | 87 |  | 1 |  | 131 | 342 | 2,439 | 454 | 30 | 1 | 1 |  | 193 |  |  |  | 405 |  |  |  |  |  |  |  | 114 |  |  |  |  |  |  |  |  |  | 1 | 1 |  |  |  |  | 767 |  |  |  |  |  |  |  |  |  |  |  |  |  |  |  |  |  | 138 |  |  | 1 | 1 |  |  |  |  |
| AGA | 102 | 469 |  | 1 |  | 75 | 2 |  |  | 22 | 119 | 1,063 | 26 | 67 |  | 1 |  |  |  | 7 |  |  |  |  |  | 301 |  |  |  |  |  |  |  | 1 |  | 3 |  |  |  |  |  | 383 |  |  |  |  |  |  |  |  |  |  |  |  |  |  |  |  |  | 19 |  |  |  |  |  |  |  |
| AGC | 231 | 5 | 829 | 2 | 6 | 3 | 208 | 7 |  | 89 | 73 | 139 | 2,936 |  | 72 | 1 |  | 3 |  | 1 |  | 9 |  | 1 |  |  | 66 |  |  |  | 2 |  |  |  |  | 5 |  |  | 1 |  |  |  | 801 |  |  |  |  |  |  |  |  |  | 1 |  |  |  |  | 1 |  |  | 88 |  |  |  |  |  |  |
| AGG | 112 | 1 | 1 | 818 |  |  |  | 35 | 1 | 1,168 | 123 | 20 | 71 |  |  | 61 |  |  |  | 1 |  | 1 |  |  |  |  |  | 502 |  |  |  |  |  |  |  | 2 |  |  |  | 2 |  |  | 1 | 505 |  |  |  |  |  |  |  |  |  |  |  |  |  |  |  |  |  | 43 |  |  |  |  |  |
| AGT | 65 | 1 |  | 1 | 791 | 1 |  |  | 212 | 68 | 2,079 | 120 | 38 |  | 2 | 1 | 67 |  |  | 1 |  |  |  |  |  |  |  |  | 41 |  |  |  |  |  |  |  |  |  |  |  |  |  |  |  | 447 |  |  |  |  |  |  |  |  |  |  |  |  |  |  |  |  |  | 59 |  |  |  |  |
| ATA | 20 | 53 |  |  | 3 | 324 |  | 1 |  | 55 |  |  |  | 23 | 346 | 409 | 228 |  |  |  |  |  |  |  |  |  |  |  |  | 99 |  |  |  |  |  |  |  | 4 |  |  |  |  |  |  |  | 677 |  |  |  |  |  |  |  |  |  |  | 1 |  |  |  |  |  |  | 44 |  |  |  |
| ATC | 36 |  | 58 |  |  |  | 345 |  |  | 1 | 103 | 1 |  | 409 | 8 | 94 | 2,749 |  |  |  | 1 |  |  |  |  |  |  |  |  |  | 127 |  |  |  |  | 1 | 3 |  |  |  |  |  |  |  |  |  | 974 |  |  |  |  |  |  |  | 1 |  |  |  |  |  |  |  |  |  | 62 |  |  |
| ATG | 85 | 6 |  | 107 | 5 |  |  | 1,133 | 1 | 1 | 9 | 66 | 1 | 629 | 82 | 41 | 171 |  |  |  | 1 | 1 |  | 1 |  |  |  |  |  |  |  | 318 |  |  |  |  | 6 |  |  |  |  |  |  |  |  | 1 |  | 1,051 |  |  |  |  |  |  | 2 |  |  |  |  |  |  |  |  |  |  | 207 | 1 |
| ATT | 45 |  |  |  | 39 |  |  | 1 | 313 |  | 1 |  | 35 | 313 | 2,238 | 91 | 10 |  |  |  | 1 |  |  |  |  |  |  |  |  |  |  |  | 74 | 1 |  |  |  |  |  |  |  |  |  |  |  |  |  |  | 782 |  |  |  |  |  |  | 1 |  |  |  |  |  |  |  |  |  |  | 120 |
| CAA | 84 | 135 |  |  |  |  |  |  |  | 1 | 3 |  |  |  |  |  |  | 30 | 105 | 2,358 | 87 | 118 |  |  |  | 512 |  |  |  | 52 |  | 1 |  | 167 |  |  |  |  |  |  |  |  |  |  |  |  |  |  |  |  |  | 54 |  |  |  |  |  |  |  |  |  |  |  |  |  |  |  |
| CAC | 125 |  | 138 |  |  |  |  |  |  |  |  |  |  |  |  |  |  | 143 | 31 | 227 | 2,905 | 2 | 103 |  | 1 |  | 790 | 2 |  |  | 81 | 1 | 1 |  | 77 |  |  | 1 | 3 |  |  |  | 1 |  |  |  | 8 |  |  |  |  |  | 332 |  |  |  |  |  |  |  |  |  |  |  |  |  |  |
| CAG | 497 |  |  | 273 |  |  |  |  |  |  |  |  |  |  |  |  |  | 2,631 | 193 | 87 | 221 | 6 | 6 | 231 |  |  |  | 1,265 |  |  |  | 100 |  |  |  | 350 |  | 1 |  |  |  |  |  |  |  |  |  |  |  |  |  |  |  | 145 |  | 2 |  |  |  |  |  |  |  |  |  |  |  |
| CAT | 73 |  |  |  | 101 |  |  |  |  |  |  |  |  |  |  |  |  | 59 | 2,423 | 221 | 15 | 2 | 3 |  | 103 |  |  |  | 565 |  | 1 | 1 | 58 |  |  |  | 98 |  |  |  |  |  |  |  |  |  |  |  |  |  |  |  |  |  | 232 |  |  |  |  |  |  |  |  |  |  |  |  |
| CCA | 187 |  |  |  |  | 155 | 1 |  |  |  |  |  |  |  |  |  |  | 146 | 1 | 2 |  | 20 | 546 | 4,285 | 336 | 82 | 1 |  | 1 | 351 | 1 | 2 |  |  |  |  |  | 105 |  |  |  |  |  | 1 |  |  |  |  |  |  |  |  |  |  |  | 463 | 1 |  |  |  |  |  |  |  |  |  |  |
| CCC | 440 | 3 |  |  |  |  | 223 |  |  | 3 |  |  |  |  |  |  |  |  | 140 | 8 |  | 492 | 93 | 927 | 3,935 |  | 155 |  |  |  | 411 | 3 |  | 1 | 1 |  |  |  | 232 | 2 |  |  | 4 |  |  |  |  |  |  |  |  |  |  |  |  | 1 | 667 |  |  | 1 |  |  |  |  |  |  |  |
| CCG | 283 |  |  |  |  | 1 | 1 | 53 | 1 |  |  |  |  |  |  |  |  |  |  | 326 |  | 5,152 | 971 | 64 | 578 |  | 2 | 257 |  | 1 |  | 1,297 | 1 |  |  |  |  | 2 | 3 | 94 |  |  |  |  |  |  |  |  |  |  |  |  |  |  |  |  | 1 | 167 |  |  |  |  |  |  |  |  |  |
| CCT | 174 |  |  |  |  |  | 1 |  | 171 | 13 |  |  |  |  |  |  |  |  |  |  | 120 | 331 | 2,898 | 422 | 32 |  |  | 1 | 86 | 1 |  | 1 | 427 |  |  |  |  |  | 1 | 5 | 163 |  |  |  |  |  |  |  |  |  |  |  |  |  |  |  |  |  | 667 |  |  |  |  |  |  |  |  |
| CGA | 21 |  |  |  |  |  |  |  |  | 317 | 1 |  |  |  |  |  |  | 702 |  | 1 |  | 63 |  |  |  | 12 | 171 | 774 | 88 | 15 |  | 1 | 1 |  |  |  |  |  |  |  |  | 78 |  |  |  |  |  |  |  |  |  |  |  |  |  |  |  |  |  | 181 |  |  |  |  |  |  |  |
| CGC | 112 |  |  |  |  |  |  |  |  |  | 122 | 1 |  |  |  |  |  | 1 | 1,081 | 3 |  |  | 134 | 10 | 1 | 190 | 62 | 316 | 1,358 |  | 61 | 2 |  | 4 |  |  |  |  |  | 1 |  |  | 151 |  |  |  |  |  |  |  |  |  |  |  |  |  |  |  |  |  | 691 | 1 |  |  |  |  |  |
| CGG | 181 |  |  |  |  |  |  | 1 |  |  |  | 702 |  |  |  |  |  |  | 3 | 1,762 |  | 1 | 4 | 240 | 1 | 902 | 291 | 68 | 223 |  | 4 | 175 |  |  |  |  |  |  | 1 | 6 |  |  |  | 154 |  |  |  |  |  |  |  |  |  |  |  |  |  |  |  |  |  | 760 |  |  |  |  |  |
| CGT | 48 |  |  |  |  |  |  |  |  |  |  |  | 69 |  |  |  |  | 7 |  |  | 773 |  | 1 | 2 | 60 | 47 | 1,129 | 201 | 8 |  |  |  | 48 |  |  |  |  |  |  | 1 |  |  |  |  | 65 |  |  |  |  |  |  |  |  |  |  |  |  |  |  |  |  |  | 386 |  |  |  |  |
| CTA | 25 |  |  |  |  |  |  |  |  |  |  |  |  | 68 |  |  |  | 30 |  | 1 |  | 220 |  |  |  | 32 |  |  |  | 4 | 293 | 1,967 | 120 |  |  |  |  |  |  |  |  |  |  |  |  | 80 |  |  |  |  |  |  |  |  |  |  |  |  |  |  |  |  |  | 600 |  |  |  |
| CTC | 109 |  |  |  |  |  |  |  | 1 |  |  |  |  |  | 177 |  |  |  | 94 | 1 | 1 |  | 311 |  | 2 |  | 70 |  |  | 340 | 29 | 758 | 2,364 |  |  |  |  |  |  |  |  |  |  |  |  |  | 144 |  |  |  |  |  |  |  |  |  | 1 |  | 2 |  |  |  |  |  | 512 |  |  |
| CTG | 162 |  |  |  |  |  |  |  |  | 1 |  |  |  |  |  | 219 |  |  | 1 | 147 |  |  | 7 | 869 |  | 1 |  | 99 | 9 | 2,649 | 835 | 93 | 568 |  |  |  |  |  | 1 | 1 |  |  | 4 |  |  |  |  | 224 |  |  |  |  |  |  |  |  |  |  |  |  |  |  |  |  |  | 2,523 |  |
| CTT | 39 |  |  |  |  |  |  |  |  |  |  |  |  |  |  |  | 107 |  |  | 1 | 117 | 1 | 1 |  | 350 |  |  |  | 84 | 156 | 1,659 | 458 | 17 |  |  |  |  |  |  |  | 1 |  |  |  |  |  |  |  | 91 |  |  |  |  |  |  |  |  |  |  |  |  |  |  |  |  |  | 264 |
| GAA | 222 | 637 |  |  |  |  | 2 |  |  |  |  |  |  |  |  | 1 |  | 163 |  | 1 |  |  |  |  |  |  |  |  |  |  |  |  |  | 33 | 232 | 3,560 | 211 | 122 |  |  | 2 | 316 |  | 3 | 2 | 43 | 1 |  |  |  |  | 24 |  |  |  |  |  |  |  |  |  |  |  |  |  |  |  |
| GAC | 201 |  | 673 |  |  |  |  |  |  |  |  |  |  |  |  |  |  |  | 70 |  |  |  |  |  |  |  |  |  |  |  |  |  |  | 334 | 50 | 348 | 4,792 |  | 108 |  |  | 8 | 402 |  | 2 |  | 44 | 1 | 2 |  |  |  | 47 |  |  |  |  |  |  |  |  |  |  |  |  |  |  |
| GAG | 507 |  |  | 902 |  |  |  |  |  |  |  |  |  |  |  |  |  |  |  | 348 |  |  |  | 2 |  |  |  |  |  |  |  | 1 |  | 4,169 | 432 | 91 | 285 | 5 | 9 | 163 | 12 | 6 | 8 | 455 | 1 | 1 |  | 109 |  |  |  |  |  | 38 |  |  |  |  |  | 1 |  |  | 1 |  |  |  |  |
| GAT | 143 |  |  |  | 566 |  |  |  |  |  |  | 1 |  |  |  |  |  |  |  |  | 69 |  |  | 1 |  |  |  |  |  |  |  |  |  | 199 | 3,637 | 295 | 67 |  |  |  | 79 | 1 |  | 1 | 284 | 1 | 10 |  | 63 |  |  |  |  |  | 68 |  |  |  |  |  |  |  |  |  |  |  | 2 |
| GCA | 127 |  |  |  |  | 1,063 |  |  |  |  |  | 8 |  |  | 1 |  |  |  |  | 5 |  | 217 |  | 2 |  |  | 1 |  |  |  |  | 1 |  | 130 |  | 1 | 1 | 40 | 660 | 3,631 | 375 | 144 | 2 |  |  | 398 |  |  |  |  |  |  |  |  |  | 186 |  |  |  |  | 5 |  |  |  |  |  |  |
| GCC | 492 |  |  |  |  |  | 1,531 |  | 1 |  | 1 |  |  |  |  |  |  |  |  |  |  |  | 247 | 4 |  |  | 3 |  |  |  | 4 |  |  |  | 120 | 17 | 1 | 577 | 99 | 826 | 4,265 |  | 243 | 5 | 1 |  | 721 |  |  |  |  |  |  |  |  |  | 375 |  |  |  | 2 |  |  |  |  |  |  |
| GCG | 316 |  |  |  |  |  |  | 515 |  |  | 2 |  |  |  |  |  |  |  |  | 6 |  |  |  | 102 |  |  | 3 | 1 |  |  |  | 1 |  |  |  | 205 | 4 | 4,521 | 761 | 79 | 463 | 6 | 14 | 176 | 2 |  |  | 1,240 |  |  |  |  |  |  |  |  | 1 | 100 |  |  |  |  |  |  |  |  |  |
| GCT | 153 |  |  |  |  |  | 1 |  | 853 |  | 1 |  |  |  |  |  |  |  |  |  |  |  | 1 |  | 216 |  |  |  |  |  |  |  |  | 11 |  |  | 86 | 324 | 3,433 | 426 | 24 |  |  | 2 | 184 |  |  |  | 453 |  |  |  |  |  |  | 1 |  |  | 211 |  |  |  |  |  |  |  |  |
| GGA | 167 | 6 |  |  |  |  |  |  |  | 476 | 1 | 3 |  |  |  |  | 8 |  |  |  |  |  |  |  |  | 58 |  | 1 |  |  |  | 1 | 10 | 426 |  |  |  | 100 |  | 4 |  | 37 | 412 | 1,661 | 260 | 78 | 1 | 1 |  |  |  |  |  |  |  |  | 3 |  |  | 34 |  | 2 |  |  |  |  |  |
| GGC | 602 | 1 |  |  |  |  |  |  |  |  | 1,264 |  |  |  |  |  |  |  |  |  |  |  |  |  |  |  | 157 | 2 |  |  |  |  |  |  | 420 | 11 |  | 1 | 298 | 13 |  | 559 | 48 | 607 | 2,678 |  | 126 | 2 |  |  |  |  |  |  |  |  | 1 | 2 |  |  | 131 |  | 1 |  |  |  | 1 |
| GGG | 376 |  |  |  |  | 4 |  |  |  |  |  | 662 |  |  |  |  |  |  |  |  |  |  | 1 | 2 |  |  |  | 223 |  |  |  | 1 |  | 1 | 5 | 549 | 1 | 3 | 8 | 111 |  | 1,607 | 525 | 70 | 355 |  |  | 142 |  |  |  |  |  |  |  |  |  |  |  |  | 1 | 90 |  |  |  |  |  |
| GGT | 123 |  |  |  |  |  |  |  |  |  |  | 1 | 584 |  |  |  |  |  |  |  |  |  |  |  |  |  |  | 1 | 80 |  |  |  |  | 1 |  | 28 | 342 |  |  |  | 114 | 243 | 2,032 | 222 | 47 |  |  | 3 | 71 |  |  |  |  |  |  |  |  |  |  |  |  |  | 54 |  |  |  |  |
| GTA | 10 |  |  |  |  |  |  |  |  |  |  |  | 1 | 847 |  |  |  |  |  |  |  |  |  |  |  |  |  |  |  | 91 |  |  |  | 39 |  |  |  | 278 | 1 |  |  | 44 | 5 |  | 6 | 10 | 216 | 1,482 | 167 |  |  |  |  |  |  |  |  |  |  |  |  |  |  | 50 |  |  |  |
| GTC | 77 |  | 3 |  |  |  |  |  |  |  |  |  |  |  | 1,387 | 1 | 1 |  |  |  |  |  |  |  |  |  |  |  |  |  | 199 |  |  |  | 50 | 1 |  |  | 454 | 11 | 2 |  | 59 | 2 | 5 | 207 | 20 | 350 | 1,403 |  |  |  |  |  |  |  |  | 1 |  |  |  |  |  |  | 83 |  |  |
| GTG | 166 |  |  |  |  |  |  |  |  |  |  |  |  |  |  | 1,556 |  |  |  | 4 |  |  | 1 | 1 | 1 |  |  |  |  |  |  | 293 |  | 9 |  | 105 |  | 1 | 1 | 1,041 | 5 | 1 | 1 | 116 | 4 | 2,039 | 433 | 58 | 382 |  |  |  |  |  |  |  |  |  |  |  |  |  |  |  |  | 140 |  |
| GTT | 66 | 1 |  |  |  | 9 |  |  |  |  |  |  | 1 |  |  |  | 879 |  |  |  |  |  |  |  |  |  |  |  |  |  |  |  | 100 | 6 |  |  | 46 | 1 |  | 1 | 420 |  |  |  | 61 | 137 | 1,107 | 355 | 17 |  |  |  |  | 1 |  |  |  |  | 4 |  |  |  |  |  |  |  | 96 |
| NGC | 1 |  |  |  |  |  |  |  |  |  |  |  |  |  |  |  |  |  |  |  |  |  |  |  |  |  |  |  |  |  |  |  |  |  |  |  |  |  |  |  |  |  |  |  |  |  |  |  |  |  |  |  |  |  |  |  |  |  |  |  |  |  |  |  |  |  |  |
| NNN | 3 |  |  |  |  |  |  |  |  |  |  |  |  |  |  |  |  |  |  |  |  |  |  |  |  |  |  |  |  |  |  |  |  |  |  |  |  |  |  |  |  |  |  |  |  |  |  |  |  |  |  |  |  |  |  |  |  |  |  |  |  |  |  |  |  |  |  |
| TAA | 23 | 12 |  |  |  |  |  |  |  |  |  |  |  |  |  |  |  | 22 |  |  |  |  |  |  |  |  |  |  |  |  |  |  |  | 8 |  |  |  |  |  |  |  |  |  |  |  |  |  |  |  |  |  | 18 | 7 | 12 | 3 | 7 |  |  |  | 25 |  |  |  | 7 | 1 |  |  |
| TAC | 46 |  | 33 |  |  |  |  |  |  |  |  |  |  |  |  |  | 3 |  | 227 |  |  |  |  |  |  |  |  |  |  | 1 |  |  |  |  | 49 |  |  |  |  |  |  |  |  |  |  |  |  |  |  |  |  | 15 | 13 | 15 | 3,498 | 3 | 44 |  |  |  | 160 | 1 |  |  | 100 |  |  |
| TAG | 6 |  |  | 7 |  |  |  |  |  |  |  |  |  |  |  |  |  |  |  | 22 |  |  |  |  |  |  |  |  |  |  |  |  |  |  |  | 9 |  |  |  |  |  |  |  |  |  |  |  |  |  |  |  | 49 | 5 | 1 | 3 |  |  | 6 |  | 2 |  | 36 |  | 1 |  | 3 |  |
| TAT | 33 |  |  |  | 23 |  |  |  |  |  |  |  |  |  |  |  |  |  |  |  | 221 |  |  |  |  |  |  |  |  |  |  |  |  |  |  |  | 70 |  |  |  |  |  |  |  |  |  |  |  |  |  |  | 11 | 2,584 | 9 | 14 |  |  |  | 63 |  |  |  | 201 | 8 |  |  | 77 |
| TCA | 65 |  |  |  |  | 113 |  |  |  |  |  |  |  |  | 1 |  |  |  |  |  |  | 331 |  |  |  |  |  |  |  | 1 | 2 |  |  |  |  |  |  | 141 |  |  |  |  |  |  |  |  | 1 |  |  |  |  | 18 | 1 | 1 | 4 | 7 | 393 | 2,404 | 241 | 11 |  |  |  | 213 | 7 | 4 |  |
| TCC | 176 |  |  |  |  |  | 162 |  |  |  | 1 |  |  |  | 1 |  |  |  |  |  |  |  | 565 |  |  |  | 3 |  |  |  | 2 |  |  |  |  |  |  |  | 275 |  |  |  | 1 |  |  |  | 2 | 1 |  |  |  |  | 79 |  |  | 477 | 38 | 411 | 2,894 |  | 75 |  | 3 |  | 230 |  |  |
| TCG | 62 |  |  |  |  |  |  | 72 |  |  |  |  |  |  |  |  |  |  |  |  |  |  |  | 156 |  |  |  |  |  |  | 2 |  |  |  |  |  |  |  | 1 | 52 |  |  |  | 1 | 1 |  |  |  |  |  |  |  |  | 11 |  | 2,997 | 606 | 14 | 380 |  |  | 44 |  |  |  | 576 |  |
| TCT | 65 |  |  |  |  | 1 |  |  | 146 |  |  |  |  |  |  |  |  |  |  |  |  |  | 2 |  | 500 |  |  |  |  |  | 4 |  |  |  |  | 1 |  |  | 1 |  | 164 |  |  |  |  |  |  |  |  |  |  |  | 1 |  | 56 | 182 | 2,442 | 292 | 30 |  |  |  | 71 | 4 | 5 |  | 291 |
| TGA | 24 |  |  |  |  |  |  |  |  | 7 |  |  |  |  |  |  |  |  |  |  |  |  |  | 3 |  | 63 |  |  |  |  |  |  |  |  |  |  |  |  |  |  |  | 17 |  |  |  |  |  | 1 |  |  |  | 57 |  |  |  | 11 |  |  |  | 9 | 8 | 25 | 9 | 8 |  |  |  |
| TGC | 72 |  |  |  |  |  |  |  |  |  | 57 |  |  |  |  | 1 |  |  |  |  |  |  |  |  |  |  | 444 |  |  |  |  |  |  |  |  |  |  |  |  |  |  |  | 63 |  |  |  |  | 1 |  |  |  |  | 182 |  |  |  | 76 |  | 3 | 16 | 32 | 47 | 1,763 |  | 52 |  |  |
| TGG | 75 |  |  |  |  |  |  |  |  |  |  | 41 |  |  |  |  |  |  |  |  |  |  |  |  |  |  |  | 472 |  |  |  | 1 |  |  |  |  |  |  |  |  |  |  |  | 64 |  |  |  | 1 |  |  |  |  |  | 81 | 1 | 1 |  | 63 |  | 54 | 55 | 35 | 55 |  |  | 72 | 1 |
| TGT | 34 |  |  |  |  |  |  |  |  |  | 1 |  | 62 |  |  |  |  |  |  |  |  |  |  |  |  |  |  |  | 295 |  |  | 1 |  |  |  |  |  |  |  |  |  |  |  |  | 76 |  |  |  |  |  |  |  |  |  | 280 |  | 2 |  | 115 | 6 | 1,341 | 59 | 8 |  | 1 | 1 | 87 |
| TTA | 40 |  |  |  |  |  |  |  |  |  |  |  |  | 76 |  |  |  |  |  |  |  |  |  |  |  |  |  |  |  | 460 |  |  |  |  |  |  |  |  |  |  |  |  |  |  |  | 52 |  |  | 1 |  |  | 5 |  |  |  | 142 |  |  |  | 2 |  |  |  | 12 | 70 | 625 | 71 |
| TTC | 59 |  |  |  |  |  |  |  |  |  |  |  |  |  | 68 |  |  |  |  |  |  |  |  |  | 1 |  |  |  |  |  | 402 |  | 1 |  |  |  |  |  | 1 |  |  |  |  |  |  |  | 53 |  |  |  |  |  | 88 |  |  |  | 175 |  |  |  | 83 | 1 |  | 59 | 24 | 81 | 2,332 |
| TTG | 34 | 1 |  |  |  |  |  |  |  |  |  |  |  |  |  | 111 | 1 |  |  |  |  |  |  |  |  |  |  |  |  |  |  | 1,953 |  |  | 1 |  |  |  |  |  |  |  |  |  |  |  |  | 118 | 2 |  |  |  | 1 | 10 |  |  |  | 419 | 9 |  |  | 39 | 1 | 860 | 120 | 19 | 96 |
| TTT | 73 |  |  |  |  |  |  |  |  |  |  |  |  |  |  |  | 65 |  |  |  |  |  |  |  |  |  |  |  | 1 | 1 | 1 |  | 239 |  |  |  |  |  |  |  |  |  |  |  |  |  |  | 2 | 81 |  |  |  |  |  | 85 |  |  |  | 204 |  | 4 |  | 68 | 70 | 2,010 | 59 | 12 |


---


 **Amino acid changes**

How to read this table:   
- Rows are reference amino acids and columns are changed amino acids. E.g. Row 'A' column 'E' indicates how many 'A' amino acids have been replaced by 'E' amino acids.  
- Red background colors indicate that more changes happened (heat-map).  
- Diagonals are indicated using grey background color   
- WARNING: This table may include different translation codon tables (e.g. mamalian DNA and mitochondrial DNA).

|  | \* | - | ? | A | C | D | E | F | G | H | I | K | L | M | N | P | Q | R | S | T | V | W | Y |
| --- | --- | --- | --- | --- | --- | --- | --- | --- | --- | --- | --- | --- | --- | --- | --- | --- | --- | --- | --- | --- | --- | --- | --- |
| \* | 173 | 44 | 9 |  | 17 |  | 17 | 1 | 17 |  |  | 19 | 19 |  |  | 3 | 44 | 70 | 24 |  | 1 | 61 | 18 |
| - | 62 |  | 1,428 | 849 | 66 | 131 | 488 | 173 | 976 | 83 | 86 | 136 | 372 | 19 | 95 | 734 | 286 | 390 | 464 | 129 | 210 | 32 | 27 |
| ? |  | 4 | 1 |  |  |  |  |  |  |  |  |  |  |  |  |  |  |  |  |  |  |  |  |
| A |  | 1,088 |  | 20,504 | 7 | 212 | 364 |  | 779 |  | 1 |  | 6 |  |  | 789 | 11 | 16 | 878 | 3,964 | 2,812 |  |  |
| C | 22 | 106 |  |  | 3,144 |  |  | 140 | 139 |  |  |  | 2 | 1 |  |  |  | 739 | 316 |  | 1 | 106 | 462 |
| D |  | 344 |  | 187 |  | 8,546 | 1,176 | 2 | 698 | 139 |  |  |  |  | 1,239 | 1 |  | 1 |  |  | 121 |  | 115 |
| E | 63 | 729 |  | 313 | 1 | 1,160 | 7,853 |  | 791 |  |  | 1,539 | 1 | 1 |  | 2 | 512 |  |  | 2 | 154 |  |  |
| F |  | 132 |  | 1 | 155 |  |  | 4,378 |  |  | 133 |  | 913 |  |  | 1 |  | 1 | 379 |  | 136 | 1 | 173 |
| G | 34 | 1,267 | 1 | 652 | 187 | 768 | 1,016 | 1 | 11,363 |  | 8 | 7 | 12 |  |  | 3 |  | 1,664 | 1,855 | 4 | 424 | 92 |  |
| H |  | 198 |  | 4 |  | 175 |  |  | 1 | 5,374 |  |  | 143 |  | 239 | 214 | 650 | 1,357 |  |  | 8 |  | 564 |
| I |  | 101 |  | 4 |  | 3 | 2 | 182 |  | 2 | 6,324 | 53 | 344 | 594 | 100 |  |  | 57 | 141 | 984 | 2,433 |  | 1 |
| K | 29 | 249 |  |  |  |  | 1,110 |  |  |  | 44 | 5,170 |  | 65 | 503 |  | 373 | 1,253 |  | 229 |  |  |  |
| L | 17 | 409 |  | 3 | 1 | 1 |  | 1,133 | 4 | 213 | 429 | 1 | 19,362 | 330 |  | 1,761 | 180 | 296 | 573 | 1 | 712 | 39 | 1 |
| M |  | 85 |  |  |  | 6 |  | 1 |  | 1 | 882 | 113 | 525 | 41 | 5 | 2 |  | 67 | 10 | 1,134 | 1,052 |  | 2 |
| N | 1 | 128 |  |  |  | 811 | 7 |  |  | 218 | 91 | 407 |  |  | 6,607 |  |  | 3 | 1,526 | 234 | 1 |  | 122 |
| P | 1 | 1,084 |  | 607 |  | 1 | 1 |  | 5 | 261 |  | 3 | 2,496 |  |  | 21,082 | 482 | 601 | 1,967 | 607 |  |  |  |
| Q | 199 | 581 |  | 1 |  |  | 517 |  |  | 606 |  | 408 | 153 |  |  | 361 | 5,106 | 1,778 | 5 |  |  |  |  |
| R | 200 | 576 |  | 11 | 1,077 |  | 10 |  | 1,337 | 1,857 | 67 | 1,289 | 307 | 62 | 1 | 518 | 2,484 | 9,936 | 531 | 114 |  | 804 |  |
| S | 41 | 664 |  | 635 | 296 |  | 6 | 533 | 1,251 |  | 143 | 9 | 810 | 2 | 1,626 | 1,564 | 5 | 526 | 18,936 | 925 | 4 | 44 | 142 |
| T |  | 352 |  | 3,106 | 1 | 10 | 2 | 1 |  | 1 | 1,395 | 196 |  | 1,245 | 321 | 421 |  | 200 | 762 | 17,511 |  | 1 |  |
| V | 1 | 319 |  | 2,216 |  | 96 | 160 | 179 | 304 |  | 3,114 | 1 | 873 | 1,557 | 3 | 3 | 4 |  | 7 | 9 | 8,383 |  |  |
| W | 135 | 75 |  |  | 110 |  |  | 1 | 64 |  |  |  | 73 |  |  |  |  | 513 | 64 |  | 1 | 35 | 1 |
| Y | 50 | 79 |  |  | 361 | 119 |  | 177 |  | 448 | 3 |  | 9 |  | 56 |  |  |  | 110 |  |  | 1 | 6,109 |


---


 **Variants by chromosome**

```
		  

		NC_006583_3, Position,0,1000000,2000000,3000000,4000000,5000000,6000000,7000000,8000000,9000000,10000000,11000000,12000000,13000000,14000000,15000000,16000000,17000000,18000000,19000000,20000000,21000000,22000000,23000000,24000000,25000000,26000000,27000000,28000000,29000000,30000000,31000000,32000000,33000000,34000000,35000000,36000000,37000000,38000000,39000000,40000000,41000000,42000000,43000000,44000000,45000000,46000000,47000000,48000000,49000000,50000000,51000000,52000000,53000000,54000000,55000000,56000000,57000000,58000000,59000000,60000000,61000000,62000000,63000000,64000000,65000000,66000000,67000000,68000000,69000000,70000000,71000000,72000000,73000000,74000000,75000000,76000000,77000000,78000000,79000000,80000000,81000000,82000000,83000000,84000000,85000000,86000000,87000000,88000000,89000000,90000000,91000000,92000000,93000000,94000000,95000000,96000000,97000000,98000000,99000000,100000000,101000000,102000000,103000000,104000000,105000000,106000000,107000000,108000000,109000000,110000000,111000000,112000000,113000000,114000000,115000000,116000000,117000000,118000000,119000000,120000000,121000000,122000000
NC_006583_3,Count,5631,8164,7690,6967,6806,6861,7212,7391,7119,7266,7552,7296,7204,7386,7132,7084,7404,7674,7375,6909,6821,7200,7767,7592,7988,6607,6770,6959,6665,7064,6706,6643,6837,7051,6293,6579,6744,6585,6639,6799,6826,6621,6095,6858,6805,6613,6534,6699,6793,7339,6814,6077,7466,7630,8126,8708,7895,6733,6490,6629,6095,5803,7127,7499,7273,6253,7159,7397,7959,6582,6567,5941,6805,6501,6850,6394,6462,6045,6368,6148,6722,6748,7058,6792,6868,6699,7165,6610,6223,7223,6724,6416,6248,6513,7495,8122,8653,7944,7435,5993,5594,5432,6550,4156,4545,5700,6225,6299,6709,6385,6588,6215,6260,6570,6678,5981,6374,7080,7974,7973,8665,8146,5458

	
```

```
		  

		NC_006584_3, Position,0,1000000,2000000,3000000,4000000,5000000,6000000,7000000,8000000,9000000,10000000,11000000,12000000,13000000,14000000,15000000,16000000,17000000,18000000,19000000,20000000,21000000,22000000,23000000,24000000,25000000,26000000,27000000,28000000,29000000,30000000,31000000,32000000,33000000,34000000,35000000,36000000,37000000,38000000,39000000,40000000,41000000,42000000,43000000,44000000,45000000,46000000,47000000,48000000,49000000,50000000,51000000,52000000,53000000,54000000,55000000,56000000,57000000,58000000,59000000,60000000,61000000,62000000,63000000,64000000,65000000,66000000,67000000,68000000,69000000,70000000,71000000,72000000,73000000,74000000,75000000,76000000,77000000,78000000,79000000,80000000,81000000,82000000,83000000,84000000,85000000
NC_006584_3,Count,5475,6097,6385,6236,1374,314,3182,6214,6066,6504,6021,5906,6232,5906,5983,6390,6729,6209,4177,6183,6615,6421,6928,6580,7348,7363,7521,7183,7192,7071,7486,8417,8816,8863,7367,5626,6068,5862,6382,6873,6335,6758,6384,5673,6336,6593,5948,6177,6409,7050,6671,6140,6853,7093,6875,7313,8160,6098,6985,6907,7297,6935,6347,7165,7256,7044,7002,6403,6108,6260,8125,6468,6147,6327,7121,7288,7630,7135,7790,7899,9529,7391,9277,8524,8651,3229

	
```

```
		  

		NC_006585_3, Position,0,1000000,2000000,3000000,4000000,5000000,6000000,7000000,8000000,9000000,10000000,11000000,12000000,13000000,14000000,15000000,16000000,17000000,18000000,19000000,20000000,21000000,22000000,23000000,24000000,25000000,26000000,27000000,28000000,29000000,30000000,31000000,32000000,33000000,34000000,35000000,36000000,37000000,38000000,39000000,40000000,41000000,42000000,43000000,44000000,45000000,46000000,47000000,48000000,49000000,50000000,51000000,52000000,53000000,54000000,55000000,56000000,57000000,58000000,59000000,60000000,61000000,62000000,63000000,64000000,65000000,66000000,67000000,68000000,69000000,70000000,71000000,72000000,73000000,74000000,75000000,76000000,77000000,78000000,79000000,80000000,81000000,82000000,83000000,84000000,85000000,86000000,87000000,88000000,89000000,90000000,91000000
NC_006585_3,Count,5836,6445,7441,6248,6725,6445,7269,6983,7179,7307,7785,6935,7143,6686,6725,5693,6793,5993,5814,5609,6086,6594,6518,7022,6888,6445,6715,6585,6005,6084,6379,3991,6395,6766,6708,6085,6087,6464,6750,6815,7190,6151,6509,6897,6434,6686,7427,6646,7179,7012,7045,7450,6545,5973,6198,6683,7444,8318,7230,8703,8873,6130,7055,6411,7115,6812,6706,7294,7241,8163,7355,7347,5809,6539,7588,7240,7879,7904,7802,7961,7689,8045,7866,7774,7768,7579,7450,8489,8149,8358,8478,6739

	
```

```
		  

		NC_006586_3, Position,0,1000000,2000000,3000000,4000000,5000000,6000000,7000000,8000000,9000000,10000000,11000000,12000000,13000000,14000000,15000000,16000000,17000000,18000000,19000000,20000000,21000000,22000000,23000000,24000000,25000000,26000000,27000000,28000000,29000000,30000000,31000000,32000000,33000000,34000000,35000000,36000000,37000000,38000000,39000000,40000000,41000000,42000000,43000000,44000000,45000000,46000000,47000000,48000000,49000000,50000000,51000000,52000000,53000000,54000000,55000000,56000000,57000000,58000000,59000000,60000000,61000000,62000000,63000000,64000000,65000000,66000000,67000000,68000000,69000000,70000000,71000000,72000000,73000000,74000000,75000000,76000000,77000000,78000000,79000000,80000000,81000000,82000000,83000000,84000000,85000000,86000000,87000000,88000000
NC_006586_3,Count,6193,6518,5619,6253,5954,6633,6594,6876,6726,6828,6540,6634,6866,6668,6468,6502,6975,6984,6143,5983,7070,7881,7778,5715,5609,6099,6551,7342,8547,8141,6881,6930,7746,7638,7949,6609,5983,7172,6625,6220,6029,6717,6252,6239,6336,7010,7058,7046,6783,6767,6830,6443,6824,7252,6611,6664,6961,6864,6862,6849,7007,5833,6228,6805,6849,7016,6456,6484,6730,6613,6630,5642,7066,6908,6520,6492,6985,7955,8031,7764,7780,8022,8431,8517,8121,8535,8979,8595,2642

	
```

```
		  

		NC_006587_3, Position,0,1000000,2000000,3000000,4000000,5000000,6000000,7000000,8000000,9000000,10000000,11000000,12000000,13000000,14000000,15000000,16000000,17000000,18000000,19000000,20000000,21000000,22000000,23000000,24000000,25000000,26000000,27000000,28000000,29000000,30000000,31000000,32000000,33000000,34000000,35000000,36000000,37000000,38000000,39000000,40000000,41000000,42000000,43000000,44000000,45000000,46000000,47000000,48000000,49000000,50000000,51000000,52000000,53000000,54000000,55000000,56000000,57000000,58000000,59000000,60000000,61000000,62000000,63000000,64000000,65000000,66000000,67000000,68000000,69000000,70000000,71000000,72000000,73000000,74000000,75000000,76000000,77000000,78000000,79000000,80000000,81000000,82000000,83000000,84000000,85000000,86000000,87000000,88000000
NC_006587_3,Count,6945,7120,7754,7691,6962,7607,6749,7134,7313,7041,6887,7021,6821,7272,7156,6737,6682,7723,6996,7324,6691,6455,6915,6815,6717,6369,7062,7226,6898,7176,7733,7882,6364,6836,7073,7139,6707,7358,7291,6244,6312,6727,6716,6295,6457,6133,6368,6065,6019,6700,6999,6660,7040,7675,7241,6868,6606,7820,8494,7704,6321,6943,6787,6159,7443,7764,7837,7829,7929,6900,6913,6855,7167,7064,6458,6553,6098,6157,5521,6357,6244,5970,6670,8469,7965,8171,8502,8509,7589

	
```

```
		  

		NC_006588_3, Position,0,1000000,2000000,3000000,4000000,5000000,6000000,7000000,8000000,9000000,10000000,11000000,12000000,13000000,14000000,15000000,16000000,17000000,18000000,19000000,20000000,21000000,22000000,23000000,24000000,25000000,26000000,27000000,28000000,29000000,30000000,31000000,32000000,33000000,34000000,35000000,36000000,37000000,38000000,39000000,40000000,41000000,42000000,43000000,44000000,45000000,46000000,47000000,48000000,49000000,50000000,51000000,52000000,53000000,54000000,55000000,56000000,57000000,58000000,59000000,60000000,61000000,62000000,63000000,64000000,65000000,66000000,67000000,68000000,69000000,70000000,71000000,72000000,73000000,74000000,75000000,76000000,77000000
NC_006588_3,Count,4081,6463,7240,5857,7234,7377,7087,6833,6592,5778,6780,6172,6306,6452,7422,7446,7089,5836,6659,7482,7265,6556,6886,5813,5953,5986,6707,7196,6870,6622,7259,7605,7207,7624,7030,6046,6563,6328,7776,8626,6240,6329,6397,5779,6648,7057,7409,7037,7565,6665,7066,6696,6786,6798,7047,6185,6555,6371,6962,6420,6709,6615,6711,6858,7524,6646,7516,7642,7267,6902,7826,7717,7707,8150,7968,7996,8301,4713

	
```

```
		  

		NC_006589_3, Position,0,1000000,2000000,3000000,4000000,5000000,6000000,7000000,8000000,9000000,10000000,11000000,12000000,13000000,14000000,15000000,16000000,17000000,18000000,19000000,20000000,21000000,22000000,23000000,24000000,25000000,26000000,27000000,28000000,29000000,30000000,31000000,32000000,33000000,34000000,35000000,36000000,37000000,38000000,39000000,40000000,41000000,42000000,43000000,44000000,45000000,46000000,47000000,48000000,49000000,50000000,51000000,52000000,53000000,54000000,55000000,56000000,57000000,58000000,59000000,60000000,61000000,62000000,63000000,64000000,65000000,66000000,67000000,68000000,69000000,70000000,71000000,72000000,73000000,74000000,75000000,76000000,77000000,78000000,79000000,80000000
NC_006589_3,Count,7762,4463,6290,6157,7003,6321,7436,7388,6842,7056,6801,6373,6324,6584,6334,6360,6591,6878,6464,6403,6306,6602,6283,6313,6049,6077,6252,5918,6111,6921,6993,6414,6544,7135,6328,6462,6890,6371,6800,6876,6871,6420,6246,6623,7076,6173,6011,6079,6393,6486,6892,6866,6421,6089,6452,6660,6024,6985,7390,7153,7493,7111,6486,6810,6729,6659,6269,6777,6782,6413,7365,6795,6849,7270,7299,6762,7556,11432,6891,7702,7457

	
```

```
		  

		NC_006590_3, Position,0,1000000,2000000,3000000,4000000,5000000,6000000,7000000,8000000,9000000,10000000,11000000,12000000,13000000,14000000,15000000,16000000,17000000,18000000,19000000,20000000,21000000,22000000,23000000,24000000,25000000,26000000,27000000,28000000,29000000,30000000,31000000,32000000,33000000,34000000,35000000,36000000,37000000,38000000,39000000,40000000,41000000,42000000,43000000,44000000,45000000,46000000,47000000,48000000,49000000,50000000,51000000,52000000,53000000,54000000,55000000,56000000,57000000,58000000,59000000,60000000,61000000,62000000,63000000,64000000,65000000,66000000,67000000,68000000,69000000,70000000,71000000,72000000,73000000,74000000
NC_006590_3,Count,5971,7375,5938,6034,6209,6502,6170,6741,6377,6261,5937,6182,6194,6136,6241,6001,6786,6608,7033,7294,7242,6589,5956,6859,7365,6568,6301,6620,6383,6084,6051,6100,6675,6148,6833,6074,6093,6663,6418,6365,6039,5857,5938,6420,6173,6597,6274,5748,6250,7111,6557,6857,6731,6729,7558,7275,7448,7770,7799,6433,7011,7044,6533,8100,8384,7791,7957,7702,7786,7902,6961,7970,8810,4590,1707

	
```

```
		  

		NC_006591_3, Position,0,1000000,2000000,3000000,4000000,5000000,6000000,7000000,8000000,9000000,10000000,11000000,12000000,13000000,14000000,15000000,16000000,17000000,18000000,19000000,20000000,21000000,22000000,23000000,24000000,25000000,26000000,27000000,28000000,29000000,30000000,31000000,32000000,33000000,34000000,35000000,36000000,37000000,38000000,39000000,40000000,41000000,42000000,43000000,44000000,45000000,46000000,47000000,48000000,49000000,50000000,51000000,52000000,53000000,54000000,55000000,56000000,57000000,58000000,59000000,60000000,61000000
NC_006591_3,Count,8838,8409,8426,8294,6916,6842,8593,3740,4269,6219,5692,5943,5976,6651,6935,7112,5693,5978,4434,6011,5886,6222,5398,71325,5780,6296,6547,7292,7125,6954,6528,6632,6873,5615,5486,6090,6271,6300,7120,7504,6352,6022,6353,5388,6224,6590,5759,6848,7017,8171,8421,7550,6786,7294,7200,6765,6669,6282,6504,6264,7445,432

	
```

```
		  

		NC_006592_3, Position,0,1000000,2000000,3000000,4000000,5000000,6000000,7000000,8000000,9000000,10000000,11000000,12000000,13000000,14000000,15000000,16000000,17000000,18000000,19000000,20000000,21000000,22000000,23000000,24000000,25000000,26000000,27000000,28000000,29000000,30000000,31000000,32000000,33000000,34000000,35000000,36000000,37000000,38000000,39000000,40000000,41000000,42000000,43000000,44000000,45000000,46000000,47000000,48000000,49000000,50000000,51000000,52000000,53000000,54000000,55000000,56000000,57000000,58000000,59000000,60000000,61000000,62000000,63000000,64000000,65000000,66000000,67000000,68000000,69000000
NC_006592_3,Count,5657,5826,6775,6724,7107,6955,6893,6251,6946,7307,6492,7232,6381,6922,7606,7227,5733,9103,9573,8576,7813,8492,7628,6571,5806,6864,6505,7672,7328,7018,6430,6657,6679,7016,7445,6355,7375,7702,6979,6881,6995,6724,6205,6052,6552,6538,6263,6557,7051,6485,6553,7366,7270,7268,6656,7023,6840,6813,5776,6595,6146,6179,6527,6096,7225,7305,7736,8440,7427,2650

	
```

```
		  

		NC_006593_3, Position,0,1000000,2000000,3000000,4000000,5000000,6000000,7000000,8000000,9000000,10000000,11000000,12000000,13000000,14000000,15000000,16000000,17000000,18000000,19000000,20000000,21000000,22000000,23000000,24000000,25000000,26000000,27000000,28000000,29000000,30000000,31000000,32000000,33000000,34000000,35000000,36000000,37000000,38000000,39000000,40000000,41000000,42000000,43000000,44000000,45000000,46000000,47000000,48000000,49000000,50000000,51000000,52000000,53000000,54000000,55000000,56000000,57000000,58000000,59000000,60000000,61000000,62000000,63000000,64000000,65000000,66000000,67000000,68000000,69000000,70000000,71000000,72000000,73000000,74000000
NC_006593_3,Count,4720,5708,6219,6312,6244,6498,6911,6860,5577,1986,4150,2604,6168,6202,6534,6258,6370,6477,6142,5931,5992,6609,6083,6492,7260,6034,5811,5993,6816,6904,6992,7176,6840,6915,6181,6537,5733,5884,6215,6534,5957,6219,6394,7164,6669,6520,6754,6716,6917,6483,5796,5239,5718,6052,6523,7314,6589,6367,7241,7518,7363,6563,6757,7408,7007,7215,6366,6993,8736,7437,8647,8332,8372,8961,2910

	
```

```
		  

		NC_006594_3, Position,0,1000000,2000000,3000000,4000000,5000000,6000000,7000000,8000000,9000000,10000000,11000000,12000000,13000000,14000000,15000000,16000000,17000000,18000000,19000000,20000000,21000000,22000000,23000000,24000000,25000000,26000000,27000000,28000000,29000000,30000000,31000000,32000000,33000000,34000000,35000000,36000000,37000000,38000000,39000000,40000000,41000000,42000000,43000000,44000000,45000000,46000000,47000000,48000000,49000000,50000000,51000000,52000000,53000000,54000000,55000000,56000000,57000000,58000000,59000000,60000000,61000000,62000000,63000000,64000000,65000000,66000000,67000000,68000000,69000000,70000000,71000000,72000000
NC_006594_3,Count,5989,5642,5993,6082,5806,6379,6123,5976,6851,7264,6730,5661,6388,6080,6817,7059,7089,7237,6880,7013,6818,7027,6634,6215,5786,6677,6790,6967,7172,6769,7555,6197,6387,6450,5813,5768,6294,6126,6648,6059,5989,6288,6126,6163,6401,6974,6549,6022,6125,6272,6906,7383,7307,7284,7068,6299,6396,6504,6679,6783,7129,7454,7198,7228,6726,6862,6755,6653,8115,8274,8722,8789,4031

	
```

```
		  

		NC_006595_3, Position,0,1000000,2000000,3000000,4000000,5000000,6000000,7000000,8000000,9000000,10000000,11000000,12000000,13000000,14000000,15000000,16000000,17000000,18000000,19000000,20000000,21000000,22000000,23000000,24000000,25000000,26000000,27000000,28000000,29000000,30000000,31000000,32000000,33000000,34000000,35000000,36000000,37000000,38000000,39000000,40000000,41000000,42000000,43000000,44000000,45000000,46000000,47000000,48000000,49000000,50000000,51000000,52000000,53000000,54000000,55000000,56000000,57000000,58000000,59000000,60000000,61000000,62000000,63000000
NC_006595_3,Count,5871,5794,5990,5987,6230,6310,6479,6731,6195,6210,6440,7246,6878,6829,7280,6680,7053,7093,6425,7107,7267,7036,7156,7123,7028,7347,6092,7546,7007,7913,7785,7526,7344,8191,8669,8351,7674,8306,7838,8294,6866,7893,7127,7245,7195,7720,7397,6685,6406,6924,7288,7447,7642,7605,7613,7830,7556,7327,6097,6710,6834,7650,7738,1065

	
```

```
		  

		NC_006596_3, Position,0,1000000,2000000,3000000,4000000,5000000,6000000,7000000,8000000,9000000,10000000,11000000,12000000,13000000,14000000,15000000,16000000,17000000,18000000,19000000,20000000,21000000,22000000,23000000,24000000,25000000,26000000,27000000,28000000,29000000,30000000,31000000,32000000,33000000,34000000,35000000,36000000,37000000,38000000,39000000,40000000,41000000,42000000,43000000,44000000,45000000,46000000,47000000,48000000,49000000,50000000,51000000,52000000,53000000,54000000,55000000,56000000,57000000,58000000,59000000,60000000
NC_006596_3,Count,4037,6026,6208,6304,6372,6413,5879,5624,6065,6235,6629,6388,6924,6485,6773,7189,6393,6035,6104,6481,6116,6153,6835,6045,6910,6794,6559,7167,6988,7016,6804,6901,6190,6548,6006,6787,6495,6232,7021,7011,6039,6564,7062,7237,6766,6115,7131,7468,7158,6892,6845,6786,6760,6567,7080,7216,8136,7837,7888,8264,7911

	
```

```
		  

		NC_006597_3, Position,0,1000000,2000000,3000000,4000000,5000000,6000000,7000000,8000000,9000000,10000000,11000000,12000000,13000000,14000000,15000000,16000000,17000000,18000000,19000000,20000000,21000000,22000000,23000000,24000000,25000000,26000000,27000000,28000000,29000000,30000000,31000000,32000000,33000000,34000000,35000000,36000000,37000000,38000000,39000000,40000000,41000000,42000000,43000000,44000000,45000000,46000000,47000000,48000000,49000000,50000000,51000000,52000000,53000000,54000000,55000000,56000000,57000000,58000000,59000000,60000000,61000000,62000000,63000000,64000000
NC_006597_3,Count,5720,6598,6158,5657,6880,8519,6315,8771,6753,6250,5701,6190,7799,6805,5601,5829,6494,6116,6691,6899,5640,6542,6914,7158,6965,6602,6601,6659,7225,6335,6519,6974,6831,6440,6468,6343,6256,6491,6339,6390,6492,6329,7348,6526,6484,6861,6822,7361,6321,7128,6640,6729,7473,7910,7706,7536,7119,8041,7931,7985,7324,7922,8610,8856,1492

	
```

```
		  

		NC_006598_3, Position,0,1000000,2000000,3000000,4000000,5000000,6000000,7000000,8000000,9000000,10000000,11000000,12000000,13000000,14000000,15000000,16000000,17000000,18000000,19000000,20000000,21000000,22000000,23000000,24000000,25000000,26000000,27000000,28000000,29000000,30000000,31000000,32000000,33000000,34000000,35000000,36000000,37000000,38000000,39000000,40000000,41000000,42000000,43000000,44000000,45000000,46000000,47000000,48000000,49000000,50000000,51000000,52000000,53000000,54000000,55000000,56000000,57000000,58000000,59000000
NC_006598_3,Count,8655,5032,7422,7429,7212,6438,6659,6786,5918,5899,4212,6475,6437,4603,6064,6657,6794,7648,8047,7155,8411,6663,6773,6757,7045,6824,5774,6758,7339,7112,7251,6964,6813,6470,6571,7247,6860,7034,6822,6603,6152,6977,7414,7501,7356,7327,7098,7225,7478,7861,6550,7595,7801,7982,6849,9040,8709,8720,8303,483

	
```

```
		  

		NC_006599_3, Position,0,1000000,2000000,3000000,4000000,5000000,6000000,7000000,8000000,9000000,10000000,11000000,12000000,13000000,14000000,15000000,16000000,17000000,18000000,19000000,20000000,21000000,22000000,23000000,24000000,25000000,26000000,27000000,28000000,29000000,30000000,31000000,32000000,33000000,34000000,35000000,36000000,37000000,38000000,39000000,40000000,41000000,42000000,43000000,44000000,45000000,46000000,47000000,48000000,49000000,50000000,51000000,52000000,53000000,54000000,55000000,56000000,57000000,58000000,59000000,60000000,61000000,62000000,63000000,64000000
NC_006599_3,Count,8330,9328,8036,7916,7652,7991,6910,7963,7500,7460,7488,7954,6868,6738,6766,7148,6703,6959,6516,6232,6697,4729,6606,7178,7136,6259,6862,7209,6553,6164,6508,6445,7002,6515,6692,7116,6848,6045,6228,6657,6898,7209,7528,7205,7430,7245,7448,7209,6736,6493,5981,6708,6414,7435,6882,6342,5571,5723,5915,5972,6321,7068,6975,7458,1686

	
```

```
		  

		NC_006600_3, Position,0,1000000,2000000,3000000,4000000,5000000,6000000,7000000,8000000,9000000,10000000,11000000,12000000,13000000,14000000,15000000,16000000,17000000,18000000,19000000,20000000,21000000,22000000,23000000,24000000,25000000,26000000,27000000,28000000,29000000,30000000,31000000,32000000,33000000,34000000,35000000,36000000,37000000,38000000,39000000,40000000,41000000,42000000,43000000,44000000,45000000,46000000,47000000,48000000,49000000,50000000,51000000,52000000,53000000,54000000,55000000
NC_006600_3,Count,7479,8347,8140,7696,7266,6904,6429,7597,6922,6403,6718,6570,7055,6386,6997,6540,6093,6482,3946,7077,7255,7017,6739,6649,6651,7430,7384,8496,8042,7642,7853,7706,7897,7817,6755,7012,6996,6554,7365,7028,4645,6498,6249,6755,8404,7086,8430,8799,8643,6958,6105,6281,7046,6707,6717,6089

	
```

```
		  

		NC_006601_3, Position,0,1000000,2000000,3000000,4000000,5000000,6000000,7000000,8000000,9000000,10000000,11000000,12000000,13000000,14000000,15000000,16000000,17000000,18000000,19000000,20000000,21000000,22000000,23000000,24000000,25000000,26000000,27000000,28000000,29000000,30000000,31000000,32000000,33000000,34000000,35000000,36000000,37000000,38000000,39000000,40000000,41000000,42000000,43000000,44000000,45000000,46000000,47000000,48000000,49000000,50000000,51000000,52000000,53000000
NC_006601_3,Count,6956,7216,6770,7062,6801,7286,7110,7355,7094,7054,6975,6387,5745,6147,7207,7093,6381,5830,6204,5378,6118,6504,6361,6704,7508,7540,7247,7479,7497,7869,6740,7198,7252,7730,7228,7115,7361,7210,6339,7469,7131,7251,7538,7918,7216,6869,6680,7539,7540,7128,7606,8079,7567,5815

	
```

```
		  

		NC_006602_3, Position,0,1000000,2000000,3000000,4000000,5000000,6000000,7000000,8000000,9000000,10000000,11000000,12000000,13000000,14000000,15000000,16000000,17000000,18000000,19000000,20000000,21000000,22000000,23000000,24000000,25000000,26000000,27000000,28000000,29000000,30000000,31000000,32000000,33000000,34000000,35000000,36000000,37000000,38000000,39000000,40000000,41000000,42000000,43000000,44000000,45000000,46000000,47000000,48000000,49000000,50000000,51000000,52000000,53000000,54000000,55000000,56000000,57000000,58000000
NC_006602_3,Count,5898,6723,5909,6835,6759,6170,6347,6452,6243,7111,7609,7291,6595,6457,6195,7194,7569,7009,7373,6880,6354,6551,6929,7258,7206,7053,6756,6906,6870,6964,7359,6928,6736,6374,5478,6830,6895,5902,5557,5756,5782,5880,7106,6456,6161,6651,6500,5741,6473,6058,6513,6728,7508,6713,7900,8243,8295,8987,1193

	
```

```
		  

		NC_006603_3, Position,0,1000000,2000000,3000000,4000000,5000000,6000000,7000000,8000000,9000000,10000000,11000000,12000000,13000000,14000000,15000000,16000000,17000000,18000000,19000000,20000000,21000000,22000000,23000000,24000000,25000000,26000000,27000000,28000000,29000000,30000000,31000000,32000000,33000000,34000000,35000000,36000000,37000000,38000000,39000000,40000000,41000000,42000000,43000000,44000000,45000000,46000000,47000000,48000000,49000000,50000000
NC_006603_3,Count,5864,6437,6887,6694,6885,6223,7357,7031,6541,7344,6961,7371,7067,6163,6315,7217,6829,6929,7354,7388,6445,6683,6543,6680,5928,5999,5986,6963,6683,6071,5993,7218,6171,5562,7440,7798,7154,6538,6815,5902,6979,7968,7566,7964,8188,8418,8877,8768,7594,8595,6518

	
```

```
		  

		NC_006604_3, Position,0,1000000,2000000,3000000,4000000,5000000,6000000,7000000,8000000,9000000,10000000,11000000,12000000,13000000,14000000,15000000,16000000,17000000,18000000,19000000,20000000,21000000,22000000,23000000,24000000,25000000,26000000,27000000,28000000,29000000,30000000,31000000,32000000,33000000,34000000,35000000,36000000,37000000,38000000,39000000,40000000,41000000,42000000,43000000,44000000,45000000,46000000,47000000,48000000,49000000,50000000,51000000,52000000,53000000,54000000,55000000,56000000,57000000,58000000,59000000,60000000,61000000
NC_006604_3,Count,7103,6800,6596,6185,6665,6503,6456,6211,6144,6823,6950,7112,7054,6999,7080,6569,5996,6788,7013,6725,7051,6815,6468,6471,6774,6666,6539,6479,5894,6332,6352,6921,6539,6957,7288,6991,7441,7342,7113,7350,7568,7167,6440,6969,7153,7016,6300,7044,6818,5858,6458,6854,6825,7557,7731,7756,7779,7815,8058,7844,8698,3100

	
```

```
		  

		NC_006605_3, Position,0,1000000,2000000,3000000,4000000,5000000,6000000,7000000,8000000,9000000,10000000,11000000,12000000,13000000,14000000,15000000,16000000,17000000,18000000,19000000,20000000,21000000,22000000,23000000,24000000,25000000,26000000,27000000,28000000,29000000,30000000,31000000,32000000,33000000,34000000,35000000,36000000,37000000,38000000,39000000,40000000,41000000,42000000,43000000,44000000,45000000,46000000,47000000,48000000,49000000,50000000,51000000,52000000
NC_006605_3,Count,6302,5934,5742,5878,6279,6511,6268,6248,6586,6888,6603,7154,7269,7251,6825,6153,6434,7371,6586,6424,6231,7088,6799,6416,6518,5968,6205,6352,6818,6562,6227,7103,6157,6137,6430,7009,7213,6451,6734,7065,7583,7223,6679,7111,7288,7282,7627,7953,7818,7814,7704,7071,2360

	
```

```
		  

		NC_006606_3, Position,0,1000000,2000000,3000000,4000000,5000000,6000000,7000000,8000000,9000000,10000000,11000000,12000000,13000000,14000000,15000000,16000000,17000000,18000000,19000000,20000000,21000000,22000000,23000000,24000000,25000000,26000000,27000000,28000000,29000000,30000000,31000000,32000000,33000000,34000000,35000000,36000000,37000000,38000000,39000000,40000000,41000000,42000000,43000000,44000000,45000000,46000000,47000000
NC_006606_3,Count,6252,6053,6181,6145,6343,6957,7135,6933,5862,6695,6693,6221,6628,6907,7138,6583,6542,6491,6874,5698,5939,5939,6407,5579,5793,6242,7016,7556,7352,6668,6950,6669,6361,6843,7154,7084,7058,7776,7201,7364,7596,8257,9048,7229,8739,9497,9365,5967

	
```

```
		  

		NC_006607_3, Position,0,1000000,2000000,3000000,4000000,5000000,6000000,7000000,8000000,9000000,10000000,11000000,12000000,13000000,14000000,15000000,16000000,17000000,18000000,19000000,20000000,21000000,22000000,23000000,24000000,25000000,26000000,27000000,28000000,29000000,30000000,31000000,32000000,33000000,34000000,35000000,36000000,37000000,38000000,39000000,40000000,41000000,42000000,43000000,44000000,45000000,46000000,47000000,48000000,49000000,50000000,51000000
NC_006607_3,Count,5778,6275,6923,5743,6648,6260,6588,6280,6399,6694,6565,6259,7063,6263,7279,7058,6215,6447,5822,5910,6098,6986,7334,7142,6478,7045,7361,6727,6027,6490,7550,6920,7182,7006,6511,6979,7488,7014,7502,7346,7538,7796,7265,6536,7164,8279,9116,8871,8705,9389,4557,5284

	
```

```
		  

		NC_006608_3, Position,0,1000000,2000000,3000000,4000000,5000000,6000000,7000000,8000000,9000000,10000000,11000000,12000000,13000000,14000000,15000000,16000000,17000000,18000000,19000000,20000000,21000000,22000000,23000000,24000000,25000000,26000000,27000000,28000000,29000000,30000000,31000000,32000000,33000000,34000000,35000000,36000000,37000000,38000000
NC_006608_3,Count,6703,6776,7720,7367,7212,6860,6138,6301,6139,5657,7258,7886,7225,7080,6644,6538,5936,6908,6802,7343,8042,6190,6096,6185,6084,1188,3390,5617,5614,7434,6594,6260,8588,8517,8608,8447,8828,8318,8744

	
```

```
		  

		NC_006609_3, Position,0,1000000,2000000,3000000,4000000,5000000,6000000,7000000,8000000,9000000,10000000,11000000,12000000,13000000,14000000,15000000,16000000,17000000,18000000,19000000,20000000,21000000,22000000,23000000,24000000,25000000,26000000,27000000,28000000,29000000,30000000,31000000,32000000,33000000,34000000,35000000,36000000,37000000,38000000,39000000,40000000,41000000,42000000,43000000,44000000,45000000
NC_006609_3,Count,7723,7021,8511,6619,6340,6251,6978,7349,6636,7570,7124,6893,7225,6441,6955,7256,6368,6448,6890,6359,6885,6140,6530,6262,6307,6542,6814,6426,6684,7130,6916,7025,6709,6546,6651,6644,6718,6176,6074,7019,6145,6183,6081,5865,5891,4985

	
```

```
		  

		NC_006610_3, Position,0,1000000,2000000,3000000,4000000,5000000,6000000,7000000,8000000,9000000,10000000,11000000,12000000,13000000,14000000,15000000,16000000,17000000,18000000,19000000,20000000,21000000,22000000,23000000,24000000,25000000,26000000,27000000,28000000,29000000,30000000,31000000,32000000,33000000,34000000,35000000,36000000,37000000,38000000,39000000,40000000,41000000
NC_006610_3,Count,6229,6324,6877,6342,6094,6280,5640,6229,6164,6112,5903,6448,6297,6123,5480,5866,7053,7190,7427,7231,7518,6841,6604,6721,7038,6990,6746,7434,7311,6949,7697,7835,7226,8541,7483,8050,8415,8680,8563,9171,9344,1274

	
```

```
		  

		NC_006611_3, Position,0,1000000,2000000,3000000,4000000,5000000,6000000,7000000,8000000,9000000,10000000,11000000,12000000,13000000,14000000,15000000,16000000,17000000,18000000,19000000,20000000,21000000,22000000,23000000,24000000,25000000,26000000,27000000,28000000,29000000,30000000,31000000,32000000,33000000,34000000,35000000,36000000,37000000,38000000,39000000,40000000,41000000
NC_006611_3,Count,5792,6677,6734,6644,6417,6375,6441,6968,6964,6769,7107,6679,7344,6898,6406,6467,6007,6836,6930,6061,6669,6860,6396,6978,6887,6979,6877,6599,6899,6746,7404,7196,7001,7306,7744,7397,7760,7608,8097,7396,8473,6719

	
```

```
		  

		NC_006612_3, Position,0,1000000,2000000,3000000,4000000,5000000,6000000,7000000,8000000,9000000,10000000,11000000,12000000,13000000,14000000,15000000,16000000,17000000,18000000,19000000,20000000,21000000,22000000,23000000,24000000,25000000,26000000,27000000,28000000,29000000,30000000,31000000,32000000,33000000,34000000,35000000,36000000,37000000,38000000,39000000,40000000
NC_006612_3,Count,5577,5821,5906,5898,5679,6073,6097,6114,5845,6152,5838,6866,7263,6730,6230,6196,6274,6592,5645,6063,6296,5928,6576,7031,6762,6232,6752,7348,6198,6756,7473,7109,7519,8454,7714,6968,7281,8263,7752,6801,1350

	
```

```
		  

		NC_006613_3, Position,0,1000000,2000000,3000000,4000000,5000000,6000000,7000000,8000000,9000000,10000000,11000000,12000000,13000000,14000000,15000000,16000000,17000000,18000000,19000000,20000000,21000000,22000000,23000000,24000000,25000000,26000000,27000000,28000000,29000000,30000000,31000000,32000000,33000000,34000000,35000000,36000000,37000000,38000000,39000000
NC_006613_3,Count,6167,6606,6125,6350,6775,6782,7002,7192,7062,6924,7069,6521,6701,6506,7440,7595,7579,6907,7588,7733,7904,7318,7511,7058,7550,7507,7431,5584,64,3106,7525,7315,6799,7385,7827,8238,9262,8880,8574,7413

	
```

```
		  

		NC_006614_3, Position,0,1000000,2000000,3000000,4000000,5000000,6000000,7000000,8000000,9000000,10000000,11000000,12000000,13000000,14000000,15000000,16000000,17000000,18000000,19000000,20000000,21000000,22000000,23000000,24000000,25000000,26000000,27000000,28000000,29000000,30000000,31000000,32000000,33000000,34000000,35000000,36000000,37000000,38000000
NC_006614_3,Count,6750,7066,7180,7167,6628,6820,6624,7084,6996,7403,7123,6682,6837,7286,7219,7186,7127,6676,7062,7308,6684,6598,6771,7014,6832,7110,6373,6920,6354,6574,6563,6532,6033,6094,6306,6674,6499,5940,4283

	
```

```
		  

		NC_006615_3, Position,0,1000000,2000000,3000000,4000000,5000000,6000000,7000000,8000000,9000000,10000000,11000000,12000000,13000000,14000000,15000000,16000000,17000000,18000000,19000000,20000000,21000000,22000000,23000000,24000000,25000000,26000000,27000000,28000000,29000000,30000000,31000000
NC_006615_3,Count,6447,6597,7099,6886,6670,7355,6042,6941,7002,7449,7522,6444,6190,6763,7025,7036,7186,6843,6445,6755,7244,7774,7081,6410,5946,6457,8048,8023,6903,7491,7829,3060

	
```

```
		  

		NC_006616_3, Position,0,1000000,2000000,3000000,4000000,5000000,6000000,7000000,8000000,9000000,10000000,11000000,12000000,13000000,14000000,15000000,16000000,17000000,18000000,19000000,20000000,21000000,22000000,23000000,24000000,25000000,26000000,27000000,28000000,29000000,30000000,31000000,32000000,33000000,34000000,35000000,36000000,37000000,38000000,39000000,40000000,41000000,42000000
NC_006616_3,Count,5589,6491,6574,6478,7027,6992,7415,8156,7814,8433,8577,8267,5117,6480,5886,6821,5852,6155,6371,7391,6796,6945,7607,7329,7460,7300,6707,7085,7467,7645,7625,7660,7619,7082,6428,7423,8070,7360,8053,8494,8504,8875,805

	
```

```
		  

		NC_006617_3, Position,0,100000,200000,300000,400000,500000,600000,700000,800000,900000,1000000,1100000,1200000,1300000,1400000,1500000,1600000,1700000,1800000,1900000,2000000,2100000,2200000,2300000,2400000,2500000,2600000,2700000,2800000,2900000,3000000,3100000,3200000,3300000,3400000,3500000,3600000,3700000,3800000,3900000,4000000,4100000,4200000,4300000,4400000,4500000,4600000,4700000,4800000,4900000,5000000,5100000,5200000,5300000,5400000,5500000,5600000,5700000,5800000,5900000,6000000,6100000,6200000,6300000,6400000,6500000,6600000,6700000,6800000,6900000,7000000,7100000,7200000,7300000,7400000,7500000,7600000,7700000,7800000,7900000,8000000,8100000,8200000,8300000,8400000,8500000,8600000,8700000,8800000,8900000,9000000,9100000,9200000,9300000,9400000,9500000,9600000,9700000,9800000,9900000,10000000,10100000,10200000,10300000,10400000,10500000,10600000,10700000,10800000,10900000,11000000,11100000,11200000,11300000,11400000,11500000,11600000,11700000,11800000,11900000,12000000,12100000,12200000,12300000,12400000,12500000,12600000,12700000,12800000,12900000,13000000,13100000,13200000,13300000,13400000,13500000,13600000,13700000,13800000,13900000,14000000,14100000,14200000,14300000,14400000,14500000,14600000,14700000,14800000,14900000,15000000,15100000,15200000,15300000,15400000,15500000,15600000,15700000,15800000,15900000,16000000,16100000,16200000,16300000,16400000,16500000,16600000,16700000,16800000,16900000,17000000,17100000,17200000,17300000,17400000,17500000,17600000,17700000,17800000,17900000,18000000,18100000,18200000,18300000,18400000,18500000,18600000,18700000,18800000,18900000,19000000,19100000,19200000,19300000,19400000,19500000,19600000,19700000,19800000,19900000,20000000,20100000,20200000,20300000,20400000,20500000,20600000,20700000,20800000,20900000,21000000,21100000,21200000,21300000,21400000,21500000,21600000,21700000,21800000,21900000,22000000,22100000,22200000,22300000,22400000,22500000,22600000,22700000,22800000,22900000,23000000,23100000,23200000,23300000,23400000,23500000,23600000,23700000,23800000,23900000,24000000,24100000,24200000,24300000,24400000,24500000,24600000,24700000,24800000,24900000,25000000,25100000,25200000,25300000,25400000,25500000,25600000,25700000,25800000,25900000,26000000,26100000,26200000,26300000,26400000,26500000
NC_006617_3,Count,415,619,573,563,525,594,736,851,791,697,719,720,699,804,776,847,783,818,763,714,728,872,720,674,628,637,716,699,850,772,726,761,792,740,724,973,904,865,798,850,925,894,738,754,706,690,735,732,719,748,796,734,773,812,705,816,693,749,801,797,788,576,763,698,687,652,683,774,807,769,727,662,755,826,815,719,704,722,890,692,646,697,755,701,804,754,704,760,743,731,741,709,697,755,751,644,810,815,812,763,733,717,777,789,767,669,786,807,713,799,810,661,800,733,761,660,664,757,833,752,713,756,701,809,735,765,743,752,810,764,724,710,703,722,757,841,763,779,744,809,720,787,728,712,607,688,765,850,672,758,747,726,739,736,733,726,633,666,670,728,750,741,651,736,693,664,661,683,671,764,781,700,660,689,655,648,729,681,760,737,727,761,790,670,743,756,689,734,729,755,677,698,737,719,679,655,729,691,760,738,714,698,729,711,719,744,762,762,847,717,694,762,755,814,767,830,760,730,715,693,663,709,757,746,727,755,698,661,707,731,723,580,683,727,751,751,642,696,631,624,655,606,709,730,656,707,753,652,755,734,704,595,748,660,624,630,701,731,677,721,434,437,719,699,137,159

	
```

```
		  

		NC_006618_3, Position,0,1000000,2000000,3000000,4000000,5000000,6000000,7000000,8000000,9000000,10000000,11000000,12000000,13000000,14000000,15000000,16000000,17000000,18000000,19000000,20000000,21000000,22000000,23000000,24000000,25000000,26000000,27000000,28000000,29000000,30000000
NC_006618_3,Count,5894,6598,6531,6823,6875,6873,6446,6627,6820,7071,6717,7415,7244,7195,6067,6026,6609,6985,6555,6544,6141,6096,6699,7319,7565,6702,7580,7939,7801,7458,5931

	
```

```
		  

		NC_006619_3, Position,0,1000000,2000000,3000000,4000000,5000000,6000000,7000000,8000000,9000000,10000000,11000000,12000000,13000000,14000000,15000000,16000000,17000000,18000000,19000000,20000000,21000000,22000000,23000000,24000000,25000000,26000000,27000000,28000000,29000000,30000000
NC_006619_3,Count,5629,7563,7761,7666,7349,7685,6923,7143,7056,6996,6509,6045,6266,7194,6370,6762,6781,7150,7475,7859,6717,7638,7675,8285,7735,6510,7529,7742,7833,7549,8550

	
```

```
		  

		NC_006620_3, Position,0,100000,200000,300000,400000,500000,600000,700000,800000,900000,1000000,1100000,1200000,1300000,1400000,1500000,1600000,1700000,1800000,1900000,2000000,2100000,2200000,2300000,2400000,2500000,2600000,2700000,2800000,2900000,3000000,3100000,3200000,3300000,3400000,3500000,3600000,3700000,3800000,3900000,4000000,4100000,4200000,4300000,4400000,4500000,4600000,4700000,4800000,4900000,5000000,5100000,5200000,5300000,5400000,5500000,5600000,5700000,5800000,5900000,6000000,6100000,6200000,6300000,6400000,6500000,6600000,6700000,6800000,6900000,7000000,7100000,7200000,7300000,7400000,7500000,7600000,7700000,7800000,7900000,8000000,8100000,8200000,8300000,8400000,8500000,8600000,8700000,8800000,8900000,9000000,9100000,9200000,9300000,9400000,9500000,9600000,9700000,9800000,9900000,10000000,10100000,10200000,10300000,10400000,10500000,10600000,10700000,10800000,10900000,11000000,11100000,11200000,11300000,11400000,11500000,11600000,11700000,11800000,11900000,12000000,12100000,12200000,12300000,12400000,12500000,12600000,12700000,12800000,12900000,13000000,13100000,13200000,13300000,13400000,13500000,13600000,13700000,13800000,13900000,14000000,14100000,14200000,14300000,14400000,14500000,14600000,14700000,14800000,14900000,15000000,15100000,15200000,15300000,15400000,15500000,15600000,15700000,15800000,15900000,16000000,16100000,16200000,16300000,16400000,16500000,16600000,16700000,16800000,16900000,17000000,17100000,17200000,17300000,17400000,17500000,17600000,17700000,17800000,17900000,18000000,18100000,18200000,18300000,18400000,18500000,18600000,18700000,18800000,18900000,19000000,19100000,19200000,19300000,19400000,19500000,19600000,19700000,19800000,19900000,20000000,20100000,20200000,20300000,20400000,20500000,20600000,20700000,20800000,20900000,21000000,21100000,21200000,21300000,21400000,21500000,21600000,21700000,21800000,21900000,22000000,22100000,22200000,22300000,22400000,22500000,22600000,22700000,22800000,22900000,23000000,23100000,23200000,23300000,23400000,23500000,23600000,23700000,23800000,23900000
NC_006620_3,Count,563,663,667,669,618,560,671,771,708,703,668,799,774,744,688,789,631,719,702,774,771,660,683,814,706,653,738,750,753,645,693,702,681,734,735,752,764,842,930,766,718,809,741,789,814,853,738,796,862,805,739,792,751,761,760,785,755,691,583,748,835,778,824,843,814,792,752,750,789,765,745,686,688,834,740,715,800,865,693,754,874,719,808,873,833,780,744,810,674,718,767,811,824,835,797,733,749,673,738,798,690,644,796,772,780,733,756,757,736,747,772,700,675,711,822,719,691,759,642,686,578,702,676,650,739,802,714,756,683,712,772,684,752,765,695,734,741,743,760,609,686,687,792,678,692,686,771,639,701,767,786,712,660,814,649,760,708,613,717,742,704,707,665,731,808,764,693,726,674,651,605,767,655,699,691,616,735,733,710,810,748,845,753,738,811,762,700,620,764,696,685,754,676,693,681,682,714,714,703,701,811,791,704,897,886,852,849,646,607,642,576,518,740,674,683,656,692,824,744,587,944,888,899,816,893,735,773,812,839,685,728,785,578,687,687,908,452,876,769,89

	
```

```
		  

		NC_006621_3, Position,0,1000000,2000000,3000000,4000000,5000000,6000000,7000000,8000000,9000000,10000000,11000000,12000000,13000000,14000000,15000000,16000000,17000000,18000000,19000000,20000000,21000000,22000000,23000000,24000000,25000000,26000000,27000000,28000000,29000000,30000000,31000000,32000000,33000000,34000000,35000000,36000000,37000000,38000000,39000000,40000000,41000000,42000000,43000000,44000000,45000000,46000000,47000000,48000000,49000000,50000000,51000000,52000000,53000000,54000000,55000000,56000000,57000000,58000000,59000000,60000000,61000000,62000000,63000000,64000000,65000000,66000000,67000000,68000000,69000000,70000000,71000000,72000000,73000000,74000000,75000000,76000000,77000000,78000000,79000000,80000000,81000000,82000000,83000000,84000000,85000000,86000000,87000000,88000000,89000000,90000000,91000000,92000000,93000000,94000000,95000000,96000000,97000000,98000000,99000000,100000000,101000000,102000000,103000000,104000000,105000000,106000000,107000000,108000000,109000000,110000000,111000000,112000000,113000000,114000000,115000000,116000000,117000000,118000000,119000000,120000000,121000000,122000000,123000000
NC_006621_3,Count,6671,7840,8200,8341,8244,7311,6357,4449,4258,4678,4390,4056,3991,4090,3825,3910,3982,3961,4244,3736,3927,4128,3837,3926,4606,4085,4084,4021,4381,4065,4234,3752,3991,4629,4712,3550,4340,4449,3990,3956,3626,3710,3563,3446,2875,3316,2634,3108,1702,3663,3778,4138,3845,4072,3629,3693,3509,3827,3620,3498,3673,3434,3835,3685,3945,3921,4030,3876,4063,4331,4397,4160,4290,4230,3978,3638,3249,3573,3928,3653,3601,3774,3878,3756,3886,3770,3926,4083,3785,3910,3828,4043,3370,3869,3866,3879,4220,3788,3967,4139,3964,3570,3818,4126,4033,3627,3741,4034,4031,3916,4232,4700,4343,4563,4201,4122,4055,3302,3729,4587,3731,4601,3296,3229

	
```

```
		  

		NW_003726127_1, Position,0,10000,20000,30000,40000,50000,60000,70000,80000,90000,100000,110000,120000,130000,140000,150000,160000,170000,180000,190000,200000,210000,220000,230000,240000,250000,260000,270000,280000,290000,300000,310000,320000,330000,340000,350000,360000,370000,380000,390000,400000,410000,420000,430000,440000,450000,460000,470000,480000,490000,500000,510000,520000,530000,540000,550000,560000,570000,580000,590000,600000,610000,620000,630000,640000,650000,660000,670000,680000,690000,700000,710000,720000,730000,740000,750000,760000,770000,780000,790000,800000,810000,820000,830000,840000,850000,860000,870000,880000,890000,900000,910000,920000,930000,940000,950000,960000,970000,980000,990000,1000000,1010000,1020000,1030000,1040000,1050000,1060000,1070000,1080000,1090000,1100000,1110000,1120000,1130000,1140000,1150000,1160000,1170000,1180000,1190000,1200000,1210000,1220000,1230000,1240000,1250000,1260000,1270000,1280000,1290000,1300000,1310000,1320000,1330000,1340000,1350000,1360000,1370000,1380000,1390000,1400000,1410000,1420000,1430000,1440000,1450000,1460000,1470000,1480000,1490000,1500000,1510000,1520000,1530000,1540000,1550000,1560000,1570000,1580000,1590000,1600000,1610000,1620000,1630000,1640000,1650000,1660000,1670000,1680000,1690000,1700000,1710000,1720000,1730000,1740000,1750000,1760000,1770000,1780000,1790000,1800000,1810000,1820000,1830000,1840000,1850000,1860000,1870000,1880000,1890000,1900000,1910000,1920000,1930000,1940000,1950000,1960000,1970000,1980000,1990000,2000000,2010000,2020000,2030000,2040000,2050000,2060000,2070000,2080000,2090000,2100000,2110000,2120000,2130000,2140000,2150000,2160000,2170000,2180000,2190000,2200000,2210000,2220000,2230000,2240000,2250000,2260000,2270000,2280000,2290000,2300000,2310000,2320000,2330000,2340000,2350000,2360000,2370000,2380000,2390000,2400000,2410000,2420000,2430000,2440000,2450000,2460000,2470000,2480000,2490000,2500000,2510000,2520000,2530000,2540000,2550000,2560000,2570000,2580000,2590000,2600000,2610000,2620000,2630000,2640000,2650000,2660000
NW_003726127_1,Count,0,0,0,0,0,0,0,0,0,0,0,0,0,0,1,0,0,0,3,0,0,1,0,0,0,0,0,0,0,0,0,0,0,0,0,1,0,0,3,0,0,0,0,0,0,0,0,0,0,0,0,0,0,0,0,0,0,0,0,0,0,0,1,0,0,0,0,0,0,0,0,0,2,0,2,0,0,0,0,0,0,0,0,0,0,0,0,1,0,0,0,1,0,0,0,0,0,0,0,0,0,0,0,0,0,0,0,0,0,0,0,0,0,0,0,0,0,0,0,0,0,5,8,0,0,0,0,0,0,0,0,0,1,0,0,0,0,0,0,0,0,0,0,0,0,0,0,0,0,0,0,0,0,0,0,0,0,0,0,0,0,0,0,0,0,2,0,0,0,0,0,0,0,0,0,0,0,0,0,0,3,0,0,0,0,0,0,5,0,0,0,0,0,0,0,0,0,0,0,0,0,0,0,0,0,0,0,0,0,0,0,3,0,0,0,0,0,0,0,1,0,0,0,0,0,0,0,0,0,0,0,0,0,0,0,0,1,0,7,7,0,3,9,2,2,9,3,9,0,0,0,0,0,0,3,4,0,10,6,7,4,9,20,1,0,0,0

	
```

```
		  

		NW_003726129_1, Position,0,10000,20000,30000,40000,50000,60000,70000,80000,90000,100000,110000,120000,130000,140000,150000,160000,170000,180000,190000,200000,210000,220000,230000,240000,250000,260000,270000,280000,290000,300000,310000,320000,330000,340000,350000,360000,370000,380000,390000,400000,410000,420000,430000,440000,450000,460000,470000,480000,490000,500000,510000,520000,530000,540000,550000,560000,570000,580000,590000,600000,610000,620000,630000,640000,650000,660000,670000,680000,690000,700000,710000,720000,730000,740000,750000,760000,770000,780000,790000,800000,810000,820000,830000,840000,850000,860000,870000,880000,890000,900000,910000,920000,930000,940000,950000,960000,970000,980000,990000,1000000,1010000,1020000,1030000,1040000,1050000,1060000,1070000,1080000,1090000,1100000,1110000,1120000,1130000,1140000,1150000,1160000,1170000,1180000,1190000,1200000,1210000,1220000,1230000,1240000,1250000,1260000,1270000,1280000,1290000,1300000,1310000,1320000,1330000,1340000,1350000,1360000,1370000,1380000,1390000,1400000,1410000
NW_003726129_1,Count,0,0,0,0,4,0,0,0,0,2,0,0,0,0,0,0,0,0,0,0,0,0,0,0,0,0,0,0,0,0,0,0,0,0,0,0,0,0,0,0,0,0,0,0,0,0,0,0,0,0,0,0,0,0,0,0,0,0,0,0,0,0,0,0,0,0,0,0,0,0,0,0,0,0,0,0,0,0,0,0,0,0,0,0,0,0,0,0,0,0,0,0,0,0,0,0,0,0,0,0,0,0,0,0,0,0,0,0,0,0,0,0,0,0,0,0,0,0,0,0,0,0,0,0,0,0,0,0,0,0,0,0,0,0,0,0,0,0,0,0,0,0

	
```

```
		  

		NW_003726130_1, Position,0,10000,20000,30000,40000,50000,60000,70000,80000,90000,100000,110000,120000,130000,140000,150000,160000,170000,180000,190000,200000,210000,220000,230000,240000,250000,260000,270000,280000,290000,300000,310000,320000,330000,340000,350000,360000,370000,380000,390000,400000,410000,420000,430000,440000,450000,460000,470000,480000,490000,500000,510000,520000,530000,540000,550000,560000,570000,580000,590000,600000,610000,620000,630000,640000,650000,660000,670000,680000,690000,700000,710000,720000,730000,740000,750000,760000,770000,780000,790000,800000,810000,820000,830000,840000,850000,860000,870000,880000,890000,900000,910000,920000,930000,940000,950000,960000,970000,980000,990000,1000000,1010000,1020000,1030000,1040000,1050000,1060000
NW_003726130_1,Count,0,0,0,0,0,0,0,0,0,0,0,0,0,0,0,0,0,0,0,0,0,0,0,0,0,0,0,0,0,0,0,0,0,0,0,0,0,0,0,0,0,0,0,0,0,0,0,0,0,0,0,0,0,0,0,0,0,0,0,0,2,0,0,0,0,0,0,0,0,0,0,0,0,0,0,0,0,0,0,0,0,0,0,0,0,0,0,0,1,0,0,0,0,0,0,0,0,0,0,0,0,2,0,0,0,15,7

	
```

```
		  

		NW_003726132_1, Position,0,10000,20000,30000,40000,50000,60000,70000,80000,90000,100000,110000,120000,130000,140000,150000,160000,170000,180000,190000,200000,210000,220000,230000,240000,250000,260000,270000,280000,290000,300000,310000,320000,330000,340000,350000,360000,370000,380000,390000,400000,410000,420000,430000,440000,450000,460000,470000,480000,490000,500000,510000,520000,530000,540000,550000,560000,570000,580000,590000,600000,610000,620000,630000,640000,650000,660000,670000,680000,690000,700000,710000,720000,730000,740000,750000,760000,770000,780000,790000,800000,810000,820000,830000,840000,850000,860000,870000,880000
NW_003726132_1,Count,0,0,0,0,0,0,3,0,0,0,0,0,0,0,0,0,0,0,2,0,0,8,7,3,0,0,0,0,0,0,0,0,0,0,0,0,0,0,0,0,0,0,0,0,0,0,0,0,0,1,0,0,0,0,0,0,0,0,0,0,0,0,0,0,0,0,2,0,0,0,0,0,0,0,0,0,0,0,0,0,0,0,0,0,0,0,0,1,0

	
```

```
		  

		NW_003726133_1, Position,0,10000,20000,30000,40000,50000,60000,70000,80000,90000,100000,110000,120000,130000,140000,150000,160000,170000,180000,190000,200000,210000,220000,230000,240000,250000,260000,270000,280000,290000,300000,310000,320000,330000,340000,350000,360000,370000,380000,390000,400000,410000,420000,430000,440000,450000,460000,470000,480000,490000,500000,510000,520000,530000,540000,550000,560000,570000,580000,590000,600000,610000,620000,630000,640000,650000,660000,670000,680000,690000,700000,710000,720000,730000,740000,750000,760000,770000,780000,790000,800000,810000,820000
NW_003726133_1,Count,0,0,0,0,60,93,108,151,94,119,123,129,96,122,128,112,102,98,118,139,122,79,6,60,107,27,79,9,42,10,6,0,0,0,0,0,0,79,164,141,39,124,123,140,140,145,131,105,95,99,32,0,0,0,0,49,93,128,104,138,129,118,97,91,99,156,62,131,152,159,156,91,104,82,8,0,0,0,0,0,0,0,0

	
```

```
		  

		NW_003726135_1, Position,0,10000,20000,30000,40000,50000,60000,70000,80000,90000,100000,110000,120000,130000,140000,150000,160000,170000,180000,190000,200000,210000,220000,230000,240000,250000,260000,270000,280000,290000,300000,310000,320000,330000,340000,350000,360000,370000,380000,390000,400000,410000,420000,430000,440000,450000,460000,470000,480000,490000,500000,510000,520000,530000,540000,550000,560000,570000,580000,590000,600000,610000,620000,630000,640000,650000,660000,670000,680000,690000,700000,710000,720000,730000,740000
NW_003726135_1,Count,0,0,0,0,0,0,0,0,0,0,0,1,0,0,0,0,0,0,0,0,0,0,0,0,0,0,0,0,0,0,0,0,0,0,4,15,15,17,5,13,0,0,0,1,0,0,0,0,3,0,0,8,7,0,7,0,3,4,10,2,5,4,0,2,0,2,9,0,1,1,4,0,6,25,19

	
```

```
		  

		NW_003726136_1, Position,0,10000,20000,30000,40000,50000,60000,70000,80000,90000,100000,110000,120000,130000,140000,150000,160000,170000,180000,190000,200000,210000,220000,230000,240000,250000,260000,270000,280000,290000,300000,310000,320000,330000,340000,350000,360000,370000,380000,390000,400000,410000,420000,430000,440000,450000,460000,470000,480000,490000,500000,510000,520000,530000,540000,550000,560000,570000,580000,590000,600000
NW_003726136_1,Count,1,7,6,2,0,0,13,8,3,0,0,0,2,2,11,2,0,5,0,1,1,2,13,6,7,0,12,5,3,2,15,1,3,0,12,5,15,8,6,11,0,0,6,7,10,1,1,9,2,0,0,8,2,3,24,66,68,52,81,83,14

	
```

```
		  

		NW_003726137_1, Position,0,10000,20000,30000,40000,50000,60000,70000,80000,90000,100000,110000,120000,130000,140000,150000,160000,170000,180000,190000,200000,210000,220000,230000,240000,250000,260000,270000,280000,290000,300000,310000,320000,330000,340000,350000,360000,370000,380000,390000,400000,410000,420000,430000,440000,450000,460000,470000,480000,490000,500000,510000,520000,530000,540000,550000,560000,570000
NW_003726137_1,Count,77,108,72,72,94,82,84,124,95,61,47,43,62,60,58,64,64,75,81,74,72,102,88,63,84,86,66,83,92,86,93,84,81,115,91,103,87,113,99,103,92,124,55,90,66,106,102,90,117,88,109,89,89,80,6,70,44,25

	
```

```
		  

		NW_003726139_1, Position,0,10000,20000,30000,40000,50000,60000,70000,80000,90000,100000,110000,120000,130000,140000,150000,160000,170000,180000,190000,200000,210000,220000,230000,240000,250000,260000,270000,280000,290000,300000,310000,320000,330000,340000,350000,360000,370000,380000,390000,400000,410000,420000,430000,440000,450000,460000,470000,480000,490000,500000,510000,520000,530000,540000,550000
NW_003726139_1,Count,1,0,1,0,0,0,0,0,0,0,0,0,0,0,0,0,0,4,0,4,0,0,0,0,0,0,0,7,21,0,1,0,0,0,4,0,1,0,0,0,0,3,0,0,3,0,0,0,1,0,9,1,5,8,10,10

	
```

```
		  

		NW_003726140_1, Position,0,10000,20000,30000,40000,50000,60000,70000,80000,90000,100000,110000,120000,130000,140000,150000,160000,170000,180000,190000,200000,210000,220000,230000,240000,250000,260000,270000,280000,290000,300000,310000,320000,330000,340000,350000,360000,370000,380000,390000,400000,410000,420000,430000,440000,450000,460000,470000,480000,490000,500000,510000
NW_003726140_1,Count,0,0,0,0,0,0,0,0,0,0,0,0,0,0,0,0,0,0,0,0,0,0,0,0,0,0,0,0,0,0,0,0,0,0,0,0,0,3,0,0,0,0,0,0,0,0,0,0,0,0,0,0

	
```

```
		  

		NW_003726141_1, Position,0,10000,20000,30000,40000,50000,60000,70000,80000,90000,100000,110000,120000,130000,140000,150000,160000,170000,180000,190000,200000,210000,220000,230000,240000,250000,260000,270000,280000,290000,300000,310000,320000,330000,340000,350000,360000,370000,380000,390000,400000,410000,420000,430000,440000,450000,460000,470000,480000,490000
NW_003726141_1,Count,23,0,0,0,0,0,3,0,0,0,0,0,0,1,16,13,0,0,0,0,0,0,0,0,0,0,0,0,0,0,0,7,1,6,1,2,0,10,19,6,0,5,0,0,0,0,0,0,1,0

	
```

```
		  

		NW_003726142_1, Position,0,10000,20000,30000,40000,50000,60000,70000,80000,90000,100000,110000,120000,130000,140000,150000,160000,170000,180000,190000,200000,210000,220000,230000,240000,250000,260000,270000,280000,290000,300000,310000,320000,330000,340000,350000,360000,370000,380000,390000,400000,410000,420000,430000,440000,450000,460000,470000,480000,490000
NW_003726142_1,Count,4,4,0,0,0,0,0,1,0,0,0,5,7,4,6,0,2,1,5,0,0,1,0,5,6,1,0,0,0,0,0,0,0,4,3,0,0,4,0,0,0,0,0,0,0,0,0,0,0,0

	
```

```
		  

		NW_003726143_1, Position,0,10000,20000,30000,40000,50000,60000,70000,80000,90000,100000,110000,120000,130000,140000,150000,160000,170000,180000,190000,200000,210000,220000,230000,240000,250000,260000,270000,280000,290000,300000,310000,320000,330000,340000,350000,360000,370000,380000,390000,400000,410000,420000,430000,440000,450000,460000,470000,480000,490000,500000,510000,520000,530000,540000,550000,560000,570000
NW_003726143_1,Count,70,64,70,62,11,0,0,0,0,1,0,67,79,68,75,62,66,6,0,7,67,59,52,54,24,7,0,0,0,0,0,0,0,0,0,0,0,0,0,0,0,0,0,0,1,6,0,0,0,0,0,0,0,0,0,0,0,0

	
```

```
		  

		NW_003726144_1, Position,0,10000,20000,30000,40000,50000,60000,70000,80000,90000,100000,110000,120000,130000,140000,150000,160000,170000,180000,190000,200000,210000,220000,230000,240000,250000,260000,270000,280000,290000,300000,310000,320000,330000,340000,350000,360000,370000,380000,390000,400000,410000,420000,430000,440000,450000,460000,470000,480000,490000,500000,510000,520000,530000,540000,550000,560000
NW_003726144_1,Count,10,1,3,0,4,9,0,1,0,0,0,8,4,1,4,8,0,0,0,0,0,0,4,1,12,3,3,6,4,0,0,1,0,9,4,4,1,0,1,4,10,2,0,0,0,0,0,0,0,0,0,9,3,14,0,17,2

	
```

```
		  

		NW_003726145_1, Position,0,10000,20000,30000,40000,50000,60000,70000,80000,90000,100000,110000,120000,130000,140000,150000,160000,170000,180000,190000,200000,210000,220000,230000,240000,250000,260000,270000,280000,290000,300000,310000,320000,330000,340000,350000,360000,370000,380000,390000,400000
NW_003726145_1,Count,2,0,0,0,0,0,0,0,0,0,0,0,0,0,0,0,0,0,0,0,0,0,0,0,0,0,0,0,0,0,0,0,0,0,0,0,0,0,0,0,0

	
```

```
		  

		NW_003726147_1, Position,0,10000,20000,30000,40000,50000,60000,70000,80000,90000,100000,110000,120000,130000,140000,150000,160000,170000,180000,190000,200000,210000,220000,230000,240000,250000,260000,270000,280000,290000,300000,310000,320000,330000,340000,350000,360000,370000,380000,390000,400000,410000,420000,430000,440000,450000,460000,470000,480000,490000,500000
NW_003726147_1,Count,5,0,0,0,0,0,0,0,0,0,0,7,0,0,0,4,3,4,0,0,0,0,0,0,0,0,0,0,0,0,2,0,0,4,0,0,0,0,0,0,0,0,0,0,0,0,0,1,0,0,0

	
```

```
		  

		NW_003726148_1, Position,0,10000,20000,30000,40000,50000,60000,70000,80000,90000,100000,110000,120000,130000,140000,150000,160000,170000,180000,190000,200000,210000,220000,230000,240000,250000,260000,270000,280000,290000,300000,310000,320000,330000,340000,350000,360000,370000,380000,390000,400000,410000,420000,430000,440000,450000,460000,470000,480000,490000,500000,510000,520000,530000,540000
NW_003726148_1,Count,2,0,0,0,0,0,0,0,0,0,0,0,0,0,0,0,0,0,0,0,25,3,0,0,0,0,0,0,0,0,0,0,0,0,0,0,0,1,0,0,9,2,0,0,0,0,0,0,0,0,0,3,0,8,0

	
```

```
		  

		NW_003726149_1, Position,0,10000,20000,30000,40000,50000,60000,70000,80000,90000,100000,110000,120000,130000,140000,150000,160000,170000,180000,190000,200000,210000,220000,230000,240000,250000,260000,270000,280000,290000,300000,310000,320000,330000,340000,350000,360000,370000,380000,390000,400000,410000,420000,430000,440000
NW_003726149_1,Count,40,67,32,59,59,34,27,40,73,25,21,33,38,70,65,60,0,0,0,0,0,0,28,26,43,20,27,10,0,70,82,50,0,0,31,47,53,56,64,71,56,63,81,60,20

	
```

```
		  

		NW_003726150_1, Position,0,10000,20000,30000,40000,50000,60000,70000,80000,90000,100000,110000,120000,130000,140000,150000,160000,170000,180000,190000,200000,210000,220000,230000,240000,250000,260000,270000,280000,290000,300000,310000,320000,330000,340000,350000,360000,370000
NW_003726150_1,Count,0,0,0,0,0,5,0,0,0,0,0,0,0,0,0,0,0,0,0,0,0,1,0,0,0,0,0,0,0,0,2,4,0,0,0,0,0,0

	
```

```
		  

		NW_003726151_1, Position,0,10000,20000,30000,40000,50000,60000,70000,80000,90000,100000,110000,120000,130000,140000,150000,160000,170000,180000,190000,200000,210000,220000,230000,240000,250000,260000,270000,280000,290000,300000,310000,320000,330000,340000,350000,360000,370000,380000
NW_003726151_1,Count,14,8,1,2,0,0,0,0,0,0,0,0,0,0,4,0,0,0,0,0,0,1,0,0,4,9,15,4,0,0,1,0,0,2,0,4,2,0,2

	
```

```
		  

		NW_003726153_1, Position,0,10000,20000,30000,40000,50000,60000,70000,80000,90000,100000,110000,120000,130000,140000,150000,160000,170000,180000,190000,200000,210000,220000,230000,240000,250000,260000,270000,280000,290000,300000,310000,320000,330000,340000,350000,360000,370000,380000,390000
NW_003726153_1,Count,16,21,8,6,0,0,0,0,0,0,0,0,0,0,0,0,0,0,0,0,0,0,0,0,0,0,0,0,0,0,0,1,1,3,5,11,2,0,0,1

	
```

```
		  

		NW_003726156_1, Position,0,10000,20000,30000,40000,50000,60000,70000,80000,90000,100000,110000,120000,130000,140000,150000,160000,170000,180000,190000,200000,210000,220000,230000,240000,250000,260000,270000,280000,290000,300000,310000,320000,330000,340000,350000,360000
NW_003726156_1,Count,34,3,3,5,0,0,11,0,0,10,10,6,1,0,13,4,7,3,0,5,6,0,8,0,0,0,0,1,0,0,0,0,7,0,12,4,11

	
```

```
		  

		NW_003726159_1, Position,0,10000,20000,30000,40000,50000,60000,70000,80000,90000,100000,110000,120000,130000,140000,150000,160000,170000,180000,190000,200000,210000,220000,230000,240000,250000,260000,270000,280000,290000,300000,310000
NW_003726159_1,Count,1,1,0,0,0,0,0,0,0,0,0,0,0,0,0,0,0,0,4,0,3,4,0,0,0,0,1,0,2,5,0,0

	
```

```
		  

		NW_003726160_1, Position,0,10000,20000,30000,40000,50000,60000,70000,80000,90000,100000,110000,120000,130000,140000,150000,160000,170000,180000,190000,200000,210000,220000,230000,240000,250000,260000,270000,280000,290000,300000,310000,320000,330000,340000,350000,360000,370000,380000,390000,400000,410000,420000,430000
NW_003726160_1,Count,1,0,0,0,0,0,0,0,0,0,0,0,0,0,3,0,0,0,0,0,0,0,0,0,0,0,0,0,0,0,0,0,0,0,0,0,0,0,0,0,0,0,0,0

	
```

```
		  

		NW_003726162_1, Position,0,1000,2000,3000,4000,5000,6000,7000,8000,9000,10000,11000,12000,13000,14000,15000,16000,17000,18000,19000,20000,21000,22000,23000,24000,25000,26000,27000,28000,29000,30000,31000,32000,33000,34000,35000,36000,37000,38000,39000,40000,41000,42000,43000,44000,45000,46000,47000,48000,49000,50000,51000,52000,53000,54000,55000,56000,57000,58000,59000,60000,61000,62000,63000,64000,65000,66000,67000,68000,69000,70000,71000,72000,73000,74000,75000,76000,77000,78000,79000,80000,81000,82000,83000,84000,85000,86000,87000,88000,89000,90000,91000,92000,93000,94000,95000,96000,97000,98000,99000,100000,101000,102000,103000,104000,105000,106000,107000,108000,109000,110000,111000,112000,113000,114000,115000,116000,117000,118000,119000,120000,121000,122000,123000,124000,125000,126000,127000,128000,129000,130000,131000,132000,133000,134000,135000,136000,137000,138000,139000,140000,141000,142000,143000,144000,145000,146000,147000,148000,149000,150000,151000,152000,153000,154000,155000,156000,157000,158000,159000,160000,161000,162000,163000,164000,165000,166000,167000,168000,169000,170000,171000,172000,173000,174000,175000,176000,177000,178000,179000,180000,181000,182000,183000,184000,185000,186000,187000,188000,189000,190000,191000,192000,193000,194000,195000,196000,197000,198000,199000,200000,201000,202000,203000,204000,205000,206000,207000,208000,209000,210000,211000,212000,213000,214000,215000,216000,217000,218000,219000,220000,221000,222000,223000,224000,225000,226000,227000,228000,229000,230000,231000,232000,233000,234000,235000,236000,237000,238000,239000,240000,241000,242000,243000,244000,245000,246000,247000,248000,249000,250000,251000,252000,253000,254000,255000,256000,257000,258000,259000,260000,261000,262000,263000,264000,265000,266000,267000,268000,269000,270000,271000,272000,273000,274000,275000,276000,277000,278000,279000,280000,281000
NW_003726162_1,Count,0,0,0,0,0,0,0,0,0,0,0,0,0,0,0,0,0,0,0,0,0,0,0,0,0,0,0,0,0,0,0,0,0,0,0,0,0,0,0,0,0,0,0,0,0,0,0,0,0,0,0,0,0,0,0,0,0,0,0,0,0,0,0,0,0,0,0,0,0,0,0,0,0,0,0,0,0,0,0,0,0,0,0,0,0,0,0,0,0,0,0,0,0,0,0,0,0,0,0,0,0,0,0,0,0,0,0,0,0,0,0,0,0,0,0,0,0,0,0,0,0,0,0,0,0,0,0,0,2,0,0,0,0,0,0,0,0,0,0,0,0,0,0,0,0,0,0,0,0,0,0,0,0,0,0,0,0,0,0,0,0,0,0,0,0,0,0,0,0,0,0,0,0,0,0,0,0,0,0,0,2,0,0,0,0,0,0,0,0,0,0,0,0,0,0,0,0,0,0,0,0,0,0,0,0,0,0,0,0,0,0,0,0,0,0,0,0,0,0,0,0,0,0,0,0,0,0,0,0,0,0,0,0,0,0,0,0,0,0,0,0,0,0,0,0,0,0,0,0,0,0,0,0,0,0,0,0,0,0,0,0,0,0,0,0,0,0,0,0,0,0,0,0,0,0,0,0,0,0,0,0,0

	
```

```
		  

		NW_003726163_1, Position,0,10000,20000,30000,40000,50000,60000,70000,80000,90000,100000,110000,120000,130000,140000,150000,160000,170000,180000,190000,200000,210000,220000,230000,240000,250000,260000,270000,280000,290000,300000,310000,320000
NW_003726163_1,Count,0,0,0,0,0,0,0,0,0,0,0,0,0,0,0,0,0,0,0,0,0,0,0,0,0,0,2,0,0,0,2,0,0

	
```

```
		  

		NW_003726167_1, Position,0,1000,2000,3000,4000,5000,6000,7000,8000,9000,10000,11000,12000,13000,14000,15000,16000,17000,18000,19000,20000,21000,22000,23000,24000,25000,26000,27000,28000,29000,30000,31000,32000,33000,34000,35000,36000,37000,38000,39000,40000,41000,42000,43000,44000,45000,46000,47000,48000,49000,50000,51000,52000,53000,54000,55000,56000,57000,58000,59000,60000,61000,62000,63000,64000,65000,66000,67000,68000,69000,70000,71000,72000,73000,74000,75000,76000,77000,78000,79000,80000,81000,82000,83000,84000,85000,86000,87000,88000,89000,90000,91000,92000,93000,94000,95000,96000,97000,98000,99000,100000,101000,102000,103000,104000,105000,106000,107000,108000,109000,110000,111000,112000,113000,114000,115000,116000,117000,118000,119000,120000,121000,122000,123000,124000,125000,126000,127000,128000,129000,130000,131000,132000,133000,134000,135000,136000,137000,138000,139000,140000,141000,142000,143000,144000,145000,146000,147000,148000,149000,150000,151000,152000,153000,154000,155000,156000,157000,158000,159000,160000,161000,162000,163000,164000,165000,166000,167000,168000,169000,170000,171000,172000,173000,174000,175000,176000,177000,178000,179000,180000,181000,182000,183000,184000,185000,186000,187000,188000,189000,190000,191000,192000,193000,194000,195000,196000,197000,198000,199000,200000,201000,202000,203000,204000,205000,206000,207000,208000,209000,210000,211000,212000,213000,214000,215000,216000,217000,218000,219000,220000,221000,222000,223000,224000,225000,226000,227000,228000,229000,230000,231000,232000,233000,234000,235000,236000,237000,238000,239000,240000,241000,242000,243000,244000,245000,246000,247000,248000,249000,250000,251000,252000
NW_003726167_1,Count,2,0,2,0,2,4,0,6,0,0,0,0,3,0,0,0,0,0,4,5,0,0,0,0,0,4,0,0,3,0,3,0,0,3,0,0,0,2,1,0,4,3,1,0,4,0,1,0,0,0,1,6,5,1,0,0,0,0,1,0,0,0,0,0,0,0,0,0,0,0,0,0,0,0,0,0,0,0,1,0,0,0,0,0,0,0,0,0,0,0,0,0,0,0,0,0,0,0,0,0,0,0,0,0,0,0,0,0,0,0,0,0,0,2,0,0,0,0,0,0,0,0,0,0,0,0,0,0,0,0,0,0,0,3,0,0,0,3,0,0,0,0,0,0,0,0,0,0,0,0,0,0,0,0,0,0,0,0,0,0,0,0,0,0,0,0,0,0,0,0,0,0,0,0,0,0,0,0,0,0,0,0,0,0,0,0,0,0,0,0,0,0,0,0,0,0,0,0,0,0,0,0,0,0,1,0,0,3,0,0,0,0,0,0,0,1,0,0,0,0,0,0,0,0,0,0,0,2,5,0,3,5,1,0,3,0,0,0,0,0,2,1,0,0,2,0,0,0,0,0,2,2,0

	
```

```
		  

		NW_003726168_1, Position,0,1000,2000,3000,4000,5000,6000,7000,8000,9000,10000,11000,12000,13000,14000,15000,16000,17000,18000,19000,20000,21000,22000,23000,24000,25000,26000,27000,28000,29000,30000,31000,32000,33000,34000,35000,36000,37000,38000,39000,40000,41000,42000,43000,44000,45000,46000,47000,48000,49000,50000,51000,52000,53000,54000,55000,56000,57000,58000,59000,60000,61000,62000,63000,64000,65000,66000,67000,68000,69000,70000,71000,72000,73000,74000,75000,76000,77000,78000,79000,80000,81000,82000,83000,84000,85000,86000,87000,88000,89000,90000,91000,92000,93000,94000,95000,96000,97000,98000,99000,100000,101000,102000,103000,104000,105000,106000,107000,108000,109000,110000,111000,112000,113000,114000,115000,116000,117000,118000,119000,120000,121000,122000,123000,124000,125000,126000,127000,128000,129000,130000,131000,132000,133000,134000,135000,136000,137000,138000,139000,140000,141000,142000,143000,144000,145000,146000,147000,148000,149000,150000,151000,152000,153000,154000,155000,156000,157000,158000,159000,160000,161000,162000,163000,164000,165000,166000,167000,168000,169000,170000,171000,172000,173000,174000,175000,176000,177000,178000,179000,180000,181000,182000,183000,184000,185000,186000,187000,188000,189000,190000,191000,192000,193000,194000,195000,196000,197000,198000,199000,200000,201000,202000,203000,204000,205000,206000,207000,208000,209000,210000,211000,212000,213000,214000,215000,216000,217000,218000,219000,220000,221000,222000,223000,224000,225000,226000,227000,228000,229000,230000,231000,232000,233000,234000,235000,236000,237000,238000,239000,240000,241000,242000,243000,244000,245000,246000,247000,248000,249000,250000,251000,252000,253000,254000
NW_003726168_1,Count,0,0,0,0,0,0,0,0,0,0,0,0,3,0,0,0,0,0,0,0,0,0,0,3,0,0,0,2,1,0,0,0,0,0,0,0,0,0,0,0,0,0,0,0,0,0,0,0,0,0,0,0,0,0,0,1,1,0,0,0,0,0,0,0,0,0,0,0,0,0,0,0,0,0,0,0,0,0,0,0,0,0,0,0,0,0,0,0,0,0,0,0,0,0,0,0,0,0,12,2,0,10,11,12,3,7,0,7,3,1,0,2,9,3,6,0,4,0,0,0,0,5,5,0,4,0,0,0,0,0,0,0,0,0,0,8,2,0,0,0,0,0,6,1,0,0,0,2,5,0,3,0,5,3,1,3,0,2,0,0,0,0,0,0,0,0,1,0,0,0,3,0,0,0,0,0,0,0,0,0,0,0,0,0,0,0,0,0,0,0,0,0,0,0,2,0,0,0,0,0,0,0,0,2,2,0,0,0,0,0,4,2,2,5,0,0,0,0,0,0,0,0,0,0,0,3,0,0,0,0,0,0,2,1,0,2,5,9,1,0,0,0,0,1,0,0,7,8,7,8,5,0,5,12,4

	
```

```
		  

		NW_003726174_1, Position,0,10000,20000,30000,40000,50000,60000,70000,80000,90000,100000,110000,120000,130000,140000,150000,160000,170000,180000,190000,200000,210000,220000,230000,240000,250000,260000,270000,280000,290000,300000,310000,320000,330000,340000,350000,360000,370000,380000
NW_003726174_1,Count,82,59,31,0,0,0,0,0,0,0,0,0,0,0,0,0,0,29,52,55,51,70,64,89,85,85,67,41,19,54,74,57,64,40,2,55,71,66,61

	
```

```
		  

		NW_003726177_1, Position,0,1000,2000,3000,4000,5000,6000,7000,8000,9000,10000,11000,12000,13000,14000,15000,16000,17000,18000,19000,20000,21000,22000,23000,24000,25000,26000,27000,28000,29000,30000,31000,32000,33000,34000,35000,36000,37000,38000,39000,40000,41000,42000,43000,44000,45000,46000,47000,48000,49000,50000,51000,52000,53000,54000,55000,56000,57000,58000,59000,60000,61000,62000,63000,64000,65000,66000,67000,68000,69000,70000,71000,72000,73000,74000,75000,76000,77000,78000,79000,80000,81000,82000,83000,84000,85000,86000,87000,88000,89000,90000,91000,92000,93000,94000,95000,96000,97000,98000,99000,100000,101000,102000,103000,104000,105000,106000,107000,108000,109000,110000,111000,112000,113000,114000,115000,116000,117000,118000,119000,120000,121000,122000,123000,124000,125000,126000,127000,128000,129000,130000,131000,132000,133000,134000,135000,136000,137000,138000,139000,140000,141000,142000,143000,144000,145000,146000,147000,148000,149000,150000,151000,152000,153000,154000,155000,156000,157000,158000,159000,160000,161000,162000,163000,164000,165000,166000,167000,168000,169000,170000,171000,172000,173000,174000,175000,176000,177000,178000,179000,180000,181000,182000,183000,184000,185000,186000,187000,188000,189000,190000,191000,192000,193000,194000,195000,196000,197000,198000,199000,200000,201000,202000,203000,204000,205000,206000,207000,208000,209000,210000,211000,212000,213000,214000,215000,216000
NW_003726177_1,Count,0,0,0,1,0,2,0,0,0,0,0,0,0,0,0,0,0,0,0,0,0,0,0,0,0,0,0,0,0,0,1,0,0,0,0,0,0,0,0,1,0,0,0,0,0,0,0,0,0,0,0,0,0,0,0,0,0,0,0,0,0,0,0,0,0,0,0,0,0,0,0,0,0,0,0,0,0,0,0,0,0,0,0,0,0,0,0,0,0,0,0,2,0,0,0,0,0,0,0,0,0,0,0,0,0,0,0,0,0,0,0,0,0,0,0,0,0,1,2,8,0,0,0,0,2,0,0,0,0,0,0,0,0,0,0,0,0,0,0,0,2,0,0,0,0,0,0,2,0,0,0,0,0,0,0,0,0,0,0,0,0,0,0,0,0,0,0,0,0,0,0,0,0,0,0,0,0,0,0,0,0,0,0,0,0,0,0,0,0,0,0,0,0,0,0,0,0,0,0,0,0,0,0,0,0,0,0,0,0,0,0,0,0,0,0,0,0

	
```

```
		  

		NW_003726180_1, Position,0,1000,2000,3000,4000,5000,6000,7000,8000,9000,10000,11000,12000,13000,14000,15000,16000,17000,18000,19000,20000,21000,22000,23000,24000,25000,26000,27000,28000,29000,30000,31000,32000,33000,34000,35000,36000,37000,38000,39000,40000,41000,42000,43000,44000,45000,46000,47000,48000,49000,50000,51000,52000,53000,54000,55000,56000,57000,58000,59000,60000,61000,62000,63000,64000,65000,66000,67000,68000,69000,70000,71000,72000,73000,74000,75000,76000,77000,78000,79000,80000,81000,82000,83000,84000,85000,86000,87000,88000,89000,90000,91000,92000,93000,94000,95000,96000,97000,98000,99000,100000,101000,102000,103000,104000,105000,106000,107000,108000,109000,110000,111000,112000,113000,114000,115000,116000,117000,118000,119000,120000,121000,122000,123000,124000,125000,126000,127000,128000,129000,130000,131000,132000,133000,134000,135000,136000,137000,138000,139000,140000,141000,142000,143000,144000,145000,146000,147000,148000,149000,150000,151000,152000,153000,154000,155000,156000,157000,158000,159000,160000,161000,162000,163000,164000,165000,166000,167000,168000,169000,170000,171000,172000,173000,174000,175000,176000,177000,178000,179000,180000,181000,182000,183000,184000,185000,186000,187000,188000,189000,190000,191000,192000,193000,194000,195000,196000,197000,198000,199000,200000
NW_003726180_1,Count,1,0,0,0,0,2,0,0,0,0,0,0,0,0,0,0,0,5,5,1,0,0,0,0,1,2,3,0,4,0,0,0,0,0,0,6,0,0,0,0,0,0,2,0,0,0,0,0,2,0,0,2,0,2,0,0,0,0,0,0,0,0,0,0,0,0,0,0,0,0,0,0,0,0,0,0,0,0,0,0,0,0,0,0,0,0,0,1,0,0,0,0,0,0,0,0,0,0,0,0,0,0,0,0,0,0,0,0,0,2,0,0,0,0,0,0,0,0,0,0,0,0,0,0,0,0,0,0,0,0,0,0,0,0,0,0,0,0,0,0,0,0,0,0,0,0,5,0,1,11,0,0,3,3,0,0,0,0,3,0,0,3,4,0,0,3,0,0,0,0,0,0,0,0,0,0,0,0,0,0,0,0,0,0,0,6,3,0,10,0,0,0,0,0,0,2,0,0,3,0,0

	
```

```
		  

		NW_003726183_1, Position,0,1000,2000,3000,4000,5000,6000,7000,8000,9000,10000,11000,12000,13000,14000,15000,16000,17000,18000,19000,20000,21000,22000,23000,24000,25000,26000,27000,28000,29000,30000,31000,32000,33000,34000,35000,36000,37000,38000,39000,40000,41000,42000,43000,44000,45000,46000,47000,48000,49000,50000,51000,52000,53000,54000,55000,56000,57000,58000,59000,60000,61000,62000,63000,64000,65000,66000,67000,68000,69000,70000,71000,72000,73000,74000,75000,76000,77000,78000,79000,80000,81000,82000,83000,84000,85000,86000,87000,88000,89000,90000,91000,92000,93000,94000,95000,96000,97000,98000,99000,100000,101000,102000,103000,104000,105000,106000,107000,108000,109000,110000,111000,112000,113000,114000,115000,116000,117000,118000,119000,120000,121000,122000,123000,124000,125000,126000,127000,128000,129000,130000,131000,132000,133000,134000,135000,136000,137000,138000,139000,140000,141000,142000,143000,144000,145000,146000,147000,148000,149000,150000,151000,152000,153000,154000,155000,156000,157000,158000,159000,160000,161000,162000,163000,164000,165000,166000,167000,168000,169000,170000,171000,172000,173000,174000,175000,176000,177000,178000,179000,180000,181000,182000,183000,184000,185000,186000,187000,188000,189000,190000,191000,192000
NW_003726183_1,Count,0,0,0,0,0,0,0,0,0,0,0,0,0,0,0,0,0,0,3,0,0,0,0,1,4,0,0,1,0,3,3,0,0,0,0,1,2,0,0,0,0,0,0,1,0,2,0,2,0,0,0,1,0,5,0,0,0,0,0,0,0,0,0,0,0,0,0,0,0,0,0,0,0,0,0,0,0,0,0,0,0,0,0,0,0,0,0,0,0,0,0,1,0,3,1,0,0,0,0,3,0,0,0,0,0,1,3,0,0,2,2,0,0,0,0,5,2,0,0,0,0,0,0,0,0,0,0,2,2,0,8,3,0,9,2,2,7,4,0,0,0,2,0,4,10,7,8,7,0,7,0,0,0,0,0,7,0,3,0,2,2,2,0,6,0,0,1,5,0,0,0,2,4,7,0,7,6,6,0,0,11,3,0,7,0,2,1,6,2,5,3,0,0

	
```

```
		  

		NW_003726184_1, Position,0,1000,2000,3000,4000,5000,6000,7000,8000,9000,10000,11000,12000,13000,14000,15000,16000,17000,18000,19000,20000,21000,22000,23000,24000,25000,26000,27000,28000,29000,30000,31000,32000,33000,34000,35000,36000,37000,38000,39000,40000,41000,42000,43000,44000,45000,46000,47000,48000,49000,50000,51000,52000,53000,54000,55000,56000,57000,58000,59000,60000,61000,62000,63000,64000,65000,66000,67000,68000,69000,70000,71000,72000,73000,74000,75000,76000,77000,78000,79000,80000,81000,82000,83000,84000,85000,86000,87000,88000,89000,90000,91000,92000,93000,94000,95000,96000,97000,98000,99000,100000,101000,102000,103000,104000,105000,106000,107000,108000,109000,110000,111000,112000,113000,114000,115000,116000,117000,118000,119000,120000,121000,122000,123000,124000,125000,126000,127000,128000,129000,130000,131000,132000,133000,134000,135000,136000,137000,138000,139000,140000,141000,142000,143000,144000,145000,146000,147000,148000,149000,150000,151000,152000,153000,154000,155000,156000,157000,158000,159000,160000,161000,162000,163000,164000,165000,166000,167000,168000,169000,170000,171000,172000,173000,174000,175000,176000,177000,178000,179000,180000,181000,182000,183000,184000,185000,186000,187000,188000,189000,190000,191000,192000,193000,194000,195000,196000,197000,198000,199000,200000,201000,202000,203000,204000,205000,206000,207000,208000,209000,210000
NW_003726184_1,Count,0,0,0,0,0,0,0,0,0,0,0,0,0,0,0,0,0,0,0,0,0,0,0,0,0,0,0,0,0,0,0,0,0,0,0,0,0,0,0,0,0,0,0,0,0,0,0,0,0,0,0,0,0,0,0,0,0,0,0,0,0,0,0,0,0,0,0,0,0,0,0,0,0,0,0,0,0,0,0,0,0,0,0,0,0,0,0,0,0,0,0,0,0,0,0,0,0,0,0,0,0,0,0,0,0,0,0,0,0,0,0,0,0,0,0,0,0,0,0,0,0,0,0,0,0,0,0,0,0,0,0,0,0,0,0,0,0,0,0,0,0,0,0,0,0,0,0,0,0,0,0,0,0,0,0,0,0,0,0,0,0,0,0,0,0,0,0,0,0,0,0,0,0,4,0,0,0,0,0,0,0,0,0,0,0,0,0,0,0,0,0,0,0,0,0,0,0,0,0,0,0,0,0,0,0,1,0,0,0,0,0

	
```

```
		  

		NW_003726185_1, Position,0,1000,2000,3000,4000,5000,6000,7000,8000,9000,10000,11000,12000,13000,14000,15000,16000,17000,18000,19000,20000,21000,22000,23000,24000,25000,26000,27000,28000,29000,30000,31000,32000,33000,34000,35000,36000,37000,38000,39000,40000,41000,42000,43000,44000,45000,46000,47000,48000,49000,50000,51000,52000,53000,54000,55000,56000,57000,58000,59000,60000,61000,62000,63000,64000,65000,66000,67000,68000,69000,70000,71000,72000,73000,74000,75000,76000,77000,78000,79000,80000,81000,82000,83000,84000,85000,86000,87000,88000,89000,90000,91000,92000,93000,94000,95000,96000,97000,98000,99000,100000,101000,102000,103000,104000,105000,106000,107000,108000,109000,110000,111000,112000,113000,114000,115000,116000,117000,118000,119000,120000,121000,122000,123000,124000,125000,126000,127000,128000,129000,130000,131000,132000,133000,134000,135000,136000,137000,138000,139000,140000,141000,142000,143000,144000,145000,146000,147000,148000,149000,150000,151000,152000,153000,154000,155000,156000,157000,158000,159000,160000,161000,162000,163000,164000,165000,166000,167000,168000,169000,170000,171000,172000,173000,174000,175000,176000,177000,178000,179000,180000,181000,182000,183000,184000,185000,186000,187000,188000,189000,190000,191000,192000,193000,194000,195000,196000,197000
NW_003726185_1,Count,0,0,0,0,0,0,0,0,0,0,0,0,0,0,0,0,0,0,0,0,0,0,0,0,0,0,1,0,0,0,0,0,0,0,0,0,0,1,2,0,0,0,0,0,0,6,0,0,2,0,0,0,0,0,0,0,0,0,0,0,0,1,0,0,0,0,0,0,0,0,0,0,0,0,0,0,3,0,0,0,0,0,0,0,0,0,0,0,3,0,0,0,0,0,0,0,0,0,0,0,0,0,0,0,0,0,0,1,0,0,0,0,0,0,0,0,0,0,0,0,0,0,0,0,0,0,0,0,0,0,0,0,0,0,0,0,0,0,0,0,0,0,0,0,0,0,0,0,0,0,0,0,0,0,0,0,0,0,0,0,0,0,0,0,0,0,0,0,0,0,0,0,0,0,0,0,0,0,0,0,0,0,0,1,0,0,0,0,0,0,0,0,0,0,0,0,0,0

	
```

```
		  

		NW_003726186_1, Position,0,1000,2000,3000,4000,5000,6000,7000,8000,9000,10000,11000,12000,13000,14000,15000,16000,17000,18000,19000,20000,21000,22000,23000,24000,25000,26000,27000,28000,29000,30000,31000,32000,33000,34000,35000,36000,37000,38000,39000,40000,41000,42000,43000,44000,45000,46000,47000,48000,49000,50000,51000,52000,53000,54000,55000,56000,57000,58000,59000,60000,61000,62000,63000,64000,65000,66000,67000,68000,69000,70000,71000,72000,73000,74000,75000,76000,77000,78000,79000,80000,81000,82000,83000,84000,85000,86000,87000,88000,89000,90000,91000,92000,93000,94000,95000,96000,97000,98000,99000,100000,101000,102000,103000,104000,105000,106000,107000,108000,109000,110000,111000,112000,113000,114000,115000,116000,117000,118000,119000,120000,121000,122000,123000,124000,125000,126000,127000,128000,129000,130000,131000,132000,133000,134000,135000,136000,137000,138000,139000,140000,141000,142000,143000,144000,145000,146000,147000,148000,149000,150000,151000,152000,153000,154000,155000,156000,157000,158000,159000,160000,161000,162000,163000,164000,165000,166000,167000,168000,169000,170000,171000,172000,173000,174000,175000,176000,177000,178000,179000,180000,181000,182000,183000,184000,185000,186000,187000,188000,189000,190000,191000,192000,193000,194000,195000,196000
NW_003726186_1,Count,0,0,0,0,0,0,0,0,0,0,0,0,0,0,0,0,0,0,0,0,0,0,0,0,0,0,0,0,0,0,0,0,0,0,0,0,0,0,0,0,0,0,3,1,0,1,0,0,0,0,0,0,0,0,0,0,0,0,0,0,0,0,0,0,0,0,0,0,0,0,0,0,0,0,0,0,0,0,0,0,0,0,10,0,0,3,4,0,1,0,0,0,0,0,4,1,1,0,2,2,4,0,3,0,0,0,0,1,2,3,6,3,5,0,7,8,4,0,0,0,0,0,0,0,0,0,0,0,0,0,1,1,0,0,0,0,0,0,0,0,0,0,0,0,0,0,0,0,0,0,0,0,0,0,0,0,0,0,0,0,0,0,0,0,0,0,0,0,0,0,0,0,0,0,0,0,0,0,0,0,0,0,0,0,0,1,2,0,0,0,0,0,0,4,0,0,1

	
```

```
		  

		NW_003726188_1, Position,0,1000,2000,3000,4000,5000,6000,7000,8000,9000,10000,11000,12000,13000,14000,15000,16000,17000,18000,19000,20000,21000,22000,23000,24000,25000,26000,27000,28000,29000,30000,31000,32000,33000,34000,35000,36000,37000,38000,39000,40000,41000,42000,43000,44000,45000,46000,47000,48000,49000,50000,51000,52000,53000,54000,55000,56000,57000,58000,59000,60000,61000,62000,63000,64000,65000,66000,67000,68000,69000,70000,71000,72000,73000,74000,75000,76000,77000,78000,79000,80000,81000,82000,83000,84000,85000,86000,87000,88000,89000,90000,91000,92000,93000,94000,95000,96000,97000,98000,99000,100000,101000,102000,103000,104000,105000,106000,107000,108000,109000,110000,111000,112000,113000,114000,115000,116000,117000,118000,119000,120000,121000,122000,123000,124000,125000,126000,127000,128000,129000,130000,131000,132000,133000,134000,135000,136000,137000,138000,139000,140000,141000,142000,143000,144000,145000,146000,147000,148000,149000,150000,151000,152000,153000,154000,155000,156000,157000,158000,159000,160000,161000,162000,163000,164000,165000,166000,167000,168000,169000,170000,171000,172000,173000,174000,175000,176000,177000,178000,179000,180000,181000,182000,183000,184000,185000,186000,187000,188000,189000,190000,191000,192000,193000,194000,195000,196000,197000,198000,199000,200000,201000,202000,203000,204000,205000,206000,207000,208000,209000,210000
NW_003726188_1,Count,0,0,0,0,0,0,0,0,0,0,0,0,0,0,0,0,0,0,0,0,0,0,0,0,5,0,0,0,0,0,0,0,0,0,0,0,0,0,0,0,0,0,0,0,0,0,0,0,0,0,0,0,0,0,0,1,3,0,0,0,3,0,0,0,0,0,0,0,0,0,0,0,0,0,0,0,0,0,0,0,0,0,0,0,0,0,0,0,0,0,0,0,0,0,0,0,0,0,0,0,0,0,0,0,0,0,5,1,4,4,0,0,0,0,0,0,0,0,0,0,0,0,0,0,0,0,0,0,0,0,0,0,0,0,0,0,0,0,0,0,0,0,0,0,0,0,0,0,0,0,0,0,0,0,0,0,0,0,0,0,0,0,0,0,0,0,0,0,0,0,0,0,0,0,0,0,0,0,0,0,0,0,0,0,0,0,0,0,0,0,0,0,0,0,0,0,0,0,0,0,0,0,0,0,0,0,0,0,0,0,0

	
```

```
		  

		NW_003726189_1, Position,0,1000,2000,3000,4000,5000,6000,7000,8000,9000,10000,11000,12000,13000,14000,15000,16000,17000,18000,19000,20000,21000,22000,23000,24000,25000,26000,27000,28000,29000,30000,31000,32000,33000,34000,35000,36000,37000,38000,39000,40000,41000,42000,43000,44000,45000,46000,47000,48000,49000,50000,51000,52000,53000,54000,55000,56000,57000,58000,59000,60000,61000,62000,63000,64000,65000,66000,67000,68000,69000,70000,71000,72000,73000,74000,75000,76000,77000,78000,79000,80000,81000,82000,83000,84000,85000,86000,87000,88000,89000,90000,91000,92000,93000,94000,95000,96000,97000,98000,99000,100000,101000,102000,103000,104000,105000,106000,107000,108000,109000,110000,111000,112000,113000,114000,115000,116000,117000,118000,119000,120000,121000,122000,123000,124000,125000,126000,127000,128000,129000,130000,131000,132000,133000,134000,135000,136000,137000,138000,139000,140000,141000,142000,143000,144000,145000,146000,147000,148000,149000,150000,151000,152000,153000,154000,155000,156000,157000,158000,159000,160000,161000,162000,163000,164000,165000,166000,167000,168000,169000,170000,171000,172000,173000,174000,175000,176000,177000
NW_003726189_1,Count,0,0,0,0,0,0,0,0,0,0,0,0,0,0,0,0,0,0,0,0,0,0,0,0,0,0,0,0,0,0,0,0,0,0,0,0,0,0,0,0,0,0,0,0,0,0,0,0,0,0,0,0,0,0,0,0,0,0,0,0,0,0,4,0,0,0,0,0,0,2,0,0,0,0,0,0,0,0,0,0,0,0,0,0,0,0,0,0,0,0,0,0,0,0,0,0,0,0,0,0,0,0,0,0,0,0,0,0,0,0,0,0,0,0,0,0,0,0,0,0,0,0,0,0,0,0,0,0,0,0,0,0,0,0,0,0,0,0,0,0,0,0,4,0,0,0,0,0,0,0,0,0,0,0,0,0,0,0,0,0,0,0,0,0,0,0,0,0,0,0,0,0,0,0,0,0,0,0

	
```

```
		  

		NW_003726191_1, Position,0,1000,2000,3000,4000,5000,6000,7000,8000,9000,10000,11000,12000,13000,14000,15000,16000,17000,18000,19000,20000,21000,22000,23000,24000,25000,26000,27000,28000,29000,30000,31000,32000,33000,34000,35000,36000,37000,38000,39000,40000,41000,42000,43000,44000,45000,46000,47000,48000,49000,50000,51000,52000,53000,54000,55000,56000,57000,58000,59000,60000,61000,62000,63000,64000,65000,66000,67000,68000,69000,70000,71000,72000,73000,74000,75000,76000,77000,78000,79000,80000,81000,82000,83000,84000,85000,86000,87000,88000,89000,90000,91000,92000,93000,94000,95000,96000,97000,98000,99000,100000,101000,102000,103000,104000,105000,106000,107000,108000,109000,110000,111000,112000,113000,114000,115000,116000,117000,118000,119000,120000,121000,122000,123000,124000,125000,126000,127000,128000,129000,130000,131000,132000,133000,134000,135000,136000,137000,138000,139000,140000,141000,142000,143000,144000,145000,146000,147000,148000,149000,150000,151000,152000,153000,154000,155000,156000,157000,158000,159000,160000,161000,162000,163000,164000,165000,166000
NW_003726191_1,Count,0,0,0,0,0,0,0,0,0,0,0,0,0,0,0,0,0,0,0,0,0,0,0,0,0,0,0,0,0,0,0,0,0,0,0,0,0,0,0,0,0,0,0,0,0,0,0,0,0,0,0,0,0,0,0,0,0,0,0,0,0,0,0,0,0,0,0,0,0,0,0,0,0,0,0,0,0,0,0,0,0,0,0,0,0,0,0,0,0,0,0,0,0,0,0,0,0,0,0,0,0,0,0,1,0,0,1,3,0,0,0,0,4,0,0,0,0,0,0,0,0,1,0,0,4,4,0,0,0,0,0,0,0,0,0,3,5,2,2,2,0,0,0,0,0,0,0,0,0,0,0,0,0,0,3,0,0,0,0,0,0,4,0,0,5,0,0

	
```

```
		  

		NW_003726193_1, Position,0,1000,2000,3000,4000,5000,6000,7000,8000,9000,10000,11000,12000,13000,14000,15000,16000,17000,18000,19000,20000,21000,22000,23000,24000,25000,26000,27000,28000,29000,30000,31000,32000,33000,34000,35000,36000,37000,38000,39000,40000,41000,42000,43000,44000,45000,46000,47000,48000,49000,50000,51000,52000,53000,54000,55000,56000,57000,58000,59000,60000,61000,62000,63000,64000,65000,66000,67000,68000,69000,70000,71000,72000,73000,74000,75000,76000,77000,78000,79000,80000,81000,82000,83000,84000,85000,86000,87000,88000,89000,90000,91000,92000,93000,94000,95000,96000,97000,98000,99000,100000,101000,102000,103000,104000,105000,106000,107000,108000,109000,110000,111000,112000,113000,114000,115000,116000,117000,118000,119000,120000,121000,122000,123000,124000,125000,126000,127000,128000,129000,130000,131000,132000,133000,134000,135000,136000,137000,138000,139000,140000,141000,142000,143000,144000,145000,146000,147000,148000,149000,150000,151000,152000,153000,154000,155000,156000,157000
NW_003726193_1,Count,0,0,0,0,0,0,0,0,0,0,0,0,0,0,0,0,0,0,0,0,0,0,0,0,0,0,0,0,0,0,0,0,0,0,0,0,0,0,0,0,0,0,0,0,0,0,0,0,0,0,0,0,0,0,0,0,0,0,0,0,0,0,0,0,0,0,0,0,3,0,0,0,0,0,0,0,2,0,0,2,0,0,0,0,0,0,3,1,5,0,0,0,0,0,0,0,2,0,5,0,0,2,0,0,0,2,2,0,0,0,0,0,0,0,1,6,0,0,0,0,0,0,0,0,3,1,5,0,0,0,0,0,0,0,0,0,7,0,0,2,0,0,0,0,6,0,4,0,0,0,0,0,2,0,0,1,0,0

	
```

```
		  

		NW_003726194_1, Position,0,1000,2000,3000,4000,5000,6000,7000,8000,9000,10000,11000,12000,13000,14000,15000,16000,17000,18000,19000,20000,21000,22000,23000,24000,25000,26000,27000,28000,29000,30000,31000,32000,33000,34000,35000,36000,37000,38000,39000,40000,41000,42000,43000,44000,45000,46000,47000,48000,49000,50000,51000,52000,53000,54000,55000,56000,57000,58000,59000,60000,61000,62000,63000,64000,65000,66000,67000,68000,69000,70000,71000,72000,73000,74000,75000,76000,77000,78000,79000,80000,81000,82000,83000,84000,85000,86000,87000,88000,89000,90000,91000,92000,93000,94000,95000,96000,97000,98000,99000,100000,101000,102000,103000,104000,105000,106000,107000,108000,109000,110000,111000,112000,113000,114000,115000,116000,117000,118000,119000,120000,121000,122000,123000,124000,125000,126000,127000,128000,129000,130000,131000,132000,133000,134000,135000,136000,137000,138000,139000,140000,141000,142000,143000
NW_003726194_1,Count,5,11,8,10,6,17,10,10,7,12,7,12,17,11,7,10,12,11,12,6,9,8,16,15,11,9,3,7,14,2,4,8,11,5,4,4,12,14,14,4,6,9,9,9,7,16,11,5,2,10,11,3,10,4,13,6,12,11,11,5,15,9,1,17,8,9,11,11,11,13,13,8,6,9,9,11,9,7,12,7,7,1,6,9,13,12,14,6,0,4,18,7,13,4,5,2,13,10,12,6,12,12,17,7,12,8,5,10,8,13,11,10,8,6,8,14,10,8,7,11,7,6,11,11,12,10,9,14,12,9,16,2,0,3,11,10,11,4,9,11,7,13,10,9

	
```

```
		  

		NW_003726195_1, Position,0,1000,2000,3000,4000,5000,6000,7000,8000,9000,10000,11000,12000,13000,14000,15000,16000,17000,18000,19000,20000,21000,22000,23000,24000,25000,26000,27000,28000,29000,30000,31000,32000,33000,34000,35000,36000,37000,38000,39000,40000,41000,42000,43000,44000,45000,46000,47000,48000,49000,50000,51000,52000,53000,54000,55000,56000,57000,58000,59000,60000,61000,62000,63000,64000,65000,66000,67000,68000,69000,70000,71000,72000,73000,74000,75000,76000,77000,78000,79000,80000,81000,82000,83000,84000,85000,86000,87000,88000,89000,90000,91000,92000,93000,94000,95000,96000,97000,98000,99000,100000,101000,102000,103000,104000,105000,106000,107000,108000,109000,110000,111000,112000,113000,114000,115000,116000,117000,118000,119000,120000,121000,122000,123000,124000,125000,126000,127000,128000,129000,130000,131000,132000,133000,134000,135000,136000,137000,138000,139000,140000,141000
NW_003726195_1,Count,9,4,11,1,4,12,2,4,4,3,5,8,3,14,7,0,0,0,0,0,0,0,0,0,0,2,0,0,4,10,10,6,4,9,5,5,4,2,10,12,3,9,8,9,8,1,0,0,0,13,15,1,9,8,4,6,13,10,6,7,0,0,0,5,12,11,10,8,7,8,8,8,6,7,10,15,5,7,8,8,11,7,6,3,2,7,6,6,4,11,5,7,10,8,0,0,0,0,7,3,0,0,6,7,7,6,7,6,9,7,4,6,0,0,0,0,0,4,14,7,8,0,0,0,0,0,0,0,4,5,10,9,6,8,8,14,11,2,0,0,0,0

	
```

```
		  

		NW_003726197_1, Position,0,1000,2000,3000,4000,5000,6000,7000,8000,9000,10000,11000,12000,13000,14000,15000,16000,17000,18000,19000,20000,21000,22000,23000,24000,25000,26000,27000,28000,29000,30000,31000,32000,33000,34000,35000,36000,37000,38000,39000,40000,41000,42000,43000,44000,45000,46000,47000,48000,49000,50000,51000,52000,53000,54000,55000,56000,57000,58000,59000,60000,61000,62000,63000,64000,65000,66000,67000,68000,69000,70000,71000,72000,73000,74000,75000,76000,77000,78000,79000,80000,81000,82000,83000,84000,85000,86000,87000,88000,89000,90000,91000,92000,93000,94000,95000,96000,97000,98000,99000,100000,101000,102000,103000,104000,105000,106000,107000,108000,109000,110000,111000,112000,113000,114000,115000,116000,117000,118000,119000,120000,121000,122000,123000,124000,125000,126000,127000,128000,129000,130000,131000,132000,133000,134000,135000,136000,137000,138000,139000,140000,141000,142000
NW_003726197_1,Count,0,0,0,0,0,0,0,0,4,0,1,0,1,5,0,3,6,12,2,3,3,6,4,5,5,5,3,2,3,1,5,2,5,3,3,5,1,6,2,0,3,1,1,0,5,0,2,2,6,1,5,2,2,0,3,0,0,0,0,0,0,0,0,0,0,0,0,0,0,0,0,0,0,0,0,0,0,0,3,0,0,0,0,0,0,0,0,0,0,0,0,0,0,0,0,0,0,0,0,0,0,0,0,0,0,0,0,0,0,0,0,0,0,0,0,0,0,0,0,0,0,0,0,0,0,0,0,0,2,0,0,3,2,4,3,3,9,2,0,0,0,0,0

	
```

```
		  

		NW_003726199_1, Position,0,1000,2000,3000,4000,5000,6000,7000,8000,9000,10000,11000,12000,13000,14000,15000,16000,17000,18000,19000,20000,21000,22000,23000,24000,25000,26000,27000,28000,29000,30000,31000,32000,33000,34000,35000,36000,37000,38000,39000,40000,41000,42000,43000,44000,45000,46000,47000,48000,49000,50000,51000,52000,53000,54000,55000,56000,57000,58000,59000,60000,61000,62000,63000,64000,65000,66000,67000,68000,69000,70000,71000,72000,73000,74000,75000,76000,77000,78000,79000,80000,81000,82000,83000,84000,85000,86000,87000,88000,89000,90000,91000,92000,93000,94000,95000,96000,97000,98000,99000,100000,101000,102000,103000,104000,105000,106000,107000,108000,109000,110000,111000,112000,113000,114000,115000,116000,117000,118000,119000,120000,121000,122000,123000,124000,125000,126000,127000,128000,129000,130000,131000,132000,133000,134000,135000,136000,137000,138000,139000,140000,141000,142000
NW_003726199_1,Count,0,0,0,0,0,0,0,0,0,0,0,0,0,0,0,0,0,0,0,0,0,0,0,0,0,0,0,0,0,0,0,0,0,0,0,0,0,0,0,0,0,0,0,0,0,0,0,0,2,0,0,0,0,4,0,2,0,0,0,0,0,4,0,0,0,0,0,0,0,0,0,0,0,0,0,0,0,0,0,0,0,0,0,0,0,0,0,0,0,0,0,0,0,0,0,0,0,0,0,0,0,0,0,0,0,0,0,0,0,0,0,0,0,0,0,0,0,0,0,0,1,0,0,0,0,0,0,0,0,0,0,0,0,0,0,0,0,0,0,0,0,0,0

	
```

```
		  

		NW_003726200_1, Position,0,1000,2000,3000,4000,5000,6000,7000,8000,9000,10000,11000,12000,13000,14000,15000,16000,17000,18000,19000,20000,21000,22000,23000,24000,25000,26000,27000,28000,29000,30000,31000,32000,33000,34000,35000,36000,37000,38000,39000,40000,41000,42000,43000,44000,45000,46000,47000,48000,49000,50000,51000,52000,53000,54000,55000,56000,57000,58000,59000,60000,61000,62000,63000,64000,65000,66000,67000,68000,69000,70000,71000,72000,73000,74000,75000,76000,77000,78000,79000,80000,81000,82000,83000,84000,85000,86000,87000,88000,89000,90000,91000,92000,93000,94000,95000,96000,97000,98000,99000,100000,101000,102000,103000,104000,105000,106000,107000,108000,109000,110000,111000,112000,113000,114000,115000,116000,117000,118000,119000,120000,121000,122000,123000,124000,125000,126000,127000,128000,129000,130000,131000,132000,133000
NW_003726200_1,Count,0,0,0,0,0,0,0,0,0,0,0,0,0,0,0,0,0,0,0,0,0,0,0,0,0,0,0,0,0,0,0,0,0,0,0,0,0,0,0,0,0,0,0,0,0,0,0,0,0,2,0,5,2,7,4,0,6,0,0,0,3,2,0,5,4,3,5,3,6,4,6,5,0,0,1,8,0,8,6,0,2,0,0,0,0,0,0,4,0,0,0,1,0,0,0,0,0,0,0,0,0,0,0,0,0,0,0,0,0,0,0,0,0,0,0,0,0,0,0,0,0,0,0,0,0,0,0,0,0,0,0,0,0,0

	
```

```
		  

		NW_003726201_1, Position,0,1000,2000,3000,4000,5000,6000,7000,8000,9000,10000,11000,12000,13000,14000,15000,16000,17000,18000,19000,20000,21000,22000,23000,24000,25000,26000,27000,28000,29000,30000,31000,32000,33000,34000,35000,36000,37000,38000,39000,40000,41000,42000,43000,44000,45000,46000,47000,48000,49000,50000,51000,52000,53000,54000,55000,56000,57000,58000,59000,60000,61000,62000,63000,64000,65000,66000,67000,68000,69000,70000,71000,72000,73000,74000,75000,76000,77000,78000,79000,80000,81000,82000,83000,84000,85000,86000,87000,88000,89000,90000,91000,92000,93000,94000,95000,96000,97000,98000,99000,100000,101000,102000,103000,104000,105000,106000,107000,108000,109000,110000,111000,112000,113000,114000,115000,116000,117000,118000,119000,120000,121000,122000,123000,124000,125000,126000,127000,128000,129000
NW_003726201_1,Count,2,5,0,5,2,0,0,0,1,0,0,1,0,0,0,2,0,6,1,1,1,0,4,1,0,0,0,1,0,0,4,3,0,0,0,0,0,0,0,0,0,0,6,0,3,3,0,0,0,0,0,1,0,3,6,0,3,0,0,0,0,0,0,0,5,3,3,7,2,0,0,0,1,0,2,1,0,0,0,5,2,3,0,0,0,0,1,0,0,0,0,0,0,0,2,0,1,3,0,5,3,1,7,8,3,4,4,7,2,0,0,5,0,8,0,3,0,0,0,0,0,0,0,0,1,2,0,0,0,0

	
```

```
		  

		NW_003726203_1, Position,0,1000,2000,3000,4000,5000,6000,7000,8000,9000,10000,11000,12000,13000,14000,15000,16000,17000,18000,19000,20000,21000,22000,23000,24000,25000,26000,27000,28000,29000,30000,31000,32000,33000,34000,35000,36000,37000,38000,39000,40000,41000,42000,43000,44000,45000,46000,47000,48000,49000,50000,51000,52000,53000,54000,55000,56000,57000,58000,59000,60000,61000,62000,63000,64000,65000,66000,67000,68000,69000,70000,71000,72000,73000,74000,75000,76000,77000,78000,79000,80000,81000,82000,83000,84000,85000,86000,87000,88000,89000,90000,91000,92000,93000,94000,95000,96000,97000,98000,99000,100000,101000,102000,103000,104000,105000,106000,107000,108000,109000,110000,111000,112000,113000,114000,115000,116000,117000,118000,119000,120000,121000,122000,123000,124000,125000,126000,127000
NW_003726203_1,Count,2,15,12,3,12,8,8,17,11,7,5,0,5,4,3,0,9,6,5,4,2,1,2,3,2,4,2,3,6,7,1,3,10,5,0,6,6,5,4,3,8,9,7,0,4,3,2,0,5,2,2,3,4,0,4,8,4,3,6,10,1,1,4,1,1,4,6,2,3,1,7,3,3,3,2,1,0,3,0,1,2,2,5,1,3,0,2,6,5,4,1,5,5,9,9,5,2,9,2,3,9,5,12,4,3,3,13,12,10,5,9,11,10,7,10,7,5,9,3,6,10,6,12,6,7,3,7,4

	
```

```
		  

		NW_003726205_1, Position,0,1000,2000,3000,4000,5000,6000,7000,8000,9000,10000,11000,12000,13000,14000,15000,16000,17000,18000,19000,20000,21000,22000,23000,24000,25000,26000,27000,28000,29000,30000,31000,32000,33000,34000,35000,36000,37000,38000,39000,40000,41000,42000,43000,44000,45000,46000,47000,48000,49000,50000,51000,52000,53000,54000,55000,56000,57000,58000,59000,60000,61000,62000,63000,64000,65000,66000,67000,68000,69000,70000,71000,72000,73000,74000,75000,76000,77000,78000,79000,80000,81000,82000,83000,84000,85000,86000,87000,88000,89000,90000,91000,92000,93000,94000,95000,96000,97000,98000,99000,100000,101000,102000,103000,104000,105000,106000,107000,108000,109000,110000,111000,112000,113000,114000,115000,116000,117000,118000,119000,120000,121000,122000,123000,124000,125000,126000,127000,128000,129000,130000,131000
NW_003726205_1,Count,7,6,11,6,9,16,9,9,2,1,9,5,10,9,8,6,4,0,0,0,0,2,4,1,2,1,8,6,8,2,5,8,8,2,9,2,7,12,9,7,15,9,9,9,6,15,5,7,5,5,8,1,8,9,8,3,7,6,3,2,0,0,0,0,0,0,5,3,4,4,2,2,12,4,7,0,0,0,0,0,0,0,0,4,4,4,6,13,14,8,10,3,3,9,10,7,4,2,18,9,4,0,0,0,0,0,0,0,0,0,0,0,0,0,0,0,0,0,0,0,0,0,0,0,0,0,0,0,0,0,2,0

	
```

```
		  

		NW_003726206_1, Position,0,1000,2000,3000,4000,5000,6000,7000,8000,9000,10000,11000,12000,13000,14000,15000,16000,17000,18000,19000,20000,21000,22000,23000,24000,25000,26000,27000,28000,29000,30000,31000,32000,33000,34000,35000,36000,37000,38000,39000,40000,41000,42000,43000,44000,45000,46000,47000,48000,49000,50000,51000,52000,53000,54000,55000,56000,57000,58000,59000,60000,61000,62000,63000,64000,65000,66000,67000,68000,69000,70000,71000,72000,73000,74000,75000,76000,77000,78000,79000,80000,81000,82000,83000,84000,85000,86000,87000,88000,89000,90000,91000,92000,93000,94000,95000,96000,97000,98000,99000,100000,101000,102000,103000,104000,105000,106000,107000,108000,109000,110000,111000,112000,113000,114000,115000,116000,117000,118000,119000,120000,121000,122000,123000,124000,125000,126000,127000,128000,129000,130000,131000,132000,133000
NW_003726206_1,Count,7,0,0,0,0,0,0,0,0,0,0,0,0,0,0,0,0,0,0,0,0,0,0,0,0,0,0,0,0,0,0,0,0,0,0,0,0,0,0,0,0,0,0,0,0,0,0,0,0,0,0,0,0,0,0,0,0,0,0,0,0,0,0,0,0,0,0,0,0,0,0,0,0,0,0,0,0,0,0,0,0,0,0,0,0,4,7,5,7,1,9,11,6,2,9,2,7,11,8,4,8,7,6,5,3,4,1,4,0,0,5,5,0,7,8,1,10,2,8,5,5,6,5,6,3,7,11,6,4,7,8,5,1,0

	
```

```
		  

		NW_003726210_1, Position,0,1000,2000,3000,4000,5000,6000,7000,8000,9000,10000,11000,12000,13000,14000,15000,16000,17000,18000,19000,20000,21000,22000,23000,24000,25000,26000,27000,28000,29000,30000,31000,32000,33000,34000,35000,36000,37000,38000,39000,40000,41000,42000,43000,44000,45000,46000,47000,48000,49000,50000,51000,52000,53000,54000,55000,56000,57000,58000,59000,60000,61000,62000,63000,64000,65000,66000,67000,68000,69000,70000,71000,72000,73000,74000,75000,76000,77000,78000,79000,80000,81000,82000,83000,84000,85000,86000,87000,88000,89000,90000,91000,92000,93000,94000,95000,96000,97000,98000,99000,100000,101000,102000,103000,104000,105000,106000,107000,108000,109000,110000,111000,112000,113000,114000,115000
NW_003726210_1,Count,0,0,0,0,0,0,0,0,0,0,0,0,0,0,0,0,0,0,0,0,0,0,0,0,0,0,0,0,1,3,0,10,13,10,4,5,11,14,11,15,10,8,7,7,0,3,0,0,0,7,3,6,4,4,9,16,8,8,19,14,9,7,11,2,0,0,5,0,0,0,7,0,0,0,0,3,6,2,1,10,11,9,13,16,17,16,15,7,12,16,12,17,19,10,0,0,0,2,0,0,0,0,0,9,10,14,10,5,10,0,16,9,6,6,15,10

	
```

```
		  

		NW_003726213_1, Position,0,1000,2000,3000,4000,5000,6000,7000,8000,9000,10000,11000,12000,13000,14000,15000,16000,17000,18000,19000,20000,21000,22000,23000,24000,25000,26000,27000,28000,29000,30000,31000,32000,33000,34000,35000,36000,37000,38000,39000,40000,41000,42000,43000,44000,45000,46000,47000,48000,49000,50000,51000,52000,53000,54000,55000,56000,57000,58000,59000,60000,61000,62000,63000,64000,65000,66000,67000,68000,69000,70000,71000,72000,73000,74000,75000,76000,77000,78000,79000,80000,81000,82000,83000,84000,85000,86000,87000,88000,89000,90000,91000,92000,93000,94000,95000,96000,97000,98000,99000,100000,101000,102000,103000,104000,105000,106000,107000,108000,109000,110000,111000,112000,113000,114000,115000,116000,117000
NW_003726213_1,Count,0,0,0,0,0,0,0,0,0,0,0,0,0,0,0,0,0,0,0,0,0,0,0,0,0,0,0,0,0,0,0,0,0,0,0,0,0,0,0,0,0,0,0,0,0,0,0,0,0,0,0,0,0,0,0,0,0,0,0,0,0,0,0,0,0,0,0,0,0,0,0,0,0,0,0,0,0,0,0,0,0,0,0,0,0,0,0,0,0,0,0,0,0,0,0,0,0,0,0,0,0,0,0,0,0,0,0,0,0,0,0,0,0,0,0,1,0,0

	
```

```
		  

		NW_003726214_1, Position,0,1000,2000,3000,4000,5000,6000,7000,8000,9000,10000,11000,12000,13000,14000,15000,16000,17000,18000,19000,20000,21000,22000,23000,24000,25000,26000,27000,28000,29000,30000,31000,32000,33000,34000,35000,36000,37000,38000,39000,40000,41000,42000,43000,44000,45000,46000,47000,48000,49000,50000,51000,52000,53000,54000,55000,56000,57000,58000,59000,60000,61000,62000,63000,64000,65000,66000,67000,68000,69000,70000,71000,72000,73000,74000,75000,76000,77000,78000,79000,80000,81000,82000,83000,84000,85000,86000,87000,88000,89000,90000,91000,92000,93000,94000,95000,96000,97000,98000,99000,100000,101000,102000,103000,104000,105000,106000,107000,108000,109000,110000,111000
NW_003726214_1,Count,12,11,7,7,7,7,11,11,3,9,7,7,8,4,7,9,14,13,5,1,7,6,9,8,9,5,8,6,11,7,4,3,4,1,5,5,5,7,6,6,2,3,9,5,9,4,2,15,6,11,5,7,13,10,6,12,6,7,11,3,9,11,4,12,15,9,12,7,10,6,5,9,10,10,2,11,7,9,12,4,8,14,9,5,15,13,15,11,9,7,7,10,5,5,10,6,1,9,5,12,12,15,8,8,8,13,17,6,6,9,4,8

	
```

```
		  

		NW_003726216_1, Position,0,1000,2000,3000,4000,5000,6000,7000,8000,9000,10000,11000,12000,13000,14000,15000,16000,17000,18000,19000,20000,21000,22000,23000,24000,25000,26000,27000,28000,29000,30000,31000,32000,33000,34000,35000,36000,37000,38000,39000,40000,41000,42000,43000,44000,45000,46000,47000,48000,49000,50000,51000,52000,53000,54000,55000,56000,57000,58000,59000,60000,61000,62000,63000,64000,65000,66000,67000,68000,69000,70000,71000,72000,73000,74000,75000,76000,77000,78000,79000,80000,81000,82000,83000,84000,85000,86000,87000,88000,89000,90000,91000,92000,93000,94000,95000,96000,97000,98000,99000,100000,101000,102000,103000,104000,105000,106000,107000,108000,109000,110000,111000,112000
NW_003726216_1,Count,0,0,0,0,0,0,0,0,0,0,0,0,0,0,0,0,0,0,0,1,0,0,0,0,0,0,0,0,0,0,0,0,0,0,0,0,0,0,0,0,0,0,0,0,0,0,0,0,0,0,0,0,0,3,0,0,0,0,0,0,0,0,0,0,0,0,0,0,0,0,0,0,0,0,0,0,0,0,2,0,0,0,1,1,0,0,0,0,0,3,0,0,0,0,0,0,0,0,0,0,0,0,0,0,0,0,0,0,0,0,0,0,0

	
```

```
		  

		NW_003726222_1, Position,0,1000,2000,3000,4000,5000,6000,7000,8000,9000,10000,11000,12000,13000,14000,15000,16000,17000,18000,19000,20000,21000,22000,23000,24000,25000,26000,27000,28000,29000,30000,31000,32000,33000,34000,35000,36000,37000,38000,39000,40000,41000,42000,43000,44000,45000,46000,47000,48000,49000,50000,51000,52000,53000,54000,55000,56000,57000,58000,59000,60000,61000,62000,63000,64000,65000,66000,67000,68000,69000,70000,71000,72000,73000,74000,75000,76000,77000,78000,79000,80000,81000,82000,83000,84000,85000,86000,87000,88000,89000,90000,91000,92000,93000,94000,95000,96000,97000,98000,99000,100000,101000,102000,103000,104000,105000,106000,107000,108000,109000,110000,111000,112000,113000,114000,115000,116000,117000,118000
NW_003726222_1,Count,1,0,0,0,0,0,0,0,0,0,0,0,0,0,0,0,0,0,0,0,0,0,0,0,0,0,0,0,1,0,0,0,4,4,0,3,10,12,12,11,1,3,10,1,0,0,4,0,0,3,3,0,0,8,7,7,4,4,5,6,7,7,6,9,9,7,7,9,13,10,0,0,10,8,3,6,9,14,12,11,14,5,15,7,1,0,1,5,1,6,9,6,0,2,14,11,11,6,4,5,3,7,1,5,5,0,0,3,7,0,9,6,6,11,7,11,8,6,1

	
```

```
		  

		NW_003726224_1, Position,0,1000,2000,3000,4000,5000,6000,7000,8000,9000,10000,11000,12000,13000,14000,15000,16000,17000,18000,19000,20000,21000,22000,23000,24000,25000,26000,27000,28000,29000,30000,31000,32000,33000,34000,35000,36000,37000,38000,39000,40000,41000,42000,43000,44000,45000,46000,47000,48000,49000,50000,51000,52000,53000,54000,55000,56000,57000,58000,59000,60000,61000,62000,63000,64000,65000,66000,67000,68000,69000,70000,71000,72000,73000,74000,75000,76000,77000,78000,79000,80000,81000,82000,83000,84000,85000,86000,87000,88000,89000,90000,91000,92000,93000,94000,95000,96000,97000,98000,99000,100000,101000,102000,103000,104000,105000,106000,107000,108000,109000
NW_003726224_1,Count,0,0,0,0,0,4,0,5,0,0,0,0,0,0,0,0,0,0,0,0,0,0,0,0,0,0,0,0,0,0,0,0,0,0,0,0,0,0,0,0,0,0,0,0,0,0,0,0,0,0,0,0,0,0,0,0,0,0,0,0,0,0,0,0,0,0,0,0,0,0,0,0,0,0,0,0,0,0,0,0,0,0,0,0,0,0,0,0,0,0,0,0,0,0,0,0,0,0,0,0,0,0,0,0,0,0,0,0,1,0

	
```

```
		  

		NW_003726225_1, Position,0,1000,2000,3000,4000,5000,6000,7000,8000,9000,10000,11000,12000,13000,14000,15000,16000,17000,18000,19000,20000,21000,22000,23000,24000,25000,26000,27000,28000,29000,30000,31000,32000,33000,34000,35000,36000,37000,38000,39000,40000,41000,42000,43000,44000,45000,46000,47000,48000,49000,50000,51000,52000,53000,54000,55000,56000,57000,58000,59000,60000,61000,62000,63000,64000,65000,66000,67000,68000,69000,70000,71000,72000,73000,74000,75000,76000,77000,78000,79000,80000,81000,82000,83000,84000,85000,86000,87000,88000,89000,90000,91000,92000,93000,94000,95000,96000,97000,98000,99000,100000,101000,102000,103000,104000,105000,106000,107000,108000,109000,110000,111000,112000,113000,114000,115000,116000,117000,118000,119000,120000,121000,122000,123000,124000
NW_003726225_1,Count,2,0,9,6,4,0,0,0,0,0,0,0,0,0,1,5,5,2,0,0,0,0,0,0,0,3,2,2,0,0,0,0,0,0,0,0,0,0,0,3,0,0,0,0,0,0,0,0,0,0,0,0,0,0,0,0,0,0,0,0,0,0,0,0,0,0,0,0,0,0,0,0,0,0,0,0,0,0,0,0,0,0,0,0,0,4,0,0,0,0,0,0,0,0,0,0,0,0,0,0,0,0,0,0,0,3,0,0,0,0,6,0,0,0,0,0,4,0,0,0,4,0,0,0,0

	
```

```
		  

		NW_003726227_1, Position,0,1000,2000,3000,4000,5000,6000,7000,8000,9000,10000,11000,12000,13000,14000,15000,16000,17000,18000,19000,20000,21000,22000,23000,24000,25000,26000,27000,28000,29000,30000,31000,32000,33000,34000,35000,36000,37000,38000,39000,40000,41000,42000,43000,44000,45000,46000,47000,48000,49000,50000,51000,52000,53000,54000,55000,56000,57000,58000,59000,60000,61000,62000,63000,64000,65000,66000,67000,68000,69000,70000,71000,72000,73000,74000,75000,76000,77000,78000,79000,80000,81000,82000,83000,84000,85000,86000,87000,88000,89000,90000,91000,92000,93000,94000,95000,96000,97000,98000,99000,100000,101000
NW_003726227_1,Count,3,10,8,11,4,5,2,11,13,3,10,5,4,2,6,9,7,3,7,10,3,10,11,5,11,6,7,6,4,1,8,4,5,6,5,9,8,7,2,5,5,5,4,3,4,6,4,1,4,8,9,2,6,3,4,6,1,2,3,8,9,7,8,11,3,5,0,7,1,9,7,4,7,8,4,5,8,14,14,10,11,8,0,1,7,9,7,6,7,5,13,10,7,7,11,4,10,4,7,0,0,0

	
```

```
		  

		NW_003726230_1, Position,0,1000,2000,3000,4000,5000,6000,7000,8000,9000,10000,11000,12000,13000,14000,15000,16000,17000,18000,19000,20000,21000,22000,23000,24000,25000,26000,27000,28000,29000,30000,31000,32000,33000,34000,35000,36000,37000,38000,39000,40000,41000,42000,43000,44000,45000,46000,47000,48000,49000,50000,51000,52000,53000,54000,55000,56000,57000,58000,59000,60000,61000,62000,63000,64000,65000,66000,67000,68000,69000,70000,71000,72000,73000,74000,75000,76000,77000,78000,79000,80000,81000,82000,83000,84000,85000,86000,87000,88000,89000,90000,91000,92000,93000,94000,95000,96000,97000,98000,99000,100000,101000,102000
NW_003726230_1,Count,8,2,2,7,4,7,9,2,11,4,1,6,4,4,0,2,2,1,1,5,5,2,6,4,4,3,4,4,2,7,6,4,6,6,3,2,3,4,4,4,2,4,3,2,3,4,0,0,0,0,0,0,3,0,8,2,0,0,0,0,0,8,1,5,2,1,4,6,3,0,0,0,0,0,0,0,1,2,5,1,7,3,3,3,4,3,4,3,7,4,2,6,2,1,0,7,7,11,2,4,9,2,2

	
```

```
		  

		NW_003726231_1, Position,0,1000,2000,3000,4000,5000,6000,7000,8000,9000,10000,11000,12000,13000,14000,15000,16000,17000,18000,19000,20000,21000,22000,23000,24000,25000,26000,27000,28000,29000,30000,31000,32000,33000,34000,35000,36000,37000,38000,39000,40000,41000,42000,43000,44000,45000,46000,47000,48000,49000,50000,51000,52000,53000,54000,55000,56000,57000,58000,59000,60000,61000,62000,63000,64000,65000,66000,67000,68000,69000,70000,71000,72000,73000,74000,75000,76000,77000,78000,79000,80000,81000,82000,83000,84000,85000,86000,87000,88000,89000,90000,91000,92000,93000,94000,95000
NW_003726231_1,Count,0,0,2,0,0,0,0,0,0,0,6,6,0,0,1,0,1,6,0,3,0,2,6,0,5,0,4,1,0,0,3,0,0,2,0,0,0,0,0,0,0,0,0,0,0,0,0,4,3,2,3,0,0,2,0,1,0,1,3,2,0,0,0,0,0,0,2,0,0,0,0,2,0,3,0,1,0,2,2,0,0,0,4,8,3,0,0,0,0,0,0,0,2,3,5,0

	
```

```
		  

		NW_003726236_1, Position,0,1000,2000,3000,4000,5000,6000,7000,8000,9000,10000,11000,12000,13000,14000,15000,16000,17000,18000,19000,20000,21000,22000,23000,24000,25000,26000,27000,28000,29000,30000,31000,32000,33000,34000,35000,36000,37000,38000,39000,40000,41000,42000,43000,44000,45000,46000,47000,48000,49000,50000,51000,52000,53000,54000,55000,56000,57000,58000,59000,60000,61000,62000,63000,64000,65000,66000,67000,68000,69000,70000,71000,72000,73000,74000,75000,76000,77000,78000,79000,80000,81000,82000,83000,84000,85000
NW_003726236_1,Count,5,12,7,4,4,7,9,11,6,12,9,7,4,5,4,11,9,10,9,13,7,8,13,7,8,6,16,12,11,10,10,12,6,7,9,11,5,11,6,12,9,6,10,6,0,0,0,0,0,0,1,8,6,6,4,8,10,3,6,4,11,2,5,8,4,4,4,7,8,6,3,10,7,6,9,12,9,8,8,3,10,9,8,9,13,1

	
```

```
		  

		NW_003726239_1, Position,0,1000,2000,3000,4000,5000,6000,7000,8000,9000,10000,11000,12000,13000,14000,15000,16000,17000,18000,19000,20000,21000,22000,23000,24000,25000,26000,27000,28000,29000,30000,31000,32000,33000,34000,35000,36000,37000,38000,39000,40000,41000,42000,43000,44000,45000,46000,47000,48000,49000,50000,51000,52000,53000,54000,55000,56000,57000,58000,59000,60000,61000,62000,63000,64000,65000,66000,67000,68000,69000,70000,71000,72000,73000,74000,75000,76000,77000,78000,79000,80000,81000,82000
NW_003726239_1,Count,11,8,8,3,3,2,6,5,6,2,5,3,10,0,0,0,0,0,0,2,3,2,3,3,2,6,6,8,7,2,8,4,4,5,2,7,5,5,3,6,3,4,4,7,8,3,8,5,8,5,7,4,4,5,2,8,6,1,0,0,0,2,1,0,4,0,0,0,0,3,4,2,5,4,6,5,2,7,5,7,3,1,0

	
```

```
		  

		NW_003726241_1, Position,0,1000,2000,3000,4000,5000,6000,7000,8000,9000,10000,11000,12000,13000,14000,15000,16000,17000,18000,19000,20000,21000,22000,23000,24000,25000,26000,27000,28000,29000,30000,31000,32000,33000,34000,35000,36000,37000,38000,39000,40000,41000,42000,43000,44000,45000,46000,47000,48000,49000,50000,51000,52000,53000,54000,55000,56000,57000,58000,59000,60000,61000,62000,63000,64000,65000,66000,67000,68000,69000,70000,71000,72000,73000,74000,75000,76000,77000,78000,79000,80000,81000,82000
NW_003726241_1,Count,0,6,2,0,0,0,0,0,0,0,3,0,0,0,6,5,0,0,5,0,0,1,0,3,0,0,3,0,3,0,0,0,0,0,0,0,0,0,0,0,0,0,0,0,0,0,0,0,0,0,0,0,0,0,0,0,0,0,0,2,0,0,0,0,0,0,0,0,0,0,0,0,0,0,0,0,0,0,0,0,0,0,0

	
```

```
		  

		NW_003726242_1, Position,0,1000,2000,3000,4000,5000,6000,7000,8000,9000,10000,11000,12000,13000,14000,15000,16000,17000,18000,19000,20000,21000,22000,23000,24000,25000,26000,27000,28000,29000,30000,31000,32000,33000,34000,35000,36000,37000,38000,39000,40000,41000,42000,43000,44000,45000,46000,47000,48000,49000,50000,51000,52000,53000,54000,55000,56000,57000,58000,59000,60000,61000,62000,63000,64000,65000,66000,67000,68000,69000,70000,71000,72000,73000,74000,75000,76000,77000,78000,79000,80000,81000,82000,83000,84000,85000,86000,87000,88000,89000,90000,91000,92000,93000,94000,95000,96000,97000,98000,99000,100000,101000,102000
NW_003726242_1,Count,0,0,0,0,0,0,0,0,0,0,0,0,0,0,0,0,0,0,0,0,0,0,0,0,0,0,0,0,0,0,0,0,0,0,0,0,0,0,0,0,0,0,0,0,0,0,0,0,0,0,0,0,0,0,0,0,0,0,0,0,0,0,0,0,2,4,0,0,0,0,0,0,0,0,0,0,0,0,0,0,0,0,0,0,0,0,0,2,0,0,0,0,0,0,0,0,0,0,0,0,0,0,0

	
```

```
		  

		NW_003726244_1, Position,0,1000,2000,3000,4000,5000,6000,7000,8000,9000,10000,11000,12000,13000,14000,15000,16000,17000,18000,19000,20000,21000,22000,23000,24000,25000,26000,27000,28000,29000,30000,31000,32000,33000,34000,35000,36000,37000,38000,39000,40000,41000,42000,43000,44000,45000,46000,47000,48000,49000,50000,51000,52000,53000,54000,55000,56000,57000,58000,59000,60000,61000,62000,63000,64000,65000,66000,67000,68000,69000,70000,71000,72000,73000,74000,75000,76000,77000,78000,79000,80000,81000,82000,83000,84000,85000,86000,87000
NW_003726244_1,Count,0,0,0,0,0,0,0,0,0,0,0,0,0,0,0,0,0,0,0,0,0,0,0,0,0,0,0,0,0,0,0,0,0,0,0,0,0,0,0,0,0,0,0,0,0,0,0,0,0,0,0,0,0,0,0,0,0,0,0,0,0,0,0,0,0,0,0,0,0,0,0,0,0,0,0,0,0,0,0,0,0,0,0,0,2,0,0,0

	
```

```
		  

		NW_003726246_1, Position,0,1000,2000,3000,4000,5000,6000,7000,8000,9000,10000,11000,12000,13000,14000,15000,16000,17000,18000,19000,20000,21000,22000,23000,24000,25000,26000,27000,28000,29000,30000,31000,32000,33000,34000,35000,36000,37000,38000,39000,40000,41000,42000,43000,44000,45000,46000,47000,48000,49000,50000,51000,52000,53000,54000,55000,56000,57000,58000,59000,60000,61000,62000,63000,64000,65000,66000,67000,68000,69000,70000,71000,72000,73000,74000,75000,76000,77000,78000,79000,80000,81000,82000,83000,84000,85000,86000,87000,88000,89000,90000,91000,92000,93000,94000,95000,96000,97000,98000,99000,100000,101000,102000,103000,104000,105000,106000,107000,108000,109000,110000,111000,112000,113000,114000,115000,116000,117000,118000,119000,120000,121000,122000,123000,124000,125000,126000,127000,128000,129000,130000,131000,132000,133000,134000,135000,136000,137000,138000,139000,140000,141000,142000,143000,144000,145000,146000,147000,148000,149000,150000,151000,152000,153000,154000,155000,156000,157000,158000,159000,160000,161000,162000,163000,164000,165000,166000,167000,168000,169000,170000,171000,172000,173000,174000,175000,176000,177000,178000,179000,180000,181000,182000,183000,184000,185000,186000,187000,188000,189000,190000,191000,192000,193000,194000,195000,196000,197000,198000,199000,200000,201000,202000,203000,204000,205000,206000,207000,208000,209000,210000,211000,212000,213000,214000,215000,216000,217000,218000,219000,220000,221000,222000,223000,224000,225000,226000,227000,228000,229000,230000
NW_003726246_1,Count,0,0,0,0,0,0,0,0,0,0,0,0,0,0,0,0,0,0,0,0,0,0,0,0,0,0,0,0,0,0,0,0,0,0,0,0,0,0,0,0,0,0,0,0,0,0,0,0,0,0,0,0,0,0,0,0,0,0,0,0,0,0,0,0,0,0,0,0,0,0,0,0,0,0,0,0,0,0,0,0,0,0,0,0,0,0,0,0,0,0,0,0,0,0,0,0,0,0,0,0,0,0,0,0,0,0,0,0,0,0,0,0,0,0,0,0,0,0,0,0,0,0,0,0,0,0,0,0,0,0,0,0,0,0,0,0,0,0,0,0,0,0,0,0,0,0,0,0,0,0,0,0,12,5,2,5,2,3,7,1,2,1,0,2,7,6,2,1,4,1,6,8,3,1,3,2,5,4,3,3,4,13,4,2,7,5,5,2,4,7,2,2,7,4,7,6,0,0,7,6,5,5,1,3,5,5,5,5,2,8,0,8,3,9,2,5,2,9,6,3,0,7,4,8,9,0,0,0,0,0,0

	
```

```
		  

		NW_003726248_1, Position,0,1000,2000,3000,4000,5000,6000,7000,8000,9000,10000,11000,12000,13000,14000,15000,16000,17000,18000,19000,20000,21000,22000,23000,24000,25000,26000,27000,28000,29000,30000,31000,32000,33000,34000,35000,36000,37000,38000,39000,40000,41000,42000,43000,44000,45000,46000,47000,48000,49000,50000,51000,52000,53000,54000,55000,56000,57000,58000,59000,60000,61000,62000,63000,64000,65000,66000,67000,68000,69000,70000,71000,72000,73000,74000,75000,76000,77000,78000,79000,80000,81000,82000,83000,84000,85000,86000,87000,88000,89000,90000,91000,92000,93000,94000,95000,96000,97000,98000,99000,100000,101000,102000,103000,104000,105000,106000,107000,108000,109000,110000,111000,112000,113000,114000,115000,116000,117000,118000,119000,120000,121000,122000,123000,124000,125000,126000,127000,128000,129000,130000,131000,132000,133000,134000,135000,136000,137000,138000,139000,140000,141000,142000,143000,144000,145000,146000,147000,148000,149000,150000,151000,152000,153000,154000,155000,156000,157000,158000,159000,160000,161000,162000,163000,164000,165000,166000,167000,168000,169000,170000,171000,172000,173000,174000,175000,176000,177000,178000,179000,180000,181000,182000,183000,184000,185000,186000,187000,188000,189000,190000,191000,192000,193000,194000,195000,196000,197000,198000,199000,200000,201000,202000,203000,204000,205000,206000,207000,208000,209000,210000,211000,212000,213000,214000,215000,216000,217000,218000,219000,220000,221000,222000,223000,224000,225000,226000,227000,228000,229000,230000,231000,232000,233000,234000,235000,236000,237000,238000,239000,240000,241000,242000,243000,244000,245000,246000,247000,248000,249000,250000,251000,252000,253000,254000,255000,256000
NW_003726248_1,Count,11,7,11,3,9,5,9,5,6,7,5,3,13,8,8,8,8,6,3,6,4,10,6,7,5,7,6,10,8,7,7,9,8,6,9,6,5,5,8,1,12,6,6,5,6,1,7,6,5,9,5,3,1,4,3,8,4,6,5,8,2,7,4,8,3,5,2,0,0,0,0,0,0,0,0,0,0,0,0,0,0,0,0,0,0,0,0,0,0,0,0,0,0,0,0,0,0,0,0,0,0,0,0,0,0,0,0,0,0,0,0,0,0,0,0,0,0,0,0,0,0,0,0,0,0,0,0,0,0,0,0,0,0,0,0,0,0,0,0,0,0,0,0,0,0,0,0,0,0,0,0,0,0,0,0,0,0,0,0,0,0,0,0,0,0,0,0,0,0,0,0,0,0,0,0,0,0,0,0,0,0,0,0,0,0,0,0,0,0,0,0,0,0,0,0,0,0,0,0,0,0,0,0,0,0,0,0,0,0,0,0,0,0,0,0,0,0,0,0,0,0,0,0,0,0,0,0,0,0,0,0,0,0,0,0,0,0,0,0,0,0,0,0,0,0,0,0,0,0,2,0,0,0,0,0,1,0

	
```

```
		  

		NW_003726251_1, Position,0,1000,2000,3000,4000,5000,6000,7000,8000,9000,10000,11000,12000,13000,14000,15000,16000,17000,18000,19000,20000,21000,22000,23000,24000,25000,26000,27000,28000,29000,30000,31000,32000,33000,34000,35000,36000,37000,38000,39000,40000,41000,42000,43000,44000,45000,46000,47000,48000,49000,50000,51000,52000,53000,54000,55000,56000,57000,58000,59000,60000,61000,62000,63000,64000,65000,66000,67000,68000,69000,70000,71000,72000,73000,74000,75000,76000,77000
NW_003726251_1,Count,0,0,0,0,0,0,0,0,0,0,0,0,0,0,0,0,0,0,0,0,0,0,0,0,0,0,0,0,0,0,0,0,0,0,0,0,0,0,0,0,0,0,0,0,0,0,0,0,0,0,3,0,0,0,0,0,0,0,0,0,0,0,0,0,0,0,0,0,0,0,0,0,0,0,0,0,0,0

	
```

```
		  

		NW_003726252_1, Position,0,1000,2000,3000,4000,5000,6000,7000,8000,9000,10000,11000,12000,13000,14000,15000,16000,17000,18000,19000,20000,21000,22000,23000,24000,25000,26000,27000,28000,29000,30000,31000,32000,33000,34000,35000,36000,37000,38000,39000,40000,41000,42000,43000,44000,45000,46000,47000,48000,49000,50000,51000,52000,53000,54000,55000,56000,57000,58000,59000,60000,61000,62000,63000,64000,65000,66000,67000,68000,69000,70000,71000,72000,73000,74000,75000,76000,77000
NW_003726252_1,Count,1,0,2,0,0,1,4,3,0,2,0,7,6,3,10,7,0,0,7,0,0,0,0,0,0,0,0,0,0,0,0,0,0,0,0,0,0,0,0,0,0,0,0,0,0,0,0,0,0,0,0,2,0,0,0,0,0,3,0,0,0,0,0,0,0,0,0,0,0,0,0,4,6,0,0,0,0,0

	
```

```
		  

		NW_003726253_1, Position,0,1000,2000,3000,4000,5000,6000,7000,8000,9000,10000,11000,12000,13000,14000,15000,16000,17000,18000,19000,20000,21000,22000,23000,24000,25000,26000,27000,28000,29000,30000,31000,32000,33000,34000,35000,36000,37000,38000,39000,40000,41000,42000,43000,44000,45000,46000,47000,48000,49000,50000,51000,52000,53000,54000,55000,56000,57000,58000,59000,60000,61000,62000,63000,64000,65000,66000,67000,68000,69000,70000,71000,72000,73000,74000,75000,76000,77000,78000,79000
NW_003726253_1,Count,8,5,4,0,0,6,0,0,0,0,0,0,2,6,0,1,0,0,0,0,0,0,0,0,0,0,0,0,0,0,0,0,5,1,0,1,3,0,0,1,1,2,3,3,0,0,0,0,0,0,0,0,0,0,0,8,0,5,5,0,0,0,0,0,0,0,0,0,0,0,0,0,0,0,0,0,0,0,0,0

	
```

```
		  

		NW_003726254_1, Position,0,1000,2000,3000,4000,5000,6000,7000,8000,9000,10000,11000,12000,13000,14000,15000,16000,17000,18000,19000,20000,21000,22000,23000,24000,25000,26000,27000,28000,29000,30000,31000,32000,33000,34000,35000,36000,37000,38000,39000,40000,41000,42000,43000,44000,45000,46000,47000,48000,49000,50000,51000,52000,53000,54000,55000,56000,57000,58000,59000,60000,61000,62000,63000,64000,65000,66000,67000,68000,69000,70000,71000,72000,73000,74000,75000,76000,77000,78000,79000,80000,81000,82000,83000,84000,85000,86000,87000,88000,89000,90000
NW_003726254_1,Count,0,0,0,0,0,0,0,0,0,0,0,0,2,0,0,0,0,0,0,0,0,0,0,0,0,0,0,0,0,0,0,0,0,0,0,0,0,0,0,0,0,2,0,0,0,0,0,0,0,0,0,0,0,0,0,0,0,0,0,0,0,0,0,0,0,0,0,0,0,0,0,3,0,0,0,0,0,0,0,0,0,0,0,0,0,0,0,0,0,0,0

	
```

```
		  

		NW_003726256_1, Position,0,1000,2000,3000,4000,5000,6000,7000,8000,9000,10000,11000,12000,13000,14000,15000,16000,17000,18000,19000,20000,21000,22000,23000,24000,25000,26000,27000,28000,29000,30000,31000,32000,33000,34000,35000,36000,37000,38000,39000,40000,41000,42000,43000,44000,45000,46000,47000,48000,49000,50000,51000,52000,53000,54000,55000,56000,57000,58000,59000,60000,61000,62000,63000,64000,65000,66000,67000,68000,69000,70000,71000,72000,73000,74000,75000,76000,77000,78000,79000,80000,81000,82000,83000,84000,85000,86000,87000,88000,89000
NW_003726256_1,Count,0,0,0,0,0,0,0,0,0,0,0,0,0,0,0,0,2,0,0,0,0,0,0,0,0,0,0,0,0,0,0,0,0,0,0,0,0,0,0,0,0,0,0,0,0,0,0,0,0,0,0,0,0,0,0,0,0,0,0,0,0,0,0,0,0,0,0,0,0,0,0,0,0,0,0,0,0,0,0,0,0,0,0,0,0,0,0,0,0,0

	
```

```
		  

		NW_003726257_1, Position,0,1000,2000,3000,4000,5000,6000,7000,8000,9000,10000,11000,12000,13000,14000,15000,16000,17000,18000,19000,20000,21000,22000,23000,24000,25000,26000,27000,28000,29000,30000,31000,32000,33000,34000,35000,36000,37000,38000,39000,40000,41000,42000,43000,44000,45000,46000,47000,48000,49000,50000,51000,52000,53000,54000,55000,56000,57000,58000,59000,60000,61000,62000,63000,64000,65000,66000,67000,68000,69000,70000,71000,72000,73000,74000,75000,76000,77000,78000,79000,80000,81000,82000,83000,84000,85000,86000,87000,88000,89000,90000,91000,92000,93000,94000,95000,96000,97000
NW_003726257_1,Count,2,0,5,6,5,1,7,5,3,5,2,4,7,4,5,6,2,2,8,5,4,5,0,0,0,0,0,0,0,0,0,0,0,0,0,0,0,0,0,0,0,0,0,0,0,0,14,5,9,0,3,5,4,2,3,2,3,4,2,3,3,4,2,2,5,2,5,4,3,5,4,8,5,4,1,1,5,7,3,4,3,3,6,0,1,5,9,4,4,4,1,0,0,0,0,0,0,0

	
```

```
		  

		NW_003726258_1, Position,0,1000,2000,3000,4000,5000,6000,7000,8000,9000,10000,11000,12000,13000,14000,15000,16000,17000,18000,19000,20000,21000,22000,23000,24000,25000,26000,27000,28000,29000,30000,31000,32000,33000,34000,35000,36000,37000,38000,39000,40000,41000,42000,43000,44000,45000,46000,47000,48000,49000,50000,51000,52000,53000,54000,55000,56000,57000,58000,59000,60000,61000,62000,63000,64000,65000,66000,67000,68000,69000,70000,71000,72000,73000,74000,75000,76000,77000,78000,79000
NW_003726258_1,Count,8,19,18,10,8,13,9,18,8,11,11,9,6,10,14,10,13,14,2,1,12,0,2,0,7,13,9,16,7,10,15,9,13,7,12,8,8,12,8,11,9,13,1,11,16,10,4,5,13,18,11,8,7,3,0,0,12,6,8,8,2,12,7,12,14,5,14,12,6,0,15,14,13,12,7,9,15,9,6,8

	
```

```
		  

		NW_003726260_1, Position,0,1000,2000,3000,4000,5000,6000,7000,8000,9000,10000,11000,12000,13000,14000,15000,16000,17000,18000,19000,20000,21000,22000,23000,24000,25000,26000,27000,28000,29000,30000,31000,32000,33000,34000,35000,36000,37000,38000,39000,40000,41000,42000,43000,44000,45000,46000,47000,48000,49000,50000,51000,52000,53000,54000,55000,56000,57000,58000,59000,60000,61000,62000,63000,64000,65000,66000,67000,68000,69000,70000,71000,72000,73000
NW_003726260_1,Count,0,0,0,0,0,0,0,0,0,0,0,0,0,0,0,0,1,0,0,0,0,0,0,0,0,0,0,0,0,0,0,0,0,0,0,0,0,0,0,0,0,0,1,0,0,0,0,0,0,2,0,0,2,0,0,0,0,3,0,0,0,0,0,0,0,0,0,0,0,0,4,1,0,0

	
```

```
		  

		NW_003726262_1, Position,0,1000,2000,3000,4000,5000,6000,7000,8000,9000,10000,11000,12000,13000,14000,15000,16000,17000,18000,19000,20000,21000,22000,23000,24000,25000,26000,27000,28000,29000,30000,31000,32000,33000,34000,35000,36000,37000,38000,39000,40000,41000,42000,43000,44000,45000,46000,47000,48000,49000,50000,51000,52000,53000,54000,55000,56000,57000,58000,59000,60000,61000,62000,63000,64000,65000,66000,67000,68000,69000,70000,71000,72000,73000
NW_003726262_1,Count,0,0,0,0,0,0,0,0,0,0,0,0,0,0,0,0,0,3,2,0,0,0,0,0,0,0,1,0,3,0,0,0,0,0,0,0,0,0,0,0,0,0,0,3,0,0,0,0,0,0,0,0,0,0,0,0,0,0,0,0,0,0,0,0,0,5,1,0,0,0,0,0,5,1

	
```

```
		  

		NW_003726263_1, Position,0,1000,2000,3000,4000,5000,6000,7000,8000,9000,10000,11000,12000,13000,14000,15000,16000,17000,18000,19000,20000,21000,22000,23000,24000,25000,26000,27000,28000,29000,30000,31000,32000,33000,34000,35000,36000,37000,38000,39000,40000,41000,42000,43000,44000,45000,46000,47000,48000,49000,50000,51000,52000,53000,54000,55000,56000,57000,58000,59000,60000,61000,62000,63000,64000,65000,66000,67000,68000,69000,70000,71000,72000,73000,74000,75000,76000,77000,78000,79000,80000,81000,82000,83000,84000,85000,86000,87000,88000,89000,90000,91000,92000,93000,94000,95000,96000,97000,98000,99000,100000,101000,102000,103000,104000
NW_003726263_1,Count,6,0,0,0,0,0,0,0,0,0,0,0,0,0,0,0,0,0,0,0,0,0,0,0,0,0,0,0,0,0,0,0,0,0,0,0,0,0,0,0,0,0,0,0,0,0,0,0,0,0,0,0,0,0,0,0,0,0,0,0,0,0,0,0,0,0,0,0,0,0,0,0,0,0,0,0,0,0,0,0,0,0,0,0,0,0,0,0,0,0,0,0,0,0,0,0,0,0,0,0,0,0,0,0,0

	
```

```
		  

		NW_003726264_1, Position,0,1000,2000,3000,4000,5000,6000,7000,8000,9000,10000,11000,12000,13000,14000,15000,16000,17000,18000,19000,20000,21000,22000,23000,24000,25000,26000,27000,28000,29000,30000,31000,32000,33000,34000,35000,36000,37000,38000,39000,40000,41000,42000,43000,44000,45000,46000,47000,48000,49000,50000,51000,52000,53000,54000,55000,56000,57000,58000,59000,60000,61000,62000,63000,64000,65000,66000,67000,68000,69000,70000,71000,72000,73000,74000,75000,76000,77000,78000
NW_003726264_1,Count,14,11,13,6,11,13,8,14,16,10,11,10,14,11,10,6,14,8,7,14,0,0,0,0,2,9,9,10,11,13,11,3,5,8,5,10,14,13,13,1,10,9,5,2,10,13,9,11,13,8,10,9,12,17,11,2,10,11,11,9,8,9,9,14,10,9,8,0,20,10,8,2,5,15,11,7,15,16,10

	
```

```
		  

		NW_003726268_1, Position,0,1000,2000,3000,4000,5000,6000,7000,8000,9000,10000,11000,12000,13000,14000,15000,16000,17000,18000,19000,20000,21000,22000,23000,24000,25000,26000,27000,28000,29000,30000,31000,32000,33000,34000,35000,36000,37000,38000,39000,40000,41000,42000,43000,44000,45000,46000,47000,48000,49000,50000,51000,52000,53000,54000,55000,56000,57000,58000,59000,60000,61000,62000,63000,64000,65000,66000,67000,68000
NW_003726268_1,Count,4,7,10,11,13,8,10,9,13,11,3,10,4,7,5,8,6,8,6,12,7,6,10,7,13,10,13,12,12,12,6,9,11,12,15,16,8,8,10,4,7,10,10,12,13,9,10,15,13,10,13,7,5,7,7,7,8,7,5,9,12,10,10,10,10,12,8,4,0

	
```

```
		  

		NW_003726273_1, Position,0,1000,2000,3000,4000,5000,6000,7000,8000,9000,10000,11000,12000,13000,14000,15000,16000,17000,18000,19000,20000,21000,22000,23000,24000,25000,26000,27000,28000,29000,30000,31000,32000,33000,34000,35000,36000,37000,38000,39000,40000,41000,42000,43000,44000,45000,46000,47000,48000,49000,50000,51000,52000,53000,54000,55000,56000,57000,58000,59000,60000,61000,62000,63000,64000,65000,66000,67000
NW_003726273_1,Count,5,6,6,6,5,9,9,1,10,7,7,12,4,8,4,7,9,2,4,6,7,5,12,8,8,7,9,5,5,10,9,4,6,5,5,4,5,2,8,2,8,7,8,10,5,11,6,12,9,8,8,10,11,11,6,5,11,10,7,6,2,3,2,8,12,11,8,2

	
```

```
		  

		NW_003726275_1, Position,0,1000,2000,3000,4000,5000,6000,7000,8000,9000,10000,11000,12000,13000,14000,15000,16000,17000,18000,19000,20000,21000,22000,23000,24000,25000,26000,27000,28000,29000,30000,31000,32000,33000,34000,35000,36000,37000,38000,39000,40000,41000,42000,43000,44000,45000,46000,47000,48000,49000,50000,51000,52000,53000,54000,55000,56000,57000,58000,59000,60000,61000,62000,63000,64000,65000,66000,67000
NW_003726275_1,Count,0,0,0,0,0,0,0,0,0,0,0,0,0,0,0,0,0,0,0,0,0,0,0,0,0,0,0,0,0,0,0,0,0,0,0,0,0,0,0,2,2,0,0,3,2,0,0,0,0,0,7,0,0,0,0,2,0,6,0,3,3,1,0,0,0,0,0,0

	
```

```
		  

		NW_003726280_1, Position,0,1000,2000,3000,4000,5000,6000,7000,8000,9000,10000,11000,12000,13000,14000,15000,16000,17000,18000,19000,20000,21000,22000,23000,24000,25000,26000,27000,28000,29000,30000,31000,32000,33000,34000,35000,36000,37000,38000,39000,40000,41000,42000,43000,44000,45000,46000,47000,48000,49000,50000,51000,52000,53000,54000,55000,56000,57000,58000,59000,60000,61000,62000,63000,64000,65000
NW_003726280_1,Count,0,0,0,0,0,0,0,0,0,1,1,0,0,0,0,0,0,0,0,0,0,4,0,0,0,8,1,0,0,1,2,0,0,0,0,0,5,4,0,2,0,0,0,0,0,0,0,2,0,0,0,0,0,0,2,0,0,0,1,0,2,0,4,0,0,0

	
```

```
		  

		NW_003726285_1, Position,0,1000,2000,3000,4000,5000,6000,7000,8000,9000,10000,11000,12000,13000,14000,15000,16000,17000,18000,19000,20000,21000,22000,23000,24000,25000,26000,27000,28000,29000,30000,31000,32000,33000,34000,35000,36000,37000,38000,39000,40000,41000,42000,43000,44000,45000,46000,47000,48000,49000,50000,51000,52000,53000,54000,55000,56000,57000,58000,59000,60000,61000,62000,63000,64000,65000,66000,67000,68000,69000,70000
NW_003726285_1,Count,0,0,0,0,0,0,0,0,1,0,0,0,0,0,0,0,0,0,0,0,0,0,2,0,0,5,8,2,4,9,8,7,5,7,12,11,11,12,0,9,6,12,4,4,9,6,13,6,6,6,4,10,8,8,8,10,6,7,8,10,4,3,8,11,10,9,3,0,0,0,0

	
```

```
		  

		NW_003726287_1, Position,0,1000,2000,3000,4000,5000,6000,7000,8000,9000,10000,11000,12000,13000,14000,15000,16000,17000,18000,19000,20000,21000,22000,23000,24000,25000,26000,27000,28000,29000,30000,31000,32000,33000,34000,35000,36000,37000,38000,39000,40000,41000,42000,43000,44000,45000,46000,47000,48000,49000,50000,51000,52000,53000,54000,55000,56000,57000,58000,59000,60000,61000,62000,63000,64000,65000,66000,67000,68000,69000
NW_003726287_1,Count,0,0,0,0,0,0,0,0,0,0,0,0,0,0,0,0,0,0,0,0,0,0,0,0,0,0,0,0,0,0,0,0,0,0,0,0,2,4,1,0,0,1,1,0,0,4,0,0,0,0,0,0,0,0,0,0,0,0,0,7,0,0,0,0,0,0,0,0,0,0

	
```

```
		  

		NW_003726289_1, Position,0,1000,2000,3000,4000,5000,6000,7000,8000,9000,10000,11000,12000,13000,14000,15000,16000,17000,18000,19000,20000,21000,22000,23000,24000,25000,26000,27000,28000,29000,30000,31000,32000,33000,34000,35000,36000,37000,38000,39000,40000,41000,42000,43000,44000,45000,46000,47000,48000,49000,50000,51000,52000,53000,54000,55000,56000,57000,58000,59000,60000,61000,62000,63000,64000
NW_003726289_1,Count,0,0,0,6,14,6,5,3,4,0,0,0,0,0,4,0,0,0,0,0,0,0,0,0,8,9,10,6,10,6,4,10,4,9,6,12,14,8,16,6,4,1,11,10,8,6,2,14,13,9,17,9,17,14,12,12,8,14,12,9,11,11,11,3,1

	
```

```
		  

		NW_003726290_1, Position,0,1000,2000,3000,4000,5000,6000,7000,8000,9000,10000,11000,12000,13000,14000,15000,16000,17000,18000,19000,20000,21000,22000,23000,24000,25000,26000,27000,28000,29000,30000,31000,32000,33000,34000,35000,36000,37000,38000,39000,40000,41000,42000,43000,44000,45000,46000,47000,48000,49000,50000,51000,52000,53000,54000,55000,56000,57000,58000,59000,60000,61000
NW_003726290_1,Count,0,0,0,0,0,0,0,0,0,0,0,0,0,0,0,0,0,0,0,0,3,2,3,0,0,0,0,0,0,0,0,0,0,0,0,0,0,0,0,8,0,0,0,0,0,0,0,0,0,0,0,0,0,0,3,0,0,0,0,0,0,0

	
```

```
		  

		NW_003726292_1, Position,0,1000,2000,3000,4000,5000,6000,7000,8000,9000,10000,11000,12000,13000,14000,15000,16000,17000,18000,19000,20000,21000,22000,23000,24000,25000,26000,27000,28000,29000,30000,31000,32000,33000,34000,35000,36000,37000,38000,39000,40000,41000,42000,43000,44000,45000,46000,47000,48000,49000,50000,51000,52000,53000,54000,55000,56000,57000,58000,59000,60000,61000
NW_003726292_1,Count,4,9,11,2,9,12,6,10,10,6,8,13,10,15,9,9,9,9,10,6,6,14,4,9,13,14,13,13,7,10,0,10,1,2,11,16,6,2,7,5,0,4,10,6,0,0,0,0,0,0,0,0,0,0,0,0,0,0,0,0,0,0

	
```

```
		  

		NW_003726293_1, Position,0,1000,2000,3000,4000,5000,6000,7000,8000,9000,10000,11000,12000,13000,14000,15000,16000,17000,18000,19000,20000,21000,22000,23000,24000,25000,26000,27000,28000,29000,30000,31000,32000,33000,34000,35000,36000,37000,38000,39000,40000,41000,42000,43000,44000,45000,46000,47000,48000,49000,50000,51000,52000,53000,54000,55000,56000,57000,58000,59000,60000,61000,62000,63000,64000,65000,66000,67000,68000,69000,70000,71000,72000,73000,74000,75000,76000,77000,78000,79000,80000,81000,82000,83000,84000,85000,86000,87000,88000,89000,90000
NW_003726293_1,Count,0,4,0,0,0,0,0,0,0,0,0,0,0,0,0,0,0,0,0,0,0,0,0,0,0,0,0,0,0,0,0,0,0,0,0,0,0,0,0,0,0,0,0,0,0,0,0,0,0,0,0,0,0,0,0,0,0,0,0,0,0,0,0,0,0,0,0,0,0,0,0,0,0,0,0,0,0,0,0,0,0,0,0,0,0,0,0,0,0,0,0

	
```

```
		  

		NW_003726294_1, Position,0,1000,2000,3000,4000,5000,6000,7000,8000,9000,10000,11000,12000,13000,14000,15000,16000,17000,18000,19000,20000,21000,22000,23000,24000,25000,26000,27000,28000,29000,30000,31000,32000,33000,34000,35000,36000,37000,38000,39000,40000,41000,42000,43000,44000,45000,46000,47000,48000,49000,50000,51000,52000,53000,54000,55000,56000,57000,58000,59000,60000,61000
NW_003726294_1,Count,0,0,0,0,0,0,0,0,0,0,0,0,0,0,0,0,0,0,0,0,0,0,0,0,0,0,0,0,0,0,0,0,0,0,0,0,1,0,0,0,0,0,0,0,0,0,0,0,0,0,0,0,0,0,0,0,0,0,0,0,0,0

	
```

```
		  

		NW_003726295_1, Position,0,1000,2000,3000,4000,5000,6000,7000,8000,9000,10000,11000,12000,13000,14000,15000,16000,17000,18000,19000,20000,21000,22000,23000,24000,25000,26000,27000,28000,29000,30000,31000,32000,33000,34000,35000,36000,37000,38000,39000,40000,41000,42000,43000,44000,45000,46000,47000,48000,49000,50000,51000,52000,53000,54000,55000,56000,57000,58000,59000,60000,61000,62000,63000
NW_003726295_1,Count,0,0,0,0,0,0,0,0,0,0,0,0,0,0,0,0,0,0,0,0,0,0,0,0,0,0,0,3,3,0,0,0,0,0,0,0,0,0,0,0,0,0,0,0,0,0,0,0,0,0,0,0,0,0,0,0,0,0,0,0,0,0,0,0

	
```

```
		  

		NW_003726297_1, Position,0,1000,2000,3000,4000,5000,6000,7000,8000,9000,10000,11000,12000,13000,14000,15000,16000,17000,18000,19000,20000,21000,22000,23000,24000,25000,26000,27000,28000,29000,30000,31000,32000,33000,34000,35000,36000,37000,38000,39000,40000,41000,42000,43000,44000,45000,46000,47000,48000,49000,50000,51000,52000,53000,54000,55000,56000,57000,58000,59000,60000,61000,62000,63000,64000,65000,66000,67000,68000,69000,70000,71000,72000,73000,74000,75000,76000,77000,78000,79000,80000,81000,82000,83000,84000,85000,86000,87000,88000
NW_003726297_1,Count,0,0,0,0,0,0,0,0,0,0,0,4,0,5,0,0,0,0,0,0,0,0,0,0,0,0,0,0,0,0,0,0,0,0,0,0,0,0,0,0,0,0,0,0,0,0,0,0,0,0,0,0,0,0,0,0,0,0,0,0,0,3,0,0,0,0,0,0,0,0,0,0,0,0,0,0,0,0,0,2,0,0,0,1,0,1,0,0,0

	
```

```
		  

		NW_003726300_1, Position,0,1000,2000,3000,4000,5000,6000,7000,8000,9000,10000,11000,12000,13000,14000,15000,16000,17000,18000,19000,20000,21000,22000,23000,24000,25000,26000,27000,28000,29000,30000,31000,32000,33000,34000,35000,36000,37000,38000,39000,40000,41000,42000,43000,44000,45000,46000,47000,48000,49000,50000,51000,52000,53000,54000,55000,56000,57000,58000,59000,60000,61000,62000,63000,64000,65000,66000,67000,68000,69000,70000
NW_003726300_1,Count,0,0,0,0,0,0,0,1,0,0,0,0,0,0,0,0,0,0,0,0,0,0,0,0,0,0,0,0,0,0,0,0,0,0,0,0,0,0,0,0,0,0,0,0,0,0,0,0,0,0,0,0,0,0,0,0,0,0,0,0,2,0,0,0,0,0,2,2,0,0,0

	
```

```
		  

		NW_003726301_1, Position,0,1000,2000,3000,4000,5000,6000,7000,8000,9000,10000,11000,12000,13000,14000,15000,16000,17000,18000,19000,20000,21000,22000,23000,24000,25000,26000,27000,28000,29000,30000,31000,32000,33000,34000,35000,36000,37000,38000,39000,40000,41000,42000,43000,44000,45000,46000,47000,48000,49000,50000,51000,52000,53000,54000,55000,56000,57000,58000,59000,60000,61000,62000,63000,64000,65000,66000,67000,68000,69000,70000,71000
NW_003726301_1,Count,0,0,0,0,3,0,0,7,0,0,0,0,0,0,0,0,0,0,0,0,0,0,0,0,0,0,0,0,0,0,0,0,0,0,0,0,0,0,0,0,0,0,0,0,0,0,0,0,0,0,0,0,0,0,0,0,0,0,0,0,0,0,0,0,0,0,0,0,0,0,0,0

	
```

```
		  

		NW_003726304_1, Position,0,1000,2000,3000,4000,5000,6000,7000,8000,9000,10000,11000,12000,13000,14000,15000,16000,17000,18000,19000,20000,21000,22000,23000,24000,25000,26000,27000,28000,29000,30000,31000,32000,33000,34000,35000,36000,37000,38000,39000,40000,41000,42000,43000,44000,45000,46000,47000,48000,49000,50000,51000,52000,53000,54000,55000,56000,57000,58000,59000,60000,61000,62000,63000,64000,65000,66000
NW_003726304_1,Count,0,0,0,0,0,0,0,0,0,0,0,0,0,0,0,0,0,0,0,0,0,0,0,0,0,0,3,0,0,0,0,0,0,0,0,0,0,0,0,0,0,0,0,0,0,0,0,0,0,4,0,0,0,0,3,0,2,6,9,4,7,9,6,7,3,9,6

	
```

```
		  

		NW_003726305_1, Position,0,1000,2000,3000,4000,5000,6000,7000,8000,9000,10000,11000,12000,13000,14000,15000,16000,17000,18000,19000,20000,21000,22000,23000,24000,25000,26000,27000,28000,29000,30000,31000,32000,33000,34000,35000,36000,37000,38000,39000,40000,41000,42000,43000,44000,45000,46000,47000,48000,49000,50000,51000,52000,53000,54000,55000,56000,57000,58000,59000,60000,61000,62000
NW_003726305_1,Count,0,0,2,0,2,0,0,0,0,0,0,3,0,0,2,0,0,1,5,0,0,0,5,4,0,1,2,0,0,0,0,0,0,0,0,0,0,0,3,0,1,0,1,3,3,0,0,0,0,3,0,3,3,0,0,0,0,0,0,0,4,3,0

	
```

```
		  

		NW_003726306_1, Position,0,1000,2000,3000,4000,5000,6000,7000,8000,9000,10000,11000,12000,13000,14000,15000,16000,17000,18000,19000,20000,21000,22000,23000,24000,25000,26000,27000,28000,29000,30000,31000,32000,33000,34000,35000,36000,37000,38000,39000,40000,41000,42000,43000,44000,45000,46000,47000,48000,49000,50000,51000,52000,53000,54000,55000,56000,57000,58000,59000,60000,61000,62000,63000,64000,65000,66000,67000,68000
NW_003726306_1,Count,0,0,0,0,0,0,0,0,0,0,0,0,0,0,0,0,0,0,0,0,0,0,0,0,0,0,0,0,0,0,0,0,0,0,0,0,6,0,0,0,0,0,0,0,0,4,0,0,0,0,0,0,0,0,0,0,0,0,0,0,0,0,1,0,0,0,0,0,0

	
```

```
		  

		NW_003726308_1, Position,0,1000,2000,3000,4000,5000,6000,7000,8000,9000,10000,11000,12000,13000,14000,15000,16000,17000,18000,19000,20000,21000,22000,23000,24000,25000,26000,27000,28000,29000,30000,31000,32000,33000,34000,35000,36000,37000,38000,39000,40000,41000,42000,43000,44000,45000,46000,47000,48000,49000,50000,51000,52000,53000,54000,55000,56000,57000,58000,59000,60000,61000,62000,63000,64000,65000,66000,67000,68000,69000,70000,71000,72000,73000,74000,75000,76000,77000,78000,79000,80000,81000,82000,83000,84000
NW_003726308_1,Count,0,0,0,0,0,0,0,0,0,0,0,0,0,0,0,0,0,0,0,0,0,0,0,0,0,0,0,0,0,0,0,0,0,2,3,0,4,0,9,3,1,3,0,0,0,5,1,7,0,5,6,12,8,8,8,1,0,0,0,0,0,0,0,0,0,0,0,0,0,0,0,0,0,0,0,0,2,0,0,0,0,0,0,0,0

	
```

```
		  

		NW_003726311_1, Position,0,1000,2000,3000,4000,5000,6000,7000,8000,9000,10000,11000,12000,13000,14000,15000,16000,17000,18000,19000,20000,21000,22000,23000,24000,25000,26000,27000,28000,29000,30000,31000,32000,33000,34000,35000,36000,37000,38000,39000,40000,41000,42000,43000,44000,45000,46000,47000,48000,49000,50000,51000,52000,53000,54000,55000,56000
NW_003726311_1,Count,0,0,0,0,0,0,0,0,0,0,0,0,0,0,0,0,0,0,0,0,0,0,0,0,0,0,0,0,0,0,0,0,0,0,0,0,0,0,0,0,0,0,0,0,0,0,0,0,0,0,0,0,0,0,0,0,3

	
```

```
		  

		NW_003726312_1, Position,0,1000,2000,3000,4000,5000,6000,7000,8000,9000,10000,11000,12000,13000,14000,15000,16000,17000,18000,19000,20000,21000,22000,23000,24000,25000,26000,27000,28000,29000,30000,31000,32000,33000,34000,35000,36000,37000,38000,39000,40000,41000,42000,43000,44000,45000,46000,47000,48000,49000,50000,51000,52000,53000,54000,55000,56000,57000,58000,59000
NW_003726312_1,Count,0,0,0,0,0,0,0,0,0,0,0,0,0,0,0,0,0,0,0,0,0,0,0,0,0,0,3,0,0,0,0,3,0,4,0,0,0,0,0,0,0,0,0,0,0,0,0,0,0,0,0,0,0,0,0,0,0,0,0,0

	
```

```
		  

		NW_003726315_1, Position,0,1000,2000,3000,4000,5000,6000,7000,8000,9000,10000,11000,12000,13000,14000,15000,16000,17000,18000,19000,20000,21000,22000,23000,24000,25000,26000,27000,28000,29000,30000,31000,32000,33000,34000,35000,36000,37000,38000,39000,40000,41000,42000,43000,44000,45000,46000,47000,48000,49000,50000,51000,52000,53000,54000
NW_003726315_1,Count,0,0,0,0,0,0,0,0,0,0,0,0,0,0,0,0,0,0,0,0,0,0,0,0,0,0,2,0,0,0,0,0,0,0,0,0,0,0,0,0,0,0,0,0,0,0,0,0,0,0,0,0,0,0,0

	
```

```
		  

		NW_003726318_1, Position,0,1000,2000,3000,4000,5000,6000,7000,8000,9000,10000,11000,12000,13000,14000,15000,16000,17000,18000,19000,20000,21000,22000,23000,24000,25000,26000,27000,28000,29000,30000,31000,32000,33000,34000,35000,36000,37000,38000,39000,40000,41000,42000,43000,44000,45000,46000,47000,48000,49000,50000,51000,52000,53000,54000,55000,56000,57000
NW_003726318_1,Count,0,0,0,0,2,0,0,0,0,0,0,0,0,0,2,1,0,0,0,0,0,0,0,0,0,0,0,2,0,3,0,0,0,0,0,0,0,0,0,0,0,0,0,0,0,0,4,0,0,5,6,5,8,11,2,0,2,0

	
```

```
		  

		NW_003726321_1, Position,0,1000,2000,3000,4000,5000,6000,7000,8000,9000,10000,11000,12000,13000,14000,15000,16000,17000,18000,19000,20000,21000,22000,23000,24000,25000,26000,27000,28000,29000,30000,31000,32000,33000,34000,35000,36000,37000,38000,39000,40000,41000,42000,43000,44000,45000,46000,47000,48000,49000,50000,51000,52000,53000,54000
NW_003726321_1,Count,0,0,0,0,0,0,0,0,0,0,0,0,0,0,0,0,0,0,0,0,0,0,0,1,0,0,0,0,0,0,0,0,0,0,0,0,0,0,0,0,0,0,0,0,0,0,0,0,0,0,0,0,0,0,0

	
```

```
		  

		NW_003726329_1, Position,0,1000,2000,3000,4000,5000,6000,7000,8000,9000,10000,11000,12000,13000,14000,15000,16000,17000,18000,19000,20000,21000,22000,23000,24000,25000,26000,27000,28000,29000,30000,31000,32000,33000,34000,35000,36000,37000,38000,39000,40000,41000,42000,43000,44000,45000,46000,47000,48000,49000,50000
NW_003726329_1,Count,0,0,0,0,0,0,0,0,0,0,0,0,0,0,0,0,0,0,0,0,0,0,0,0,0,0,0,0,0,4,2,0,0,3,0,0,0,0,6,0,0,0,0,0,0,0,0,0,0,0,0

	
```

```
		  

		NW_003726330_1, Position,0,1000,2000,3000,4000,5000,6000,7000,8000,9000,10000,11000,12000,13000,14000,15000,16000,17000,18000,19000,20000,21000,22000,23000,24000,25000,26000,27000,28000,29000,30000,31000,32000,33000,34000,35000,36000,37000,38000,39000,40000,41000,42000,43000,44000,45000,46000,47000,48000,49000,50000,51000,52000,53000
NW_003726330_1,Count,0,1,0,0,0,2,0,0,0,0,0,0,0,0,0,0,0,0,0,0,0,0,0,3,0,0,0,0,0,0,0,0,0,0,0,0,0,0,0,0,0,0,0,0,0,0,0,0,0,0,0,0,0,0

	
```

```
		  

		NW_003726333_1, Position,0,1000,2000,3000,4000,5000,6000,7000,8000,9000,10000,11000,12000,13000,14000,15000,16000,17000,18000,19000,20000,21000,22000,23000,24000,25000,26000,27000,28000,29000,30000,31000,32000,33000,34000,35000,36000,37000,38000,39000,40000,41000,42000,43000,44000,45000,46000,47000,48000,49000,50000,51000,52000,53000,54000,55000,56000,57000,58000,59000,60000,61000,62000,63000
NW_003726333_1,Count,0,0,0,0,0,0,0,0,0,0,2,0,0,0,0,0,0,0,0,0,0,0,0,0,0,0,0,0,0,0,0,0,0,0,0,0,0,0,0,0,0,0,0,0,0,0,0,0,0,0,0,0,0,0,0,0,0,0,0,0,0,0,0,0

	
```

```
		  

		NW_003726334_1, Position,0,1000,2000,3000,4000,5000,6000,7000,8000,9000,10000,11000,12000,13000,14000,15000,16000,17000,18000,19000,20000,21000,22000,23000,24000,25000,26000,27000,28000,29000,30000,31000,32000,33000,34000,35000,36000,37000,38000,39000,40000,41000,42000,43000,44000,45000,46000,47000,48000,49000,50000,51000,52000,53000,54000,55000,56000,57000,58000,59000
NW_003726334_1,Count,0,0,0,0,0,0,3,0,0,0,0,0,4,0,0,0,0,0,0,0,0,0,0,0,0,0,0,0,2,0,0,1,0,0,0,3,6,15,1,0,0,0,0,0,5,13,11,5,10,5,9,8,4,0,12,11,9,4,3,7

	
```

```
		  

		NW_003726336_1, Position,0,1000,2000,3000,4000,5000,6000,7000,8000,9000,10000,11000,12000,13000,14000,15000,16000,17000,18000,19000,20000,21000,22000,23000,24000,25000,26000,27000,28000,29000,30000,31000,32000,33000,34000,35000,36000,37000,38000,39000,40000,41000,42000,43000,44000,45000,46000,47000,48000,49000
NW_003726336_1,Count,0,0,0,0,5,4,6,5,10,15,5,8,6,9,8,10,8,13,15,15,10,1,7,14,11,7,9,5,4,5,4,2,5,0,9,4,5,10,5,4,7,7,0,2,4,0,8,0,0,0

	
```

```
		  

		NW_003726338_1, Position,0,1000,2000,3000,4000,5000,6000,7000,8000,9000,10000,11000,12000,13000,14000,15000,16000,17000,18000,19000,20000,21000,22000,23000,24000,25000,26000,27000,28000,29000,30000,31000,32000,33000,34000,35000,36000,37000,38000,39000,40000,41000,42000,43000,44000,45000,46000,47000,48000,49000,50000,51000,52000,53000,54000,55000,56000,57000
NW_003726338_1,Count,0,0,0,0,0,2,0,0,0,0,0,0,0,0,0,0,0,0,0,0,0,0,0,0,0,0,0,0,0,0,0,0,0,0,0,0,0,0,0,0,0,0,0,0,0,0,0,0,0,0,0,0,0,0,0,0,0,0

	
```

```
		  

		NW_003726342_1, Position,0,1000,2000,3000,4000,5000,6000,7000,8000,9000,10000,11000,12000,13000,14000,15000,16000,17000,18000,19000,20000,21000,22000,23000,24000,25000,26000,27000,28000,29000,30000,31000,32000,33000,34000,35000,36000,37000,38000,39000,40000,41000,42000,43000,44000,45000,46000,47000,48000,49000,50000,51000,52000,53000,54000,55000,56000,57000,58000,59000,60000,61000,62000,63000,64000,65000,66000,67000,68000,69000,70000,71000,72000,73000,74000,75000
NW_003726342_1,Count,1,0,0,0,0,0,0,0,0,0,0,0,0,0,0,0,0,0,0,0,0,0,0,0,0,0,0,0,0,0,0,0,0,0,0,0,0,0,0,0,0,0,0,0,0,0,0,0,0,0,0,0,0,0,0,0,0,0,0,0,0,0,0,0,0,0,0,0,0,0,0,0,0,0,0,0

	
```

```
		  

		NW_003726343_1, Position,0,1000,2000,3000,4000,5000,6000,7000,8000,9000,10000,11000,12000,13000,14000,15000,16000,17000,18000,19000,20000,21000,22000,23000,24000,25000,26000,27000,28000,29000,30000,31000,32000,33000,34000,35000,36000,37000,38000,39000,40000,41000,42000,43000,44000,45000,46000,47000,48000
NW_003726343_1,Count,1,13,7,1,4,4,2,1,7,7,10,9,5,0,4,15,2,4,8,3,0,0,0,0,0,0,0,0,0,0,0,0,0,0,0,0,0,0,0,0,0,0,0,0,0,0,0,0,0

	
```

```
		  

		NW_003726344_1, Position,0,1000,2000,3000,4000,5000,6000,7000,8000,9000,10000,11000,12000,13000,14000,15000,16000,17000,18000,19000,20000,21000,22000,23000,24000,25000,26000,27000,28000,29000,30000,31000,32000,33000,34000,35000,36000,37000,38000,39000,40000,41000,42000,43000,44000,45000,46000,47000
NW_003726344_1,Count,0,0,0,0,7,6,13,13,10,15,7,2,17,8,6,5,11,6,11,4,9,7,10,7,8,7,14,14,11,11,10,11,9,7,6,7,14,10,7,7,1,3,1,0,0,0,0,0

	
```

```
		  

		NW_003726348_1, Position,0,1000,2000,3000,4000,5000,6000,7000,8000,9000,10000,11000,12000,13000,14000,15000,16000,17000,18000,19000,20000,21000,22000,23000,24000,25000,26000,27000,28000,29000,30000,31000,32000,33000,34000,35000,36000,37000,38000,39000,40000,41000,42000,43000,44000,45000,46000,47000,48000,49000,50000,51000,52000,53000
NW_003726348_1,Count,5,10,7,2,0,0,5,5,1,3,3,0,4,12,7,4,4,6,3,0,1,3,8,2,2,11,7,2,1,7,2,2,10,0,0,0,0,0,0,2,0,0,2,0,0,0,0,0,0,0,0,0,0,0

	
```

```
		  

		NW_003726349_1, Position,0,1000,2000,3000,4000,5000,6000,7000,8000,9000,10000,11000,12000,13000,14000,15000,16000,17000,18000,19000,20000,21000,22000,23000,24000,25000,26000,27000,28000,29000,30000,31000,32000,33000,34000,35000,36000,37000,38000,39000,40000,41000,42000,43000,44000,45000,46000,47000,48000,49000,50000,51000,52000,53000,54000,55000,56000,57000,58000,59000,60000,61000,62000,63000,64000,65000,66000,67000,68000,69000,70000,71000,72000,73000
NW_003726349_1,Count,0,0,0,0,0,0,0,0,0,0,0,0,0,0,0,0,0,0,0,0,0,0,0,4,9,10,6,0,0,0,0,0,0,0,0,0,0,0,0,0,0,0,0,0,0,0,0,0,0,0,0,0,0,0,0,0,0,0,0,0,0,0,0,0,0,0,0,0,0,0,0,0,0,0

	
```

```
		  

		NW_003726350_1, Position,0,1000,2000,3000,4000,5000,6000,7000,8000,9000,10000,11000,12000,13000,14000,15000,16000,17000,18000,19000,20000,21000,22000,23000,24000,25000,26000,27000,28000,29000,30000,31000,32000,33000,34000,35000,36000,37000,38000,39000,40000,41000,42000,43000,44000,45000,46000,47000,48000,49000,50000,51000,52000,53000,54000,55000,56000,57000
NW_003726350_1,Count,3,0,0,0,0,0,0,0,0,0,0,0,0,0,0,0,0,0,0,0,0,0,0,0,0,0,0,0,0,0,0,0,0,0,0,0,0,0,0,0,0,0,0,0,0,0,0,0,0,0,0,0,0,0,0,0,0,0

	
```

```
		  

		NW_003726351_1, Position,0,1000,2000,3000,4000,5000,6000,7000,8000,9000,10000,11000,12000,13000,14000,15000,16000,17000,18000,19000,20000,21000,22000,23000,24000,25000,26000,27000,28000,29000,30000,31000,32000,33000,34000,35000,36000,37000,38000,39000,40000,41000,42000,43000,44000,45000,46000,47000,48000,49000,50000,51000,52000,53000,54000,55000,56000,57000,58000,59000,60000,61000,62000,63000,64000,65000,66000,67000,68000,69000,70000
NW_003726351_1,Count,0,0,0,0,0,0,0,0,0,0,0,0,0,0,0,0,0,0,0,0,0,0,0,0,1,0,0,0,0,0,0,0,0,0,0,0,0,0,0,0,0,0,0,0,0,0,0,0,0,1,0,0,0,0,0,0,0,0,0,0,0,0,0,0,0,0,0,0,0,0,0

	
```

```
		  

		NW_003726352_1, Position,0,1000,2000,3000,4000,5000,6000,7000,8000,9000,10000,11000,12000,13000,14000,15000,16000,17000,18000,19000,20000,21000,22000,23000,24000,25000,26000,27000,28000,29000,30000,31000,32000,33000,34000,35000,36000,37000,38000,39000,40000,41000,42000,43000,44000,45000,46000,47000,48000,49000,50000,51000,52000,53000,54000,55000,56000,57000,58000,59000,60000,61000,62000,63000,64000,65000,66000,67000
NW_003726352_1,Count,2,0,0,0,0,0,0,0,0,0,0,0,0,0,0,0,0,0,0,0,0,0,0,0,0,0,0,0,0,0,0,0,0,0,0,0,0,0,0,0,0,0,0,0,0,0,0,0,0,0,0,0,0,0,0,0,0,0,0,0,0,0,0,0,0,0,0,0

	
```

```
		  

		NW_003726353_1, Position,0,1000,2000,3000,4000,5000,6000,7000,8000,9000,10000,11000,12000,13000,14000,15000,16000,17000,18000,19000,20000,21000,22000,23000,24000,25000,26000,27000,28000,29000,30000,31000,32000,33000,34000,35000,36000,37000,38000,39000,40000,41000,42000,43000,44000,45000,46000,47000,48000
NW_003726353_1,Count,5,0,4,4,0,0,0,0,0,0,0,0,0,1,0,0,11,0,3,0,0,1,0,0,0,5,3,0,0,0,1,0,2,0,1,2,0,1,0,0,0,0,0,1,2,4,0,0,0

	
```

```
		  

		NW_003726356_1, Position,0,1000,2000,3000,4000,5000,6000,7000,8000,9000,10000,11000,12000,13000,14000,15000,16000,17000,18000,19000,20000,21000,22000,23000,24000,25000,26000,27000,28000,29000,30000,31000,32000,33000,34000,35000,36000,37000,38000,39000,40000,41000,42000,43000,44000,45000,46000,47000,48000,49000,50000,51000,52000,53000,54000,55000,56000,57000,58000
NW_003726356_1,Count,5,2,0,0,5,4,4,6,0,0,0,2,8,7,6,5,10,10,5,3,4,6,4,7,8,1,7,0,9,4,0,0,0,0,0,0,0,0,0,0,0,6,0,0,4,9,2,0,0,9,7,0,3,4,2,7,6,2,4

	
```

```
		  

		NW_003726357_1, Position,0,1000,2000,3000,4000,5000,6000,7000,8000,9000,10000,11000,12000,13000,14000,15000,16000,17000,18000,19000,20000,21000,22000,23000,24000,25000,26000,27000,28000,29000,30000,31000,32000,33000,34000,35000,36000,37000,38000,39000,40000,41000,42000,43000,44000,45000
NW_003726357_1,Count,0,0,0,0,0,0,0,0,0,0,3,2,0,0,0,0,0,0,0,0,0,0,0,0,0,0,0,0,0,0,0,0,0,0,0,0,0,2,0,0,1,0,0,0,2,0

	
```

```
		  

		NW_003726358_1, Position,0,1000,2000,3000,4000,5000,6000,7000,8000,9000,10000,11000,12000,13000,14000,15000,16000,17000,18000,19000,20000,21000,22000,23000,24000,25000,26000,27000,28000,29000,30000,31000,32000,33000,34000,35000,36000,37000,38000,39000,40000,41000,42000,43000,44000,45000,46000
NW_003726358_1,Count,0,0,0,0,0,0,0,0,0,0,0,1,1,0,0,2,0,0,0,0,0,0,0,0,0,0,0,0,0,0,0,0,0,0,0,0,6,0,0,0,0,0,0,0,0,0,0

	
```

```
		  

		NW_003726361_1, Position,0,1000,2000,3000,4000,5000,6000,7000,8000,9000,10000,11000,12000,13000,14000,15000,16000,17000,18000,19000,20000,21000,22000,23000,24000,25000,26000,27000,28000,29000,30000,31000,32000,33000,34000,35000,36000,37000,38000,39000,40000,41000,42000,43000,44000,45000
NW_003726361_1,Count,0,0,0,0,0,0,0,0,0,0,0,0,0,0,0,0,0,0,0,0,0,0,0,0,0,0,0,0,0,0,0,0,2,0,0,0,0,0,0,0,0,0,0,0,0,0

	
```

```
		  

		NW_003726362_1, Position,0,1000,2000,3000,4000,5000,6000,7000,8000,9000,10000,11000,12000,13000,14000,15000,16000,17000,18000,19000,20000,21000,22000,23000,24000,25000,26000,27000,28000,29000,30000,31000,32000,33000,34000,35000,36000,37000,38000,39000,40000,41000,42000,43000,44000,45000,46000,47000
NW_003726362_1,Count,0,0,0,0,0,0,0,0,0,0,0,0,0,0,0,0,0,0,0,0,0,0,0,0,0,0,0,0,0,2,0,0,0,0,0,0,0,0,0,0,0,0,0,0,0,0,0,0

	
```

```
		  

		NW_003726363_1, Position,0,1000,2000,3000,4000,5000,6000,7000,8000,9000,10000,11000,12000,13000,14000,15000,16000,17000,18000,19000,20000,21000,22000,23000,24000,25000,26000,27000,28000,29000,30000,31000,32000,33000,34000,35000,36000,37000,38000,39000,40000,41000,42000,43000,44000
NW_003726363_1,Count,0,0,0,0,0,0,0,0,0,0,0,0,0,0,0,0,0,0,0,0,0,0,0,0,1,0,0,0,0,0,0,0,0,0,0,0,0,0,0,0,0,0,0,0,0

	
```

```
		  

		NW_003726364_1, Position,0,1000,2000,3000,4000,5000,6000,7000,8000,9000,10000,11000,12000,13000,14000,15000,16000,17000,18000,19000,20000,21000,22000,23000,24000,25000,26000,27000,28000,29000,30000,31000,32000,33000,34000,35000,36000,37000,38000,39000,40000,41000,42000,43000,44000,45000,46000,47000,48000,49000,50000,51000,52000,53000,54000,55000,56000,57000,58000,59000,60000,61000,62000,63000,64000,65000,66000,67000,68000,69000,70000,71000,72000,73000,74000,75000,76000,77000,78000,79000,80000,81000,82000,83000,84000
NW_003726364_1,Count,0,0,0,0,0,0,0,0,0,0,0,0,0,0,0,0,0,0,0,0,0,0,0,0,0,0,0,0,3,0,0,0,0,0,0,0,0,0,0,0,0,0,0,0,0,0,0,0,0,0,0,0,0,0,0,0,0,0,0,0,0,0,0,0,0,0,0,0,0,0,0,0,0,0,0,0,0,0,0,0,0,0,0,0,0

	
```

```
		  

		NW_003726365_1, Position,0,1000,2000,3000,4000,5000,6000,7000,8000,9000,10000,11000,12000,13000,14000,15000,16000,17000,18000,19000,20000,21000,22000,23000,24000,25000,26000,27000,28000,29000,30000,31000,32000,33000,34000,35000,36000,37000,38000,39000,40000,41000,42000,43000,44000,45000,46000,47000,48000,49000,50000,51000
NW_003726365_1,Count,0,0,0,0,0,0,0,0,0,0,0,0,0,0,0,0,0,0,0,1,0,0,0,0,0,0,0,3,0,0,8,5,0,5,1,4,0,0,0,6,2,0,0,1,0,0,0,0,0,0,0,0

	
```

```
		  

		NW_003726367_1, Position,0,1000,2000,3000,4000,5000,6000,7000,8000,9000,10000,11000,12000,13000,14000,15000,16000,17000,18000,19000,20000,21000,22000,23000,24000,25000,26000,27000,28000,29000,30000,31000,32000,33000,34000,35000,36000,37000,38000,39000,40000,41000,42000,43000,44000,45000,46000,47000,48000,49000,50000
NW_003726367_1,Count,0,0,0,0,0,0,0,0,0,0,0,0,0,0,0,0,3,4,0,0,0,0,0,0,0,0,0,0,0,0,0,0,0,0,0,0,0,0,0,0,0,0,0,0,0,0,0,0,0,0,0

	
```

```
		  

		NW_003726369_1, Position,0,1000,2000,3000,4000,5000,6000,7000,8000,9000,10000,11000,12000,13000,14000,15000,16000,17000,18000,19000,20000,21000,22000,23000,24000,25000,26000,27000,28000,29000,30000,31000,32000,33000,34000,35000,36000,37000,38000,39000,40000,41000,42000,43000,44000
NW_003726369_1,Count,0,0,0,0,0,0,0,0,0,0,0,0,0,0,0,0,0,0,0,0,0,0,0,6,0,0,0,0,0,0,0,0,0,0,0,0,0,0,0,0,0,0,0,0,0

	
```

```
		  

		NW_003726370_1, Position,0,1000,2000,3000,4000,5000,6000,7000,8000,9000,10000,11000,12000,13000,14000,15000,16000,17000,18000,19000,20000,21000,22000,23000,24000,25000,26000,27000,28000,29000,30000,31000,32000,33000,34000,35000,36000,37000,38000,39000,40000,41000,42000,43000
NW_003726370_1,Count,3,0,6,3,6,7,5,0,1,4,8,7,1,7,7,6,1,2,4,0,2,8,3,3,3,4,7,7,4,2,3,5,2,7,4,2,5,4,6,3,3,0,4,2

	
```

```
		  

		NW_003726378_1, Position,0,1000,2000,3000,4000,5000,6000,7000,8000,9000,10000,11000,12000,13000,14000,15000,16000,17000,18000,19000,20000,21000,22000,23000,24000,25000,26000,27000,28000,29000,30000,31000,32000,33000,34000,35000,36000,37000,38000,39000,40000,41000,42000
NW_003726378_1,Count,0,0,0,0,0,0,0,0,0,0,0,0,0,0,0,0,0,0,0,0,0,0,0,0,0,0,0,0,0,0,0,0,2,0,0,0,0,0,0,0,0,1,1

	
```

```
		  

		NW_003726381_1, Position,0,1000,2000,3000,4000,5000,6000,7000,8000,9000,10000,11000,12000,13000,14000,15000,16000,17000,18000,19000,20000,21000,22000,23000,24000,25000,26000,27000,28000,29000,30000,31000,32000,33000,34000,35000,36000,37000,38000,39000,40000,41000,42000,43000,44000,45000,46000,47000,48000,49000,50000,51000,52000,53000,54000,55000,56000,57000,58000,59000,60000,61000,62000,63000,64000,65000,66000,67000,68000,69000
NW_003726381_1,Count,0,0,0,0,0,0,0,0,0,0,0,0,0,0,0,0,0,0,0,0,0,0,0,0,0,0,0,0,0,0,0,0,0,0,0,0,0,0,0,0,0,0,0,0,0,0,0,0,0,0,0,0,0,0,0,4,3,5,0,0,0,0,0,0,0,0,0,0,0,0

	
```

```
		  

		NW_003726386_1, Position,0,1000,2000,3000,4000,5000,6000,7000,8000,9000,10000,11000,12000,13000,14000,15000,16000,17000,18000,19000,20000,21000,22000,23000,24000,25000,26000,27000,28000,29000,30000,31000,32000,33000,34000,35000,36000,37000,38000,39000,40000,41000,42000,43000
NW_003726386_1,Count,0,0,0,4,9,9,0,0,0,0,0,0,0,0,0,0,15,9,12,9,8,4,2,2,3,9,2,2,10,9,12,10,3,15,3,0,7,7,18,10,8,0,0,0

	
```

```
		  

		NW_003726387_1, Position,0,1000,2000,3000,4000,5000,6000,7000,8000,9000,10000,11000,12000,13000,14000,15000,16000,17000,18000,19000,20000,21000,22000,23000,24000,25000,26000,27000,28000,29000,30000,31000,32000,33000,34000,35000,36000,37000,38000,39000,40000,41000,42000,43000,44000,45000,46000,47000,48000,49000,50000,51000,52000,53000,54000,55000,56000,57000
NW_003726387_1,Count,0,0,0,0,0,0,0,0,0,0,0,0,0,0,0,0,0,0,0,0,0,0,0,0,0,0,0,0,0,0,0,0,0,0,0,0,0,0,0,0,0,0,0,1,0,0,0,0,0,0,0,0,0,0,0,0,0,0

	
```

```
		  

		NW_003726392_1, Position,0,1000,2000,3000,4000,5000,6000,7000,8000,9000,10000,11000,12000,13000,14000,15000,16000,17000,18000,19000,20000,21000,22000,23000,24000,25000,26000,27000,28000,29000,30000,31000,32000,33000,34000,35000,36000,37000,38000,39000,40000
NW_003726392_1,Count,0,6,5,5,4,5,3,3,3,3,8,3,8,5,8,3,7,5,1,6,4,2,4,9,5,6,2,2,5,9,6,6,5,12,9,3,7,0,0,0,0

	
```

```
		  

		NW_003726394_1, Position,0,1000,2000,3000,4000,5000,6000,7000,8000,9000,10000,11000,12000,13000,14000,15000,16000,17000,18000,19000,20000,21000,22000,23000,24000,25000,26000,27000,28000,29000,30000,31000,32000,33000,34000,35000,36000,37000,38000,39000,40000,41000
NW_003726394_1,Count,0,0,0,0,0,0,0,0,0,0,0,0,0,0,0,0,0,0,0,0,1,0,0,0,0,0,0,0,0,0,0,0,0,0,0,0,0,0,0,0,0,0

	
```

```
		  

		NW_003726396_1, Position,0,1000,2000,3000,4000,5000,6000,7000,8000,9000,10000,11000,12000,13000,14000,15000,16000,17000,18000,19000,20000,21000,22000,23000,24000,25000,26000,27000,28000,29000,30000,31000,32000,33000,34000,35000,36000,37000,38000,39000,40000,41000,42000,43000,44000,45000,46000
NW_003726396_1,Count,0,0,0,0,0,0,0,4,2,0,0,0,1,0,0,0,2,0,0,0,3,0,0,0,2,0,0,0,0,0,0,0,0,0,0,0,0,0,0,0,0,0,0,0,0,0,0

	
```

```
		  

		NW_003726399_1, Position,0,1000,2000,3000,4000,5000,6000,7000,8000,9000,10000,11000,12000,13000,14000,15000,16000,17000,18000,19000,20000,21000,22000,23000,24000,25000,26000,27000,28000,29000,30000,31000,32000,33000,34000,35000,36000,37000,38000,39000,40000
NW_003726399_1,Count,0,0,0,0,0,0,0,0,0,0,1,0,0,0,0,0,0,0,0,0,0,0,0,1,0,3,1,0,0,0,0,0,0,0,0,0,0,0,0,0,0

	
```

```
		  

		NW_003726401_1, Position,0,1000,2000,3000,4000,5000,6000,7000,8000,9000,10000,11000,12000,13000,14000,15000,16000,17000,18000,19000,20000,21000,22000,23000,24000,25000,26000,27000,28000,29000,30000,31000,32000,33000,34000,35000,36000,37000,38000,39000,40000,41000,42000,43000,44000,45000,46000
NW_003726401_1,Count,0,0,0,0,0,0,0,0,0,0,0,0,0,0,6,0,0,0,0,0,0,0,0,0,0,0,0,0,0,0,0,0,0,0,0,0,0,0,0,0,0,0,0,0,0,0,0

	
```

```
		  

		NW_003726405_1, Position,0,1000,2000,3000,4000,5000,6000,7000,8000,9000,10000,11000,12000,13000,14000,15000,16000,17000,18000,19000,20000,21000,22000,23000,24000,25000,26000,27000,28000,29000,30000,31000,32000,33000,34000,35000,36000,37000,38000,39000
NW_003726405_1,Count,11,12,1,9,13,5,4,4,6,4,6,2,7,4,7,3,13,9,4,5,10,6,8,3,7,7,6,8,3,2,2,6,7,10,0,8,2,2,2,0

	
```

```
		  

		NW_003726407_1, Position,0,1000,2000,3000,4000,5000,6000,7000,8000,9000,10000,11000,12000,13000,14000,15000,16000,17000,18000,19000,20000,21000,22000,23000,24000,25000,26000,27000,28000,29000,30000,31000,32000,33000,34000,35000,36000,37000,38000,39000,40000,41000,42000,43000,44000,45000,46000,47000
NW_003726407_1,Count,0,0,2,0,0,0,0,0,0,0,0,0,0,9,18,3,0,7,1,2,1,0,0,0,0,0,0,0,0,0,0,0,0,0,0,0,0,0,0,0,0,0,0,0,0,0,0,0

	
```

```
		  

		NW_003726410_1, Position,0,1000,2000,3000,4000,5000,6000,7000,8000,9000,10000,11000,12000,13000,14000,15000,16000,17000,18000,19000,20000,21000,22000,23000,24000,25000,26000,27000,28000,29000,30000,31000,32000,33000,34000,35000,36000,37000,38000,39000,40000,41000,42000,43000,44000,45000,46000,47000,48000,49000,50000,51000,52000,53000,54000,55000,56000,57000,58000,59000,60000,61000,62000,63000,64000
NW_003726410_1,Count,0,0,0,0,0,0,0,0,0,0,0,1,1,0,0,0,0,0,0,0,0,0,0,0,0,0,0,0,0,0,0,0,0,0,0,0,0,12,5,6,3,8,0,0,0,0,0,0,0,0,0,0,0,0,0,0,0,0,0,0,0,0,0,0,0

	
```

```
		  

		NW_003726416_1, Position,0,1000,2000,3000,4000,5000,6000,7000,8000,9000,10000,11000,12000,13000,14000,15000,16000,17000,18000,19000,20000,21000,22000,23000,24000,25000,26000,27000,28000,29000,30000,31000,32000,33000,34000,35000,36000,37000,38000,39000,40000,41000
NW_003726416_1,Count,0,2,0,6,0,0,4,0,2,0,0,0,0,3,0,0,0,0,0,0,3,0,0,0,0,0,0,0,0,0,0,0,0,0,0,0,0,0,0,0,0,0

	
```

```
		  

		NW_003726419_1, Position,0,1000,2000,3000,4000,5000,6000,7000,8000,9000,10000,11000,12000,13000,14000,15000,16000,17000,18000,19000,20000,21000,22000,23000,24000,25000,26000,27000,28000,29000,30000,31000,32000,33000,34000,35000,36000,37000,38000,39000,40000,41000,42000,43000,44000,45000,46000,47000,48000,49000,50000,51000,52000,53000,54000,55000,56000,57000,58000,59000
NW_003726419_1,Count,0,0,0,0,0,0,0,0,0,0,0,0,0,0,0,0,0,0,0,0,0,0,0,0,0,0,0,0,0,0,0,0,0,0,0,0,0,0,0,0,0,0,0,0,0,0,0,0,0,0,0,0,0,0,1,0,0,0,0,0

	
```

```
		  

		NW_003726422_1, Position,0,1000,2000,3000,4000,5000,6000,7000,8000,9000,10000,11000,12000,13000,14000,15000,16000,17000,18000,19000,20000,21000,22000,23000,24000,25000,26000,27000,28000,29000,30000,31000,32000,33000,34000,35000,36000,37000,38000,39000,40000,41000,42000,43000,44000,45000,46000,47000,48000,49000,50000,51000,52000
NW_003726422_1,Count,0,0,0,0,0,0,0,0,0,0,2,0,0,0,0,0,0,0,0,0,0,0,0,0,0,0,0,0,0,0,0,0,0,0,0,0,0,0,0,0,0,0,3,0,0,0,0,0,0,0,0,0,0

	
```

```
		  

		NW_003726424_1, Position,0,1000,2000,3000,4000,5000,6000,7000,8000,9000,10000,11000,12000,13000,14000,15000,16000,17000,18000,19000,20000,21000,22000,23000,24000,25000,26000,27000,28000,29000,30000,31000,32000,33000,34000,35000,36000,37000,38000,39000,40000,41000,42000,43000,44000,45000,46000,47000,48000,49000,50000,51000,52000,53000,54000,55000,56000,57000,58000,59000,60000,61000,62000,63000,64000,65000,66000,67000,68000,69000
NW_003726424_1,Count,0,0,0,0,0,0,0,0,0,0,0,0,0,0,0,0,0,0,0,0,0,0,0,0,0,0,1,0,0,0,0,0,0,0,0,0,0,0,0,0,0,0,0,0,0,0,0,0,0,0,0,0,0,0,0,0,0,0,0,0,0,0,0,0,0,0,0,0,0,0

	
```

```
		  

		NW_003726425_1, Position,0,1000,2000,3000,4000,5000,6000,7000,8000,9000,10000,11000,12000,13000,14000,15000,16000,17000,18000,19000,20000,21000,22000,23000,24000,25000,26000,27000,28000,29000,30000,31000,32000,33000,34000,35000,36000,37000,38000,39000,40000,41000,42000,43000,44000
NW_003726425_1,Count,0,0,0,0,0,0,0,0,0,0,0,0,0,0,0,0,0,0,0,0,1,0,0,0,0,0,0,0,0,0,0,0,0,3,0,0,0,5,0,0,0,0,0,0,0

	
```

```
		  

		NW_003726427_1, Position,0,1000,2000,3000,4000,5000,6000,7000,8000,9000,10000,11000,12000,13000,14000,15000,16000,17000,18000,19000,20000,21000,22000,23000,24000,25000,26000,27000,28000,29000,30000,31000,32000,33000,34000,35000,36000
NW_003726427_1,Count,3,0,0,0,0,0,0,0,0,0,0,0,0,0,0,0,0,0,0,0,3,0,0,0,0,2,0,0,0,0,4,2,6,6,6,3,4

	
```

```
		  

		NW_003726435_1, Position,0,1000,2000,3000,4000,5000,6000,7000,8000,9000,10000,11000,12000,13000,14000,15000,16000,17000,18000,19000,20000,21000,22000,23000,24000,25000,26000,27000,28000,29000,30000,31000,32000,33000,34000,35000,36000,37000
NW_003726435_1,Count,0,0,0,0,0,0,0,0,0,0,4,0,0,0,0,0,0,0,0,9,23,7,0,0,0,24,11,21,22,22,18,0,0,0,0,0,0,0

	
```

```
		  

		NW_003726437_1, Position,0,1000,2000,3000,4000,5000,6000,7000,8000,9000,10000,11000,12000,13000,14000,15000,16000,17000,18000,19000,20000,21000,22000,23000,24000,25000,26000,27000,28000,29000,30000,31000,32000,33000,34000,35000,36000,37000,38000,39000,40000,41000
NW_003726437_1,Count,0,2,7,3,5,0,0,0,0,0,2,6,1,3,11,1,7,4,4,8,10,7,5,5,5,2,9,1,4,3,4,0,0,0,0,1,3,4,3,5,9,2

	
```

```
		  

		NW_003726438_1, Position,0,1000,2000,3000,4000,5000,6000,7000,8000,9000,10000,11000,12000,13000,14000,15000,16000,17000,18000,19000,20000,21000,22000,23000,24000,25000,26000,27000,28000,29000,30000,31000,32000,33000,34000,35000,36000,37000,38000,39000,40000,41000,42000,43000,44000,45000
NW_003726438_1,Count,0,0,0,0,0,0,0,0,0,0,0,0,0,0,0,0,0,5,0,0,0,0,0,0,0,0,0,0,0,0,0,0,0,0,0,0,0,0,0,0,0,0,0,0,3,0

	
```

```
		  

		NW_003726439_1, Position,0,1000,2000,3000,4000,5000,6000,7000,8000,9000,10000,11000,12000,13000,14000,15000,16000,17000,18000,19000,20000,21000,22000,23000,24000,25000,26000,27000,28000,29000,30000,31000,32000,33000,34000,35000,36000,37000,38000,39000,40000
NW_003726439_1,Count,0,0,0,0,0,0,0,0,0,0,0,0,0,0,0,0,0,0,0,0,2,0,0,0,0,0,0,0,0,0,0,0,0,0,0,0,0,0,0,0,0

	
```

```
		  

		NW_003726442_1, Position,0,1000,2000,3000,4000,5000,6000,7000,8000,9000,10000,11000,12000,13000,14000,15000,16000,17000,18000,19000,20000,21000,22000,23000,24000,25000,26000,27000,28000,29000,30000,31000,32000,33000,34000,35000
NW_003726442_1,Count,5,7,6,13,8,6,7,2,9,9,10,6,7,10,12,8,11,10,9,7,1,5,5,8,7,7,5,0,0,0,8,2,0,10,10,10

	
```

```
		  

		NW_003726445_1, Position,0,1000,2000,3000,4000,5000,6000,7000,8000,9000,10000,11000,12000,13000,14000,15000,16000,17000,18000,19000,20000,21000,22000,23000,24000,25000,26000,27000,28000,29000,30000,31000,32000,33000,34000,35000,36000,37000,38000,39000,40000,41000,42000,43000,44000,45000,46000,47000,48000,49000,50000,51000,52000,53000,54000,55000,56000,57000,58000,59000,60000,61000,62000,63000,64000,65000,66000,67000
NW_003726445_1,Count,0,0,0,0,0,0,0,0,0,0,0,0,0,0,0,0,0,0,0,0,0,0,0,0,0,0,0,0,0,0,0,0,0,0,0,0,0,0,0,0,0,0,0,0,0,0,0,0,0,0,0,0,0,0,0,0,0,0,0,0,0,4,0,0,0,0,0,0

	
```

```
		  

		NW_003726455_1, Position,0,1000,2000,3000,4000,5000,6000,7000,8000,9000,10000,11000,12000,13000,14000,15000,16000,17000,18000,19000,20000,21000,22000,23000,24000,25000,26000,27000,28000,29000,30000,31000,32000,33000,34000
NW_003726455_1,Count,1,4,5,7,6,9,5,7,7,5,6,3,0,0,0,0,4,12,7,5,10,2,6,1,6,5,0,0,0,1,0,2,7,0,0

	
```

```
		  

		NW_003726457_1, Position,0,1000,2000,3000,4000,5000,6000,7000,8000,9000,10000,11000,12000,13000,14000,15000,16000,17000,18000,19000,20000,21000,22000,23000,24000,25000,26000,27000,28000,29000,30000,31000,32000,33000,34000,35000,36000,37000
NW_003726457_1,Count,0,0,0,5,2,3,0,0,5,0,0,0,0,0,0,0,0,0,0,0,0,0,0,0,0,0,0,1,0,0,0,2,0,0,0,1,0,0

	
```

```
		  

		NW_003726458_1, Position,0,1000,2000,3000,4000,5000,6000,7000,8000,9000,10000,11000,12000,13000,14000,15000,16000,17000,18000,19000,20000,21000,22000,23000,24000,25000,26000,27000,28000,29000,30000,31000,32000,33000,34000,35000,36000,37000,38000,39000,40000,41000,42000,43000,44000,45000,46000,47000,48000,49000,50000,51000,52000,53000,54000,55000,56000,57000,58000,59000
NW_003726458_1,Count,0,0,0,0,0,0,0,0,0,0,0,0,0,0,2,0,0,0,0,0,0,0,0,0,0,0,0,0,0,0,0,0,0,0,0,0,0,0,0,0,0,0,0,0,0,0,0,0,0,0,0,0,0,0,0,0,0,0,0,0

	
```

```
		  

		NW_003726460_1, Position,0,1000,2000,3000,4000,5000,6000,7000,8000,9000,10000,11000,12000,13000,14000,15000,16000,17000,18000,19000,20000,21000,22000,23000,24000,25000,26000,27000,28000,29000,30000,31000,32000,33000,34000,35000
NW_003726460_1,Count,0,0,0,0,0,0,0,1,6,0,0,0,0,3,0,0,0,3,9,3,0,0,0,0,0,0,0,0,0,0,0,0,0,0,0,0

	
```

```
		  

		NW_003726461_1, Position,0,1000,2000,3000,4000,5000,6000,7000,8000,9000,10000,11000,12000,13000,14000,15000,16000,17000,18000,19000,20000,21000,22000,23000,24000,25000,26000,27000,28000,29000,30000,31000,32000,33000,34000,35000,36000,37000,38000,39000,40000,41000
NW_003726461_1,Count,0,0,0,0,0,0,0,0,0,0,0,0,0,0,0,0,0,0,0,0,2,0,0,0,0,0,0,0,0,0,0,0,0,0,0,12,7,11,4,4,13,3

	
```

```
		  

		NW_003726462_1, Position,0,1000,2000,3000,4000,5000,6000,7000,8000,9000,10000,11000,12000,13000,14000,15000,16000,17000,18000,19000,20000,21000,22000,23000,24000,25000,26000,27000,28000,29000,30000,31000,32000,33000
NW_003726462_1,Count,5,9,2,5,6,1,0,0,0,0,0,10,8,0,0,2,3,3,0,0,2,1,0,0,0,0,0,0,0,0,1,0,0,0

	
```

```
		  

		NW_003726465_1, Position,0,1000,2000,3000,4000,5000,6000,7000,8000,9000,10000,11000,12000,13000,14000,15000,16000,17000,18000,19000,20000,21000,22000,23000,24000,25000,26000,27000,28000,29000,30000,31000,32000,33000
NW_003726465_1,Count,0,0,0,0,0,0,0,0,0,0,1,0,0,0,0,0,0,0,0,0,0,0,0,0,0,0,2,0,0,0,4,1,3,0

	
```

```
		  

		NW_003726466_1, Position,0,1000,2000,3000,4000,5000,6000,7000,8000,9000,10000,11000,12000,13000,14000,15000,16000,17000,18000,19000,20000,21000,22000,23000,24000,25000,26000,27000,28000,29000,30000,31000,32000,33000,34000
NW_003726466_1,Count,0,0,0,0,3,0,1,2,0,6,2,0,1,0,3,0,1,2,0,0,0,2,0,0,0,0,0,3,0,0,0,0,0,0,0

	
```

```
		  

		NW_003726477_1, Position,0,1000,2000,3000,4000,5000,6000,7000,8000,9000,10000,11000,12000,13000,14000,15000,16000,17000,18000,19000,20000,21000,22000,23000,24000,25000,26000,27000,28000,29000,30000,31000,32000,33000,34000,35000,36000,37000,38000,39000,40000,41000,42000
NW_003726477_1,Count,0,0,0,0,0,0,0,0,0,0,0,0,0,0,0,0,0,0,0,2,0,0,0,0,0,0,0,0,0,0,0,0,0,0,0,0,0,0,0,0,0,0,0

	
```

```
		  

		NW_003726478_1, Position,0,1000,2000,3000,4000,5000,6000,7000,8000,9000,10000,11000,12000,13000,14000,15000,16000,17000,18000,19000,20000,21000,22000,23000,24000,25000,26000,27000,28000,29000,30000,31000
NW_003726478_1,Count,0,1,0,3,0,1,0,4,0,0,0,0,3,1,0,0,3,3,0,0,3,0,2,3,2,0,0,0,0,0,1,0

	
```

```
		  

		NW_003726480_1, Position,0,1000,2000,3000,4000,5000,6000,7000,8000,9000,10000,11000,12000,13000,14000,15000,16000,17000,18000,19000,20000,21000,22000,23000,24000,25000,26000,27000,28000,29000,30000,31000,32000,33000,34000,35000,36000,37000,38000,39000,40000,41000,42000,43000,44000,45000,46000,47000,48000,49000,50000,51000
NW_003726480_1,Count,0,0,0,0,0,0,0,0,0,0,0,0,0,0,0,0,0,3,0,0,0,0,0,0,0,0,0,0,0,0,0,0,0,0,0,0,0,0,0,0,0,0,0,0,0,0,0,0,0,0,0,0

	
```

```
		  

		NW_003726481_1, Position,0,1000,2000,3000,4000,5000,6000,7000,8000,9000,10000,11000,12000,13000,14000,15000,16000,17000,18000,19000,20000,21000,22000,23000,24000,25000,26000,27000,28000,29000,30000,31000
NW_003726481_1,Count,10,10,10,5,9,10,9,10,9,10,13,10,10,8,5,5,17,15,11,11,13,6,2,3,4,6,7,8,7,13,8,2

	
```

```
		  

		NW_003726482_1, Position,0,1000,2000,3000,4000,5000,6000,7000,8000,9000,10000,11000,12000,13000,14000,15000,16000,17000,18000,19000,20000,21000,22000,23000,24000,25000,26000,27000,28000,29000,30000,31000,32000
NW_003726482_1,Count,0,3,1,0,0,0,0,0,0,0,0,0,0,0,3,0,0,0,0,0,0,0,0,0,0,0,0,0,0,0,0,0,0

	
```

```
		  

		NW_003726485_1, Position,0,1000,2000,3000,4000,5000,6000,7000,8000,9000,10000,11000,12000,13000,14000,15000,16000,17000,18000,19000,20000,21000,22000,23000,24000,25000,26000,27000,28000,29000,30000,31000,32000,33000,34000,35000,36000,37000,38000,39000,40000,41000,42000,43000,44000,45000,46000,47000,48000,49000,50000,51000,52000
NW_003726485_1,Count,0,0,0,0,0,0,0,0,0,0,0,0,0,0,0,0,0,0,0,0,0,0,0,0,0,0,0,0,0,0,0,0,0,0,0,0,0,0,0,0,0,0,3,0,0,0,0,0,0,0,0,0,0

	
```

```
		  

		NW_003726487_1, Position,0,1000,2000,3000,4000,5000,6000,7000,8000,9000,10000,11000,12000,13000,14000,15000,16000,17000,18000,19000,20000,21000,22000,23000,24000,25000,26000,27000,28000,29000,30000,31000
NW_003726487_1,Count,4,10,5,9,3,8,9,6,6,5,12,13,8,6,4,11,10,8,6,0,0,0,0,0,0,0,0,0,0,0,0,0

	
```

```
		  

		NW_003726488_1, Position,0,1000,2000,3000,4000,5000,6000,7000,8000,9000,10000,11000,12000,13000,14000,15000,16000,17000,18000,19000,20000,21000,22000,23000,24000,25000,26000,27000,28000,29000,30000
NW_003726488_1,Count,0,0,0,0,3,2,4,9,10,15,9,8,7,2,7,5,10,4,4,5,8,8,9,9,10,8,8,0,0,0,0

	
```

```
		  

		NW_003726489_1, Position,0,1000,2000,3000,4000,5000,6000,7000,8000,9000,10000,11000,12000,13000,14000,15000,16000,17000,18000,19000,20000,21000,22000,23000,24000,25000,26000,27000,28000,29000,30000,31000,32000,33000,34000,35000,36000,37000,38000,39000,40000,41000,42000,43000,44000,45000
NW_003726489_1,Count,0,0,0,0,0,0,0,0,0,0,0,0,0,0,0,0,0,0,0,0,0,0,0,0,0,0,0,0,0,0,0,0,0,0,0,0,4,0,0,0,0,0,0,0,0,0

	
```

```
		  

		NW_003726491_1, Position,0,1000,2000,3000,4000,5000,6000,7000,8000,9000,10000,11000,12000,13000,14000,15000,16000,17000,18000,19000,20000,21000,22000,23000,24000,25000,26000,27000,28000,29000,30000,31000,32000,33000,34000,35000,36000,37000,38000,39000
NW_003726491_1,Count,0,0,0,0,0,0,0,0,0,0,0,0,0,0,0,0,0,0,0,0,0,0,0,0,0,0,0,0,0,0,1,3,0,0,0,0,0,0,0,0

	
```

```
		  

		NW_003726492_1, Position,0,1000,2000,3000,4000,5000,6000,7000,8000,9000,10000,11000,12000,13000,14000,15000,16000,17000,18000,19000,20000,21000,22000,23000,24000,25000,26000,27000,28000,29000,30000,31000,32000,33000,34000,35000,36000,37000,38000,39000
NW_003726492_1,Count,0,0,0,0,0,0,0,0,0,0,0,0,0,0,0,0,0,0,0,0,0,0,0,2,0,0,0,0,0,0,0,0,0,0,0,0,0,0,0,0

	
```

```
		  

		NW_003726497_1, Position,0,1000,2000,3000,4000,5000,6000,7000,8000,9000,10000,11000,12000,13000,14000,15000,16000,17000,18000,19000,20000,21000,22000,23000,24000,25000,26000,27000,28000,29000,30000
NW_003726497_1,Count,0,0,0,0,0,0,0,0,0,0,0,0,1,0,0,0,0,0,0,0,0,0,0,0,0,0,0,0,0,0,0

	
```

```
		  

		NW_003726498_1, Position,0,1000,2000,3000,4000,5000,6000,7000,8000,9000,10000,11000,12000,13000,14000,15000,16000,17000,18000,19000,20000,21000,22000,23000,24000,25000,26000,27000,28000,29000,30000
NW_003726498_1,Count,0,6,11,5,5,6,0,4,0,0,0,0,0,0,0,0,0,0,0,0,0,0,0,0,0,0,0,0,0,2,0

	
```

```
		  

		NW_003726501_1, Position,0,1000,2000,3000,4000,5000,6000,7000,8000,9000,10000,11000,12000,13000,14000,15000,16000,17000,18000,19000,20000,21000,22000,23000,24000,25000,26000,27000,28000,29000,30000,31000
NW_003726501_1,Count,0,0,0,2,7,8,10,5,0,0,0,3,0,0,0,0,0,0,0,0,3,10,4,6,0,8,5,12,7,3,10,5

	
```

```
		  

		NW_003726505_1, Position,0,1000,2000,3000,4000,5000,6000,7000,8000,9000,10000,11000,12000,13000,14000,15000,16000,17000,18000,19000,20000,21000,22000,23000,24000,25000,26000,27000,28000,29000,30000
NW_003726505_1,Count,0,0,0,0,10,11,2,15,6,17,9,10,10,9,11,9,6,12,9,5,11,5,14,15,8,6,1,9,4,0,0

	
```

```
		  

		NW_003726511_1, Position,0,100,200,300,400,500,600,700,800,900,1000,1100,1200,1300,1400,1500,1600,1700,1800,1900,2000,2100,2200,2300,2400,2500,2600,2700,2800,2900,3000,3100,3200,3300,3400,3500,3600,3700,3800,3900,4000,4100,4200,4300,4400,4500,4600,4700,4800,4900,5000,5100,5200,5300,5400,5500,5600,5700,5800,5900,6000,6100,6200,6300,6400,6500,6600,6700,6800,6900,7000,7100,7200,7300,7400,7500,7600,7700,7800,7900,8000,8100,8200,8300,8400,8500,8600,8700,8800,8900,9000,9100,9200,9300,9400,9500,9600,9700,9800,9900,10000,10100,10200,10300,10400,10500,10600,10700,10800,10900,11000,11100,11200,11300,11400,11500,11600,11700,11800,11900,12000,12100,12200,12300,12400,12500,12600,12700,12800,12900,13000,13100,13200,13300,13400,13500,13600,13700,13800,13900,14000,14100,14200,14300,14400,14500,14600,14700,14800,14900,15000,15100,15200,15300,15400,15500,15600,15700,15800,15900,16000,16100,16200,16300,16400,16500,16600,16700,16800,16900,17000,17100,17200,17300,17400,17500,17600,17700,17800,17900,18000,18100,18200,18300,18400,18500,18600,18700,18800,18900,19000,19100,19200,19300,19400,19500,19600,19700,19800,19900,20000,20100,20200,20300,20400,20500,20600,20700,20800,20900,21000,21100,21200,21300,21400,21500,21600,21700,21800,21900,22000,22100,22200,22300,22400,22500,22600,22700,22800,22900,23000,23100,23200,23300,23400,23500,23600,23700,23800,23900,24000,24100,24200,24300,24400,24500,24600,24700,24800,24900,25000,25100,25200,25300,25400,25500,25600,25700,25800,25900,26000,26100,26200,26300,26400,26500,26600,26700,26800,26900,27000,27100,27200,27300,27400,27500,27600,27700,27800,27900,28000,28100,28200,28300,28400,28500,28600,28700,28800,28900,29000,29100,29200,29300,29400,29500,29600,29700,29800,29900
NW_003726511_1,Count,0,0,0,0,0,0,0,0,0,0,0,0,0,0,0,0,0,0,0,0,0,0,0,0,0,0,0,0,0,0,0,0,0,0,0,0,0,0,0,0,0,0,0,0,0,0,0,0,0,0,0,0,0,0,0,0,0,0,0,0,0,0,0,0,0,0,0,0,0,2,1,0,0,0,0,0,0,0,0,0,0,0,0,0,0,0,0,0,0,0,0,0,0,0,0,0,0,0,0,0,0,0,0,0,0,0,0,0,0,0,0,0,0,0,0,0,0,0,0,0,0,0,0,0,0,0,0,0,0,0,0,0,0,0,0,0,0,0,0,0,0,0,0,0,0,0,0,0,0,0,0,0,0,0,0,0,0,0,0,0,0,0,0,0,0,0,0,0,0,0,0,0,0,0,0,0,0,0,0,0,0,0,0,0,0,0,0,0,0,0,0,0,0,0,0,0,0,0,0,0,0,0,0,0,0,0,0,0,0,0,0,0,0,0,0,0,0,0,0,0,0,0,0,0,0,0,0,0,0,0,0,0,0,0,0,0,0,0,0,0,0,0,0,0,0,0,0,0,0,0,0,0,0,0,0,0,0,0,0,0,0,0,0,0,0,0,0,0,0,0,0,0,0,0,0,0,0,0,0,0,0,0,0,0,0,0,0,0,0,0,0,0,0,0,0,0,0,0,0,0

	
```

```
		  

		NW_003726512_1, Position,0,1000,2000,3000,4000,5000,6000,7000,8000,9000,10000,11000,12000,13000,14000,15000,16000,17000,18000,19000,20000,21000,22000,23000,24000,25000,26000,27000,28000,29000,30000,31000,32000,33000,34000,35000,36000,37000,38000
NW_003726512_1,Count,0,0,0,0,0,0,0,0,0,0,0,0,0,0,0,0,0,0,0,0,0,0,0,0,0,0,0,0,0,0,0,0,0,0,0,0,0,2,1

	
```

```
		  

		NW_003726513_1, Position,0,1000,2000,3000,4000,5000,6000,7000,8000,9000,10000,11000,12000,13000,14000,15000,16000,17000,18000,19000,20000,21000,22000,23000,24000,25000,26000,27000,28000,29000,30000,31000,32000,33000,34000,35000,36000,37000,38000,39000,40000,41000
NW_003726513_1,Count,0,0,0,0,0,0,0,0,0,0,0,0,0,0,0,0,0,0,0,0,0,0,0,0,0,0,0,0,0,0,0,0,0,0,0,0,0,0,0,0,1,0

	
```

```
		  

		NW_003726519_1, Position,0,100,200,300,400,500,600,700,800,900,1000,1100,1200,1300,1400,1500,1600,1700,1800,1900,2000,2100,2200,2300,2400,2500,2600,2700,2800,2900,3000,3100,3200,3300,3400,3500,3600,3700,3800,3900,4000,4100,4200,4300,4400,4500,4600,4700,4800,4900,5000,5100,5200,5300,5400,5500,5600,5700,5800,5900,6000,6100,6200,6300,6400,6500,6600,6700,6800,6900,7000,7100,7200,7300,7400,7500,7600,7700,7800,7900,8000,8100,8200,8300,8400,8500,8600,8700,8800,8900,9000,9100,9200,9300,9400,9500,9600,9700,9800,9900,10000,10100,10200,10300,10400,10500,10600,10700,10800,10900,11000,11100,11200,11300,11400,11500,11600,11700,11800,11900,12000,12100,12200,12300,12400,12500,12600,12700,12800,12900,13000,13100,13200,13300,13400,13500,13600,13700,13800,13900,14000,14100,14200,14300,14400,14500,14600,14700,14800,14900,15000,15100,15200,15300,15400,15500,15600,15700,15800,15900,16000,16100,16200,16300,16400,16500,16600,16700,16800,16900,17000,17100,17200,17300,17400,17500,17600,17700,17800,17900,18000,18100,18200,18300,18400,18500,18600,18700,18800,18900,19000,19100,19200,19300,19400,19500,19600,19700,19800,19900,20000,20100,20200,20300,20400,20500,20600,20700,20800,20900,21000,21100,21200,21300,21400,21500,21600,21700,21800,21900,22000,22100,22200,22300,22400,22500,22600,22700,22800,22900,23000,23100,23200,23300,23400,23500,23600,23700,23800,23900,24000,24100,24200,24300,24400,24500,24600,24700,24800,24900,25000,25100,25200,25300,25400,25500,25600,25700,25800,25900,26000,26100,26200,26300,26400,26500,26600,26700,26800,26900,27000,27100,27200,27300,27400,27500,27600,27700,27800,27900,28000,28100,28200,28300,28400,28500,28600,28700,28800,28900,29000,29100,29200,29300,29400,29500,29600,29700
NW_003726519_1,Count,0,0,0,1,0,0,0,0,0,0,0,0,0,0,0,0,0,0,0,0,0,0,0,0,0,0,0,0,1,1,2,1,2,0,1,3,2,0,0,0,0,0,0,0,0,0,0,0,0,0,0,0,0,0,1,1,0,0,0,0,0,0,0,0,0,0,0,0,0,0,0,0,0,0,0,0,0,0,0,0,0,0,0,1,1,0,0,0,0,0,0,0,0,0,0,0,0,0,0,0,0,0,0,0,0,0,0,0,0,0,0,0,0,0,0,0,0,0,0,0,0,0,0,0,0,0,0,0,0,0,0,1,0,0,0,0,0,0,0,0,0,0,0,0,0,0,0,0,0,0,0,0,0,0,0,0,0,0,0,0,0,0,0,0,0,0,0,0,0,0,0,0,0,0,0,0,0,0,0,0,0,0,0,0,0,0,0,1,2,0,0,0,0,0,0,0,0,0,0,0,0,0,0,0,0,0,0,0,0,0,0,0,0,0,0,0,0,0,0,0,0,1,0,0,1,0,0,0,0,0,0,0,0,0,0,0,0,0,0,0,0,0,0,0,0,0,0,0,0,0,0,0,0,0,0,0,0,0,0,0,0,0,0,0,0,0,0,0,0,0,0,0,0,0,0,0,0,0,0,0,0,0,0,0,0,0,0,0,0,0,0,0,0,0,0,0,0,0

	
```

```
		  

		NW_003726520_1, Position,0,100,200,300,400,500,600,700,800,900,1000,1100,1200,1300,1400,1500,1600,1700,1800,1900,2000,2100,2200,2300,2400,2500,2600,2700,2800,2900,3000,3100,3200,3300,3400,3500,3600,3700,3800,3900,4000,4100,4200,4300,4400,4500,4600,4700,4800,4900,5000,5100,5200,5300,5400,5500,5600,5700,5800,5900,6000,6100,6200,6300,6400,6500,6600,6700,6800,6900,7000,7100,7200,7300,7400,7500,7600,7700,7800,7900,8000,8100,8200,8300,8400,8500,8600,8700,8800,8900,9000,9100,9200,9300,9400,9500,9600,9700,9800,9900,10000,10100,10200,10300,10400,10500,10600,10700,10800,10900,11000,11100,11200,11300,11400,11500,11600,11700,11800,11900,12000,12100,12200,12300,12400,12500,12600,12700,12800,12900,13000,13100,13200,13300,13400,13500,13600,13700,13800,13900,14000,14100,14200,14300,14400,14500,14600,14700,14800,14900,15000,15100,15200,15300,15400,15500,15600,15700,15800,15900,16000,16100,16200,16300,16400,16500,16600,16700,16800,16900,17000,17100,17200,17300,17400,17500,17600,17700,17800,17900,18000,18100,18200,18300,18400,18500,18600,18700,18800,18900,19000,19100,19200,19300,19400,19500,19600,19700,19800,19900,20000,20100,20200,20300,20400,20500,20600,20700,20800,20900,21000,21100,21200,21300,21400,21500,21600,21700,21800,21900,22000,22100,22200,22300,22400,22500,22600,22700,22800,22900,23000,23100,23200,23300,23400,23500,23600,23700,23800,23900,24000,24100,24200,24300,24400,24500,24600,24700,24800,24900,25000,25100,25200,25300,25400,25500,25600,25700,25800,25900,26000,26100,26200,26300,26400,26500,26600,26700,26800,26900,27000,27100,27200,27300,27400,27500,27600,27700,27800,27900,28000,28100,28200,28300,28400,28500,28600,28700,28800,28900,29000,29100,29200,29300
NW_003726520_1,Count,2,1,1,1,1,0,0,0,0,0,0,0,0,0,0,0,0,0,0,0,0,0,0,0,0,0,0,0,0,0,0,0,0,0,0,0,0,0,0,1,1,2,0,1,0,0,0,0,0,0,0,0,0,0,2,0,2,0,0,1,1,0,0,0,1,1,3,0,2,1,1,1,0,2,0,0,1,0,0,0,2,0,0,0,0,0,0,0,0,0,0,0,0,0,0,0,0,0,0,0,0,0,2,2,0,1,0,1,0,3,1,1,1,0,0,0,0,0,0,0,0,0,0,0,0,0,0,0,0,0,4,0,0,0,0,0,0,0,1,0,0,0,0,0,0,0,0,0,1,1,1,1,2,2,0,0,0,0,0,0,0,0,1,2,2,0,1,1,1,1,1,0,0,0,0,0,0,0,0,0,0,0,0,0,0,0,0,0,0,0,0,0,1,0,2,0,1,0,0,0,0,1,0,0,0,1,0,0,0,0,0,0,2,2,0,0,0,1,1,0,0,0,0,0,0,0,1,1,2,0,0,0,0,2,0,1,0,0,1,1,3,1,1,2,0,2,2,0,2,1,0,2,1,2,3,1,0,0,0,0,1,0,1,2,1,1,1,2,1,0,0,0,0,2,1,1,2,0,0,0,0,0,2,0,3,0,1,4,1,3,1,0,0,0

	
```

```
		  

		NW_003726523_1, Position,0,100,200,300,400,500,600,700,800,900,1000,1100,1200,1300,1400,1500,1600,1700,1800,1900,2000,2100,2200,2300,2400,2500,2600,2700,2800,2900,3000,3100,3200,3300,3400,3500,3600,3700,3800,3900,4000,4100,4200,4300,4400,4500,4600,4700,4800,4900,5000,5100,5200,5300,5400,5500,5600,5700,5800,5900,6000,6100,6200,6300,6400,6500,6600,6700,6800,6900,7000,7100,7200,7300,7400,7500,7600,7700,7800,7900,8000,8100,8200,8300,8400,8500,8600,8700,8800,8900,9000,9100,9200,9300,9400,9500,9600,9700,9800,9900,10000,10100,10200,10300,10400,10500,10600,10700,10800,10900,11000,11100,11200,11300,11400,11500,11600,11700,11800,11900,12000,12100,12200,12300,12400,12500,12600,12700,12800,12900,13000,13100,13200,13300,13400,13500,13600,13700,13800,13900,14000,14100,14200,14300,14400,14500,14600,14700,14800,14900,15000,15100,15200,15300,15400,15500,15600,15700,15800,15900,16000,16100,16200,16300,16400,16500,16600,16700,16800,16900,17000,17100,17200,17300,17400,17500,17600,17700,17800,17900,18000,18100,18200,18300,18400,18500,18600,18700,18800,18900,19000,19100,19200,19300,19400,19500,19600,19700,19800,19900,20000,20100,20200,20300,20400,20500,20600,20700,20800,20900,21000,21100,21200,21300,21400,21500,21600,21700,21800,21900,22000,22100,22200,22300,22400,22500,22600,22700,22800,22900,23000,23100,23200,23300,23400,23500,23600,23700,23800,23900,24000,24100,24200,24300,24400,24500,24600,24700,24800,24900,25000,25100,25200,25300,25400,25500,25600,25700,25800,25900,26000,26100,26200,26300,26400,26500,26600,26700,26800,26900,27000,27100,27200,27300,27400,27500,27600,27700,27800,27900,28000,28100,28200,28300,28400,28500,28600,28700,28800,28900,29000,29100,29200,29300,29400,29500,29600,29700
NW_003726523_1,Count,0,0,0,0,0,0,0,0,0,0,0,0,0,0,0,0,0,0,0,0,0,0,0,0,0,0,1,2,1,0,0,0,0,0,0,0,0,0,0,0,0,0,0,0,0,0,0,0,0,0,0,1,2,0,0,0,0,0,0,1,0,2,0,0,0,0,0,0,0,0,0,0,0,0,0,0,0,0,0,0,0,0,0,0,0,1,0,0,0,0,0,0,0,0,0,1,0,0,0,0,1,0,1,0,0,2,1,1,2,1,3,0,0,0,0,0,0,0,0,0,0,0,0,0,0,0,0,1,0,1,0,0,0,0,0,0,0,0,0,0,0,0,0,0,0,0,0,0,0,0,0,0,1,0,0,1,1,0,1,0,0,0,0,0,0,0,0,0,0,0,0,0,0,0,0,0,0,0,0,0,0,0,0,0,0,0,0,0,0,0,0,0,0,0,0,0,0,0,0,0,1,1,3,2,0,0,0,0,3,1,0,0,0,0,0,0,0,0,0,0,0,0,0,0,0,0,2,1,0,0,0,0,0,0,0,0,0,0,0,2,0,0,0,0,0,0,0,0,0,0,0,2,3,0,0,0,0,0,0,0,0,0,0,0,0,0,0,0,0,0,0,0,0,0,1,1,0,0,0,0,0,0,0,0,0,0,0,0,0,0,0,0,0,0,0,0,0,0

	
```

```
		  

		NW_003726526_1, Position,0,1000,2000,3000,4000,5000,6000,7000,8000,9000,10000,11000,12000,13000,14000,15000,16000,17000,18000,19000,20000,21000,22000,23000,24000,25000,26000,27000,28000,29000,30000
NW_003726526_1,Count,9,7,9,10,14,11,11,7,10,5,6,11,10,8,6,14,9,7,9,7,6,7,8,5,10,9,7,6,6,4,0

	
```

```
		  

		NW_003726528_1, Position,0,100,200,300,400,500,600,700,800,900,1000,1100,1200,1300,1400,1500,1600,1700,1800,1900,2000,2100,2200,2300,2400,2500,2600,2700,2800,2900,3000,3100,3200,3300,3400,3500,3600,3700,3800,3900,4000,4100,4200,4300,4400,4500,4600,4700,4800,4900,5000,5100,5200,5300,5400,5500,5600,5700,5800,5900,6000,6100,6200,6300,6400,6500,6600,6700,6800,6900,7000,7100,7200,7300,7400,7500,7600,7700,7800,7900,8000,8100,8200,8300,8400,8500,8600,8700,8800,8900,9000,9100,9200,9300,9400,9500,9600,9700,9800,9900,10000,10100,10200,10300,10400,10500,10600,10700,10800,10900,11000,11100,11200,11300,11400,11500,11600,11700,11800,11900,12000,12100,12200,12300,12400,12500,12600,12700,12800,12900,13000,13100,13200,13300,13400,13500,13600,13700,13800,13900,14000,14100,14200,14300,14400,14500,14600,14700,14800,14900,15000,15100,15200,15300,15400,15500,15600,15700,15800,15900,16000,16100,16200,16300,16400,16500,16600,16700,16800,16900,17000,17100,17200,17300,17400,17500,17600,17700,17800,17900,18000,18100,18200,18300,18400,18500,18600,18700,18800,18900,19000,19100,19200,19300,19400,19500,19600,19700,19800,19900,20000,20100,20200,20300,20400,20500,20600,20700,20800,20900,21000,21100,21200,21300,21400,21500,21600,21700,21800,21900,22000,22100,22200,22300,22400,22500,22600,22700,22800,22900,23000,23100,23200,23300,23400,23500,23600,23700,23800,23900,24000,24100,24200,24300,24400,24500,24600,24700,24800,24900,25000,25100,25200,25300,25400,25500,25600,25700,25800,25900,26000,26100,26200,26300,26400,26500,26600,26700,26800,26900,27000,27100,27200,27300,27400,27500,27600,27700,27800,27900,28000,28100,28200,28300,28400,28500,28600,28700,28800,28900,29000,29100,29200,29300
NW_003726528_1,Count,0,0,0,0,0,0,0,0,0,0,0,0,0,0,0,0,0,0,0,0,0,0,0,0,0,0,0,0,0,0,0,0,0,0,0,0,0,0,0,0,0,0,0,0,0,0,0,0,0,0,0,0,0,0,0,0,0,0,0,0,0,0,0,0,0,0,0,0,0,0,0,0,0,0,0,0,0,0,0,0,0,0,0,0,0,0,0,0,0,0,0,0,0,0,0,0,0,0,0,0,0,0,0,0,0,0,0,0,0,0,0,0,0,0,0,0,0,0,0,0,0,0,0,0,0,0,0,0,0,0,0,0,0,0,0,0,0,0,0,0,0,0,0,0,0,0,0,0,0,0,0,0,0,0,0,0,0,0,0,0,0,0,0,0,0,0,0,0,0,0,0,0,0,0,0,0,0,0,0,0,0,0,0,0,0,0,0,0,0,0,0,0,0,0,0,0,0,0,0,0,0,0,0,0,0,0,0,0,0,0,0,0,0,0,0,0,0,0,0,0,0,0,0,0,0,0,0,0,0,0,0,0,0,0,0,0,0,0,0,0,0,0,0,0,0,0,0,0,0,0,0,0,0,0,0,0,0,0,0,0,0,0,0,0,0,0,0,0,0,0,0,0,0,0,0,0,0,0,0,0,0,0,0,0,0,0,0,0,1,1,0,0,0,0

	
```

```
		  

		NW_003726529_1, Position,0,100,200,300,400,500,600,700,800,900,1000,1100,1200,1300,1400,1500,1600,1700,1800,1900,2000,2100,2200,2300,2400,2500,2600,2700,2800,2900,3000,3100,3200,3300,3400,3500,3600,3700,3800,3900,4000,4100,4200,4300,4400,4500,4600,4700,4800,4900,5000,5100,5200,5300,5400,5500,5600,5700,5800,5900,6000,6100,6200,6300,6400,6500,6600,6700,6800,6900,7000,7100,7200,7300,7400,7500,7600,7700,7800,7900,8000,8100,8200,8300,8400,8500,8600,8700,8800,8900,9000,9100,9200,9300,9400,9500,9600,9700,9800,9900,10000,10100,10200,10300,10400,10500,10600,10700,10800,10900,11000,11100,11200,11300,11400,11500,11600,11700,11800,11900,12000,12100,12200,12300,12400,12500,12600,12700,12800,12900,13000,13100,13200,13300,13400,13500,13600,13700,13800,13900,14000,14100,14200,14300,14400,14500,14600,14700,14800,14900,15000,15100,15200,15300,15400,15500,15600,15700,15800,15900,16000,16100,16200,16300,16400,16500,16600,16700,16800,16900,17000,17100,17200,17300,17400,17500,17600,17700,17800,17900,18000,18100,18200,18300,18400,18500,18600,18700,18800,18900,19000,19100,19200,19300,19400,19500,19600,19700,19800,19900,20000,20100,20200,20300,20400,20500,20600,20700,20800,20900,21000,21100,21200,21300,21400,21500,21600,21700,21800,21900,22000,22100,22200,22300,22400,22500,22600,22700,22800,22900,23000,23100,23200,23300,23400,23500,23600,23700,23800,23900,24000,24100,24200,24300,24400,24500,24600,24700,24800,24900,25000,25100,25200,25300,25400,25500,25600,25700,25800,25900,26000,26100,26200,26300,26400,26500,26600,26700,26800,26900,27000,27100,27200,27300,27400,27500,27600,27700,27800,27900,28000,28100,28200,28300,28400,28500,28600,28700,28800,28900
NW_003726529_1,Count,0,0,0,0,0,0,0,0,0,0,0,0,1,4,3,0,0,0,0,0,0,0,0,1,2,1,0,0,0,0,0,0,0,0,0,0,0,0,0,0,0,0,0,0,0,0,0,0,0,0,0,0,0,0,1,0,2,2,2,0,0,0,0,0,0,0,0,0,0,0,0,0,0,0,0,0,1,3,0,0,0,0,0,0,0,1,0,1,2,0,0,0,0,0,0,0,0,0,0,0,0,0,0,0,0,0,0,0,0,0,0,0,0,0,0,0,0,0,0,0,0,0,2,0,1,0,0,0,0,0,0,0,0,0,0,0,0,0,0,0,0,0,0,0,0,0,0,0,0,0,0,0,0,0,0,0,0,0,0,0,0,0,0,0,0,0,0,0,0,0,0,0,3,0,0,0,0,0,0,0,0,0,0,0,0,0,1,1,0,0,0,0,0,0,0,0,0,0,0,0,0,0,0,0,0,0,0,0,1,0,0,0,0,0,0,0,0,0,0,0,0,0,0,0,0,0,0,0,0,0,0,0,0,0,0,0,0,0,0,0,0,0,0,0,0,0,0,0,0,0,0,0,0,0,0,0,0,0,0,0,0,0,0,0,0,0,0,0,0,0,0,0,0,0,0,0,0,0,0,0,0,0,0,0,0,0,0,0,0,0

	
```

```
		  

		NW_003726530_1, Position,0,1000,2000,3000,4000,5000,6000,7000,8000,9000,10000,11000,12000,13000,14000,15000,16000,17000,18000,19000,20000,21000,22000,23000,24000,25000,26000,27000,28000,29000,30000,31000,32000
NW_003726530_1,Count,10,11,6,5,1,3,2,14,12,15,8,1,10,19,10,5,6,7,4,7,3,7,8,14,6,2,6,1,0,1,13,5,9

	
```

```
		  

		NW_003726532_1, Position,0,100,200,300,400,500,600,700,800,900,1000,1100,1200,1300,1400,1500,1600,1700,1800,1900,2000,2100,2200,2300,2400,2500,2600,2700,2800,2900,3000,3100,3200,3300,3400,3500,3600,3700,3800,3900,4000,4100,4200,4300,4400,4500,4600,4700,4800,4900,5000,5100,5200,5300,5400,5500,5600,5700,5800,5900,6000,6100,6200,6300,6400,6500,6600,6700,6800,6900,7000,7100,7200,7300,7400,7500,7600,7700,7800,7900,8000,8100,8200,8300,8400,8500,8600,8700,8800,8900,9000,9100,9200,9300,9400,9500,9600,9700,9800,9900,10000,10100,10200,10300,10400,10500,10600,10700,10800,10900,11000,11100,11200,11300,11400,11500,11600,11700,11800,11900,12000,12100,12200,12300,12400,12500,12600,12700,12800,12900,13000,13100,13200,13300,13400,13500,13600,13700,13800,13900,14000,14100,14200,14300,14400,14500,14600,14700,14800,14900,15000,15100,15200,15300,15400,15500,15600,15700,15800,15900,16000,16100,16200,16300,16400,16500,16600,16700,16800,16900,17000,17100,17200,17300,17400,17500,17600,17700,17800,17900,18000,18100,18200,18300,18400,18500,18600,18700,18800,18900,19000,19100,19200,19300,19400,19500,19600,19700,19800,19900,20000,20100,20200,20300,20400,20500,20600,20700,20800,20900,21000,21100,21200,21300,21400,21500,21600,21700,21800,21900,22000,22100,22200,22300,22400,22500,22600,22700,22800,22900,23000,23100,23200,23300,23400,23500,23600,23700,23800,23900,24000,24100,24200,24300,24400,24500,24600,24700,24800,24900,25000,25100,25200,25300,25400,25500,25600,25700,25800,25900,26000,26100,26200,26300,26400,26500,26600,26700,26800,26900,27000,27100,27200,27300,27400,27500,27600,27700,27800,27900,28000,28100,28200,28300,28400,28500,28600,28700,28800,28900
NW_003726532_1,Count,0,0,0,0,0,0,0,0,0,0,0,0,0,0,0,0,0,0,0,0,0,0,0,0,0,0,0,0,0,0,0,0,0,0,0,0,0,0,0,0,0,0,0,0,0,0,0,0,0,0,0,0,0,0,0,0,0,0,0,0,2,0,2,1,1,0,0,0,0,0,0,0,0,0,0,0,0,0,0,0,0,0,0,0,0,0,0,0,0,0,0,0,0,0,0,0,0,0,0,0,0,0,0,0,0,0,0,0,0,0,1,0,1,0,1,0,1,1,1,0,2,0,2,1,0,0,1,0,1,0,0,0,0,2,0,0,0,1,0,0,0,2,0,0,2,0,1,0,2,1,1,0,1,0,0,0,0,1,1,0,1,0,0,1,1,2,0,0,0,1,0,0,2,0,0,0,1,1,0,1,0,0,0,0,0,0,0,0,1,0,1,1,0,1,0,0,0,1,2,2,1,0,0,1,3,0,4,0,0,0,0,0,0,0,0,0,0,0,0,0,0,0,0,0,0,0,0,0,0,0,0,0,0,0,0,0,0,0,0,0,0,0,0,0,0,0,0,0,0,0,0,0,0,0,0,0,0,0,0,0,0,0,0,0,0,0,0,3,0,2,0,0,0,0,0,0,0,0,0,0,0,0,0,0,0,0,2,2,1,0

	
```

```
		  

		NW_003726534_1, Position,0,100,200,300,400,500,600,700,800,900,1000,1100,1200,1300,1400,1500,1600,1700,1800,1900,2000,2100,2200,2300,2400,2500,2600,2700,2800,2900,3000,3100,3200,3300,3400,3500,3600,3700,3800,3900,4000,4100,4200,4300,4400,4500,4600,4700,4800,4900,5000,5100,5200,5300,5400,5500,5600,5700,5800,5900,6000,6100,6200,6300,6400,6500,6600,6700,6800,6900,7000,7100,7200,7300,7400,7500,7600,7700,7800,7900,8000,8100,8200,8300,8400,8500,8600,8700,8800,8900,9000,9100,9200,9300,9400,9500,9600,9700,9800,9900,10000,10100,10200,10300,10400,10500,10600,10700,10800,10900,11000,11100,11200,11300,11400,11500,11600,11700,11800,11900,12000,12100,12200,12300,12400,12500,12600,12700,12800,12900,13000,13100,13200,13300,13400,13500,13600,13700,13800,13900,14000,14100,14200,14300,14400,14500,14600,14700,14800,14900,15000,15100,15200,15300,15400,15500,15600,15700,15800,15900,16000,16100,16200,16300,16400,16500,16600,16700,16800,16900,17000,17100,17200,17300,17400,17500,17600,17700,17800,17900,18000,18100,18200,18300,18400,18500,18600,18700,18800,18900,19000,19100,19200,19300,19400,19500,19600,19700,19800,19900,20000,20100,20200,20300,20400,20500,20600,20700,20800,20900,21000,21100,21200,21300,21400,21500,21600,21700,21800,21900,22000,22100,22200,22300,22400,22500,22600,22700,22800,22900,23000,23100,23200,23300,23400,23500,23600,23700,23800,23900,24000,24100,24200,24300,24400,24500,24600,24700,24800,24900,25000,25100,25200,25300,25400,25500,25600,25700,25800,25900,26000,26100,26200,26300,26400,26500,26600,26700,26800,26900,27000,27100,27200,27300,27400,27500,27600,27700,27800,27900,28000,28100,28200,28300,28400,28500,28600,28700
NW_003726534_1,Count,0,2,1,0,1,0,2,2,1,2,0,0,0,2,3,0,1,1,2,0,3,1,0,0,0,0,0,0,1,1,0,3,0,0,0,0,0,1,0,0,1,2,0,2,2,0,0,0,0,0,0,0,0,0,0,0,0,0,0,0,2,2,2,1,1,0,0,0,0,0,0,0,0,0,0,0,1,0,0,0,0,0,0,0,0,0,0,0,0,0,0,0,0,0,0,0,0,0,0,0,0,0,0,0,0,0,3,2,2,0,0,0,0,0,0,0,0,0,0,0,1,1,0,0,0,0,0,2,2,0,0,0,0,0,0,0,0,0,0,0,0,0,0,0,0,0,0,0,0,0,0,0,0,0,0,0,1,0,1,0,0,0,0,0,0,0,0,0,0,0,0,1,1,1,0,0,0,0,0,0,0,0,0,0,0,0,0,0,0,0,0,0,0,0,0,0,0,0,0,0,0,0,0,0,0,0,0,0,0,0,0,0,0,0,0,0,0,0,0,0,0,0,0,0,0,0,0,1,0,1,3,1,3,2,2,1,0,0,0,0,0,0,0,0,3,2,0,0,0,0,0,0,0,0,0,0,0,0,0,0,0,0,0,0,0,0,0,0,0,0,0,0,0,0,0,0,0,0,0,0,0,0,0,0,0,0,0,0

	
```

```
		  

		NW_003726540_1, Position,0,100,200,300,400,500,600,700,800,900,1000,1100,1200,1300,1400,1500,1600,1700,1800,1900,2000,2100,2200,2300,2400,2500,2600,2700,2800,2900,3000,3100,3200,3300,3400,3500,3600,3700,3800,3900,4000,4100,4200,4300,4400,4500,4600,4700,4800,4900,5000,5100,5200,5300,5400,5500,5600,5700,5800,5900,6000,6100,6200,6300,6400,6500,6600,6700,6800,6900,7000,7100,7200,7300,7400,7500,7600,7700,7800,7900,8000,8100,8200,8300,8400,8500,8600,8700,8800,8900,9000,9100,9200,9300,9400,9500,9600,9700,9800,9900,10000,10100,10200,10300,10400,10500,10600,10700,10800,10900,11000,11100,11200,11300,11400,11500,11600,11700,11800,11900,12000,12100,12200,12300,12400,12500,12600,12700,12800,12900,13000,13100,13200,13300,13400,13500,13600,13700,13800,13900,14000,14100,14200,14300,14400,14500,14600,14700,14800,14900,15000,15100,15200,15300,15400,15500,15600,15700,15800,15900,16000,16100,16200,16300,16400,16500,16600,16700,16800,16900,17000,17100,17200,17300,17400,17500,17600,17700,17800,17900,18000,18100,18200,18300,18400,18500,18600,18700,18800,18900,19000,19100,19200,19300,19400,19500,19600,19700,19800,19900,20000,20100,20200,20300,20400,20500,20600,20700,20800,20900,21000,21100,21200,21300,21400,21500,21600,21700,21800,21900,22000,22100,22200,22300,22400,22500,22600,22700,22800,22900,23000,23100,23200,23300,23400,23500,23600,23700,23800,23900,24000,24100,24200,24300,24400,24500,24600,24700,24800,24900,25000,25100,25200,25300,25400,25500,25600,25700,25800,25900,26000,26100,26200,26300,26400,26500,26600,26700,26800,26900,27000,27100,27200,27300,27400,27500,27600,27700,27800,27900,28000,28100,28200,28300,28400,28500,28600,28700,28800,28900,29000,29100
NW_003726540_1,Count,0,0,0,0,0,0,0,0,0,0,0,0,0,0,0,0,0,0,0,0,0,0,0,0,0,0,0,0,0,0,0,0,0,0,0,0,0,0,0,0,0,0,0,0,0,0,0,0,0,0,0,0,0,0,0,0,0,0,0,0,0,0,0,0,0,0,0,0,0,0,0,0,0,0,0,0,0,0,0,0,0,0,0,0,0,0,0,0,0,0,0,0,0,0,0,0,0,0,0,0,0,0,0,0,0,0,0,0,0,0,0,0,0,0,0,0,0,0,0,0,0,0,0,0,0,0,0,0,0,0,0,0,0,0,0,0,0,0,0,0,0,0,0,0,0,0,0,0,0,0,0,0,0,0,0,0,0,0,0,0,0,0,0,0,0,0,0,0,0,0,0,0,0,0,0,0,0,0,0,0,0,0,0,0,0,0,0,0,0,0,0,0,0,0,0,0,0,0,0,0,0,0,0,0,0,0,0,0,0,0,0,0,0,0,0,0,0,0,0,0,0,0,0,0,0,0,0,0,0,0,0,0,0,1,1,0,0,0,0,0,0,0,0,0,0,0,0,0,0,0,0,0,0,0,0,0,0,0,0,0,0,0,0,0,0,0,0,0,0,0,0,0,0,0,0,0,0,0,0,0,0,0,0,0,0,0,0,0,0,0,0,0

	
```

```
		  

		NW_003726545_1, Position,0,1000,2000,3000,4000,5000,6000,7000,8000,9000,10000,11000,12000,13000,14000,15000,16000,17000,18000,19000,20000,21000,22000,23000,24000,25000,26000,27000,28000,29000,30000,31000,32000,33000,34000,35000,36000,37000,38000,39000,40000,41000,42000,43000,44000,45000,46000,47000,48000,49000,50000,51000
NW_003726545_1,Count,8,5,0,0,0,0,0,0,0,8,8,0,0,0,0,0,0,0,0,0,0,0,0,0,0,0,0,0,0,0,0,0,0,0,0,7,0,0,0,0,0,0,0,0,0,0,0,0,0,0,0,0

	
```

```
		  

		NW_003726549_1, Position,0,100,200,300,400,500,600,700,800,900,1000,1100,1200,1300,1400,1500,1600,1700,1800,1900,2000,2100,2200,2300,2400,2500,2600,2700,2800,2900,3000,3100,3200,3300,3400,3500,3600,3700,3800,3900,4000,4100,4200,4300,4400,4500,4600,4700,4800,4900,5000,5100,5200,5300,5400,5500,5600,5700,5800,5900,6000,6100,6200,6300,6400,6500,6600,6700,6800,6900,7000,7100,7200,7300,7400,7500,7600,7700,7800,7900,8000,8100,8200,8300,8400,8500,8600,8700,8800,8900,9000,9100,9200,9300,9400,9500,9600,9700,9800,9900,10000,10100,10200,10300,10400,10500,10600,10700,10800,10900,11000,11100,11200,11300,11400,11500,11600,11700,11800,11900,12000,12100,12200,12300,12400,12500,12600,12700,12800,12900,13000,13100,13200,13300,13400,13500,13600,13700,13800,13900,14000,14100,14200,14300,14400,14500,14600,14700,14800,14900,15000,15100,15200,15300,15400,15500,15600,15700,15800,15900,16000,16100,16200,16300,16400,16500,16600,16700,16800,16900,17000,17100,17200,17300,17400,17500,17600,17700,17800,17900,18000,18100,18200,18300,18400,18500,18600,18700,18800,18900,19000,19100,19200,19300,19400,19500,19600,19700,19800,19900,20000,20100,20200,20300,20400,20500,20600,20700,20800,20900,21000,21100,21200,21300,21400,21500,21600,21700,21800,21900,22000,22100,22200,22300,22400,22500,22600,22700,22800,22900,23000,23100,23200,23300,23400,23500,23600,23700,23800,23900,24000,24100,24200,24300,24400,24500,24600,24700,24800,24900,25000,25100,25200,25300,25400,25500,25600,25700,25800,25900,26000,26100,26200,26300,26400,26500,26600,26700,26800,26900,27000,27100,27200,27300,27400,27500,27600,27700,27800,27900,28000,28100,28200
NW_003726549_1,Count,0,0,1,2,0,1,0,0,2,1,3,1,1,1,0,0,0,1,0,0,0,0,2,1,1,1,0,0,0,1,0,0,0,0,1,0,0,0,0,0,2,0,1,0,1,0,0,0,0,1,0,1,3,0,0,0,0,1,1,0,1,0,2,0,0,0,0,0,1,2,1,0,1,1,1,1,0,0,1,2,0,0,0,3,2,1,0,1,0,0,1,0,1,0,1,0,0,1,0,1,0,0,1,0,1,0,1,0,0,0,0,0,2,1,0,0,1,0,2,1,0,1,1,0,1,1,1,1,1,0,1,1,0,1,0,1,0,0,1,2,1,0,0,1,1,1,2,0,1,1,1,0,1,1,1,1,1,0,1,1,0,1,1,2,0,0,1,1,2,0,0,0,1,1,1,1,0,0,0,0,0,4,0,1,0,0,0,0,3,1,0,0,0,0,0,1,0,0,0,0,0,0,2,1,0,2,1,2,1,1,2,0,2,1,1,1,1,0,0,0,0,0,1,0,1,1,1,2,1,0,0,2,0,0,0,2,0,3,0,1,0,0,0,0,0,1,0,2,0,0,0,0,0,0,0,1,1,0,1,0,2,0,0,1,1,0,0,0,1,0,1,0,0,1,0,0,0,0,0,0,0,0,0

	
```

```
		  

		NW_003726551_1, Position,0,1000,2000,3000,4000,5000,6000,7000,8000,9000,10000,11000,12000,13000,14000,15000,16000,17000,18000,19000,20000,21000,22000,23000,24000,25000,26000,27000,28000,29000,30000,31000,32000,33000,34000,35000,36000,37000,38000
NW_003726551_1,Count,0,0,0,0,0,0,0,0,0,0,0,0,0,0,0,0,0,0,0,0,0,0,0,0,0,0,0,0,0,2,0,0,0,0,0,0,0,0,0

	
```

```
		  

		NW_003726552_1, Position,0,1000,2000,3000,4000,5000,6000,7000,8000,9000,10000,11000,12000,13000,14000,15000,16000,17000,18000,19000,20000,21000,22000,23000,24000,25000,26000,27000,28000,29000,30000,31000,32000,33000,34000,35000,36000,37000,38000
NW_003726552_1,Count,0,0,0,0,0,0,0,0,0,0,0,0,0,0,0,0,0,0,0,0,0,0,0,0,0,0,0,0,0,0,0,0,0,0,0,0,4,0,0

	
```

```
		  

		NW_003726553_1, Position,0,1000,2000,3000,4000,5000,6000,7000,8000,9000,10000,11000,12000,13000,14000,15000,16000,17000,18000,19000,20000,21000,22000,23000,24000,25000,26000,27000,28000,29000,30000,31000,32000,33000,34000,35000,36000,37000
NW_003726553_1,Count,0,0,0,0,0,0,0,0,0,0,0,1,0,0,2,0,0,0,0,0,0,0,0,0,0,0,0,0,0,0,0,0,0,0,0,0,0,0

	
```

```
		  

		NW_003726555_1, Position,0,100,200,300,400,500,600,700,800,900,1000,1100,1200,1300,1400,1500,1600,1700,1800,1900,2000,2100,2200,2300,2400,2500,2600,2700,2800,2900,3000,3100,3200,3300,3400,3500,3600,3700,3800,3900,4000,4100,4200,4300,4400,4500,4600,4700,4800,4900,5000,5100,5200,5300,5400,5500,5600,5700,5800,5900,6000,6100,6200,6300,6400,6500,6600,6700,6800,6900,7000,7100,7200,7300,7400,7500,7600,7700,7800,7900,8000,8100,8200,8300,8400,8500,8600,8700,8800,8900,9000,9100,9200,9300,9400,9500,9600,9700,9800,9900,10000,10100,10200,10300,10400,10500,10600,10700,10800,10900,11000,11100,11200,11300,11400,11500,11600,11700,11800,11900,12000,12100,12200,12300,12400,12500,12600,12700,12800,12900,13000,13100,13200,13300,13400,13500,13600,13700,13800,13900,14000,14100,14200,14300,14400,14500,14600,14700,14800,14900,15000,15100,15200,15300,15400,15500,15600,15700,15800,15900,16000,16100,16200,16300,16400,16500,16600,16700,16800,16900,17000,17100,17200,17300,17400,17500,17600,17700,17800,17900,18000,18100,18200,18300,18400,18500,18600,18700,18800,18900,19000,19100,19200,19300,19400,19500,19600,19700,19800,19900,20000,20100,20200,20300,20400,20500,20600,20700,20800,20900,21000,21100,21200,21300,21400,21500,21600,21700,21800,21900,22000,22100,22200,22300,22400,22500,22600,22700,22800,22900,23000,23100,23200,23300,23400,23500,23600,23700,23800,23900,24000,24100,24200,24300,24400,24500,24600,24700,24800,24900,25000,25100,25200,25300,25400,25500,25600,25700,25800,25900,26000,26100,26200,26300,26400,26500,26600,26700,26800,26900,27000,27100,27200,27300,27400,27500,27600,27700,27800,27900
NW_003726555_1,Count,0,0,0,0,0,0,0,0,0,0,0,2,1,0,1,0,0,1,0,0,2,1,2,1,0,2,0,0,1,1,0,1,2,0,1,2,1,0,0,0,0,0,1,3,0,1,0,1,1,1,0,1,2,0,0,0,0,0,0,0,0,0,0,0,0,0,0,0,0,0,0,0,0,0,0,0,0,0,0,0,0,0,0,0,0,0,0,0,0,0,0,0,0,0,0,0,0,0,0,0,0,0,0,0,0,0,0,2,2,2,0,1,0,0,0,3,1,0,0,0,0,1,1,2,1,1,0,0,0,0,1,1,1,2,0,1,1,2,0,1,0,0,0,0,1,1,0,0,2,0,0,0,0,0,0,0,0,0,0,2,0,0,0,0,0,0,0,0,0,0,0,0,0,0,0,0,0,0,0,0,0,0,0,0,0,0,0,0,0,0,0,0,0,0,0,0,0,0,0,0,0,0,0,0,0,0,0,0,0,0,0,1,0,0,0,0,0,1,0,2,3,0,1,1,0,0,0,0,0,0,0,0,0,0,0,0,0,0,0,0,0,0,0,0,0,0,0,0,0,0,0,0,0,0,0,0,0,0,0,0,0,0,0,0,0,0,0,0,0,0,0,0,0,0,0,0,0,0,0,0

	
```

```
		  

		NW_003726556_1, Position,0,1000,2000,3000,4000,5000,6000,7000,8000,9000,10000,11000,12000,13000,14000,15000,16000,17000,18000,19000,20000,21000,22000,23000,24000,25000,26000,27000,28000,29000,30000,31000,32000,33000,34000,35000,36000,37000,38000
NW_003726556_1,Count,0,0,0,0,0,0,0,0,0,0,0,0,0,0,0,0,0,0,0,0,0,0,2,1,0,0,0,0,0,0,0,0,0,0,0,0,0,0,0

	
```

```
		  

		NW_003726562_1, Position,0,100,200,300,400,500,600,700,800,900,1000,1100,1200,1300,1400,1500,1600,1700,1800,1900,2000,2100,2200,2300,2400,2500,2600,2700,2800,2900,3000,3100,3200,3300,3400,3500,3600,3700,3800,3900,4000,4100,4200,4300,4400,4500,4600,4700,4800,4900,5000,5100,5200,5300,5400,5500,5600,5700,5800,5900,6000,6100,6200,6300,6400,6500,6600,6700,6800,6900,7000,7100,7200,7300,7400,7500,7600,7700,7800,7900,8000,8100,8200,8300,8400,8500,8600,8700,8800,8900,9000,9100,9200,9300,9400,9500,9600,9700,9800,9900,10000,10100,10200,10300,10400,10500,10600,10700,10800,10900,11000,11100,11200,11300,11400,11500,11600,11700,11800,11900,12000,12100,12200,12300,12400,12500,12600,12700,12800,12900,13000,13100,13200,13300,13400,13500,13600,13700,13800,13900,14000,14100,14200,14300,14400,14500,14600,14700,14800,14900,15000,15100,15200,15300,15400,15500,15600,15700,15800,15900,16000,16100,16200,16300,16400,16500,16600,16700,16800,16900,17000,17100,17200,17300,17400,17500,17600,17700,17800,17900,18000,18100,18200,18300,18400,18500,18600,18700,18800,18900,19000,19100,19200,19300,19400,19500,19600,19700,19800,19900,20000,20100,20200,20300,20400,20500,20600,20700,20800,20900,21000,21100,21200,21300,21400,21500,21600,21700,21800,21900,22000,22100,22200,22300,22400,22500,22600,22700,22800,22900,23000,23100,23200,23300,23400,23500,23600,23700,23800,23900,24000,24100,24200,24300,24400,24500,24600,24700,24800,24900,25000,25100,25200,25300,25400,25500,25600,25700,25800,25900,26000,26100,26200,26300,26400,26500,26600,26700,26800,26900,27000,27100,27200,27300,27400,27500,27600,27700,27800
NW_003726562_1,Count,0,0,0,0,0,0,0,0,0,0,0,0,0,0,0,0,0,0,0,0,0,0,0,0,0,0,0,0,0,0,0,0,0,0,0,0,0,0,0,0,0,0,0,0,0,0,0,0,0,0,0,0,0,0,0,0,0,0,0,0,0,0,0,0,0,0,0,0,0,0,0,0,0,0,0,0,0,0,0,0,0,0,0,0,0,0,0,0,0,0,0,0,0,0,0,0,0,0,0,0,0,0,0,0,0,0,3,0,0,0,0,0,0,0,0,0,0,0,0,0,0,0,0,0,0,0,0,0,0,0,0,0,0,0,0,0,0,0,0,0,0,0,0,0,0,0,0,0,0,0,0,0,0,0,0,0,0,2,0,2,0,0,0,0,0,0,0,0,0,0,0,2,2,1,1,0,0,0,0,0,0,0,1,0,0,0,0,0,0,0,0,0,0,0,0,0,0,0,0,0,0,0,0,0,0,0,0,0,0,0,0,0,0,0,0,0,0,0,0,0,0,0,0,0,0,0,0,0,0,0,2,2,0,1,0,0,0,0,0,0,0,0,0,0,0,0,0,0,0,0,0,0,0,0,1,0,0,0,0,0,0,0,0,0,3,1,0,2,1,2,0,0,0,0,0,0,0,0,0

	
```

```
		  

		NW_003726563_1, Position,0,1000,2000,3000,4000,5000,6000,7000,8000,9000,10000,11000,12000,13000,14000,15000,16000,17000,18000,19000,20000,21000,22000,23000,24000,25000,26000,27000,28000,29000,30000,31000,32000,33000,34000,35000,36000,37000,38000,39000,40000,41000
NW_003726563_1,Count,0,0,0,0,0,0,0,0,0,0,1,0,0,1,2,0,3,0,0,0,1,2,1,1,0,0,0,0,0,0,0,0,0,0,3,2,2,2,0,0,0,0

	
```

```
		  

		NW_003726567_1, Position,0,100,200,300,400,500,600,700,800,900,1000,1100,1200,1300,1400,1500,1600,1700,1800,1900,2000,2100,2200,2300,2400,2500,2600,2700,2800,2900,3000,3100,3200,3300,3400,3500,3600,3700,3800,3900,4000,4100,4200,4300,4400,4500,4600,4700,4800,4900,5000,5100,5200,5300,5400,5500,5600,5700,5800,5900,6000,6100,6200,6300,6400,6500,6600,6700,6800,6900,7000,7100,7200,7300,7400,7500,7600,7700,7800,7900,8000,8100,8200,8300,8400,8500,8600,8700,8800,8900,9000,9100,9200,9300,9400,9500,9600,9700,9800,9900,10000,10100,10200,10300,10400,10500,10600,10700,10800,10900,11000,11100,11200,11300,11400,11500,11600,11700,11800,11900,12000,12100,12200,12300,12400,12500,12600,12700,12800,12900,13000,13100,13200,13300,13400,13500,13600,13700,13800,13900,14000,14100,14200,14300,14400,14500,14600,14700,14800,14900,15000,15100,15200,15300,15400,15500,15600,15700,15800,15900,16000,16100,16200,16300,16400,16500,16600,16700,16800,16900,17000,17100,17200,17300,17400,17500,17600,17700,17800,17900,18000,18100,18200,18300,18400,18500,18600,18700,18800,18900,19000,19100,19200,19300,19400,19500,19600,19700,19800,19900,20000,20100,20200,20300,20400,20500,20600,20700,20800,20900,21000,21100,21200,21300,21400,21500,21600,21700,21800,21900,22000,22100,22200,22300,22400,22500,22600,22700,22800,22900,23000,23100,23200,23300,23400,23500,23600,23700,23800,23900,24000,24100,24200,24300,24400,24500,24600,24700,24800,24900,25000,25100,25200,25300,25400,25500,25600,25700,25800,25900,26000,26100,26200,26300,26400,26500,26600,26700,26800,26900,27000,27100,27200,27300,27400
NW_003726567_1,Count,1,1,2,0,1,2,1,0,0,1,1,0,1,1,0,0,0,3,1,1,1,2,0,1,0,3,0,1,1,0,2,2,1,1,0,2,2,1,0,0,0,0,2,0,0,0,1,0,3,1,1,0,0,1,1,1,0,2,1,0,0,1,0,0,1,0,0,0,0,1,1,2,1,1,1,1,2,1,1,1,1,0,1,1,1,1,1,0,0,0,0,1,1,1,0,2,0,0,2,1,0,2,0,1,2,2,0,0,0,2,2,0,0,0,0,0,1,1,0,1,0,0,0,1,0,2,1,0,0,0,2,0,0,0,1,0,0,0,1,0,1,0,2,2,1,0,0,0,0,0,0,0,0,0,0,1,1,2,0,0,1,0,0,0,1,0,0,1,1,0,0,3,0,0,0,0,1,0,1,1,0,0,0,0,0,0,0,0,1,2,1,0,2,1,0,4,1,1,0,2,0,2,1,2,1,0,0,1,0,1,1,2,0,1,0,0,1,1,2,3,0,0,0,0,0,0,0,1,1,1,2,0,1,0,0,0,0,0,0,0,0,0,0,0,0,0,0,0,0,0,0,0,0,0,0,0,0,0,1,0,2,1,0,0,0,2,0,0,2,0,0,1,1,2,2

	
```

```
		  

		NW_003726568_1, Position,0,100,200,300,400,500,600,700,800,900,1000,1100,1200,1300,1400,1500,1600,1700,1800,1900,2000,2100,2200,2300,2400,2500,2600,2700,2800,2900,3000,3100,3200,3300,3400,3500,3600,3700,3800,3900,4000,4100,4200,4300,4400,4500,4600,4700,4800,4900,5000,5100,5200,5300,5400,5500,5600,5700,5800,5900,6000,6100,6200,6300,6400,6500,6600,6700,6800,6900,7000,7100,7200,7300,7400,7500,7600,7700,7800,7900,8000,8100,8200,8300,8400,8500,8600,8700,8800,8900,9000,9100,9200,9300,9400,9500,9600,9700,9800,9900,10000,10100,10200,10300,10400,10500,10600,10700,10800,10900,11000,11100,11200,11300,11400,11500,11600,11700,11800,11900,12000,12100,12200,12300,12400,12500,12600,12700,12800,12900,13000,13100,13200,13300,13400,13500,13600,13700,13800,13900,14000,14100,14200,14300,14400,14500,14600,14700,14800,14900,15000,15100,15200,15300,15400,15500,15600,15700,15800,15900,16000,16100,16200,16300,16400,16500,16600,16700,16800,16900,17000,17100,17200,17300,17400,17500,17600,17700,17800,17900,18000,18100,18200,18300,18400,18500,18600,18700,18800,18900,19000,19100,19200,19300,19400,19500,19600,19700,19800,19900,20000,20100,20200,20300,20400,20500,20600,20700,20800,20900,21000,21100,21200,21300,21400,21500,21600,21700,21800,21900,22000,22100,22200,22300,22400,22500,22600,22700,22800,22900,23000,23100,23200,23300,23400,23500,23600,23700,23800,23900,24000,24100,24200,24300,24400,24500,24600,24700,24800,24900,25000,25100,25200,25300,25400,25500,25600,25700,25800,25900,26000,26100,26200,26300,26400,26500,26600,26700,26800,26900,27000,27100,27200,27300,27400
NW_003726568_1,Count,0,0,0,0,0,0,0,0,0,0,0,0,0,0,0,0,0,0,0,0,0,0,0,0,0,0,0,0,0,0,0,0,0,0,0,0,0,0,0,0,0,0,0,0,0,0,0,0,0,0,0,0,0,0,0,0,0,0,0,0,0,0,0,0,0,0,0,0,0,0,0,0,0,0,0,0,0,0,0,0,0,0,0,0,0,0,0,0,0,0,0,0,0,0,0,0,0,0,0,0,0,0,0,0,0,0,0,0,0,0,0,0,0,0,0,0,0,0,0,0,0,0,0,0,0,0,0,0,0,0,0,0,0,0,0,0,0,0,0,0,0,0,0,0,0,0,0,0,0,0,0,0,0,0,0,0,0,0,0,0,0,0,0,0,0,0,0,0,0,0,0,0,0,0,0,0,0,0,0,0,0,0,0,0,0,0,0,0,0,0,0,0,0,0,0,0,0,0,0,0,0,0,0,0,0,0,0,0,0,0,0,0,0,0,0,0,0,0,0,0,0,0,0,0,0,0,0,0,0,0,0,0,0,0,0,0,0,0,0,0,0,0,0,0,0,0,0,0,0,0,0,0,0,0,0,0,0,0,0,0,0,0,0,0,0,0,0,3,0,0,2,1,1,0,0

	
```

```
		  

		NW_003726569_1, Position,0,100,200,300,400,500,600,700,800,900,1000,1100,1200,1300,1400,1500,1600,1700,1800,1900,2000,2100,2200,2300,2400,2500,2600,2700,2800,2900,3000,3100,3200,3300,3400,3500,3600,3700,3800,3900,4000,4100,4200,4300,4400,4500,4600,4700,4800,4900,5000,5100,5200,5300,5400,5500,5600,5700,5800,5900,6000,6100,6200,6300,6400,6500,6600,6700,6800,6900,7000,7100,7200,7300,7400,7500,7600,7700,7800,7900,8000,8100,8200,8300,8400,8500,8600,8700,8800,8900,9000,9100,9200,9300,9400,9500,9600,9700,9800,9900,10000,10100,10200,10300,10400,10500,10600,10700,10800,10900,11000,11100,11200,11300,11400,11500,11600,11700,11800,11900,12000,12100,12200,12300,12400,12500,12600,12700,12800,12900,13000,13100,13200,13300,13400,13500,13600,13700,13800,13900,14000,14100,14200,14300,14400,14500,14600,14700,14800,14900,15000,15100,15200,15300,15400,15500,15600,15700,15800,15900,16000,16100,16200,16300,16400,16500,16600,16700,16800,16900,17000,17100,17200,17300,17400,17500,17600,17700,17800,17900,18000,18100,18200,18300,18400,18500,18600,18700,18800,18900,19000,19100,19200,19300,19400,19500,19600,19700,19800,19900,20000,20100,20200,20300,20400,20500,20600,20700,20800,20900,21000,21100,21200,21300,21400,21500,21600,21700,21800,21900,22000,22100,22200,22300,22400,22500,22600,22700,22800,22900,23000,23100,23200,23300,23400,23500,23600,23700,23800,23900,24000,24100,24200,24300,24400,24500,24600,24700,24800,24900,25000,25100,25200,25300,25400,25500,25600,25700,25800,25900,26000,26100,26200,26300,26400,26500,26600,26700,26800,26900,27000,27100,27200,27300,27400,27500,27600,27700,27800,27900,28000
NW_003726569_1,Count,0,0,0,0,0,0,0,0,0,0,0,0,0,0,0,0,0,0,0,1,2,0,0,0,0,0,0,0,0,0,0,0,0,0,0,0,0,0,0,0,0,0,0,0,0,0,0,0,0,0,0,0,0,0,0,0,0,0,0,0,0,0,0,0,0,0,0,0,0,0,0,0,0,0,0,0,0,0,0,0,0,0,0,0,0,0,0,0,0,0,0,0,0,0,0,0,0,0,0,0,0,0,0,0,0,0,0,0,0,0,0,0,0,0,0,0,0,0,0,0,0,0,0,0,0,0,0,0,0,0,0,0,0,0,0,0,0,0,0,0,0,0,0,0,0,0,0,0,0,0,0,0,0,0,1,1,0,1,0,0,0,0,0,0,0,0,0,0,0,0,0,0,0,0,0,0,0,0,0,0,0,0,0,0,0,0,0,0,0,0,0,0,0,0,0,0,0,0,0,0,0,0,0,0,0,0,0,0,1,0,0,0,0,0,0,0,0,0,0,0,0,0,0,0,0,0,0,0,0,0,0,0,0,0,0,0,0,0,0,0,0,0,0,0,0,0,0,0,0,0,0,0,0,0,0,0,0,0,1,1,2,0,0,0,0,0,0,0,0,0,0,0,0,0,0,0,0,0,0,0,0

	
```

```
		  

		NW_003726570_1, Position,0,100,200,300,400,500,600,700,800,900,1000,1100,1200,1300,1400,1500,1600,1700,1800,1900,2000,2100,2200,2300,2400,2500,2600,2700,2800,2900,3000,3100,3200,3300,3400,3500,3600,3700,3800,3900,4000,4100,4200,4300,4400,4500,4600,4700,4800,4900,5000,5100,5200,5300,5400,5500,5600,5700,5800,5900,6000,6100,6200,6300,6400,6500,6600,6700,6800,6900,7000,7100,7200,7300,7400,7500,7600,7700,7800,7900,8000,8100,8200,8300,8400,8500,8600,8700,8800,8900,9000,9100,9200,9300,9400,9500,9600,9700,9800,9900,10000,10100,10200,10300,10400,10500,10600,10700,10800,10900,11000,11100,11200,11300,11400,11500,11600,11700,11800,11900,12000,12100,12200,12300,12400,12500,12600,12700,12800,12900,13000,13100,13200,13300,13400,13500,13600,13700,13800,13900,14000,14100,14200,14300,14400,14500,14600,14700,14800,14900,15000,15100,15200,15300,15400,15500,15600,15700,15800,15900,16000,16100,16200,16300,16400,16500,16600,16700,16800,16900,17000,17100,17200,17300,17400,17500,17600,17700,17800,17900,18000,18100,18200,18300,18400,18500,18600,18700,18800,18900,19000,19100,19200,19300,19400,19500,19600,19700,19800,19900,20000,20100,20200,20300,20400,20500,20600,20700,20800,20900,21000,21100,21200,21300,21400,21500,21600,21700,21800,21900,22000,22100,22200,22300,22400,22500,22600,22700,22800,22900,23000,23100,23200,23300,23400,23500,23600,23700,23800,23900,24000,24100,24200,24300,24400,24500,24600,24700,24800,24900,25000,25100,25200,25300,25400,25500,25600,25700,25800,25900,26000,26100,26200,26300,26400,26500,26600,26700,26800,26900,27000,27100,27200,27300
NW_003726570_1,Count,0,0,0,0,0,0,0,0,0,0,0,0,0,0,0,0,0,0,0,0,0,0,0,0,0,0,0,0,0,0,0,0,0,0,0,0,0,0,0,0,0,0,0,0,0,0,0,0,0,0,0,0,0,0,0,0,0,0,0,0,0,0,0,0,0,0,0,0,0,0,0,0,0,0,0,0,0,0,0,0,0,0,0,0,0,0,0,0,0,0,0,0,0,0,0,0,0,0,0,0,0,0,0,0,0,0,0,0,0,0,0,0,0,0,0,0,0,0,0,0,0,0,0,0,0,0,0,0,0,0,0,0,0,0,0,0,0,0,1,2,0,0,0,0,0,0,1,2,1,0,0,0,0,0,0,0,0,0,0,0,0,0,0,0,0,0,0,0,0,0,1,0,0,0,0,0,0,0,0,0,0,0,0,0,0,0,0,0,0,0,0,0,0,0,0,0,0,0,0,0,0,0,0,0,0,0,0,0,0,0,0,0,0,0,0,0,0,0,0,0,0,0,0,0,0,0,0,0,0,0,0,0,0,0,0,0,0,0,0,0,0,0,0,0,0,0,0,0,0,0,0,0,0,1,0,0,0,0,0,0,0,0,0,0,0,0,0,0,0,0,0,0,0,0

	
```

```
		  

		NW_003726575_1, Position,0,1000,2000,3000,4000,5000,6000,7000,8000,9000,10000,11000,12000,13000,14000,15000,16000,17000,18000,19000,20000,21000,22000,23000,24000,25000,26000,27000,28000,29000,30000,31000,32000
NW_003726575_1,Count,0,0,0,0,0,0,0,0,0,0,0,0,0,0,0,0,4,1,0,0,0,0,0,0,0,0,0,0,0,0,0,0,0

	
```

```
		  

		NW_003726584_1, Position,0,100,200,300,400,500,600,700,800,900,1000,1100,1200,1300,1400,1500,1600,1700,1800,1900,2000,2100,2200,2300,2400,2500,2600,2700,2800,2900,3000,3100,3200,3300,3400,3500,3600,3700,3800,3900,4000,4100,4200,4300,4400,4500,4600,4700,4800,4900,5000,5100,5200,5300,5400,5500,5600,5700,5800,5900,6000,6100,6200,6300,6400,6500,6600,6700,6800,6900,7000,7100,7200,7300,7400,7500,7600,7700,7800,7900,8000,8100,8200,8300,8400,8500,8600,8700,8800,8900,9000,9100,9200,9300,9400,9500,9600,9700,9800,9900,10000,10100,10200,10300,10400,10500,10600,10700,10800,10900,11000,11100,11200,11300,11400,11500,11600,11700,11800,11900,12000,12100,12200,12300,12400,12500,12600,12700,12800,12900,13000,13100,13200,13300,13400,13500,13600,13700,13800,13900,14000,14100,14200,14300,14400,14500,14600,14700,14800,14900,15000,15100,15200,15300,15400,15500,15600,15700,15800,15900,16000,16100,16200,16300,16400,16500,16600,16700,16800,16900,17000,17100,17200,17300,17400,17500,17600,17700,17800,17900,18000,18100,18200,18300,18400,18500,18600,18700,18800,18900,19000,19100,19200,19300,19400,19500,19600,19700,19800,19900,20000,20100,20200,20300,20400,20500,20600,20700,20800,20900,21000,21100,21200,21300,21400,21500,21600,21700,21800,21900,22000,22100,22200,22300,22400,22500,22600,22700,22800,22900,23000,23100,23200,23300,23400,23500,23600,23700,23800,23900,24000,24100,24200,24300,24400,24500,24600,24700,24800,24900,25000,25100,25200,25300,25400,25500,25600,25700,25800,25900,26000,26100,26200,26300,26400,26500,26600,26700,26800
NW_003726584_1,Count,0,0,0,0,0,0,0,0,0,0,0,0,0,0,0,0,0,0,0,0,0,0,0,0,0,0,0,0,0,0,0,0,0,0,0,0,0,0,0,0,0,0,0,0,0,0,0,0,0,0,0,0,0,0,0,0,0,0,0,0,0,0,0,0,0,0,0,0,0,0,0,0,0,0,0,0,0,0,0,0,0,0,0,0,0,0,0,0,0,0,0,0,0,0,0,0,0,0,0,0,0,0,0,0,0,0,0,0,0,0,0,0,0,0,0,0,0,0,0,0,0,0,0,0,0,0,0,0,0,0,0,0,0,0,0,0,0,0,0,0,0,0,0,0,0,0,0,0,0,0,0,0,0,0,0,0,0,0,0,0,0,0,0,0,0,0,0,0,0,0,0,0,0,0,0,0,0,0,0,0,0,0,0,0,0,0,0,0,0,0,0,0,0,0,0,0,0,0,2,0,0,0,0,0,0,0,0,0,0,0,0,0,0,0,0,0,0,0,0,0,0,0,0,0,0,0,0,0,0,0,0,0,0,0,0,0,0,0,0,0,0,0,0,0,0,0,0,0,0,0,0,0,0,0,0,0,0,0,0,0,0,0,0,0,0,0,0,0,0

	
```

```
		  

		NW_003726588_1, Position,0,100,200,300,400,500,600,700,800,900,1000,1100,1200,1300,1400,1500,1600,1700,1800,1900,2000,2100,2200,2300,2400,2500,2600,2700,2800,2900,3000,3100,3200,3300,3400,3500,3600,3700,3800,3900,4000,4100,4200,4300,4400,4500,4600,4700,4800,4900,5000,5100,5200,5300,5400,5500,5600,5700,5800,5900,6000,6100,6200,6300,6400,6500,6600,6700,6800,6900,7000,7100,7200,7300,7400,7500,7600,7700,7800,7900,8000,8100,8200,8300,8400,8500,8600,8700,8800,8900,9000,9100,9200,9300,9400,9500,9600,9700,9800,9900,10000,10100,10200,10300,10400,10500,10600,10700,10800,10900,11000,11100,11200,11300,11400,11500,11600,11700,11800,11900,12000,12100,12200,12300,12400,12500,12600,12700,12800,12900,13000,13100,13200,13300,13400,13500,13600,13700,13800,13900,14000,14100,14200,14300,14400,14500,14600,14700,14800,14900,15000,15100,15200,15300,15400,15500,15600,15700,15800,15900,16000,16100,16200,16300,16400,16500,16600,16700,16800,16900,17000,17100,17200,17300,17400,17500,17600,17700,17800,17900,18000,18100,18200,18300,18400,18500,18600,18700,18800,18900,19000,19100,19200,19300,19400,19500,19600,19700,19800,19900,20000,20100,20200,20300,20400,20500,20600,20700,20800,20900,21000,21100,21200,21300,21400,21500,21600,21700,21800,21900,22000,22100,22200,22300,22400,22500,22600,22700,22800,22900,23000,23100,23200,23300,23400,23500,23600,23700,23800,23900,24000,24100,24200,24300,24400,24500,24600,24700,24800,24900,25000,25100,25200,25300,25400,25500,25600,25700,25800,25900,26000,26100,26200,26300,26400,26500,26600,26700,26800,26900,27000,27100,27200,27300,27400,27500,27600,27700,27800,27900,28000,28100,28200,28300,28400,28500,28600,28700,28800,28900,29000,29100,29200,29300,29400,29500
NW_003726588_1,Count,0,0,0,3,1,0,0,0,0,0,0,2,0,0,0,0,0,0,0,0,0,0,0,0,0,0,0,0,0,0,0,0,0,0,0,0,0,0,0,0,0,0,0,0,0,0,0,0,0,0,0,0,0,0,0,0,0,0,0,0,0,0,0,0,0,1,0,0,0,0,0,0,0,0,0,0,0,0,0,0,0,0,0,0,0,0,0,0,0,0,0,0,0,0,0,0,0,0,0,0,0,0,0,0,0,0,0,0,0,0,0,0,0,0,0,0,0,0,0,0,0,0,0,0,0,0,0,0,0,0,0,0,0,0,0,0,0,0,0,0,0,0,0,0,0,0,0,0,0,0,0,0,0,1,1,1,0,0,0,0,0,0,0,0,0,0,0,0,0,0,0,0,0,0,0,0,0,0,1,1,0,0,0,0,0,0,0,0,0,0,0,0,0,0,0,0,0,0,0,0,0,0,0,0,0,0,0,0,0,0,0,0,0,0,0,0,0,0,0,0,0,0,0,0,0,0,0,0,0,0,0,0,0,0,0,0,0,0,0,0,0,0,0,0,0,0,0,0,0,0,0,0,0,0,0,0,0,0,0,0,0,0,0,0,0,0,0,0,0,0,0,0,0,0,0,0,0,0,0,0,0,0,0,0,0,0,0,0,0,0,0,0,0,0,0,0

	
```

```
		  

		NW_003726589_1, Position,0,100,200,300,400,500,600,700,800,900,1000,1100,1200,1300,1400,1500,1600,1700,1800,1900,2000,2100,2200,2300,2400,2500,2600,2700,2800,2900,3000,3100,3200,3300,3400,3500,3600,3700,3800,3900,4000,4100,4200,4300,4400,4500,4600,4700,4800,4900,5000,5100,5200,5300,5400,5500,5600,5700,5800,5900,6000,6100,6200,6300,6400,6500,6600,6700,6800,6900,7000,7100,7200,7300,7400,7500,7600,7700,7800,7900,8000,8100,8200,8300,8400,8500,8600,8700,8800,8900,9000,9100,9200,9300,9400,9500,9600,9700,9800,9900,10000,10100,10200,10300,10400,10500,10600,10700,10800,10900,11000,11100,11200,11300,11400,11500,11600,11700,11800,11900,12000,12100,12200,12300,12400,12500,12600,12700,12800,12900,13000,13100,13200,13300,13400,13500,13600,13700,13800,13900,14000,14100,14200,14300,14400,14500,14600,14700,14800,14900,15000,15100,15200,15300,15400,15500,15600,15700,15800,15900,16000,16100,16200,16300,16400,16500,16600,16700,16800,16900,17000,17100,17200,17300,17400,17500,17600,17700,17800,17900,18000,18100,18200,18300,18400,18500,18600,18700,18800,18900,19000,19100,19200,19300,19400,19500,19600,19700,19800,19900,20000,20100,20200,20300,20400,20500,20600,20700,20800,20900,21000,21100,21200,21300,21400,21500,21600,21700,21800,21900,22000,22100,22200,22300,22400,22500,22600,22700,22800,22900,23000,23100,23200,23300,23400,23500,23600,23700,23800,23900,24000,24100,24200,24300,24400,24500,24600,24700,24800,24900,25000,25100,25200,25300,25400,25500,25600,25700,25800,25900,26000,26100,26200,26300,26400,26500,26600,26700,26800,26900,27000,27100,27200,27300,27400,27500,27600,27700,27800,27900,28000,28100,28200,28300,28400,28500,28600,28700
NW_003726589_1,Count,0,0,0,0,1,1,2,0,2,1,0,2,1,1,1,2,0,3,0,1,0,4,2,1,0,3,1,0,0,1,0,0,0,0,1,0,1,2,1,1,2,1,0,0,2,0,2,0,0,0,0,0,0,1,0,0,1,0,0,1,2,0,2,0,0,0,1,1,0,1,0,1,1,1,2,0,0,0,0,3,1,0,0,0,1,0,1,1,0,0,0,1,1,2,1,0,0,0,0,0,0,1,0,3,0,1,2,0,0,0,0,0,1,2,2,1,1,0,0,2,3,0,0,0,0,0,0,1,1,1,0,0,1,1,2,2,0,0,2,0,0,1,0,1,1,1,3,1,1,0,1,0,0,2,0,0,1,0,0,0,1,0,0,1,0,1,2,0,0,2,1,1,1,1,1,0,1,1,0,1,0,0,0,0,1,0,0,2,1,2,1,1,0,1,0,0,1,1,1,1,1,2,2,1,2,0,0,0,1,1,0,1,2,0,0,0,4,0,1,0,1,1,1,3,1,2,1,4,0,3,1,1,1,0,1,2,0,0,4,0,1,1,0,2,0,0,1,2,0,1,0,0,0,0,0,0,0,0,0,0,0,0,0,1,3,3,3,1,0,0,3,0,0,3,1,0,2,0,1,2,3,0,0,0,0,0,0,0

	
```

```
		  

		NW_003726592_1, Position,0,100,200,300,400,500,600,700,800,900,1000,1100,1200,1300,1400,1500,1600,1700,1800,1900,2000,2100,2200,2300,2400,2500,2600,2700,2800,2900,3000,3100,3200,3300,3400,3500,3600,3700,3800,3900,4000,4100,4200,4300,4400,4500,4600,4700,4800,4900,5000,5100,5200,5300,5400,5500,5600,5700,5800,5900,6000,6100,6200,6300,6400,6500,6600,6700,6800,6900,7000,7100,7200,7300,7400,7500,7600,7700,7800,7900,8000,8100,8200,8300,8400,8500,8600,8700,8800,8900,9000,9100,9200,9300,9400,9500,9600,9700,9800,9900,10000,10100,10200,10300,10400,10500,10600,10700,10800,10900,11000,11100,11200,11300,11400,11500,11600,11700,11800,11900,12000,12100,12200,12300,12400,12500,12600,12700,12800,12900,13000,13100,13200,13300,13400,13500,13600,13700,13800,13900,14000,14100,14200,14300,14400,14500,14600,14700,14800,14900,15000,15100,15200,15300,15400,15500,15600,15700,15800,15900,16000,16100,16200,16300,16400,16500,16600,16700,16800,16900,17000,17100,17200,17300,17400,17500,17600,17700,17800,17900,18000,18100,18200,18300,18400,18500,18600,18700,18800,18900,19000,19100,19200,19300,19400,19500,19600,19700,19800,19900,20000,20100,20200,20300,20400,20500,20600,20700,20800,20900,21000,21100,21200,21300,21400,21500,21600,21700,21800,21900,22000,22100,22200,22300,22400,22500,22600,22700,22800,22900,23000,23100,23200,23300,23400,23500,23600,23700,23800,23900,24000,24100,24200,24300,24400,24500,24600,24700,24800,24900,25000,25100,25200,25300,25400,25500,25600,25700,25800,25900,26000,26100,26200,26300,26400,26500
NW_003726592_1,Count,0,0,0,0,0,0,0,0,0,0,0,0,0,0,0,0,0,0,0,0,0,0,0,0,0,0,0,0,0,0,0,0,0,0,0,0,0,0,1,2,0,0,0,0,0,0,0,0,1,1,0,4,0,0,0,0,2,1,0,1,2,0,0,3,1,0,0,0,0,0,0,0,0,0,0,0,0,0,0,0,0,0,0,0,0,0,0,0,0,0,0,0,0,0,0,0,0,0,0,0,0,0,0,0,0,0,1,0,0,0,1,1,2,1,1,1,0,1,1,0,0,0,0,1,1,0,0,4,0,1,0,0,0,1,0,1,1,2,0,0,1,0,0,1,0,0,0,2,1,2,1,0,2,1,1,0,1,0,1,1,2,0,1,0,0,1,0,1,0,1,2,1,1,1,0,4,2,0,0,0,0,2,0,0,2,0,1,1,1,1,1,2,0,0,1,0,1,0,0,0,0,1,0,1,1,1,0,1,0,0,0,1,1,0,0,1,0,2,0,0,0,0,0,0,0,0,0,0,0,0,0,0,0,0,0,0,0,0,0,0,0,0,0,0,0,0,0,0,0,0,0,0,0,0,0,0,0,0,0,0,0,0,0,0,0,0

	
```

```
		  

		NW_003726593_1, Position,0,1000,2000,3000,4000,5000,6000,7000,8000,9000,10000,11000,12000,13000,14000,15000,16000,17000,18000,19000,20000,21000,22000,23000,24000,25000,26000,27000,28000,29000,30000,31000,32000,33000,34000,35000,36000,37000,38000,39000,40000,41000,42000,43000,44000
NW_003726593_1,Count,0,0,0,0,0,0,0,0,5,9,0,0,0,0,0,0,0,0,0,0,0,0,0,0,0,0,0,0,0,0,0,0,0,0,0,0,0,0,0,0,0,0,0,0,0

	
```

```
		  

		NW_003726594_1, Position,0,1000,2000,3000,4000,5000,6000,7000,8000,9000,10000,11000,12000,13000,14000,15000,16000,17000,18000,19000,20000,21000,22000,23000,24000,25000,26000,27000,28000,29000,30000,31000,32000,33000
NW_003726594_1,Count,3,0,0,0,0,0,0,0,0,0,0,0,0,0,0,0,0,0,0,0,0,0,0,0,0,0,0,0,0,0,0,0,0,0

	
```

```
		  

		NW_003726597_1, Position,0,100,200,300,400,500,600,700,800,900,1000,1100,1200,1300,1400,1500,1600,1700,1800,1900,2000,2100,2200,2300,2400,2500,2600,2700,2800,2900,3000,3100,3200,3300,3400,3500,3600,3700,3800,3900,4000,4100,4200,4300,4400,4500,4600,4700,4800,4900,5000,5100,5200,5300,5400,5500,5600,5700,5800,5900,6000,6100,6200,6300,6400,6500,6600,6700,6800,6900,7000,7100,7200,7300,7400,7500,7600,7700,7800,7900,8000,8100,8200,8300,8400,8500,8600,8700,8800,8900,9000,9100,9200,9300,9400,9500,9600,9700,9800,9900,10000,10100,10200,10300,10400,10500,10600,10700,10800,10900,11000,11100,11200,11300,11400,11500,11600,11700,11800,11900,12000,12100,12200,12300,12400,12500,12600,12700,12800,12900,13000,13100,13200,13300,13400,13500,13600,13700,13800,13900,14000,14100,14200,14300,14400,14500,14600,14700,14800,14900,15000,15100,15200,15300,15400,15500,15600,15700,15800,15900,16000,16100,16200,16300,16400,16500,16600,16700,16800,16900,17000,17100,17200,17300,17400,17500,17600,17700,17800,17900,18000,18100,18200,18300,18400,18500,18600,18700,18800,18900,19000,19100,19200,19300,19400,19500,19600,19700,19800,19900,20000,20100,20200,20300,20400,20500,20600,20700,20800,20900,21000,21100,21200,21300,21400,21500,21600,21700,21800,21900,22000,22100,22200,22300,22400,22500,22600,22700,22800,22900,23000,23100,23200,23300,23400,23500,23600,23700,23800,23900,24000,24100,24200,24300,24400,24500,24600,24700,24800,24900,25000,25100,25200,25300,25400,25500,25600,25700,25800,25900,26000,26100,26200,26300
NW_003726597_1,Count,0,0,0,0,0,0,0,0,0,0,0,0,0,0,0,0,0,0,0,0,0,0,0,0,0,0,0,0,0,0,0,0,0,0,0,0,0,0,0,0,0,0,0,0,0,0,0,0,0,0,0,0,0,0,0,0,0,0,0,0,0,0,0,0,0,0,0,0,0,0,0,0,0,0,0,0,0,0,0,0,0,0,0,0,0,0,0,0,0,0,0,0,0,0,0,0,0,0,0,0,0,0,0,0,0,0,0,0,0,0,0,0,0,0,0,0,0,0,0,0,0,0,0,0,0,0,0,0,0,0,0,0,0,0,0,0,0,0,0,0,0,0,0,0,0,0,0,0,0,0,0,0,0,0,0,0,0,0,0,0,0,0,0,0,0,0,0,0,0,0,0,0,0,0,0,0,0,0,0,0,0,0,0,0,0,0,0,0,0,0,0,0,0,0,0,0,0,0,0,0,0,0,0,0,0,0,0,0,0,0,0,0,0,0,0,0,0,0,0,0,0,0,1,3,0,0,0,0,0,0,0,0,0,0,0,0,0,0,0,0,0,0,0,0,0,0,0,0,0,0,0,0,0,0,0,0,0,0,0,0,0,0,0,0

	
```

```
		  

		NW_003726599_1, Position,0,100,200,300,400,500,600,700,800,900,1000,1100,1200,1300,1400,1500,1600,1700,1800,1900,2000,2100,2200,2300,2400,2500,2600,2700,2800,2900,3000,3100,3200,3300,3400,3500,3600,3700,3800,3900,4000,4100,4200,4300,4400,4500,4600,4700,4800,4900,5000,5100,5200,5300,5400,5500,5600,5700,5800,5900,6000,6100,6200,6300,6400,6500,6600,6700,6800,6900,7000,7100,7200,7300,7400,7500,7600,7700,7800,7900,8000,8100,8200,8300,8400,8500,8600,8700,8800,8900,9000,9100,9200,9300,9400,9500,9600,9700,9800,9900,10000,10100,10200,10300,10400,10500,10600,10700,10800,10900,11000,11100,11200,11300,11400,11500,11600,11700,11800,11900,12000,12100,12200,12300,12400,12500,12600,12700,12800,12900,13000,13100,13200,13300,13400,13500,13600,13700,13800,13900,14000,14100,14200,14300,14400,14500,14600,14700,14800,14900,15000,15100,15200,15300,15400,15500,15600,15700,15800,15900,16000,16100,16200,16300,16400,16500,16600,16700,16800,16900,17000,17100,17200,17300,17400,17500,17600,17700,17800,17900,18000,18100,18200,18300,18400,18500,18600,18700,18800,18900,19000,19100,19200,19300,19400,19500,19600,19700,19800,19900,20000,20100,20200,20300,20400,20500,20600,20700,20800,20900,21000,21100,21200,21300,21400,21500,21600,21700,21800,21900,22000,22100,22200,22300,22400,22500,22600,22700,22800,22900,23000,23100,23200,23300,23400,23500,23600,23700,23800,23900,24000,24100,24200,24300,24400,24500,24600,24700,24800,24900,25000,25100,25200,25300,25400,25500,25600,25700,25800,25900,26000,26100,26200,26300,26400,26500,26600,26700,26800,26900,27000,27100,27200,27300
NW_003726599_1,Count,0,0,0,0,0,0,3,0,0,2,1,1,3,0,0,0,0,0,0,0,0,0,0,0,0,0,0,0,0,0,0,0,0,0,0,2,1,3,0,2,2,0,0,0,0,0,0,0,0,1,2,2,0,0,0,0,0,0,0,0,2,2,1,3,2,1,2,1,3,1,0,0,0,0,0,0,0,0,0,0,0,0,0,0,0,0,0,0,0,0,0,0,0,0,0,0,0,0,0,0,0,0,1,0,1,0,3,0,2,2,1,1,3,3,1,1,1,2,3,1,4,1,1,1,0,0,0,0,0,0,0,0,0,0,0,0,0,0,0,0,2,1,1,0,0,0,0,0,0,0,0,0,0,0,0,0,0,0,0,0,0,2,2,0,0,0,0,0,0,3,0,0,0,0,0,0,0,0,0,0,0,0,0,0,0,0,0,0,0,0,0,0,0,0,0,0,0,0,0,3,3,1,0,0,0,0,0,0,3,0,0,0,0,0,1,2,2,0,0,0,1,3,0,3,0,0,0,0,0,2,3,0,0,0,1,2,1,1,0,0,0,2,1,0,0,0,0,3,0,1,3,2,0,2,0,0,1,0,0,0,0,0,0,0,0,0,0,0,0,0,0,0,0,0

	
```

```
		  

		NW_003726600_1, Position,0,1000,2000,3000,4000,5000,6000,7000,8000,9000,10000,11000,12000,13000,14000,15000,16000,17000,18000,19000,20000,21000,22000,23000,24000,25000,26000,27000,28000,29000,30000,31000,32000,33000,34000,35000,36000
NW_003726600_1,Count,2,3,5,2,1,0,0,0,0,0,0,0,0,0,0,0,6,0,10,8,0,0,0,0,0,0,0,0,0,0,0,0,0,0,0,2,3

	
```

```
		  

		NW_003726602_1, Position,0,100,200,300,400,500,600,700,800,900,1000,1100,1200,1300,1400,1500,1600,1700,1800,1900,2000,2100,2200,2300,2400,2500,2600,2700,2800,2900,3000,3100,3200,3300,3400,3500,3600,3700,3800,3900,4000,4100,4200,4300,4400,4500,4600,4700,4800,4900,5000,5100,5200,5300,5400,5500,5600,5700,5800,5900,6000,6100,6200,6300,6400,6500,6600,6700,6800,6900,7000,7100,7200,7300,7400,7500,7600,7700,7800,7900,8000,8100,8200,8300,8400,8500,8600,8700,8800,8900,9000,9100,9200,9300,9400,9500,9600,9700,9800,9900,10000,10100,10200,10300,10400,10500,10600,10700,10800,10900,11000,11100,11200,11300,11400,11500,11600,11700,11800,11900,12000,12100,12200,12300,12400,12500,12600,12700,12800,12900,13000,13100,13200,13300,13400,13500,13600,13700,13800,13900,14000,14100,14200,14300,14400,14500,14600,14700,14800,14900,15000,15100,15200,15300,15400,15500,15600,15700,15800,15900,16000,16100,16200,16300,16400,16500,16600,16700,16800,16900,17000,17100,17200,17300,17400,17500,17600,17700,17800,17900,18000,18100,18200,18300,18400,18500,18600,18700,18800,18900,19000,19100,19200,19300,19400,19500,19600,19700,19800,19900,20000,20100,20200,20300,20400,20500,20600,20700,20800,20900,21000,21100,21200,21300,21400,21500,21600,21700,21800,21900,22000,22100,22200,22300,22400,22500,22600,22700,22800,22900,23000,23100,23200,23300,23400,23500,23600,23700,23800,23900,24000,24100,24200,24300,24400,24500,24600,24700,24800,24900,25000,25100,25200,25300,25400,25500,25600,25700,25800,25900,26000,26100
NW_003726602_1,Count,0,1,3,1,2,1,0,0,1,4,3,1,2,1,2,1,1,1,1,2,0,1,0,0,1,1,0,0,1,1,3,2,2,2,0,1,0,3,1,1,0,1,0,1,0,0,0,2,1,0,1,0,1,2,0,3,0,0,0,1,2,0,1,0,0,0,0,1,2,0,3,3,0,0,0,0,0,0,0,0,0,0,0,0,0,0,0,1,1,2,0,0,0,2,3,1,2,1,0,2,1,1,1,0,0,2,1,1,0,3,0,0,0,0,0,0,0,0,0,0,0,0,0,0,0,0,0,0,0,0,0,0,0,0,0,0,0,0,0,1,0,0,2,1,1,1,1,0,0,0,0,0,0,0,0,1,1,0,1,1,0,0,0,0,1,0,0,0,0,0,0,0,0,0,0,2,0,1,1,0,0,0,0,1,2,0,2,0,0,0,0,0,0,3,0,1,0,2,1,0,1,0,0,0,0,1,2,1,1,1,1,1,1,1,1,1,0,1,3,0,0,0,0,0,0,0,2,1,0,0,0,0,0,0,0,0,0,0,0,1,0,1,0,0,0,0,0,1,0,0,0,0,0,0,0,0,0,0,0,0,0,0

	
```

```
		  

		NW_003726605_1, Position,0,1000,2000,3000,4000,5000,6000,7000,8000,9000,10000,11000,12000,13000,14000,15000,16000,17000,18000,19000,20000,21000,22000,23000,24000,25000,26000,27000,28000,29000,30000,31000,32000,33000,34000,35000,36000,37000,38000,39000,40000,41000
NW_003726605_1,Count,0,0,0,0,0,0,0,0,0,0,0,0,0,0,0,1,0,2,0,7,0,0,0,0,0,0,0,0,0,0,0,0,0,0,0,0,0,0,0,0,0,0

	
```

```
		  

		NW_003726610_1, Position,0,100,200,300,400,500,600,700,800,900,1000,1100,1200,1300,1400,1500,1600,1700,1800,1900,2000,2100,2200,2300,2400,2500,2600,2700,2800,2900,3000,3100,3200,3300,3400,3500,3600,3700,3800,3900,4000,4100,4200,4300,4400,4500,4600,4700,4800,4900,5000,5100,5200,5300,5400,5500,5600,5700,5800,5900,6000,6100,6200,6300,6400,6500,6600,6700,6800,6900,7000,7100,7200,7300,7400,7500,7600,7700,7800,7900,8000,8100,8200,8300,8400,8500,8600,8700,8800,8900,9000,9100,9200,9300,9400,9500,9600,9700,9800,9900,10000,10100,10200,10300,10400,10500,10600,10700,10800,10900,11000,11100,11200,11300,11400,11500,11600,11700,11800,11900,12000,12100,12200,12300,12400,12500,12600,12700,12800,12900,13000,13100,13200,13300,13400,13500,13600,13700,13800,13900,14000,14100,14200,14300,14400,14500,14600,14700,14800,14900,15000,15100,15200,15300,15400,15500,15600,15700,15800,15900,16000,16100,16200,16300,16400,16500,16600,16700,16800,16900,17000,17100,17200,17300,17400,17500,17600,17700,17800,17900,18000,18100,18200,18300,18400,18500,18600,18700,18800,18900,19000,19100,19200,19300,19400,19500,19600,19700,19800,19900,20000,20100,20200,20300,20400,20500,20600,20700,20800,20900,21000,21100,21200,21300,21400,21500,21600,21700,21800,21900,22000,22100,22200,22300,22400,22500,22600,22700,22800,22900,23000,23100,23200,23300,23400,23500,23600,23700,23800,23900,24000,24100,24200,24300,24400,24500,24600,24700,24800,24900,25000,25100,25200,25300,25400,25500,25600,25700,25800,25900,26000,26100,26200,26300,26400,26500,26600,26700,26800,26900,27000,27100,27200,27300,27400,27500,27600,27700,27800,27900,28000,28100,28200,28300,28400,28500,28600,28700,28800
NW_003726610_1,Count,0,0,0,0,0,0,0,0,0,0,0,0,0,0,0,0,0,0,0,0,0,0,0,0,0,0,0,0,0,0,0,0,0,0,0,0,0,0,0,0,0,0,0,0,0,0,0,0,0,0,0,0,0,0,0,0,0,0,0,0,0,0,0,0,0,0,0,0,0,0,0,0,0,0,0,0,0,0,0,0,0,0,0,0,0,0,0,0,0,0,0,0,0,0,0,0,0,0,0,0,0,0,0,0,0,0,0,0,0,0,0,0,0,0,0,0,0,0,0,0,0,0,0,0,0,0,0,0,0,0,0,0,0,0,0,0,0,0,0,0,0,0,0,0,0,0,0,0,0,0,0,0,0,0,0,0,0,0,0,0,0,0,0,0,0,0,0,0,0,0,0,0,0,0,0,0,0,0,0,0,0,0,0,0,0,0,0,0,0,0,0,0,0,0,0,0,0,0,0,0,0,0,0,0,0,0,0,0,0,0,0,0,0,0,0,0,0,0,0,0,0,0,0,0,0,0,0,0,0,0,0,0,0,0,0,0,0,0,0,0,0,0,0,0,0,0,0,0,0,0,0,0,0,0,0,0,0,0,0,0,0,0,0,0,0,0,0,0,0,0,0,0,0,0,0,0,0,0,0,0,0,1,2,0,1,0,0,0,0

	
```

```
		  

		NW_003726611_1, Position,0,100,200,300,400,500,600,700,800,900,1000,1100,1200,1300,1400,1500,1600,1700,1800,1900,2000,2100,2200,2300,2400,2500,2600,2700,2800,2900,3000,3100,3200,3300,3400,3500,3600,3700,3800,3900,4000,4100,4200,4300,4400,4500,4600,4700,4800,4900,5000,5100,5200,5300,5400,5500,5600,5700,5800,5900,6000,6100,6200,6300,6400,6500,6600,6700,6800,6900,7000,7100,7200,7300,7400,7500,7600,7700,7800,7900,8000,8100,8200,8300,8400,8500,8600,8700,8800,8900,9000,9100,9200,9300,9400,9500,9600,9700,9800,9900,10000,10100,10200,10300,10400,10500,10600,10700,10800,10900,11000,11100,11200,11300,11400,11500,11600,11700,11800,11900,12000,12100,12200,12300,12400,12500,12600,12700,12800,12900,13000,13100,13200,13300,13400,13500,13600,13700,13800,13900,14000,14100,14200,14300,14400,14500,14600,14700,14800,14900,15000,15100,15200,15300,15400,15500,15600,15700,15800,15900,16000,16100,16200,16300,16400,16500,16600,16700,16800,16900,17000,17100,17200,17300,17400,17500,17600,17700,17800,17900,18000,18100,18200,18300,18400,18500,18600,18700,18800,18900,19000,19100,19200,19300,19400,19500,19600,19700,19800,19900,20000,20100,20200,20300,20400,20500,20600,20700,20800,20900,21000,21100,21200,21300,21400,21500,21600,21700,21800,21900,22000,22100,22200,22300,22400,22500,22600,22700,22800,22900,23000,23100,23200,23300,23400,23500,23600,23700,23800,23900,24000,24100,24200,24300,24400,24500,24600,24700,24800,24900,25000,25100,25200,25300,25400,25500,25600,25700,25800,25900,26000
NW_003726611_1,Count,0,0,0,0,0,0,0,0,0,0,0,0,0,0,0,0,0,0,0,0,0,0,0,0,0,0,0,0,0,0,0,0,0,0,0,0,0,0,0,0,0,0,0,0,0,0,0,0,0,0,0,0,0,0,0,0,0,0,0,0,0,0,0,0,0,0,0,0,0,0,0,0,0,0,0,0,0,0,0,0,0,0,0,0,0,0,0,0,0,0,0,0,0,0,0,0,1,4,0,1,2,0,0,0,0,0,0,0,0,0,0,0,0,0,0,0,0,0,0,0,0,0,0,0,0,0,0,0,0,0,0,0,0,0,0,0,0,0,0,0,0,0,0,0,0,0,0,0,0,0,0,0,0,0,0,0,0,0,0,0,0,0,0,0,0,0,0,0,0,0,0,0,0,0,0,0,0,0,0,0,0,0,0,0,0,0,0,0,0,0,0,0,0,0,0,0,0,0,0,0,0,0,0,0,0,0,0,0,0,0,0,0,0,0,0,0,0,0,0,0,0,0,0,0,0,0,0,0,0,0,0,0,0,0,0,0,0,0,0,0,0,0,0,0,0,0,0,0,0,0,0,0,0,0,0,0,0,0,0,0,0

	
```

```
		  

		NW_003726613_1, Position,0,100,200,300,400,500,600,700,800,900,1000,1100,1200,1300,1400,1500,1600,1700,1800,1900,2000,2100,2200,2300,2400,2500,2600,2700,2800,2900,3000,3100,3200,3300,3400,3500,3600,3700,3800,3900,4000,4100,4200,4300,4400,4500,4600,4700,4800,4900,5000,5100,5200,5300,5400,5500,5600,5700,5800,5900,6000,6100,6200,6300,6400,6500,6600,6700,6800,6900,7000,7100,7200,7300,7400,7500,7600,7700,7800,7900,8000,8100,8200,8300,8400,8500,8600,8700,8800,8900,9000,9100,9200,9300,9400,9500,9600,9700,9800,9900,10000,10100,10200,10300,10400,10500,10600,10700,10800,10900,11000,11100,11200,11300,11400,11500,11600,11700,11800,11900,12000,12100,12200,12300,12400,12500,12600,12700,12800,12900,13000,13100,13200,13300,13400,13500,13600,13700,13800,13900,14000,14100,14200,14300,14400,14500,14600,14700,14800,14900,15000,15100,15200,15300,15400,15500,15600,15700,15800,15900,16000,16100,16200,16300,16400,16500,16600,16700,16800,16900,17000,17100,17200,17300,17400,17500,17600,17700,17800,17900,18000,18100,18200,18300,18400,18500,18600,18700,18800,18900,19000,19100,19200,19300,19400,19500,19600,19700,19800,19900,20000,20100,20200,20300,20400,20500,20600,20700,20800,20900,21000,21100,21200,21300,21400,21500,21600,21700,21800,21900,22000,22100,22200,22300,22400,22500,22600,22700,22800,22900,23000,23100,23200,23300,23400,23500,23600,23700,23800,23900,24000,24100,24200,24300,24400,24500,24600,24700,24800,24900,25000,25100,25200,25300,25400,25500,25600
NW_003726613_1,Count,0,0,0,0,0,0,0,0,0,0,0,0,0,1,0,1,0,1,0,2,1,0,0,1,0,0,0,0,1,0,0,1,0,0,0,0,0,0,0,0,1,0,0,1,1,3,1,0,0,0,0,0,0,0,0,1,1,2,0,0,0,0,1,0,0,0,1,2,0,2,0,1,0,2,0,0,0,1,0,0,0,2,0,0,0,0,0,0,0,0,1,1,0,1,0,1,0,2,0,0,0,2,2,0,1,1,2,1,2,4,0,1,1,0,1,0,0,1,1,0,0,0,3,1,0,0,1,0,0,0,0,0,0,0,0,0,0,0,0,0,0,0,0,0,2,0,0,0,1,1,1,0,0,0,0,0,2,0,1,2,0,1,0,0,0,0,0,0,1,0,0,1,1,2,0,0,0,0,1,0,0,1,0,1,1,0,0,2,0,3,0,1,0,1,2,0,1,1,0,0,0,0,1,0,0,0,0,0,0,0,2,1,1,0,2,0,1,1,0,0,1,0,0,3,1,0,0,0,0,0,0,2,0,2,0,0,0,0,0,0,1,2,0,0,0,0,0,0,0,0,0,0,0,0,0,0,0

	
```

```
		  

		NW_003726614_1, Position,0,1000,2000,3000,4000,5000,6000,7000,8000,9000,10000,11000,12000,13000,14000,15000,16000,17000,18000,19000,20000,21000,22000,23000,24000,25000,26000,27000,28000,29000,30000,31000,32000,33000,34000,35000,36000,37000,38000,39000,40000,41000,42000,43000,44000,45000
NW_003726614_1,Count,0,0,0,0,0,0,0,0,0,0,0,0,0,0,0,0,4,0,0,0,0,0,0,0,0,0,0,0,0,0,0,0,0,0,0,0,0,0,0,0,0,3,0,0,0,0

	
```

```
		  

		NW_003726619_1, Position,0,100,200,300,400,500,600,700,800,900,1000,1100,1200,1300,1400,1500,1600,1700,1800,1900,2000,2100,2200,2300,2400,2500,2600,2700,2800,2900,3000,3100,3200,3300,3400,3500,3600,3700,3800,3900,4000,4100,4200,4300,4400,4500,4600,4700,4800,4900,5000,5100,5200,5300,5400,5500,5600,5700,5800,5900,6000,6100,6200,6300,6400,6500,6600,6700,6800,6900,7000,7100,7200,7300,7400,7500,7600,7700,7800,7900,8000,8100,8200,8300,8400,8500,8600,8700,8800,8900,9000,9100,9200,9300,9400,9500,9600,9700,9800,9900,10000,10100,10200,10300,10400,10500,10600,10700,10800,10900,11000,11100,11200,11300,11400,11500,11600,11700,11800,11900,12000,12100,12200,12300,12400,12500,12600,12700,12800,12900,13000,13100,13200,13300,13400,13500,13600,13700,13800,13900,14000,14100,14200,14300,14400,14500,14600,14700,14800,14900,15000,15100,15200,15300,15400,15500,15600,15700,15800,15900,16000,16100,16200,16300,16400,16500,16600,16700,16800,16900,17000,17100,17200,17300,17400,17500,17600,17700,17800,17900,18000,18100,18200,18300,18400,18500,18600,18700,18800,18900,19000,19100,19200,19300,19400,19500,19600,19700,19800,19900,20000,20100,20200,20300,20400,20500,20600,20700,20800,20900,21000,21100,21200,21300,21400,21500,21600,21700,21800,21900,22000,22100,22200,22300,22400,22500,22600,22700,22800,22900,23000,23100,23200,23300,23400,23500,23600,23700,23800,23900,24000,24100,24200,24300,24400,24500,24600,24700,24800,24900,25000,25100,25200,25300,25400,25500
NW_003726619_1,Count,0,0,0,0,0,0,0,0,0,0,0,0,0,0,0,0,0,0,0,0,0,0,0,0,0,0,0,0,0,0,0,0,0,0,0,0,0,0,0,0,0,0,0,0,0,0,0,0,0,0,0,0,0,0,0,0,0,0,0,0,0,0,0,0,0,0,0,0,0,0,0,0,0,0,0,0,0,0,0,0,0,0,0,0,0,0,0,0,0,0,0,0,0,0,0,0,0,0,0,0,0,0,0,0,0,0,0,0,0,0,0,0,0,0,0,0,0,0,0,0,0,0,0,0,0,0,0,0,0,0,0,0,0,0,0,0,0,0,0,0,0,0,0,0,0,0,0,0,0,0,0,0,0,0,0,0,0,0,0,0,0,0,0,0,0,0,0,0,0,0,0,0,0,0,0,0,0,0,0,0,0,0,0,0,0,0,0,0,0,0,0,0,0,0,0,0,0,0,0,0,0,0,0,0,0,0,0,0,0,0,0,0,0,0,0,0,0,1,0,0,0,0,0,0,0,0,0,0,0,0,0,0,0,0,0,0,0,0,0,0,0,0,0,0,0,0,0,0,0,0,0,0,0,0,0,0

	
```

```
		  

		NW_003726620_1, Position,0,1000,2000,3000,4000,5000,6000,7000,8000,9000,10000,11000,12000,13000,14000,15000,16000,17000,18000,19000,20000,21000,22000,23000,24000,25000,26000,27000,28000,29000,30000,31000,32000,33000,34000,35000,36000,37000
NW_003726620_1,Count,0,0,0,0,0,0,0,0,0,0,0,0,0,0,0,0,0,0,0,0,0,0,0,0,0,0,0,0,0,0,0,2,0,0,0,0,0,0

	
```

```
		  

		NW_003726630_1, Position,0,100,200,300,400,500,600,700,800,900,1000,1100,1200,1300,1400,1500,1600,1700,1800,1900,2000,2100,2200,2300,2400,2500,2600,2700,2800,2900,3000,3100,3200,3300,3400,3500,3600,3700,3800,3900,4000,4100,4200,4300,4400,4500,4600,4700,4800,4900,5000,5100,5200,5300,5400,5500,5600,5700,5800,5900,6000,6100,6200,6300,6400,6500,6600,6700,6800,6900,7000,7100,7200,7300,7400,7500,7600,7700,7800,7900,8000,8100,8200,8300,8400,8500,8600,8700,8800,8900,9000,9100,9200,9300,9400,9500,9600,9700,9800,9900,10000,10100,10200,10300,10400,10500,10600,10700,10800,10900,11000,11100,11200,11300,11400,11500,11600,11700,11800,11900,12000,12100,12200,12300,12400,12500,12600,12700,12800,12900,13000,13100,13200,13300,13400,13500,13600,13700,13800,13900,14000,14100,14200,14300,14400,14500,14600,14700,14800,14900,15000,15100,15200,15300,15400,15500,15600,15700,15800,15900,16000,16100,16200,16300,16400,16500,16600,16700,16800,16900,17000,17100,17200,17300,17400,17500,17600,17700,17800,17900,18000,18100,18200,18300,18400,18500,18600,18700,18800,18900,19000,19100,19200,19300,19400,19500,19600,19700,19800,19900,20000,20100,20200,20300,20400,20500,20600,20700,20800,20900,21000,21100,21200,21300,21400,21500,21600,21700,21800,21900,22000,22100,22200,22300,22400,22500,22600,22700,22800,22900,23000,23100,23200,23300,23400,23500,23600,23700,23800,23900,24000,24100,24200,24300,24400,24500,24600,24700,24800,24900,25000,25100,25200,25300,25400,25500
NW_003726630_1,Count,0,0,0,0,0,0,0,0,0,0,0,0,0,0,0,0,0,0,0,0,0,0,0,0,0,0,0,0,0,0,0,0,0,0,0,0,0,0,0,0,0,0,0,0,0,0,3,1,0,0,0,0,0,0,0,0,0,0,0,0,0,0,0,0,0,0,0,0,0,0,0,0,0,0,0,0,0,0,0,0,0,0,0,0,0,0,0,0,0,0,0,0,1,0,0,0,0,0,0,0,0,0,0,0,0,0,0,0,0,0,0,0,0,0,0,0,0,0,0,0,0,0,0,0,0,0,0,0,0,0,0,0,0,0,0,0,0,0,0,0,0,0,0,0,0,0,0,0,0,0,0,0,0,0,0,0,0,0,0,0,0,0,0,0,0,0,0,0,0,0,0,0,0,0,0,0,0,0,0,0,0,2,0,0,0,0,0,0,0,0,3,0,0,0,0,0,0,0,0,0,0,0,0,0,0,0,0,0,0,0,0,0,0,0,0,0,0,0,0,0,0,0,0,0,0,0,0,0,0,0,0,0,1,0,0,0,0,0,0,0,0,0,0,0,0,0,0,0,0,0,0,0,0,0,0,0

	
```

```
		  

		NW_003726632_1, Position,0,100,200,300,400,500,600,700,800,900,1000,1100,1200,1300,1400,1500,1600,1700,1800,1900,2000,2100,2200,2300,2400,2500,2600,2700,2800,2900,3000,3100,3200,3300,3400,3500,3600,3700,3800,3900,4000,4100,4200,4300,4400,4500,4600,4700,4800,4900,5000,5100,5200,5300,5400,5500,5600,5700,5800,5900,6000,6100,6200,6300,6400,6500,6600,6700,6800,6900,7000,7100,7200,7300,7400,7500,7600,7700,7800,7900,8000,8100,8200,8300,8400,8500,8600,8700,8800,8900,9000,9100,9200,9300,9400,9500,9600,9700,9800,9900,10000,10100,10200,10300,10400,10500,10600,10700,10800,10900,11000,11100,11200,11300,11400,11500,11600,11700,11800,11900,12000,12100,12200,12300,12400,12500,12600,12700,12800,12900,13000,13100,13200,13300,13400,13500,13600,13700,13800,13900,14000,14100,14200,14300,14400,14500,14600,14700,14800,14900,15000,15100,15200,15300,15400,15500,15600,15700,15800,15900,16000,16100,16200,16300,16400,16500,16600,16700,16800,16900,17000,17100,17200,17300,17400,17500,17600,17700,17800,17900,18000,18100,18200,18300,18400,18500,18600,18700,18800,18900,19000,19100,19200,19300,19400,19500,19600,19700,19800,19900,20000,20100,20200,20300,20400,20500,20600,20700,20800,20900,21000,21100,21200,21300,21400,21500,21600,21700,21800,21900,22000,22100,22200,22300,22400,22500,22600,22700,22800,22900,23000,23100,23200,23300,23400,23500,23600,23700,23800,23900,24000,24100,24200,24300,24400,24500,24600,24700,24800
NW_003726632_1,Count,0,0,0,0,0,0,0,0,0,0,0,0,0,0,0,0,0,0,0,0,0,1,2,1,1,0,0,0,0,0,0,0,0,0,0,0,0,0,0,0,0,0,0,0,0,0,0,1,0,0,1,1,1,0,2,1,0,0,0,0,0,2,1,1,1,0,0,0,0,0,0,0,0,0,0,0,0,0,0,2,0,0,0,0,0,0,0,0,0,0,0,0,0,0,0,0,0,1,1,0,0,0,0,2,1,1,0,0,1,1,0,2,1,1,2,0,1,2,2,0,0,0,0,0,0,1,0,0,0,1,2,0,0,1,0,0,0,1,0,0,2,0,0,0,1,0,0,0,0,0,1,0,0,2,2,0,1,1,2,0,0,0,0,0,0,0,0,1,1,0,0,0,2,2,1,1,2,0,0,1,1,0,3,0,0,1,0,2,1,1,1,0,1,0,2,0,0,0,0,0,0,0,0,0,0,0,0,0,0,0,0,0,0,0,0,0,0,0,0,0,0,0,0,0,0,0,0,0,0,0,0,0,0,0,1,0,2,0,0,0,0,0,0,0,0,0,0,0,0

	
```

```
		  

		NW_003726636_1, Position,0,100,200,300,400,500,600,700,800,900,1000,1100,1200,1300,1400,1500,1600,1700,1800,1900,2000,2100,2200,2300,2400,2500,2600,2700,2800,2900,3000,3100,3200,3300,3400,3500,3600,3700,3800,3900,4000,4100,4200,4300,4400,4500,4600,4700,4800,4900,5000,5100,5200,5300,5400,5500,5600,5700,5800,5900,6000,6100,6200,6300,6400,6500,6600,6700,6800,6900,7000,7100,7200,7300,7400,7500,7600,7700,7800,7900,8000,8100,8200,8300,8400,8500,8600,8700,8800,8900,9000,9100,9200,9300,9400,9500,9600,9700,9800,9900,10000,10100,10200,10300,10400,10500,10600,10700,10800,10900,11000,11100,11200,11300,11400,11500,11600,11700,11800,11900,12000,12100,12200,12300,12400,12500,12600,12700,12800,12900,13000,13100,13200,13300,13400,13500,13600,13700,13800,13900,14000,14100,14200,14300,14400,14500,14600,14700,14800,14900,15000,15100,15200,15300,15400,15500,15600,15700,15800,15900,16000,16100,16200,16300,16400,16500,16600,16700,16800,16900,17000,17100,17200,17300,17400,17500,17600,17700,17800,17900,18000,18100,18200,18300,18400,18500,18600,18700,18800,18900,19000,19100,19200,19300,19400,19500,19600,19700,19800,19900,20000,20100,20200,20300,20400,20500,20600,20700,20800,20900,21000,21100,21200,21300,21400,21500,21600,21700,21800,21900,22000,22100,22200,22300,22400,22500,22600,22700,22800,22900,23000,23100,23200,23300,23400,23500,23600,23700,23800,23900,24000,24100,24200,24300,24400,24500,24600
NW_003726636_1,Count,0,1,0,2,1,3,1,0,0,1,0,0,0,1,0,0,0,0,1,1,2,2,0,1,2,0,2,1,2,2,0,0,0,1,0,2,0,2,0,1,0,0,0,0,0,1,2,0,0,1,2,0,4,1,2,0,0,1,0,0,0,2,2,0,0,1,3,1,0,1,0,2,0,0,1,1,2,0,1,2,1,1,1,1,2,0,1,3,1,3,3,0,0,0,0,2,0,1,1,0,0,3,1,3,1,2,0,0,2,1,1,0,4,0,0,4,3,1,3,1,1,0,2,1,1,2,0,2,0,2,0,1,1,3,2,1,3,0,3,0,2,0,1,0,0,0,0,0,0,0,0,0,0,0,0,0,0,0,0,0,0,0,4,1,0,0,2,0,0,0,0,0,0,0,0,0,0,0,0,0,0,0,0,0,0,0,1,2,2,3,0,0,0,1,0,0,0,1,0,0,0,0,0,1,1,2,0,0,0,0,0,0,0,0,0,0,0,0,0,0,0,0,0,0,0,0,1,2,0,1,0,0,0,0,0,0,0,0,0,0,0,0,0,0,0,0,0

	
```

```
		  

		NW_003726637_1, Position,0,100,200,300,400,500,600,700,800,900,1000,1100,1200,1300,1400,1500,1600,1700,1800,1900,2000,2100,2200,2300,2400,2500,2600,2700,2800,2900,3000,3100,3200,3300,3400,3500,3600,3700,3800,3900,4000,4100,4200,4300,4400,4500,4600,4700,4800,4900,5000,5100,5200,5300,5400,5500,5600,5700,5800,5900,6000,6100,6200,6300,6400,6500,6600,6700,6800,6900,7000,7100,7200,7300,7400,7500,7600,7700,7800,7900,8000,8100,8200,8300,8400,8500,8600,8700,8800,8900,9000,9100,9200,9300,9400,9500,9600,9700,9800,9900,10000,10100,10200,10300,10400,10500,10600,10700,10800,10900,11000,11100,11200,11300,11400,11500,11600,11700,11800,11900,12000,12100,12200,12300,12400,12500,12600,12700,12800,12900,13000,13100,13200,13300,13400,13500,13600,13700,13800,13900,14000,14100,14200,14300,14400,14500,14600,14700,14800,14900,15000,15100,15200,15300,15400,15500,15600,15700,15800,15900,16000,16100,16200,16300,16400,16500,16600,16700,16800,16900,17000,17100,17200,17300,17400,17500,17600,17700,17800,17900,18000,18100,18200,18300,18400,18500,18600,18700,18800,18900,19000,19100,19200,19300,19400,19500,19600,19700,19800,19900,20000,20100,20200,20300,20400,20500,20600,20700,20800,20900,21000,21100,21200,21300,21400,21500,21600,21700,21800,21900,22000,22100,22200,22300,22400,22500,22600,22700,22800,22900,23000,23100,23200,23300,23400,23500,23600,23700,23800,23900,24000,24100,24200,24300,24400,24500,24600,24700,24800
NW_003726637_1,Count,0,1,3,0,0,0,0,3,1,0,4,0,1,0,0,0,0,0,0,0,0,0,0,2,2,1,1,2,2,2,0,2,0,0,0,2,4,0,0,0,0,0,0,0,0,0,0,0,0,0,0,0,0,0,0,0,0,0,0,0,0,0,0,0,0,0,0,0,0,0,0,0,0,0,0,0,0,0,0,0,0,0,0,0,0,0,0,0,0,0,0,0,0,0,0,0,0,0,0,0,0,0,0,0,0,0,0,0,0,0,0,0,0,0,0,0,0,0,0,0,0,0,0,0,0,0,0,0,0,0,0,0,0,0,0,0,0,0,0,0,0,0,0,0,0,0,0,0,0,0,0,0,0,0,0,0,0,0,0,0,0,0,0,0,0,0,0,0,0,0,0,0,0,0,0,0,0,0,0,0,0,0,0,0,0,0,0,0,0,0,0,0,0,0,0,0,0,0,0,0,0,0,0,0,0,0,0,0,0,0,0,0,0,0,0,0,0,0,0,0,0,0,0,0,0,0,0,0,2,2,2,1,2,2,0,0,1,1,2,0,0,0,0,0,2,3,3,1,0

	
```

```
		  

		NW_003726638_1, Position,0,1000,2000,3000,4000,5000,6000,7000,8000,9000,10000,11000,12000,13000,14000,15000,16000,17000,18000,19000,20000,21000,22000,23000,24000,25000,26000,27000,28000,29000,30000,31000,32000,33000
NW_003726638_1,Count,0,0,0,0,0,0,0,0,0,0,0,0,0,0,0,0,0,0,1,2,0,0,0,0,0,0,0,0,0,0,0,0,0,0

	
```

```
		  

		NW_003726643_1, Position,0,1000,2000,3000,4000,5000,6000,7000,8000,9000,10000,11000,12000,13000,14000,15000,16000,17000,18000,19000,20000,21000,22000,23000,24000,25000,26000,27000,28000,29000,30000,31000,32000,33000
NW_003726643_1,Count,0,0,0,0,0,0,0,0,0,0,0,0,0,0,0,0,0,0,2,0,0,0,0,0,0,0,0,0,0,0,0,0,0,0

	
```

```
		  

		NW_003726644_1, Position,0,100,200,300,400,500,600,700,800,900,1000,1100,1200,1300,1400,1500,1600,1700,1800,1900,2000,2100,2200,2300,2400,2500,2600,2700,2800,2900,3000,3100,3200,3300,3400,3500,3600,3700,3800,3900,4000,4100,4200,4300,4400,4500,4600,4700,4800,4900,5000,5100,5200,5300,5400,5500,5600,5700,5800,5900,6000,6100,6200,6300,6400,6500,6600,6700,6800,6900,7000,7100,7200,7300,7400,7500,7600,7700,7800,7900,8000,8100,8200,8300,8400,8500,8600,8700,8800,8900,9000,9100,9200,9300,9400,9500,9600,9700,9800,9900,10000,10100,10200,10300,10400,10500,10600,10700,10800,10900,11000,11100,11200,11300,11400,11500,11600,11700,11800,11900,12000,12100,12200,12300,12400,12500,12600,12700,12800,12900,13000,13100,13200,13300,13400,13500,13600,13700,13800,13900,14000,14100,14200,14300,14400,14500,14600,14700,14800,14900,15000,15100,15200,15300,15400,15500,15600,15700,15800,15900,16000,16100,16200,16300,16400,16500,16600,16700,16800,16900,17000,17100,17200,17300,17400,17500,17600,17700,17800,17900,18000,18100,18200,18300,18400,18500,18600,18700,18800,18900,19000,19100,19200,19300,19400,19500,19600,19700,19800,19900,20000,20100,20200,20300,20400,20500,20600,20700,20800,20900,21000,21100,21200,21300,21400,21500,21600,21700,21800,21900,22000,22100,22200,22300,22400,22500,22600,22700,22800,22900,23000,23100,23200,23300,23400,23500,23600,23700,23800,23900,24000,24100,24200,24300,24400,24500,24600,24700,24800,24900,25000,25100,25200,25300,25400,25500,25600,25700,25800,25900,26000,26100,26200,26300,26400,26500,26600,26700,26800,26900,27000,27100,27200,27300,27400,27500,27600,27700,27800,27900,28000,28100,28200,28300,28400,28500,28600,28700,28800,28900,29000,29100,29200,29300,29400,29500,29600,29700,29800
NW_003726644_1,Count,0,0,0,0,0,0,0,0,0,0,0,0,0,0,0,0,0,0,0,0,0,0,0,0,0,0,0,0,0,0,0,0,0,0,0,0,0,0,0,0,0,0,0,0,0,0,0,0,0,0,0,0,0,0,0,0,0,0,0,0,0,0,0,0,0,0,0,0,0,0,0,0,0,0,0,0,0,0,0,0,0,0,0,0,0,0,0,0,0,0,0,0,0,0,0,0,0,0,0,0,0,0,0,0,0,0,0,0,0,0,0,0,0,0,0,0,0,0,0,0,0,0,0,0,0,0,0,0,0,0,0,0,0,0,0,0,0,0,0,0,0,0,0,0,0,0,0,0,0,0,0,0,0,0,0,0,1,0,0,0,0,0,0,0,0,0,0,0,0,0,0,0,0,0,0,0,0,0,0,0,0,0,0,0,0,0,0,0,0,0,0,0,0,0,0,0,0,0,0,0,0,0,0,0,0,0,0,0,0,0,0,0,0,0,0,0,0,0,0,0,0,0,0,0,0,0,0,0,0,0,0,0,0,0,0,0,0,0,0,0,0,0,0,0,0,0,0,0,0,0,0,0,0,0,0,0,0,0,0,0,0,0,0,0,0,0,0,0,0,0,0,0,0,0,0,0,0,0,0,0,0,0,0,0,0,0,0,0,0,0,0,0,0,0,0,0,0,0,0

	
```

```
		  

		NW_003726646_1, Position,0,100,200,300,400,500,600,700,800,900,1000,1100,1200,1300,1400,1500,1600,1700,1800,1900,2000,2100,2200,2300,2400,2500,2600,2700,2800,2900,3000,3100,3200,3300,3400,3500,3600,3700,3800,3900,4000,4100,4200,4300,4400,4500,4600,4700,4800,4900,5000,5100,5200,5300,5400,5500,5600,5700,5800,5900,6000,6100,6200,6300,6400,6500,6600,6700,6800,6900,7000,7100,7200,7300,7400,7500,7600,7700,7800,7900,8000,8100,8200,8300,8400,8500,8600,8700,8800,8900,9000,9100,9200,9300,9400,9500,9600,9700,9800,9900,10000,10100,10200,10300,10400,10500,10600,10700,10800,10900,11000,11100,11200,11300,11400,11500,11600,11700,11800,11900,12000,12100,12200,12300,12400,12500,12600,12700,12800,12900,13000,13100,13200,13300,13400,13500,13600,13700,13800,13900,14000,14100,14200,14300,14400,14500,14600,14700,14800,14900,15000,15100,15200,15300,15400,15500,15600,15700,15800,15900,16000,16100,16200,16300,16400,16500,16600,16700,16800,16900,17000,17100,17200,17300,17400,17500,17600,17700,17800,17900,18000,18100,18200,18300,18400,18500,18600,18700,18800,18900,19000,19100,19200,19300,19400,19500,19600,19700,19800,19900,20000,20100,20200,20300,20400,20500,20600,20700,20800,20900,21000,21100,21200,21300,21400,21500,21600,21700,21800,21900,22000,22100,22200,22300,22400,22500,22600,22700,22800,22900,23000,23100,23200,23300,23400,23500,23600,23700,23800,23900,24000,24100
NW_003726646_1,Count,0,0,0,0,0,0,2,0,0,2,1,2,2,0,1,0,0,0,0,0,0,0,0,0,0,0,0,0,0,0,0,0,0,0,0,0,0,0,0,0,0,0,0,0,0,0,0,1,3,0,0,1,0,0,0,0,0,0,0,0,0,0,0,0,0,0,0,0,0,0,0,0,0,0,0,0,0,0,0,0,0,0,0,0,0,0,0,0,0,0,0,0,0,0,0,0,0,0,0,0,0,0,1,1,0,0,0,0,0,0,0,0,0,0,0,0,0,0,0,0,0,0,0,0,0,0,0,0,0,0,0,0,0,0,0,0,0,0,0,0,1,1,0,0,0,0,0,0,0,0,0,0,0,0,0,0,0,0,0,0,0,1,2,0,2,0,0,0,0,0,0,1,1,2,2,0,1,0,0,0,0,0,0,0,0,0,0,0,0,0,0,0,0,0,0,0,0,0,0,0,0,0,0,0,0,0,0,0,0,0,0,0,0,0,0,0,0,0,0,0,0,0,0,0,0,0,0,0,0,0,0,0,0,0,0,0,0,0,0,0,0,0

	
```

```
		  

		NW_003726647_1, Position,0,1000,2000,3000,4000,5000,6000,7000,8000,9000,10000,11000,12000,13000,14000,15000,16000,17000,18000,19000,20000,21000,22000,23000,24000,25000,26000,27000,28000,29000,30000,31000,32000,33000,34000,35000,36000,37000,38000,39000,40000
NW_003726647_1,Count,0,0,0,0,0,0,0,0,0,0,0,0,2,5,0,0,0,0,2,8,0,0,0,4,0,0,0,5,0,0,12,1,0,0,0,0,0,2,10,4,0

	
```

```
		  

		NW_003726649_1, Position,0,1000,2000,3000,4000,5000,6000,7000,8000,9000,10000,11000,12000,13000,14000,15000,16000,17000,18000,19000,20000,21000,22000,23000,24000,25000,26000,27000,28000,29000,30000,31000,32000,33000,34000,35000,36000
NW_003726649_1,Count,0,0,0,0,0,0,0,0,0,0,0,0,0,0,0,0,0,0,0,0,0,0,0,0,0,0,4,0,0,0,0,0,0,0,0,0,0

	
```

```
		  

		NW_003726650_1, Position,0,100,200,300,400,500,600,700,800,900,1000,1100,1200,1300,1400,1500,1600,1700,1800,1900,2000,2100,2200,2300,2400,2500,2600,2700,2800,2900,3000,3100,3200,3300,3400,3500,3600,3700,3800,3900,4000,4100,4200,4300,4400,4500,4600,4700,4800,4900,5000,5100,5200,5300,5400,5500,5600,5700,5800,5900,6000,6100,6200,6300,6400,6500,6600,6700,6800,6900,7000,7100,7200,7300,7400,7500,7600,7700,7800,7900,8000,8100,8200,8300,8400,8500,8600,8700,8800,8900,9000,9100,9200,9300,9400,9500,9600,9700,9800,9900,10000,10100,10200,10300,10400,10500,10600,10700,10800,10900,11000,11100,11200,11300,11400,11500,11600,11700,11800,11900,12000,12100,12200,12300,12400,12500,12600,12700,12800,12900,13000,13100,13200,13300,13400,13500,13600,13700,13800,13900,14000,14100,14200,14300,14400,14500,14600,14700,14800,14900,15000,15100,15200,15300,15400,15500,15600,15700,15800,15900,16000,16100,16200,16300,16400,16500,16600,16700,16800,16900,17000,17100,17200,17300,17400,17500,17600,17700,17800,17900,18000,18100,18200,18300,18400,18500,18600,18700,18800,18900,19000,19100,19200,19300,19400,19500,19600,19700,19800,19900,20000,20100,20200,20300,20400,20500,20600,20700,20800,20900,21000,21100,21200,21300,21400,21500,21600,21700,21800,21900,22000,22100,22200,22300,22400,22500,22600,22700,22800,22900,23000,23100,23200,23300,23400,23500,23600,23700,23800
NW_003726650_1,Count,0,0,0,0,0,0,0,0,0,0,0,0,0,0,0,0,0,0,0,0,0,0,0,0,0,0,0,0,0,0,0,0,0,0,0,0,0,0,0,0,0,0,0,0,0,0,0,0,0,0,0,0,0,0,0,0,0,0,0,0,0,0,0,0,0,0,0,0,0,0,0,0,0,1,1,0,0,0,0,0,1,0,1,0,0,0,0,0,0,0,0,0,0,0,0,0,0,0,0,0,0,0,0,0,0,0,0,0,0,0,0,0,0,0,0,0,0,0,0,0,0,0,0,0,0,0,0,0,0,0,0,0,0,0,0,0,0,0,0,0,0,0,0,0,0,0,0,0,0,0,0,0,0,0,0,0,0,0,0,0,0,0,0,0,0,0,0,0,0,0,0,0,0,0,0,0,0,0,0,0,0,0,0,0,0,0,0,0,0,0,0,0,0,0,0,0,0,0,0,0,0,0,0,0,0,0,0,0,0,0,0,0,0,0,0,3,2,4,0,0,0,0,0,0,0,0,0,0,0,0,0,0,0,0,0,0,0,0,0

	
```

```
		  

		NW_003726651_1, Position,0,100,200,300,400,500,600,700,800,900,1000,1100,1200,1300,1400,1500,1600,1700,1800,1900,2000,2100,2200,2300,2400,2500,2600,2700,2800,2900,3000,3100,3200,3300,3400,3500,3600,3700,3800,3900,4000,4100,4200,4300,4400,4500,4600,4700,4800,4900,5000,5100,5200,5300,5400,5500,5600,5700,5800,5900,6000,6100,6200,6300,6400,6500,6600,6700,6800,6900,7000,7100,7200,7300,7400,7500,7600,7700,7800,7900,8000,8100,8200,8300,8400,8500,8600,8700,8800,8900,9000,9100,9200,9300,9400,9500,9600,9700,9800,9900,10000,10100,10200,10300,10400,10500,10600,10700,10800,10900,11000,11100,11200,11300,11400,11500,11600,11700,11800,11900,12000,12100,12200,12300,12400,12500,12600,12700,12800,12900,13000,13100,13200,13300,13400,13500,13600,13700,13800,13900,14000,14100,14200,14300,14400,14500,14600,14700,14800,14900,15000,15100,15200,15300,15400,15500,15600,15700,15800,15900,16000,16100,16200,16300,16400,16500,16600,16700,16800,16900,17000,17100,17200,17300,17400,17500,17600,17700,17800,17900,18000,18100,18200,18300,18400,18500,18600,18700,18800,18900,19000,19100,19200,19300,19400,19500,19600,19700,19800,19900,20000,20100,20200,20300,20400,20500,20600,20700,20800,20900,21000,21100,21200,21300,21400,21500,21600,21700,21800,21900,22000,22100,22200,22300,22400,22500,22600,22700,22800,22900,23000,23100,23200,23300,23400,23500,23600,23700,23800,23900,24000,24100,24200,24300,24400,24500,24600,24700,24800,24900,25000,25100,25200,25300,25400,25500,25600,25700,25800,25900,26000,26100,26200,26300,26400,26500,26600,26700,26800,26900,27000,27100,27200,27300,27400,27500,27600,27700,27800,27900,28000,28100,28200,28300,28400,28500,28600,28700,28800,28900,29000,29100,29200,29300,29400
NW_003726651_1,Count,0,0,0,0,0,0,0,0,0,0,0,0,0,0,0,0,0,0,0,0,0,0,0,0,0,0,0,0,0,0,0,0,0,0,0,0,0,0,0,0,0,0,0,0,0,0,0,0,0,0,0,0,0,0,0,0,0,0,0,0,0,0,0,0,0,0,0,0,0,0,0,0,0,0,0,0,0,0,0,0,0,0,0,0,0,0,0,0,0,0,0,0,0,0,0,0,0,1,0,0,0,0,0,0,0,0,0,0,0,0,0,0,0,0,0,0,0,0,0,0,0,0,0,0,0,0,0,0,0,0,0,0,0,0,0,0,0,0,0,0,0,0,0,0,0,0,0,0,0,0,0,0,0,0,0,0,0,0,0,0,0,0,0,0,0,0,0,0,0,0,0,0,0,0,0,0,0,0,0,0,0,0,0,0,0,0,0,0,0,0,0,0,0,0,0,0,0,0,0,0,0,0,0,0,0,0,0,0,0,0,0,0,0,0,0,0,0,0,0,0,0,0,0,0,0,0,0,0,0,0,0,0,0,0,0,0,0,0,0,0,0,0,0,0,0,0,0,0,0,0,0,0,0,0,0,0,0,0,0,0,0,0,0,0,0,0,0,0,0,0,0,0,0,0,0,0,0,0,0,0,0,0,0,0,0,0,0,0,0,0,0,0,0,0,0

	
```

```
		  

		NW_003726654_1, Position,0,1000,2000,3000,4000,5000,6000,7000,8000,9000,10000,11000,12000,13000,14000,15000,16000,17000,18000,19000,20000,21000,22000,23000,24000,25000,26000,27000,28000,29000,30000,31000,32000,33000,34000,35000,36000
NW_003726654_1,Count,0,0,1,6,0,0,0,0,0,0,0,0,0,0,0,0,0,0,0,0,0,0,0,0,0,0,0,0,0,0,0,0,0,0,0,0,0

	
```

```
		  

		NW_003726663_1, Position,0,100,200,300,400,500,600,700,800,900,1000,1100,1200,1300,1400,1500,1600,1700,1800,1900,2000,2100,2200,2300,2400,2500,2600,2700,2800,2900,3000,3100,3200,3300,3400,3500,3600,3700,3800,3900,4000,4100,4200,4300,4400,4500,4600,4700,4800,4900,5000,5100,5200,5300,5400,5500,5600,5700,5800,5900,6000,6100,6200,6300,6400,6500,6600,6700,6800,6900,7000,7100,7200,7300,7400,7500,7600,7700,7800,7900,8000,8100,8200,8300,8400,8500,8600,8700,8800,8900,9000,9100,9200,9300,9400,9500,9600,9700,9800,9900,10000,10100,10200,10300,10400,10500,10600,10700,10800,10900,11000,11100,11200,11300,11400,11500,11600,11700,11800,11900,12000,12100,12200,12300,12400,12500,12600,12700,12800,12900,13000,13100,13200,13300,13400,13500,13600,13700,13800,13900,14000,14100,14200,14300,14400,14500,14600,14700,14800,14900,15000,15100,15200,15300,15400,15500,15600,15700,15800,15900,16000,16100,16200,16300,16400,16500,16600,16700,16800,16900,17000,17100,17200,17300,17400,17500,17600,17700,17800,17900,18000,18100,18200,18300,18400,18500,18600,18700,18800,18900,19000,19100,19200,19300,19400,19500,19600,19700,19800,19900,20000,20100,20200,20300,20400,20500,20600,20700,20800,20900,21000,21100,21200,21300,21400,21500,21600,21700,21800,21900,22000,22100,22200,22300,22400,22500,22600,22700,22800,22900,23000,23100,23200,23300,23400,23500
NW_003726663_1,Count,0,0,0,0,0,0,0,0,0,0,0,0,0,0,0,0,0,0,0,0,0,0,0,0,0,0,0,0,0,0,0,0,0,0,0,0,0,0,0,0,0,0,0,0,0,0,0,0,0,0,0,0,0,0,0,0,0,0,0,0,0,0,0,0,0,0,0,0,0,0,0,0,0,0,0,0,0,0,0,0,0,0,0,0,0,0,0,0,0,0,0,0,2,2,0,0,0,0,0,0,0,0,0,0,0,0,0,0,0,0,0,0,0,0,0,0,0,0,0,0,0,4,0,0,0,2,2,1,0,0,0,0,0,0,2,1,1,0,0,0,0,1,2,1,2,2,2,0,0,0,0,0,0,0,0,0,0,0,0,0,0,2,0,2,0,0,0,0,0,0,0,0,0,0,0,0,0,0,0,0,1,0,0,0,0,1,3,0,0,0,0,3,0,0,0,4,0,1,1,2,0,1,0,0,0,0,1,0,2,0,0,0,0,0,0,0,0,1,1,3,0,0,0,1,0,2,2,1,1,1,2,1,3,1,1,0

	
```

```
		  

		NW_003726664_1, Position,0,100,200,300,400,500,600,700,800,900,1000,1100,1200,1300,1400,1500,1600,1700,1800,1900,2000,2100,2200,2300,2400,2500,2600,2700,2800,2900,3000,3100,3200,3300,3400,3500,3600,3700,3800,3900,4000,4100,4200,4300,4400,4500,4600,4700,4800,4900,5000,5100,5200,5300,5400,5500,5600,5700,5800,5900,6000,6100,6200,6300,6400,6500,6600,6700,6800,6900,7000,7100,7200,7300,7400,7500,7600,7700,7800,7900,8000,8100,8200,8300,8400,8500,8600,8700,8800,8900,9000,9100,9200,9300,9400,9500,9600,9700,9800,9900,10000,10100,10200,10300,10400,10500,10600,10700,10800,10900,11000,11100,11200,11300,11400,11500,11600,11700,11800,11900,12000,12100,12200,12300,12400,12500,12600,12700,12800,12900,13000,13100,13200,13300,13400,13500,13600,13700,13800,13900,14000,14100,14200,14300,14400,14500,14600,14700,14800,14900,15000,15100,15200,15300,15400,15500,15600,15700,15800,15900,16000,16100,16200,16300,16400,16500,16600,16700,16800,16900,17000,17100,17200,17300,17400,17500,17600,17700,17800,17900,18000,18100,18200,18300,18400,18500,18600,18700,18800,18900,19000,19100,19200,19300,19400,19500,19600,19700,19800,19900,20000,20100,20200,20300,20400,20500,20600,20700,20800,20900,21000,21100,21200,21300,21400,21500,21600,21700,21800,21900,22000,22100,22200,22300,22400,22500,22600,22700,22800,22900,23000,23100,23200,23300,23400,23500,23600,23700,23800,23900,24000,24100,24200,24300,24400,24500,24600,24700,24800,24900,25000,25100,25200,25300,25400,25500,25600,25700,25800,25900,26000,26100,26200,26300,26400,26500,26600,26700
NW_003726664_1,Count,0,0,0,0,0,0,0,0,0,0,0,0,0,0,0,0,0,0,0,0,0,0,0,0,0,0,0,0,0,0,0,0,0,0,0,0,0,0,0,0,0,0,0,3,1,0,0,0,0,0,0,0,0,0,0,0,0,0,0,0,0,0,0,0,0,0,1,0,3,0,0,0,0,1,0,2,0,0,0,0,3,2,0,0,2,0,0,0,0,0,0,0,0,0,0,0,0,0,0,0,0,0,0,0,0,2,0,1,1,0,2,1,1,2,2,0,0,3,0,0,2,0,0,0,0,0,0,0,2,0,2,1,0,0,0,3,0,0,3,0,0,0,1,1,2,1,0,0,2,3,1,2,2,1,1,3,2,1,1,1,0,0,0,0,2,0,0,0,0,0,0,0,2,1,0,0,2,0,2,0,0,0,0,0,0,0,1,0,0,1,0,0,0,0,0,0,0,1,0,0,0,1,1,0,1,0,0,0,0,0,1,1,1,0,0,1,0,0,0,0,0,0,0,0,0,0,0,0,0,0,0,0,0,0,0,0,0,1,2,1,1,2,0,1,1,0,0,0,0,0,0,0,0,0,0,0,0,0,0,0,0,0,0,0,0,0,0,0

	
```

```
		  

		NW_003726665_1, Position,0,100,200,300,400,500,600,700,800,900,1000,1100,1200,1300,1400,1500,1600,1700,1800,1900,2000,2100,2200,2300,2400,2500,2600,2700,2800,2900,3000,3100,3200,3300,3400,3500,3600,3700,3800,3900,4000,4100,4200,4300,4400,4500,4600,4700,4800,4900,5000,5100,5200,5300,5400,5500,5600,5700,5800,5900,6000,6100,6200,6300,6400,6500,6600,6700,6800,6900,7000,7100,7200,7300,7400,7500,7600,7700,7800,7900,8000,8100,8200,8300,8400,8500,8600,8700,8800,8900,9000,9100,9200,9300,9400,9500,9600,9700,9800,9900,10000,10100,10200,10300,10400,10500,10600,10700,10800,10900,11000,11100,11200,11300,11400,11500,11600,11700,11800,11900,12000,12100,12200,12300,12400,12500,12600,12700,12800,12900,13000,13100,13200,13300,13400,13500,13600,13700,13800,13900,14000,14100,14200,14300,14400,14500,14600,14700,14800,14900,15000,15100,15200,15300,15400,15500,15600,15700,15800,15900,16000,16100,16200,16300,16400,16500,16600,16700,16800,16900,17000,17100,17200,17300,17400,17500,17600,17700,17800,17900,18000,18100,18200,18300,18400,18500,18600,18700,18800,18900,19000,19100,19200,19300,19400,19500,19600,19700,19800,19900,20000,20100,20200,20300,20400,20500,20600,20700,20800,20900,21000,21100,21200,21300,21400,21500,21600,21700,21800,21900,22000,22100,22200,22300,22400,22500,22600,22700,22800,22900,23000,23100,23200,23300,23400
NW_003726665_1,Count,0,0,0,0,0,0,0,0,0,3,0,0,0,0,0,0,0,0,0,0,0,0,0,0,0,0,0,0,0,0,0,0,0,0,0,0,0,0,0,0,0,0,0,0,0,0,0,0,0,0,0,0,0,0,0,1,2,1,0,0,0,0,0,0,0,1,0,0,0,0,0,0,0,0,0,0,0,0,0,0,0,0,0,0,0,0,0,0,0,1,0,0,2,1,0,1,1,1,1,0,1,1,1,1,0,1,0,0,0,0,0,0,0,0,0,0,0,0,0,0,0,0,0,0,0,0,0,0,0,2,0,1,1,1,3,1,0,0,0,0,0,0,0,0,0,0,0,0,0,0,0,0,0,0,0,0,0,0,0,0,0,0,0,0,0,0,0,0,0,0,0,0,0,0,0,0,0,0,0,0,0,0,0,0,0,0,0,0,0,1,3,0,2,2,0,0,0,0,0,0,0,0,1,2,1,0,0,3,0,1,0,0,0,0,0,0,0,1,1,2,0,0,0,0,0,0,0,0,0,0,0,0,0,0,0

	
```

```
		  

		NW_003726666_1, Position,0,100,200,300,400,500,600,700,800,900,1000,1100,1200,1300,1400,1500,1600,1700,1800,1900,2000,2100,2200,2300,2400,2500,2600,2700,2800,2900,3000,3100,3200,3300,3400,3500,3600,3700,3800,3900,4000,4100,4200,4300,4400,4500,4600,4700,4800,4900,5000,5100,5200,5300,5400,5500,5600,5700,5800,5900,6000,6100,6200,6300,6400,6500,6600,6700,6800,6900,7000,7100,7200,7300,7400,7500,7600,7700,7800,7900,8000,8100,8200,8300,8400,8500,8600,8700,8800,8900,9000,9100,9200,9300,9400,9500,9600,9700,9800,9900,10000,10100,10200,10300,10400,10500,10600,10700,10800,10900,11000,11100,11200,11300,11400,11500,11600,11700,11800,11900,12000,12100,12200,12300,12400,12500,12600,12700,12800,12900,13000,13100,13200,13300,13400,13500,13600,13700,13800,13900,14000,14100,14200,14300,14400,14500,14600,14700,14800,14900,15000,15100,15200,15300,15400,15500,15600,15700,15800,15900,16000,16100,16200,16300,16400,16500,16600,16700,16800,16900,17000,17100,17200,17300,17400,17500,17600,17700,17800,17900,18000,18100,18200,18300,18400,18500,18600,18700,18800,18900,19000,19100,19200,19300,19400,19500,19600,19700,19800,19900,20000,20100,20200,20300,20400,20500,20600,20700,20800,20900,21000,21100,21200,21300,21400,21500,21600,21700,21800,21900,22000,22100,22200,22300,22400,22500,22600,22700,22800,22900,23000,23100,23200,23300,23400,23500,23600,23700,23800,23900,24000,24100,24200,24300,24400,24500,24600,24700,24800,24900
NW_003726666_1,Count,0,0,0,0,0,2,0,1,2,2,0,0,0,1,1,2,1,0,1,0,2,2,0,1,1,0,0,0,0,0,0,0,0,0,0,0,0,1,2,0,0,0,0,0,0,0,3,1,0,1,0,2,3,2,0,0,0,0,0,0,0,0,0,0,0,0,0,0,2,2,1,0,0,0,0,0,0,1,0,0,1,0,1,1,3,1,0,0,0,0,0,0,0,0,0,0,0,0,0,0,0,0,0,0,0,0,0,0,0,0,0,0,0,0,1,0,0,0,0,0,0,0,0,0,0,0,0,0,0,0,1,2,1,0,0,1,1,0,0,0,0,0,0,0,0,0,0,2,0,3,0,0,0,0,0,0,0,3,0,0,0,0,0,0,0,0,0,1,2,2,3,0,0,0,0,0,0,0,0,0,0,1,2,1,2,3,0,0,0,2,0,2,3,0,0,0,0,0,0,0,1,2,0,0,1,1,1,0,1,2,0,3,0,0,0,1,2,0,4,1,2,3,1,0,0,2,1,2,2,2,0,0,0,0,0,0,0,0,0,0,0,0,0,0,0,0,0,0,0,0

	
```

```
		  

		NW_003726668_1, Position,0,100,200,300,400,500,600,700,800,900,1000,1100,1200,1300,1400,1500,1600,1700,1800,1900,2000,2100,2200,2300,2400,2500,2600,2700,2800,2900,3000,3100,3200,3300,3400,3500,3600,3700,3800,3900,4000,4100,4200,4300,4400,4500,4600,4700,4800,4900,5000,5100,5200,5300,5400,5500,5600,5700,5800,5900,6000,6100,6200,6300,6400,6500,6600,6700,6800,6900,7000,7100,7200,7300,7400,7500,7600,7700,7800,7900,8000,8100,8200,8300,8400,8500,8600,8700,8800,8900,9000,9100,9200,9300,9400,9500,9600,9700,9800,9900,10000,10100,10200,10300,10400,10500,10600,10700,10800,10900,11000,11100,11200,11300,11400,11500,11600,11700,11800,11900,12000,12100,12200,12300,12400,12500,12600,12700,12800,12900,13000,13100,13200,13300,13400,13500,13600,13700,13800,13900,14000,14100,14200,14300,14400,14500,14600,14700,14800,14900,15000,15100,15200,15300,15400,15500,15600,15700,15800,15900,16000,16100,16200,16300,16400,16500,16600,16700,16800,16900,17000,17100,17200,17300,17400,17500,17600,17700,17800,17900,18000,18100,18200,18300,18400,18500,18600,18700,18800,18900,19000,19100,19200,19300,19400,19500,19600,19700,19800,19900,20000,20100,20200,20300,20400,20500,20600,20700,20800,20900,21000,21100,21200,21300,21400,21500,21600,21700,21800,21900,22000,22100,22200,22300,22400,22500,22600,22700,22800,22900,23000,23100,23200,23300
NW_003726668_1,Count,0,0,0,0,0,0,0,0,0,0,0,0,0,0,0,0,0,0,0,0,0,0,0,0,0,0,0,0,0,0,0,0,0,0,0,0,0,0,0,0,0,0,0,0,0,0,0,0,0,0,0,0,0,0,0,0,0,0,0,0,0,0,0,0,0,0,0,0,0,0,0,0,0,0,0,0,3,0,0,0,0,0,0,0,0,0,0,0,0,0,0,0,0,0,0,5,1,0,0,0,0,0,0,0,0,0,0,0,0,0,0,0,0,0,0,0,0,0,0,0,0,0,0,0,0,0,0,0,0,0,0,0,0,0,0,0,0,0,0,0,0,0,0,0,0,0,0,0,0,0,0,0,0,0,0,0,0,0,0,0,0,0,0,0,0,0,0,0,0,0,0,0,0,0,0,0,0,0,0,0,0,0,0,0,0,0,0,0,0,0,0,0,0,0,0,0,0,0,0,0,0,0,0,0,0,0,0,0,0,0,0,0,0,0,0,0,0,0,0,0,0,0,0,0,0,0,0,0,0,0,0,0,0,0

	
```

```
		  

		NW_003726674_1, Position,0,1000,2000,3000,4000,5000,6000,7000,8000,9000,10000,11000,12000,13000,14000,15000,16000,17000,18000,19000,20000,21000,22000,23000,24000,25000,26000,27000,28000,29000,30000,31000,32000,33000,34000,35000
NW_003726674_1,Count,0,0,0,0,0,0,0,0,0,0,0,0,0,3,0,0,0,0,0,0,0,0,0,0,0,0,0,0,0,0,0,0,0,0,0,0

	
```

```
		  

		NW_003726676_1, Position,0,1000,2000,3000,4000,5000,6000,7000,8000,9000,10000,11000,12000,13000,14000,15000,16000,17000,18000,19000,20000,21000,22000,23000,24000,25000,26000,27000,28000,29000,30000,31000,32000,33000,34000,35000,36000,37000
NW_003726676_1,Count,0,0,0,2,0,0,0,0,0,0,0,0,0,0,0,0,0,0,0,0,0,0,0,0,0,0,0,0,0,0,0,0,0,0,0,0,0,0

	
```

```
		  

		NW_003726677_1, Position,0,1000,2000,3000,4000,5000,6000,7000,8000,9000,10000,11000,12000,13000,14000,15000,16000,17000,18000,19000,20000,21000,22000,23000,24000,25000,26000,27000,28000,29000,30000,31000,32000,33000,34000,35000,36000
NW_003726677_1,Count,0,0,0,0,0,0,0,0,0,0,0,0,0,0,0,0,0,0,0,0,0,0,0,0,0,12,16,15,3,0,0,0,0,0,0,0,0

	
```

```
		  

		NW_003726681_1, Position,0,100,200,300,400,500,600,700,800,900,1000,1100,1200,1300,1400,1500,1600,1700,1800,1900,2000,2100,2200,2300,2400,2500,2600,2700,2800,2900,3000,3100,3200,3300,3400,3500,3600,3700,3800,3900,4000,4100,4200,4300,4400,4500,4600,4700,4800,4900,5000,5100,5200,5300,5400,5500,5600,5700,5800,5900,6000,6100,6200,6300,6400,6500,6600,6700,6800,6900,7000,7100,7200,7300,7400,7500,7600,7700,7800,7900,8000,8100,8200,8300,8400,8500,8600,8700,8800,8900,9000,9100,9200,9300,9400,9500,9600,9700,9800,9900,10000,10100,10200,10300,10400,10500,10600,10700,10800,10900,11000,11100,11200,11300,11400,11500,11600,11700,11800,11900,12000,12100,12200,12300,12400,12500,12600,12700,12800,12900,13000,13100,13200,13300,13400,13500,13600,13700,13800,13900,14000,14100,14200,14300,14400,14500,14600,14700,14800,14900,15000,15100,15200,15300,15400,15500,15600,15700,15800,15900,16000,16100,16200,16300,16400,16500,16600,16700,16800,16900,17000,17100,17200,17300,17400,17500,17600,17700,17800,17900,18000,18100,18200,18300,18400,18500,18600,18700,18800,18900,19000,19100,19200,19300,19400,19500,19600,19700,19800,19900,20000,20100,20200,20300,20400,20500,20600,20700,20800,20900,21000,21100,21200,21300,21400,21500,21600,21700,21800,21900,22000,22100,22200,22300,22400,22500,22600,22700,22800,22900,23000,23100,23200,23300,23400,23500,23600,23700,23800,23900
NW_003726681_1,Count,0,1,1,2,0,1,1,2,1,0,0,0,2,1,2,1,1,1,0,1,2,2,2,0,2,0,0,2,0,1,2,2,1,0,0,0,0,3,0,1,0,3,1,1,0,1,2,1,0,2,1,2,0,0,1,0,0,0,2,1,1,2,1,1,0,0,0,1,3,1,3,2,1,0,0,4,0,1,0,2,2,1,1,0,1,1,0,0,0,2,0,1,2,0,1,1,1,0,0,0,1,1,0,1,0,1,0,2,0,1,1,0,0,0,0,1,0,0,0,0,0,0,0,0,0,0,0,0,1,2,0,3,0,0,1,0,0,1,1,2,0,0,0,2,0,0,0,0,0,0,2,0,0,0,1,3,2,0,0,0,0,0,0,1,0,0,1,0,1,1,0,0,0,2,2,0,0,1,0,0,0,0,0,0,0,0,0,0,1,0,1,0,1,0,1,0,0,0,1,0,0,1,2,0,1,0,0,0,0,0,0,0,1,0,0,0,0,0,0,1,0,1,1,0,0,1,0,0,0,0,0,0,1,2,1,1,2,1,0,0

	
```

```
		  

		NW_003726682_1, Position,0,100,200,300,400,500,600,700,800,900,1000,1100,1200,1300,1400,1500,1600,1700,1800,1900,2000,2100,2200,2300,2400,2500,2600,2700,2800,2900,3000,3100,3200,3300,3400,3500,3600,3700,3800,3900,4000,4100,4200,4300,4400,4500,4600,4700,4800,4900,5000,5100,5200,5300,5400,5500,5600,5700,5800,5900,6000,6100,6200,6300,6400,6500,6600,6700,6800,6900,7000,7100,7200,7300,7400,7500,7600,7700,7800,7900,8000,8100,8200,8300,8400,8500,8600,8700,8800,8900,9000,9100,9200,9300,9400,9500,9600,9700,9800,9900,10000,10100,10200,10300,10400,10500,10600,10700,10800,10900,11000,11100,11200,11300,11400,11500,11600,11700,11800,11900,12000,12100,12200,12300,12400,12500,12600,12700,12800,12900,13000,13100,13200,13300,13400,13500,13600,13700,13800,13900,14000,14100,14200,14300,14400,14500,14600,14700,14800,14900,15000,15100,15200,15300,15400,15500,15600,15700,15800,15900,16000,16100,16200,16300,16400,16500,16600,16700,16800,16900,17000,17100,17200,17300,17400,17500,17600,17700,17800,17900,18000,18100,18200,18300,18400,18500,18600,18700,18800,18900,19000,19100,19200,19300,19400,19500,19600,19700,19800,19900,20000,20100,20200,20300,20400,20500,20600,20700,20800,20900,21000,21100,21200,21300,21400,21500,21600,21700,21800,21900,22000,22100,22200,22300,22400,22500,22600,22700,22800,22900,23000
NW_003726682_1,Count,0,1,0,1,0,0,0,0,0,0,0,0,0,0,0,1,1,0,0,1,0,2,1,1,0,0,3,0,0,0,0,0,1,0,1,1,0,0,0,0,2,0,0,0,1,0,2,0,2,1,0,0,0,1,2,1,0,3,3,0,0,1,2,3,0,1,0,1,0,1,1,0,0,0,3,1,0,0,0,0,0,0,0,0,0,0,0,0,0,0,0,0,0,0,0,0,0,0,0,0,0,0,0,0,0,0,0,0,0,0,0,0,0,0,0,0,0,0,0,0,0,0,0,0,0,0,0,0,0,0,0,0,0,0,0,0,0,0,0,0,0,0,0,0,0,0,0,0,0,1,1,0,0,0,1,0,3,0,0,0,0,0,0,1,1,2,1,1,0,1,3,0,0,1,0,1,3,0,1,3,0,0,0,2,0,1,2,0,0,0,0,0,0,0,0,0,0,0,0,0,0,0,0,0,0,0,0,0,0,0,0,0,0,0,0,0,0,0,0,0,0,0,0,0,0,0,0,0,0,0,0

	
```

```
		  

		NW_003726687_1, Position,0,1000,2000,3000,4000,5000,6000,7000,8000,9000,10000,11000,12000,13000,14000,15000,16000,17000,18000,19000,20000,21000,22000,23000,24000,25000,26000,27000,28000,29000,30000,31000,32000,33000,34000,35000,36000,37000
NW_003726687_1,Count,0,0,0,0,0,0,0,0,0,0,0,1,0,0,0,0,0,0,0,0,0,0,0,0,0,0,0,0,4,0,0,0,0,0,0,0,0,0

	
```

```
		  

		NW_003726691_1, Position,0,100,200,300,400,500,600,700,800,900,1000,1100,1200,1300,1400,1500,1600,1700,1800,1900,2000,2100,2200,2300,2400,2500,2600,2700,2800,2900,3000,3100,3200,3300,3400,3500,3600,3700,3800,3900,4000,4100,4200,4300,4400,4500,4600,4700,4800,4900,5000,5100,5200,5300,5400,5500,5600,5700,5800,5900,6000,6100,6200,6300,6400,6500,6600,6700,6800,6900,7000,7100,7200,7300,7400,7500,7600,7700,7800,7900,8000,8100,8200,8300,8400,8500,8600,8700,8800,8900,9000,9100,9200,9300,9400,9500,9600,9700,9800,9900,10000,10100,10200,10300,10400,10500,10600,10700,10800,10900,11000,11100,11200,11300,11400,11500,11600,11700,11800,11900,12000,12100,12200,12300,12400,12500,12600,12700,12800,12900,13000,13100,13200,13300,13400,13500,13600,13700,13800,13900,14000,14100,14200,14300,14400,14500,14600,14700,14800,14900,15000,15100,15200,15300,15400,15500,15600,15700,15800,15900,16000,16100,16200,16300,16400,16500,16600,16700,16800,16900,17000,17100,17200,17300,17400,17500,17600,17700,17800,17900,18000,18100,18200,18300,18400,18500,18600,18700,18800,18900,19000,19100,19200,19300,19400,19500,19600,19700,19800,19900,20000,20100,20200,20300,20400,20500,20600,20700,20800,20900,21000,21100,21200,21300,21400,21500,21600,21700,21800,21900,22000,22100,22200,22300,22400,22500,22600,22700,22800,22900
NW_003726691_1,Count,0,0,0,0,0,0,0,0,0,0,0,0,0,0,0,0,0,0,0,0,0,0,0,0,0,0,0,0,0,0,0,0,0,0,0,0,0,0,0,0,0,0,0,0,0,0,1,0,0,1,2,1,1,2,1,3,0,0,2,1,2,0,0,1,0,0,2,1,0,1,1,0,0,0,0,0,2,2,1,0,0,0,0,0,0,0,0,0,0,0,0,0,0,0,0,0,0,0,0,0,0,0,0,0,0,0,0,0,0,0,0,0,3,0,0,2,0,1,1,0,0,0,0,2,1,2,1,2,1,3,0,2,0,1,0,0,1,0,0,0,0,0,0,0,0,1,2,0,1,1,1,1,0,1,0,3,0,1,0,0,0,1,0,3,1,0,0,1,0,0,1,1,0,0,0,0,0,0,3,1,0,2,0,3,1,0,0,0,0,0,0,0,0,0,0,0,0,0,0,0,0,0,0,0,0,0,0,0,0,0,0,0,0,0,0,0,0,0,0,0,0,0,0,0,0,0,0,0,0,0

	
```

```
		  

		NW_003726700_1, Position,0,100,200,300,400,500,600,700,800,900,1000,1100,1200,1300,1400,1500,1600,1700,1800,1900,2000,2100,2200,2300,2400,2500,2600,2700,2800,2900,3000,3100,3200,3300,3400,3500,3600,3700,3800,3900,4000,4100,4200,4300,4400,4500,4600,4700,4800,4900,5000,5100,5200,5300,5400,5500,5600,5700,5800,5900,6000,6100,6200,6300,6400,6500,6600,6700,6800,6900,7000,7100,7200,7300,7400,7500,7600,7700,7800,7900,8000,8100,8200,8300,8400,8500,8600,8700,8800,8900,9000,9100,9200,9300,9400,9500,9600,9700,9800,9900,10000,10100,10200,10300,10400,10500,10600,10700,10800,10900,11000,11100,11200,11300,11400,11500,11600,11700,11800,11900,12000,12100,12200,12300,12400,12500,12600,12700,12800,12900,13000,13100,13200,13300,13400,13500,13600,13700,13800,13900,14000,14100,14200,14300,14400,14500,14600,14700,14800,14900,15000,15100,15200,15300,15400,15500,15600,15700,15800,15900,16000,16100,16200,16300,16400,16500,16600,16700,16800,16900,17000,17100,17200,17300,17400,17500,17600,17700,17800,17900,18000,18100,18200,18300,18400,18500,18600,18700,18800,18900,19000,19100,19200,19300,19400,19500,19600,19700,19800,19900,20000,20100,20200,20300,20400,20500,20600,20700,20800,20900,21000,21100,21200,21300,21400,21500,21600,21700,21800,21900,22000,22100,22200,22300,22400,22500,22600,22700,22800,22900,23000,23100,23200,23300,23400,23500,23600,23700,23800,23900
NW_003726700_1,Count,0,0,0,0,0,0,0,0,0,0,0,0,0,0,0,0,0,0,0,0,0,0,0,0,0,0,0,0,0,0,0,0,0,0,0,0,0,0,0,0,0,0,0,0,0,0,0,0,0,0,0,0,0,0,0,0,0,0,0,0,0,0,0,0,0,0,0,0,0,1,1,0,0,0,0,0,0,0,0,0,0,0,0,0,0,0,0,0,0,0,0,0,0,0,0,0,0,0,0,0,0,0,0,0,0,0,0,0,0,0,0,0,0,0,0,0,0,0,0,0,0,0,0,0,0,0,0,0,0,0,0,0,0,0,0,0,0,0,0,0,0,0,0,0,0,0,0,0,0,0,0,0,0,0,0,0,0,0,0,0,0,0,0,0,0,0,0,0,0,0,0,0,0,0,0,0,0,0,0,0,0,0,0,0,0,0,0,0,0,0,0,0,0,0,0,0,0,0,0,0,0,0,0,0,0,0,0,0,0,0,0,0,0,0,0,0,0,0,0,0,0,0,0,0,0,0,0,0,0,0,0,0,0,0,0,0,0,0,0,0

	
```

```
		  

		NW_003726702_1, Position,0,1000,2000,3000,4000,5000,6000,7000,8000,9000,10000,11000,12000,13000,14000,15000,16000,17000,18000,19000,20000,21000,22000,23000,24000,25000,26000,27000,28000,29000,30000
NW_003726702_1,Count,0,0,0,0,0,0,0,1,0,0,10,5,0,11,11,0,1,0,0,0,0,0,0,0,0,6,11,13,7,10,1

	
```

```
		  

		NW_003726706_1, Position,0,100,200,300,400,500,600,700,800,900,1000,1100,1200,1300,1400,1500,1600,1700,1800,1900,2000,2100,2200,2300,2400,2500,2600,2700,2800,2900,3000,3100,3200,3300,3400,3500,3600,3700,3800,3900,4000,4100,4200,4300,4400,4500,4600,4700,4800,4900,5000,5100,5200,5300,5400,5500,5600,5700,5800,5900,6000,6100,6200,6300,6400,6500,6600,6700,6800,6900,7000,7100,7200,7300,7400,7500,7600,7700,7800,7900,8000,8100,8200,8300,8400,8500,8600,8700,8800,8900,9000,9100,9200,9300,9400,9500,9600,9700,9800,9900,10000,10100,10200,10300,10400,10500,10600,10700,10800,10900,11000,11100,11200,11300,11400,11500,11600,11700,11800,11900,12000,12100,12200,12300,12400,12500,12600,12700,12800,12900,13000,13100,13200,13300,13400,13500,13600,13700,13800,13900,14000,14100,14200,14300,14400,14500,14600,14700,14800,14900,15000,15100,15200,15300,15400,15500,15600,15700,15800,15900,16000,16100,16200,16300,16400,16500,16600,16700,16800,16900,17000,17100,17200,17300,17400,17500,17600,17700,17800,17900,18000,18100,18200,18300,18400,18500,18600,18700,18800,18900,19000,19100,19200,19300,19400,19500,19600,19700,19800,19900,20000,20100,20200,20300,20400,20500,20600,20700,20800,20900,21000,21100,21200,21300,21400,21500,21600,21700,21800,21900,22000,22100,22200,22300
NW_003726706_1,Count,0,0,0,0,0,0,0,0,0,0,0,0,0,0,0,2,1,1,1,0,3,2,0,0,3,1,0,0,0,0,0,0,0,0,0,2,1,1,2,1,2,5,0,0,0,0,1,0,0,0,0,1,1,1,0,1,1,2,2,4,0,0,0,1,2,2,1,3,2,0,0,0,2,0,1,3,4,0,2,2,2,0,2,2,3,0,0,0,0,0,2,2,1,2,0,0,0,2,0,3,0,1,1,2,0,1,0,3,0,2,1,1,0,0,3,0,2,0,1,1,0,0,0,1,1,0,1,1,0,0,1,1,2,0,0,0,0,1,1,0,1,0,0,1,0,2,1,0,2,0,0,0,1,0,0,1,0,1,2,0,2,0,0,0,0,0,0,0,1,0,1,0,0,1,0,0,0,0,0,0,2,0,0,0,0,0,0,0,1,0,2,1,3,0,0,0,1,0,2,3,0,1,0,0,0,0,0,0,0,0,0,0,0,0,0,0,0,0,2,2,2,0,0,0

	
```

```
		  

		NW_003726710_1, Position,0,100,200,300,400,500,600,700,800,900,1000,1100,1200,1300,1400,1500,1600,1700,1800,1900,2000,2100,2200,2300,2400,2500,2600,2700,2800,2900,3000,3100,3200,3300,3400,3500,3600,3700,3800,3900,4000,4100,4200,4300,4400,4500,4600,4700,4800,4900,5000,5100,5200,5300,5400,5500,5600,5700,5800,5900,6000,6100,6200,6300,6400,6500,6600,6700,6800,6900,7000,7100,7200,7300,7400,7500,7600,7700,7800,7900,8000,8100,8200,8300,8400,8500,8600,8700,8800,8900,9000,9100,9200,9300,9400,9500,9600,9700,9800,9900,10000,10100,10200,10300,10400,10500,10600,10700,10800,10900,11000,11100,11200,11300,11400,11500,11600,11700,11800,11900,12000,12100,12200,12300,12400,12500,12600,12700,12800,12900,13000,13100,13200,13300,13400,13500,13600,13700,13800,13900,14000,14100,14200,14300,14400,14500,14600,14700,14800,14900,15000,15100,15200,15300,15400,15500,15600,15700,15800,15900,16000,16100,16200,16300,16400,16500,16600,16700,16800,16900,17000,17100,17200,17300,17400,17500,17600,17700,17800,17900,18000,18100,18200,18300,18400,18500,18600,18700,18800,18900,19000,19100,19200,19300,19400,19500,19600,19700,19800,19900,20000,20100,20200,20300,20400,20500,20600,20700,20800,20900,21000,21100,21200,21300,21400,21500,21600,21700,21800,21900,22000,22100,22200
NW_003726710_1,Count,0,0,0,0,0,0,3,1,0,0,0,0,0,1,2,0,0,0,0,0,0,0,0,0,0,0,0,0,0,0,0,0,0,0,0,0,0,0,0,0,0,0,0,0,0,0,2,0,1,1,1,1,1,1,0,0,0,0,0,0,0,0,0,0,0,0,0,0,1,0,1,0,0,0,0,0,0,0,0,0,0,0,0,0,0,0,0,0,0,0,0,0,0,0,1,1,0,0,0,0,0,0,0,0,0,0,0,0,0,0,0,0,0,0,0,0,0,0,0,0,0,0,0,0,0,0,0,0,0,0,0,0,0,0,0,0,0,0,0,0,0,0,0,0,0,0,0,0,0,0,0,0,0,0,0,0,0,0,0,0,0,0,0,0,0,0,0,0,0,0,0,0,0,0,0,0,0,0,0,0,0,0,0,0,0,0,0,0,0,0,0,0,0,0,0,0,0,0,0,0,0,0,0,0,0,0,0,0,0,0,0,0,0,0,0,0,0,0,0,0,0,0,0

	
```

```
		  

		NW_003726713_1, Position,0,100,200,300,400,500,600,700,800,900,1000,1100,1200,1300,1400,1500,1600,1700,1800,1900,2000,2100,2200,2300,2400,2500,2600,2700,2800,2900,3000,3100,3200,3300,3400,3500,3600,3700,3800,3900,4000,4100,4200,4300,4400,4500,4600,4700,4800,4900,5000,5100,5200,5300,5400,5500,5600,5700,5800,5900,6000,6100,6200,6300,6400,6500,6600,6700,6800,6900,7000,7100,7200,7300,7400,7500,7600,7700,7800,7900,8000,8100,8200,8300,8400,8500,8600,8700,8800,8900,9000,9100,9200,9300,9400,9500,9600,9700,9800,9900,10000,10100,10200,10300,10400,10500,10600,10700,10800,10900,11000,11100,11200,11300,11400,11500,11600,11700,11800,11900,12000,12100,12200,12300,12400,12500,12600,12700,12800,12900,13000,13100,13200,13300,13400,13500,13600,13700,13800,13900,14000,14100,14200,14300,14400,14500,14600,14700,14800,14900,15000,15100,15200,15300,15400,15500,15600,15700,15800,15900,16000,16100,16200,16300,16400,16500,16600,16700,16800,16900,17000,17100,17200,17300,17400,17500,17600,17700,17800,17900,18000,18100,18200,18300,18400,18500,18600,18700,18800,18900,19000,19100,19200,19300,19400,19500,19600,19700,19800,19900,20000,20100,20200,20300,20400,20500,20600,20700,20800,20900,21000,21100,21200,21300,21400,21500,21600,21700,21800,21900,22000,22100,22200,22300,22400,22500,22600,22700,22800,22900,23000,23100,23200,23300,23400,23500,23600,23700,23800,23900,24000,24100,24200,24300,24400,24500,24600,24700,24800,24900,25000,25100,25200,25300,25400,25500,25600,25700,25800,25900,26000,26100
NW_003726713_1,Count,0,0,0,0,0,0,0,0,0,0,0,0,0,0,0,0,0,0,0,0,0,0,0,0,0,0,0,0,0,0,0,0,0,0,0,0,0,0,0,0,0,0,0,0,0,0,0,0,0,0,0,0,0,0,0,0,0,0,0,0,0,0,0,0,0,0,0,0,0,0,0,0,0,0,0,0,0,0,0,0,0,0,0,0,0,0,0,0,0,0,0,0,0,0,0,0,0,0,0,0,0,0,0,0,0,0,0,0,0,0,0,0,0,0,0,0,0,0,0,0,0,0,0,0,0,0,0,0,0,0,0,0,0,0,0,0,0,0,0,0,0,0,0,0,0,0,0,0,0,0,0,0,0,0,0,0,0,0,0,0,0,0,0,1,0,0,0,0,0,1,0,0,0,0,0,0,0,0,0,0,0,0,0,0,0,0,0,0,0,0,0,0,0,0,0,1,1,0,0,0,0,0,0,0,0,0,0,0,0,0,0,0,0,1,0,0,0,0,0,0,0,1,0,1,2,0,1,3,0,0,0,0,0,2,0,1,2,2,0,0,0,2,0,0,0,0,3,1,0,1,0,1,0,1,0,1,1,3,1,3,1,0

	
```

```
		  

		NW_003726716_1, Position,0,100,200,300,400,500,600,700,800,900,1000,1100,1200,1300,1400,1500,1600,1700,1800,1900,2000,2100,2200,2300,2400,2500,2600,2700,2800,2900,3000,3100,3200,3300,3400,3500,3600,3700,3800,3900,4000,4100,4200,4300,4400,4500,4600,4700,4800,4900,5000,5100,5200,5300,5400,5500,5600,5700,5800,5900,6000,6100,6200,6300,6400,6500,6600,6700,6800,6900,7000,7100,7200,7300,7400,7500,7600,7700,7800,7900,8000,8100,8200,8300,8400,8500,8600,8700,8800,8900,9000,9100,9200,9300,9400,9500,9600,9700,9800,9900,10000,10100,10200,10300,10400,10500,10600,10700,10800,10900,11000,11100,11200,11300,11400,11500,11600,11700,11800,11900,12000,12100,12200,12300,12400,12500,12600,12700,12800,12900,13000,13100,13200,13300,13400,13500,13600,13700,13800,13900,14000,14100,14200,14300,14400,14500,14600,14700,14800,14900,15000,15100,15200,15300,15400,15500,15600,15700,15800,15900,16000,16100,16200,16300,16400,16500,16600,16700,16800,16900,17000,17100,17200,17300,17400,17500,17600,17700,17800,17900,18000,18100,18200,18300,18400,18500,18600,18700,18800,18900,19000,19100,19200,19300,19400,19500,19600,19700,19800,19900,20000,20100,20200,20300,20400,20500,20600,20700,20800,20900,21000,21100,21200,21300,21400,21500,21600,21700,21800,21900,22000
NW_003726716_1,Count,0,0,2,1,0,0,0,0,0,0,2,0,3,0,0,2,0,1,0,0,0,0,0,0,0,2,0,0,3,0,2,1,1,0,0,1,1,0,0,2,3,3,1,2,1,0,0,0,0,0,0,2,2,3,2,1,1,1,0,0,0,1,1,0,0,0,0,1,2,0,0,0,1,0,2,1,2,2,4,1,1,0,1,0,2,2,0,4,1,2,3,0,0,3,0,0,0,1,0,0,0,0,0,0,0,0,0,2,0,1,2,2,2,0,1,3,1,1,0,0,0,0,0,0,0,1,2,1,2,1,1,0,0,0,0,1,1,2,0,1,0,4,0,2,1,3,1,1,2,1,0,2,2,0,0,0,0,0,0,0,1,1,1,0,1,0,2,0,1,2,0,1,1,0,0,0,0,2,3,2,1,2,0,0,0,3,0,2,0,0,0,0,0,1,1,0,0,0,2,3,2,3,1,1,1,0,1,0,0,1,0,1,3,0,0,0,1,3,1,2,0

	
```

```
		  

		NW_003726719_1, Position,0,100,200,300,400,500,600,700,800,900,1000,1100,1200,1300,1400,1500,1600,1700,1800,1900,2000,2100,2200,2300,2400,2500,2600,2700,2800,2900,3000,3100,3200,3300,3400,3500,3600,3700,3800,3900,4000,4100,4200,4300,4400,4500,4600,4700,4800,4900,5000,5100,5200,5300,5400,5500,5600,5700,5800,5900,6000,6100,6200,6300,6400,6500,6600,6700,6800,6900,7000,7100,7200,7300,7400,7500,7600,7700,7800,7900,8000,8100,8200,8300,8400,8500,8600,8700,8800,8900,9000,9100,9200,9300,9400,9500,9600,9700,9800,9900,10000,10100,10200,10300,10400,10500,10600,10700,10800,10900,11000,11100,11200,11300,11400,11500,11600,11700,11800,11900,12000,12100,12200,12300,12400,12500,12600,12700,12800,12900,13000,13100,13200,13300,13400,13500,13600,13700,13800,13900,14000,14100,14200,14300,14400,14500,14600,14700,14800,14900,15000,15100,15200,15300,15400,15500,15600,15700,15800,15900,16000,16100,16200,16300,16400,16500,16600,16700,16800,16900,17000,17100,17200,17300,17400,17500,17600,17700,17800,17900,18000,18100,18200,18300,18400,18500,18600,18700,18800,18900,19000,19100,19200,19300,19400,19500,19600,19700,19800,19900,20000,20100,20200,20300,20400,20500,20600,20700,20800,20900,21000,21100,21200,21300,21400,21500,21600,21700,21800,21900,22000
NW_003726719_1,Count,0,0,0,0,0,0,0,0,0,0,0,0,0,0,0,0,0,0,0,0,0,0,0,0,0,0,0,0,0,0,0,0,0,0,0,0,0,0,0,0,0,0,0,0,0,0,0,0,0,0,0,0,0,0,0,0,0,0,0,0,0,0,0,0,0,0,0,0,0,0,0,0,0,0,0,0,0,0,0,0,0,0,0,0,0,1,0,0,0,0,0,1,3,2,2,3,2,1,2,0,0,0,0,0,0,1,0,0,0,0,0,0,0,0,0,0,0,0,0,0,0,0,0,0,0,0,0,0,0,0,0,0,0,0,0,0,0,0,0,0,0,0,0,0,0,0,0,0,0,0,0,0,0,0,0,0,0,0,0,0,0,0,0,0,0,0,0,0,0,0,0,0,0,0,0,0,0,0,0,0,0,0,0,0,0,0,0,0,0,0,0,0,0,0,0,0,0,0,0,0,0,0,0,0,0,0,0,0,0,0,0,0,0,0,0,0,0,0,0,0,0

	
```

```
		  

		NW_003726722_1, Position,0,100,200,300,400,500,600,700,800,900,1000,1100,1200,1300,1400,1500,1600,1700,1800,1900,2000,2100,2200,2300,2400,2500,2600,2700,2800,2900,3000,3100,3200,3300,3400,3500,3600,3700,3800,3900,4000,4100,4200,4300,4400,4500,4600,4700,4800,4900,5000,5100,5200,5300,5400,5500,5600,5700,5800,5900,6000,6100,6200,6300,6400,6500,6600,6700,6800,6900,7000,7100,7200,7300,7400,7500,7600,7700,7800,7900,8000,8100,8200,8300,8400,8500,8600,8700,8800,8900,9000,9100,9200,9300,9400,9500,9600,9700,9800,9900,10000,10100,10200,10300,10400,10500,10600,10700,10800,10900,11000,11100,11200,11300,11400,11500,11600,11700,11800,11900,12000,12100,12200,12300,12400,12500,12600,12700,12800,12900,13000,13100,13200,13300,13400,13500,13600,13700,13800,13900,14000,14100,14200,14300,14400,14500,14600,14700,14800,14900,15000,15100,15200,15300,15400,15500,15600,15700,15800,15900,16000,16100,16200,16300,16400,16500,16600,16700,16800,16900,17000,17100,17200,17300,17400,17500,17600,17700,17800,17900,18000,18100,18200,18300,18400,18500,18600,18700,18800,18900,19000,19100,19200,19300,19400,19500,19600,19700,19800,19900,20000,20100,20200,20300,20400,20500,20600,20700,20800,20900,21000,21100,21200,21300,21400,21500,21600,21700,21800,21900
NW_003726722_1,Count,0,0,0,0,0,0,0,0,0,0,0,0,0,0,0,0,0,0,0,0,2,2,1,2,0,1,0,1,0,0,0,0,0,0,0,0,0,0,0,0,0,0,0,0,0,0,0,0,0,0,0,0,0,0,0,0,0,1,1,0,0,0,2,0,1,0,0,0,0,0,0,0,0,0,0,0,0,0,0,0,0,0,0,0,0,0,0,0,0,0,0,0,0,0,0,0,0,0,0,3,0,1,0,1,3,1,1,0,1,1,0,1,2,2,1,0,1,1,1,0,1,0,0,0,0,0,0,0,0,4,1,0,0,0,0,0,0,0,0,0,0,0,0,0,0,3,1,1,2,0,2,1,1,3,1,2,0,2,2,1,4,1,1,0,0,0,0,0,0,0,0,0,0,0,0,0,0,0,0,0,0,0,0,1,2,3,0,0,0,2,0,0,3,1,0,0,1,1,1,3,4,2,0,0,0,0,0,0,0,0,0,0,0,0,0,0,0,0,0,0

	
```

```
		  

		NW_003726729_1, Position,0,100,200,300,400,500,600,700,800,900,1000,1100,1200,1300,1400,1500,1600,1700,1800,1900,2000,2100,2200,2300,2400,2500,2600,2700,2800,2900,3000,3100,3200,3300,3400,3500,3600,3700,3800,3900,4000,4100,4200,4300,4400,4500,4600,4700,4800,4900,5000,5100,5200,5300,5400,5500,5600,5700,5800,5900,6000,6100,6200,6300,6400,6500,6600,6700,6800,6900,7000,7100,7200,7300,7400,7500,7600,7700,7800,7900,8000,8100,8200,8300,8400,8500,8600,8700,8800,8900,9000,9100,9200,9300,9400,9500,9600,9700,9800,9900,10000,10100,10200,10300,10400,10500,10600,10700,10800,10900,11000,11100,11200,11300,11400,11500,11600,11700,11800,11900,12000,12100,12200,12300,12400,12500,12600,12700,12800,12900,13000,13100,13200,13300,13400,13500,13600,13700,13800,13900,14000,14100,14200,14300,14400,14500,14600,14700,14800,14900,15000,15100,15200,15300,15400,15500,15600,15700,15800,15900,16000,16100,16200,16300,16400,16500,16600,16700,16800,16900,17000,17100,17200,17300,17400,17500,17600,17700,17800,17900,18000,18100,18200,18300,18400,18500,18600,18700,18800,18900,19000,19100,19200,19300,19400,19500,19600,19700,19800,19900,20000,20100,20200,20300,20400,20500,20600,20700,20800,20900,21000,21100,21200,21300,21400,21500,21600
NW_003726729_1,Count,0,0,0,0,0,0,0,1,1,2,0,0,2,1,0,0,0,0,0,0,0,0,0,0,0,0,0,0,0,0,0,0,0,0,1,1,2,4,0,0,0,1,0,1,1,0,2,0,0,0,0,2,2,1,2,2,2,0,0,0,0,0,0,2,2,1,3,1,0,1,0,3,1,0,1,0,0,0,0,0,0,0,0,0,0,0,0,0,0,0,0,0,0,0,0,0,0,0,0,0,0,0,0,0,0,0,0,0,0,0,0,0,0,0,0,0,0,0,0,0,2,1,0,0,0,0,2,1,2,2,1,1,0,2,0,2,1,0,0,0,0,0,0,0,0,2,0,3,0,1,3,0,0,0,0,0,0,0,0,0,0,1,1,0,0,0,0,3,0,0,0,0,0,0,0,0,0,0,1,1,0,1,1,2,0,1,3,1,1,1,1,0,1,0,2,1,0,3,0,0,1,0,0,0,1,1,0,0,0,2,0,2,1,0,4,0,0

	
```

```
		  

		NW_003726730_1, Position,0,100,200,300,400,500,600,700,800,900,1000,1100,1200,1300,1400,1500,1600,1700,1800,1900,2000,2100,2200,2300,2400,2500,2600,2700,2800,2900,3000,3100,3200,3300,3400,3500,3600,3700,3800,3900,4000,4100,4200,4300,4400,4500,4600,4700,4800,4900,5000,5100,5200,5300,5400,5500,5600,5700,5800,5900,6000,6100,6200,6300,6400,6500,6600,6700,6800,6900,7000,7100,7200,7300,7400,7500,7600,7700,7800,7900,8000,8100,8200,8300,8400,8500,8600,8700,8800,8900,9000,9100,9200,9300,9400,9500,9600,9700,9800,9900,10000,10100,10200,10300,10400,10500,10600,10700,10800,10900,11000,11100,11200,11300,11400,11500,11600,11700,11800,11900,12000,12100,12200,12300,12400,12500,12600,12700,12800,12900,13000,13100,13200,13300,13400,13500,13600,13700,13800,13900,14000,14100,14200,14300,14400,14500,14600,14700,14800,14900,15000,15100,15200,15300,15400,15500,15600,15700,15800,15900,16000,16100,16200,16300,16400,16500,16600,16700,16800,16900,17000,17100,17200,17300,17400,17500,17600,17700,17800,17900,18000,18100,18200,18300,18400,18500,18600,18700,18800,18900,19000,19100,19200,19300,19400,19500,19600,19700,19800,19900,20000,20100,20200,20300,20400,20500,20600,20700,20800,20900,21000,21100,21200,21300,21400,21500,21600
NW_003726730_1,Count,0,0,0,0,0,0,0,0,1,1,0,0,0,0,0,0,0,0,0,0,0,0,0,0,0,0,0,0,0,0,0,0,0,0,0,0,0,0,0,0,0,0,0,0,0,0,0,0,0,0,0,0,0,0,0,0,1,2,0,0,0,0,0,0,0,0,0,0,0,0,0,0,0,0,0,0,0,0,0,0,0,0,0,0,0,0,0,0,0,0,0,0,0,0,0,0,0,0,0,0,0,0,0,0,0,0,0,0,0,0,0,0,0,0,0,0,0,0,0,0,0,0,0,0,1,0,0,2,0,0,0,0,0,0,0,0,0,0,0,0,0,0,0,0,0,0,0,0,0,0,0,0,0,0,0,0,0,0,0,0,0,0,0,0,0,0,0,0,0,0,0,0,0,0,0,0,0,0,0,0,0,0,0,0,0,0,0,0,0,0,0,0,0,0,0,0,0,0,0,0,0,0,0,0,0,0,0,0,0,0,0,0,0,0,0,0,0

	
```

```
		  

		NW_003726735_1, Position,0,100,200,300,400,500,600,700,800,900,1000,1100,1200,1300,1400,1500,1600,1700,1800,1900,2000,2100,2200,2300,2400,2500,2600,2700,2800,2900,3000,3100,3200,3300,3400,3500,3600,3700,3800,3900,4000,4100,4200,4300,4400,4500,4600,4700,4800,4900,5000,5100,5200,5300,5400,5500,5600,5700,5800,5900,6000,6100,6200,6300,6400,6500,6600,6700,6800,6900,7000,7100,7200,7300,7400,7500,7600,7700,7800,7900,8000,8100,8200,8300,8400,8500,8600,8700,8800,8900,9000,9100,9200,9300,9400,9500,9600,9700,9800,9900,10000,10100,10200,10300,10400,10500,10600,10700,10800,10900,11000,11100,11200,11300,11400,11500,11600,11700,11800,11900,12000,12100,12200,12300,12400,12500,12600,12700,12800,12900,13000,13100,13200,13300,13400,13500,13600,13700,13800,13900,14000,14100,14200,14300,14400,14500,14600,14700,14800,14900,15000,15100,15200,15300,15400,15500,15600,15700,15800,15900,16000,16100,16200,16300,16400,16500,16600,16700,16800,16900,17000,17100,17200,17300,17400,17500,17600,17700,17800,17900,18000,18100,18200,18300,18400,18500,18600,18700,18800,18900,19000,19100,19200,19300,19400,19500,19600,19700,19800,19900,20000,20100,20200,20300,20400,20500,20600,20700,20800,20900,21000,21100,21200,21300,21400,21500,21600,21700,21800,21900,22000,22100,22200,22300,22400,22500,22600,22700,22800,22900,23000,23100,23200,23300,23400,23500,23600,23700,23800,23900,24000,24100,24200,24300,24400,24500,24600,24700,24800,24900,25000,25100,25200,25300,25400,25500,25600,25700,25800,25900,26000,26100,26200,26300,26400,26500,26600,26700,26800,26900,27000,27100,27200,27300,27400,27500,27600,27700,27800,27900,28000,28100,28200
NW_003726735_1,Count,0,0,0,0,0,0,0,0,0,0,0,0,0,0,0,0,0,0,0,0,0,0,0,0,0,0,0,0,0,0,0,0,0,0,0,0,0,0,0,0,0,0,0,0,0,0,0,0,0,0,0,0,0,0,0,0,0,0,0,0,0,0,0,0,0,0,0,0,0,0,0,0,0,0,0,0,0,0,0,0,0,0,0,0,0,0,0,0,0,0,0,0,0,0,0,0,0,0,0,0,0,0,0,0,0,0,0,0,0,0,0,0,1,1,0,2,0,0,0,0,0,0,0,0,0,0,0,0,0,0,0,0,0,0,0,0,0,0,0,0,0,0,0,0,0,0,0,0,0,0,0,0,0,0,0,0,0,0,0,0,0,0,0,0,0,0,0,0,0,0,1,0,1,0,0,0,0,0,0,0,0,0,0,0,0,0,0,0,0,0,0,0,0,0,0,0,0,0,0,0,0,0,0,0,0,0,0,0,0,0,0,0,0,0,0,0,1,1,1,0,0,0,0,0,0,0,0,0,0,0,0,0,0,0,0,0,0,0,0,0,0,0,0,0,0,0,0,0,0,0,0,0,0,0,0,0,0,0,0,0,0,0,0,0,0,0,0,0,0,0,0,0,0,0,0,0,0,0,0,0,0,0,0

	
```

```
		  

		NW_003726739_1, Position,0,1000,2000,3000,4000,5000,6000,7000,8000,9000,10000,11000,12000,13000,14000,15000,16000,17000,18000,19000,20000,21000,22000,23000,24000,25000,26000,27000,28000,29000,30000,31000,32000,33000,34000,35000,36000,37000,38000,39000,40000,41000,42000
NW_003726739_1,Count,0,0,0,0,0,0,0,0,0,0,0,0,0,0,0,0,0,6,0,0,0,0,0,0,0,0,0,0,0,0,0,0,0,0,0,0,0,0,0,0,0,0,0

	
```

```
		  

		NW_003726740_1, Position,0,100,200,300,400,500,600,700,800,900,1000,1100,1200,1300,1400,1500,1600,1700,1800,1900,2000,2100,2200,2300,2400,2500,2600,2700,2800,2900,3000,3100,3200,3300,3400,3500,3600,3700,3800,3900,4000,4100,4200,4300,4400,4500,4600,4700,4800,4900,5000,5100,5200,5300,5400,5500,5600,5700,5800,5900,6000,6100,6200,6300,6400,6500,6600,6700,6800,6900,7000,7100,7200,7300,7400,7500,7600,7700,7800,7900,8000,8100,8200,8300,8400,8500,8600,8700,8800,8900,9000,9100,9200,9300,9400,9500,9600,9700,9800,9900,10000,10100,10200,10300,10400,10500,10600,10700,10800,10900,11000,11100,11200,11300,11400,11500,11600,11700,11800,11900,12000,12100,12200,12300,12400,12500,12600,12700,12800,12900,13000,13100,13200,13300,13400,13500,13600,13700,13800,13900,14000,14100,14200,14300,14400,14500,14600,14700,14800,14900,15000,15100,15200,15300,15400,15500,15600,15700,15800,15900,16000,16100,16200,16300,16400,16500,16600,16700,16800,16900,17000,17100,17200,17300,17400,17500,17600,17700,17800,17900,18000,18100,18200,18300,18400,18500,18600,18700,18800,18900,19000,19100,19200,19300,19400,19500,19600,19700,19800,19900,20000,20100,20200,20300,20400,20500,20600,20700,20800,20900,21000,21100,21200,21300,21400,21500,21600,21700,21800,21900,22000,22100,22200,22300,22400,22500,22600,22700,22800,22900,23000,23100,23200,23300,23400,23500,23600,23700,23800,23900,24000,24100,24200,24300,24400,24500,24600,24700,24800,24900,25000,25100,25200,25300,25400,25500,25600,25700,25800,25900,26000,26100,26200,26300,26400,26500,26600,26700,26800,26900,27000,27100,27200,27300,27400,27500
NW_003726740_1,Count,0,0,3,1,0,1,1,0,2,0,2,0,1,0,2,0,0,0,1,1,1,0,0,0,1,1,1,0,0,0,0,1,0,0,2,0,3,0,1,0,0,0,0,0,0,1,0,0,1,0,1,3,1,1,0,1,0,0,1,0,1,0,0,1,1,0,0,0,1,1,0,1,0,0,0,0,1,0,0,0,1,0,0,3,0,0,1,1,1,1,1,0,0,0,0,0,2,2,1,0,0,0,0,0,0,0,0,0,0,0,0,0,0,0,0,0,0,0,0,0,0,0,0,0,0,0,0,0,0,0,0,0,0,0,0,0,0,0,0,0,0,0,0,0,0,0,0,0,0,0,0,0,0,0,0,0,0,0,0,0,0,0,0,0,0,0,0,0,0,0,0,0,0,0,0,0,0,0,0,0,0,0,0,0,0,0,0,0,0,0,0,0,0,0,0,0,1,0,1,0,2,1,2,1,3,1,0,2,2,0,1,1,0,0,0,0,3,1,0,0,2,0,1,4,0,2,1,0,0,0,0,0,0,0,0,0,0,0,0,1,1,0,1,1,1,0,0,0,1,1,0,0,0,0,1,0,0,0,0,0,0,0,0,0,2,0,0,0,2,1,1,2,1,0,0,0

	
```

```
		  

		NW_003726741_1, Position,0,100,200,300,400,500,600,700,800,900,1000,1100,1200,1300,1400,1500,1600,1700,1800,1900,2000,2100,2200,2300,2400,2500,2600,2700,2800,2900,3000,3100,3200,3300,3400,3500,3600,3700,3800,3900,4000,4100,4200,4300,4400,4500,4600,4700,4800,4900,5000,5100,5200,5300,5400,5500,5600,5700,5800,5900,6000,6100,6200,6300,6400,6500,6600,6700,6800,6900,7000,7100,7200,7300,7400,7500,7600,7700,7800,7900,8000,8100,8200,8300,8400,8500,8600,8700,8800,8900,9000,9100,9200,9300,9400,9500,9600,9700,9800,9900,10000,10100,10200,10300,10400,10500,10600,10700,10800,10900,11000,11100,11200,11300,11400,11500,11600,11700,11800,11900,12000,12100,12200,12300,12400,12500,12600,12700,12800,12900,13000,13100,13200,13300,13400,13500,13600,13700,13800,13900,14000,14100,14200,14300,14400,14500,14600,14700,14800,14900,15000,15100,15200,15300,15400,15500,15600,15700,15800,15900,16000,16100,16200,16300,16400,16500,16600,16700,16800,16900,17000,17100,17200,17300,17400,17500,17600,17700,17800,17900,18000,18100,18200,18300,18400,18500,18600,18700,18800,18900,19000,19100,19200,19300,19400,19500,19600,19700,19800,19900,20000,20100,20200,20300,20400,20500,20600,20700,20800,20900,21000,21100,21200,21300
NW_003726741_1,Count,0,0,0,0,0,0,0,0,0,0,0,0,1,1,0,2,0,0,0,0,1,0,2,1,1,0,0,0,1,0,0,3,0,2,0,1,0,0,0,0,0,0,0,1,0,0,0,0,1,0,1,1,1,0,1,3,0,1,0,3,1,0,2,0,0,0,0,0,0,3,1,0,1,1,0,1,0,2,1,2,1,1,0,1,0,2,0,0,0,0,2,0,1,0,1,1,1,0,0,0,0,0,0,2,1,1,0,0,1,0,1,0,1,1,1,1,2,1,1,0,0,2,0,0,0,0,0,0,0,2,0,0,0,0,0,1,1,0,2,0,1,0,0,0,0,0,0,0,1,1,2,0,0,1,1,0,0,0,0,1,0,3,1,0,0,1,0,1,0,1,0,0,0,1,1,0,1,0,1,1,2,0,0,0,0,2,0,0,0,2,1,1,1,0,0,1,1,0,1,2,0,1,1,0,3,0,1,0,0,0,0,0,0,0

	
```

```
		  

		NW_003726742_1, Position,0,100,200,300,400,500,600,700,800,900,1000,1100,1200,1300,1400,1500,1600,1700,1800,1900,2000,2100,2200,2300,2400,2500,2600,2700,2800,2900,3000,3100,3200,3300,3400,3500,3600,3700,3800,3900,4000,4100,4200,4300,4400,4500,4600,4700,4800,4900,5000,5100,5200,5300,5400,5500,5600,5700,5800,5900,6000,6100,6200,6300,6400,6500,6600,6700,6800,6900,7000,7100,7200,7300,7400,7500,7600,7700,7800,7900,8000,8100,8200,8300,8400,8500,8600,8700,8800,8900,9000,9100,9200,9300,9400,9500,9600,9700,9800,9900,10000,10100,10200,10300,10400,10500,10600,10700,10800,10900,11000,11100,11200,11300,11400,11500,11600,11700,11800,11900,12000,12100,12200,12300,12400,12500,12600,12700,12800,12900,13000,13100,13200,13300,13400,13500,13600,13700,13800,13900,14000,14100,14200,14300,14400,14500,14600,14700,14800,14900,15000,15100,15200,15300,15400,15500,15600,15700,15800,15900,16000,16100,16200,16300,16400,16500,16600,16700,16800,16900,17000,17100,17200,17300,17400,17500,17600,17700,17800,17900,18000,18100,18200,18300,18400,18500,18600,18700,18800,18900,19000,19100,19200,19300,19400,19500,19600,19700,19800,19900,20000,20100,20200,20300,20400,20500,20600,20700,20800,20900,21000,21100,21200
NW_003726742_1,Count,0,0,0,0,0,0,0,0,0,0,0,0,0,0,0,0,0,0,0,0,0,0,0,0,0,0,0,0,0,0,0,0,0,0,0,0,0,0,0,0,0,0,0,0,0,0,0,0,0,0,0,0,0,0,0,0,0,0,0,0,0,0,0,0,0,0,0,0,1,2,1,2,2,2,1,4,0,0,1,1,1,1,0,0,0,0,0,0,0,0,0,0,0,0,0,0,0,0,0,1,2,2,0,0,0,0,0,0,0,0,0,0,0,0,0,0,1,1,1,0,0,0,0,0,0,0,0,0,0,0,0,0,0,0,0,0,0,1,0,0,0,0,0,0,0,0,0,0,0,0,0,1,0,0,0,0,0,0,0,0,0,0,0,1,1,1,0,0,0,0,0,0,0,0,0,0,1,0,0,0,0,0,0,0,0,0,0,0,0,0,0,0,0,0,0,0,0,0,0,0,0,0,0,0,0,0,0,0,0,0,0,0,0

	
```

```
		  

		NW_003726743_1, Position,0,100,200,300,400,500,600,700,800,900,1000,1100,1200,1300,1400,1500,1600,1700,1800,1900,2000,2100,2200,2300,2400,2500,2600,2700,2800,2900,3000,3100,3200,3300,3400,3500,3600,3700,3800,3900,4000,4100,4200,4300,4400,4500,4600,4700,4800,4900,5000,5100,5200,5300,5400,5500,5600,5700,5800,5900,6000,6100,6200,6300,6400,6500,6600,6700,6800,6900,7000,7100,7200,7300,7400,7500,7600,7700,7800,7900,8000,8100,8200,8300,8400,8500,8600,8700,8800,8900,9000,9100,9200,9300,9400,9500,9600,9700,9800,9900,10000,10100,10200,10300,10400,10500,10600,10700,10800,10900,11000,11100,11200,11300,11400,11500,11600,11700,11800,11900,12000,12100,12200,12300,12400,12500,12600,12700,12800,12900,13000,13100,13200,13300,13400,13500,13600,13700,13800,13900,14000,14100,14200,14300,14400,14500,14600,14700,14800,14900,15000,15100,15200,15300,15400,15500,15600,15700,15800,15900,16000,16100,16200,16300,16400,16500,16600,16700,16800,16900,17000,17100,17200,17300,17400,17500,17600,17700,17800,17900,18000,18100,18200,18300,18400,18500,18600,18700,18800,18900,19000,19100,19200,19300,19400,19500,19600,19700,19800,19900,20000,20100,20200,20300,20400,20500,20600,20700,20800,20900,21000,21100,21200,21300,21400,21500,21600,21700,21800,21900,22000,22100,22200,22300,22400,22500,22600,22700,22800,22900,23000
NW_003726743_1,Count,0,0,0,0,0,0,0,0,0,0,0,0,0,0,0,0,0,0,0,0,0,0,0,0,0,0,0,0,0,0,0,0,0,0,0,0,0,0,0,0,0,0,0,0,0,0,0,0,0,0,0,0,0,0,0,0,0,0,0,0,0,0,0,0,0,0,0,0,0,0,0,0,0,1,0,0,1,0,0,0,0,0,0,0,0,0,0,0,0,0,0,0,0,0,0,0,0,0,0,0,0,0,0,0,0,0,0,0,0,0,0,0,0,0,0,0,0,0,0,0,0,0,0,0,0,0,0,0,0,0,0,0,0,0,0,0,0,0,0,0,0,0,0,0,0,0,0,0,0,0,0,0,0,0,0,0,0,0,0,0,0,0,0,0,0,0,0,0,0,0,0,0,0,0,0,0,0,0,0,0,0,0,0,0,0,0,0,0,0,0,0,0,0,0,0,0,0,0,0,0,0,0,0,0,0,0,0,0,0,0,0,0,0,0,0,0,0,0,0,0,0,0,0,0,0,0,0,0,0,0,0

	
```

```
		  

		NW_003726745_1, Position,0,100,200,300,400,500,600,700,800,900,1000,1100,1200,1300,1400,1500,1600,1700,1800,1900,2000,2100,2200,2300,2400,2500,2600,2700,2800,2900,3000,3100,3200,3300,3400,3500,3600,3700,3800,3900,4000,4100,4200,4300,4400,4500,4600,4700,4800,4900,5000,5100,5200,5300,5400,5500,5600,5700,5800,5900,6000,6100,6200,6300,6400,6500,6600,6700,6800,6900,7000,7100,7200,7300,7400,7500,7600,7700,7800,7900,8000,8100,8200,8300,8400,8500,8600,8700,8800,8900,9000,9100,9200,9300,9400,9500,9600,9700,9800,9900,10000,10100,10200,10300,10400,10500,10600,10700,10800,10900,11000,11100,11200,11300,11400,11500,11600,11700,11800,11900,12000,12100,12200,12300,12400,12500,12600,12700,12800,12900,13000,13100,13200,13300,13400,13500,13600,13700,13800,13900,14000,14100,14200,14300,14400,14500,14600,14700,14800,14900,15000,15100,15200,15300,15400,15500,15600,15700,15800,15900,16000,16100,16200,16300,16400,16500,16600,16700,16800,16900,17000,17100,17200,17300,17400,17500,17600,17700,17800,17900,18000,18100,18200,18300,18400,18500,18600,18700,18800,18900,19000,19100,19200,19300,19400,19500,19600,19700,19800,19900,20000,20100,20200,20300,20400,20500,20600,20700,20800,20900,21000,21100,21200,21300,21400,21500,21600,21700,21800,21900,22000,22100,22200,22300,22400,22500,22600,22700,22800,22900,23000,23100,23200,23300,23400,23500,23600,23700,23800,23900,24000,24100,24200,24300,24400,24500,24600,24700,24800,24900
NW_003726745_1,Count,0,5,1,4,1,2,0,0,0,0,0,0,4,3,1,0,0,0,0,0,0,0,0,0,0,0,0,0,0,0,0,0,0,0,0,0,0,0,0,0,0,0,0,0,0,0,0,0,0,0,0,0,0,0,0,0,0,0,0,0,0,0,0,0,0,0,0,0,0,0,0,0,0,0,0,0,0,0,0,0,0,0,0,0,0,0,0,0,0,0,0,0,0,0,0,0,0,0,0,0,0,0,0,0,0,0,0,0,0,0,0,0,0,0,0,0,0,0,0,0,0,0,0,0,0,0,0,0,0,0,0,0,0,0,0,1,0,0,0,0,0,0,0,0,0,0,0,0,2,2,1,0,0,0,0,0,0,0,0,0,0,0,0,0,0,0,0,0,0,0,0,0,0,0,0,0,0,0,0,0,0,0,0,0,0,0,0,0,0,0,0,0,0,0,0,0,0,0,0,0,0,0,0,0,0,0,0,0,0,0,0,0,0,0,0,0,0,0,0,0,0,0,0,0,0,0,0,0,0,0,0,0,0,0,0,0,0,0,0,0,0,0,0,0,0,0,0,0,0,0

	
```

```
		  

		NW_003726747_1, Position,0,100,200,300,400,500,600,700,800,900,1000,1100,1200,1300,1400,1500,1600,1700,1800,1900,2000,2100,2200,2300,2400,2500,2600,2700,2800,2900,3000,3100,3200,3300,3400,3500,3600,3700,3800,3900,4000,4100,4200,4300,4400,4500,4600,4700,4800,4900,5000,5100,5200,5300,5400,5500,5600,5700,5800,5900,6000,6100,6200,6300,6400,6500,6600,6700,6800,6900,7000,7100,7200,7300,7400,7500,7600,7700,7800,7900,8000,8100,8200,8300,8400,8500,8600,8700,8800,8900,9000,9100,9200,9300,9400,9500,9600,9700,9800,9900,10000,10100,10200,10300,10400,10500,10600,10700,10800,10900,11000,11100,11200,11300,11400,11500,11600,11700,11800,11900,12000,12100,12200,12300,12400,12500,12600,12700,12800,12900,13000,13100,13200,13300,13400,13500,13600,13700,13800,13900,14000,14100,14200,14300,14400,14500,14600,14700,14800,14900,15000,15100,15200,15300,15400,15500,15600,15700,15800,15900,16000,16100,16200,16300,16400,16500,16600,16700,16800,16900,17000,17100,17200,17300,17400,17500,17600,17700,17800,17900,18000,18100,18200,18300,18400,18500,18600,18700,18800,18900,19000,19100,19200,19300,19400,19500,19600,19700,19800,19900,20000,20100,20200,20300,20400,20500,20600,20700,20800,20900,21000
NW_003726747_1,Count,0,0,0,0,0,0,0,0,0,0,0,0,0,0,0,0,0,0,0,0,0,0,0,0,0,0,0,0,2,1,1,0,0,0,0,0,0,0,0,0,0,0,0,0,0,0,0,0,0,0,0,0,0,0,0,0,0,0,0,0,1,0,0,0,0,0,0,0,0,0,0,0,0,0,0,0,0,0,0,0,0,0,0,0,0,0,0,0,0,0,0,0,1,0,0,0,0,0,0,0,0,0,0,0,0,0,0,0,0,0,0,0,0,0,0,0,0,0,0,0,0,0,1,1,1,0,0,0,3,2,1,0,0,0,0,0,0,2,2,0,1,2,0,0,0,0,0,0,0,0,2,0,0,0,0,0,0,0,0,0,0,0,0,0,0,0,0,0,0,0,0,0,0,0,0,0,0,0,0,0,0,0,0,0,0,0,0,0,0,0,0,0,0,0,0,0,0,0,0,0,0,0,0,0,0,0,0,0,0,0,0

	
```

```
		  

		NW_003726748_1, Position,0,100,200,300,400,500,600,700,800,900,1000,1100,1200,1300,1400,1500,1600,1700,1800,1900,2000,2100,2200,2300,2400,2500,2600,2700,2800,2900,3000,3100,3200,3300,3400,3500,3600,3700,3800,3900,4000,4100,4200,4300,4400,4500,4600,4700,4800,4900,5000,5100,5200,5300,5400,5500,5600,5700,5800,5900,6000,6100,6200,6300,6400,6500,6600,6700,6800,6900,7000,7100,7200,7300,7400,7500,7600,7700,7800,7900,8000,8100,8200,8300,8400,8500,8600,8700,8800,8900,9000,9100,9200,9300,9400,9500,9600,9700,9800,9900,10000,10100,10200,10300,10400,10500,10600,10700,10800,10900,11000,11100,11200,11300,11400,11500,11600,11700,11800,11900,12000,12100,12200,12300,12400,12500,12600,12700,12800,12900,13000,13100,13200,13300,13400,13500,13600,13700,13800,13900,14000,14100,14200,14300,14400,14500,14600,14700,14800,14900,15000,15100,15200,15300,15400,15500,15600,15700,15800,15900,16000,16100,16200,16300,16400,16500,16600,16700,16800,16900,17000,17100,17200,17300,17400,17500,17600,17700,17800,17900,18000,18100,18200,18300,18400,18500,18600,18700,18800,18900,19000,19100,19200,19300,19400,19500,19600,19700,19800,19900,20000,20100,20200,20300,20400,20500,20600,20700,20800,20900,21000
NW_003726748_1,Count,0,0,0,0,1,0,0,1,0,1,0,0,0,0,0,3,0,0,0,1,3,0,0,1,0,0,0,0,0,1,0,0,1,0,0,2,1,1,2,0,0,0,1,0,1,1,0,0,0,0,0,0,0,0,0,0,0,0,0,0,0,0,0,0,0,0,0,0,0,0,0,0,0,0,0,0,0,0,0,0,0,0,0,0,0,0,0,0,0,0,0,0,0,0,0,0,0,0,0,0,0,0,0,0,0,0,0,0,0,0,0,0,0,0,0,0,0,0,0,0,0,0,0,0,0,0,0,0,0,0,0,0,0,0,0,0,0,0,0,0,0,0,0,0,0,0,0,0,0,1,0,1,0,1,1,0,1,0,2,0,0,0,1,1,1,0,0,0,0,1,0,0,0,0,0,0,0,0,0,0,0,0,0,0,0,0,0,0,0,0,0,0,0,0,0,0,0,0,0,1,1,0,0,2,0,0,1,0,0,0,0

	
```

```
		  

		NW_003726749_1, Position,0,100,200,300,400,500,600,700,800,900,1000,1100,1200,1300,1400,1500,1600,1700,1800,1900,2000,2100,2200,2300,2400,2500,2600,2700,2800,2900,3000,3100,3200,3300,3400,3500,3600,3700,3800,3900,4000,4100,4200,4300,4400,4500,4600,4700,4800,4900,5000,5100,5200,5300,5400,5500,5600,5700,5800,5900,6000,6100,6200,6300,6400,6500,6600,6700,6800,6900,7000,7100,7200,7300,7400,7500,7600,7700,7800,7900,8000,8100,8200,8300,8400,8500,8600,8700,8800,8900,9000,9100,9200,9300,9400,9500,9600,9700,9800,9900,10000,10100,10200,10300,10400,10500,10600,10700,10800,10900,11000,11100,11200,11300,11400,11500,11600,11700,11800,11900,12000,12100,12200,12300,12400,12500,12600,12700,12800,12900,13000,13100,13200,13300,13400,13500,13600,13700,13800,13900,14000,14100,14200,14300,14400,14500,14600,14700,14800,14900,15000,15100,15200,15300,15400,15500,15600,15700,15800,15900,16000,16100,16200,16300,16400,16500,16600,16700,16800,16900,17000,17100,17200,17300,17400,17500,17600,17700,17800,17900,18000,18100,18200,18300,18400,18500,18600,18700,18800,18900,19000,19100,19200,19300,19400,19500,19600,19700,19800,19900,20000,20100,20200,20300,20400,20500,20600,20700,20800,20900,21000,21100,21200,21300,21400,21500,21600,21700,21800,21900,22000
NW_003726749_1,Count,1,1,1,1,3,0,0,2,0,0,0,0,1,1,0,1,1,0,1,1,0,1,2,3,0,0,1,0,0,1,0,3,1,0,0,0,0,0,0,0,0,0,1,1,2,1,1,1,0,1,1,2,0,1,0,2,1,2,3,0,0,0,0,1,1,0,0,0,1,1,0,1,0,0,1,0,0,1,1,0,1,2,1,1,0,0,0,1,2,1,1,1,0,0,2,0,0,0,0,0,0,0,0,0,0,0,0,0,0,0,0,0,0,0,0,0,0,0,0,0,0,0,0,0,1,1,0,1,0,2,2,2,2,1,1,2,1,2,1,0,2,0,0,0,0,0,0,0,3,0,0,0,0,0,1,1,1,2,2,1,2,0,0,0,0,0,1,1,0,0,1,0,1,0,0,0,2,1,1,0,0,2,2,1,0,2,1,2,2,0,1,2,1,1,0,4,1,1,2,2,2,1,1,1,1,2,1,0,0,2,1,1,1,0,3,0,0,1,0,1,0

	
```

```
		  

		NW_003726751_1, Position,0,100,200,300,400,500,600,700,800,900,1000,1100,1200,1300,1400,1500,1600,1700,1800,1900,2000,2100,2200,2300,2400,2500,2600,2700,2800,2900,3000,3100,3200,3300,3400,3500,3600,3700,3800,3900,4000,4100,4200,4300,4400,4500,4600,4700,4800,4900,5000,5100,5200,5300,5400,5500,5600,5700,5800,5900,6000,6100,6200,6300,6400,6500,6600,6700,6800,6900,7000,7100,7200,7300,7400,7500,7600,7700,7800,7900,8000,8100,8200,8300,8400,8500,8600,8700,8800,8900,9000,9100,9200,9300,9400,9500,9600,9700,9800,9900,10000,10100,10200,10300,10400,10500,10600,10700,10800,10900,11000,11100,11200,11300,11400,11500,11600,11700,11800,11900,12000,12100,12200,12300,12400,12500,12600,12700,12800,12900,13000,13100,13200,13300,13400,13500,13600,13700,13800,13900,14000,14100,14200,14300,14400,14500,14600,14700,14800,14900,15000,15100,15200,15300,15400,15500,15600,15700,15800,15900,16000,16100,16200,16300,16400,16500,16600,16700,16800,16900,17000,17100,17200,17300,17400,17500,17600,17700,17800,17900,18000,18100,18200,18300,18400,18500,18600,18700,18800,18900,19000,19100,19200,19300,19400,19500,19600,19700,19800,19900,20000,20100,20200,20300,20400,20500,20600,20700,20800,20900
NW_003726751_1,Count,1,0,2,2,1,0,2,2,1,2,2,0,2,1,1,1,3,0,1,0,1,0,1,0,0,1,0,0,0,0,1,1,2,0,0,0,0,0,0,1,1,0,0,1,0,1,0,1,0,3,1,1,0,2,1,2,0,0,0,2,1,0,1,1,2,1,0,0,3,0,1,1,0,0,1,0,1,2,1,0,1,1,0,4,2,1,2,0,0,0,1,2,2,2,2,1,2,0,2,3,2,0,2,2,2,3,0,0,0,1,0,3,1,2,1,0,0,0,0,3,0,0,1,1,2,2,1,1,2,0,3,1,1,1,2,1,2,2,1,1,1,3,0,0,0,3,1,1,2,0,0,0,1,1,4,0,0,0,1,2,3,0,1,2,2,2,1,1,1,0,0,2,0,0,0,1,0,2,2,0,0,1,1,0,0,2,1,1,3,1,1,2,2,0,1,0,0,0,0,0,0,0,0,0,0,0,0,0,0,0

	
```

```
		  

		NW_003726755_1, Position,0,100,200,300,400,500,600,700,800,900,1000,1100,1200,1300,1400,1500,1600,1700,1800,1900,2000,2100,2200,2300,2400,2500,2600,2700,2800,2900,3000,3100,3200,3300,3400,3500,3600,3700,3800,3900,4000,4100,4200,4300,4400,4500,4600,4700,4800,4900,5000,5100,5200,5300,5400,5500,5600,5700,5800,5900,6000,6100,6200,6300,6400,6500,6600,6700,6800,6900,7000,7100,7200,7300,7400,7500,7600,7700,7800,7900,8000,8100,8200,8300,8400,8500,8600,8700,8800,8900,9000,9100,9200,9300,9400,9500,9600,9700,9800,9900,10000,10100,10200,10300,10400,10500,10600,10700,10800,10900,11000,11100,11200,11300,11400,11500,11600,11700,11800,11900,12000,12100,12200,12300,12400,12500,12600,12700,12800,12900,13000,13100,13200,13300,13400,13500,13600,13700,13800,13900,14000,14100,14200,14300,14400,14500,14600,14700,14800,14900,15000,15100,15200,15300,15400,15500,15600,15700,15800,15900,16000,16100,16200,16300,16400,16500,16600,16700,16800,16900,17000,17100,17200,17300,17400,17500,17600,17700,17800,17900,18000,18100,18200,18300,18400,18500,18600,18700,18800,18900,19000,19100,19200,19300,19400,19500,19600,19700,19800,19900,20000,20100,20200,20300,20400,20500,20600,20700,20800
NW_003726755_1,Count,0,0,0,0,0,0,0,0,0,0,0,0,0,2,1,1,1,1,0,0,0,0,0,0,0,0,0,0,0,0,0,0,0,0,0,0,0,0,0,0,0,0,0,0,0,0,0,0,0,0,0,0,0,0,0,0,0,0,0,0,0,0,0,0,0,0,0,0,0,0,0,0,0,0,0,0,0,0,0,0,0,0,0,0,0,0,0,0,0,0,0,0,0,0,0,0,0,0,0,0,0,0,0,0,0,0,0,0,0,0,0,0,0,0,0,0,0,0,0,0,0,0,0,0,0,0,0,0,0,0,0,0,0,0,0,0,0,0,0,0,0,0,0,0,0,0,0,0,0,0,0,0,0,0,0,0,0,0,0,0,0,0,0,0,0,0,0,0,0,0,0,0,0,0,0,0,0,0,0,0,0,0,0,0,0,0,0,0,0,0,0,0,0,0,0,0,0,0,0,0,0,0,0,0,0,0,0,0,0

	
```

```
		  

		NW_003726758_1, Position,0,100,200,300,400,500,600,700,800,900,1000,1100,1200,1300,1400,1500,1600,1700,1800,1900,2000,2100,2200,2300,2400,2500,2600,2700,2800,2900,3000,3100,3200,3300,3400,3500,3600,3700,3800,3900,4000,4100,4200,4300,4400,4500,4600,4700,4800,4900,5000,5100,5200,5300,5400,5500,5600,5700,5800,5900,6000,6100,6200,6300,6400,6500,6600,6700,6800,6900,7000,7100,7200,7300,7400,7500,7600,7700,7800,7900,8000,8100,8200,8300,8400,8500,8600,8700,8800,8900,9000,9100,9200,9300,9400,9500,9600,9700,9800,9900,10000,10100,10200,10300,10400,10500,10600,10700,10800,10900,11000,11100,11200,11300,11400,11500,11600,11700,11800,11900,12000,12100,12200,12300,12400,12500,12600,12700,12800,12900,13000,13100,13200,13300,13400,13500,13600,13700,13800,13900,14000,14100,14200,14300,14400,14500,14600,14700,14800,14900,15000,15100,15200,15300,15400,15500,15600,15700,15800,15900,16000,16100,16200,16300,16400,16500,16600,16700,16800,16900,17000,17100,17200,17300,17400,17500,17600,17700,17800,17900,18000,18100,18200,18300,18400,18500,18600,18700,18800,18900,19000,19100,19200,19300,19400,19500,19600,19700,19800,19900,20000,20100,20200,20300,20400,20500,20600,20700
NW_003726758_1,Count,0,0,0,0,0,0,0,0,0,0,0,0,0,0,0,0,0,0,0,0,0,0,0,0,1,0,1,0,0,0,0,0,0,0,0,0,0,0,0,0,0,0,0,0,0,0,0,0,0,0,0,0,0,0,0,0,0,0,0,0,0,0,0,0,0,0,0,1,0,0,0,1,1,0,0,0,0,1,0,1,1,2,0,1,1,0,0,0,0,0,0,0,0,0,0,0,0,0,0,0,0,0,0,0,0,0,0,0,0,0,0,0,0,0,0,0,0,0,0,0,0,0,0,0,0,0,0,0,0,0,0,0,2,1,0,0,0,0,0,0,0,0,0,0,0,0,0,0,3,0,0,0,0,0,0,0,0,0,0,0,0,0,0,0,0,0,0,0,0,0,0,0,0,0,0,0,0,0,0,0,0,0,0,0,0,0,0,0,0,0,0,0,0,0,0,0,0,0,0,0,0,0,0,0,0,0,0,0

	
```

```
		  

		NW_003726759_1, Position,0,1000,2000,3000,4000,5000,6000,7000,8000,9000,10000,11000,12000,13000,14000,15000,16000,17000,18000,19000,20000,21000,22000,23000,24000,25000,26000,27000,28000,29000,30000,31000,32000,33000,34000
NW_003726759_1,Count,4,5,0,0,0,1,0,0,0,11,0,0,0,7,4,0,6,6,9,4,0,0,0,0,0,0,0,0,6,6,0,8,11,6,3

	
```

```
		  

		NW_003726760_1, Position,0,100,200,300,400,500,600,700,800,900,1000,1100,1200,1300,1400,1500,1600,1700,1800,1900,2000,2100,2200,2300,2400,2500,2600,2700,2800,2900,3000,3100,3200,3300,3400,3500,3600,3700,3800,3900,4000,4100,4200,4300,4400,4500,4600,4700,4800,4900,5000,5100,5200,5300,5400,5500,5600,5700,5800,5900,6000,6100,6200,6300,6400,6500,6600,6700,6800,6900,7000,7100,7200,7300,7400,7500,7600,7700,7800,7900,8000,8100,8200,8300,8400,8500,8600,8700,8800,8900,9000,9100,9200,9300,9400,9500,9600,9700,9800,9900,10000,10100,10200,10300,10400,10500,10600,10700,10800,10900,11000,11100,11200,11300,11400,11500,11600,11700,11800,11900,12000,12100,12200,12300,12400,12500,12600,12700,12800,12900,13000,13100,13200,13300,13400,13500,13600,13700,13800,13900,14000,14100,14200,14300,14400,14500,14600,14700,14800,14900,15000,15100,15200,15300,15400,15500,15600,15700,15800,15900,16000,16100,16200,16300,16400,16500,16600,16700,16800,16900,17000,17100,17200,17300,17400,17500,17600,17700,17800,17900,18000,18100,18200,18300,18400,18500,18600,18700,18800,18900,19000,19100,19200,19300,19400,19500,19600,19700,19800,19900,20000,20100,20200,20300,20400,20500,20600,20700,20800,20900,21000
NW_003726760_1,Count,0,0,0,0,0,0,0,0,0,0,0,0,0,0,0,0,0,0,0,0,0,0,0,0,0,0,0,0,0,0,0,0,0,0,0,0,0,0,0,0,0,0,0,0,0,0,0,0,0,0,0,0,0,0,0,0,0,0,0,0,0,0,0,0,0,0,0,0,0,1,0,1,0,0,0,0,0,0,0,0,0,0,0,0,0,0,0,0,0,0,0,0,0,0,0,0,0,0,0,0,0,0,0,0,0,0,0,0,0,0,0,0,0,0,0,0,0,0,0,0,0,0,0,0,0,0,0,0,0,0,0,0,0,0,0,0,0,0,0,0,0,0,0,0,0,0,0,0,0,0,0,0,0,0,0,0,0,0,0,0,0,0,0,0,0,0,0,0,0,0,0,0,0,0,0,0,0,0,0,0,0,0,0,0,0,0,0,0,0,0,0,0,0,0,0,0,0,0,0,0,0,0,0,0,0,0,0,0,0,0,0

	
```

```
		  

		NW_003726764_1, Position,0,100,200,300,400,500,600,700,800,900,1000,1100,1200,1300,1400,1500,1600,1700,1800,1900,2000,2100,2200,2300,2400,2500,2600,2700,2800,2900,3000,3100,3200,3300,3400,3500,3600,3700,3800,3900,4000,4100,4200,4300,4400,4500,4600,4700,4800,4900,5000,5100,5200,5300,5400,5500,5600,5700,5800,5900,6000,6100,6200,6300,6400,6500,6600,6700,6800,6900,7000,7100,7200,7300,7400,7500,7600,7700,7800,7900,8000,8100,8200,8300,8400,8500,8600,8700,8800,8900,9000,9100,9200,9300,9400,9500,9600,9700,9800,9900,10000,10100,10200,10300,10400,10500,10600,10700,10800,10900,11000,11100,11200,11300,11400,11500,11600,11700,11800,11900,12000,12100,12200,12300,12400,12500,12600,12700,12800,12900,13000,13100,13200,13300,13400,13500,13600,13700,13800,13900,14000,14100,14200,14300,14400,14500,14600,14700,14800,14900,15000,15100,15200,15300,15400,15500,15600,15700,15800,15900,16000,16100,16200,16300,16400,16500,16600,16700,16800,16900,17000,17100,17200,17300,17400,17500,17600,17700,17800,17900,18000,18100,18200,18300,18400,18500,18600,18700,18800,18900,19000,19100,19200,19300,19400,19500,19600,19700,19800,19900,20000,20100,20200,20300,20400,20500
NW_003726764_1,Count,0,0,0,0,0,0,0,0,1,3,0,0,0,0,1,3,0,0,2,0,0,0,1,0,0,0,0,0,2,1,2,1,2,0,0,1,3,0,1,3,1,1,2,2,0,1,0,0,2,0,3,0,0,1,2,1,1,0,0,3,1,0,2,1,1,1,2,0,1,0,2,1,1,0,0,2,0,0,2,0,2,0,2,1,0,1,1,1,0,0,0,0,0,0,0,0,0,0,0,0,1,2,2,0,0,0,0,0,0,0,0,0,1,1,0,0,0,0,0,0,0,0,0,0,0,0,0,0,0,0,0,0,0,0,0,0,0,0,0,0,0,0,0,0,0,0,0,0,0,0,0,0,0,0,0,0,0,0,0,0,1,0,0,0,1,0,0,0,1,1,0,0,0,0,0,0,0,0,0,0,0,0,0,0,0,0,0,0,0,0,0,0,0,0,0,0,0,0,0,2,1,0,0,0,0,0

	
```

```
		  

		NW_003726768_1, Position,0,100,200,300,400,500,600,700,800,900,1000,1100,1200,1300,1400,1500,1600,1700,1800,1900,2000,2100,2200,2300,2400,2500,2600,2700,2800,2900,3000,3100,3200,3300,3400,3500,3600,3700,3800,3900,4000,4100,4200,4300,4400,4500,4600,4700,4800,4900,5000,5100,5200,5300,5400,5500,5600,5700,5800,5900,6000,6100,6200,6300,6400,6500,6600,6700,6800,6900,7000,7100,7200,7300,7400,7500,7600,7700,7800,7900,8000,8100,8200,8300,8400,8500,8600,8700,8800,8900,9000,9100,9200,9300,9400,9500,9600,9700,9800,9900,10000,10100,10200,10300,10400,10500,10600,10700,10800,10900,11000,11100,11200,11300,11400,11500,11600,11700,11800,11900,12000,12100,12200,12300,12400,12500,12600,12700,12800,12900,13000,13100,13200,13300,13400,13500,13600,13700,13800,13900,14000,14100,14200,14300,14400,14500,14600,14700,14800,14900,15000,15100,15200,15300,15400,15500,15600,15700,15800,15900,16000,16100,16200,16300,16400,16500,16600,16700,16800,16900,17000,17100,17200,17300,17400,17500,17600,17700,17800,17900,18000,18100,18200,18300,18400,18500,18600,18700,18800,18900,19000,19100,19200,19300,19400,19500,19600,19700,19800,19900,20000,20100,20200,20300,20400
NW_003726768_1,Count,0,0,0,0,0,0,0,0,0,0,0,0,0,0,0,0,0,0,0,0,0,0,0,0,0,0,0,0,0,0,0,0,0,0,0,0,0,0,0,0,0,0,0,0,0,0,0,0,0,0,0,0,0,0,0,0,0,1,1,0,0,0,0,0,0,1,0,0,0,0,0,0,0,0,0,0,0,0,0,0,0,0,0,0,0,0,0,0,0,0,0,0,0,0,0,0,0,0,0,0,0,0,0,0,0,0,0,0,0,0,0,0,0,0,0,0,4,1,0,0,0,0,1,2,0,0,0,0,0,0,0,0,0,0,0,0,0,0,0,0,0,0,0,0,0,0,0,0,0,0,0,0,0,0,0,0,0,0,0,0,0,1,1,1,1,0,0,0,0,0,0,0,0,1,0,0,0,0,0,0,0,0,0,0,0,0,0,0,0,0,0,0,0,0,0,0,0,0,0,0,0,0,0,0,0

	
```

```
		  

		NW_003726769_1, Position,0,100,200,300,400,500,600,700,800,900,1000,1100,1200,1300,1400,1500,1600,1700,1800,1900,2000,2100,2200,2300,2400,2500,2600,2700,2800,2900,3000,3100,3200,3300,3400,3500,3600,3700,3800,3900,4000,4100,4200,4300,4400,4500,4600,4700,4800,4900,5000,5100,5200,5300,5400,5500,5600,5700,5800,5900,6000,6100,6200,6300,6400,6500,6600,6700,6800,6900,7000,7100,7200,7300,7400,7500,7600,7700,7800,7900,8000,8100,8200,8300,8400,8500,8600,8700,8800,8900,9000,9100,9200,9300,9400,9500,9600,9700,9800,9900,10000,10100,10200,10300,10400,10500,10600,10700,10800,10900,11000,11100,11200,11300,11400,11500,11600,11700,11800,11900,12000,12100,12200,12300,12400,12500,12600,12700,12800,12900,13000,13100,13200,13300,13400,13500,13600,13700,13800,13900,14000,14100,14200,14300,14400,14500,14600,14700,14800,14900,15000,15100,15200,15300,15400,15500,15600,15700,15800,15900,16000,16100,16200,16300,16400,16500,16600,16700,16800,16900,17000,17100,17200,17300,17400,17500,17600,17700,17800,17900,18000,18100,18200,18300,18400,18500,18600,18700,18800,18900,19000,19100,19200,19300,19400,19500,19600,19700,19800,19900,20000,20100,20200,20300,20400
NW_003726769_1,Count,0,0,0,0,0,0,0,0,0,0,0,0,0,0,0,0,0,0,0,0,0,0,0,0,0,0,0,0,0,0,0,0,0,0,0,0,0,0,0,0,0,0,0,0,0,0,0,0,0,0,0,0,0,0,0,0,0,0,0,0,0,0,0,0,0,0,0,0,0,0,0,0,0,0,0,0,0,0,0,0,0,0,0,0,0,0,0,0,0,0,0,0,0,0,0,0,0,0,0,0,0,0,0,0,0,0,0,0,0,0,0,0,0,0,0,0,0,0,1,1,1,0,1,2,2,3,0,2,1,0,0,0,0,0,0,0,0,0,0,0,0,0,0,0,0,0,2,1,1,0,1,0,2,1,2,0,1,0,2,0,0,1,0,2,0,1,3,0,1,2,0,0,0,0,0,0,0,0,0,0,0,0,0,2,0,0,0,0,0,0,0,0,1,2,3,3,1,0,0,0,1,3,0,0,0

	
```

```
		  

		NW_003726780_1, Position,0,100,200,300,400,500,600,700,800,900,1000,1100,1200,1300,1400,1500,1600,1700,1800,1900,2000,2100,2200,2300,2400,2500,2600,2700,2800,2900,3000,3100,3200,3300,3400,3500,3600,3700,3800,3900,4000,4100,4200,4300,4400,4500,4600,4700,4800,4900,5000,5100,5200,5300,5400,5500,5600,5700,5800,5900,6000,6100,6200,6300,6400,6500,6600,6700,6800,6900,7000,7100,7200,7300,7400,7500,7600,7700,7800,7900,8000,8100,8200,8300,8400,8500,8600,8700,8800,8900,9000,9100,9200,9300,9400,9500,9600,9700,9800,9900,10000,10100,10200,10300,10400,10500,10600,10700,10800,10900,11000,11100,11200,11300,11400,11500,11600,11700,11800,11900,12000,12100,12200,12300,12400,12500,12600,12700,12800,12900,13000,13100,13200,13300,13400,13500,13600,13700,13800,13900,14000,14100,14200,14300,14400,14500,14600,14700,14800,14900,15000,15100,15200,15300,15400,15500,15600,15700,15800,15900,16000,16100,16200,16300,16400,16500,16600,16700,16800,16900,17000,17100,17200,17300,17400,17500,17600,17700,17800,17900,18000,18100,18200,18300,18400,18500,18600,18700,18800,18900,19000,19100,19200,19300,19400,19500,19600,19700,19800,19900,20000,20100,20200,20300,20400,20500,20600,20700,20800,20900,21000,21100,21200,21300,21400,21500,21600,21700,21800,21900,22000,22100,22200,22300,22400,22500,22600,22700,22800,22900,23000,23100,23200,23300,23400,23500,23600,23700,23800,23900,24000,24100,24200,24300,24400,24500,24600,24700,24800,24900,25000,25100,25200,25300,25400,25500,25600,25700,25800,25900,26000,26100,26200,26300,26400,26500,26600,26700,26800,26900,27000,27100,27200,27300,27400,27500,27600,27700,27800,27900,28000,28100,28200,28300,28400,28500,28600,28700,28800,28900,29000,29100,29200,29300,29400,29500,29600
NW_003726780_1,Count,0,0,0,0,0,0,0,0,0,0,0,0,0,0,0,0,0,0,0,0,0,0,0,0,0,0,0,0,0,0,0,0,0,0,0,0,0,0,0,0,0,0,0,0,0,0,0,0,0,0,0,0,0,0,0,0,0,0,0,0,0,0,0,0,0,0,0,0,0,0,0,0,0,0,0,0,0,0,0,0,0,0,0,0,0,0,0,0,0,0,0,0,0,0,0,0,0,0,0,0,0,0,0,0,0,0,0,0,0,0,0,0,0,0,0,0,0,0,0,0,0,0,0,0,0,0,0,0,0,0,0,0,0,0,0,0,0,0,0,0,0,0,0,0,0,0,0,0,0,0,0,0,0,0,0,0,0,0,0,0,0,0,0,0,0,0,0,0,0,0,0,0,0,0,0,0,0,0,0,0,0,0,0,0,0,0,0,0,0,0,0,0,0,0,0,0,0,0,0,0,0,0,0,0,0,0,0,0,0,0,0,0,0,0,0,0,0,0,0,0,0,0,0,0,0,0,0,0,2,0,0,0,0,0,0,0,0,0,0,0,0,0,0,0,0,0,0,0,0,0,0,0,0,0,0,0,0,0,0,0,0,0,0,0,0,0,0,0,0,0,0,0,0,0,0,0,0,0,0,0,0,0,0,0,0,0,0,0,0,0,0,0,0,0,0,0,0

	
```

```
		  

		NW_003726783_1, Position,0,100,200,300,400,500,600,700,800,900,1000,1100,1200,1300,1400,1500,1600,1700,1800,1900,2000,2100,2200,2300,2400,2500,2600,2700,2800,2900,3000,3100,3200,3300,3400,3500,3600,3700,3800,3900,4000,4100,4200,4300,4400,4500,4600,4700,4800,4900,5000,5100,5200,5300,5400,5500,5600,5700,5800,5900,6000,6100,6200,6300,6400,6500,6600,6700,6800,6900,7000,7100,7200,7300,7400,7500,7600,7700,7800,7900,8000,8100,8200,8300,8400,8500,8600,8700,8800,8900,9000,9100,9200,9300,9400,9500,9600,9700,9800,9900,10000,10100,10200,10300,10400,10500,10600,10700,10800,10900,11000,11100,11200,11300,11400,11500,11600,11700,11800,11900,12000,12100,12200,12300,12400,12500,12600,12700,12800,12900,13000,13100,13200,13300,13400,13500,13600,13700,13800,13900,14000,14100,14200,14300,14400,14500,14600,14700,14800,14900,15000,15100,15200,15300,15400,15500,15600,15700,15800,15900,16000,16100,16200,16300,16400,16500,16600,16700,16800,16900,17000,17100,17200,17300,17400,17500,17600,17700,17800,17900,18000,18100,18200,18300,18400,18500,18600,18700,18800,18900,19000,19100,19200,19300,19400,19500,19600,19700,19800,19900,20000
NW_003726783_1,Count,0,0,0,0,0,0,0,0,0,0,0,0,0,0,0,0,0,0,0,0,0,0,0,0,0,0,0,0,0,0,0,0,0,0,0,0,0,0,0,0,4,2,0,0,1,2,1,0,1,1,1,1,0,1,2,1,1,1,1,0,0,1,2,0,2,0,1,1,1,0,1,0,2,1,1,1,2,1,1,1,2,1,4,1,2,0,0,1,1,1,1,1,0,1,2,2,1,2,0,0,3,1,0,3,0,0,1,1,3,0,0,0,0,0,0,0,1,0,2,0,2,1,2,2,0,0,0,2,2,1,3,0,1,1,1,0,3,0,0,0,0,2,2,2,0,1,0,1,1,1,0,0,0,1,2,0,1,1,1,0,0,2,1,0,0,0,1,0,1,3,0,3,2,1,0,3,0,1,0,0,0,0,0,2,1,0,1,0,0,0,0,0,0,0,0,0,0,0,0,0,0

	
```

```
		  

		NW_003726786_1, Position,0,100,200,300,400,500,600,700,800,900,1000,1100,1200,1300,1400,1500,1600,1700,1800,1900,2000,2100,2200,2300,2400,2500,2600,2700,2800,2900,3000,3100,3200,3300,3400,3500,3600,3700,3800,3900,4000,4100,4200,4300,4400,4500,4600,4700,4800,4900,5000,5100,5200,5300,5400,5500,5600,5700,5800,5900,6000,6100,6200,6300,6400,6500,6600,6700,6800,6900,7000,7100,7200,7300,7400,7500,7600,7700,7800,7900,8000,8100,8200,8300,8400,8500,8600,8700,8800,8900,9000,9100,9200,9300,9400,9500,9600,9700,9800,9900,10000,10100,10200,10300,10400,10500,10600,10700,10800,10900,11000,11100,11200,11300,11400,11500,11600,11700,11800,11900,12000,12100,12200,12300,12400,12500,12600,12700,12800,12900,13000,13100,13200,13300,13400,13500,13600,13700,13800,13900,14000,14100,14200,14300,14400,14500,14600,14700,14800,14900,15000,15100,15200,15300,15400,15500,15600,15700,15800,15900,16000,16100,16200,16300,16400,16500,16600,16700,16800,16900,17000,17100,17200,17300,17400,17500,17600,17700,17800,17900,18000,18100,18200,18300,18400,18500,18600,18700,18800,18900,19000,19100,19200,19300,19400,19500,19600,19700,19800,19900
NW_003726786_1,Count,0,0,0,0,0,0,0,0,0,0,0,0,0,0,0,0,0,0,0,0,0,0,0,0,0,0,0,0,0,0,0,0,0,0,0,0,0,0,0,0,0,0,0,0,0,0,0,0,0,0,0,0,0,0,0,0,0,0,0,0,0,0,0,0,0,0,0,0,0,0,0,0,0,0,0,0,0,0,0,0,0,0,0,0,0,0,0,0,0,0,0,0,0,0,0,0,0,0,0,0,0,0,0,0,0,0,0,0,0,0,0,0,0,0,0,0,0,0,0,0,0,0,0,0,0,0,0,0,0,0,0,0,0,0,0,0,0,0,0,0,0,0,0,0,0,0,0,0,0,0,0,0,0,0,0,0,0,0,0,0,0,0,0,0,0,0,0,0,0,0,0,0,0,0,0,0,0,0,0,0,0,0,0,0,0,0,0,0,0,0,0,0,0,0,0,0,3,1,0,0

	
```

```
		  

		NW_003726787_1, Position,0,100,200,300,400,500,600,700,800,900,1000,1100,1200,1300,1400,1500,1600,1700,1800,1900,2000,2100,2200,2300,2400,2500,2600,2700,2800,2900,3000,3100,3200,3300,3400,3500,3600,3700,3800,3900,4000,4100,4200,4300,4400,4500,4600,4700,4800,4900,5000,5100,5200,5300,5400,5500,5600,5700,5800,5900,6000,6100,6200,6300,6400,6500,6600,6700,6800,6900,7000,7100,7200,7300,7400,7500,7600,7700,7800,7900,8000,8100,8200,8300,8400,8500,8600,8700,8800,8900,9000,9100,9200,9300,9400,9500,9600,9700,9800,9900,10000,10100,10200,10300,10400,10500,10600,10700,10800,10900,11000,11100,11200,11300,11400,11500,11600,11700,11800,11900,12000,12100,12200,12300,12400,12500,12600,12700,12800,12900,13000,13100,13200,13300,13400,13500,13600,13700,13800,13900,14000,14100,14200,14300,14400,14500,14600,14700,14800,14900,15000,15100,15200,15300,15400,15500,15600,15700,15800,15900,16000,16100,16200,16300,16400,16500,16600,16700,16800,16900,17000,17100,17200,17300,17400,17500,17600,17700,17800,17900,18000,18100,18200,18300,18400,18500,18600,18700,18800,18900,19000,19100,19200,19300,19400,19500,19600,19700,19800,19900
NW_003726787_1,Count,0,0,0,0,0,2,3,0,0,0,0,0,0,0,0,0,0,0,1,0,1,0,0,0,0,0,0,0,0,0,0,0,2,0,0,1,1,1,0,0,0,0,0,0,0,0,0,0,2,0,0,0,1,3,0,3,1,0,0,0,0,0,0,0,0,0,0,0,0,0,0,0,0,0,0,0,0,0,0,0,0,0,0,0,0,0,0,0,0,0,0,0,0,0,0,0,0,0,0,0,0,0,0,0,0,0,0,0,0,0,0,0,0,0,0,0,0,0,0,0,0,0,0,0,0,0,0,0,0,0,0,0,0,0,0,0,0,0,0,0,0,0,0,0,0,0,0,0,0,0,0,2,1,1,3,1,0,0,0,0,0,0,0,3,0,0,2,0,0,0,0,0,0,2,2,1,0,0,0,0,2,0,0,0,0,0,0,0,0,0,0,0,0,0,0,0,0,0,0,0

	
```

```
		  

		NW_003726791_1, Position,0,100,200,300,400,500,600,700,800,900,1000,1100,1200,1300,1400,1500,1600,1700,1800,1900,2000,2100,2200,2300,2400,2500,2600,2700,2800,2900,3000,3100,3200,3300,3400,3500,3600,3700,3800,3900,4000,4100,4200,4300,4400,4500,4600,4700,4800,4900,5000,5100,5200,5300,5400,5500,5600,5700,5800,5900,6000,6100,6200,6300,6400,6500,6600,6700,6800,6900,7000,7100,7200,7300,7400,7500,7600,7700,7800,7900,8000,8100,8200,8300,8400,8500,8600,8700,8800,8900,9000,9100,9200,9300,9400,9500,9600,9700,9800,9900,10000,10100,10200,10300,10400,10500,10600,10700,10800,10900,11000,11100,11200,11300,11400,11500,11600,11700,11800,11900,12000,12100,12200,12300,12400,12500,12600,12700,12800,12900,13000,13100,13200,13300,13400,13500,13600,13700,13800,13900,14000,14100,14200,14300,14400,14500,14600,14700,14800,14900,15000,15100,15200,15300,15400,15500,15600,15700,15800,15900,16000,16100,16200,16300,16400,16500,16600,16700,16800,16900,17000,17100,17200,17300,17400,17500,17600,17700,17800,17900,18000,18100,18200,18300,18400,18500,18600,18700,18800,18900,19000,19100,19200,19300,19400,19500,19600,19700,19800,19900,20000,20100,20200,20300,20400,20500,20600,20700,20800,20900,21000,21100,21200,21300,21400,21500,21600,21700,21800,21900,22000,22100,22200,22300,22400,22500,22600,22700,22800,22900,23000,23100,23200,23300,23400,23500,23600,23700,23800,23900,24000,24100,24200,24300,24400,24500,24600,24700,24800,24900,25000,25100,25200,25300,25400,25500,25600,25700,25800,25900,26000,26100,26200
NW_003726791_1,Count,0,2,1,0,0,2,2,0,1,0,1,1,0,0,0,0,0,0,0,0,0,0,0,0,0,0,0,0,0,0,0,0,0,0,0,0,0,0,0,1,0,0,0,1,0,1,0,1,0,0,0,1,0,0,0,0,0,0,0,0,0,0,0,0,0,0,0,0,0,0,1,0,0,0,0,1,0,0,0,0,0,0,0,0,0,0,0,0,0,0,0,0,0,0,0,0,0,0,0,0,0,0,0,0,0,0,0,0,0,0,0,0,0,0,0,0,0,0,0,0,0,0,0,0,0,0,0,0,0,0,0,0,0,0,0,0,0,0,0,0,0,0,0,0,0,0,0,0,0,0,0,0,0,0,0,0,0,0,0,0,0,0,0,0,0,0,0,0,0,0,0,0,0,0,0,0,0,0,0,0,0,0,0,0,0,0,0,0,0,0,0,0,0,0,0,0,0,0,0,0,0,0,0,0,0,0,0,0,0,0,0,0,0,0,0,0,0,0,0,0,0,0,0,0,0,0,0,0,0,0,0,0,0,0,0,0,0,0,0,0,0,0,0,0,0,0,0,0,0,0,0,0,0,0,0,0,0,0,0,0,0,0,0

	
```

```
		  

		NW_003726793_1, Position,0,100,200,300,400,500,600,700,800,900,1000,1100,1200,1300,1400,1500,1600,1700,1800,1900,2000,2100,2200,2300,2400,2500,2600,2700,2800,2900,3000,3100,3200,3300,3400,3500,3600,3700,3800,3900,4000,4100,4200,4300,4400,4500,4600,4700,4800,4900,5000,5100,5200,5300,5400,5500,5600,5700,5800,5900,6000,6100,6200,6300,6400,6500,6600,6700,6800,6900,7000,7100,7200,7300,7400,7500,7600,7700,7800,7900,8000,8100,8200,8300,8400,8500,8600,8700,8800,8900,9000,9100,9200,9300,9400,9500,9600,9700,9800,9900,10000,10100,10200,10300,10400,10500,10600,10700,10800,10900,11000,11100,11200,11300,11400,11500,11600,11700,11800,11900,12000,12100,12200,12300,12400,12500,12600,12700,12800,12900,13000,13100,13200,13300,13400,13500,13600,13700,13800,13900,14000,14100,14200,14300,14400,14500,14600,14700,14800,14900,15000,15100,15200,15300,15400,15500,15600,15700,15800,15900,16000,16100,16200,16300,16400,16500,16600,16700,16800,16900,17000,17100,17200,17300,17400,17500,17600,17700,17800,17900,18000,18100,18200,18300,18400,18500,18600,18700,18800,18900,19000,19100,19200,19300,19400,19500,19600,19700,19800,19900,20000,20100,20200,20300,20400,20500,20600,20700,20800,20900,21000,21100,21200,21300,21400
NW_003726793_1,Count,0,0,0,0,0,0,0,0,0,0,0,0,0,0,0,0,0,0,0,0,0,0,0,0,0,0,0,0,0,0,0,0,0,0,0,0,0,0,0,0,0,0,0,0,0,0,0,0,0,0,0,0,1,4,2,2,0,0,6,0,0,2,0,0,0,0,1,3,0,1,1,2,2,3,0,0,2,0,0,0,0,0,1,1,1,3,0,0,1,2,3,0,0,1,3,1,0,1,2,0,1,1,1,0,4,1,0,0,0,1,2,1,3,1,1,2,1,2,1,1,2,0,0,2,1,3,0,2,0,3,2,0,2,1,0,0,1,1,0,2,1,1,2,0,0,2,0,0,3,1,0,0,2,1,2,3,3,1,3,2,1,1,3,0,0,2,0,1,0,0,0,0,1,1,1,1,1,1,0,1,1,3,2,2,3,1,0,0,0,0,0,0,0,0,0,0,0,0,0,0,1,1,1,2,0,0,1,2,1,0,0,1,1,0,0

	
```

```
		  

		NW_003726799_1, Position,0,100,200,300,400,500,600,700,800,900,1000,1100,1200,1300,1400,1500,1600,1700,1800,1900,2000,2100,2200,2300,2400,2500,2600,2700,2800,2900,3000,3100,3200,3300,3400,3500,3600,3700,3800,3900,4000,4100,4200,4300,4400,4500,4600,4700,4800,4900,5000,5100,5200,5300,5400,5500,5600,5700,5800,5900,6000,6100,6200,6300,6400,6500,6600,6700,6800,6900,7000,7100,7200,7300,7400,7500,7600,7700,7800,7900,8000,8100,8200,8300,8400,8500,8600,8700,8800,8900,9000,9100,9200,9300,9400,9500,9600,9700,9800,9900,10000,10100,10200,10300,10400,10500,10600,10700,10800,10900,11000,11100,11200,11300,11400,11500,11600,11700,11800,11900,12000,12100,12200,12300,12400,12500,12600,12700,12800,12900,13000,13100,13200,13300,13400,13500,13600,13700,13800,13900,14000,14100,14200,14300,14400,14500,14600,14700,14800,14900,15000,15100,15200,15300,15400,15500,15600,15700,15800,15900,16000,16100,16200,16300,16400,16500,16600,16700,16800,16900,17000,17100,17200,17300,17400,17500,17600,17700,17800,17900,18000,18100,18200,18300,18400,18500,18600,18700,18800,18900,19000,19100,19200,19300,19400,19500,19600,19700,19800
NW_003726799_1,Count,0,0,0,0,0,0,0,0,0,0,0,0,0,0,0,0,0,0,0,0,0,0,0,0,0,0,0,0,0,0,0,0,0,0,0,0,0,0,0,0,0,0,0,0,0,0,0,0,0,0,0,0,0,0,0,0,0,0,0,0,0,0,0,0,0,0,0,0,0,0,0,0,0,0,0,0,0,0,0,0,0,0,0,0,0,0,0,0,0,0,0,0,0,1,3,1,0,0,0,0,0,0,0,0,0,0,0,0,0,0,0,0,0,0,0,0,0,0,0,0,0,0,0,0,0,0,0,0,0,0,0,0,0,0,0,0,0,0,0,0,0,0,0,0,0,0,0,0,0,0,0,0,0,0,0,0,0,0,0,1,0,0,0,0,0,0,0,0,0,0,0,0,0,0,0,0,0,0,0,0,0,0,0,0,0,0,0,0,0,0,0,0,0,0,0,0,0,0,0

	
```

```
		  

		NW_003726800_1, Position,0,100,200,300,400,500,600,700,800,900,1000,1100,1200,1300,1400,1500,1600,1700,1800,1900,2000,2100,2200,2300,2400,2500,2600,2700,2800,2900,3000,3100,3200,3300,3400,3500,3600,3700,3800,3900,4000,4100,4200,4300,4400,4500,4600,4700,4800,4900,5000,5100,5200,5300,5400,5500,5600,5700,5800,5900,6000,6100,6200,6300,6400,6500,6600,6700,6800,6900,7000,7100,7200,7300,7400,7500,7600,7700,7800,7900,8000,8100,8200,8300,8400,8500,8600,8700,8800,8900,9000,9100,9200,9300,9400,9500,9600,9700,9800,9900,10000,10100,10200,10300,10400,10500,10600,10700,10800,10900,11000,11100,11200,11300,11400,11500,11600,11700,11800,11900,12000,12100,12200,12300,12400,12500,12600,12700,12800,12900,13000,13100,13200,13300,13400,13500,13600,13700,13800,13900,14000,14100,14200,14300,14400,14500,14600,14700,14800,14900,15000,15100,15200,15300,15400,15500,15600,15700,15800,15900,16000,16100,16200,16300,16400,16500,16600,16700,16800,16900,17000,17100,17200,17300,17400,17500,17600,17700,17800,17900,18000,18100,18200,18300,18400,18500,18600,18700,18800,18900,19000,19100,19200,19300,19400,19500,19600,19700,19800,19900,20000,20100,20200,20300,20400,20500,20600,20700,20800,20900,21000,21100,21200,21300,21400,21500,21600,21700,21800,21900,22000,22100,22200
NW_003726800_1,Count,0,0,0,0,0,0,0,0,0,0,0,0,0,0,0,0,0,0,0,0,0,0,0,0,0,0,0,0,0,0,0,0,0,0,0,0,0,0,0,0,0,0,0,0,0,0,0,0,0,0,0,0,0,0,0,0,0,0,0,0,0,0,0,0,0,0,0,0,0,0,0,0,0,0,0,0,0,0,0,0,0,0,0,0,0,0,0,0,0,0,0,1,0,0,0,0,0,0,0,0,0,0,0,0,0,0,0,0,0,0,0,0,0,0,0,0,0,0,0,0,0,0,0,0,0,0,0,0,0,0,0,0,0,0,0,0,0,0,0,0,0,0,0,0,0,0,0,0,0,0,0,0,0,0,0,0,0,0,0,0,0,0,0,0,0,0,0,0,0,0,0,0,0,0,0,0,0,0,0,0,0,0,0,0,0,0,0,0,0,0,0,0,0,0,0,0,0,0,0,0,0,0,0,0,0,0,0,0,0,0,0,0,0,0,0,0,0,0,0,0,0,0,0

	
```

```
		  

		NW_003726807_1, Position,0,100,200,300,400,500,600,700,800,900,1000,1100,1200,1300,1400,1500,1600,1700,1800,1900,2000,2100,2200,2300,2400,2500,2600,2700,2800,2900,3000,3100,3200,3300,3400,3500,3600,3700,3800,3900,4000,4100,4200,4300,4400,4500,4600,4700,4800,4900,5000,5100,5200,5300,5400,5500,5600,5700,5800,5900,6000,6100,6200,6300,6400,6500,6600,6700,6800,6900,7000,7100,7200,7300,7400,7500,7600,7700,7800,7900,8000,8100,8200,8300,8400,8500,8600,8700,8800,8900,9000,9100,9200,9300,9400,9500,9600,9700,9800,9900,10000,10100,10200,10300,10400,10500,10600,10700,10800,10900,11000,11100,11200,11300,11400,11500,11600,11700,11800,11900,12000,12100,12200,12300,12400,12500,12600,12700,12800,12900,13000,13100,13200,13300,13400,13500,13600,13700,13800,13900,14000,14100,14200,14300,14400,14500,14600,14700,14800,14900,15000,15100,15200,15300,15400,15500,15600,15700,15800,15900,16000,16100,16200,16300,16400,16500,16600,16700,16800,16900,17000,17100,17200,17300,17400,17500,17600,17700,17800,17900,18000,18100,18200,18300,18400,18500,18600,18700,18800,18900,19000,19100,19200,19300,19400,19500,19600,19700,19800,19900,20000,20100,20200,20300,20400,20500,20600
NW_003726807_1,Count,0,0,0,0,0,0,0,0,0,0,0,0,0,0,0,0,0,0,0,0,0,0,0,0,0,0,0,0,0,0,0,0,0,0,0,0,0,0,0,0,0,0,0,0,0,0,0,0,0,0,0,0,0,0,2,0,0,0,0,0,0,0,0,0,0,0,0,0,0,0,0,0,0,0,0,0,0,0,0,0,0,0,0,0,0,0,0,0,0,0,0,0,0,0,0,0,0,0,0,0,0,0,0,0,0,0,0,0,0,0,0,0,0,0,0,0,0,0,0,0,0,0,0,0,0,0,0,0,0,0,0,0,0,0,0,0,0,0,0,0,0,0,0,0,0,0,0,0,0,0,0,0,0,0,0,0,0,0,0,0,0,0,0,0,0,0,0,0,0,0,0,0,0,0,0,0,0,0,0,0,0,0,0,0,0,0,0,0,0,0,0,0,0,0,0,0,0,0,0,0,0,0,0,0,0,0,0

	
```

```
		  

		NW_003726810_1, Position,0,100,200,300,400,500,600,700,800,900,1000,1100,1200,1300,1400,1500,1600,1700,1800,1900,2000,2100,2200,2300,2400,2500,2600,2700,2800,2900,3000,3100,3200,3300,3400,3500,3600,3700,3800,3900,4000,4100,4200,4300,4400,4500,4600,4700,4800,4900,5000,5100,5200,5300,5400,5500,5600,5700,5800,5900,6000,6100,6200,6300,6400,6500,6600,6700,6800,6900,7000,7100,7200,7300,7400,7500,7600,7700,7800,7900,8000,8100,8200,8300,8400,8500,8600,8700,8800,8900,9000,9100,9200,9300,9400,9500,9600,9700,9800,9900,10000,10100,10200,10300,10400,10500,10600,10700,10800,10900,11000,11100,11200,11300,11400,11500,11600,11700,11800,11900,12000,12100,12200,12300,12400,12500,12600,12700,12800,12900,13000,13100,13200,13300,13400,13500,13600,13700,13800,13900,14000,14100,14200,14300,14400,14500,14600,14700,14800,14900,15000,15100,15200,15300,15400,15500,15600,15700,15800,15900,16000,16100,16200,16300,16400,16500,16600,16700,16800,16900,17000,17100,17200,17300,17400,17500,17600,17700,17800,17900,18000,18100,18200,18300,18400,18500,18600,18700,18800,18900,19000,19100,19200,19300,19400,19500,19600,19700,19800
NW_003726810_1,Count,0,0,0,0,0,0,0,0,0,0,0,0,0,2,0,0,0,1,0,0,2,0,0,0,0,0,0,0,0,0,0,0,0,0,0,0,0,0,0,0,0,0,0,0,0,1,3,0,2,0,0,0,0,0,0,0,0,0,0,0,0,0,0,0,0,0,0,0,0,0,0,0,0,0,0,0,0,0,0,0,0,0,0,0,0,0,0,0,0,1,2,1,0,0,0,0,0,0,0,0,0,0,0,0,0,0,0,0,0,0,0,0,0,0,0,0,0,0,0,0,0,0,0,0,0,0,0,0,0,0,0,0,0,0,0,0,0,0,0,3,1,0,1,1,1,2,0,0,0,0,0,0,0,1,0,3,0,0,2,0,0,0,0,0,0,0,0,0,0,0,0,0,0,0,1,3,1,0,0,0,0,0,0,0,0,0,0,0,0,0,0,0,0,0,0,0,0,0,0

	
```

```
		  

		NW_003726813_1, Position,0,100,200,300,400,500,600,700,800,900,1000,1100,1200,1300,1400,1500,1600,1700,1800,1900,2000,2100,2200,2300,2400,2500,2600,2700,2800,2900,3000,3100,3200,3300,3400,3500,3600,3700,3800,3900,4000,4100,4200,4300,4400,4500,4600,4700,4800,4900,5000,5100,5200,5300,5400,5500,5600,5700,5800,5900,6000,6100,6200,6300,6400,6500,6600,6700,6800,6900,7000,7100,7200,7300,7400,7500,7600,7700,7800,7900,8000,8100,8200,8300,8400,8500,8600,8700,8800,8900,9000,9100,9200,9300,9400,9500,9600,9700,9800,9900,10000,10100,10200,10300,10400,10500,10600,10700,10800,10900,11000,11100,11200,11300,11400,11500,11600,11700,11800,11900,12000,12100,12200,12300,12400,12500,12600,12700,12800,12900,13000,13100,13200,13300,13400,13500,13600,13700,13800,13900,14000,14100,14200,14300,14400,14500,14600,14700,14800,14900,15000,15100,15200,15300,15400,15500,15600,15700,15800,15900,16000,16100,16200,16300,16400,16500,16600,16700,16800,16900,17000,17100,17200,17300,17400,17500,17600,17700,17800,17900,18000,18100,18200,18300,18400,18500,18600,18700,18800,18900,19000,19100,19200,19300,19400
NW_003726813_1,Count,0,0,0,0,0,0,0,0,0,0,0,0,0,0,0,0,0,0,0,0,0,0,0,0,0,0,0,0,0,0,0,0,0,0,0,0,0,0,0,0,0,0,0,0,0,0,0,0,0,0,0,0,0,1,0,0,0,0,0,0,0,0,0,0,0,0,0,0,0,0,0,0,0,0,0,0,0,0,0,0,0,0,0,0,0,0,0,0,0,0,0,0,0,0,0,0,0,0,0,0,0,0,0,2,1,2,0,0,1,0,0,0,0,0,0,0,0,0,3,1,6,3,0,3,0,0,1,2,0,0,0,0,0,0,0,0,0,0,0,0,0,0,0,0,0,0,0,0,0,0,0,0,0,0,0,0,0,0,0,0,0,0,0,0,0,0,0,0,0,0,0,0,0,0,0,0,0,1,0,0,2,0,0,0,0,0,0,0,0,0,0,0,0,0,0

	
```

```
		  

		NW_003726817_1, Position,0,1000,2000,3000,4000,5000,6000,7000,8000,9000,10000,11000,12000,13000,14000,15000,16000,17000,18000,19000,20000,21000,22000,23000,24000,25000,26000,27000,28000,29000,30000,31000,32000,33000
NW_003726817_1,Count,0,0,0,0,0,0,0,0,0,0,0,0,0,0,0,0,1,0,0,0,0,0,0,0,0,0,0,0,0,0,0,0,0,0

	
```

```
		  

		NW_003726819_1, Position,0,1000,2000,3000,4000,5000,6000,7000,8000,9000,10000,11000,12000,13000,14000,15000,16000,17000,18000,19000,20000,21000,22000,23000,24000,25000,26000,27000,28000,29000,30000,31000,32000,33000,34000,35000,36000,37000,38000,39000,40000,41000,42000,43000,44000,45000,46000,47000,48000,49000,50000,51000,52000,53000
NW_003726819_1,Count,0,0,0,0,0,0,0,0,0,0,0,0,0,0,0,0,0,0,0,0,0,0,0,0,0,0,0,0,0,0,0,0,0,0,0,0,0,0,0,0,0,0,0,0,0,0,0,0,0,0,0,0,4,0

	
```

```
		  

		NW_003726820_1, Position,0,100,200,300,400,500,600,700,800,900,1000,1100,1200,1300,1400,1500,1600,1700,1800,1900,2000,2100,2200,2300,2400,2500,2600,2700,2800,2900,3000,3100,3200,3300,3400,3500,3600,3700,3800,3900,4000,4100,4200,4300,4400,4500,4600,4700,4800,4900,5000,5100,5200,5300,5400,5500,5600,5700,5800,5900,6000,6100,6200,6300,6400,6500,6600,6700,6800,6900,7000,7100,7200,7300,7400,7500,7600,7700,7800,7900,8000,8100,8200,8300,8400,8500,8600,8700,8800,8900,9000,9100,9200,9300,9400,9500,9600,9700,9800,9900,10000,10100,10200,10300,10400,10500,10600,10700,10800,10900,11000,11100,11200,11300,11400,11500,11600,11700,11800,11900,12000,12100,12200,12300,12400,12500,12600,12700,12800,12900,13000,13100,13200,13300,13400,13500,13600,13700,13800,13900,14000,14100,14200,14300,14400,14500,14600,14700,14800,14900,15000,15100,15200,15300,15400,15500,15600,15700,15800,15900,16000,16100,16200,16300,16400,16500,16600,16700,16800,16900,17000,17100,17200,17300,17400,17500,17600,17700,17800,17900,18000,18100,18200,18300,18400,18500,18600,18700,18800,18900,19000,19100,19200,19300
NW_003726820_1,Count,0,0,0,0,4,1,0,0,0,0,0,2,2,2,1,0,0,0,1,1,1,1,2,1,0,1,1,0,1,0,2,0,1,1,1,1,0,1,1,1,0,0,1,1,0,0,0,1,0,1,1,0,2,0,1,0,0,1,1,2,1,1,0,2,1,1,3,3,2,2,0,3,0,1,1,0,0,1,1,0,0,1,2,1,0,1,1,1,0,1,1,0,2,0,0,0,1,1,2,2,1,0,1,3,1,0,1,2,1,1,0,0,0,2,2,1,0,2,1,1,3,2,3,2,3,2,1,2,3,2,1,1,0,1,2,1,1,1,1,0,1,0,2,1,1,1,0,0,3,2,0,0,0,1,0,2,0,0,2,1,0,2,0,1,0,2,0,0,0,0,0,0,0,0,0,0,0,0,0,0,0,0,0,0,0,0,0,0,0,0,0,0,0,0

	
```

```
		  

		NW_003726823_1, Position,0,100,200,300,400,500,600,700,800,900,1000,1100,1200,1300,1400,1500,1600,1700,1800,1900,2000,2100,2200,2300,2400,2500,2600,2700,2800,2900,3000,3100,3200,3300,3400,3500,3600,3700,3800,3900,4000,4100,4200,4300,4400,4500,4600,4700,4800,4900,5000,5100,5200,5300,5400,5500,5600,5700,5800,5900,6000,6100,6200,6300,6400,6500,6600,6700,6800,6900,7000,7100,7200,7300,7400,7500,7600,7700,7800,7900,8000,8100,8200,8300,8400,8500,8600,8700,8800,8900,9000,9100,9200,9300,9400,9500,9600,9700,9800,9900,10000,10100,10200,10300,10400,10500,10600,10700,10800,10900,11000,11100,11200,11300,11400,11500,11600,11700,11800,11900,12000,12100,12200,12300,12400,12500,12600,12700,12800,12900,13000,13100,13200,13300,13400,13500,13600,13700,13800,13900,14000,14100,14200,14300,14400,14500,14600,14700,14800,14900,15000,15100,15200,15300,15400,15500,15600,15700,15800,15900,16000,16100,16200,16300,16400,16500,16600,16700,16800,16900,17000,17100,17200,17300,17400,17500,17600,17700,17800,17900,18000,18100,18200,18300,18400,18500,18600,18700,18800,18900,19000,19100,19200,19300,19400,19500,19600,19700,19800,19900,20000,20100,20200,20300,20400,20500,20600,20700,20800,20900,21000,21100,21200,21300,21400,21500,21600,21700,21800,21900,22000,22100,22200,22300,22400,22500,22600,22700,22800,22900,23000,23100,23200,23300,23400,23500,23600,23700,23800,23900,24000,24100,24200,24300,24400,24500,24600,24700,24800,24900,25000,25100,25200,25300,25400,25500,25600,25700,25800,25900,26000,26100,26200,26300,26400,26500,26600,26700,26800,26900,27000,27100,27200,27300,27400,27500,27600,27700,27800,27900,28000,28100,28200,28300,28400
NW_003726823_1,Count,0,0,0,0,0,0,0,0,0,0,0,0,0,0,0,0,0,0,0,0,0,0,0,0,0,0,0,0,0,0,0,0,0,0,0,0,0,0,0,0,0,0,0,0,0,0,0,0,0,0,0,0,0,0,0,0,0,0,0,0,0,0,0,0,0,0,0,0,0,0,0,0,0,0,0,0,0,0,0,2,1,0,0,1,1,0,1,0,0,1,3,2,1,2,1,0,0,0,0,0,0,0,0,0,0,0,0,0,0,0,0,0,0,0,0,0,0,0,0,0,0,0,0,0,0,0,0,0,0,0,0,0,0,0,0,0,0,0,0,0,0,0,0,0,0,0,0,0,0,0,0,0,0,0,0,0,0,0,0,0,0,0,0,0,0,0,0,0,0,0,0,0,0,0,0,0,0,0,0,0,0,0,0,0,0,0,0,0,0,0,0,0,0,0,0,0,0,0,0,0,0,0,0,0,0,0,0,0,0,0,0,0,0,0,0,0,0,0,0,0,0,0,0,0,0,0,0,0,0,0,0,0,1,3,1,0,0,0,0,0,0,0,0,0,0,0,0,0,2,1,0,0,0,0,0,0,0,0,0,0,0,0,0,0,0,0,0,0,0,0,0,0,0,0,0,0,0,0,0,0,0,0,0,0,0

	
```

```
		  

		NW_003726826_1, Position,0,100,200,300,400,500,600,700,800,900,1000,1100,1200,1300,1400,1500,1600,1700,1800,1900,2000,2100,2200,2300,2400,2500,2600,2700,2800,2900,3000,3100,3200,3300,3400,3500,3600,3700,3800,3900,4000,4100,4200,4300,4400,4500,4600,4700,4800,4900,5000,5100,5200,5300,5400,5500,5600,5700,5800,5900,6000,6100,6200,6300,6400,6500,6600,6700,6800,6900,7000,7100,7200,7300,7400,7500,7600,7700,7800,7900,8000,8100,8200,8300,8400,8500,8600,8700,8800,8900,9000,9100,9200,9300,9400,9500,9600,9700,9800,9900,10000,10100,10200,10300,10400,10500,10600,10700,10800,10900,11000,11100,11200,11300,11400,11500,11600,11700,11800,11900,12000,12100,12200,12300,12400,12500,12600,12700,12800,12900,13000,13100,13200,13300,13400,13500,13600,13700,13800,13900,14000,14100,14200,14300,14400,14500,14600,14700,14800,14900,15000,15100,15200,15300,15400,15500,15600,15700,15800,15900,16000,16100,16200,16300,16400,16500,16600,16700,16800,16900,17000,17100,17200,17300,17400,17500,17600,17700,17800,17900,18000,18100,18200,18300,18400,18500,18600,18700,18800,18900,19000,19100,19200
NW_003726826_1,Count,1,0,0,2,1,0,1,0,0,0,0,1,0,1,0,1,2,0,0,0,0,0,1,0,0,1,1,0,0,1,0,0,1,1,1,0,1,0,0,0,0,0,2,1,2,0,0,0,0,0,0,0,0,0,1,0,0,0,1,0,0,0,0,0,0,1,0,0,0,0,0,0,0,1,0,0,0,0,3,0,0,0,0,0,0,0,0,1,2,1,1,2,0,0,0,0,0,0,0,1,0,0,0,0,0,1,0,0,0,1,0,0,0,0,0,2,0,1,0,1,1,0,0,0,0,2,0,1,0,0,0,1,2,0,0,2,0,0,0,0,0,0,0,0,0,0,0,0,0,0,0,0,0,0,0,0,0,0,0,0,0,0,0,0,0,0,0,0,0,0,1,0,1,1,3,0,0,0,1,0,2,0,0,2,0,1,1,1,0,0,0,0,0

	
```

```
		  

		NW_003726830_1, Position,0,100,200,300,400,500,600,700,800,900,1000,1100,1200,1300,1400,1500,1600,1700,1800,1900,2000,2100,2200,2300,2400,2500,2600,2700,2800,2900,3000,3100,3200,3300,3400,3500,3600,3700,3800,3900,4000,4100,4200,4300,4400,4500,4600,4700,4800,4900,5000,5100,5200,5300,5400,5500,5600,5700,5800,5900,6000,6100,6200,6300,6400,6500,6600,6700,6800,6900,7000,7100,7200,7300,7400,7500,7600,7700,7800,7900,8000,8100,8200,8300,8400,8500,8600,8700,8800,8900,9000,9100,9200,9300,9400,9500,9600,9700,9800,9900,10000,10100,10200,10300,10400,10500,10600,10700,10800,10900,11000,11100,11200,11300,11400,11500,11600,11700,11800,11900,12000,12100,12200,12300,12400,12500,12600,12700,12800,12900,13000,13100,13200,13300,13400,13500,13600,13700,13800,13900,14000,14100,14200,14300,14400,14500,14600,14700,14800,14900,15000,15100,15200,15300,15400,15500,15600,15700,15800,15900,16000,16100,16200,16300,16400,16500,16600,16700,16800,16900,17000,17100,17200,17300,17400,17500,17600,17700,17800,17900,18000,18100,18200,18300,18400,18500,18600,18700,18800,18900,19000,19100
NW_003726830_1,Count,0,2,1,3,1,2,0,1,1,1,1,0,0,1,2,2,0,0,0,2,0,0,2,2,2,2,1,1,0,3,2,2,2,2,0,2,2,2,1,2,0,3,2,1,0,0,2,1,2,1,0,0,0,0,0,3,1,3,1,3,2,0,0,0,0,0,1,0,0,0,0,3,0,2,1,0,2,2,0,0,0,0,0,3,1,1,1,0,0,0,1,1,0,0,0,0,2,0,3,0,3,2,1,0,2,1,2,1,1,1,2,0,1,1,0,0,0,0,1,3,0,1,0,1,1,0,1,0,1,1,0,0,2,1,2,0,0,0,0,1,1,1,0,1,1,1,2,2,0,1,0,0,1,0,2,2,1,2,0,1,3,0,0,0,0,2,2,1,2,2,1,0,3,2,1,1,0,0,0,0,1,2,0,1,2,1,2,3,1,3,0,2

	
```

```
[truncated: 329,722 more chars]
